# Supplementary material for: A Protein Misfolding Shaking Amplification-based method for the spontaneous generation of hundreds of bona fide prions
Source: Nat Commun. 2024 Mar 8;15:2112. doi: 10.1038/s41467-024-46360-2 (PMC10923866; doi:10.1038/s41467-024-46360-2)
Supplement: Supplementary file 1 — Supplementary Information [file 41467_2024_46360_MOESM1_ESM.pdf]

A Protein Misfolding Shaking Amplification-based method for the spontaneous generation of hundreds of infectious prions

Hasier Eraña[1,2,3], Cristina Sampedro-Torres-Quevedo[1], Jorge M. Charco[1,2,3], Carlos M. Díaz-Domínguez[1,2], Francesca Peccati[1], Maitena San-Juan-Ansoleaga[1], Enric Vidal[4,5], Nuno Gonçalves-Anjo[1], Miguel A. Pérez-Castro[1], Ezequiel González-Miranda[1], Patricia Piñeiro[1], Leire Fernández-Veiga[1], Josu Galarza-Ahumada[1], Eva Fernández-Muñoz[1], Guiomar Perez de Nanclares[6], Glenn Telling[7], Mariví Geijo[8], Gonzalo Jiménez-Osés[1,9] and Joaquín Castilla[1,2,9*]

[1] Center for Cooperative Research in Biosciences (CIC BioGUNE), Basque Research and Technology Alliance (BRTA), Derio, Spain.

[2] Centro de Investigación Biomédica en Red de Enfermedades infecciosas (CIBERINFEC), Carlos III National Health Institute, Madrid, Spain.

[3] ATLAS Molecular Pharma S. L., Derio, Spain.

[4] IRTA. Programa de Sanitat Animal. Centre de Recerca en Sanitat Animal (CReSA). Campus de la Universitat Autònoma de Barcelona (UAB), Bellaterra, Catalonia. Spain.

[5] Unitat mixta d'Investigació IRTA-UAB en Sanitat Animal. Centre de Recerca en Sanitat Animal (CReSA). Campus de la Universitat Autònoma de Barcelona (UAB), Bellaterra, Catalonia. Spain.

[6] Molecular (Epi)Genetics Laboratory, Bioaraba Health Research Institute, Araba University Hospital, Vitoria-Gasteiz, Spain.

[7] Prion Research Center. Colorado State University, Fort Collins, USA.

[8]Animal Health Department, NEIKER-Basque Institute for Agricultural Research and Development. Basque Research and Technology Alliance (BRTA), Derio, Spain.

[9] IKERBASQUE, Basque Foundation for Science, Bilbao, Spain.

*Corresponding author: Joaquín Castilla; jcastilla@cicbiogune.es

SUPPLEMENTARY INFORMATION

Supplementary Fig. 1

Supplementary Fig. 2

Supplementary Fig. 3

Supplementary Fig. 4

Supplementary Methods

- **Recombinant prion protein expression and purification**
- **Substrate preparation for Protein Misfolding Shaking Amplification**
- **Protein Misfolding Shaking Amplification**
- **Purification of rec-PrP^{res} to be used as seeds in PMCA**
- **List of reagents, company name and catalogue numbers**

Supplementary Table 1

Supplementary Table 2

Supplementary Table 3

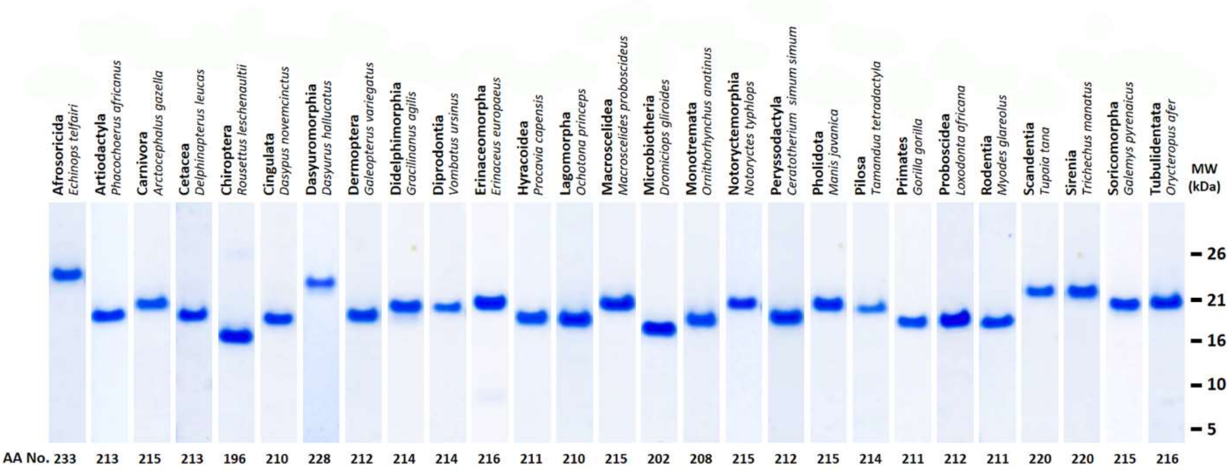

Supplementary figure 1. Illustrative example of PMSA substrates visualized by electrophoresis and total protein staining, prepared for the assessment of their spontaneous misfolding capacity through PMSA. Substrates containing 27 different rec-PrP variants from mammal species belonging to each distinct order studied are shown after their analysis by electrophoresis and total protein staining. The gel image illustrates that all prepared substrates present similar rec-PrP concentrations and the absence of detectable multimers or protein fragments. This indicates the overall high protein quality achieved through our production, purification, and substrate preparation procedures. Below the gel, the number of amino acids of each protein is indicated, confirming their expected size according to the molecular weight marker (MW) used for reference. This alignment was performed by comparing the original gels used as quality control of each substrate, which were consistently run alongside the same molecular weight marker. Additionally, to minimize variability derived from the unavoidable inter-gel comparison, PMSA substrates were run in groups of 8 proteins per gel, with 3-4 gels processed in parallel, making 24-32 proteins processed in parallel each time. Original, uncropped gels are publicly available in Zenodo repository (10.5281/zenodo.10579518) [1], as detailed in Data Availability section.

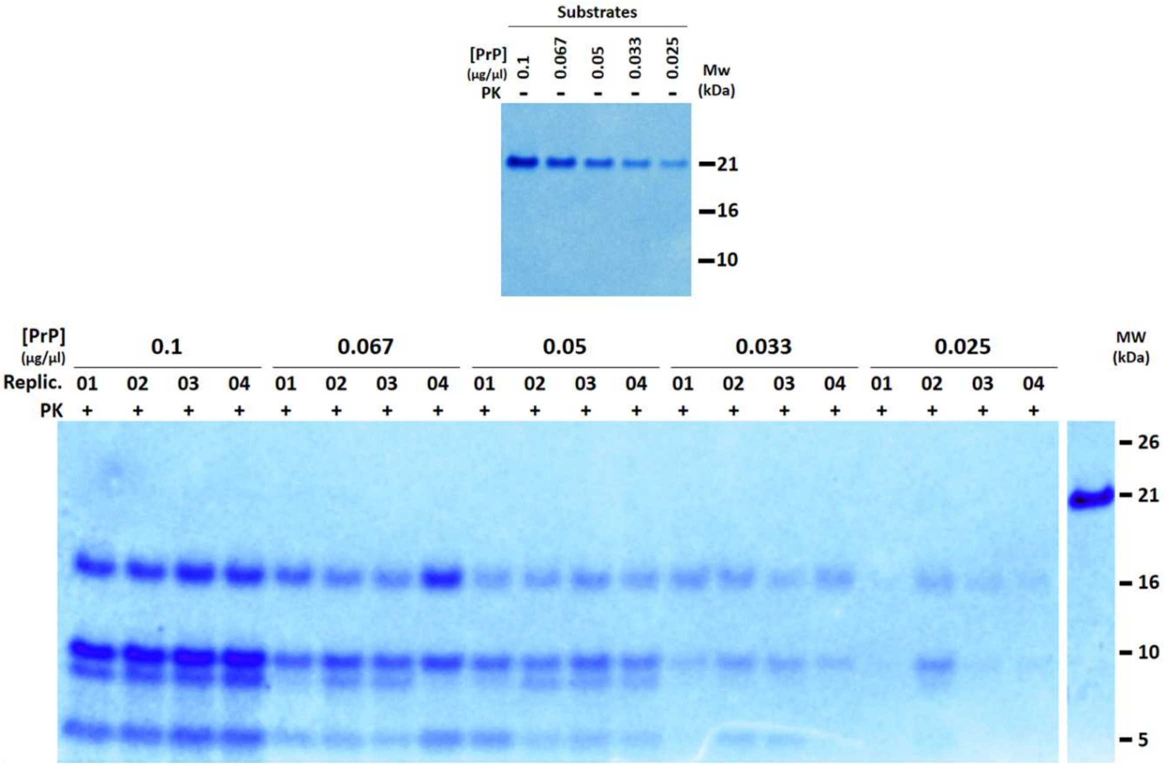

Supplementary figure 2. Assessment of rec-PrP concentration range in PMSA substrate required for reproducible evaluation of spontaneous misfolding capacity. To determine the range of rec-PrP concentration within a PMSA substrate that allows consistent results in terms of spontaneous misfolding capacity, substrates containing distinct bank vole rec-PrP concentrations (0.1, 0.067, 0.05, 0.033 and 0.025 µg/µl) were prepared, visualized through electrophoresis and total protein staining (upper gel) and subjected to a single 24 h PMSA round. Given the influence of the rec-PrP concentration on its spontaneous misfolding capacity, determining a range that allows consistent results in terms of misfolding is convenient to avoid precise quantitative measurement of the rec-PrP concentration of each of the PMSA substrates prepared for the study. 4 replicates of each substrate were used to evaluate misfolding (n=4), and as shown in the gel at 0.033 µg/µl all the tubes are positive albeit with a lower signal and even at 0.025 µg/µl, with four times less rec-PrP in the substrate three out of four tubes are clearly positive and the fourth shows a very faint signal that would undoubtedly be positive in a second PMSA round. Therefore, we can conclude that in a range of 0.1 to 0.025 µg/µl of rec-PrP in the substrate, spontaneous misfolding score is not significantly altered, allowing to work with all the different substrates prepared throughout the study such as the ones included in Supplementary figure 1, that in any case show greater rec-PrP concentration differences than those tested here. Original, uncropped gels are publicly available in Zenodo repository (10.5281/zenodo.10579518) [1], as detailed in Data Availability section.

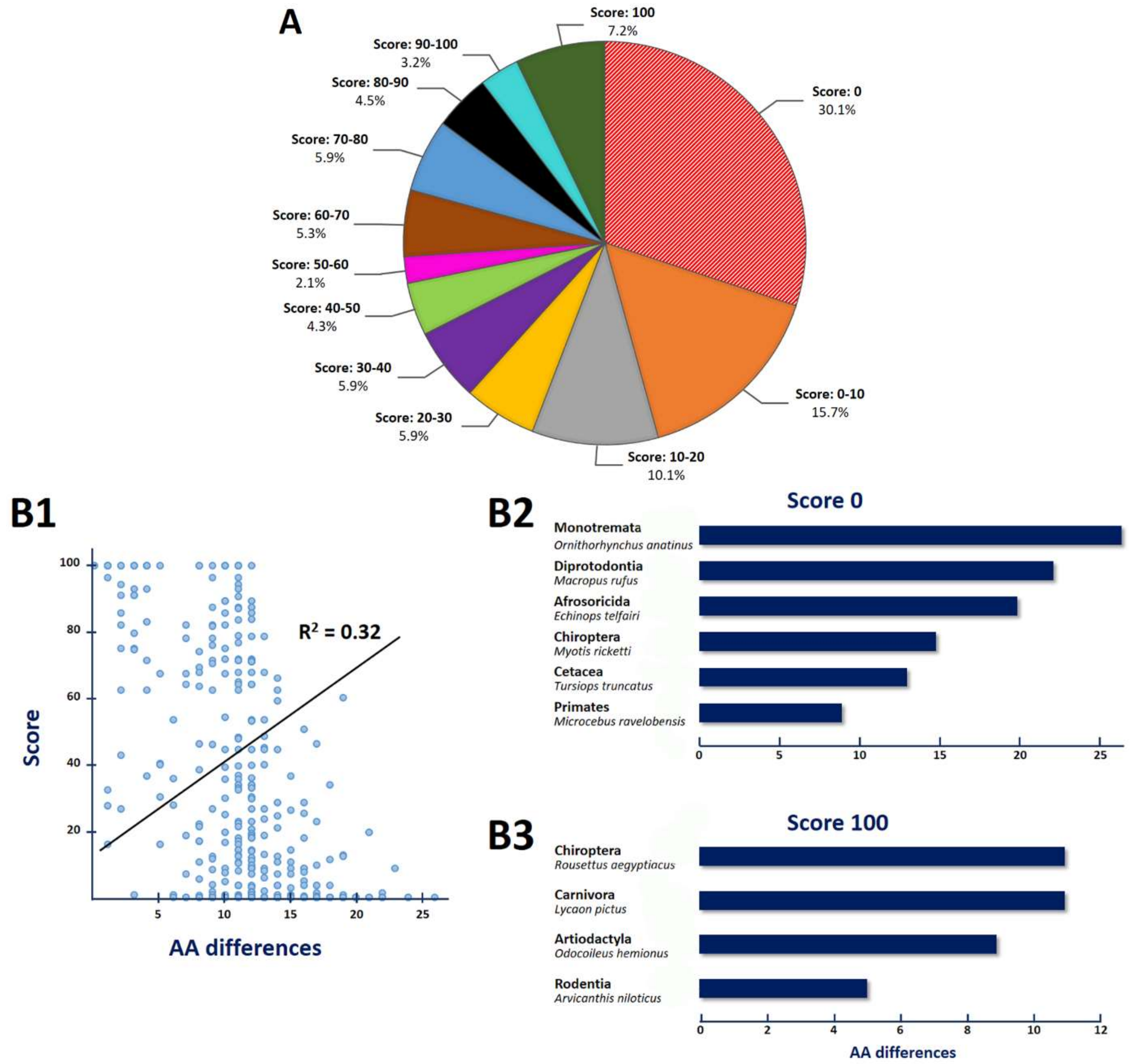

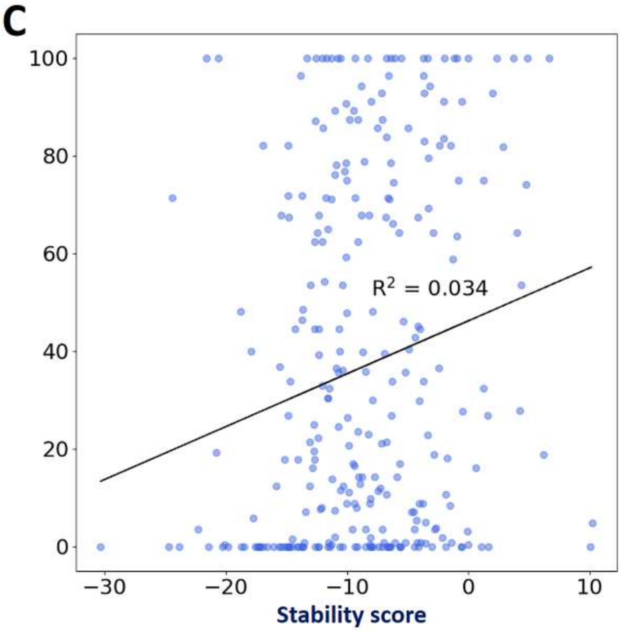

Supplementary figure 3. General analysis of PMSA results including distribution of rec-PrP variants with respect to misfolding score percentile and examination of potential correlations between misfolding score, amino acid differences and predicted stability. A) Pie chart depicting the distribution of rec-PrP variants grouped in deciles according to their misfolding proneness score. To get an overview of the overall misfolding capacity of the 382 distinct rec-PrP variants, the percentage of species with misfolding scores in the same decile was plotted. This figure illustrates the intriguing number of rec-PrP variants encountered with very low misfolding scores, representing almost half of the variants analyzed, being 30.1 % unable to misfold in our system. From the roughly 70 % that spontaneously misfolded, there is a wide distribution of species, with lowest and highest misfolding score deciles being more populated. Approximately 16 % of the species displayed misfolding scores below 20, while roughly 15 % of the species had scores higher

than 80, highlighting the resolution of our methodology in assessing the diverse misfolding capacities of different rec-PrP variants. **B) Analysis of potential correlation between misfolding score and number of amino acids differing from bank vole rec-PrP.** Given that our methodology was originally developed using bank vole rec-PrP, a species known for its high susceptibility to a wide range of prion diseases and thus, our gold standard in terms of misfolding proneness, we performed a correlative analysis, plotting misfolding score of each rec-PrP variant against the number of amino acids differing from bank vole rec-PrP. As shown in panel B1, there is no significant correlation (R^2=0.32) between these two parameters. This suggests that it is not a set of specific residues but rather their differential effect in the context of each rec-PrP variant what determines the spontaneous misfolding proneness. To better illustrate this, six species with misfolding scores of 0 (panel B2) and four species with misfolding scores of 100 (as bank vole rec-PrP, panel B3), and their number of differing residues with respect to bank vole rec-PrP are shown, indicating that high or low misfolding scores are not associated only with sequence similarity to bank vole. **C) Analysis of potential correlation between misfolding score and predicted stability of the natively folded rec-PrP.** To check if the stability of the natively folded isoform of each PrP variant could be somehow related to its spontaneous misfolding capacity, first the native structures of virtually all rec-PrP variants submitted to PMSA were predicted *in silico* using AlphaFold, as well as their relative thermodynamic stability, when possible, based on the similarity to bank vole PrP in terms of total amino acid number. The plot represents calculated thermodynamic stability ($\Delta\Delta G_f$, x-axis) vs. misfolding proneness score (y-axis) for 284 unique, equally long prion sequences. The low coefficient of determination (R^2=0.034) indicates that there is no significant correlation between the two variables, indicating that misfolding proneness is independent of the thermodynamic stability of the native isoform. All the Source Data used for these figures is publicly available in Zenodo repository (10.5281/zenodo.10579518) [1] as detailed in the Data Availability section.

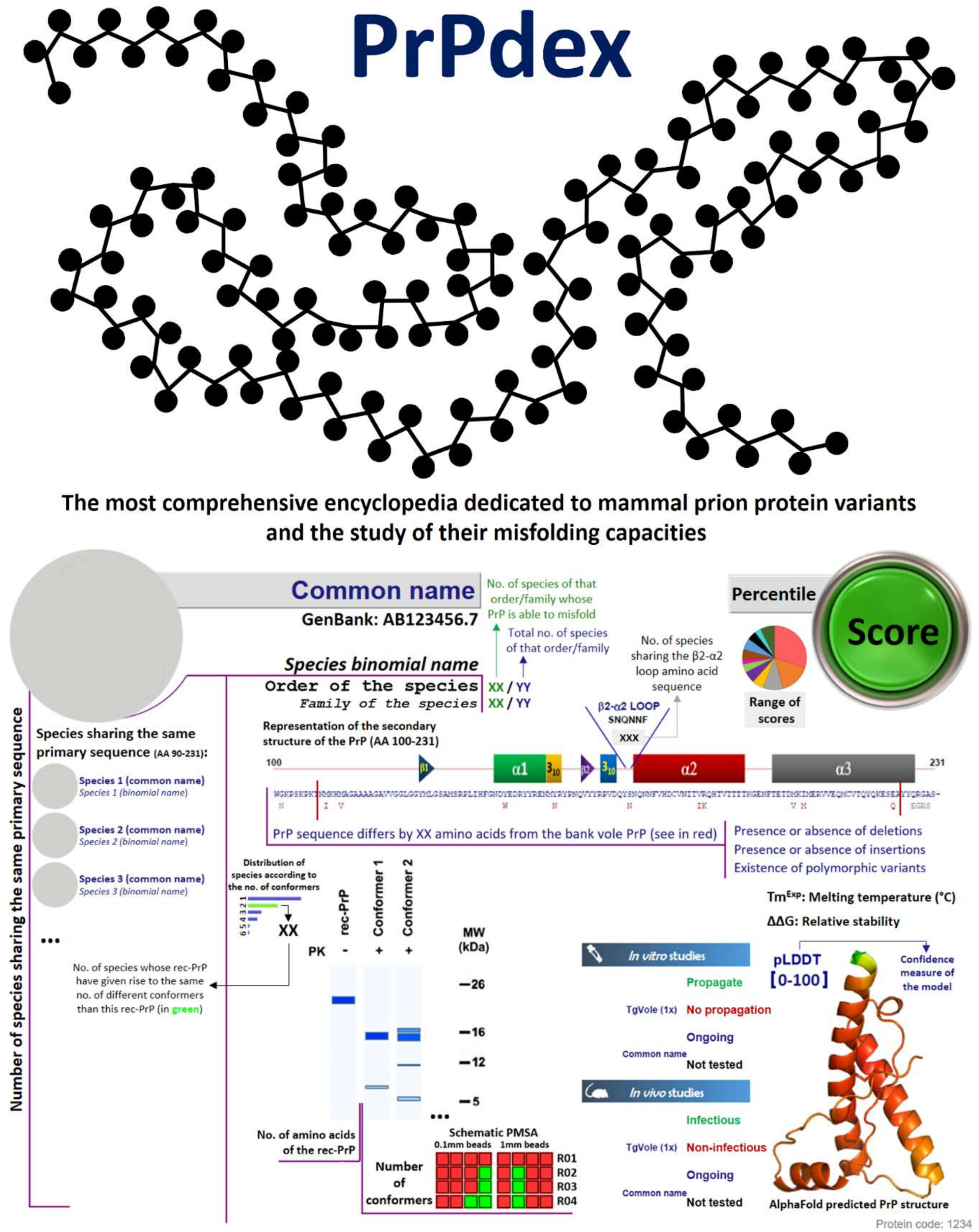

A pdf file with all 382 files available in https://prpdex.com/prpdex.pdf is also provided as Supplementary Data 1 in this manuscript.

Supplementary figure 4. Main page and example file of the PrPdex, the most comprehensive mammal PrP variant database. The database compiling all the results derived from this study, was named PrPdex, as it intends to be a dynamic and continuously growing resource with information on the PrP sequences and misfolding properties of all mammal species. Hosted in the https://prpdex.com webpage, this database contains 382 files corresponding to the PrP variants analyzed so far. Behind the front page, where a hypothetic three-dimensional structure of a PrP^{Sc} molecule (based on [2]) is depicted creatively substituting amino acids for images of some of the misfolding prone species studied, individual files for each of them can be found. Each file includes a

photograph of the mammal species, both their common and binomial names and the GenBank accession number of the PrP sequence in the upper left corner. On the left of each file, a list of species sharing the same PrP sequence from amino acid 90 onwards (the region thought to be most influential for misfolding) is provided. In cases where there are no shared sequences, the message "Distinct primary sequence across species" is displayed. In the upper right part of the file, the misfolding score is shown within a green circle if greater than 0, or within a red circle if the PrP was unable to misfold spontaneously in our system. The percentile rank in which the PrP is placed based on its misfolding proneness with regards to the whole sample is indicated, ranging from 29 % to 100 % (given the roughly 30 % of non misfoldeable PrP variants found). In addition, the pie chart classifies all analyzed PrP variants into deciles, highlighting the specific decile to which each PrP belongs in terms of misfolding capacity. Related to the spontaneous misfolding propensity, below the binomial name of the species, the file includes the number of species from the same order and family analyzed (blue) and the number or species in each case that was positive for spontaneous misfolding (green). The central part of the file illustrates the main secondary structure elements of the PrP, the two short β-sheets (β1 and β2), the three α-helixes (α1, α2 and α3) and the two 3_{10} helixes, emphasizing other potentially relevant sequences such as the β2-α2 loop. The corresponding amino acid sequence from residues 100 to the C-terminus is highlighted in red, indicating amino acids that differ from bank vole PrP. Any indels or polymorphic variants specific to the species are also disclosed. Finally, the results obtained throughout this study are shown summarized, including the electrophoretic mobility pattern of the different conformers detected as observed after PK treatment electrophoresis, and total protein staining (including a bar plot of the proportion of variants with 1 to 6 conformers), the rec-PrP substrate and its length in amino acids, a schematic representation of rec-PrP^{res} detection through the serial PMSA rounds (green squares representing rec-PrP^{res} positive samples in each replicate and round and red squares rec-PrP^{res} negative ones), and the infectivity studies performed *in vitro* and *in vivo*, with pictures of the models in which each of them was tested and a brief comment on the result. The AlphaFold predicted structure of each PrP is also provided in the file in an interactive format when accessing through the https://prpdex.com webpage, with the quality of the prediction indicated (pLDDT score), as well as the relative thermodynamic stability calculated from the structure (expressed as ΔΔG), together with the experimentally determined melting temperature (Tm) when available. The Source Data for Tm calculation is publicly available in Zenodo repository (10.5281/zenodo.10579518) as detailed in the Data Availability section.

SUPPLEMENTARY METHODS

Recombinant prion protein expression and purification

This protocol provides a comprehensive guide to obtain recombinant prion protein, in our case from pOPIN E plasmids, but also from any other vector for recombinant protein expression in *Escherichia coli*. Taking advantage of the histidine rich Octapeptide Repeats (OR) of all rec-PrP variants, that allow their purification by immobilized metal affinity chromatography, we strongly recommend avoiding rec-PrP expression fused to any affinity tag, since they could interfere with the downstream misfolding proneness assessment. In our case, despite pOPIN E plasmids originally contained a N-ter 6x histidine tag to be expressed fused to the recombinant protein, a stop codon was introduced between the PrP and histidine tag sequences. With the PRNP sequence of each variant cloned under the control of the *operon lac* promoter region, recombinant protein expression is inducible through addition of isopropyl β-D-1-thiogalactopyranoside (IPTG). Our vectors also contain an ampicillin resistance cassette, although any other antibiotic resistance cassette can be used. All PrP sequences subjected to this study and cloned into the bacterial expression vectors were obtained either from genomic DNA through PCR or synthesized (by Nzytech and GeneScript). In all cases, they express the PrP from residues 23-231 (in bank vole PrP numbering) or equivalent positions for species with longer or shorter PrP sequences.

Tip 1: For a standard study on spontaneous misfolding, it is essential to work with complete proteins that lack N-terminal or C-terminal deletions and are free from any type of tag associated with either end of the protein. While proteins lacking a significant portion of the flexible N-ter region can also be used, their production and purification without any tags is more complex, and the yield is significantly lower.

Materials

Chemicals

- S.O.C culture media (Invitrogen)
- Ampicillin sodium salt (Sigma-Aldrich)
- Luria Bertoni (LB) agar (Becton Dickinson)
- Luria Bertoni (LB) broth (Condalab)
- Glycerol (Acros)
- Isopropyl β-D-1-thiogalactopyranoside (IPTG) (GoldBio)
- $MgCl_2$ (Sigma-Aldrich)
- NaCl (Fisher Chemicals)
- Tris(hydroxymethyl)aminomethane hydrochloride Sodium phosphate monobasic (Tris-HCl) (Fisher Bioreagents)
- Triton X-100 (Sigma-Aldrich)
- Phenylmethylsulfonyl fluoride (PMSF) (Sigma-Aldrich)
- Ethylenediaminetetraacetic acid (EDTA) (Sigma-Aldrich)
- Sodium lauroyl sarcosinate (Sarkosyl) (Sigma-Aldrich)
- Guanidine hydrochloride (Sigma-Aldrich)
- Imidazole (Sigma-Aldrich)

Biologicals

- pOPIN E vectors containing the rec-PrP variant sequences (OPPF, gene synthesis and subcloning by Nzytech or GenScript)
- *Escherichia coli* Rossetta DE3 competent cells (Thermo Scientific)
- *DNase* (Sigma-Aldrich)
- Lysozyme (Sigma-Aldrich)

Solutions and other preparations

- LB agar plates with 50 µg/ml of ampicillin sodium salt
- LB broth with 50 µg/ml of ampicillin sodium salt
- GeneJET Plasmid Miniprep Kit (ThermoFisher Scientific)
- IPTG stock solution (100 mM) in distilled water
- $MgCl_2$ stock solution (200 mM) in distilled water
- *DNase* stock solution (2 mg/ml) in distilled water
- Lysis buffer [Tris-HCl 50 mM, EDTA 5 mM, Triton X-100 1% (v/v), PMSF 1 mM, Lysozyme 2 mg/ml, pH 8]
- Washing buffer [Tris-HCl 20 mM, EDTA 1 mM, Sarkosyl 1 % (w/v), pH 8]
- Inclusion buffer [Tris-HCl 20 mM, NaCl 500 mM, Guanidinium-HCl 6 M, pH 8]
- Binding buffer [Tris-HCl 20 mM, NaCl 500 mM, Guanidinium-HCl 2 M, Imidazole 5 mM, pH 8]
- Elution buffer [Tris-HCl 20 mM, NaCl 500 mM, Guanidinium-HCl 2 M, Imidazole 500 mM, pH 8]
- Stripping buffer [Sodium phosphate 20 mM, NaCl 500 mM, EDTA 500 mM, pH 7.4]
- BlueSafe (Nzytech)
- NuPage 4x Loading Buffer (Invitrogen)

Equipment

- Ice bucket
- Water bath set at 42 °C (JP Selecta)
- Laboratory oven set at 37 °C (Nahita)
- Petri dishes (Fisher Scientific)
- L-shaped spreader or autoclaved 5 mm diameter glass beads (Sigma-Aldrich)
- Bunsen burner
- 14 ml bacterial culture tubes (Simport)
- Shaking incubator for bacterial growth (Jeio Tech)
- 250 ml Erlenmeyer flasks
- 1 L Erlenmeyer flask
- Biowave Cell density meter CO8000 (WPA)
- 2.5 ml polystyrene disposable cuvettes (Labbox Labware)
- Sorvall Legend XTR centrifuge (Thermo Scientific)
- Swinging bucket rotor TX-750 (Thermo Scientific)
- FiberLite F15-6x100 fixed angle rotor (Thermo Scientific)
- 500 ml centrifugation bottles (Thermo Scientific)
- 50 ml centrifugation tubes (Thermo Scientific)
- HisTrap FF Crude 5 ml chromatographic columns (GE Healthcare)
- Nanodrop (ThermoFisher Scientific)
- 0.22 µm pore size cellulose acetate syringe filters (Corning)
- FPLC equipment, AKTA Start (GE Healthcare)
- Filter/Storage Bottle System, 0.22 µm. Cellulose Nitrate. 500ml (Corning) and vacuum pump

Methods

Bacterial transformation by heat shock

1. Thaw the competent *E. coli* Rossetta DE3 cells carefully on ice. Keep them at 4 °C throughout the entire transformation process.
2. Mix 1 µl of the bacterial expression plasmid solution (100 ng/µl) with 9 µl of competent E. coli Rossetta DE3 cells.
3. Incubate the mixture on ice for 20 minutes.
4. Heat-shock the mixture by placing it in a water bath at 42 °C for 30 seconds.
5. Immediately return the mixture to ice and let it incubate for 5 minutes.
6. Add 90 µl of prewarmed S.O.C. culture media to the mixture.

7. Incubate the tube at 37 °C for 1 hour.
8. Take an LB agar plate with ampicillin and carefully pour 50 µl of the transformed bacteria solution into the center of the plate.
9. Using a sterile spreader or 3 mm sterile glass beads within the sterile area provided by a Bunsen burner, spread the solution evenly over the surface of the agar plate.
10. Incubate the agar plate overnight at 37 °C in a laboratory oven.
11. From the grown bacterial colonies on the agar plate, select at least one colony for inoculation.
12. Inoculate the selected colony into 2.5 ml of LB broth with ampicillin. This step is to check for the presence of the desired plasmid.
13. After incubating the bacterial culture overnight at 37 °C with vigorous shaking, perform a miniprep for plasmid extraction following the manufacturer's instructions.
14. Analyze the extracted plasmid using your preferred method. In our case, we use sequencing of the ORF of the PRNP cloned into the vector.
15. Once the correctness of the plasmid is confirmed, use the exact same colony to inoculate a new LB agar plate for immediate use.
16. Additionally, grow the colony again in 2.5 ml of LB broth with ampicillin for long-term storage at -80 °C. Mix 800 µl of the grown culture with 200 µl of glycerol to prepare glycerol stocks.

Tip 2: It is important to mix thoroughly using vortex, if necessary, the bacterial culture and glycerol mix and to freeze it immediately at -80 °C, since the glycerol induced toxicity can greatly reduce the living bacteria in the glycerol stock for subsequent purification.

Recombinant prion protein expression

17. Inoculate 50 ml of LB broth with 50 µg/ml of ampicillin placed in a 250 ml Erlenmeyer flask either from the agar plate or from the glycerol stock, under the Bunsen burner, and let this pre-culture grow at 37 °C with vigorous shaking overnight.
18. Once grown, add the pre-culture to 500 ml of LB broth with ampicillin and placed in a 1 L Erlenmeyer flask, and let the bacteria grow until the culture reaches an optical density (OD) between 0.8 and 1, measuring absorbance at 600 nm, which in our case takes normally about 2-3 h.
19. Once the desired OD is reached, recombinant protein expression is induced through addition of IPTG at a final concentration of 1 mM from the stock solution.
20. After induction, keep incubating the bacterial culture with vigorous shaking for 3 h at 37 °C for maximum recombinant prion protein expression yield. Alternatively, the culture can be kept overnight at 30 °C, also with vigorous shaking.
21. After expression, place the Erlenmeyer flasks in ice for 15 min and then transfer the culture to appropriate centrifuge tubes, in our case 500 ml centrifugation bottles, for centrifugation at 4,500 *g* for 15 min at 4 °C, to pellet the bacteria and separate them from the culture media.
22. Discard the supernatant and keep the bacterial pellet for purification. These bacterial pellets can be used immediately or stored at -20 °C for posterior purification.

Lysis of the bacterial pellets and solubilization of inclusion bodies

23. Suspend thoroughly the bacterial pellets using 50 ml of Lysis buffer and adding afterwards 37.5 µl of DNase from the stock solution for a final concentration of 5 µg/ml and 5.5 ml of the $MgCl_2$ stock solution for a final concentration of 20 mM.
24. Transfer the suspension to appropriate centrifugal tubes and incubate the suspension with gentle shaking for 30 min at room temperature.
25. After incubation, centrifuge the lysate at 8,500 *g* for 1 h at 4 °C.

26. Discard the supernatant and suspend the pellet in 50 ml of washing buffer until getting a homogeneous suspension, what we usually do in two parts, suspending first the pellet in 30 ml and adding the other 20 ml at the end.
27. Centrifuge the suspension in the same centrifugal tubes at 8,500 *g* for 1 h and 4 °C and discard the supernatant.
28. Carefully wash the pellet with distilled water to remove any residual detergents and debris adhering to the pellet and tube walls. Ensure thorough washing while avoiding suspension of the pellet. Discard the water, and then suspend the washed pellet in 12 ml of inclusion buffer.
29. Incubate the suspension at 37 °C with gentle shaking overnight. Consider covering the tubes with aluminum foil to protect the resuspended protein from excessive light exposure.

Tip 3: Throughout the protein preparation procedure, it is strongly advised to prevent protein oxidation. Therefore, exposure to light, especially during overnight processes, should be minimized. While the addition of DTT or other reducing agents is permissible, the potential impact of these agents on the final outcome remains unknown. Regardless, it is not recommended to use more than 1 mM of DTT during the purification process.

30. Centrifuge the suspension in the same centrifugal tubes at 8,500 *g* for 1 h and 4 °C and keep the supernatant in this case, discarding the pellet.
31. At this point the solubilized protein can be stored at -20 ºC. Due to the inclusion buffer's resistance to freezing at this temperature, ensure the tube is covered with aluminum foil to protect from light exposure. If turbidity is observed, centrifuge the sample at 4,500 *g* for 15 min at 4 ºC and keep the supernatant.
32. Filter the supernatant through a 0.22 µm pore-size syringe filter. If the filter clogs after 3-4 ml of solubilized protein, prefilter the protein with 5 µm pore-size syringe filter before using the 0.22 µm pore-size filter.

Tip 4: It is important to emphasize that protein filtration should always be performed before loading onto the AKTA system. This step is crucial to prevent aggregates from entering the column. Never filter the protein and then freeze it since this can lead to aggregate formation. The ideal practice is to filter and use the protein on the same day.

33. Take an aliquot of the lysate of 10 µl to monitor the lysis and purification process at the end.

Purification by immobilized metal affinity chromatography using FPLC equipment

34. An FPLC equipment with at least two separate valves is necessary for this procedure. For this study, we have used an AKTA Start equipment. Before starting purification, make sure that all valves to be used, tubing and HisTrap column are thoroughly washed with distilled water. For instance, 500 ml of LB-cultured bacteria usually results in approximately 12 ml of solubilized protein, and a 1 x HisTrap FF Crude 5 ml column should be used. If a larger quantity of protein, such as 3 liters of LB-cultured bacteria, needs purification, we advise using three interconnected 3 x HisTrap FF Crude 5 ml columns to prevent protein loss, as the column's protein-binding capacity is limited.

Comment: While we don't have a definitive explanation, it's a known fact that not all plasmids yield the same amount of protein, despite their similarity, even when working with genes that have been previously optimized for specific expression in E. coli. For this reason, there have been instances in which obtaining the required amount of protein for a comprehensive misfolding study has required up to 3 L, instead of the usual 500 ml.

35. In the case of the valve used to load the sample in the column, perform an additional wash with inclusion buffer. Use 15 ml of water followed by 15 ml of inclusion buffer for this step.

36. Once the whole system, including the HisTrap column, is properly washed and equilibrated with water, place valve A in a bottle containing filtered binding buffer, and valve B in a bottle containing filtered elution buffer.
37. Equilibrate the HisTrap column with at least 5 column volumes (CV) of binding buffer.
38. Load the approximately 12 ml of the previously filtered supernatant derived from the bacterial lysate into the HisTrap column.
39. Collect the fraction(s) corresponding to the column loading to monitor the purification procedure, keeping at least 10 µl for this purpose.
40. After loading the lysate into the column and collecting the "Loading" fractions, proceed with washing and elution steps. Begin with 7 CV of binding buffer (valve A) first and conclude with 10 CV of elution buffer (valve B). Note that no gradient is performed by mixing both buffers.
41. During elution, a distinct peak should be visible in the chromatogram at 280 nm corresponding to the purified protein. Mix all the fractions containing detectable protein by absorbance at 280 nm and reserve 10 µl of the mixture to monitor the purification process.
42. Add guanidine-HCl to the elution fractions mixture to achieve a final concentration of 6 M (for instance, add 3.83 g of guanidine-HCl for every 10 ml of elution volume). Ensure the elution fraction mix is stored at 4 °C, protected from light.
43. Wash the valves, tubing, and HisTrap column with at least 10 CV of distilled water, and then switch to a 20 % ethanol solution if the equipment and HisTrap column are not going to be used immediately.
44. To reuse the chromatographic column with different prion protein variants, it is necessary to strip them and reload them with nickel. Follow these steps:

Tip 5: Reusing nickel columns is a common practice, especially when dealing with numerous proteins. However, it's important to note that despite their longevity, columns may eventually compromise the protein's ability to misfold consistently. This phenomenon is attributed to the gradual buildup of substances that resist complete removal during rigorous cleaning processes. Such accumulation may elevate the risk of protein oxidation or damage. To maintain optimal protein functionality, it is advisable not to exceed 10 reuse cycles for these columns, even if the purified protein appears indistinguishable from those obtained using less-utilized columns.

    a. Using the same FPLC equipment, introduce valve A in a bottle with distilled water, and valve B in a bottle containing stripping buffer.
    b. Wash the tubing system and the column with 7 CV of distilled water (valve A), and then with 7 CV of stripping buffer (valve B).
    c. Change valve B to a bottle containing binding buffer and wash the column with 7 CV of binding buffer (valve B), and then with 7 CV of distilled water (valve A).
    d. Inject 5 ml of $NiSO_4$ solution to the column.
    e. Wash the column again with 7 CV of distilled water (valve A), and then with 7 CV of binding buffer (valve B).
    f. Transfer valves A and B to a bottle with 20 % ethanol solution. Wash valves, tubing, and column with 7 CV of 20 % ethanol. Disconnect the column from the FPLC equipment and seal the ends for long-term storage at 4 °C. Also, keep the valves in ethanol solution until the FPLC equipment is used again.

45. Precipitate the 10 µl from the lysis, loading, and elution fractions collected by adding at least 60 µl of ice-cold methanol to each sample in separate 1.5 ml Eppendorf tubes. Incubate the mixes for a minimum of 30 min at -20 °C.
46. After incubation, centrifuge the samples at 20,000 *g* for 30 min to pellet the protein. Carefully discard the supernatant without disturbing the pellet. Allow the protein pellets to dry completely, and then resuspend them in 10 to 15 µl of loading buffer to visualize them through electrophoresis and total protein staining.

47. Measure the concentration of the elution fraction mix by assessing absorbance at 280 nm. It is recommended using equipment like Nanodrop for this purpose.
48. If necessary, concentrate the eluted protein using centrifugal filter units with a cut-off of 10 kDa. Centrifuge at 4,500 *g* and 4 °C until reaching a concentration of 25 mg/ml.

Comment: The recommended storage concentration is 25 mg/ml. This selection is well-suited for subsequent substrate preparation. It's worth highlighting that protein concentration is a pivotal variable in achieving reliable results in spontaneous misfolding studies. As the rec-PrP misfolding *in vitro* is influenced by the concentration within the substrate, it is important to keep similar concentrations among the different substrates, always within a certain range as illustrated in Supplementary figure 2. While the impact of the final working concentration may vary among proteins, it exerts a more pronounced effect on those with limited misfolding capacity. Regardless, maintaining a precise record of the protein concentration at every stage of the procedure is of paramount importance.

49. Store the aliquots at -80°C until they are ready for use.

Tip 6: Although prion proteins are inherently stable, it is crucial to store them properly to maintain their integrity for spontaneous misfolding studies. Incorrect storage at -20°C, instead of -80°C, can lead to significant protein damage.

Substrate preparation for Protein Misfolding Shaking Amplification

Once recombinant prion protein variants have been produced and purified, the critical next step in successfully implementing this technique in any laboratory is substrate preparation for PMSA. This involves dialyzing the protein, which is stored at -80 °C and with a 6 M of Gdn-HCl solution, to facilitate its re-folding into its native globular conformation. Subsequently, it is combined with other essential components of the substrate.

Tip 7: As mentioned previously, laboratories engaged in the production of prion proteins or similar proteins for various techniques, including amplification methods like RT-QuIC, or for their use in other biophysical techniques, are experienced in producing high-quality recombinant proteins through more sophisticated purification procedures. We firmly believe that to attain the reliable spontaneous misfolding results described in this study, it is of paramount importance for the protein to undergo refolding via dialysis, as detailed below. This approach ensures the production of high-quality protein, as demonstrated by the presence of a single, distinct band in the hundreds of purified and refolded proteins.

Materials

Chemicals

- Triton-X-100 (Sigma-Aldrich)
- NaCl (Fisher Cehmicals)
- PBS (HyClone)
- Guanidine hydrochloride (Gdn-HCl) (Sigma-Aldrich)
- BlueSafe (Nzytech)

Solutions

- Conversion Buffer (CB) [NaCl 0.15M, Triton-X-100 1% (v/v) in PBS]
- Dextran sulfate stock solution [Dextran sulfate 5% (w/v) in PBS]

Biologicals

- Dextran sulfate from *Leuconostoc spp.* 6,500-10,000 MW (Sigma-Aldrich)
- Purified rec-PrP

Equipment

- Dialysis cassettes (Thermo Scientific)
- Ice bucket
- 5 L beakers
- Magnetic stirrer and magnet
- 2 ml tubes with conical bottom and screw cap (Fisherbrand)
- Disposable pipettes and aspirator
- 1 mm diameter glass beads (BioSpec Products, Inc.)
- 0.1 mm diameter glass beads (BioSpec Products, Inc.)
- Centrifuge ST 16 R (Thermo Sorvall)

Methods

Dialysis of the rec-PrP for re-folding

1. Thaw 1 aliquot (100 µl) of the rec-PrP on ice, transfer the completely thawed rec-PrP to a 5 ml Eppendorf tube and add 400 µl of PBS to achieve an initial 1:5 dilution.

Tip 8: We recommend preparing this mixture by directly adding the denatured protein, which is in a 6 M Gdn HCl solution, to the PBS. The opposite approach is not recommended. This is because it has been observed that less protein precipitates, leading to a higher final yield.

2. Incubate the diluted protein on ice for 5-10 min at room temperature while preparing the rest of the materials for dialysis.
3. Prepare 5 L of PBS (for a 1:1,000 dilution) in a 5 L plastic beaker and wet the dialysis cassette membranes by immersing them in the PBS solution for 1 min.

TIP 9: While one might intuitively expect to work at ice-cold temperatures (4 °C), our experimental data suggest that performing dialysis at room temperature yields better results.

4. Introduce the diluted recombinant protein in the dialysis cassette using a 1 ml disposable pipette and submerge the cassette in the PBS solution with a magnetic stirrer for gentle agitation during dialysis.
5. Dialyze for 1 h at room temperature. Meanwhile, prepare 5 L of fresh PBS in another 5 L beaker.
6. After 1 h, transfer the cassette to the new beaker and continue dialysis for an additional 1 h (final dilution 1:1,000,000) with mild agitation at room temperature.
7. Extract the dialyzed rec-PrP from the cassette and transfer the dialyzed solution to 2 ml Eppendorf tubes for centrifugation at 19,000 *g* for 15 min at 4 °C, to remove insoluble proteins.
8. Carefully collect the supernatant without disturbing the pellet and transfer them to a clean 2 ml Eppendorf tube.

Comment: This procedure is pivotal for the preparation of a suitable substrate, as the presence of potentially amorphous aggregates of proteins, or other insoluble impurities can profoundly influence the subsequent outcome of spontaneous misfolding. Performing it at 4 °C is recommended since it enhances the precipitation of aggregates overall.

Preparation of the PMSA substrate

9. Mix the dialyzed and centrifuged recombinant protein with 4.5 ml of conversion buffer (CB) at a 1:9 dilution, maintaining the mixture on ice as long as possible.
10. After thoroughly mixing the protein and the CB, add 0.5 ml of the 5 % sulfated dextran solution to achieve a final concentration in the substrate of 0.5 % (w/v).

Comment: Throughout the study, we exclusively utilized a single cofactor, sulfated dextran. This choice stemmed from the fact that, despite initial testing of other similar cofactors with a limited number of different proteins, only sulfated dextran consistently and robustly facilitated spontaneous misfolding in conjunction with the other components.

11. Prepare a set of 2 ml tubes with conical bottoms and screw cap. Add 100 mg of 1 mm diameter glass beads to at least 16 tubes and 100 mg of 0.1 mm diameter glass beads to another set of at least 16 tubes.

Comment: One of the critical components that facilitate spontaneous misfolding is the inclusion of glass beads to the system. Our previous studies, which delved into understanding the mechanism through which the glass surfaces induce conformational changes, have made it very clear that careful attention must be paid not only to the size of the beads but also to their prior processing, along with other variables as described in the reference [2]. Therefore, we strongly recommend utilizing the same glass beads and adhering to the same conditions as outlined in this study.

12. Reserve 60 µl of the substrate for quality control in a separate Eppendorf tube. Aliquot the remaining substrate into the previously prepared 2 ml tubes with conical bottoms and screw caps placing 600 µl of substrate in each tube. Store the aliquoted substrate at -80 °C until needed for PMSA.
13. For the quality control, mix the reserved 60 µl of the substrate with 20 µl of electrophoresis loading buffer (4x) to reach a 1x concentration. Boil the mixture at 100 °C for 10 min and load it onto an electrophoresis gel along with the appropriate molecular weight marker.
14. Run the gel for 10 min at 70 V, 10 min at 110 V and 60 min at 150 V. Stain the gel with a total protein staining solution (e. g. BlueSafe) by placing the gel in a cuvette containing 50 ml of the staining solution. Incubate with gentle shaking at room temperature for 1 h.
15. Assess the protein amount (signal intensity) and quality (presence of a single band with expected size, absence of multimers or proteolytic fragments) based on the previous gel. Compare with other prepared substrates and, if the substrate meets the criteria for adequacy, it is considered suitable for PMSA.

Comment: The presence of a clearly discernible single band, approximately 20-23 kDa in size, after total protein staining is a reliable indicator of a high-quality protein with correct folding, as confirmed by circular dichroism studies. This condition is an indispensable requirement for initiate the studies on spontaneous misfolding.

Tip 10: It is advisable to carefully consider the purification method for the recombinant protein as it significantly influences the desired outcomes of protein misfolding. Purification via alternative methods, which involve protein folding within affinity columns with or without additional purification using HPLC, commonly employed in many laboratories for this protein, may not yield the same results for spontaneous protein misfolding studies.

Tip 11: We strongly discourage the process of lyophilization or any other procedures that could potentially facilitate the oxidation of methionine residues in the purified prion protein.

Protein Misfolding Shaking Amplification

Once the substrate is prepared and protein concentration and quality have been evaluated through electrophoresis and total protein staining, unseeded PMSA reactions can be set to assess the misfolding proneness of each rec-PrP variant. These reactions are carried out in the presence of glass beads, as they play a crucial role in promoting spontaneous misfolding. Although other experimental designs could allow ranking rec-PrP variants in terms of misfolding proneness, we believe this approach

is the most cost-effective and provides enough resolution to distinguish among variants with differential misfolding capabilities. However, we acknowledge that increasing the number of replicates or conducting serial PMSA rounds, among other potential adjustments to the experimental setting, could lead to further discrimination between those variants with identical or similar scores according to our methodology. Additionally, as done in this work, those rec-PrP variants with all replicates negative for rec-PrP^{res} and thus, apparently unable to misfold, should be repeated twice to confirm the negative results.

Materials

Chemicals

- BlueSafe (Nzytech)

Biologicals

- PMSA substrates complemented with 1- and 0.1-mm diameter glass beads
- Proteinase K (PK) (Roche)

Solutions

- Proteinase K stock solution at 10 µg/µl in MilliQ grade water

Equipment

- Ice bucket
- Thermomixer (Eppendorf) or Digital shaking Drybath (Thermofisher Scientific)
- Centrifuge ST 16 R (Thermo Sorvall)

Methods

Unseeded serial PMSA rounds

1. Thaw four tubes of the substrate, each aliquoted with 1 mm diameter glass beads, and four tubes containing 0.1 mm glass beads, all kept in ice.
2. Label these tubes, indicating the rec-PrP variant, bead type, replicate tube (a to d), and the PMSA round (R01).
3. Place the labeled tubes in a shaker with temperature and shaking speed control, set at 39 °C and continuous shaking at 700 rpm for 24 h. We have used either a Thermomixer from Eppendorf or a Digital shaking Drybath from Thermofisher Scientific.

> **Comment:** Although the study presented in this manuscript has been performed at 39 °C, previous data from our laboratory indicates that PMSA temperature is not pivotal and that similar results could be obtained working within a quite wide temperature range, from 35 to 40 °C.

4. After the first 24 h PMSA round, thaw another set of eight tubes of the aliquoted substrate in ice, again four with 1 mm diameter glass beads and four with 0.1 mm diameter glass beads. Properly label the tubes, indicating the rec-PrP variant, bead type, replicate (a to d), and this will be the second PMSA round (R02).
5. Inoculate this second set of tubes for the second round with 66 µl of the products from the first round. Perform a 1:10 dilution of the products from the first round in the new substrate, ensuring that each replicate is inoculated with the equivalent replicate from the previous round.

6. Place the newly inoculated tubes for the second round in the shaker at 39 °C and 700 rpm for 24 h.
7. Repeat the same procedure for the third and fourth PMSA rounds. Inoculate a new set of tubes with fresh substrate from the replicates of the previous round at 1:10 dilution and subject them to a 24 h PMSA round at 39 °C and continuous shaking at 700 rpm.

Assessment of rec-PrP^{res} formation

8. Transfer 450 µl of each PMSA product to clean 1.5 ml eppendorf tubes for proteinase K digestion.
9. Add 11 µl of the 1 µg/µl PK solution to each sample, mixing gently to achieve a final concentration of 25 µg/ml.
10. Incubate the tubes for 1 h at 42 °C in a laboratory oven without shaking.
11. After the digestion, immediately centrifuge the samples at 19,000 *g* for 15 min and 4 °C and carefully discard the supernatant without disturbing the pellet.
12. Wash the pellets without resuspension by adding 500 µl of PBS and then centrifuge the samples at 19,000 g for 15 minutes at 4 °C. Resuspend the pellets in 16 µl of electrophoresis loading buffer previously diluted to 1x in PBS and boil them for 10 min at 100 °C to completely halt PK digestion.
13. Load 15 µl of the boiled samples onto an acrylamide gel and run the electrophoresis. In our case, use the following settings: at 70 V 10 min, at 110 V for another 10 min and at 150 V for 60 min.
14. Once the gel run is complete, stain the gel submerging it in 50 ml of BlueSafe for 1 h with mild agitation at room temperature for total protein staining.
15. For the interpretation of the results, based on our experience with *in vitro* misfolding of rec-PrP, only those samples displaying a 16 kDa band after PK digestion, electrophoresis, and staining are considered as rec-PrP^{res} positive, regardless of smaller molecular weight fragments.

Comment: Despite our incomplete understanding of the spontaneous misfolding process and the resulting fragments observed during total protein staining, we consider the presence of a band spanning approximately from residues 90 to 230 to be a positive indicator of infectivity. However, it is worth noting that some conformers may produce only fragments, including those suggesting greater susceptibility to PK digestion in certain regions of the protein. While we cannot definitively establish a direct correlation between fragment types and infectivity, for the purposes of our study, we consider *bona fide* misfolding when the 16 kDa band is present, whether or not it is accompanied by smaller fragments.

Tip 12: Some of the critical parameters for replicating the results are both the speed and type of shaking. We have observed that shaking speeds below 500 rpm and above 1000 rpm have a negative impact on spontaneous misfolding. It cannot be ruled out that the optimal adjustment for consistent results may need to be made for each of the proteins used. Given the vast number of proteins tested and preliminary findings, it was agreed that a shaking speed of 700 rpm was suitable under these circumstances.

In addition to shaking speed, two other important factors are the type of shaking and the position of the tubes during shaking. Unfortunately, equipment allowing for variations in shaking type, some being broader and orbital than others, were not found. Since this can influence the results, it is recommended to work with the equipment described here or others with the same shaking specifications.

Furthermore, it has also been observed that positioning the tubes horizontally during shaking promotes spontaneous misfolding, likely due to providing a larger shaking surface area along with the beads. Regrettably, no commercial equipment options support this type of shaking with horizontally placed tubes. As a solution, we chose to create our own racks through 3D printing, which were used in simple shakers maintained at the appropriate temperature by placing them in an air oven.

Purification of rec-PrP^{res} to be used as seeds in PMCA

To assess if the rec-PrP^{res} generated in PMSA could potentially induce a transmissible spongiform encephalopathy *in vivo*, we first evaluated their capacity to misfold brain-derived PrP^{C} *in vitro*, a strong indicator of infectivity *in vivo*. Given the challenge of obtaining brain homogenates for the varied number of rec-PrP sequences to ascertain the capacity to misfold homologous PrP^{C}, and aware that interspecies transmission barriers could lead to underestimate the potential infectious capacity of the rec-PrP^{res}, we decided to employ TgVole brain homogenates as substrate. This transgenic mouse line, expressing the PrP^{C} from bank vole with I109 polymorphism approximately 1-fold, as the bank voles themselves behave as universal acceptor of prions, being susceptible to a wide range of prion strains from distinct species.

Comment: This study can be conducted using brains from various sources that match the seed being tested or even human brains to assess transmission barriers in a straightforward manner.

To enhance the efficiency of the PMCA reactions seeded with rec-PrP^{res}, we chose to concentrate the misfolded protein. However, to avoid problems due to excessive concentration of other components of the PMSA product, such as the cofactor or detergent, and considering that continuous shaking can lead to the release of bead fragments, we conducted a partial purification of the rec-PrP^{res} by ultracentrifugation through a density gradient.

Materials

Chemicals

- Cesium sulfate (Cs_2SO_4) (Sigma-Aldrich)
- PBS (HyClone)

Biologicals

- PMSA product containing rec-PrP^{res}
- Perfused TgVole 1x brains
- Sha31 monoclonal anti-PrP antibody (Bertin Bioreagents)

Solutions

- 1 M and 1.7 M Cs_2SO_4 solutions in distilled water
- Conversion buffer (CB) with Complete Protease inhibitor cocktail (Roche)

Equipment

- 15 ml gradient mixer (Sigma-Aldrich)
- Thinwall Ultra-Clear, 13.2 ml, centrifuge tubes (Beckman Coulter)
- SW41 Ti Swinging bucket rotor and corresponding buckets (Beckman Coulter)
- Optima L-90K ultracentrifuge (Beckman Coulter)

Methods

Concentration and purification of rec-PrP^{res} from PMSA products

1. Place 10 ml of a PMSA product resulting from a 24 h PMSA reaction to a 15 ml conical-bottom Falcon tube. Allow the protein aggregates sediment for at least 12 h at 4 °C. A white sediment should be observable at the bottom of the tube within a few hours.

2. Once sedimented, carefully discard the supernatant, reducing the volume of the suspension containing sedimented rec-PrP^{res} to a minimum. Then, add PBS solution to reach a final volume of 2 ml.

Comment: After conducting studies on numerous preparations post-PMSA, it has been observed that, following 12 h of sedimentation without centrifugation, the high density of rec-PrP^{res} causes it to precipitate completely. As a result, neither rec-PrP nor rec-PrP^{res} can be detected in the supernatant.

3. Prepare a continuous density gradient of Cs_2SO_4 using a gradient mixer with two chambers. Place at least 7 ml of a 1 M Cs_2SO_4 solution in one chamber and a 1.7 M solution in the other, creating a final gradient ranging from 1 M at the top to 1.7 M at the bottom. Dispense the gradient (approximately 11.2 ml in total) in a Thinwall Ultra-Clear, 13.2 ml centrifuge tube.
4. Place the ultracentrifuge tube in the corresponding swinging bucket, close the lid, and ultracentrifuge the samples at 210,000 *g* for 15 h at 20 °C. In our case was performed using a SW41 Ti Swinging bucket rotor and an Optima L-90K ultracentrifuge.
5. After centrifugation, you should observe at least one white-colored halo within the gradient, where according to their density, most of the rec-PrP^{res} is concentrated.
6. Carefully collect the fraction(s) containing visible precipitated protein halo with a pipette and transfer it to a new 5 ml Eppendorf tube for washing.

Comment: After conducting this purification process with more than 40 different conformers, we have observed significant variations in the appearance and position of the gradient halo. This behavior may be linked to the specific structural characteristics of each conformer. This information is crucial because, in some cases, it may be necessary to adjust the gradient type to prevent rec-PrP^{res} from precipitating, thus ensuring successful purification.

7. Add MilliQ-grade water to the partially purified rec-PrP^{res} until the 5 ml tube is filled to dilute the Cs_2SO_4. Centrifuge the sample at 4,000 g for 30 min at room temperature.
8. Discard almost all of the supernatant, leaving a small volume (approximately 50-100 µl) to resuspend the pellet. Transfer the suspended pellet to a new 1.5 ml Eppendorf tube for further washing.
9. Add 1 ml of PBS to the tube and thoroughly mix until complete resuspension is achieved. Centrifuge the tube for 15 min at 19,000 *g*. Repeat the PBS suspension and centrifugation step at least two additional times.
10. After the third wash, remove all of the supernatant and resuspend the pellet in 25-50 µl of PBS, adjusting the volume based on the final pellet size. This purified and concentrated rec-PrP^{res} will be used as seed for the following PMCA reactions.

PMCA to assess the capacity of the rec-PrP^{res} to misfold brain-derived PrP^C

11. The PMCA procedure closely follows previously published methods [3] with minimal variations, and therefore will be briefly explained.
12. Prepare substrates using Glass/Teflon Potter Elvehjem homogenizers using perfused TgVole 1x brain homogenate at a concentration of 10 % (w/v) in conversion buffer supplemented with protease inhibitor cocktail. Aliquot the substrates into PCR tubes, with 50 µl per tube, and add 2-5 1 mm diameter zirconium silicate beads to each tube. Properly aliquoted substrate can be stored at -80 °C until its use.
13. Seed two substrate tubes or replicates at a 1:100 dilution with the previously purified rec-PrP^{res} seed. Seal the tubes with parafilm to reduce cross-contamination and prevent accidental opening. Subject the tubes to a 24 h PMCA round with incubation cycles of 30 min, followed

by sonication pulses of 20 s at 80 % power at 38 °C regulated by a circulating water bath. Include at least two unseeded tubes as controls for cross-contamination or spontaneous misfolding.

14. After the first 24 h PMCA round, perform two additional serial PMCA rounds by seeding a set of freshly thawed substrate tubes at a 1:10 dilution with the PMCA product from the previous round, and again include unseeded controls. Conduct the second serial PMCA round under the same conditions as the first, also for 24 h.
15. Finally, carry out a third serial round using the same method, seeding a new set of substrate tubes at a 1:10 dilution with the product from the previous round.

Comment: The application of a highly reliable *in vitro* method such as PMCA proves to be of immense utility in evaluating the infectious potential of seeds purified using the proposed method. In this study, the decision was made to employ three rounds of PMCA for the scope of this investigation. Seeds that fail to propagate through brain derived PrP^{C} after the third round are categorized as negative. However, it is worth noting that this criterion could be adjusted to include additional rounds to detect seeds with very low infectivity.

16. Assess the presence of brain derived PrP^{Sc} in all replicates of each PMCA round by proteinase K digestion, electrophoresis, and Western blotting, as previously described [2], using Sha31 (1:4000) as primary antibody.

References

1. Eraña H*, et al.* Source Data Files from publication: A Protein Misfolding Shaking Amplification-based method for the spontaneous generation of hundreds of infectious prions, Nature Communications 2024. (2024).
2. Manka SW*, et al.* A structural basis for prion strain diversity. *Nat Chem Biol* **19**, 607-613 (2023).
3. Erana H*, et al.* Understanding the key features of the spontaneous formation of bona fide prions through a novel methodology that enables their swift and consistent generation. *Acta Neuropathol Commun* **11**, 145 (2023).

List of reagents

Reagent	Company	Catalog no.
E. coli Rosetta™ (DE3) competent cells	EMD Millipore	16440054
Isopropyl β-D-1-thiogalactopyranoside (IPTG)	Gold biotechnology	I2481C200
S.O.C culture media	Invitrogen	11528896
Ampicillin sodium salt	Sigma-Aldrich	A9518-25G
Luria Bertoni (LB) agar	Becton Dickinson	W5374D
Luria Bertoni (LB) broth	Condalab	1231
Glycerol	Acros	10296200
$MgCl_2$	Sigma-Aldrich	M8266-100G
NaCl	Fisher Chemicals	10356340
Tris(hydroxymethyl)aminomethane hydrochloride Sodium phosphate monobasic (Tris-HCl)	Fisher Bioreagents	10001223
Triton X-100	Sigma-Aldrich	T8787-250ML

Phenylmethylsulfonyl fluoride (PMSF)	Sigma-Aldrich	78830-5G
Ethylenediaminetetraacetic acid (EDTA)	Sigma-Aldrich	03690-100ML
Sodium lauroyl sarcosinate (Sarkosyl)	Sigma-Aldrich	L9150-250G
Imidazole	Sigma-Aldrich	I2399-500G
Guanidine hydrochloride	Sigma-Aldrich	G4505-2KG
DNAse	Sigma-Aldrich	DN25-100MG
Lysozyme	Sigma-Aldrich	62971-50G-F
BlueSafe	Nzytech	MB15201
NuPage 4x Loading Buffer	Invitrogen	11559166
HisTrap FF Crude 5 ml chromatographic columns	(GE Healthcare)	11723219
PBS	(HyClone)	1070-8334
Dextran sulfate from *Leuconostoc spp.* 6,500-10,000 MW	Sigma-Aldrich	D4911-100G
Proteinase K (PK)	Roche	3115879001
Cesium sulfate (Cs_2SO_4)	Sigma-Aldrich	C3136-250G
Sha31 monoclonal anti-PrP antibody	Bertin Bioreagents	A03213
Complete Protease inhibitor cocktail	Roche	11836145001
4-12 % acrylamide gels (NuPAGE Midi gel	Invitrogen Life Technologies	WG1403BOX
peroxidase-conjugated secondary anti-mouse antibody (m-IgGκ BP-HRP)	Santa Cruz Biotechnology	sc-516102
BCA assay	Thermo Scientific	10741395

Supplementary table 1. List of 725 mammal species whose prion proteins have been analyzed in the study.

ORDER	BINOMIAL NAME	COMMON NAME	ACCESSION No. (Link)
Afrosoricida	*Chrysochloris asiatica*	Cape golden mole	XM_006870413
	Amblysomus hottentotus	Hottentot golden mole	AY133061
	Echinops telfairi	Lesser hedgehog tenrec	BK064944
	Microgale talazaci	Talazac's shrew tenrec	BK063959
	Tenrec ecaudatus	Tailless tenrec	AY133060
Artiodactyla	*Aepyceros melampus*	Impala	BK063929
	Antilocapra americana	Pronghorn	AF156187
	Antilope cervicapra	Blackbuck	AY720706
	Antidorcas marsupialis	Springbok	BK064068
	Eudorcas thomsonii	Thomson's gazelle	EU032301
	Gazella dorcas	Dorcas gazelle	OR47264
	Gazella subgutturosa	Goitered gazelle	AF117313
	Nanger dama	Dama gazelle	OR472459
	Nanger granti	Grant's gazelle	BK064108
	Procapra przewalskii	Przewalski's gazelle	BK064131
	Babyrousa celebensis	North Sulawesi babirusa	BK064782
	Bos grunniens	Domestic yak	KC137646
	Bos taurus	Cow	NM_181015
	Bison bison	American bison	AY720696
	Bison bonasus	European bison	EU032298
	Bos frontalis	Gayal	AB534901
	Bos javanicus	Banteng	AY720693
	Boselaphus tragocamelus	Nilgai	AY720700
	Bubalus bubalis	Water buffalo	MK342630
	Bubalus depressicornis	Anoa	BK063916
	Budorcas taxicolor	Takin	AB060290
	Camelus bactrianus ferus	Bactrian camel	HQ204566
	Camelus dromedarius	Camel	Y09760
	Catagonus wagneri	Chacoan peccary	BK063918
	Dicotyles tajacu	Collared peccary	OR47262
	Cervus elaphus canadensis	Elk	EU032294
	Axis porcinus	Indian hog deer	BK063963
	Cervus nippon	Sika deer	EF057409
	Przewalskium albirostris	Thorold's deer	BK064057
	Connochaetes taurinus	Blue wildebeest	EF165086
	Connochaetes gnou	Black wildebeest	OR47263
	Dama dama	Fallow deer	EF139175
	Elaphurus davidianus	Père David's deer	MW804583
	Giraffa camelopardalis reticulata	Reticulata giraffe	AF113942
	Hexaprotodon liberiensis	Pygmy hippopotamus	AB919084
	Hippopotamus amphibius	Hippopotamus	AB919083
	Hippotragus niger niger	Sable antelope	BK064056
	Addax nasomaculatus	Addax	BK063914
	Beatragus hunteri	Hirola	BK064028
	Damaliscus lunatus	Common tsessebe	BK064037
	Hippotragus equinus	Roan antelope	BK064055
	Oryx dammah	Scimitar oryx	BK064024
	Kobus ellipsiprymnus	Waterbuck	EF165087
	Kobus megaceros	Nile lechwe	EF165088
	Kobus leche leche	Lechwe	BK064060
	Litocranius walleri	Gerenuk	BK064079
	Cephalophus natalensis harveyi	Harvey's red duiker	BK064031
	Madoqua kirkii	Kirk's dik-dik	BK064081

Artiodactyla	*Neotragus moschatus*	Suni	BK064111
	Oreotragus oreotragus	Klipspringer	BK064025
	Ourebia ourebi	Oribi	BK064118
	Moschus chrysogaster	Alpine musk deer	AY723286
	Moschus berezovskii	Dwarf musk deer	BK064096
	Saiga tatarica	Saiga antelope	BK064016
	Moschus moschiferus	Siberian musk deer	BK063923
	Muntiacus muntjak	Indian muntjac	BK064098
	Muntiacus reevesi	Reeves's muntjac	KC788406
	Muntiacus crinifrons	Hairy-fronted muntjac	BK064097
	Muntiacus gongshanensis	Gongshan muntjac	BK064177
	Neotragus pygmaeus	Royal antelope	BK064112
	Odocoileus hemionus	Mule deer	MT709729
	Alces alces	Moose	MN970212
	Capreolus capreolus	Roe deer	AY639096
	Capreolus pygargus	Siberian roe deer	BK064073
	Hydropotes inermis	Water deer	MK103026
	Odocoileus virginianus	White-tailed deer	XM_020883647
	Rangifer tarandus	Reindeer	MW557845
	Rucervus eldii thamin	Eld's deer	OL961483
	Okapia johnstoni	Okapi	BK064032
	Oryx gazella	Gemsbok	BK064117
	Ovis aries	Sheep	NM_001009481
	Ammotragus lervia	Barbary sheep	EF165080
	Capra falconeri	Markhor	MG214330
	Capra hircus	Goat	XM_005688157
	Capra ibex	Alpine ibex	EF139174
	Capra pyrenaica	Iberian ibex	KT845865
	Capra sibirica	Siberian ibex	BK063969
	Capricornis crispus	Japanese serow	MG214326
	Hemitragus hylocrius	Nilgiri tahr	BK064052
	Hemitragus jayakari	Arabian tahr	MG214331
	Naemorhedus griseus	Chinese goral	MG214329
	Oreamnos americanus	Mountain goat	EF999825
	Ovibos moschatus	Muskox	AF117320
	Ovis ammon	Argali	BK064119
	Ovis dalli	Dall sheep	DQ648474
	Ovis nivicola lydekkeri	Snow sheep	BK064120
	Pantholops hodgsonii	Tibetan antelope	BK064020
	Pseudois nayaur	Bharal	BK064132
	Rupicapra pyrenaica	Pyrenean chamois	KT845868
	Ovis canadensis	Bighorn sheep	DQ648476
	Phacochoerus africanus	Common warthog	BK063968
	Potamochoerus porcus	Red river hog	OR47265
	Philantomba maxwellii	Maxwell's duiker	BK064126
	Procapra gutturosa	Mongolian gazelle	AB473615
	Raphicerus campestris	Steenbok	BK064134
	Redunca redunca	Bohor reedbuck	BK064135
	Sus scrofa	Pig	L07623
	Sylvicapra grimmia	Common duiker	BK064038
	Syncerus caffer	African buffalo	AY720686
	Taurotragus oryx	Common eland	EF165082
	Tragelaphus angasii	Lowland nyala	EU032296
	Tragelaphus buxtoni	Mountain nyala	BK064065
	Tragelaphus imberbis	Lesser kudu	AY720704
	Tragelaphus scriptus	Harnessed bushbuck	BK063937
	Tragelaphus spekii	Sitatunga	EF165083
	Tragelaphus eurycerus	Bongo	BK064137
	Tragelaphus strepsiceros	Greater kudu	EF165081
	Tragulus javanicus	Java mouse-deer	BK064004
	Tragulus kanchil	Lesser mouse-deer	BK064005

Artiodactyla	*Vicugna vicugna*	Vicuna	BK063925
	Lama glama cacsilensis	Guanaco	BK064076
	Vicugna pacos	Alpaca	XM_006207354
Carnivora	*Ailurus fulgens*	Red panda	EU341495
	Arctictis binturong	Binturong	JX218945
	Paradoxurus hermaphroditus	Asian palm civet	BK063947
	Arctocephalus gazella	Antarctic fur seal	BK064071
	Canis latrans	Coyote	JX218953
	Chrysocyon brachyurus	Maned wolf	JX218978
	Speothos venaticus	Bush dog	JX218950
	Canis lupus familiaris	Dog	KY649563
	Canis mesomelas	Black-backed jackal	BK064772
	Caracal caracal	Caracal	BK063932
	Catopuma temminckii	Asian golden cat	JX218947
	Crocuta crocuta	Spotted hyena	JX218985
	Hyaena hyaena	Striped hyena	BK064018
	Cryptoprocta ferox	Fossa	BK063948
	Erignathus barbatus	Bearded seal	BK064769
	Felis silvestris catus	Cat	EU588730
	Felis chaus	Jungle cat	BK064169
	Felis nigripes	Black-footed cat	BK064047
	Otocolobus manul	Pallas's cat	BK064223
	Gulo gulo	Wolverine	EU341500
	Eira barbara	Tayra	BK064178
	Martes flavigula	Yellow-throated marten	BK064787
	Martes foina	Beech marten	BK064788
	Martes martes	European pine marten	JX218964
	Martes zibellina	Sable	BK063939
	Taxidea taxus jeffersonii	American badger	BK064062
	Helogale parvula	Common dwarf mongoose	BK064051
	Herpestes javanicus	Javan mongoose	EU341501
	Leopardus geoffroyi	Geoffroy's cat	BK064182
	Leopardus tigrinus	Oncilla	BK064829
	Leopardus wiedii	Margay	JX218979
	Leptonychotes weddellii	Weddell seal	XM_006744312
	Lycaon pictus	African wild dog	BK064019
	Lynx pardinus	Iberian lynx	BK064941
	Acinonyx jubatus	Cheetah	XM_027069624
	Lynx canadensis	Canada lynx	XM_030310019
	Lynx lynx	Eurasian lynx	OR472456
	Prionailurus bengalensis euptilurus	Leopard cat	BK064130
	Prionailurus iriomotensis	Iriomote cat	BK064141
	Prionailurus viverrinus	Fishing cat	BK064149
	Puma concolor	Cougar	XM_025932838
	Puma yagouaroundi	Jaguarundi	BK064172
	Lynx rufus	Bobcat	EU341503
	Mellivora capensis	Honey badger	BK064009
	Mephitis mephitis	Skunk	EU341504
	Mirounga leonina	Southern elephant seal	XM_035005902
	Mirounga angustirostris	Northern elephant seal	BK064093
	Monachus schauinslandi	Hawaiian monk seal	XM_021689157
	Mungos mungo	Banded mongoose	BK064099
	Mustela erminea	Stoat	EU341505
	Aonyx cinerea	Asian small-clawed otter	BK064764
	Bassariscus astutus	Ring-tailed cat	BK064783
	Bassariscus sumichrasti	Cacomistle	BK064784
	Enhydra lutris	Sea otter	XM_022494297
	Lontra canadensis	North American river otter	XM_032859119
	Lutra lutra	European otter	JX218963

Carnivora	*Meles meles*	European badger	JX218960
	Mustela lutreola	European mink	OR472458
	Mustela nigripes	Black-footed ferret	BK064188
	Mustela nivalis	Least weasel	BK064157
	Nasua narica	White-nosed coati	BK064789
	Nasua nasua	South American coati	OR47267
	Procyon lotor	Raccoon	EU341510
	Pteronura brasiliensis	Giant otter	BK064061
	Mustela putorius	Ferret	BK064943
	Neovison vison	American mink	EF508270
	Odobenus rosmarus	Walrus	EU341508
	Arctocephalus forsteri	Australasian fur seal	BK064830
	Arctocephalus pusillus	Brown fur seal	OR47268
	Arctocephalus townsendi	Guadalupe fur seal	BK064775
	Callorhinus ursinus	Northern fur seal	XM_025876616
	Eumetopias jubatus	Steller sea lion	EU341498
	Otaria flavescens	Sea lion	JX218981
	Zalophus californianus	California sea lion	XM_027623145
	Panthera leo	Lion	EU236260
	Neofelis diardi	Sunda clouded leopard	BK064215
	Neofelis nebulosa	Clouded leopard	EU341506
	Panthera onca	Jaguar	JX218977
	Panthera pardus	Leopard	XM_019462612
	Panthera tigris	Siberian tiger	OR472455
	Panthera uncia	Snow leopard	BK064798
	Phoca vitulina	Common seal	EU341509
	Cystophora cristata	Hooded seal	JX218976
	Halichoerus grypus	Grey seal	BK064050
	Pagophilus groenlandicus	Harp seal	JX218971
	Phoca largha	Spotted seal	BK064792
	Pusa sibirica	Baikal seal	BK064225
	Potos flavus	Kinkajou	BK063964
	Proteles cristata	Aardwolf	BK064008
	Pusa hispida saimensis	Ringed seal	BK064218
	Spilogale gracilis	Western spotted skunk	BK064000
	Spilogale putorius interrupta	Eastern spotted skunk	BK064191
	Suricata suricatta	Meerkat	XP_029772432
	Tremarctos ornatus	Spectacled bear	BK064152
	Ursus arctos	Brown bear	JX218961
	Ailuropoda melanoleuca	Giant panda	XM_034640276
	Helarctos malayanus	Sun bear	OR47260
	Ursus americanus	Black bear	EU341512
	Ursus maritimus	Polar bear	BK063942
	Ursus thibetanus thibetanus	Black bear	BK063926
	Vulpes vulpes	Red fox	XM_026012328
	Canis adustus	Side-striped jackal	BK064957
	Nyctereutes procyonoides	Raccoon dog	EU341507
	Otocyon megalotis	Bat-eared fox	BK064006
	Urocyon cinereoargenteus	Gray fox	BK064774
	Urocyon littoralis	Island fox	BK064773
	Vulpes corsac	Corsac fox	MN381732
	Vulpes lagopus	Arctic fox	EU365392
	Vulpes velox	Swift fox	EU341513
	Vulpes zerda	Fennec fox	OR47269

Cetacea	*Balaenoptera physalus*	Whale	DQ884475
	Balaenoptera acutorostrata	Common minke whale	XM_007191701
	Balaenoptera bonaerensis	Antarctic minke whale	BK064027
	Balaenoptera borealis	Sei whale	BK064828
	Balaenoptera edeni	Bryde's whale	BK064766

Cetacea	*Balaenoptera musculus*	Blue whale	BK064013
	Balaenoptera ricei	Rice's whale	BK064217
	Caperea marginata	Pygmy right whale	BK064226
	Eschrichtius robustus	Gray whale	BK064015
	Eubalaena australis	Southern right whale	BK064770
	Eubalaena glacialis	North Atlantic right whale	BK064771
	Eubalaena japonica	North Pacific right whale	BK064042
	Megaptera novaeangliae	Humpback whale	BK064082
	Delphinapterus leucas	Beluga whale	XM_022556485
	Lagenorhynchus acutus	Atlantic white-sided dolphin	BK064776
	Lagenorhynchus albirostris	White-beaked dolphin	BK064229
	Monodon monoceros	Narwhal	XM_029221065
	Orcinus orca	Orca	XM_012535450
	Phocoena phocoena	Harbour porpoise	AB919069
	Phocoenoides dalli	Dall's porpoise	AB919068
	Hyperoodon ampullatus	Northern bottlenose whale	BK064204
	Inia geoffrensis	Amazon river dolphin	BK064059
	Lagenorhynchus obliquidens	Pacific white-sided dolphin	XM_027118243
	Lipotes vexillifer	Baiji	XM_007463977
	Mesoplodon bidens	Sowerby's beaked whale	BK064083
	Mesoplodon densirostris	Blainville's beaked whale	BK064778
	Mesoplodon europaeus	Gervais' beaked whale	BK064779
	Mesoplodon stejnegeri	Stejneger's beaked whale	BK064780
	Neophocaena phocaenoides asiaeorientalis	Narrow-ridged finless porpoise	XM_024764249
	Phocoena sinus	Vaquita	XM_032605446
	Physeter catodon	Sperm whale	XM_007123285
	Kogia breviceps	Pygmy sperm whale	AB919063
	Kogia sima	Dwarf sperm whale	AB919064
	Platanista gangetica	South Asian river dolphin	BK064007
	Platanista minor	South Asian river dolphin	BK064144
	Pontoporia blainvillei	La Plata dolphin	BK064129
	Tursiops truncatus	Dolphin	XM_019941481
	Cephalorhynchus commersonii	Commerson's dolphin	BK064767
	Delphinus delphis	Dolphin	DQ884473
	Feresa attenuata	Pygmy killer whale	AB919061
	Globicephala melas	Long-finned pilot whale	XM_030872630
	Grampus griseus	Dolphin	DQ884471
	Peponocephala electra	Melon-headed whale	AB919067
	Sousa chinensis	Indo-Pacific humpback dolphin	BK064136
	Stenella attenuata	Pantropical spotted dolphin	BK064799
	Stenella clymene	Clymene dolphin	BK064800
	Stenella coeruleoalba	Dolphin	DQ884470
	Stenella frontalis	Atlantic spotted dolphin	BK064801
	Stenella longirostris	Spinner dolphin	AB919073
	Steno bredanensis	Rough-toothed dolphin	DQ884468
	Tursiops aduncus	Indo-Pacific bottlenose dolphin	AB919075
	Ziphius cavirostris	Ziphius	DQ884467
Chiroptera	*Anoura caudifer*	Tailed tailless bat	BK064067
	Antrozous pallidus	Pallid bat	BK064069
	Artibeus jamaicensis	Jamaican fruit bat	BK064072
	Carollia perspicillata	Seba's short-tailed bat	BK063935
	Corynorhinus townsendii	Townsend's big-eared bat	BK064211
	Craseonycteris thonglongyai	Kitti's hog-nosed bat	BK063940
	Cynopterus sphinx	Indian short-nosed fruit bat	AY133046
	Cynopterus brachyotis	Lesser short-nosed fruit bat	BK063952
	Desmodus rotundus	Common vampire bat	BK064947
	Eidolon helvum	Straw-coloured fruit bat	BK064039
	Eidolon dupreanum	Madagascan fruit bat	BK064786
	Eonycteris spelaea	Cave nectar bat	BK064041

Chiroptera	*Eptesicus fuscus*	Big brown bat	BK064945
	Hipposideros armiger	Great roundleaf bat	XM_019644637
	Hipposideros turpis pendleburyi	Pendlebury's roundleaf bat	BK064185
	Hipposideros galeritus	Cantor's roundleaf bat	BK064054
	Ia io	Great evening bat	BK064208
	Lasiurus cinereus	Hoary bat	BK063928
	Corynorhinus rafinesquii	Rafinesque's big-eared bat	BK064814
	Lasiurus borealis	Eastern red bat	BK064077
	Macroglossus sobrinus	Long-tongued fruit bat	BK063991
	Macrotus californicus	California leaf-nosed bat	AY133049
	Megaderma lyra	Greater false vampire bat	BK064080
	Micronycteris hirsuta	Hairy big-eared bat	BK064088
	Miniopterus natalensis	Natal long-fingered bat	XM_016212061
	Miniopterus schreibersii	Common bent-wing bat	BK064092
	Molossus molossus	Velvety free-tailed bat	KAF6472180
	Molossus alvarezi	Alvarez's mastiff bat	BK064952
	Molossus rufus	Northern black mastiff bat	BK064213
	Mormoops blainvillei	Antillean ghost-faced bat	BK063931
	Murina aurata feae	Little tube-nosed bat	BK064100
	Myotis brandtii	Brandt's bat	XM_005874678
	Myotis lucifugus	Little brown bat	XM_006096427
	Myotis davidii	David's myotis	XM_006758058
	Myotis daubentonii	Daubenton's bat	AY133048
	Myotis myotis	Greater mouse-eared bat	BK064107
	Myotis ricketti	Rickett's big-footed bat	BK064146
	Myotis septentrionalis	Northern long-eared bat	BK064803
	Myotis yumanensis	Yuma myotis	BK064222
	Noctilio leporinus	Greater bulldog bat	BK064113
	Nycticeius humeralis	Evening bat	BK064115
	Phyllostomus discolor	Pale spear-nosed bat	XP_035865486
	Pipistrellus kuhlii	Kuhl's pipistrelle	XM_036417932
	Pipistrellus pipistrellus	Common pipistrelle	BK064127
	Plecotus auritus	Lump-nosed bat	OR472453
	Pteronotus parnellii	Parnell's mustached bat	BK063967
	Pteropus alecto	Black flying fox	XM_015597302
	Pteropus medius	Indian flying fox	XM_039863787
	Pteropus pselaphon	Bonin flying fox	BK063965
	Pteropus rodricensis	Rodrigues flying fox	BK064817
	Pteropus rufus	Madagascan flying fox	BK064809
	Pteropus vampyrus	Large flying fox	XM_011369934
	Rhinolophus ferrumequinum	Greater horseshoe bat	BK063922
	Rhinolophus sinicus	Chinese rufous horseshoe bat	BK063992
	Rousettus aegyptiacus	Egyptian fruit bat	XM_016135122
	Rousettus leschenaultii	Leschenault's rousette	BK063997
	Rousettus madagascariensis	Madagascan rousette	BK064805
	Sturnira ludovici hondurensis	Honduran yellow-shouldered bat	BK063970
	Tadarida brasiliensis	Mexican free-tailed bat	BK064001
	Tonatia saurophila	Stripe-headed round-eared bat	BK064003
	Phyllostomus hastatus	Greater spear-nosed bat	BK064158
	Trachops cirrhosus	Fringe-lipped bat	BK064958
Cingulata	*Cabassous unicinctus*	Southern naked-tailed armadillo	BK064228
	Chaetophractus vellerosus	Screaming hairy armadillo	BK064063
	Euphractus sexcinctus	Six-banded armadillo	OR47276
	Dasypus novemcinctus	Nine-banded armadillo	EU559337
	Tolypeutes matacus	Southern three-banded armadillo	BK064002
Dasyuromorphia	*Antechinus flavipes*	Yellow-footed antechinus	BK063971
	Antechinus stuartii	Brown antechinus	BK063930

Dasyuromorphia	*Dasyurus hallucatus*	Northern quoll	BK064760
	Dasyurus viverrinus	Eastern quoll	BK064810
	Myrmecobius fasciatus	Numbat	BK064811
	Phascogale tapoatafa	Brush-tailed phascogale	BK064816
	Phascogale calura	Red-tailed phascogale	BK064832
	Sarcophilus harrisii	Tasmanian devil	XM_003757949
	Sminthopsis crassicaudata	Fat-tailed dunnart	BK064171
	Thylacinus cynocephalus	Thylacine	BK064189
Dermoptera	*Galeopterus variegatus*	Sunda flying lemur	AY133034
	Cynocephalus volans	Philippine flying lemur	BK064216
Didelphimorphia	*Didelphis virginiana*	Virginia opossum	BK064768
	Gracilinanus agilis	Agile gracile opossum	BK063958
	Monodelphis domestica	Opossum	NM_001040028
Diprotodontia	*Bettongia penicillata ogilbyi*	Woylie	BK064808
	Cercartetus concinnus	Western pygmy possum	BK064813
	Dendrolagus matschiei	Matschie's tree-kangaroo	BK064823
	Gymnobelideus leadbeateri	Leadbeater's possum	BK064074
	Lagorchestes hirsutus	Rufous hare-wallaby	BK064777
	Macropus eugenii	Wallaby	AY659988
	Macropus irma	Western Brush Wallaby	BK064824
	Macropus rufogriseus	Red-necked wallaby	OR47270
	Macropus giganteus	Eastern grey kangaroo	BK064781
	Macropus fuliginosus	Western grey kangaroo	BK064820
	Macropus rufus	Red kangaroo	OR47271
	Petaurus breviceps	Sugar glider	BK064219
	Petrogale xanthopus	Yellow-footed rock-wallaby	OR47266
	Wallabia bicolor	Swamp wallaby	BK064825
	Phalanger gymnotis	Ground cuscus	BK064791
	Phascolarctos cinereus	Koala	XM_020985342
	Potorous gilbertii	Gilbert's potoroo	BK064793
	Pseudocheirus peregrinus occidentalis	Western ringtail	BK064794
	Pseudochirops corinnae	Plush-coated ringtail possum	BK064795
	Pseudochirops cupreus	Coppery ringtail possum	BK064796
	Setonix brachyurus	Quokka	BK064765
	Trichosurus vulpecula	Common brushtail possum	L38993
	Vombatus ursinus	Common wombat	XM_027865324
Erinaceomorpha	*Atelerix albiventris*	Four-toed hedgehog	OR47273
	Erinaceus europaeus	European hedgehog	EU572708
	Hylomys suillus	Short-tailed gymnure	AY133044
Hyracoidea	*Procavia capensis*	Rock hyrax	AY133057
	Heterohyrax brucei	Yellow-spotted rock hyrax	BK064053
Lagomorpha	*Lepus europaeus*	Brown hare	OR472454
	Lepus americanus	Snowshoe hare	BK063972
	Lepus timidus	Mountain hare	BK063973
	Lepus townsendii	White-tailed jackrabbit	BK064227
	Ochotona princeps	American pika	EU555402
	Ochotona curzoniae	Plateau pika	MT371369
	Oryctolagus cuniculus	Rabbit	NM_001082021
	Sylvilagus bachmani	Brush rabbit	BK063974

Lagomorpha	*Sylvilagus transitionalis*	New England cottontail	BK064962
Macroscelidea	*Elephantulus edwardii*	Cape elephant shrew	XM_006894222
	Macroscelides proboscideus	Round-eared elephant shrew	AY133059
Microbiotheria	*Dromiciops gliroides*	Colocolo opossum	BK064161
	Dromiciops bozinovici	Pancho's colocolo opossum	OR47274
Monotremata	*Ornithorhynchus anatinus*	Platypus	XM_029065640
	Tachyglossus aculeatus	Short-beaked echidna	BK063950
Notoryctemorphia	*Notoryctes typhlops*	Southern marsupial mole	BK064812
Perissodactyla	*Ceratotherium simum simum*	Southern white rhinoceros	XM_014790100
	Dicerorhinus sumatrensis harrissoni	Bornean rhinoceros	BK063994
	Diceros bicornis	Black rhinoceros	AY133052
	Rhinoceros unicornis	White rhinoceros	BK064948
	Equus asinus	Asinus	BK064942
	Equus africanus	African wild ass	OR47283
	Equus caballus ferus	Horse	EU887260
	Equus burchellii	Burchell's zebra	EF165074
	Equus grevyi	Grévy's zebra	BK064961
	Equus kiang	Kiang	EF165076
	Equus quagga boehmi	Grant's zebra	AF117329
	Equus zebra hartmannae	Mountain zebra	EF165073
	Equus caballus przewalskii	Przewalski's horse	XM_008519788
	Tapirus indicus	Malayan tapir	BK063977
	Tapirus terrestris	South American tapir	BK063978
	Tapirus bairdii	Baird's tapir	BK064815
Pholidota	*Manis javanica*	Pangolin	XM_017663893
	Manis crassicaudata	Chinese pangolin	BK063961
	Manis pentadactyla	Chinese pangolin	BK063962
	Manis tricuspis	Tree pangolin	BK063960
Pilosa	*Bradypus variegatus*	Brown-throated sloth	BK063934
	Choloepus didactylus	Linnaeus's two-toed sloth	BK063945
	Choloepus hoffmanni	Hoffmann's two-toed sloth	BK063946
	Cyclopes didactylus	Silky anteater	AY133063
	Myrmecophaga tridactyla	Giant anteater	BK063979
	Tamandua tetradactyla	Southern tamandua	BK063980
Primates	*Alouatta belzebul*	Red-handed howler	AY765382
	Alouatta palliata	Mantled howler	BK064066
	Aotus lemurinus	Gray-bellied night monkey	AY765387
	Aotus nancymaae	Nancy Ma's night monkey	XM_012455393
	Aotus trivirgatus	Three-striped night monkey	U08293
	Ateles geoffroyi	Geoffroy's spider monkey	U08309
	Ateles hybridus	Brown spider monkey	BK064173
	Ateles paniscus	Red-faced spider monkey	U15164
	Brachyteles arachnoides	Southern muriqui	AY765383
	Callicebus donacophilus	White-eared titi	BK064142
	Callicebus moloch	Red-bellied titi	U08312

Primates	*Callithrix jacchus*	Common marmoset	XM_008995703
	Callimico goeldii	Goeldi's marmoset	AY765390
	Callithrix argentata	Silvery marmoset	OR47282
	Callithrix geoffroyi	White-headed marmoset	OR47261
	Callithrix pygmaea	Pygmy marmoset	AY765392
	Cebus capucinus	Panamanian white-faced capuchin	XM_017524036
	Cebus albifrons	Humboldt's white-fronted capuchin	BK064029
	Cebus apella	Tufted capuchin	XM_032295507
	Cercocebus atys	Sooty mangabey	XM_012052159
	Cercocebus torquatus	Collared mangabey	U75385
	Cheirogaleus medius	Fat-tailed dwarf lemur	BK063936
	Chiropotes satanas	Black bearded saki	AY765380
	Cacajao calvus	Bald uakari	AY765381
	Colobus angolensis palliatus	Angola colobus	XM_011941048
	Colobus guereza	Mantled guereza	U75389
	Nasalis larvatus	Proboscis monkey	BK064109
	Piliocolobus tephrosceles	Ugandan red colobus	XM_023217934
	Pygathrix nemaeus	Red-shanked douc	BK064133
	Pygathrix nigripes	Black-shanked douc	BK064197
	Rhinopithecus bieti	Black snub-nosed monkey	XM_017878075
	Rhinopithecus roxellana	Golden snub-nosed monkey	BK063920
	Rhinopithecus strykeri	Myanmar snub-nosed monkey	BK064200
	Semnopithecus entellus	Northern plains gray langur	BK064033
	Trachypithecus francoisi	François' langur	XM_033185731
	Trachypithecus phayrei crepuscula	Phayre's leaf monkey	BK064195
	Daubentonia madagascariensis	Aye-aye	BK064012
	Galago moholi	Mohol bushbaby	BK064194
	Gorilla gorilla	Western gorilla	U08300
	Homo sapiens	Human	NM_001080123
	Hoolock leuconedys	Eastern hoolock gibbon	BK064201
	Indri indri	Indri	BK064058
	Lagothrix lagotricha	Brown woolly monkey	AY765384
	Leontopithecus rosalia	Golden lion tamarin	BK063917
	Loris tardigradus	Golden-headed lion tamarin	BK064822
	Loris tardigradus	Red slender loris	BK064198
	Macaca nemestrina	Pigtail macaque	XM_011741222
	Allenopithecus nigroviridis	Allen's swamp monkey	BK064763
	Cercopithecus albogularis	Sykes' monkey	BK064193
	Cercopithecus diana	Guenons	U08292
	Cercopithecus mona	Mona monkey	U75386
	Cercopithecus neglectus	DeBrazza's monkey	U75387
	Chlorocebus aethiops	Vervet monkey	U08291
	Chlorocebus sabaeus	Green monkey	XM_008019068
	Erythrocebus patas	Patas monkey	U75388
	Lophocebus aterrimus	Black crested mangabey	U75384
	Macaca arctoides	Stump-tailed macaque	U08311
	Macaca assamensis	Assam macaque	EF455529
	Macaca cyclopis	Formosan rock macaque	BK064212
	Macaca fascicularis	Crab-eating macaque	U08298
	Macaca fuscata	Japanese macaque	U08301
	Macaca mulatta	Rhesus macaque	NM_001047152
	Macaca silenus	Lion-tailed macaque	BK064196
	Macaca sylvanus	Barbary macaque	U75382
	Papio anubis	Olive baboon	XM_031656585
	Papio hamadryas	Hamadryas baboon	U08294
	Papio papio	Guinea baboon	BK064790
	Mandrillus leucophaeus	Drill	XM_011975232
	Mandrillus sphinx	Mandrill	U08303
	Microcebus murinus	Gray mouse lemur	NM_001309920
	Microcebus griseorufus	Reddish-gray mouse lemur	BK064084
	Microcebus mittermeieri	Mittermeier's mouse lemur	BK064085

Primates	*Microcebus tavaratra*	Northern rufous mouse lemur	BK064087
	Microcebus ravelobensis	Golden-brown mouse lemur	BK064086
	Miopithecus talapoin	Angolan talapoin	BK064818
	Mirza coquereli	Coquerel's giant mouse lemur	BK064094
	Mirza zaza	Northern giant mouse lemur	BK064095
	Nycticebus coucang	Sunda slow loris	BK064114
	Nycticebus bengalensis	Bengal slow loris	BK064202
	Nycticebus pygmaeus	Pygmy slow loris	OR47281
	Otolemur garnettii	Northern greater galago	XM_003788036
	Pan troglodytes	Chimpanzee	U08296
	Hylobates agilis	Agile gibbon	BK064785
	Hylobates lar	Lar gibbon	U08299
	Hylobates moloch	Silvery gibbon	XM_032143110
	Hylobates muelleri	Müller's gibbon	OR472457
	Hylobates pileatus	Pileated gibbon	BK064186
	Nomascus leucogenys	Northern white-cheeked gibbon	XM_012511857
	Nomascus siki	Southern white-cheeked gibbon	BK064199
	Pan paniscus	Bonobo	BK064946
	Symphalangus syndactylus	Siamang	U08308
	Pithecia irrorata	Rio Tapajós saki	AY765379
	Pithecia pithecia	White-faced saki	BK064128
	Pongo pygmaeus	Bornean orangutan	U08305
	Pongo abelii	Sumatran orangutan	XM_009233395
	Prolemur simus	Greater bamboo lemur	BK063921
	Eulemur albifrons	White-headed lemur	OR47279
	Eulemur fulvus	Common brown lemur	BK064044
	Eulemur macaco	Black lemur	BK064045
	Eulemur mongoz	Mongoose lemur	BK064802
	Lemur catta	Ring-tailed lemur	BK064078
	Propithecus coquereli	Coquerel's sifaka	XM_012639270
	Saguinus bicolor	Pied tamarin	AY765388
	Saguinus imperator	Emperor tamarin	BK064034
	Saguinus midas	Golden-handed tamarin	BK064187
	Saimiri boliviensis	Black-capped squirrel monkey	BK064035
	Saimiri sciureus	Squirrel monkey	U15165
	Tarsius syrichta	Philippine tarsier	XM_021710036
	Cephalopachus bancanus	Horsfield's tarsier	BK064214
	Theropithecus gelada	Gelada	XM_025399776
Proboscidea	*Loxodonta africana*	Elephant	EU588731
	Elephas maximus	Asian elephant	AY133055
Rodentia	*Acomys cahirinus*	Cairo spiny mouse	EF467171
	Allactaga bullata	Gobi jerboa	BK064143
	Aplodontia rufa	Mountain beaver	BK064070
	Apodemus fulvipectus	Steppe field mouse	KF466953
	Arvicanthis niloticus	African grass rat	XM_034493892
	Arvicola amphibius	European water vole	BK063953
	Microtus gerbei	Gerbe's vole	OR47278
	Bandicota indica	Greater bandicoot rat	KF466950
	Bandicota savilei	Savile's bandicoot rat	KF466951
	Berylmys berdmorei	Small white-toothed rat	KF466946
	Leopoldamys edwardsi	Edwards's long-tailed giant rat	KF466944
	Leopoldamys sabanus	Long-tailed giant rat	KF466943
	Rattus exulans	Polynesian rat	KF466949
	Capromys pilorides	Desmarest's hutia	BK064046
	Castor canadensis	North American beaver	XM_020184027
	Cavia aperea	Brazilian guinea pig	KM357834
	Cavia porcellus	Guinea pig	XM_003476602

Rodentia	*Cavia tschudii*	Montane guinea pig	BK063938
	Chinchilla lanigera	Long-tailed chinchilla	XM_005380818
	Coendou prehensilis	Brazilian porcupine	OR47272
	Cricetomys gambianus	Gambian pouched rat	BK063941
	Cricetomys ansorgei	Southern giant pouched rat	BK064819
	Cricetulus barabensis	Chinese hamster	M33958
	Cricetulus migratorius	Armenian hamster	M33959
	Cryptomys damarensis	Damaraland mole-rat	XM_010607544
	Cryptomys darlingi	Mashona mole-rat	BK064181
	Ctenodactylus gundi	Common gundi	BK063949
	Ctenomys sociabilis	Social tuco-tuco	BK063951
	Dasyprocta punctata	Central American agouti	BK063998
	Dinomys branickii	Pacarana	BK063954
	Dipodomys merriami	Merriam's kangaroo rat	BK064205
	Dipodomys ordii	Ord's kangaroo rat	XM_013010955
	Dipodomys spectabilis	Banner-tailed kangaroo rat	BK064155
	Dipodomys stephensi	Stephens' kangaroo rat	BK064064
	Dolichotis patagonum	Patagonian mara	KM357833
	Ellobius lutescens	Transcaucasian mole vole	BK064014
	Ellobius talpinus	Northern mole vole	BK064040
	Eospalax fontanierii	Chinese zokor	BK064949
	Erethizon dorsata	North American porcupine	BK063957
	Glaucomys volans	Southern flying squirrel	BK064183
	Glis glis	Edible dormouse	BK064048
	Grammomys surdaster	African woodland thicket rat	XM_028757107
	Graphiurus murinus	Woodland dormouse	BK064049
	Heterocephalus glaber	Naked mole-rat	XM_004840771
	Hydrochoerus hydrochaeris	Capybara	KM357831
	Kerodon rupestris	Rock cavy	KM357832
	Hylomyscus alleni	Allen's wood mouse	BK064167
	Hystrix brachyura	Malayan porcupine	BK063933
	Hystrix cristata	Crested porcupine	BK063944
	Jaculus jaculus	Lesser Egyptian jerboa	BK064939
	Lophiomys imhausi	Maned rat	BK064153
	Marmota flaviventris	Yellow-bellied marmot	XM_027955665
	Marmota monax	Groundhog	XM_046433852
	Marmota vancouverensis	Vancouver Island marmot	BK063989
	Marmota marmota	Alpine marmot	XM_015482632
	Marmota himalayana	Himalayan marmot	BK063993
	Mastomys coucha	Southern multimammate mouse	XM_031371966
	Mastomys natalensis	Natal multimammate mouse	BK064165
	Tokudaia muenninki	Muennink's spiny rat	BK064955
	Tokudaia osimensis	Ryukyu spiny rat	BK064210
	Tokudaia tokunoshimensis	Tokunoshima spiny rat	BK064956
	Maxomys surifer	Red spiny rat	KF466947
	Mus pahari	Gairdner's shrewmouse	XM_021192940
	Meriones libycus	Libyan jird	BK064826
	Meriones unguiculatus	Mongolian gerbil	XM_021651076
	Rhombomys opimus	Great gerbil	BK063996
	Mesocricetus auratus	Syrian hamster	AH001830
	Mesocricetus brandti	Turkish hamster	EU886369
	Microtus agrestis	Field vole	AF367625
	Microtus arvalis	Common vole	BK064089
	Microtus fortis	Reed vole	BK064090
	Microtus ochrogaster	Prairie vole	XM_005365568
	Microtus oeconomus	Tundra vole	BK064091
	Microtus oregoni	Creeping vole	BK064150
	Microtus pennsylvanicus	Meadow vole	GQ850541
	Microtus californicus	California vole	BK064221
	Microtus montanus	Montane vole	BK064179
	Microtus richardsoni	Water vole	BK064156

Rodentia	*Mus musculus*	Mouse	NM_011170
	Apodemus mystacinus	Eastern broad-toothed field mouse	KF466942
	Apodemus speciosus	Large Japanese field mouse	BK064145
	Apodemus sylvaticus	Wood mouse	AF367623
	Chiropodomys gliroides	Indomalayan pencil-tailed tree mouse	KF466945
	Hydromys chrysogaster	Rakali	BK064827
	Mastacomys fuscus	Broad-toothed mouse	BK064762
	Mus caroli	Ryukyu mouse	XM_021185802
	Mus cervicolor	Fawn-colored mouse	KF466939
	Mus cookii	Cook's mouse	KF466940
	Mus fragilicauda	Sheath-tailed mouse	KF466956
	Mus minutoides	African pygmy mouse	BK064101
	Mus spicilegus	Steppe mouse	BK063924
	Mus spretus	Algerian mouse	BK064105
	Praomys delectorum	Delectable soft-furred mouse	BK064166
	Pseudomys desertor	Desert mouse	BK064761
	Pseudomys fumeus	Smoky mouse	BK064821
	Muscardinus avellanarius	Hazel dormouse	BK064106
	Myocastor coypus	Coypu	BK064010
	Myodes gapperi	Southern red-backed vole	GQ850538
	Myodes glareolus	Bank vole	AF367624
	Neodon shergylaensis	Shergylaensis vole	BK064207
	Neotoma lepida	Desert woodrat	BK064110
	Octodon degus	Common degu	XM_023718589
	Octomys mimax	Mountain viscacha rat	BK064011
	Ondatra zibethicus	Muskrat	BK064026
	Dicrostonyx torquatus	Arctic lemming	BK064224
	Pedetes capensis	South African springhare	BK064021
	Perognathus longimembris pacificus	Little pocket mouse	BK064022
	Peromyscus attwateri	Texas mouse	BK064023
	Peromyscus nasutus	Northern rock mouse	BK064804
	Peromyscus californicus insignis	California mouse	BK064122
	Peromyscus eremicus	Cactus mouse	BK064123
	Peromyscus crinitus	Canyon mouse	BK064807
	Peromyscus leucopus	White-footed mouse	XM_028878611
	Abrothrix hirta	Long-haired grass mouse	BK064176
	Abrothrix longipilis hirtus	Long-haired grass mouse	BK064147
	Onychomys torridus	Southern grasshopper mouse	XM_036185260
	Peromyscus aztecus	Aztec mouse	BK064121
	Peromyscus melanophrys	Plateau mouse	BK064124
	Peromyscus mexicanus	Mexican deer mouse	BK064125
	Peromyscus polionotus subgriseus	Oldfield mouse	EF467170
	Peromyscus maniculatus bairdii	Deer mouse	XM_006983992
	Phodopus roborovskii	Roborovski hamster	OR47275
	Petaurista alborufus	Red and white giant flying squirrel	KM357835
	Petromus typicus	Dassie rat	BK063990
	Phodopus campbelli	Campbell's dwarf hamster	EU886367
	Phodopus sungorus	Russian hamster	EU886368
	Psammomys obesus	Fat sand rat	BK063966
	Rattus argentiventer	Ricefield rat	KF466955
	Rattus norvegicus	Brown rat	BK063913
	Rattus losea	Lesser ricefield rat	KF466954
	Rattus nitidus	Himalayan field rat	KF466952
	Rattus rattus	Rat	XM_032904254
	Rattus tanezumi	Tanezumi rat	KF466948
	Rhabdomys dilectus	Mesic four-striped grass rat	BK064164
	Rhabdomys pumilio	Four-striped grass mouse	BK064232
	Rhizomys pruinosus	Hoary bamboo rat	BK063999
	Rhynchomys soricoides	Mount Data shrew-rat	BK064163
	Saxatilomys paulinae	Paulina’s limestone rat	KF466958
	Sciurus carolinensis	Eastern gray squirrel	BK064159

Rodentia	*Sciurus niger*	Fox squirrel	BK064184
	Sciurus stramineus	Guayaquil squirrel	KM357836
	Sciurus vulgaris	Red squirrel	AY133037
	Sciurus lis	Japanese squirrel	FN678794
	Xerus inauris	Cape ground squirrel	BK064148
	Sigmodon fulviventer	Tawny-bellied cotton rat	AF117324
	Sigmodon hispidus	Hispid cotton rat	BK064036
	Spalax galili	Upper Galilee Mountains blind mole-rat	XM_008835893
	Spermophilus beecheyi	California ground squirrel	BK064192
	Spermophilus dauricus	Daurian ground squirrel	BK063919
	Spermophilus parryii	Arctic ground squirrel	XM_026385776
	Spermophilus tridecemlineatus	Thirteen-lined ground squirrel	XM_005320503
	Cynomys gunnisoni	Gunnison's prairie dog	BK064140
	Cynomys ludovicianus	Black-tailed prairie dog	OR47277
	Tamias sibiricus	Siberian chipmunk	BK064209
	Thomomys bottae	Botta's pocket gopher	BK064206
	Thryonomys swinderianus	Greater cane rat	BK064162
	Tympanoctomys barrerae	Plains viscacha rat	BK064138
	Typhlomys cinereus	Chinese pygmy dormouse	BK064190
	Uromys caudimaculatus	Giant white-tailed rat	BK064806
	Xerus rutilus	Unstriped ground squirrel	BK064797
	Zapus hudsonius	Meadow jumping mouse	BK064139
Scandentia	*Tupaia belangeri chinensis*	Chinese tree shrew	XM_006163978
	Tupaia tana	Large treeshrew	AY133035
Sirenia	*Dugong dugon*	Dugong	BK063981
	Hydrodamalis gigas	Steller's sea cow	BK063982
	Trichechus manatus	West Indian manatee	AY133056
Soricomorpha	*Condylura cristata*	Star-nosed mole	XM_012728373
	Crocidura russula	Greater white-toothed shrew	OR47280
	Galemys pyrenaicus	Pyrenean desman	BK063927
	Scalopus aquaticus	Eastern mole	BK063985
	Solenodon paradoxus woodi	Hispaniolan solenodon	BK063984
	Sorex araneus	Common shrew	BK064940
	Sorex maritimensis	Maritime shrew	BK064231
	Sorex cinereus	Cinereous shrew	BK063915
	Sorex fumeus	Smoky shrew	BK064954
	Sorex palustris	American water shrew	BK064220
	Suncus etruscus	Etruscan shrew	BK064203
	Talpa europaea	European mole	AY133042
	Talpa occidentalis	Spanish mole	BK063986
	Uropsilus gracilis	Gracile shrew mole	BK063987
Tubulidentata	Orycteropus afer	Aardvark	AY133058

The species have been classified by their respective orders and are then arranged alphabetically based on their binomial nomenclature. The list includes common names and the corresponding Genbank accession numbers for their PrP sequences, each of which is linked to Genbank for further reference. Newly introduced accession numbers for this particular study are highlighted in green. Species sharing the same PrP sequence as the reference species are highlighted in blue for easy identification.

Supplementary table 2. Comprehensive data for 382 analyzed species: Misfolding scores, structural and biochemical insights, and sequencing details (linked to PrPdex database)

BINOMIAL NAME	COMMON NAME	ORDER	AA No.[a]	Conf No.[b]	Stability[c]	Score[d]	PDB[e]	PrPdex[f]
Arvicanthis niloticus	African grass rat	Rodentia	5	2	-5.57	100	2544	2544
Bandicota indica	Greater bandicoot rat	Rodentia	4	1	-9.35	100	1340	1340
Cervus elaphus canadensis	Elk	Artiodactyla	10	2	-11.73	100	1178	1178
Dama dama	Fallow deer	Artiodactyla	11	3	-10.8	100	0980	0980
Dipodomys stephensi	Stephens' kangaroo rat	Rodentia	11	4	ND	100	3240	3240
Ellobius lutescens	Transcaucasian mole vole	Rodentia	1	1	6.65	100	3175	3175
Ellobius talpinus	Northern mole vole	Rodentia	1	1	3.73	100	3206	3206
Lycaon pictus	African wild dog	Carnivora	11	1	-6.06	100	3183	3183
Maxomys surifer	Red spiny rat	Rodentia	3	1	-0.97	100	1440	1440
Mellivora capensis	Honey badger	Carnivora	12	3	-6.43	100	3170	3170
Meriones unguiculatus	Mongolian gerbil	Rodentia	3	1	-3.71	100	1111	1111
Microtus agrestis	Field vole	Rodentia	1	1	-1.99	100	1423	1423
Microtus arvalis	Common vole	Rodentia	1	1	2.34	100	3268	3268
Microtus ochrogaster	Prairie vole	Rodentia	2	1	-13.33	100	1094	1094
Microtus oeconomus	Tundra vole	Rodentia	1	1	-3.36	100	3270	3270
Mustela putorius	Ferret	Carnivora	10	2	-8.29	100	1072	1072
Myodes gapperi	Southern red-backed vole	Rodentia	2	2	4.84	100	1390	1390
Myodes glareolus	Bank vole	Rodentia	0	4	0	100	0092	0092
Neotragus pygmaeus	Royal antelope	Artiodactyla	10	3	-11.29	100	3292	3292
Odocoileus hemionus	Mule deer	Artiodactyla	9	2	-21.62	100	0174	0174
Pedetes capensis	South African springhare	Rodentia	8	1	ND	100	3186	3186
Petaurista alborufus	Red and white giant flying squirrel	Rodentia	11	1	-12.03	100	1454	1454
Phodopus sungorus	Russian hamster	Rodentia	3	1	-1.18	100	0822	0822
Rattus norvegicus	Brown rat	Rodentia	4	1	-6.76	100	0825	0825
Rousettus aegyptiacus	Egyptian fruit bat	Chiroptera	11	1	-20.62	100	1107	1107
Saxatilomys paulinae	Paulina's limestone rat	Rodentia	4	1	-10.57	100	1449	1449
Tragulus javanicus	Java mouse-deer	Artiodactyla	8	1	-12.57	100	3165	3165
Microtus pennsylvanicus	Meadow vole	Rodentia	1	1	-3.72	96,4	1422	1422
Muntiacus reevesi	Reeves's muntjac	Artiodactyla	9	1	-13.83	96,4	0950	0950
Pteropus alecto	Black flying fox	Chiroptera	11	1	-6.57	96,4	1101	1101
Microtus fortis	Reed vole	Rodentia	2	3	-3.21	94,3	3269	3269
Mustela erminea	Stoat	Carnivora	11	4	-8.8	94,3	0549	0549
Cricetulus migratorius	Armenian hamster	Rodentia	4	1	-7.18	92,9	0824	0824
Dipodomys merriami	Merriam's kangaroo rat	Rodentia	11	1	1.98	92,9	4560	4560
Neotoma lepida	Desert woodrat	Rodentia	3	2	-3.66	92,9	3290	3290
Acomys cahirinus	Cairo spiny mouse	Rodentia	3	1	-2.04	91,1	1370	1370
Peromyscus californicus insignis	California mouse	Rodentia	3	1	-8.01	91,1	3303	3303
Phodopus campbelli	Campbell's dwarf hamster	Rodentia	2	1	-0.53	91,1	1433	1433
Tragelaphus angasii	Lowland nyala	Artiodactyla	11	3	-10.1	90,7	1327	1327
Elaphurus davidianus	Père David's deer	Artiodactyla	10	1	-9.46	89,3	1450	1450
Raphicerus campestris	Steenbok	Artiodactyla	10	4	-11.02	89,3	3318	3318
Tupaia tana	Large treeshrew	Scandentia	12	2	ND	89,3	1371	1371
Cynopterus sphinx	Indian short-nosed fruit bat	Chiroptera	9	2	-7.08	87,5	1156	1156
Rhizomys pruinosus	Hoary bamboo rat	Rodentia	12	2	-9.79	87,5	3159	3159
Suricata suricatta	Meerkat	Carnivora	11	2	-9.09	87,5	0939	0939
Okapia johnstoni	Okapi	Artiodactyla	11	2	-12.63	87,1	3197	3197
Boselaphus tragocamelus	Nilgai	Artiodactyla	12	1	-7.53	85,7	1357	1357
Cricetulus barabensis	Chinese hamster	Rodentia	2	2	-4.97	85,7	0835	0835
Hippotragus niger niger	Sable antelope	Artiodactyla	10	2	-11.98	85,7	3225	3225

BINOMIAL NAME	COMMON NAME	ORDER	AA No.[a]	Conf No.[b]	Stability [c]	Score [d]	PDB [e]	PrPdex [f]
Lynx rufus	Bobcat	Carnivora	12	4	-6.74	83,9	1333	1333
Canis lupus familiaris	Dog	Carnivora	11	2	-2.04	83,6	0139	0139
Grammomys surdaster	African woodland thicket rat	Rodentia	4	2	-3.65	83	1001	1001
Eidolon helvum	Straw-coloured fruit bat	Chiroptera	10	1	-16.93	82,1	3205	3205
Hipposideros armiger	Great roundleaf bat	Chiroptera	7	2	-2.36	82,1	1109	1109
Macroglossus sobrinus	Long-tongued fruit bat	Chiroptera	9	1	-14.88	82,1	3149	3149
Peromyscus leucopus	White-footed mouse	Rodentia	2	2	-1.47	82,1	1011	1011
Dipodomys ordii	Ord's kangaroo rat	Rodentia	9	3	2.83	81,8	2568	2568
Mus musculus	Mouse	Rodentia	3	2	-3.3	79,6	0113	0113
Glaucomys volans	Southern flying squirrel	Rodentia	11	3	-8.59	78,9	4022	4022
Allactaga bullata	Gobi jerboa	Rodentia	13	3	-10.12	78,6	3330	3330
Panthera leo	Lion	Carnivora	12	5	-6.42	78,6	1085	1085
Castor canadensis	North American beaver	Rodentia	9	4	ND	78,2	0940	0940
Spalax galili	Upper Galilee M.	Rodentia	7	2	-10.9	78,2	1088	1088
Tragelaphus spekii	Sitatunga	Artiodactyla	11	4	-10.2	76,8	1381	1381
Oryx gazella	Gemsbok	Artiodactyla	9	2	-11.01	76,1	3297	3297
Psammomys obesus	Fat sand rat	Rodentia	3	2	1.23	75	3096	3096
Sigmodon fulviventer	Tawny-bellied cotton rat	Rodentia	2	1	-0.82	75	1349	1349
Syncerus caffer	African buffalo	Artiodactyla	10	1	-10.06	75	1434	1434
Mastomys coucha	Southern multimammate mouse	Rodentia	3	1	-6.17	74,6	1098	1098
Callicebus donacophilus	White-eared titi	Primates	8	4	4.73	74,1	3329	3329
Kobus megaceros	Nile lechwe	Artiodactyla	11	2	-13.74	71,8	1448	1448
Muntiacus muntjak	Indian muntjac	Artiodactyla	10	1	-14.89	71,8	3277	3277
Tupaia belangeri chinensis	Chinese tree shrew	Scandentia	12	1	ND	71,8	1093	1093
Neovison vison	American mink	Carnivora	11	1	-6.64	71,4	0180	0180
Octodon degus	Common degu	Rodentia	11	2	ND	71,4	1003	1003
Philantomba maxwellii	Maxwell's duiker	Artiodactyla	9	2	-11.79	71,4	3307	3307
Taurotragus oryx	Common eland	Artiodactyla	11	3	-24.41	71,4	1379	1379
Thomomys bottae	Botta's pocket gopher	Rodentia	12	1	ND	71,4	4580	4580
Uromys caudimaculatus	Giant white-tailed rat	Rodentia	4	1	-9.36	71,4	3974	3974
Aplodontia rufa	Mountain beaver	Rodentia	12	1	-11.28	71,1	3247	3247
Ctenodactylus gundi	Common gundi	Rodentia	12	2	-6.5	71,1	3076	3076
Thryonomys swinderianus	Greater cane rat	Rodentia	9	3	ND	70,4	3776	3776
Eospalax fontanierii	Chinese zokor	Rodentia	8	4	-3.33	69,3	4002	4002
Ailurus fulgens	Red panda	Carnivora	13	2	-12.32	67,9	0948	0948
Sus scrofa	Pig	Artiodactyla	12	3	-15.4	67,9	0243	0243
Theropithecus gelada	Gelada	Primates	8	4	-8.22	67,9	1204	1204
Vulpes vulpes	Red fox	Carnivora	11	2	-8.8	67,9	1114	1114
Aepyceros melampus	Impala	Artiodactyla	10	2	-14.79	67,5	3047	3047
Callithrix jacchus	Common marmoset	Primates	7	1	-4.16	67,5	1121	1121
Rhynchomys soricoides	Mount Data shrew-rat	Rodentia	5	1	-6.81	67,5	3799	3799
Leptonychotes weddellii	Weddell seal	Carnivora	14	4	-6.26	66,1	1081	1081
Moschus moschiferus	Siberian musk deer	Artiodactyla	11	2	-11.5	65	2032	2032
Callicebus moloch	Red-bellied titi	Primates	7	6	3.98	64,3	1429	1429
Canis latrans	Coyote	Carnivora	11	2	-5.73	64,3	1353	1353
Catopuma temminckii	Asian golden cat	Carnivora	12	4	-12.45	64,3	1408	1408
Glis glis	Edible dormouse	Rodentia	12	4	-2.93	64,3	3217	3217
Alouatta belzebul	Red-handed howler	Primates	8	4	-0.92	63,6	1383	1383
Antilope cervicapra	Blackbuck	Artiodactyla	9	1	-12.66	62,5	1350	1350
Meriones libycus	Libyan jird	Rodentia	4	2	-15,45	62,5	4640	4640
Mirounga leonina	Southern elephant seal	Carnivora	14	3	-12.06	62,5	2188	2188
Neodon shergylaensis	Shergylaensis vole	Rodentia	2	1	-15,47	62,5	4584	4584
Ovis canadensis	Bighorn sheep	Artiodactyla	11	1	-9.13	62,5	1478	1478

BINOMIAL NAME	COMMON NAME	ORDER	AA No.[a]	Conf No.[b]	Stability[c]	Score[d]	PDB[e]	PrPdex[f]
Gymnobelideus leadbeateri	Leadbeater's possum	Diprotodontia	19	3	ND	60,2	3252	3252
Proteles cristata	Aardwolf	Carnivora	14	3	-10.08	59,3	3169	3169
Ovis aries	Sheep	Artiodactyla	10	2	-11.89	54,3	0195	0195
Saguinus imperator	Emperor tamarin	Primates	6	2	4.37	53,6	3200	3200
Tremarctos ornatus	Spectacled bear	Carnivora	13	1	-10.4	53,6	3583	3583
Xerus rutilus	Unstriped ground squirrel	Rodentia	12	2	-13.01	53,6	3962	3962
Cavia tschudii	Montane guinea pig	Rodentia	12	4	ND	53,2	3061	3061
Miniopterus natalensis	Natal long-fingered bat	Chiroptera	16	4	ND	50,7	1125	1125
Phacochoerus africanus	Common warthog	Artiodactyla	13	2	-13.67	48,6	3101	3101
Petromus typicus	Dassie rat	Rodentia	11	1	ND	48,2	3148	3148
Rousettus madagascariensis	Madagascan rousette	Chiroptera	11	1	-18.77	48,2	3973	3973
Sciurus stramineus	Guayaquil squirrel	Rodentia	11	3	-7.89	48,2	1091	1091
Indri indri	Indri	Primates	11	4	-10.04	47,9	3227	3227
Pseudocheirus peregrinus o.	Western ringtail	Diprotodontia	17	3	ND	46,4	3959	3959
Typhlomys cinereus	Chinese pygmy dormouse	Rodentia	8	1	-13.72	46,4	4478	4478
Aotus trivirgatus	Three-striped night monkey	Primates	9	3	-5.4	46,1	1385	1385
Bos grunniens	Domestic yak	Artiodactyla	13	4	-4.17	45,2	1748	1748
Kobus ellipsiprymnus	Waterbuck	Artiodactyla	11	1	-12.69	44,6	1351	1351
Sciurus carolinensis	Eastern gray squirrel	Rodentia	12	1	-14.31	44,6	3708	3708
Suncus etruscus	Etruscan shrew	Soricomorpha	14	2	-3.98	44,6	4534	4534
Tragelaphus imberbis	Lesser kudu	Artiodactyla	10	2	-12.32	44,6	1380	1380
Ursus arctos	Brown bear	Carnivora	13	1	-10.67	44,6	0927	0927
Microtus oregoni	Creeping vole	Rodentia	2	1	-4.39	42,9	3388	3388
Rhabdomys dilectus	Mesic four-striped grass rat	Rodentia	5	2	-4.89	40,4	3800	3800
Catagonus wagneri	Chacoan peccary	Artiodactyla	13	6	-17.92	40	2022	2022
Hylomyscus alleni	Allen's wood mouse	Rodentia	5	1	-10.63	40	3803	3803
Dipodomys spectabilis	Banner-tailed kangaroo rat	Rodentia	12	4	-8.72	39,8	3688	3688
Herpestes javanicus	Javan mongoose	Carnivora	11	3	-6.95	39,6	1345	1345
Moschus chrysogaster	Alpine musk deer	Artiodactyla	10	2	-12.34	39,3	0920	0920
Manis javanica	Pangolin	Pholidota	8	5	ND	38,6	0942	0942
Tragelaphus scriptus	Harnessed bushbuck	Artiodactyla	11	2	-15.56	36,8	3060	3060
Lophiomys imhausi	Maned rat	Rodentia	4	1	-2.47	36,6	3608	3608
Tamandua tetradactyla	Southern tamandua	Pilosa	15	2	-10.89	36,6	3137	3137
Hylomys suillus	Short-tailed gymnure	Erinaceomorpha	12	2	-10.37	36,1	1344	1344
Hipposideros galeritus	Cantor's roundleaf bat	Chiroptera	6	1	-8.51	35,9	3223	3223
Arctictis binturong	Binturong	Carnivora	11	1	-5.22	35,7	0925	0925
Mandrillus leucophaeus	Drill	Primates	10	3	-10.7	35,7	2078	2078
Octomys mimax	Mountain viscacha rat	Rodentia	11	1	ND	33,9	3172	3172
Potos flavus	Kinkajou	Carnivora	12	3	-6.27	33,9	3094	3094
Propithecus coquereli	Coquerel's sifaka	Primates	11	2	-3.69	33,9	1127	1127
Tragelaphus strepsiceros	Greater kudu	Artiodactyla	12	1	-14.67	33,9	1482	1482
Trichosurus vulpecula	Common brushtail possum	Diprotodontia	18	3	ND	33,9	1335	1335
Babyrousa celebensis	North Sulawesi babirusa	Artiodactyla	12	1	-12.04	33	3940	3940
Daubentonia madagascariensis	Aye-aye	Primates	11	3	ND	33	3173	3173
Arctocephalus gazella	Antarctic fur seal	Carnivora	11	2	-11.5	32,5	3248	3248
Peromyscus polionotus s.	Oldfield mouse	Rodentia	1	2	1.23	32,5	0941	0941
Apodemus fulvipectus	Steppe field mouse	Rodentia	5	1	-11.57	30,4	1395	1395
Homo sapiens	Human	Primates	12	2	-11.6	30,4	0178	0178
Lagothrix lagotricha	Brown woolly monkey	Primates	10	4	-7.89	30	1389	1389
Gulo gulo	Wolverine	Carnivora	12	3	-4.08	29,8	0123	0123
Ctenomys sociabilis	Social tuco-tuco	Rodentia	14	2	ND	28,6	3078	3078
Phalanger gymnotis	Ground cuscus	Diprotodontia	16	5	ND	28,6	3956	3956
Mirza coquereli	Coquerel's giant mouse lemur	Primates	6	3	4.23	27,9	3273	3273

BINOMIAL NAME	COMMON NAME	ORDER	AA No.[a]	Conf No.[b]	Stability [c]	Score [d]	PDB [e]	PrPdex [f]
Arvicola amphibius	European water vole	Rodentia	1	1	-0.51	27,7	3080	3080
Dinomys branickii	Pacarana	Rodentia	12	1	ND	26,8	3082	3082
Jaculus jaculus	Lesser Egyptian jerboa	Rodentia	13	2	-14.88	26,8	0936	0936
Macaca nemestrina	Pigtail macaque	Primates	9	1	-6.33	26,8	1970	1970
Peromyscus attwateri	Texas mouse	Rodentia	2	1	1.56	26,8	3188	3188
Macroscelides proboscideus	Round-eared elephant shrew	Macroscelidea	15	4	-9.98	26,4	1343	1343
Atelerix albiventris	Four-toed hedgehog	Erinaceomorpha	16	2	ND	25,4	4029	4029
Sylvicapra grimmia	Common duiker	Artiodactyla	10	3	-12.74	25	3204	3204
Monachus schauinslandi	Hawaiian monk seal	Carnivora	14	2	-10.71	24,6	1112	1112
Mungos mungo	Banded mongoose	Carnivora	13	2	-9.1	23,6	3278	3278
Canis mesomelas	Black-backed jackal	Carnivora	12	2	-8.24	23	3927	3927
Gracilinanus agilis	Agile gracile opossum	Didelphimorphia	17	3	ND	22,9	3086	3086
Talpa occidentalis	Spanish mole	Soricomorpha	11	4	-3.35	22,9	3143	3143
Budorcas taxicolor	Takin	Artiodactyla	11	1	-12.4	22,3	1348	1348
Manis pentadactyla	Chinese pangolin	Pholidota	8	4	ND	22,1	3092	3092
Ateles geoffroyi	Geoffroy's spider monkey	Primates	8	1	-6.77	21,4	1417	1417
Eonycteris spelaea	Cave nectar bat	Chiroptera	11	1	-13.08	21,4	3207	3207
Molossus molossus	Velvety free-tailed bat	Chiroptera	14	2	-7.17	21,1	2386	2386
Pan troglodytes	Chimpanzee	Primates	12	2	-9.88	20,7	1134	1134
Antilocapra americana	Pronghorn	Artiodactyla	10	1	-12.73	19,6	0945	0945
Sminthopsis crassicaudata	Fat-tailed dunnart	Dasyuromorphia	21	3	ND	19,6	3850	3850
Rousettus leschenaultii	Leschenault's rousette	Chiroptera	12	2	-20.82	19,3	3157	3157
Chinchilla lanigera	Long-tailed chinchilla	Rodentia	13	1	ND	18,8	1136	1136
Saguinus bicolor	Pied tamarin	Primates	7	2	6.16	18,8	1456	1456
Talpa europaea	European mole	Soricomorpha	12	1	-2.87	18,8	0974	0974
Spilogale gracilis	Western spotted skunk	Carnivora	12	1	-1.74	18,2	3161	3161
Ochotona princeps	American pika	Lagomorpha	11	1	-12.67	17,9	1140	1140
Sciurus vulgaris	Red squirrel	Rodentia	12	1	-15.13	17,9	0949	0949
Sorex araneus	Common shrew	Soricomorpha	16	1	-14.08	17,9	0976	0976
Manis tricuspis	Tree pangolin	Pholidota	8	2	ND	17	3089	3089
Muscardinus avellanarius	Hazel dormouse	Rodentia	11	2	-5.66	17	3285	3285
Tarsius syrichta	Philippine tarsier	Primates	8	1	-9.49	17	0943	0943
Galemys pyrenaicus	Pyrenean desman	Soricomorpha	10	3	-9.42	16,6	2562	2562
Bubalus bubalis	Water buffalo	Artiodactyla	11	3	-12.88	16,1	1198	1198
Ondatra zibethicus	Muskrat	Rodentia	1	1	0.61	16,1	3191	3191
Rattus argentiventer	Ricefield rat	Rodentia	5	1	-12,39	16,1	1341	1341
Equus caballus przewalskii	Przewalski's horse	Perissodactyla	11	1	-8.75	14,3	0413	0413
Gorilla gorilla	Western gorilla	Primates	11	1	-5.87	14,3	1148	1148
Tapirus indicus	Malayan tapir	Perissodactyla	12	1	-9.07	14,3	3134	3134
Tapirus terrestris	South American tapir	Perissodactyla	15	2	-7.74	14,3	3135	3135
Hoolock leuconedys	Eastern hoolock gibbon	Primates	12	3	-11.24	13,9	4530	4530
Cryptomys damarensis	Damaraland mole-rat	Rodentia	13	2	ND	13	1130	1130
Felis silvestris catus	Cat	Carnivora	11	2	-8.92	12,9	0173	0173
Pseudochirops corinnae	Plush-coated ringtail possum	Diprotodontia	19	2	ND	12,9	3960	3960
Bos taurus	Cow	Artiodactyla	11	2	-10.33	12,5	0201	0201
Lagorchestes hirsutus	Rufous hare-wallaby	Diprotodontia	19	1	ND	12,5	3934	3934
Litocranius walleri	Gerenuk	Artiodactyla	9	2	-13.08	12,5	3257	3257
Sylvilagus bachmani	Brush rabbit	Lagomorpha	14	2	-15.86	12,5	3131	3131
Vicugna vicugna	Vicuna	Artiodactyla	12	2	-7.28	12	2088	2088
Pongo pygmaeus	Bornean orangutan	Primates	9	1	-10.55	11,6	0926	0926
Graphiurus murinus	Woodland dormouse	Rodentia	18	1	-7.47	11,4	3218	3218
Phoca vitulina	Common seal	Carnivora	12	3	-9.87	11,1	0929	0929
Cercartetus concinnus	Western pygmy possum	Diprotodontia	15	1	ND	10,7	4104	4104

BINOMIAL NAME	COMMON NAME	ORDER	AA No.[a]	Conf No.[b]	Stability[c]	Score[d]	PDB[e]	PrPdex[f]
Craseonycteris thonglongyai	Kitti's hog-nosed bat	Chiroptera	8	2	-6.77	10,7	3066	3066
Cryptomys darlingi	Mashona mole-rat	Rodentia	14	3	ND	10,7	4004	4004
Nycticebus coucang	Sunda slow loris	Primates	11	2	-1.87	10,7	3294	3294
Dasyprocta punctata	Central American agouti	Rodentia	11	4	ND	10,4	3158	3158
Bettongia penicillata ogilbyi	Woylie	Diprotodontia	17	1	ND	9,8	3976	3976
Cryptoprocta ferox	Fossa	Carnivora	12	1	-8.09	9,8	3075	3075
Camelus bactrianus ferus	Bactrian camel	Artiodactyla	12	1	-3.78	8,9	0544	0544
Cercocebus atys	Sooty mangabey	Primates	10	1	-9.39	8,9	2057	2057
Dasyurus hallucatus	Northern quoll	Dasyuromorphia	23	1	ND	8,9	3849	3849
Hexaprotodon liberiensis	Pygmy hippopotamus	Artiodactyla	12	1	ND	8,9	0946	0946
Mephitis mephitis	Skunk	Carnivora	13	1	-4.03	8,9	0538	0538
Myotis septentrionalis	Northern long-eared bat	Chiroptera	15	1	-10.06	8,9	3970	3970
Solenodon paradoxus woodi	Hispaniolan solenodon	Soricomorpha	16	1	-8.12	8,9	3141	3141
Trichechus manatus	West Indian manatee	Sirenia	12	1	ND	8,9	0959	0959
Vombatus ursinus	Common wombat	Diprotodontia	16	1	ND	8,9	1100	1100
Colobus angolensis palliatus	Angola colobus	Primates	9	2	-1.54	8,4	1200	1200
Odobenus rosmarus	Walrus	Carnivora	11	2	-12.13	8	0547	0547
Zapus hudsonius	Meadow jumping mouse	Rodentia	13	2	-9.25	8	3323	3323
Myotis brandtii	Brandt's bat	Chiroptera	16	2	-12.24	7,7	1102	1102
Miopithecus talapoin	Angolan talapoin	Primates	10	2	-10.99	7,5	4508	4508
Equus caballus ferus	Horse	Perissodactyla	12	1	-4.71	7,1	0115	0115
Myotis davidii	David's myotis	Chiroptera	14	1	-13.43	7,1	1132	1132
Saguinus midas	Golden-handed tamarin	Primates	7	2	-4.49	7,1	4093	4093
Tadarida brasiliensis	Mexican free-tailed bat	Chiroptera	15	1	ND	7,1	3162	3162
Lepus europaeus	Brown hare	Lagomorpha	13	1	-17.76	5,9	0533	0533
Cricetomys gambianus	Gambian pouched rat	Rodentia	8	1	-4.31	5,5	3067	3067
Pipistrellus kuhlii	Kuhl's pipistrelle	Chiroptera	16	1	-3.52	5	2535	2535
Loris tardigradus	Red slender loris	Primates	10	1	10.2	4,8	4527	4527
Cebus capucinus	Panamanian white-faced capuchin	Primates	9	2	-2.68	3,9	1084	1084
Connochaetes taurinus	Blue wildebeest	Artiodactyla	11	1	-8.49	3,6	1414	1414
Dromiciops gliroides	Colocolo opossum	Microbiotheria	14	1	ND	3,6	3732	3732
Equus asinus	Asinus	Perissodactyla	12	1	-2.76	3,6	1070	1070
Lasiurus cinereus	Hoary bat	Chiroptera	16	1	-4.45	3,6	3046	3046
Leopardus geoffroyi	Geoffroy's cat	Carnivora	11	1	-7.1	3,6	4011	4011
Monodelphis domestica	Opossum	Didelphimorphia	17	1	ND	3,6	0398	0398
Petaurus breviceps	Sugar glider	Diprotodontia	17	1	-22,28	3,6	4816	4816
Procapra gutturosa	Mongolian gazelle	Artiodactyla	14	1	-9.58	3,6	1143	1143
Thylacinus cynocephalus	Thylacine	Dasyuromorphia	18	1	ND	3,6	4339	4339
Tympanoctomys barrerae	Plains viscacha rat	Rodentia	11	1	ND	3,6	3322	3322
Condylura cristata	Star-nosed mole	Soricomorpha	12	2	-0.07	3,2	1138	1138
Chrysochloris asiatica	Cape golden mole	Afrosoricida	15	1	ND	2,9	1128	1128
Ceratotherium simum simum	Southern white rhinoceros	Perissodactyla	13	1	-11.01	2	0955	0955
Galeopterus variegatus	Sunda flying lemur	Dermoptera	12	1	-8.07	1,8	0957	0957
Otolemur garnettii	Northern greater galago	Primates	9	1	-2.15	1,8	1105	1105
Giraffa camelopardalis r.	Reticulata giraffe	Artiodactyla	12	1	-14.52	1,6	0951	0951
Dasyurus viverrinus	Eastern quoll	Dasyuromorphia	22	1	ND	1,4	4008	4008
Macrotus californicus	California leaf-nosed bat	Chiroptera	17	3	ND	1,4	1151	1151
Helogale parvula	Common dwarf mongoose	Carnivora	12	1	-11.52	0,9	3220	3220
Hippopotamus amphibius	Hippopotamus	Artiodactyla	12	1	ND	0,9	1359	1359
Leontopithecus rosalia	Golden lion tamarin	Primates	6	1	-4.23	0,9	1437	1437
Leopardus wiedii	Margay	Carnivora	12	1	ND	0,9	1443	1443
Mandrillus sphinx	Mandrill	Primates	9	1	-6.05	0,9	1208	1208
Oryctolagus cuniculus	Rabbit	Lagomorpha	12	1	-13.56	0,9	0135	0135

BINOMIAL NAME	COMMON NAME	ORDER	AA No.[a]	Conf No.[b]	Stability [c]	Score [d]	PDB [e]	PrPdex [f]
Perognathus longimembris p.	Little pocket mouse	Rodentia	14	1	ND	0,9	3187	3187
Peromyscus crinitus	Canyon mouse	Rodentia	3	1	-1.39	0,9	3975	3975
Prolemur simus	Greater bamboo lemur	Primates	10	1	-6.96	0,9	2028	2028
Rhinolophus sinicus	Chinese rufous horseshoe bat	Chiroptera	15	1	-3.92	0,9	3151	3151
Saimiri boliviensis	Black-capped squirrel monkey	Primates	11	1	-5.19	0,9	1975	1975
Spermophilus dauricus	Daurian ground squirrel	Rodentia	19	1	ND	0,9	2025	2025
Chiropotes satanas	Black bearded saki	Primates	8	1	-3.52	0,7	1431	1431
Dasypus novemcinctus	Nine-banded armadillo	Cingulata	16	1	-20.08	0,4	0968	0968
Desmodus rotundus	Common vampire bat	Chiroptera	15	1	ND	0,4	1472	1472
Eptesicus fuscus	Big brown bat	Chiroptera	17	1	-0.01	0,4	1139	1139
Lynx pardinus	Iberian lynx	Carnivora	11	1	-8.12	0,4	0977	0977
Spermophilus beecheyi	California ground squirrel	Rodentia	15	1	-11.31	0,4	4486	4486
Anoura caudifer	Tailed tailless bat	Chiroptera	15	0	ND	0	3243	3243
Antechinus flavipes	Yellow-footed antechinus	Dasyuromorphia	18	0	ND	0	3128	3128
Antechinus stuartii	Brown antechinus	Dasyuromorphia	19	0	ND	0	3048	3048
Antrozous pallidus	Pallid bat	Chiroptera	17	0	-1.85	0	3246	3246
Aotus lemurinus	Gray-bellied night monkey	Primates	9	0	1.07	0	1386	1386
Artibeus jamaicensis	Jamaican fruit bat	Chiroptera	16	0	ND	0	3249	3249
Ateles paniscus	Red-faced spider monkey	Primates	9	0	-5.68	0	1415	1415
Balaenoptera physalus	Whale	Cetacea	14	0	-16.96	0	0960	0960
Brachyteles arachnoides	Southern muriqui	Primates	7	0	-4.43	0	1337	1337
Bradypus variegatus	Brown-throated sloth	Pilosa	16	0	-11.57	0	3057	3057
Bubalus depressicornis	Anoa	Artiodactyla	12	0	-12.62	0	1363	1363
Cabassous unicinctus	Southern naked-tailed armadillo	Cingulata	22	0	-14.75	0	4842	4842
Capromys pilorides	Desmarest's hutia	Rodentia	11	0	ND	0	3213	3213
Caracal caracal	Caracal	Carnivora	13	0	-6.41	0	3054	3054
Carollia perspicillata	Seba's short-tailed bat	Chiroptera	15	0	ND	0	3058	3058
Cavia aperea	Brazilian guinea pig	Rodentia	13	0	ND	0	1470	1470
Cavia porcellus	Guinea pig	Rodentia	13	0	-6.62	0	0815	0815
Chaetophractus vellerosus	Screaming hairy armadillo	Cingulata	17	0	-17.17	0	3238	3238
Cheirogaleus medius	Fat-tailed dwarf lemur	Primates	7	0	10.05	0	3059	3059
Choloepus didactylus	Linnaeus's two-toed sloth	Pilosa	18	0	-14.14	0	3071	3071
Coendou prehensilis	Brazilian porcupine	Rodentia	15	0	ND	0	3785	3785
Corynorhinus townsendii	Townsend's big-eared bat	Chiroptera	17	0	-17.27	0	4684	4684
Crocidura russula	Greater white-toothed shrew	Soricomorpha	13	0	-18.73	0	4606	4606
Crocuta crocuta	Spotted hyena	Carnivora	15	0	-9.33	0	0531	0531
Cyclopes didactylus	Silky anteater	Pilosa	16	0	ND	0	0923	0923
Delphinapterus leucas	Beluga whale	Cetacea	12	0	-17.39	0	0924	0924
Dendrolagus matschiei	Matschie's tree-kangaroo	Diprotodontia	19	0	-23.84	0	4627	4627
Didelphis virginiana	Virginia opossum	Didelphimorphia	18	0	ND	0	3918	3918
Dolichotis patagonum	Patagonian mara	Rodentia	13	0	ND	0	1087	1087
Dugong dugon	Dugong	Sirenia	12	0	ND	0	3138	3138
Echinops telfairi	Lesser hedgehog tenrec	Afrosoricida	20	0	ND	0	1082	1082
Elephantulus edwardii	Cape elephant shrew	Macroscelidea	15	0	ND	0	0972	0972
Erethizon dorsata	North American porcupine	Rodentia	17	0	ND	0	3085	3085
Erignathus barbatus	Bearded seal	Carnivora	12	0	-8.49	0	3919	3919
Erinaceus europaeus	European hedgehog	Erinaceomorpha	16	0	ND	0,9	0931	0931
Galago moholi	Mohol bushbaby	Primates	10	0	-7.11	0	4523	4523
Heterocephalus glaber	Naked mole-rat	Rodentia	12	0	ND	0	0978	0978
Hydrochoerus hydrochaeris	Capybara	Rodentia	14	0	ND	0	1435	1435
Hyperoodon ampullatus	Northern bottlenose whale	Cetacea	15	0	-20.25	0	4556	4556
Hystrix brachyura	Malayan porcupine	Rodentia	13	0	ND	0	3056	3056
Ia io	Great evening bat	Chiroptera	15	0	-13.71	0	4617	4617

BINOMIAL NAME	COMMON NAME	ORDER	AA No.[a]	Conf No.[b]	Stability[c]	Score[d]	PDB[e]	PrPdex[f]
Inia geoffrensis	Amazon river dolphin	Cetacea	12	0	-17.25	0	3228	3228
Lagenorhynchus obliquidens	Pacific white-sided dolphin	Cetacea	13	0	-30.33	0	1416	1416
Leopardus tigrinus	Oncilla	Carnivora	11	0	-14.52	0	4815	4815
Lipotes vexillifer	Baiji	Cetacea	13	0	-10.64	0	0922	0922
Loxodonta africana	Elephant	Proboscidea	16	0	ND	0	0930	0930
Macropus eugenii	Wallaby	Diprotodontia	19	0	ND	0	3333	3333
Macropus giganteus	Eastern grey kangaroo	Diprotodontia	20	0	ND	0	3938	3938
Macropus rufus	Red kangaroo	Diprotodontia	22	0	ND	0	3775	3775
Marmota flaviventris	Yellow-bellied marmot	Rodentia	16	0	-10.36	0	1099	1099
Marmota marmota	Alpine marmot	Rodentia	17	0	-8.03	0	2667	2667
Megaderma lyra	Greater false vampire bat	Chiroptera	8	0	ND	0	3258	3258
Mesocricetus auratus	Syrian hamster	Rodentia	6	0	-0.58	0	0133	0133
Mesoplodon bidens	Sowerby's beaked whale	Cetacea	13	0	-15.45	0	3262	3262
Mesoplodon stejnegeri	Stejneger's beaked whale	Cetacea	13	0	-16.57	0	3937	3937
Microcebus murinus	Gray mouse lemur	Primates	8	0	1.62	0	0933	0933
Microcebus ravelobensis	Golden-brown mouse lemur	Primates	9	0	-0.56	0	3265	3265
Microgale talazaci	Talazac's shrew tenrec	Afrosoricida	24	0	ND	0	3087	3087
Micronycteris hirsuta	Hairy big-eared bat	Chiroptera	15	0	ND	0	3267	3267
Miniopterus schreibersii	Common bent-wing bat	Chiroptera	13	0	ND	0	3271	3271
Mormoops blainvillei	Antillean ghost-faced bat	Chiroptera	15	0	-14.83	0	3049	3049
Murina aurata feae	Little tube-nosed bat	Chiroptera	16	0	-7.85	0	3279	3279
Myocastor coypus	Coypu	Rodentia	10	0	ND	0	3171	3171
Myotis ricketti	Rickett's big-footed bat	Chiroptera	15	0	-12.32	0	3338	3338
Myotis yumanensis	Yuma myotis	Chiroptera	16	0	-17.57	0	4819	4819
Myrmecobius fasciatus	Numbat	Dasyuromorphia	19	0	ND	0	4079	4079
Myrmecophaga tridactyla	Giant anteater	Pilosa	16	0	-15.61	0	3136	3136
Neophocaena phocaenoides a.	Narrow-ridged finless porpoise	Cetacea	12	0	-24.72	0	1126	1126
Noctilio leporinus	Greater bulldog bat	Chiroptera	14	0	ND	0	3293	3293
Notoryctes typhlops	Southern marsupial mole	Notoryctemorphia	18	0	-15.2	0	4081	4081
Nycticebus pygmaeus	Pygmy slow loris	Primates	14	0	-6.58	0	4835	4835
Nycticeius humeralis	Evening bat	Chiroptera	16	0	-7.38	0	3295	3295
Ornithorhynchus anatinus	Platypus	Monotremata	26	0	ND	0	0944	0944
Orycteropus afer	Aardvark	Tubulidentata	15	0	ND	0	4930	4930
Petrogale xanthopus	Yellow-footed rock-wallaby	Diprotodontia	20	0	ND	0	3762	3762
Phascogale tapoatafa	Brush-tailed phascogale	Dasyuromorphia	21	0	ND	0	4470	4470
Phascolarctos cinereus	Koala	Diprotodontia	19	0	ND	0	0935	0935
Phyllostomus discolor	Pale spear-nosed bat	Chiroptera	15	0	ND	0	3765	3765
Physeter catodon	Sperm whale	Cetacea	12	0	-16.05	0	0956	0956
Pithecia irrorata	Rio Tapajós saki	Primates	9	0	-6.48	0	1411	1411
Platanista gangetica	South Asian river dolphin	Cetacea	13	0	-14.99	0	3168	3168
Plecotus auritus	Lump-nosed bat	Chiroptera	17	0	-3.01	0	0114	0114
Pontoporia blainvillei	La Plata dolphin	Cetacea	13	0	-13.69	0	3311	3311
Potorous gilbertii	Gilbert's potoroo	Diprotodontia	18	0	ND	0	3958	3958
Procavia capensis	Rock hyrax	Hyracoidea	14	0	ND	0	0952	0952
Pteronotus parnellii	Parnell's mustached bat	Chiroptera	17	0	ND	0	3097	3097
Pusa hispida saimensis	Ringed seal	Carnivora	13	0	-14.83	0	4786	4786
Redunca redunca	Bohor reedbuck	Artiodactyla	10	0	-14.8	0	3319	3319
Rhinolophus ferrumequinum	Greater horseshoe bat	Chiroptera	13	0	-6.69	0	2030	2030
Saimiri sciureus	Squirrel monkey	Primates	10	0	-5.63	0	0362	0362
Sarcophilus harrisii	Tasmanian devil	Dasyuromorphia	22	0	ND	0	0970	0970
Scalopus aquaticus	Eastern mole	Soricomorpha	15	0	-8.06	0	3142	3142
Setonix brachyurus	Quokka	Diprotodontia	20	0	ND	0	3862	3862
Sorex cinereus	Cinereous shrew	Soricomorpha	16	0	-4.11	0	1352	1352

BINOMIAL NAME	COMMON NAME	ORDER	AA No.[a]	Conf No.[b]	Stability [c]	Score [d]	PDB [e]	PrPdex [f]
Sorex palustris	American water shrew	Soricomorpha	16	0	-9.15	0	4817	4817
Spermophilus parryii	Arctic ground squirrel	Rodentia	18	0	ND	0	1119	1119
Spermophilus tridecemlineatus	Thirteen-lined ground squirrel	Rodentia	17	0	ND	0	1106	1106
Sturnira ludovici hondurensis	Honduran yellow-shouldered bat	Chiroptera	15	0	ND	0	3126	3126
Sylvilagus transitionalis	New England cottontail	Lagomorpha	13	0	-18.5	0	4742	4742
Tachyglossus aculeatus	Short-beaked echidna	Monotremata	24	0	ND	0	3077	3077
Tamias sibiricus	Siberian chipmunk	Rodentia	15	0	-11.42	0	4618	4618
Tenrec ecaudatus	Tailless tenrec	Afrosoricida	17	0	ND	0	1347	1347
Tolypeutes matacus	Southern three-banded armadillo	Cingulata	21	0	-8.09	0	3163	3163
Tonatia saurophila	Stripe-headed round-eared bat	Chiroptera	15	0	ND	0	3164	3164
Trachops cirrhosus	Fringe-lipped bat	Chiroptera	13	0	-13.99	0	4512	4512
Tragelaphus buxtoni	Mountain nyala	Artiodactyla	15	0	-13.61	0	3241	3241
Tursiops truncatus	Common bottlenose dolphin	Cetacea	13	0	-19.79	0	0409	0409
Uropsilus gracilis	Gracile shrew mole	Soricomorpha	9	0	-5.6	0	3144	3144
Ziphius cavirostris	Ziphius	Cetacea	14	0	-21.45	0	0961	0961

This table provides a comprehensive list of the 382 species analyzed in the study, along with their corresponding misfolding scores. The species are arranged in descending order of misfolding proneness scores, ranging from 100 to 0. In cases where species share the same score, they are further organized alphabetically based on their binomial names.
Readers can access detailed information about each protein by clicking on certain data (PDB and PrPdex). These codes link to various valuable pieces of information collected in this study, all integrated into the PrPdex database (https://prpdex.com).

[a] Number of amino acids differing from the bank vole PrP

[b] Number of distinct biochemical conformers observed.

[c] Predicted stability of the globular counterpart

[d] Misfolding proneness score

[e] PDB code to access to a specific URL displaying the PDB details within the AlphaFold predicted structure.

[f] PrPdex code to access to a specific URL containing a comprehensive file that encompasses all data obtained in this study for each protein.

Supplementary table 3. List of 29 mammal species whose prion proteins' infectivity is being tested either *in vitro* or *in vivo.*

ORDER	COMMON NAME	BINOMIAL NAME	*IN VITRO* PROPAGATION		*IN VIVO* INFECTIVITY	
			In TgVole 1X	In homologous substrate	In TgVole 1X	In homologous substrate
Artiodactyla	Cow	*Bos taurus*	Propagate	Ongoing	Infectious	Not tested
	Elk	*Cervus elaphus canadensis*	Propagate	Propagate	Infectious	Ongoing (TgElk)
	Mule deer	*Odocoileus hemionus*	Propagate	Propagate	Infectious	Ongoing (TgMule Deer)
	Sheep	*Ovis aries*	Propagate	Propagate	Infectious	Infectious (Tg338)
	Pig	*Sus scrofa*	Propagate	Ongoing	Infectious	Not tested
Carnivora	Dog	*Canis lupus familiaris*	Propagate	Ongoing	Infectious	Ongoing (TgDog)
	Cat	*Felis silvestris catus*	Propagate	Ongoing	Ongoing	Not tested
	Skunk	*Mephitis mephitis*	Ongoing	Ongoing	Ongoing	Not tested
	Ferret	*Mustela putorius*	Propagate	Ongoing	Ongoing	Not tested
	American mink	*Neovison vison*	Propagate	Ongoing	Infectious	Not tested
Chiroptera	Egyptian fruit bat	*Rousettus aegyptiacus*	Propagate	Ongoing	Infectious	Not tested
Dasyuromorphia	Thylacine	*Thylacinus cynocephalus*	Ongoing	Not tested	Ongoing	Not tested
Dermoptera	Sunda flying lemur	*Galeopterus variegatus*	No propagation	Not tested	Ongoing	Not tested
Didelphimorphia	Agile gracile opossum	*Gracilinanus agilis*	Ongoing	Not tested	Ongoing	Not tested
	Opossum	*Monodelphis domestica*	No propagation	Not tested	Non infectious	Not tested
Diprotodontia	Western pygmy possum	*Cercartetus concinnus*	Propagate	Not tested	Infectious	Not tested
Lagomorpha	Rabbit	*Oryctolagus cuniculus*	Propagate	Ongoing	Infectious	Ongoing
Microbiotheria	Colocolo opossum	*Dromiciops gliroides*	No propagation	Not tested	Ongoing	Not tested
Perissodactyla	Horse	*Equus caballus ferus*	Propagate	Ongoing	Ongoing	Not tested
Pholidota	Pangolin	*Manis javanica*	Ongoing	Not tested	Ongoing	Not tested
Primates	Human	*Homo sapiens*	Ongoing	Ongoing	Infectious	Ongoing
	Pigtail macaque	*Macaca nemestrina*	No propagation	Not tested	Ongoing	Not tested
Rodentia	Montane guinea pig	*Cavia tschudii*	Propagate	Ongoing (in guinea pig)	Ongoing	Not tested
	Long-tailed chinchilla	*Chinchilla lanigera*	Propagate	Ongoing	Ongoing	Not tested
	Mongolian gerbil	*Meriones unguiculatus*	Propagate	Propagate	Infectious	Ongoing (mongolian gerbil)
	Bank vole	*Myodes glareolus*	Propagate	Propagate (in mouse)	Infectious	Infectious (mouse)
	Russian hamster	*Phodopus sungorus*	Propagate	Propagate	Infectious	Not tested
	Brown rat	*Rattus norvegicus*	Propagate	Propagate	Infectious	Not tested
	Unstriped ground squirrel	*Xerus rutilus*	Propagate	Not tested	Ongoing	Not tested
