# Supplementary material for: A Protein Misfolding Shaking Amplification-based method for the spontaneous generation of hundreds of bona fide prions
Source: Nat Commun. 2024 Mar 8;15:2112. doi: 10.1038/s41467-024-46360-2 (PMC10923866; doi:10.1038/s41467-024-46360-2)
Supplement: Supplementary file 2 — Reporting Summary [file 41467_2024_46360_MOESM2_ESM.pdf]

nature portfolio

Corresponding author(s): Joaquín Castilla

Last updated by author(s): Jan 22, 2024

Reporting Summary

Nature Portfolio wishes to improve the reproducibility of the work that we publish. This form provides structure for consistency and transparency in reporting. For further information on Nature Portfolio policies, see our Editorial Policies and the Editorial Policy Checklist.

Statistics

For all statistical analyses, confirm that the following items are present in the figure legend, table legend, main text, or Methods section.

n/a	Confirmed	
☐	☒	The exact sample size (n) for each experimental group/condition, given as a discrete number and unit of measurement
☐	☒	A statement on whether measurements were taken from distinct samples or whether the same sample was measured repeatedly
☒	☐	The statistical test(s) used AND whether they are one- or two-sided<br>*Only common tests should be described solely by name; describe more complex techniques in the Methods section.*
☒	☐	A description of all covariates tested
☐	☒	A description of any assumptions or corrections, such as tests of normality and adjustment for multiple comparisons
☐	☒	A full description of the statistical parameters including central tendency (e.g. means) or other basic estimates (e.g. regression coefficient) AND variation (e.g. standard deviation) or associated estimates of uncertainty (e.g. confidence intervals)
☒	☐	For null hypothesis testing, the test statistic (e.g. F, t, r) with confidence intervals, effect sizes, degrees of freedom and P value noted<br>*Give P values as exact values whenever suitable.*
☒	☐	For Bayesian analysis, information on the choice of priors and Markov chain Monte Carlo settings
☒	☐	For hierarchical and complex designs, identification of the appropriate level for tests and full reporting of outcomes
☒	☐	Estimates of effect sizes (e.g. Cohen's d, Pearson's r), indicating how they were calculated

Our web collection on statistics for biologists contains articles on many of the points above.

Software and code

Policy information about availability of computer code

Data collection	Most of the study results and data were collected and organised in the Filemaker Pro (version 12) database designing software, a commercially available software.
Data analysis	The study did not require for specific software for analysis. Excel (Office 365 version) was used for most of the calculations. Additionally, for image analysis AlphaView software (version 3.4.0) from ProteinSimple was used for image analysis, AlphaFold 2.3.2 for the generation of three dimensional protein structural models and PyMOL 2.4.0 to generate final figures.

For manuscripts utilizing custom algorithms or software that are central to the research but not yet described in published literature, software must be made available to editors and reviewers. We strongly encourage code deposition in a community repository (e.g. GitHub). See the Nature Portfolio guidelines for submitting code & software for further information.

Data

Policy information about availability of data

All manuscripts must include a data availability statement. This statement should provide the following information, where applicable:

- Accession codes, unique identifiers, or web links for publicly available datasets
- A description of any restrictions on data availability
- For clinical datasets or third party data, please ensure that the statement adheres to our policy

All the source data generated and analysed during this study and published in this article (and its supplementary information files), are publicly available in the Zenodo repository, as a Dataset, with DOI: 10.5281/zenodo.10579518. Additionally, the datasets generated during and/or analysed during the current study are

available in the prpdex.com webpage, https://prpdex.com/. The Data Availability Statement includes a detailed list of the information available in Zenodo and the link to easily access this information.

Research involving human participants, their data, or biological material

Policy information about studies with human participants or human data. See also policy information about sex, gender (identity/presentation), and sexual orientation and race, ethnicity and racism.

Reporting on sex and gender	N/A
Reporting on race, ethnicity, or other socially relevant groupings	N/A
Population characteristics	N/A
Recruitment	N/A
Ethics oversight	N/A

Note that full information on the approval of the study protocol must also be provided in the manuscript.

Field-specific reporting

Please select the one below that is the best fit for your research. If you are not sure, read the appropriate sections before making your selection.

☒ Life sciences ☐ Behavioural & social sciences ☐ Ecological, evolutionary & environmental sciences

For a reference copy of the document with all sections, see nature.com/documents/nr-reporting-summary-flat.pdf

Life sciences study design

All studies must disclose on these points even when the disclosure is negative.

Sample size	For the misfolding score calculation, we worked with all the prion proteins from mammals known so far. 725 prion protein variant sequences from 725 distinct mammal species were used, without excluding any. Thus all available data was used in the study and no sample size calculation was required. Regarding animal experiments, no formal sample size calculation was performed given the exploratory nature of the study and the lack of prior data regarding the infectivity of these recombinant prions. Therefore, the number of animals (n=5-7) was decided based on previous one-to-one animal transmission studies, in which for pilot studies, groups of 5 animals have been deemed sufficient, including up to 7 animals per group to prevent losses due to intercurrent diseases.
Data exclusions	No data were excluded.
Replication	All the experiments were performed using 4+4 replicates. The negative results were repeated twice using the same number of replicates. As included in Statistics and Reproducibility section, from 130 repeated assays, variations were detected in the 16 % upon repetition. However, considering that the variation was minimal in most of the repetitions (scores varying from 0 to 3 or less), greater variations were only found in 5 % of repeated assays.
Randomization	Animals were randomly allocated in experimental groups, through an internal coding system. Considering the lack of relevant covariates influencing prion disease susceptibility in the single animal model used throughout the study, no further randomization strategy was required.
Blinding	Animals were blindly allocated in experimental groups thanks to the internal coding system used also for randomization. Therefore, to minimize biases, the personnel assessing clinical signs was unaware of specific group assignments.

Reporting for specific materials, systems and methods

We require information from authors about some types of materials, experimental systems and methods used in many studies. Here, indicate whether each material, system or method listed is relevant to your study. If you are not sure if a list item applies to your research, read the appropriate section before selecting a response.

Materials & experimental systems

n/a	Involved in the study
☐	☒ Antibodies
☒	☐ Eukaryotic cell lines
☒	☐ Palaeontology and archaeology
☐	☒ Animals and other organisms
☒	☐ Clinical data
☒	☐ Dual use research of concern
☒	☐ Plants

Methods

n/a	Involved in the study
☒	☐ ChIP-seq
☒	☐ Flow cytometry
☒	☐ MRI-based neuroimaging

Antibodies

Antibodies used	Primary antibody: Sha31 (Bertin Bioreagents, cat. No. A03213, clone Sha31, lot no. 2013) was used at 1:4000 dilution.<br>Secondary antibody: m-IgGκ BP-HRP, Santa Cruz Biotechnology, cat. No. sc-516102, lot no. D1123 used at dilution 1:3000.
Validation	Validation of the primary Sha31 antibody for its use with bank vole prion protein and Western blotting can be found in several different manuscripts. For example: Nonno R, et al. Studies in bank voles reveal strain differences between chronic wasting disease prions from Norway and North America. Proc Natl Acad Sci U S A. 2020 Dec 8;117(49):31417-31426. doi: 10.1073/pnas.2013237117. Espinosa JC, et al. PrPC Governs Susceptibility to Prion Strains in Bank Vole, While Other Host Factors Modulate Strain Features. J Virol. 2016 Nov 14;90(23):10660-10669. doi: 10.1128/JVI.01592-16. The first reference has been added to the manuscript as proof of validation of the antibody.

Animals and other research organisms

Policy information about studies involving animals; ARRIVE guidelines recommended for reporting animal research, and Sex and Gender in Research

Laboratory animals	We used transgenic mice expressing bank vole PrP, TgVole 1x mice. [strain name FVB/N-Tg(Prnp-BVole109I)C594PRC/J, genetic background FVB/N, age and sex and randomization codes detailed in the Source Data, publicly available in Zenodo (10.5281/zenodo.10579518). All female animals were used with ages ranging from 28 to 54 days at inoculation. We used a BSL-1 animal facility to breed the animals (CIC bioGUNE) and another two BSL-3 facilities to inoculate them (Neiker and CReSA). The permits and ethics comittee aproval codes are described in the manuscript, as well as the guidelines followed for their care. Mice were kept in a controlled environment at a room temperature of 22 ºC, 12 h light-darkness cycle and 60% relative humidity. This information has been included in the manuscript as indicated.
Wild animals	No wild animals were used in this study.
Reporting on sex	Female TgVole animals were used because prion diseases do not have any differential effect depending on sex and they are less aggressive to be housed together for long periods of time.
Field-collected samples	No filed collected samples were used in this study.
Ethics oversight	The project was approved by the Ethical Committees on Animal Welfare (project codes assigned by the Ethical Comittees P-CBG-CBBA-0519 (CIC bioGUNE), NEIKER-OEBA-2021-003 (Neiker) and project 11926679 (CReSA). CIC bioGUNE animal facility is ALAAAC certified.

Note that full information on the approval of the study protocol must also be provided in the manuscript.

Plants

Seed stocks	N/A
Novel plant genotypes	N/A
Authentication	N/A
