# Supplementary material for: A Protein Misfolding Shaking Amplification-based method for the spontaneous generation of hundreds of bona fide prions
Source: Nat Commun. 2024 Mar 8;15:2112. doi: 10.1038/s41467-024-46360-2 (PMC10923866; doi:10.1038/s41467-024-46360-2)
Supplement: Supplementary file 4 — Description of Additional Supplementary Files [file 41467_2024_46360_MOESM4_ESM.pdf]

Supplementary Data 1

Individual files accessible also in the PrPdex webpage summarizing the results for each of the 382 prion proteins analyzed in PMSA.
