# Supplementary material for: A Protein Misfolding Shaking Amplification-based method for the spontaneous generation of hundreds of bona fide prions
Source: Nat Commun. 2024 Mar 8;15:2112. doi: 10.1038/s41467-024-46360-2 (PMC10923866; doi:10.1038/s41467-024-46360-2)
Supplement: Supplementary file 5 — Supplementary Data 1 [file 41467_2024_46360_MOESM5_ESM.pdf]

Cape golden mole

GenBank: XM_006870413

Chrysochloris asiatica

Afrosoricida 1/4
Chrysochloridae 1/1

P36%

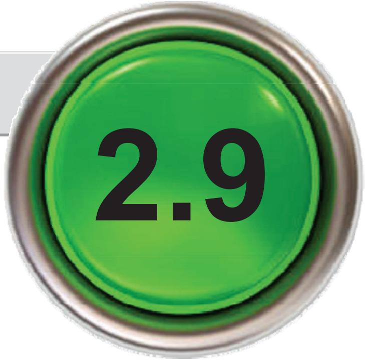

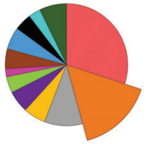

0-10

1 species sharing the same primary sequence

Hottentot golden mole
Amblysomus hottentotus

β2-α2 LOOP
NNQNNF
164

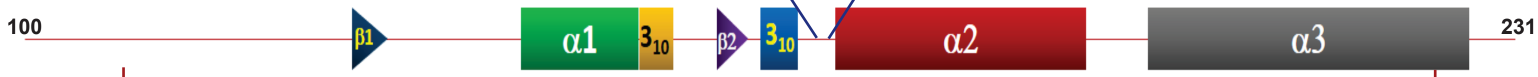

WNKPNKPKTNMKHMAGAAAAGAVVGGLGGYMLGSAMSRPPMHFGNDYEDRYYRENMNRYPNQVYYYRPVDQYNNQNNFVHDCINITVKQHTTTTTTKGENFTETDIKIMERVVEQMCITQYEKASLEYHRGSS-

S I V MI W V I V V M V Q E QA YE R S

PrP sequence differs by 15 amino acids from the bank vole PrP (see in red)

No deletions
No insertions
No polymorphic variants found

135

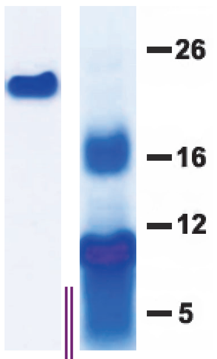

204 AA

1 Conformers

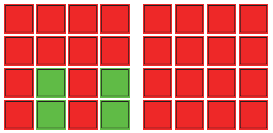

Tm[Exp]: ND

ΔΔG: ND

In vitro studies

TgVole (1x) Not tested

Cape golden mole Not tested

In vivo studies

TgVole (1x) Not tested

Cape golden mole Not tested

pLDDT
[89.33]

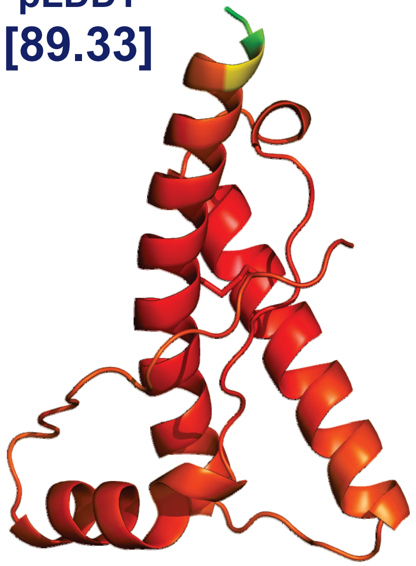

Protein code: 1128

Lesser hedgehog tenrec

GenBank: BK064944

Echinops telfairi

Afrosoricida 1/4
Tenrecidae 0/3

P29%

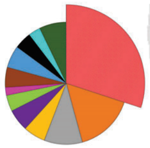

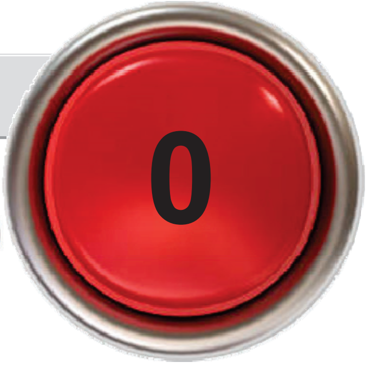

0

β2-α2 LOOP
NNQNNF
164

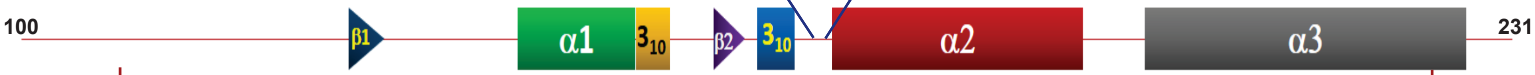

WNKPSKPKTNAKQMLGAAAAGAVVGGLGGYVVGSAMSRPLMHFGNDYEDRYYRENMHRYPNQVYYRPVQEYNNQNNFVHDCVNITVKHHTTTTTTKGENFTETDVKIMERVVEQMCVTQYQQASLDYQRGLR-
I HVA ML MI W N DQ I Q V M KE QA YE RSS

PrP sequence differs by 20 amino acids from the bank vole PrP (see in red)

No deletions
No insertions
No polymorphic variants found

Distinct primary sequence across species

Tm^{Exp}: ND

ΔΔG: ND

PK - rec-PrP

MW (kDa)

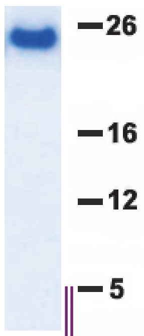

233 AA

NO MISFOLDING

0
Conformers

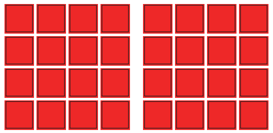

In vitro studies

NOT APPLICABLE

In vivo studies

NOT APPLICABLE

pLDDT
[92.61]

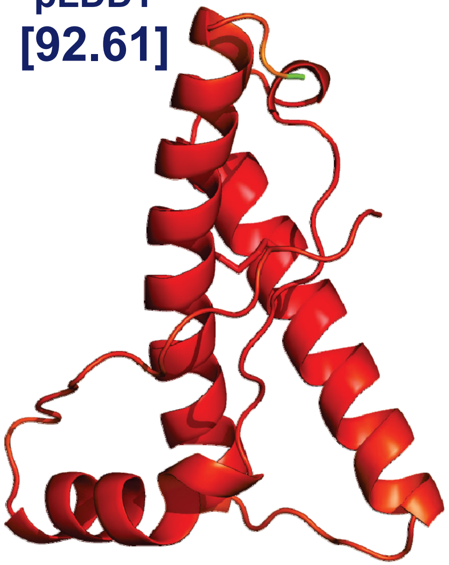

Protein code: 1082

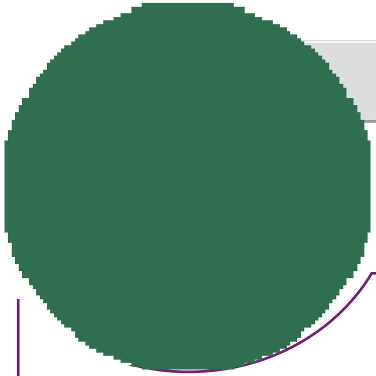

Talazac's shrew tenrec

GenBank: BK063959

Microgale talazaci

Afrosoricida 1/4

Tenrecidae 0/3

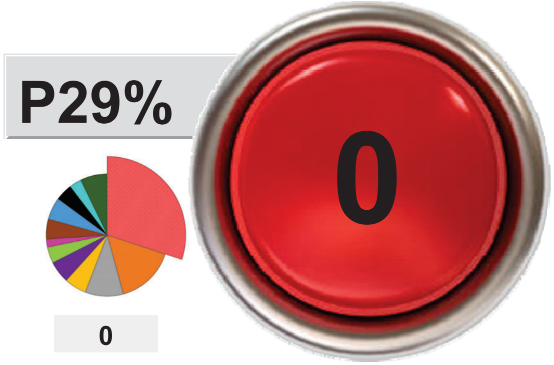

β2-α2 LOOP
SNQNNF
273

100 β1 α1 3_{10} β2 3_{10} α2 α3 231

```
WNKPNKPKTNTKHMLGAAAAGAVVGGLGGYMVGSAMSRPLMHFGNDYEDRYYRENMHRYPNQVFYRPVQEYSNQNNFVHDCVNFTVKEHTTTTTTKGENFTQTDIKIMERVVEQMCITQYQQASLEYQEYQR-
     S     I  VA                L       MI     W            N        Y    DQ N                I I Q  V               E   V M            V     KE QA Y GRSS
```

PrP sequence differs by 24 amino acids from the bank vole PrP (see in red)

No deletions

No insertions

No polymorphic variants found

Distinct primary sequence across species

Tm^{Exp}: ND

ΔΔG: ND

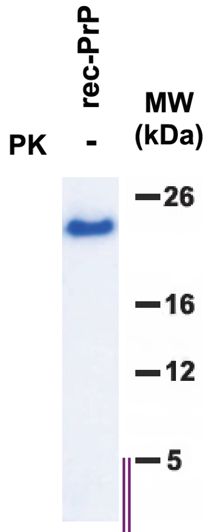

214 AA

NO MISFOLDING

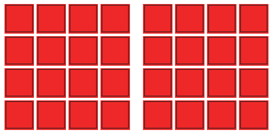

0 Conformers

In vitro studies

NOT APPLICABLE

In vivo studies

NOT APPLICABLE

pLDDT [96.16]

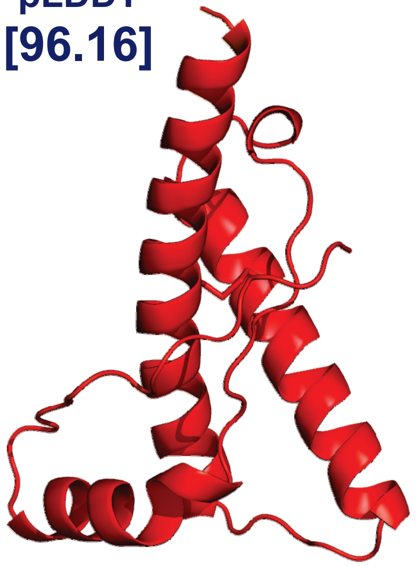

Protein code: 3087

Tailless tenrec

GenBank: AY133060

P29%

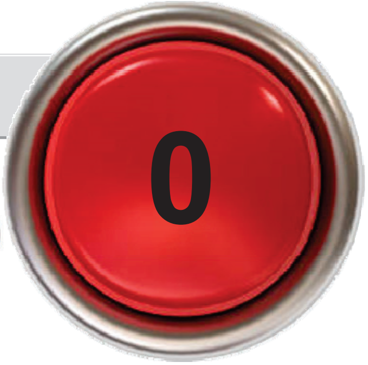

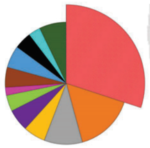

0

Tenrec ecaudatus

Afrosoricida 1/4

Tenrecidae 0/3

β2-α2 LOOP
NNQNNF
164

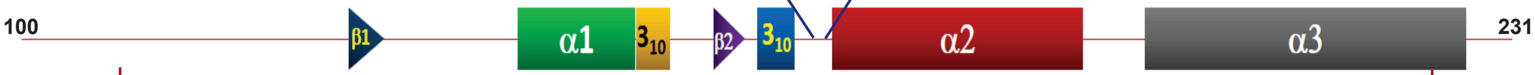

WNKPNKPKTNTKQVLGAAAAGAVVGGLGGYMLGSAMSRPLMHFGNDYEDRYYRENMHRYPNQVYYRPVQEYNNQNNFVHDCVNITVKQHTTTTTTKGENFTETDVKIMERVVEQMCITQYQQASLDYQRGLH-

S I H A MI W N DQ I V M V KE QA YE RSS

PrP sequence differs by 17 amino acids from the bank vole PrP (see in red)

No deletions

No insertions

No polymorphic variants found

Distinct primary sequence across species

Tm^{Exp}: ND

ΔΔG: ND

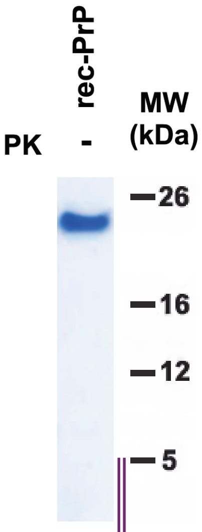

224 AA

NO MISFOLDING

0 Conformers

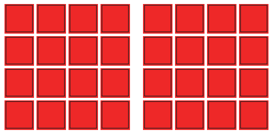

In vitro studies

NOT APPLICABLE

In vivo studies

NOT APPLICABLE

pLDDT [93.05]

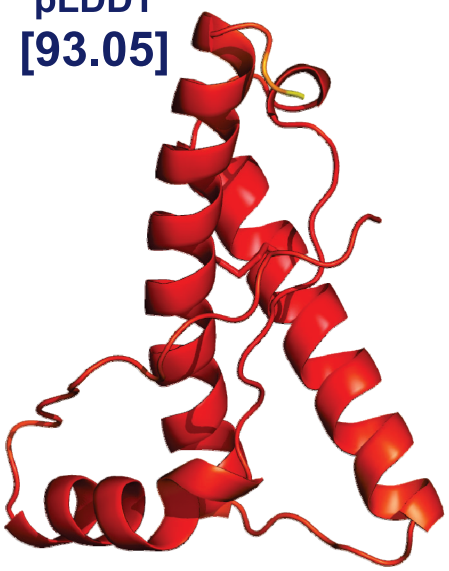

Protein code: 1347

Mongolian gazelle

GenBank: AB473615

P39%

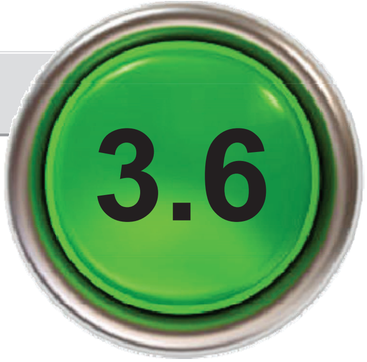

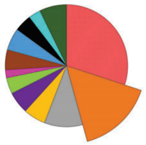

0-10

Procapra gutturosa

Artiodactyla 46/50
Bovidae 26/30

β2-α2 LOOP

0

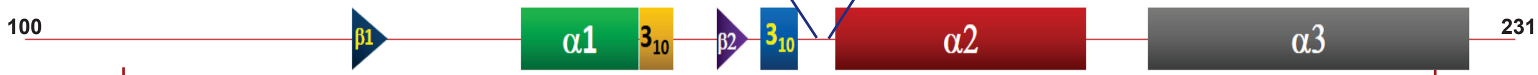

WNKPSKPKTSMKHVAGAAAAGAVVGGLGGYILGGAMSRPLIHFGNDYEDRHYRENMYRYPNQAYYRPVDQYSNQNNFVHDCVNITVKQHTVTTTTKGENFTETDIKMMERVVEQMCITQYQRESQAYYQRGAS-

PrP sequence differs by 0 amino acids from the bank vole PrP (see in red)

No deletions
No insertions
It shows polymorphic variants

Distinct primary sequence across species

135

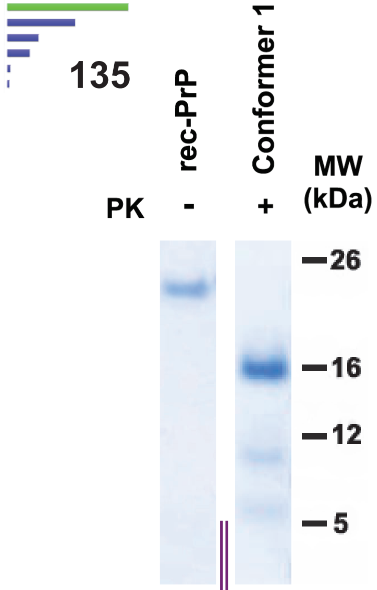

220 AA

1 Conformers

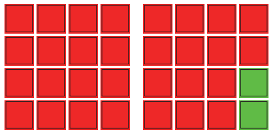

Tm^{Exp}: ND

ΔΔG: -9.58

In vitro studies

TgVole (1x)	Not tested
Mongolian gazelle	Not tested

In vivo studies

TgVole (1x)	Not tested
Mongolian gazelle	Not tested

pLDDT [88.8]

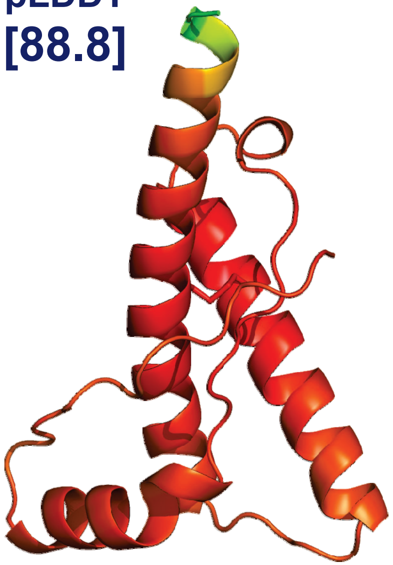

Protein code: 1143

Impala

GenBank: BK063929

Aepyceros melampus

Artiodactyla 46/50

Bovidae 26/30

Distinct primary sequence across species

P77%

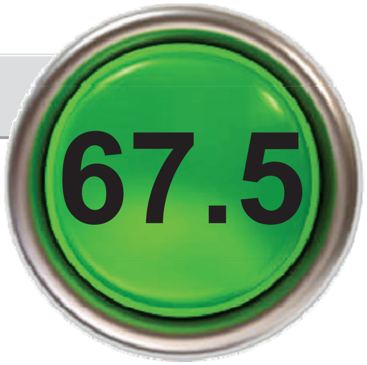

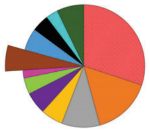

60-70

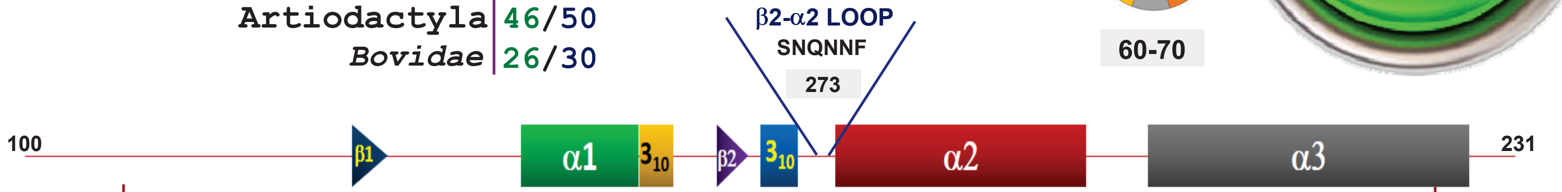

WNKPSKPKTNMKHVAGAAAAGAVVGGLGGYMLGSAMSRPLIHFGNDYEDRYYRENMYRYPNQVYYRPVDQYSNQNNFVHDCVNITVKQHTVTTTTKGENFTETDIKMMERVVEQMCITQYQRESEAYYQRGAS-

I M W N N I V V K Q EGRS

PrP sequence differs by 10 amino acids from the bank vole PrP (see in red)

No deletions

No insertions

No polymorphic variants found

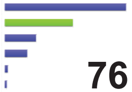

76

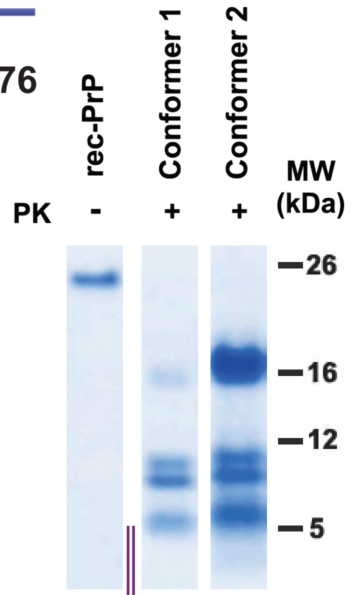

220 AA

2 Conformers

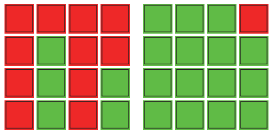

Tm^{Exp}: ND

ΔΔG: -14.79

In vitro studies

TgVole (1x) Not tested

Impala Not tested

In vivo studies

TgVole (1x) Not tested

Impala Not tested

pLDDT

[87.37]

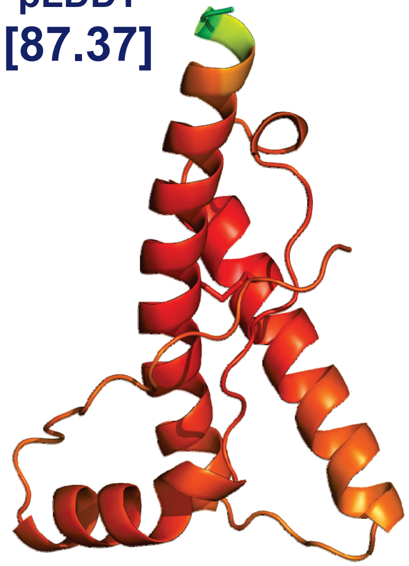

Protein code: 3047

Pronghorn

GenBank: AF156187

Antilocapra americana

Artiodactyla 46/50

Antilocapridae 1/1

Distinct primary sequence across species

P55%

19.6

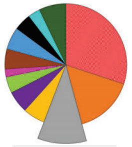

10-20

β2-α2 LOOP
SNQNTF
75

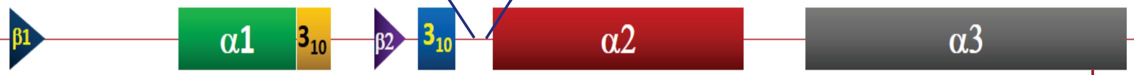

WNKPSKPKTNMKHVAGAAAAGAVVGGLGGYMLGSAMSRPLIHFGNDYEDRYYRENMYRYPNQVYYRPVDQYSNQNTFVHDCVNITVKQHTVTTTTKGENFTETDIKMMERVVEQMCITQYQRESQAYYQRGAS-

I M W N N N I V V K EGRS

PrP sequence differs by 10 amino acids from the bank vole PrP (see in red)

No deletions

No insertions

No polymorphic variants found

135

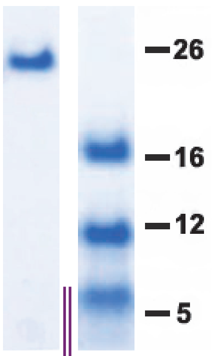

220 AA

1
Conformers

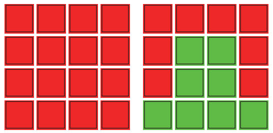

Tm^{Exp}: ND

ΔΔG: -12.73

In vitro studies

TgVole (1x) Not tested

Pronghorn Not tested

In vivo studies

TgVole (1x) Not tested

Pronghorn Not tested

pLDDT
[86.38]

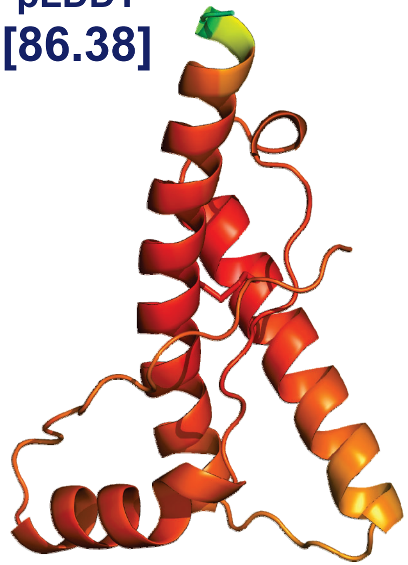

Protein code: 0945

Blackbuck

GenBank: AY720706

Antilope cervicapra

Artiodactyla 46/50
Bovidae 26/30

P75%

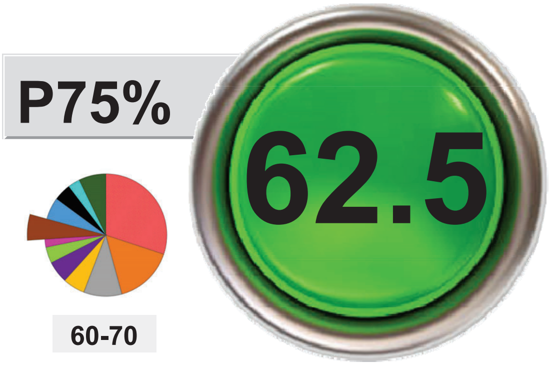

7 species sharing the same primary sequence

- **Springbok** *Antidorcas marsupialis*
- **Thomson's gazelle** *Eudorcas thomsonii*
- **Dorcas gazelle** *Gazella dorcas*
- **Goitered gazelle** *Gazella subgutturosa*
- **Dama gazelle** *Nanger dama*
- **Grant's gazelle** *Nanger granti*
- **Przewalski's gazelle** *Procapra przewalskii*

β2-α2 LOOP
SNQNNF
273

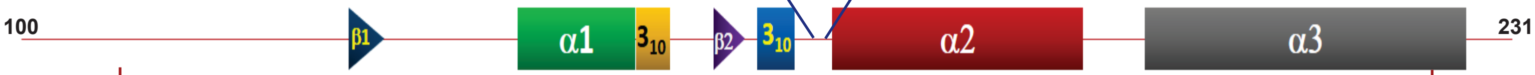

WNKPSKPKTNMKHVAGAAAAGAVVGGLGGYMLGSAMSRPLIHFGNDYEDRYYRENMYRYPNQVYYRPVDQYSNQNNFVHDCVNITVKQHTVTTTTKGENFTETDIKMMERVVEQMCITQYQRESQAYYQRGAS-

I M W N N I V V K EGRS

PrP sequence differs by 9 amino acids from the bank vole PrP (see in red)

No deletions
No insertions
No polymorphic variants found

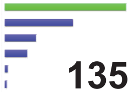

220 AA

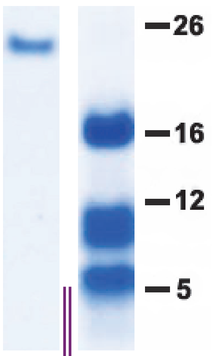

1 Conformers

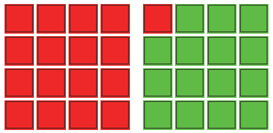

Tm^{Exp}: ND

ΔΔG: -12.66

In vitro studies

TgVole (1x)	Not tested
Blackbuck	Not tested

In vivo studies

TgVole (1x)	Not tested
Blackbuck	Not tested

pLDDT [89.11]

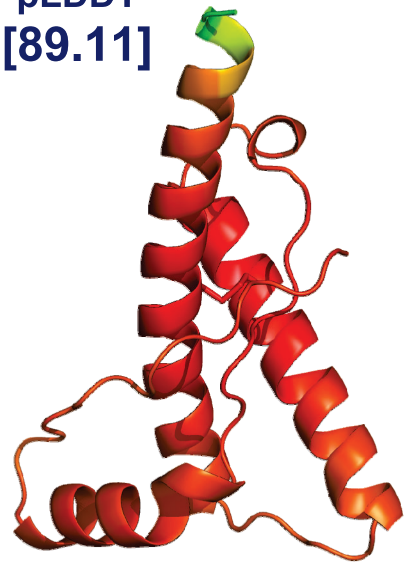

Protein code: 1350

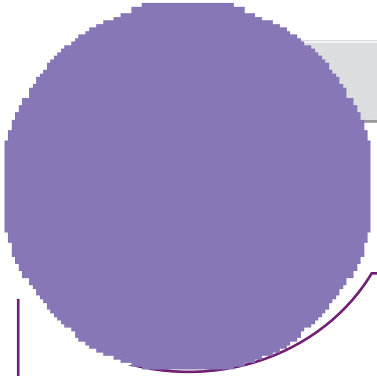

North Sulawesi babirusa

GenBank: BK064782

P63%

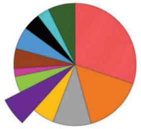

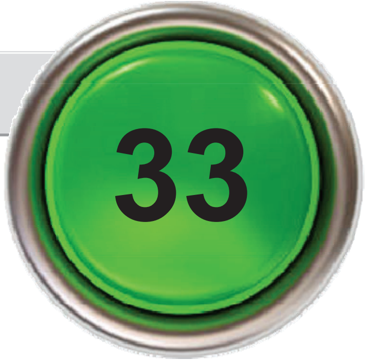

Babyrousa celebensis

Artiodactyla 46/50

Suidae 3/3

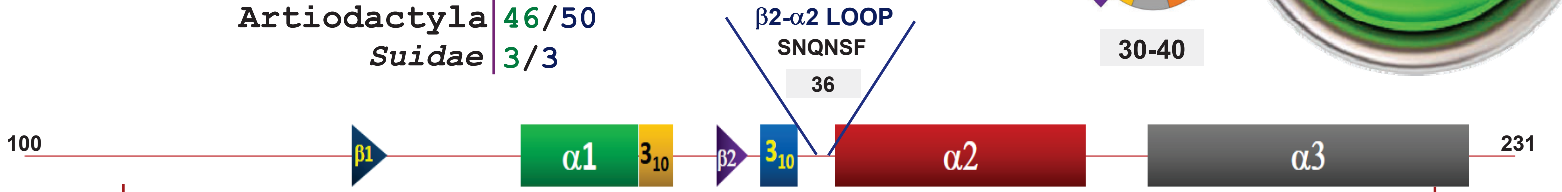

WNKPSKPKTNMKHVAGAAAAGAVVGGLGGYMLGSAMSRPLIHFGSDYEDRYYRENMYRYPNQVYYRPVDQYSNQNSFVHDCVNITVKQHTVTTTTKGENFTETDVKMIERVVEQMCITQYQKEYEAYAQRGAS-

I M N W N N N I M V SQ YEGRS

PrP sequence differs by 12 amino acids from the bank vole PrP (see in red)

No deletions

No insertions

No polymorphic variants found

Distinct primary sequence across species

135

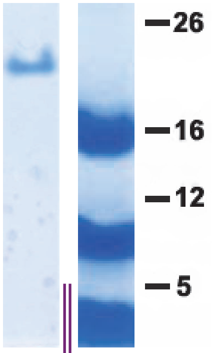

213 AA

1

Conformers

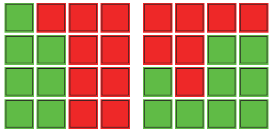

Tm^{Exp}: ND

ΔΔG: -12.04

In vitro studies

TgVole (1x) Not tested

North Sulawesi babirusa Not tested

In vivo studies

TgVole (1x) Not tested

North Sulawesi babirusa Not tested

pLDDT [87.69]

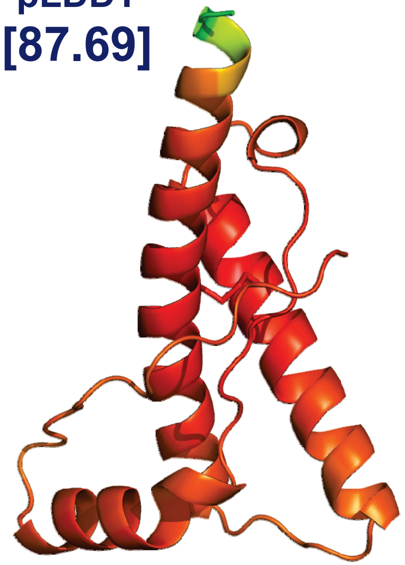

Protein code: 3940

Domestic yak

GenBank: KC137646

Distinct primary sequence across species

Bos grunniens

Artiodactyla	46/50
Bovidae	26/30

P69% **45.2**

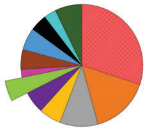

40-50

β2-α2 LOOP
SNQNNF
273

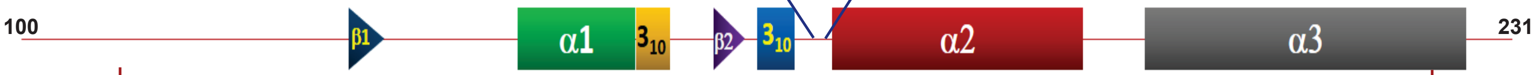


```
WNKPSKPKTNMKRVAGAAAAGAVVGGLGGYMLGSAMNRPLIHFGSDYEDRYYRENMHRYPNQVYYRPVDQYSNQNNFVHDCVNITVKEHTVTTTTKGENFTETDIKMMERVVEQMCITQYQRESQAYYQRGAS-
           I H                                    S  M    N W        N             N            I Q                 V           V    K       EGRS
```

PrP sequence differs by 13 amino acids from the bank vole PrP (see in red)

No deletions

No insertions

It shows polymorphic variants

25

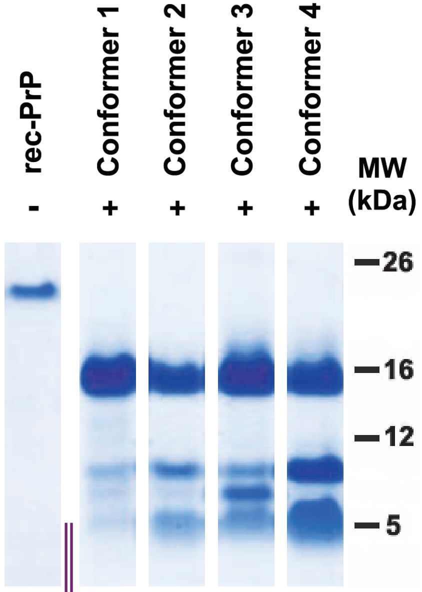

220 AA

4 Conformers

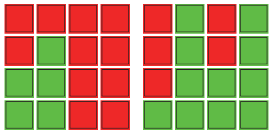

Tm^{Exp}: ND

ΔΔG: -4.17

In vitro studies

TgVole (1x)	Not tested
Domestic yak	Not tested

In vivo studies

TgVole (1x)	Not tested
Domestic yak	Not tested

pLDDT [88.15]

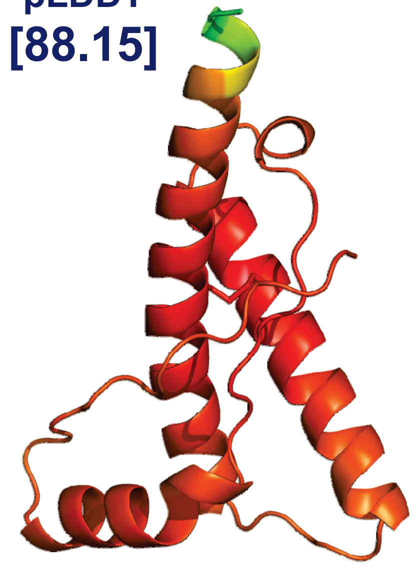

Protein code: 1748

Cow

GenBank: NM_181015

Bos taurus

Artiodactyla 46/50

Bovidae 26/30

P48%

12.5

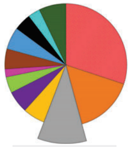

10-20

β2-α2 LOOP
SNQNNF
273

100 β1 α1 3_{10} β2 3_{10} α2 α3 231

WNKPSKPKTNMKHVAGAAAAGAVVGGLGGYMLGSAMSRPLIHFGSDYEDRYYRENMHRYPNQVYYRPVDQYSNQNNFVHDCVNITVKEHTVTTTTKGENFTETDIKMMERVVEQMCITQYQRESQAYYQRGAS-

I M N W N N I Q V V K EGRS

PrP sequence differs by 11 amino acids from the bank vole PrP (see in red)

No deletions

No insertions

It shows polymorphic variants

4 species sharing the same primary sequence

American bison
Bison bison

European bison
Bison bonasus

Gayal
Bos frontalis

Banteng
Bos javanicus

76

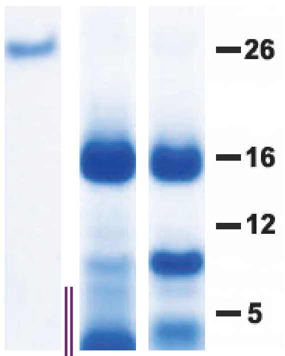

220 AA

2
Conformers

Tm^{Exp}: ND

ΔΔG: -10.33

In vitro studies

TgVole (1x)	Propagate
Cow	Propagate

In vivo studies

TgVole (1x)	Infectious
TgCow	Not tested

pLDDT
[89.11]

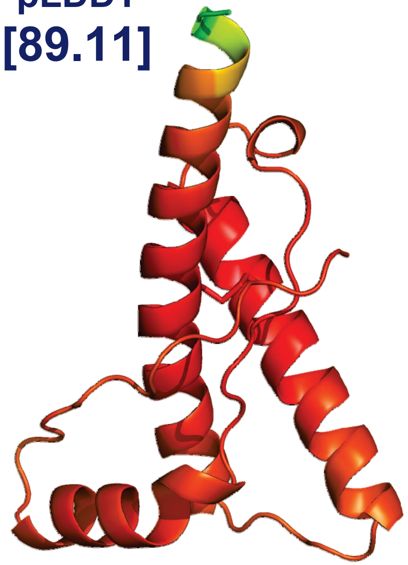

Nilgai

GenBank: AY720700

P88%

85.7

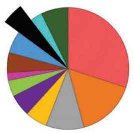

80-90

Boselaphus tragocamelus

Artiodactyla 46/50

Bovidae 26/30

β2-α2 LOOP
SNQNNF
273

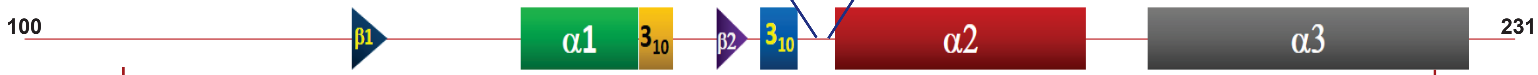

WNKPSKPKTNMKHVAGAAAAGAVVGGLGGYMLGSAMNRPLIHFGNDYEDRYYRENMHRYPNQVYYRPVDQYSNQNNFVRDCVNITVKEHTVTTTTKGENFTETDIKMMERVVEQMCITQYQTESQAYYQRGAS-

I S M W N N H I Q V V K EGRS

PrP sequence differs by 12 amino acids from the bank vole PrP (see in red)

No deletions

No insertions

No polymorphic variants found

Distinct primary sequence across species

135

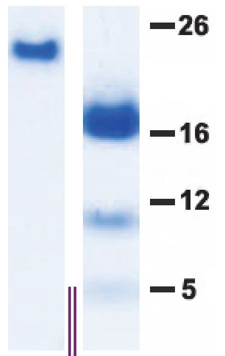

220 AA

Tm^{Exp}: ND

ΔΔG: -7.53

In vitro studies

TgVole (1x) Not tested

Nilgai Not tested

In vivo studies

TgVole (1x) Not tested

Nilgai Not tested

pLDDT [89.06]

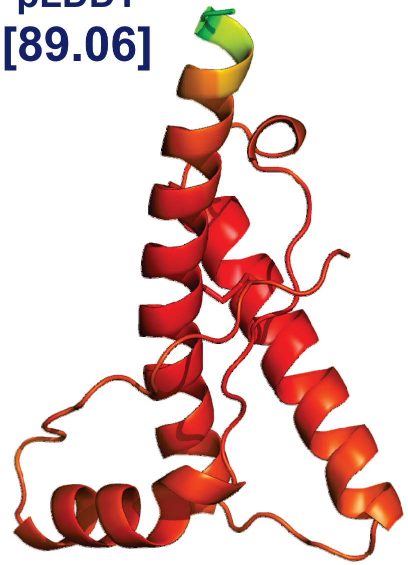

1 Conformers

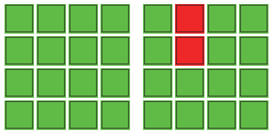

Protein code: 1357

Water buffalo

GenBank: MK342630

Bubalus bubalis

Artiodactyla 46/50
Bovidae 26/30

P51%

16.1

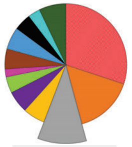

Distinct primary sequence across species

β2-α2 LOOP
SNQNNF
273

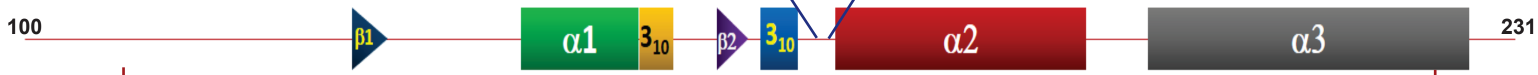

WNKPSKPKTNMKHMAGAAAAGAVVGGLGGYMLGSAMSRPLIHFGNDYEDRYYRENMHRYPNQVYYRPVDQYSNQNNFVHDCVNITVKEHTVTTTTKGENFTETDIKMMERVVEQMCITQYQRESQAYYQRGAS-
I V M W N N I Q V V K EGRS

PrP sequence differs by 11 amino acids from the bank vole PrP (see in red)

No deletions
No insertions
It shows polymorphic variants

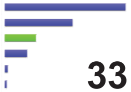

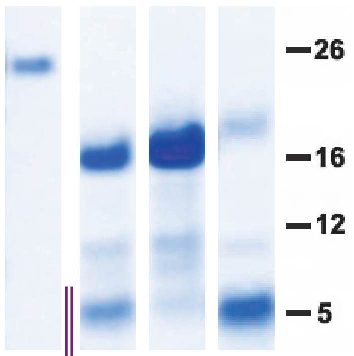

220 AA

3
Conformers

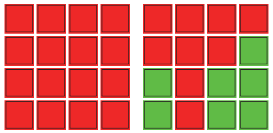

Tm[Exp]: ND

ΔΔG: -12.88

In vitro studies

TgVole (1x) Not tested

Water buffalo Not tested

In vivo studies

TgVole (1x) Not tested

Water buffalo Not tested

pLDDT
[89.18]

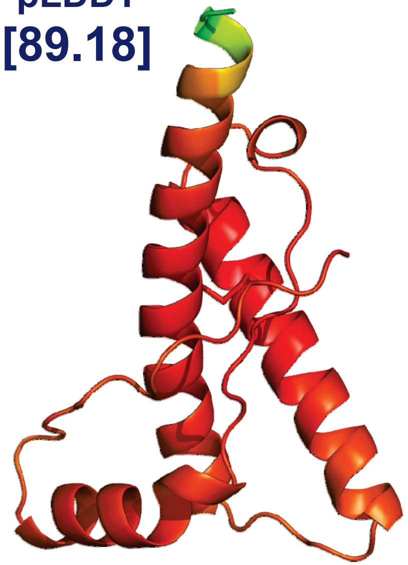

Protein code: 1198

Anoa

GenBank: BK063916

Bubalus depressicornis

Artiodactyla 46/50

Bovidae 26/30

P29%

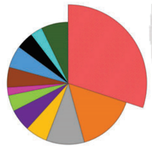

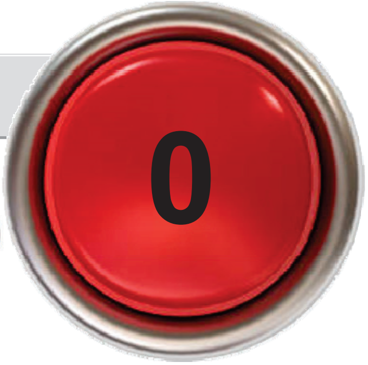

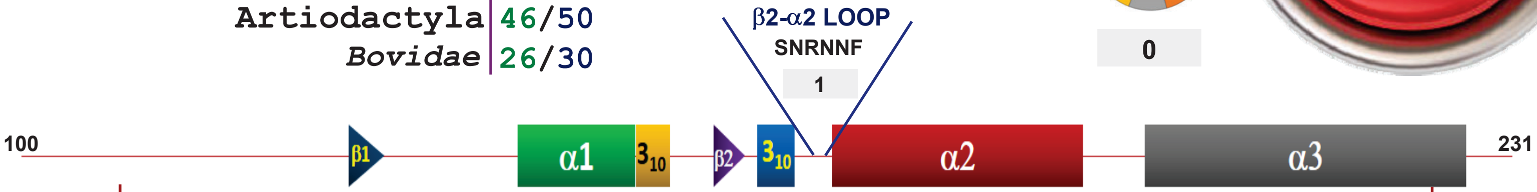


```
WNKPSKPKTNMKHMAGAAAAGAVVGGLGGYMLGSAMSRPLIHFGNDYEDRYYRENMHRYPNQVYYRPVDQYSNRNNFVHDCVNITVKEHTVTTTTKGENFTETDIKMMERVVEQMCITQYQRESQAYYQRGAS-
          I V                        M     W      N         N Q      I Q            V      V   K    EGRS
```

PrP sequence differs by 12 amino acids from the bank vole PrP (see in red)

No deletions

No insertions

No polymorphic variants found

Distinct primary sequence across species

Tm[Exp]: ND

ΔΔG: -12.62

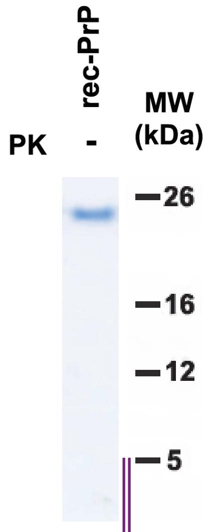

220 AA

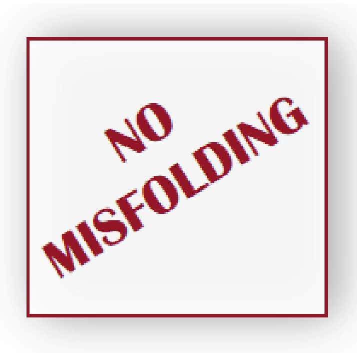

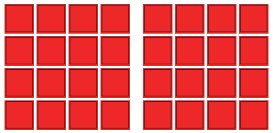

0

Conformers

In vitro studies

NOT APPLICABLE

In vivo studies

NOT APPLICABLE

pLDDT [86.73]

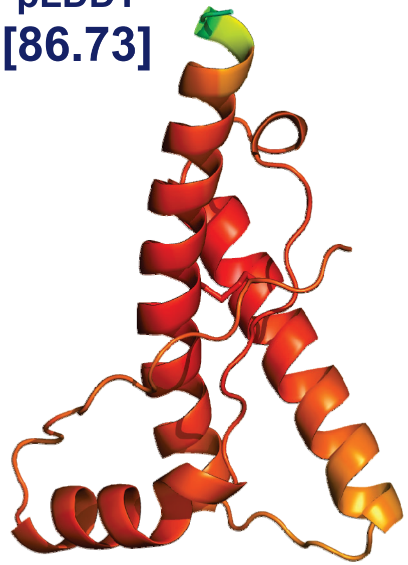

Protein code: 1363

Takin

GenBank: AB060290

P56%

22.3

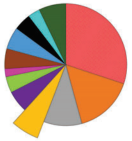

20-30

Budorcas taxicolor

Artiodactyla 46/50

Bovidae 26/30

β2-α2 LOOP
SNQNNF
273

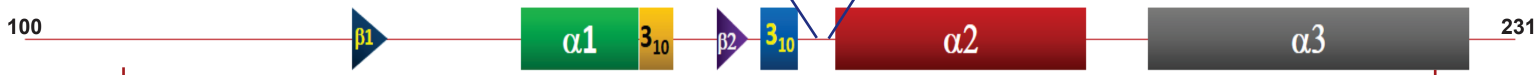

WNKPSKPKTNMKHVAGAAAAGAVVGGLGGYMLGSAMSRPLIHFGSDYEDRYYRENMYRYPNQVYYRPVDQYSNQNNFVHDCVNITVKQHTVTTTTKGENFTETDIKIMERVVEQMCITQYQRESQAYYQRGAS-

I M N W N N I V M V K EGRS

PrP sequence differs by 11 amino acids from the bank vole PrP (see in red)

No deletions

No insertions

No polymorphic variants found

Distinct primary sequence across species

135

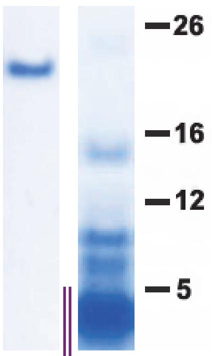

212 AA

1

Conformers

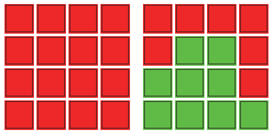

Tm^{Exp}: ND

ΔΔG: -12.4

In vitro studies

TgVole (1x) Not tested

Takin Not tested

In vivo studies

TgVole (1x) Not tested

Takin Not tested

pLDDT
[86]

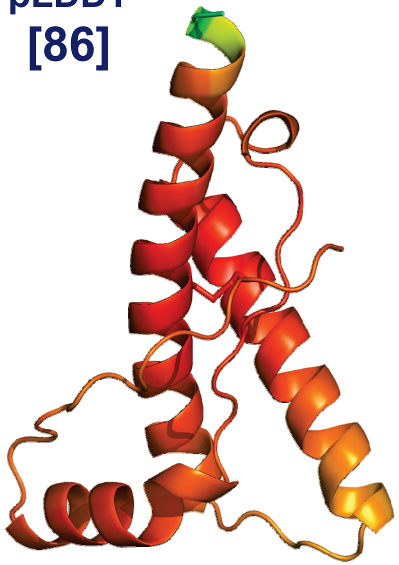

Protein code: 1348

Bactrian camel

GenBank: HQ204566

Camelus bactrianus ferus

Artiodactyla 46/50
Camelidae 2/2

1 species sharing the same primary sequence

Camel
Camelus dromedarius

P45%

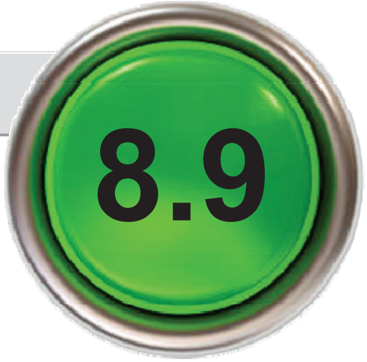

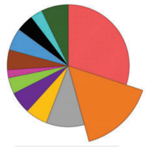

0-10

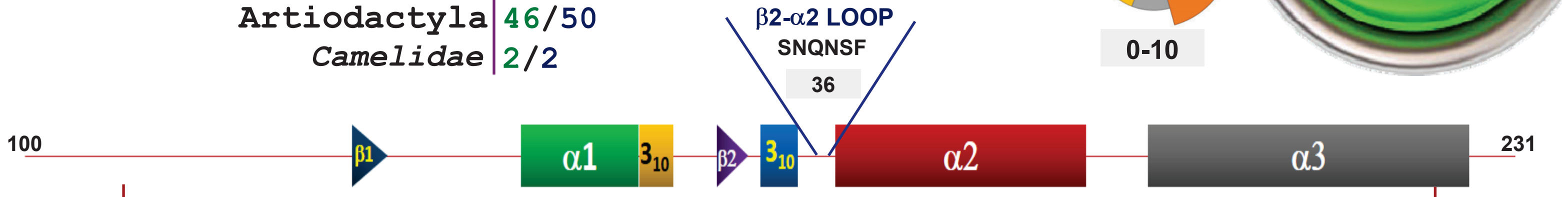


```
WNKPSKPKTSMKHVAGAAAAGAVVGGLGGYMLGSAMSRPLIHFGNDYEDRYYRENMYRYPNQVYYKPVDQYSNQNSFVHDCVNITVKQHTVTTTTKGENFTETDVKMMERVVEQMCITQYQREYQASYGRGAS-
         NI                            M     W       N     R     N  N       I                            V   K S  Y EGRS
```

PrP sequence differs by 12 amino acids from the bank vole PrP (see in red)

No deletions
No insertions
No polymorphic variants found

135

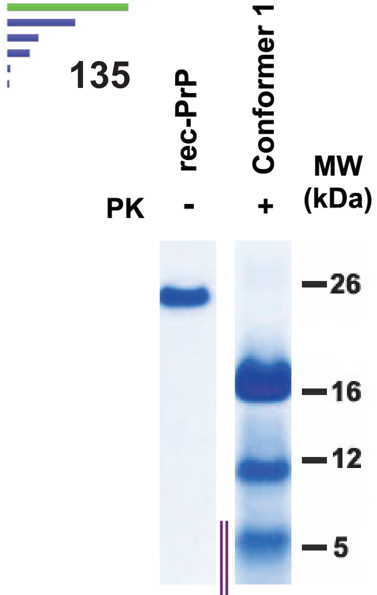

212 AA

1
Conformers

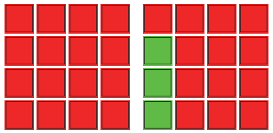

Tm[Exp]: ND
ΔΔG: -3.78

pLDDT
[85.87]

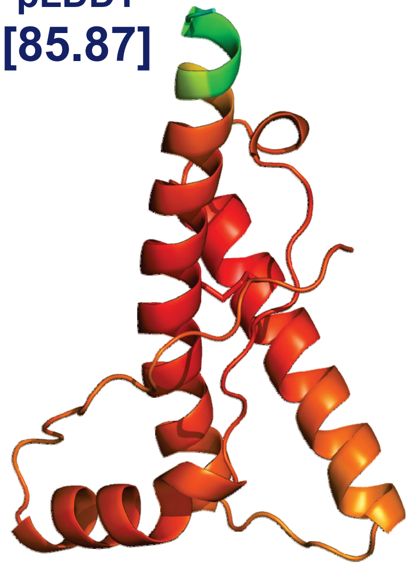

In vitro studies

TgVole (1x)	Not tested
Bactrian camel	Not tested

In vivo studies

TgVole (1x)	Not tested
Bactrian camel	Not tested

Protein code: 0544

Chacoan peccary

GenBank: BK063918

P67%

40

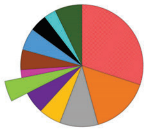

40-50

Catagonus wagneri

Artiodactyla 46/50

Tayassuidae 1/1

β2-α2 LOOP
SSQNNF
42

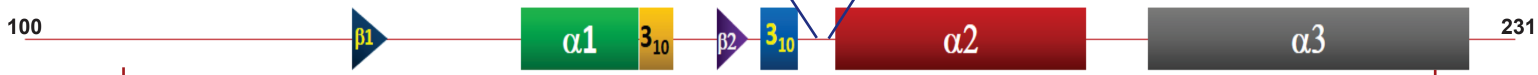


```
WNKPSKPKTSMKHVAGAAAAGAVVGGLGGYMLGSAMSRPLIHFGSDYEDRYYRENMYRYPNQVYYRPVDQYSSQNNFVHDCVNITIKQHTVTTTTKGENFTETDIKMMERVVEQMCTTQYQREYEAYAQRGAS-
          NI                        M    N W        N          NN                             V         V    K SQ  YEGRS
```

PrP sequence differs by 13 amino acids from the bank vole PrP (see in red)

No deletions

No insertions

No polymorphic variants found

1 species sharing the same primary sequence

Collared peccary
Dicotyles tajacu

2

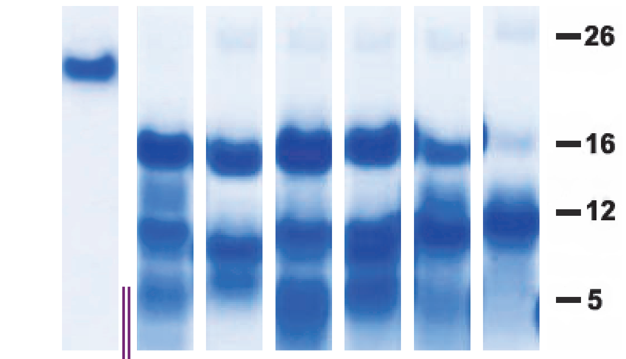

213 AA

6
Conformers

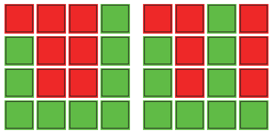

Tm^{Exp}: ND

ΔΔG: -17.92

***In vitro* studies**

TgVole (1x) — Not tested

Chacoan peccary — Not tested

***In vivo* studies**

TgVole (1x) — Not tested

Chacoan peccary — Not tested

pLDDT
[88.23]

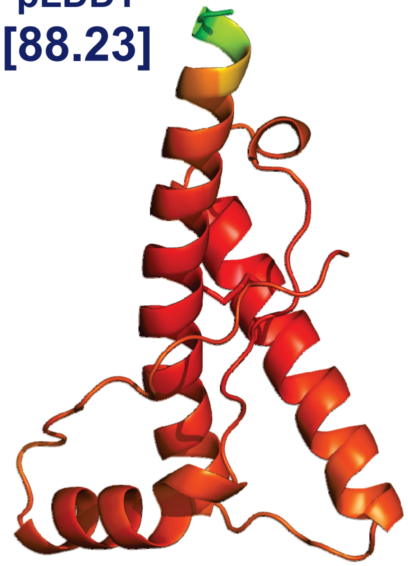

Protein code: 2022

Elk

GenBank: EU032294

Cervus elaphus canadensis

Artiodactyla 46/50
Cervidae 6/6

3 species sharing the same primary sequence

Indian hog deer *Axis porcinus*

Sika deer *Cervus nippon*

Thorold's deer *Przewalskium albirostris*

P100%

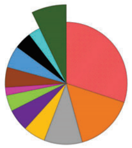

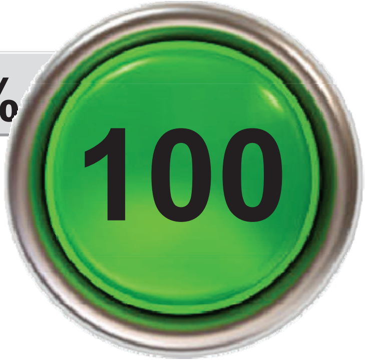

100

β2-α2 LOOP
NNQNTF
72

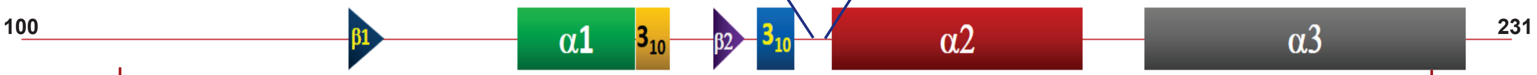

WNKPSKPKTNMKHVAGAAAAGAVVGGLGGYMLGSAMSRPLIHFGNDYEDRYYRENMYRYPNQVYYRPVDQYNNQNTFVHDCVNITVKQHTVTTTTKGENFTETDIKMMERVVEQMCITQYQRESEAYYQRGAS-
I M W N N I V V K Q EGRS

PrP sequence differs by 10 amino acids from the bank vole PrP (see in red)

No deletions
No insertions
It shows polymorphic variants

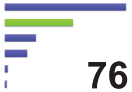

76

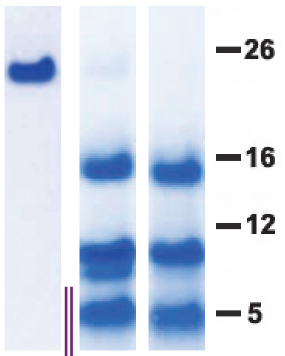

212 AA

2 Conformers

Tm^{Exp}: 69 ºC

ΔΔG: -11.73

pLDDT [89.43]

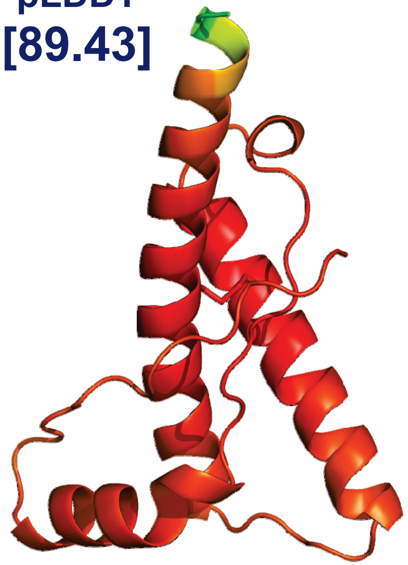

In vitro studies

TgVole (1x)	Propagate
Elk	Propagate

In vivo studies

TgVole (1x)	Infectious
TgElk	Ongoing

Protein code: 1178

Blue wildebeest

GenBank: EF165086

Connochaetes taurinus

Artiodactyla 46/50

Bovidae 26/30

P39%

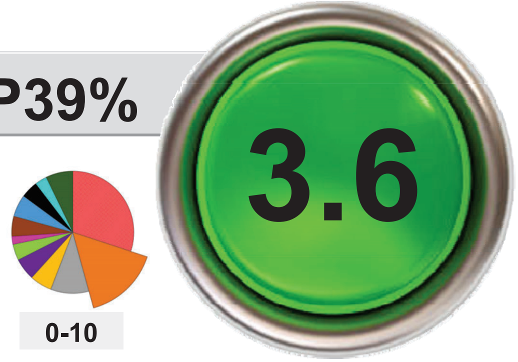

1 species sharing the same primary sequence

Black wildebeest
Connochaetes gnou

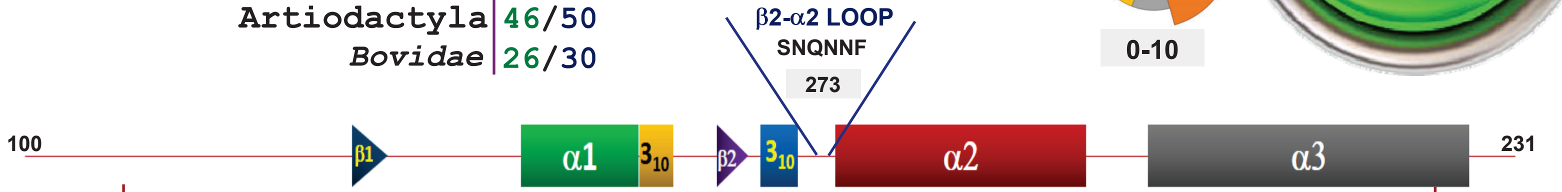

WNKPSKPKTNMKHMAGAAAAGAVVGGLGGYMLGSAMSRPLIHFGNDYEDRYYRENMYRYPNQVYYRPVDQYSNQNNFVHDCVNITVKQHTVTTTTKGENFTETDIKIMERVVEQMCITQYQRESQAYYQRGAS-

I V M W N N I V M V K EGRS

PrP sequence differs by 11 amino acids from the bank vole PrP (see in red)

No deletions

No insertions

No polymorphic variants found

135

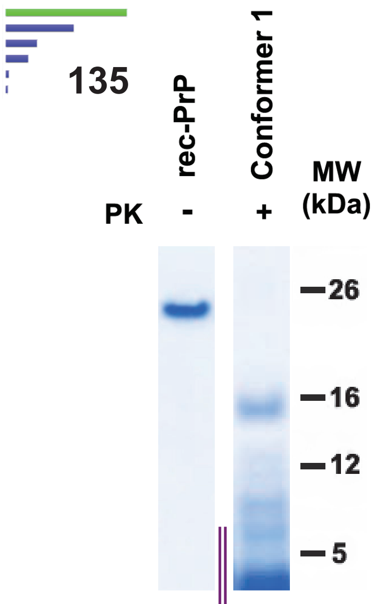

212 AA

1

Conformers

Tm^{Exp}: ND

ΔΔG: -8.49

In vitro studies

TgVole (1x) Not tested

Blue wildebeest Not tested

In vivo studies

TgVole (1x) Not tested

Blue wildebeest Not tested

pLDDT [89.2]

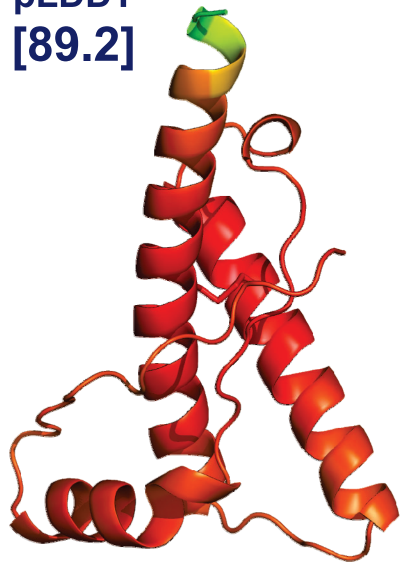

Protein code: 1414

Fallow deer

GenBank: EF139175

P100%

100

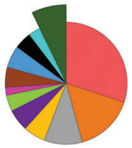

100

Dama dama

Artiodactyla 46/50

Cervidae 6/6

β2-α2 LOOP

NNQNTF

72

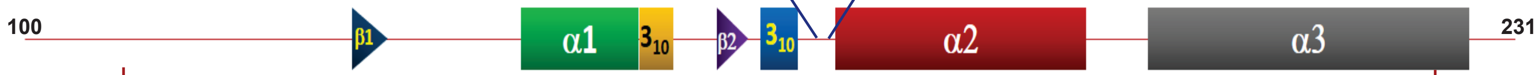

WNKPSKPKTNMKHVAGAAAAGAVVGGLGGYMLGSAMNRPLIHFGNDYEDRYYRENMYRYPNQVYYRPVDQYNNQNTFVHDCVNITVKQHTVTTTTKGENFTETDIKMMERVVEQMCITQYQRESEAYYQRGAS-

I S M W N N I V V K Q EGRS

PrP sequence differs by 11 amino acids from the bank vole PrP (see in red)

No deletions

No insertions

No polymorphic variants found

Distinct primary sequence across species

33

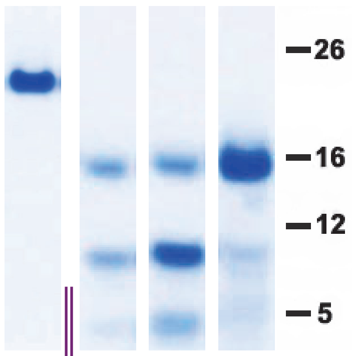

212 AA

3 Conformers

Tm^{Exp}: 66.8 ºC

ΔΔG: -10.8

In vitro studies

TgVole (1x)	Not tested
Fallow deer	Not tested

In vivo studies

TgVole (1x)	Not tested
Fallow deer	Not tested

pLDDT [88.45]

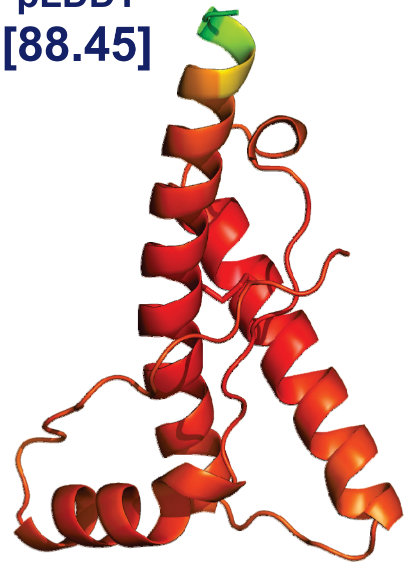

Protein code: 0980

Reticulata giraffe

GenBank: AF113942

P35%

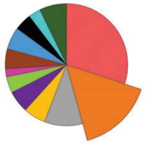

0-10

1.6

Giraffa camelopardalis reticulata

Artiodactyla 46/50

Giraffidae 2/2

β2-α2 LOOP

SNQNTF

75

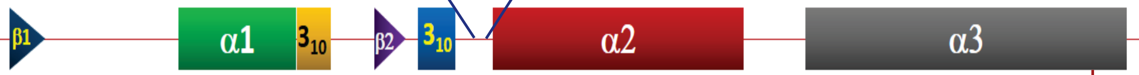

WNKPSKPKTNMKHMAGAAAAGAVVGGLGGYMLGSAMSRPLIHFGNDYEDRYYRENMYRYPNQVYYRPVDQYSNQNTFVHDCVNITVKQHTVTTTTKGENFTETDIKMMERVVEQMCITQYQRESEAYYQRGAS-

I V M W N N N I V V K Q EGRS

PrP sequence differs by 12 amino acids from the bank vole PrP (see in red)

No deletions

No insertions

No polymorphic variants found

Distinct primary sequence across species

135

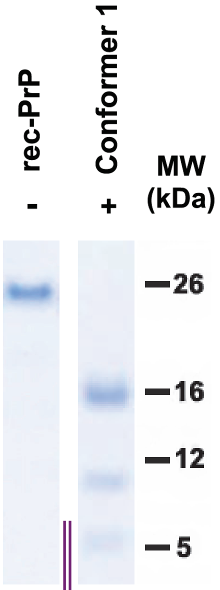

220 AA

1

Conformers

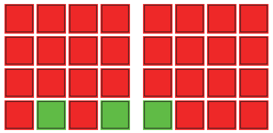

Tm^{Exp}: ND

ΔΔG: -14.52

In vitro studies

TgVole (1x) Not tested

Reticulata giraffe Not tested

In vivo studies

TgVole (1x) Not tested

Reticulata giraffe Not tested

pLDDT

[88.64]

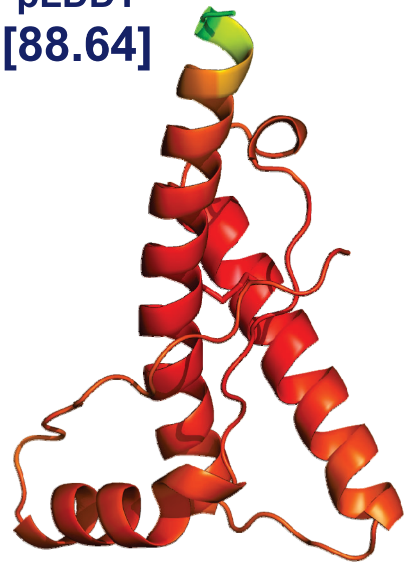

Protein code: 0951

Pygmy hippopotamus

GenBank: AB919084

Hexaprotodon liberiensis

`Artiodactyla` 46/50
`Hippopotamidae` 2/2

P45%

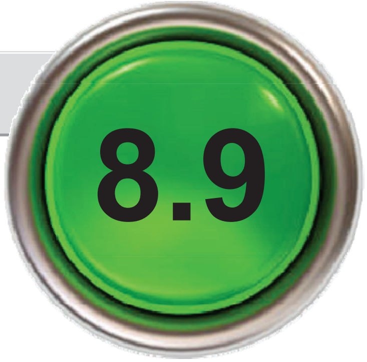

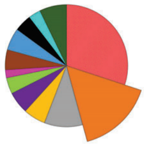

0-10

β2-α2 LOOP
HNQNNF
2

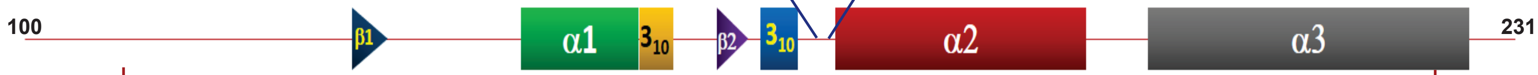

WXKPSKPKTNMKHMAGAAAAGAVVGGLGGYMLGSAMSRPLIHFGSDYEDRYYRENMHRYPNQVYYRPVDQYHNQNNFVHDCVNITVKQHTVTTTTKGENFTETDIKMIERVVEQMCITQYQKEYQAYYERGAS-
N I V M N W N N I V M V S GRS

PrP sequence differs by 12 amino acids from the bank vole PrP (see in red)

No deletions
No insertions
No polymorphic variants found

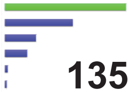

135

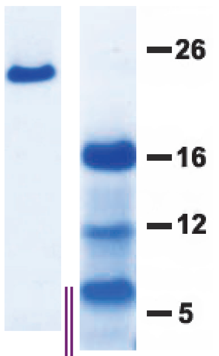

212 AA

1
Conformers

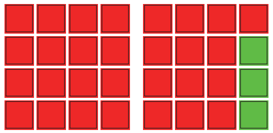

Tm^{Exp}: ND

ΔΔG: ND

In vitro studies

TgVole (1x) Not tested

Pygmy hippopotamus Not tested

In vivo studies

TgVole (1x) Not tested

Pygmy hippopotamus Not tested

pLDDT
[86.66]

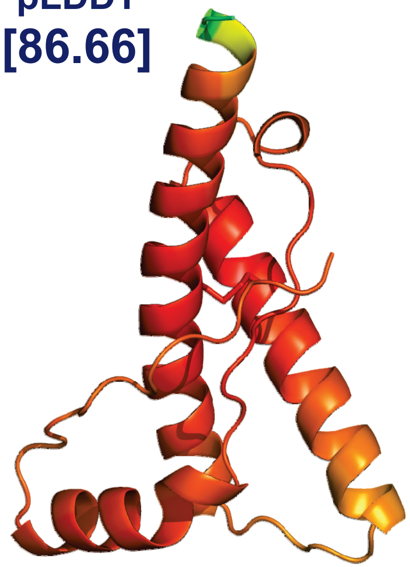

Distinct primary sequence across species

Hippopotamus

GenBank: AB919083

P34%

0.9

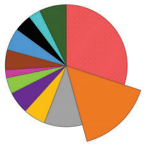

0-10

Hippopotamus amphibius

Artiodactyla 46/50

Hippopotamidae 2/2

β2-α2 LOOP
HNQNNF
2

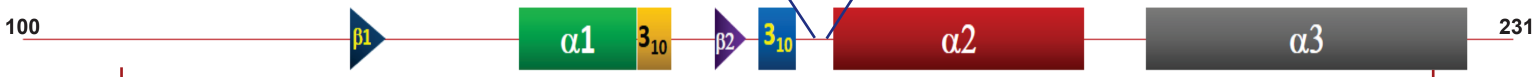

WXKPSKPKTNMKHMAGAAAAGAVVGGLGGYMLGSAMSRPLIHFGSDYEDRYYRENMHRYPNQVYYRPVDQYHNQNNFVHDCVNITVKQHTVTTTTKGENFTETDIKMIERVVEQMCITQYQKEYQAYYERGAS-

N I V M N W N N I V M V S GRS

PrP sequence differs by 12 amino acids from the bank vole PrP (see in red)

No deletions

No insertions

No polymorphic variants found

135

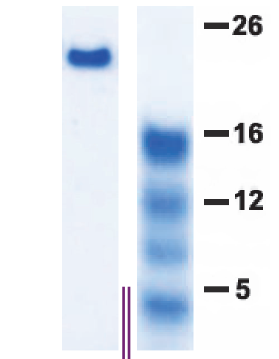

212 AA

1

Conformers

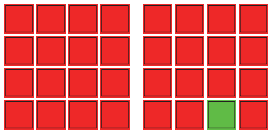

Tm^{Exp}: ND

ΔΔG: ND

In vitro studies

TgVole (1x) Not tested

Hippopotamus Not tested

In vivo studies

TgVole (1x) Not tested

Hippopotamus Not tested

pLDDT

[86.24]

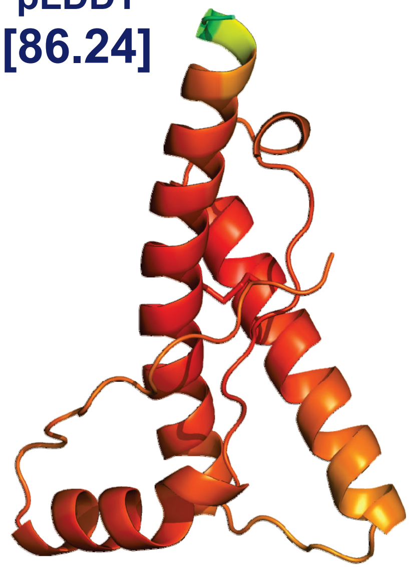

Distinct primary sequence across species

Protein code: 1359

Sable antelope

GenBank: BK064056

P88%

85.7

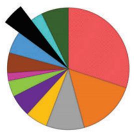

80-90

Hippotragus niger niger

Artiodactyla	46/50
Bovidae	26/30

β2-α2 LOOP
SNQNNF
273

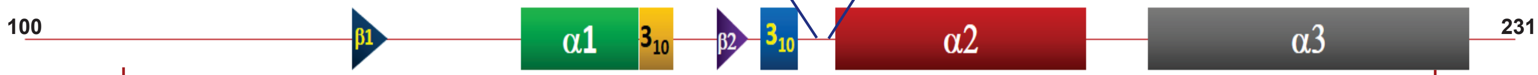

WNKPSKPKTNMKHVAGAAAAGAVVGGLGGYMLGSAMSRPLIHFGNDYEDRYYRENMYRYPNQVYYRPVDQYSNQNNFVHDCVNITVKQHTVTTTTKGENFTETDIKIMERVVEQMCITQYQRESQAYYQRGAS-

I M W N N I V M V K EGRS

PrP sequence differs by 10 amino acids from the bank vole PrP (see in red)

No deletions

No insertions

It shows polymorphic variants

5 species sharing the same primary sequence

- **Addax** *Addax nasomaculatus*
- **Hirola** *Beatragus hunteri*
- **Common tsessebe** *Damaliscus lunatus*
- **Roan antelope** *Hippotragus equinus*
- **Scimitar oryx** *Oryx dammah*

76

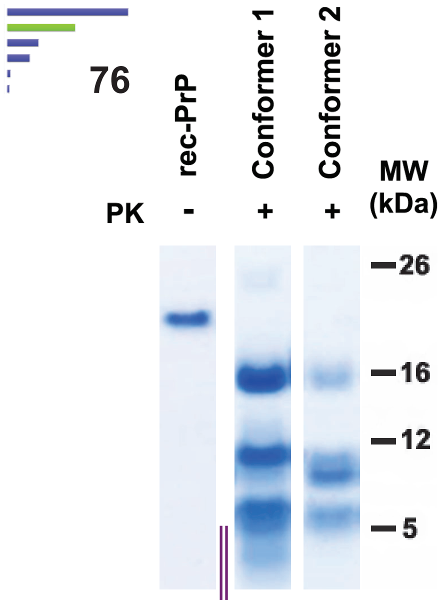

212 AA

2
Conformers

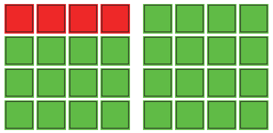

Tm^{Exp}: ND

ΔΔG: -11.98

pLDDT [88.97]

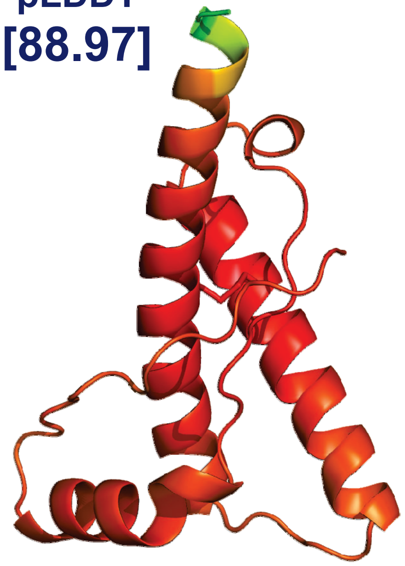

In vitro studies

TgVole (1x) Not tested

Sable antelope Not tested

In vivo studies

TgVole (1x) Not tested

Sable antelope Not tested

Protein code: 3225

Waterbuck

GenBank: EF165087

Kobus ellipsiprymnus

Artiodactyla 46/50
Bovidae 26/30

P69%

44.6

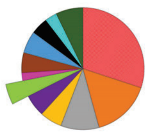

40-50

β2-α2 LOOP
SNQNNF
273

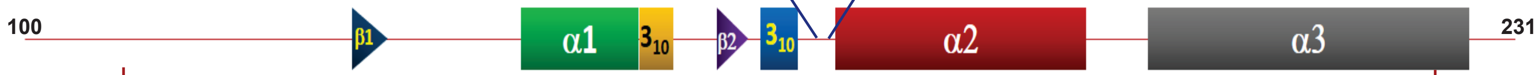

WNKPSKPKTNMKHMAGAAAAGAVVGGLGGYMLGSAMSRPLIHFGNDYEDRYYRENMYRYPNQVYYRPVDQYSNQNNFVHDCVNITVKQHTVTTTTKGENFTETDIKMMERVVEQMCITQYQRESEAYYQRGAS-

I V M W N N I V V K Q EGRS

PrP sequence differs by 11 amino acids from the bank vole PrP (see in red)

No deletions
No insertions
No polymorphic variants found

135

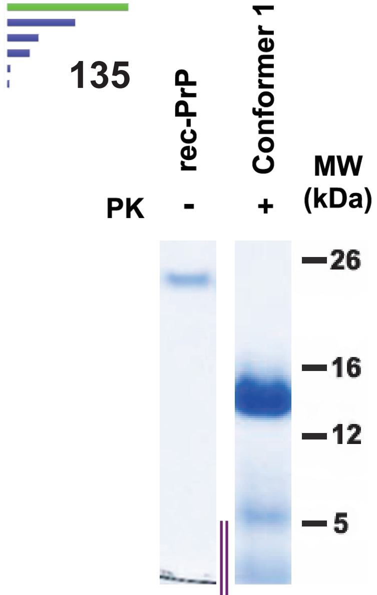

220 AA

1
Conformers

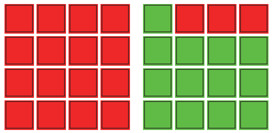

Tm[Exp]: ND

ΔΔG: -12.69

In vitro studies

TgVole (1x) Not tested

Waterbuck Not tested

In vivo studies

TgVole (1x) Not tested

Waterbuck Not tested

pLDDT
[89.78]

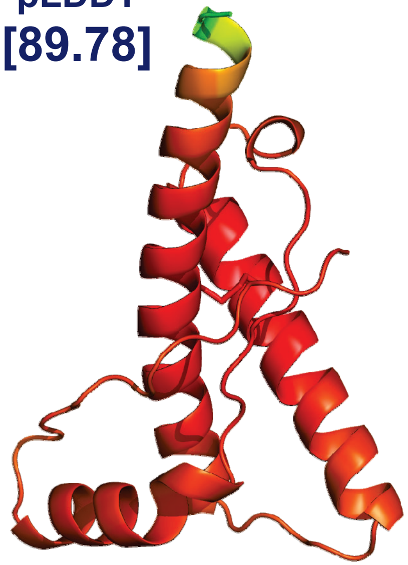

Distinct primary sequence across species

Protein code: 1351

Nile lechwe

GenBank: EF165088

Kobus megaceros

Artiodactyla 46/50

Bovidae 26/30

P82%

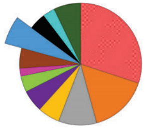

70-80

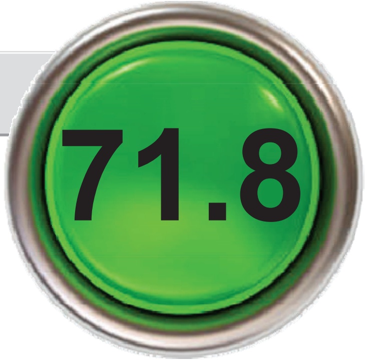

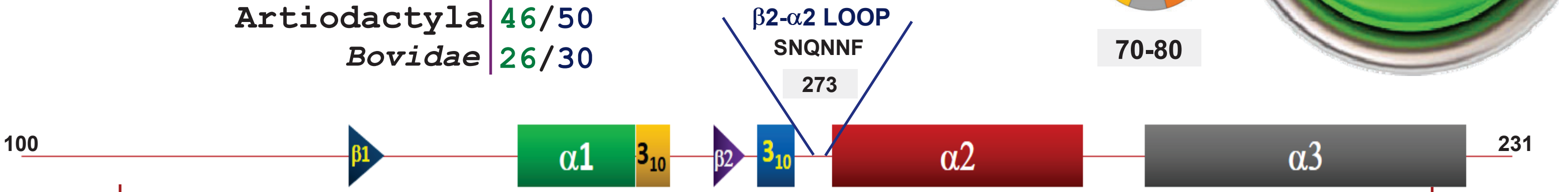

WNKPSKPKTNMKHVAGAAAAGAVVGGLGGYMLGSAMSRPLIHFGNDYEDRYYRENMYRYPNQVYYRPVDQYSNQNNFVHDCVNITVKQHTVTTTTKGENFTETDIKIMERVVEQMCITQYQRESEAYYQRGAS-

I M W N N I V M V K Q EGRS

PrP sequence differs by 11 amino acids from the bank vole PrP (see in red)

No deletions

No insertions

No polymorphic variants found

1 species sharing the same primary sequence

Lechwe
Kobus leche leche

76

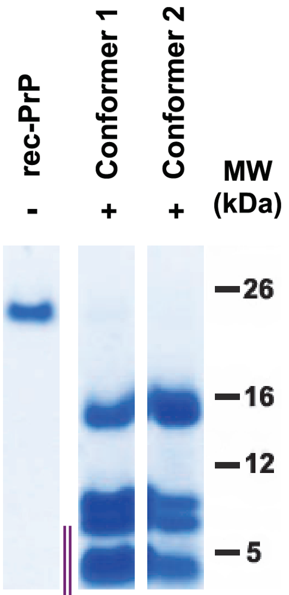

212 AA

2
Conformers

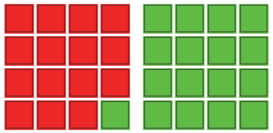

Tm[Exp]: 69.6 ºC

ΔΔG: -13.74

In vitro studies

TgVole (1x) Not tested

Nile lechwe Not tested

In vivo studies

TgVole (1x) Not tested

Nile lechwe Not tested

pLDDT
[85.79]

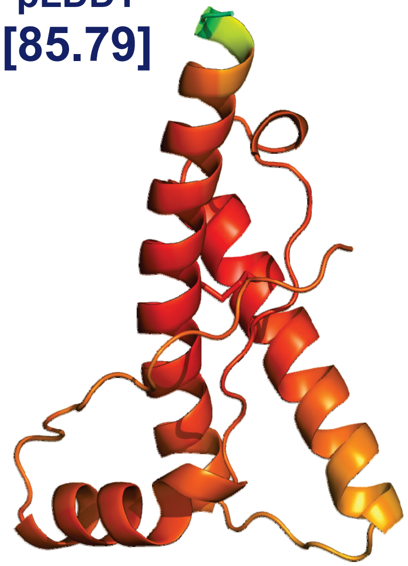

Protein code: 1448

Gerenuk

GenBank: BK064079

Litocranius walleri

Artiodactyla 46/50

Bovidae 26/30

P48%

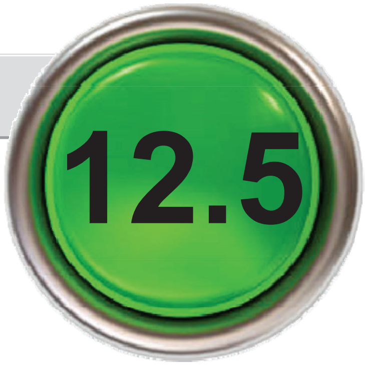

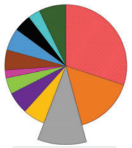

10-20

5 species sharing the same primary sequence

Harvey's red duiker
Cephalophus natalensis harveyi

Kirk's dik-dik
Madoqua kirkii

Suni
Neotragus moschatus

Klipspringer
Oreotragus oreotragus

Oribi
Ourebia ourebi

β2-α2 LOOP
SNQNNF
273

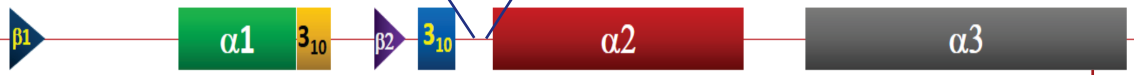

WNKPSKPKTNMKHVAGAAAAGAVVGGLGGYMLGSAMSRPLIHFGNDYEDRYYRENMYRYPNQVYYRPVDQYSNQNNFVHDCVNITVKQHTVTTTTKGENFTETDIKMMERVVEQMCITQYQRESQAYYQRGAS-

I M W N N I V V K EGRS

PrP sequence differs by 9 amino acids from the bank vole PrP (see in red)

No deletions

No insertions

No polymorphic variants found

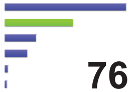

76

220 AAA

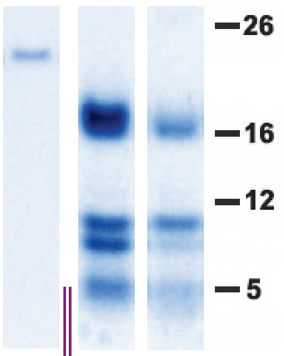

220 AA

2

Conformers

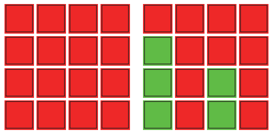

Tm^{Exp}: ND

ΔΔG: -13.08

In vitro studies

TgVole (1x) — Not tested

Gerenuk — Not tested

In vivo studies

TgVole (1x) — Not tested

Gerenuk — Not tested

pLDDT [86.3]

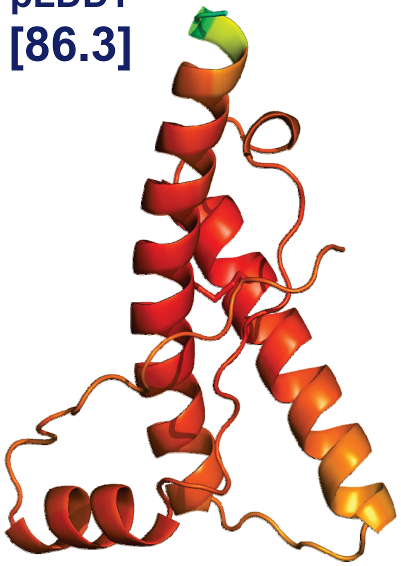

Protein code: 3257

Alpine musk deer

GenBank: AY723286

Moschus chrysogaster

Artiodactyla 46/50

Moschidae 2/2

P66%

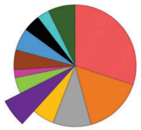

30-40

39.3

2 species sharing the same primary sequence

Dwarf musk deer
Moschus berezovskii

Saiga antelope
Saiga tatarica

β2-α2 LOOP
SNQNNF
273

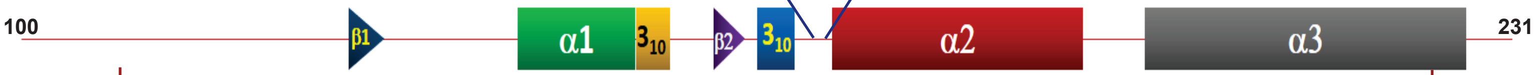

WNKPSKPKTNMKHVAGAAAAGAVVGGLGGYMLGSAMSRPLIHFGNDYEDRYYRENMYRYPNQVYYRPVDQYSNQNNFVHDCVNITVKQHTVTTTTKGENFTETDIKIMERVVEQMCITQYQRESQAYYQRGAS-

I M W N N I V M V K EGRS

PrP sequence differs by 10 amino acids from the bank vole PrP (see in red)

No deletions

No insertions

No polymorphic variants found

76

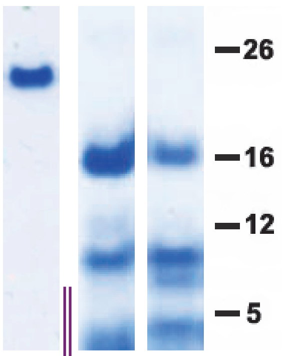

212 AA

2
Conformers

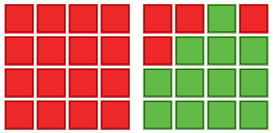

Tm[Exp]: ND

ΔΔG: -12.34

In vitro studies

TgVole (1x) Not tested

Alpine musk deer Not tested

In vivo studies

TgVole (1x) Not tested

Alpine musk deer Not tested

pLDDT
[85.61]

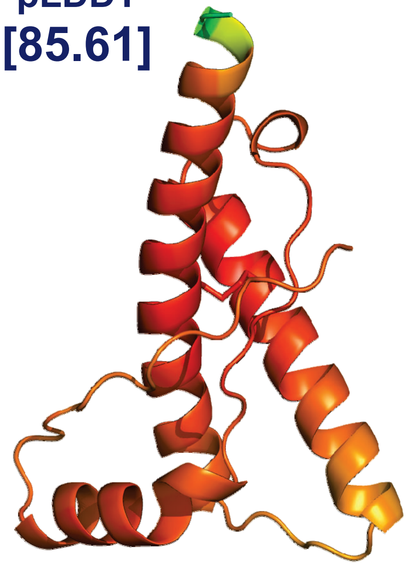

Protein code: 0920

Siberian musk deer

GenBank: BK063923

Moschus moschiferus

Artiodactyla 46/50
Moschidae 2/2

P76%

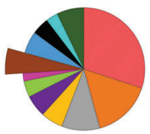

60-70

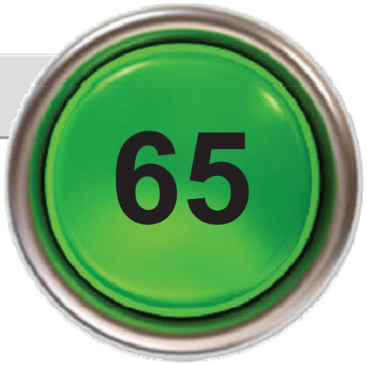

β2-α2 LOOP
SNQNNF
273

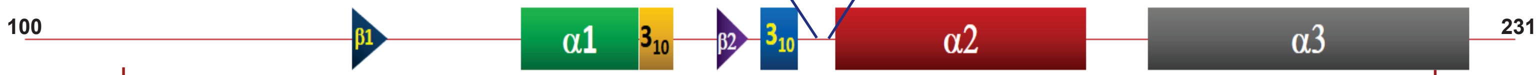

WNKPSKPKTNMKHVAGAAAAGAVVGGLGGYMLGSAMSRPLIHFGNDYEDRYYRENMYRYPDQVYYRPVDQYSNQNNFVHDCVNITVKQHTVTTTTKGENFTETDIKIMERVVEQMCITQYQRESQAYYQRGAS-

I M W N N N I V M V K EGRS

PrP sequence differs by 11 amino acids from the bank vole PrP (see in red)

No deletions

No insertions

No polymorphic variants found

Distinct primary sequence across species

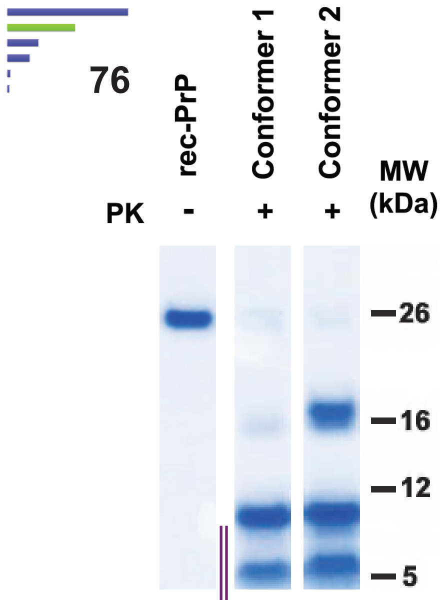

212 AA

2
Conformers

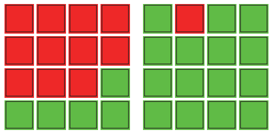

Tm^{Exp}: ND

ΔΔG: -11.59

In vitro studies

TgVole (1x) Not tested

Siberian musk deer Not tested

In vivo studies

TgVole (1x) Not tested

Siberian musk deer Not tested

pLDDT
[86.15]

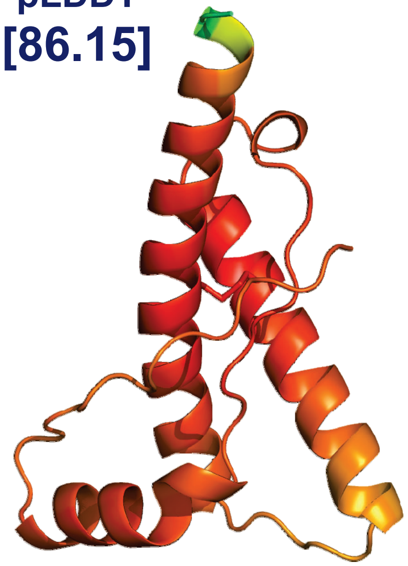

Protein code: 2032

Indian muntjac

GenBank: BK064098

Muntiacus muntjak

Artiodactyla 46/50
Cervidae 6/6

P82% **71.8**

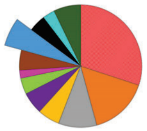

70-80

β2-α2 LOOP
NNQNTF
72

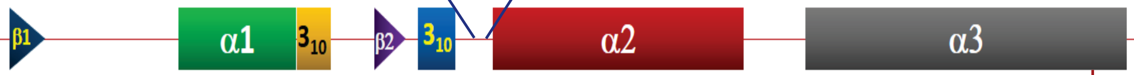

WNKPSKPKTNMKHVAGAAAAGAVVGGLGGYMLGSAMSRPLIHFGNDYEDRYYRENMYRYPNQVYYRPVDQYNNQNTFVHDCVNITVKQHTVTTTTKGENFTETDIKMMERVVEQMCITQYQRESEAYYQRGAS-
I M W N N I V V K Q EGRS

PrP sequence differs by 10 amino acids from the bank vole PrP (see in red)

No deletions
No insertions
No polymorphic variants found

Distinct primary sequence across species

135

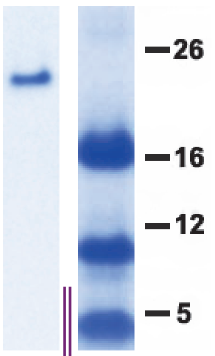

212 AA

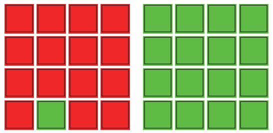

1
Conformers

Tm^{Exp}: ND

ΔΔG: -14.89

In vitro studies

TgVole (1x) Not tested

Indian muntjac Not tested

In vivo studies

TgVole (1x) Not tested

Indian muntjac Not tested

pLDDT
[89.05]

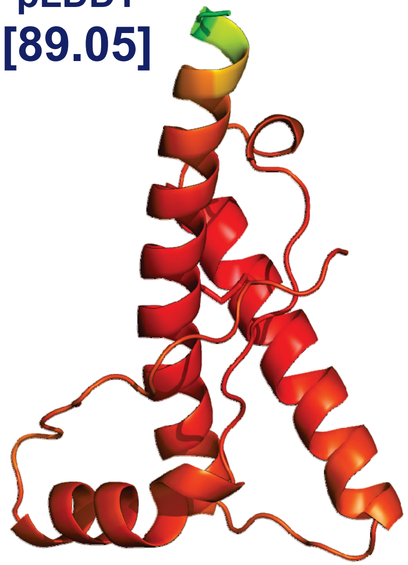

Protein code: 3277

Reeve's muntjac

GenBank: KC788406

Muntiacus reevesi

Artiodactyla 46/50
Cervidae 6/6

P92%

96.4

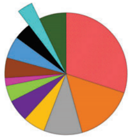

90-100

β2-α2 LOOP
NNQNTF
72

100 — β1 — α1 — 3_{10} — β2 — 3_{10} — α2 — α3 — 231

WNKPSKPKTNMKHVAGAAAAGAVVGGLGGYMLGSAMSRPLIHFGNDYEDRYYRENMYRYPNQVYYRPVDQYNNQNTFVHDCVNITVKQHTVTTTTKGENFTETDIKMMERVVEQMCITQYQRESQAYYQRGAS-

I M W N N I V V K EGRS

PrP sequence differs by 9 amino acids from the bank vole PrP (see in red)

No deletions
No insertions
No polymorphic variants found

2 species sharing the same primary sequence

Hairy-fronted muntjac
Muntiacus crinifrons

Gongshan muntjac
Muntiacus gongshanensis

135

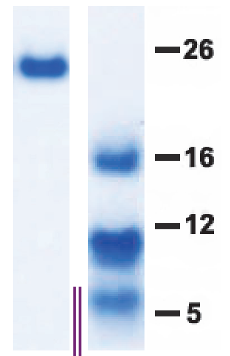

212 AA

1
Conformers

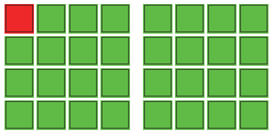

Tm^Exp: 68.1 ºC
ΔΔG: -13.83

In vitro studies

TgVole (1x) — Not tested
Reeve's muntjac — Not tested

In vivo studies

TgVole (1x) — Not tested
Reeve's muntjac — Not tested

pLDDT
[86.43]

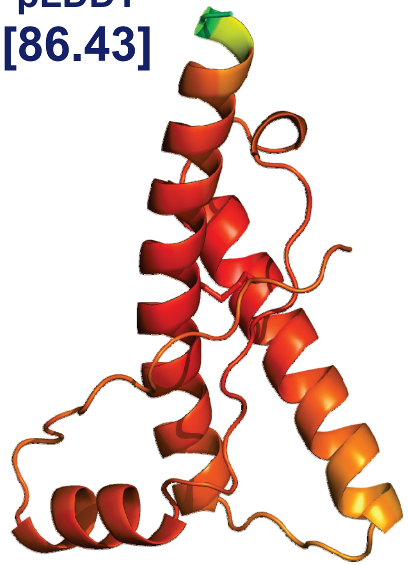

Protein code: 0950

Royal antelope

GenBank: BK064112

Neotragus pygmaeus

Artiodactyla 46/50

Bovidae 26/30

P100%

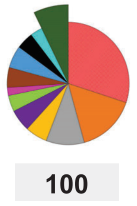

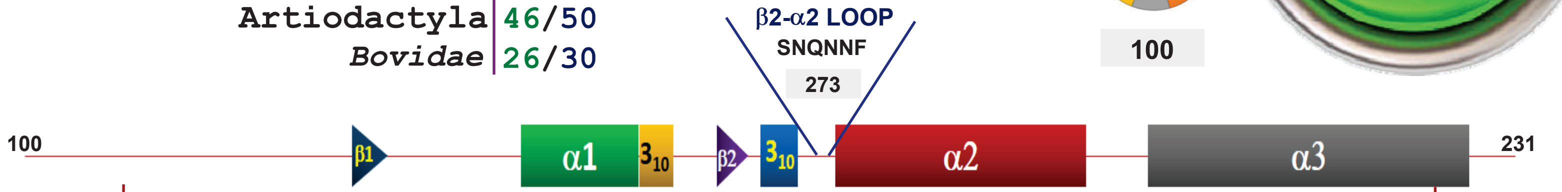

WNKPSKPKTSMKHVAGAAAAGAVVGGLGGYMLGSAMSRPLIHFGNDYEDRYYRENMYRYPNQVYYRPVDQYSNQNNFVHDCVNITVKQHTVTTTTKGENFTETDIKMMERVVEQMCITQYQRESQAYYQRGAS-

NI M W N N I V V K EGRS

PrP sequence differs by 10 amino acids from the bank vole PrP (see in red)

No deletions

No insertions

No polymorphic variants found

Distinct primary sequence across species

33

212 AA

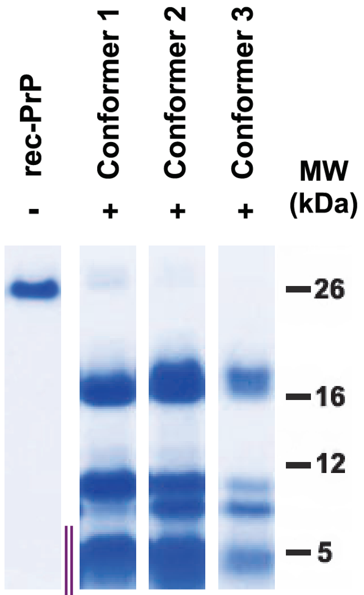

3 Conformers

Tm^{Exp}: ND

ΔΔG: -11.29

In vitro studies

TgVole (1x) Not tested

Royal antelope Not tested

In vivo studies

TgVole (1x) Not tested

Royal antelope Not tested

pLDDT [89.4]

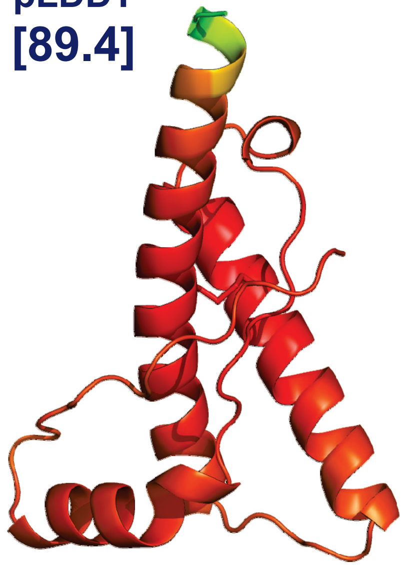

Protein code: 3292

Mule deer

GenBank: MT709729

Odocoileus hemionus

Artiodactyla 46/50
Cervidae 6/6

P100%

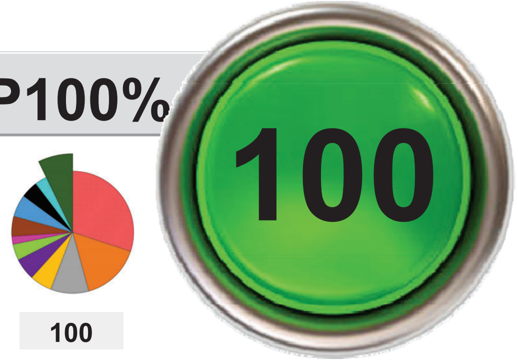

β2-α2 LOOP
NNQNTF
72

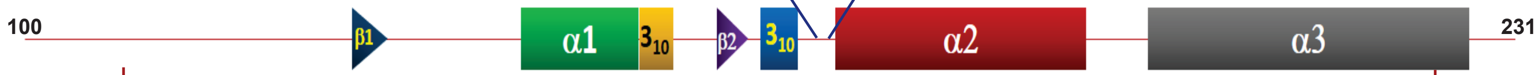

WNKPSKPKTNMKHVAGAAAAGAVVGGLGGYMLGSAMSRPLIHFGNDYEDRYYRENMYRYPNQVYYRPVDQYNNQNTFVHDCVNITVKQHTVTTTTKGENFTETDIKMMERVVEQMCITQYQRESQAYYQRGAS-
I M W N N I V V K EGRS

PrP sequence differs by 9 amino acids from the bank vole PrP (see in red)

No deletions
No insertions
It shows polymorphic variants

7 species sharing the same primary sequence

- **Moose** *Alces alces*
- **Roe deer** *Capreolus capreolus*
- **Siberian roe deer** *Capreolus pygargus*
- **Water deer** *Hydropotes inermis*
- **White-tailed deer** *Odocoileus virginianus*
- **Reindeer** *Rangifer tarandus*
- **Eld's deer** *Rucervus eldii thamin*

76

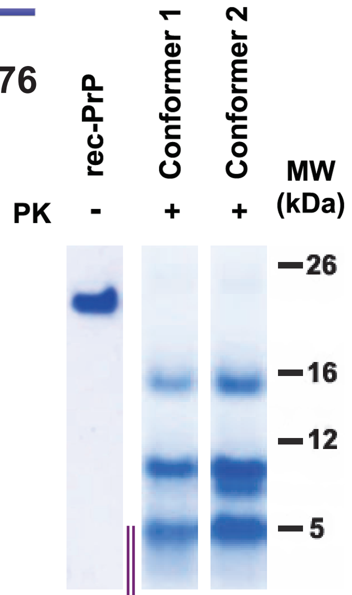

212 AA

2
Conformers

Tm^{Exp}: 67.7 ºC
ΔΔG: -21.62

In vitro studies

TgVole (1x)	Propagate
Mule deer	Propagate

In vivo studies

TgVole (1x)	Infectious
TgMule deer	Ongoing

pLDDT
[87.1]

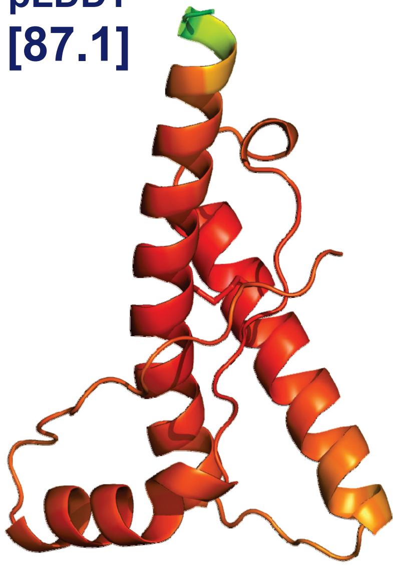

Protein code: 0174

Okapi

GenBank: BK064032

Okapia johnstoni

Artiodactyla 46/50

Giraffidae 2/2

P88%

87.1

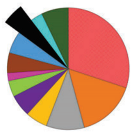

80-90

β2-α2 LOOP
SNQNTF
75

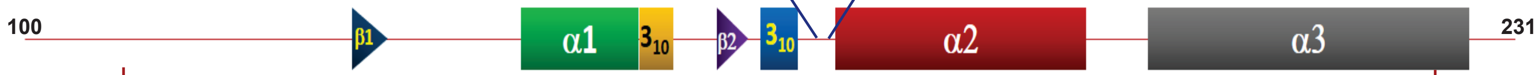

WNKPSKPKTNMKHVAGAAAAGAVVGGLGGYMLGSAMSRPLIHFGNDYEDRYYRENMYRYPNQVYYRPVDQYSNQNTFVHDCVNITVKQHTVTTTTKGENFTETDIKMMERVVEQMCITQYQRESEAYYQRGAS-

I M W N N N I V V K Q EGRS

PrP sequence differs by 11 amino acids from the bank vole PrP (see in red)

No deletions

No insertions

No polymorphic variants found

Distinct primary sequence across species

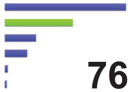

76

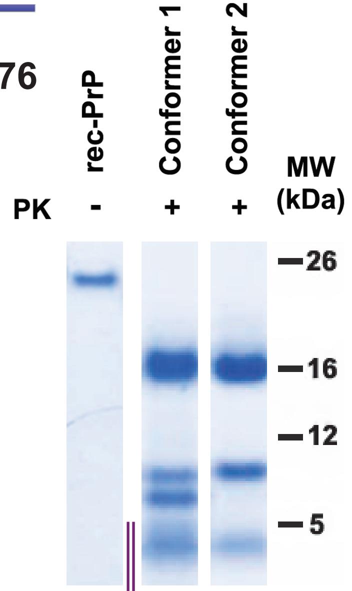

220 AA

2

Conformers

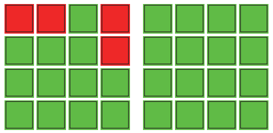

Tm[Exp]: ND

ΔΔG: -12.63

In vitro studies

TgVole (1x) Not tested

Okapi Not tested

In vivo studies

TgVole (1x) Not tested

Okapi Not tested

pLDDT

[86.56]

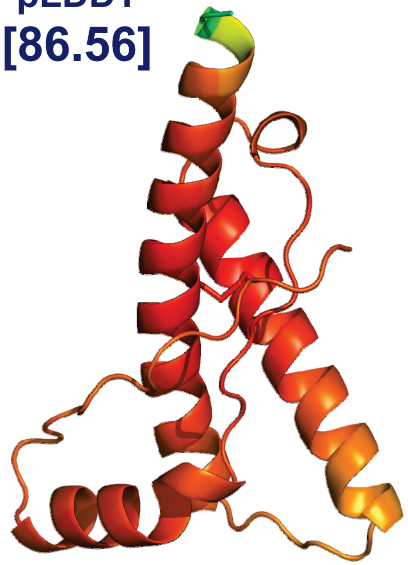

Protein code: 3197

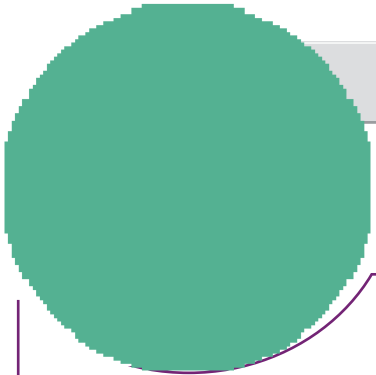

Gemsbok

GenBank: BK064117

Oryx gazella

Artiodactyla 46/50

Bovidae 26/30

P83%

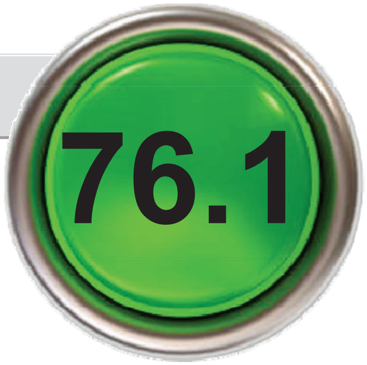

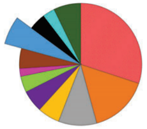

70-80

β2-α2 LOOP
SNQNNF
273

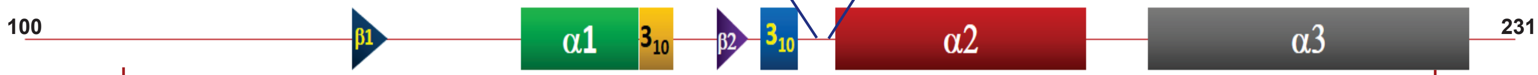

WNKPSKPKTNMKHVAGAAAAGAVVGGLGGYMLGSAMSRPLIHFGNDYEDRYYRENMYRYPNQVYYRPVDQYSNQNNFVHDCVNITVKQHTVTTTTKGENFTETDIKIMERVVEQMCITQYQKESQAYYQRGAS-

I M W N N I V M V EGRS

PrP sequence differs by 9 amino acids from the bank vole PrP (see in red)

No deletions

No insertions

No polymorphic variants found

Distinct primary sequence across species

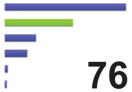

76

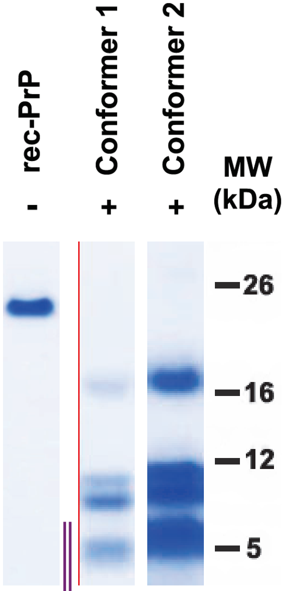

212 AA

2 Conformers

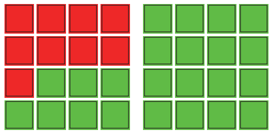

Tm[Exp]: ND

ΔΔG: -11.01

In vitro studies

TgVole (1x) Not tested

Gemsbok Not tested

In vivo studies

TgVole (1x) Not tested

Gemsbok Not tested

pLDDT [85.73]

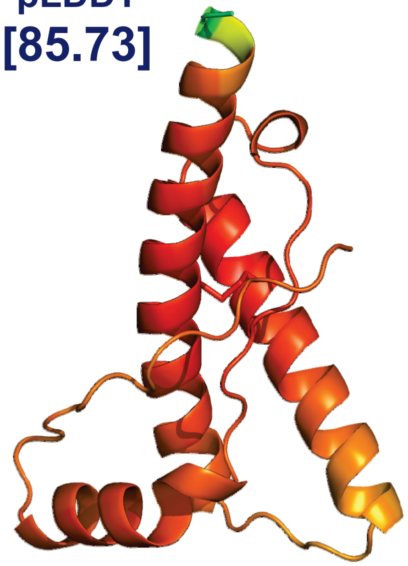

Protein code: 3297

Sheep

GenBank: NM_001009481

Ovis aries

Artiodactyla 46/50

Bovidae 26/30

P73%

54.3

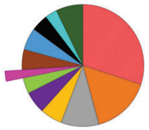

50-60

β2-α2 LOOP
SNQNNF
273

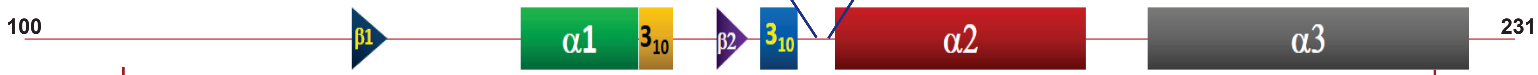

WNKPSKPKTNMKHVAGAAAAGAVVGGLGGYMLGSAMSRPLIHFGNDYEDRYYRENMYRYPNQVYYRPVDQYSNQNNFVHDCVNITVKQHTVTTTTKGENFTETDIKIMERVVEQMCITQYQRESQAYYQRGAS-

I M W N N I V M V K EGRS

PrP sequence differs by 10 amino acids from the bank vole PrP (see in red)

No deletions

No insertions

It shows polymorphic variants

18 species sharing the same primary sequence

- **Goat** *Capra hircus*
- **Dall sheep** *Ovis dalli*
- **Muskox** *Ovibos moschatus*
- **Alpine ibex** *Capra ibex*
- **Markhor** *Capra falconeri*
- **Iberian ibex** *Capra pyrenaica*
- **Barbary sheep** *Ammotragus lervia*
- **Pyrenean chamois** *Rupicapra pyrenaica*
- **Arabian tahr** *Hemitragus jayakari*
- **Japanese serow** *Capricornis crispus*
- **Mountain goat** *Oreamnos americanus*
- **Chinese goral** *Naemorhedus griseus*
- **Siberian ibex** *Capra sibirica*
- **Tibetan antelope** *Pantholops hodgsonii*
- **Nilgiri tahr** *Hemitragus hylocrius*
- **Argali** *Ovis ammon*
- **Snow sheep** *Ovis nivicola lydekkeri*
- **Bharal** *Pseudois nayaur*

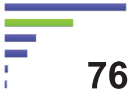

76

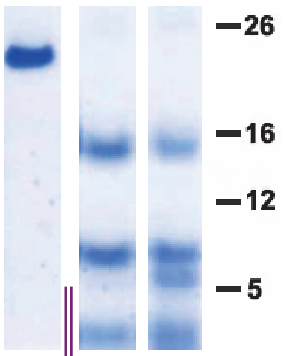

212 AA

2

Conformers

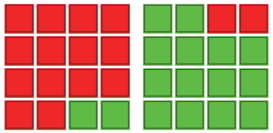

Tm^{Exp}: 71.7 ºC

ΔΔG: -11.89

pLDDT [87.26]

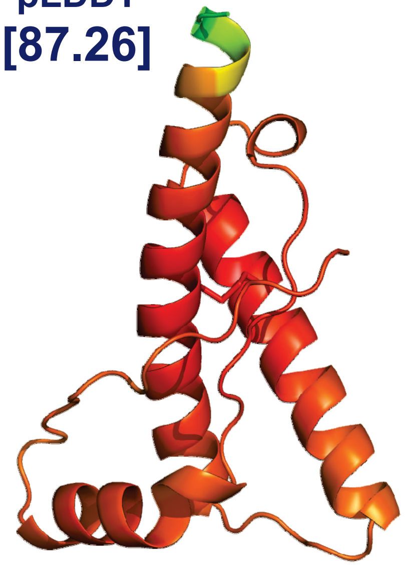

In vitro studies

TgVole (1x)	Propagate
TgSheep	Propagate

In vivo studies

TgVole (1x)	Infectious
TgSheep	Infectious

Protein code: 0195

Bighorn sheep

GenBank: DQ648476

Ovis canadensis

Artiodactyla 46/50

Bovidae 26/30

Distinct primary sequence across species

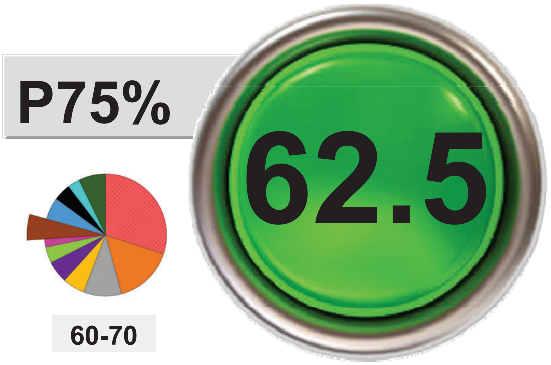

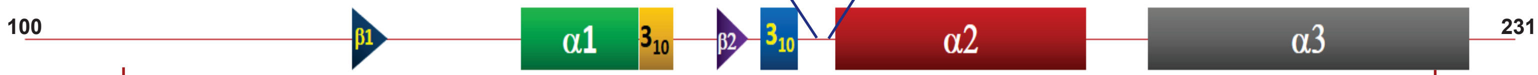

WNKPSKPKTNMKHVAGAAAAGAVVGGLGGYMLGSAMSRPLIHFGNDYEDRYYRENMYRYPNQVYYRPVDQYSNQNNFVHDCVNITVKQHTVTTTTKGENFTETDIKIMGRVVEQMCITQYQRESQAYYQRGAS-

I M W N N I V M E V K EGRS

PrP sequence differs by 11 amino acids from the bank vole PrP (see in red)

No deletions

No insertions

It shows polymorphic variants

Tm^{Exp}: ND

ΔΔG: -9.13

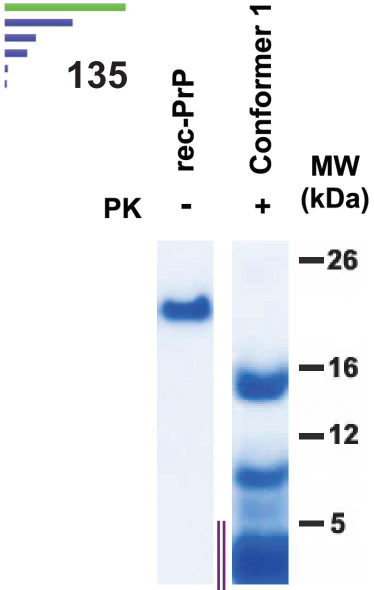

212 AA

1

Conformers

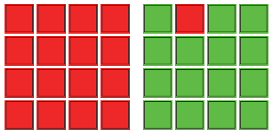

In vitro studies

TgVole (1x) Not tested

Bighorn sheep Not tested

In vivo studies

TgVole (1x) Not tested

Bighorn sheep Not tested

pLDDT [89.23]

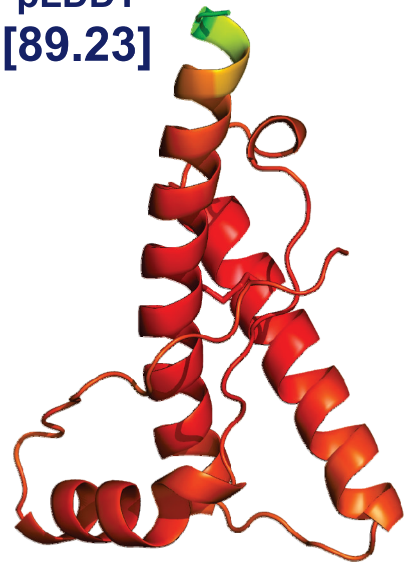

Protein code: 1478

Common warthog

GenBank: BK063968

P71%

48.6

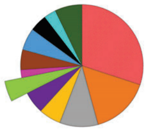

40-50

Phacochoerus africanus

Artiodactyla 46/50

Suidae 3/3

1 species sharing the same primary sequence

Red river hog
Potamochoerus porcus

β2-α2 LOOP
SNQNSF
36

100 — β1 — α1 — 3_{10} — β2 — 3_{10} — α2 — α3 — 231

WNKPSKPKTNMKHVAGAAAAGAVVGGLGGYMLGSAMNRPLIHFGSDYEDRYYRENMYRYPNQVYYRPVDQYSNQNSFVHDCVNITVKQHTVTTTTKGENFTETDVKMIERVVEQMCITQYQKEYEAYAQRGAS-

I S M N W N N N I M V SQ YEGRS

PrP sequence differs by 13 amino acids from the bank vole PrP (see in red)

No deletions

No insertions

No polymorphic variants found

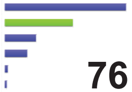

76

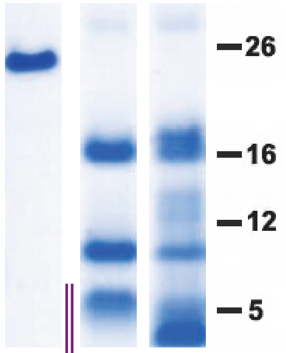

213 AA

2
Conformers

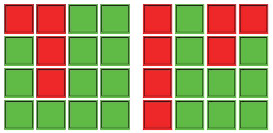

Tm^{Exp}: ND

ΔΔG: -13.67

In vitro studies

TgVole (1x) — Not tested

Common warthog — Not tested

In vivo studies

TgVole (1x) — Not tested

Common warthog — Not tested

pLDDT
[84.9]

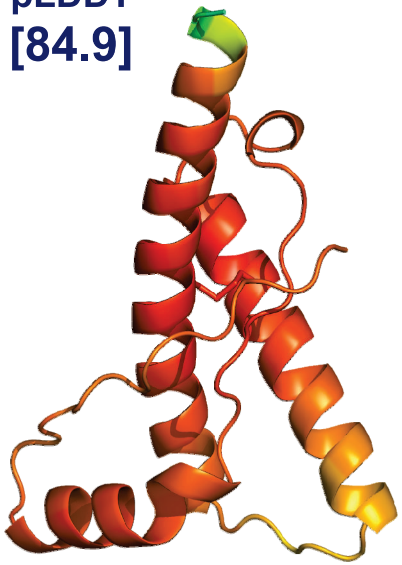

Protein code: 3101

Maxwell's duiker

GenBank: BK064126

Philantomba maxwellii

Artiodactyla 46/50

Bovidae 26/30

P81%

71.4

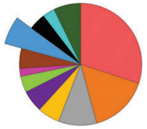

70-80

β2-α2 LOOP
SNQNNF
273

100 β1 α1 3_{10} β2 3_{10} α2 α3 231

WNKPSKPKTNMKHVAGAAAAGAVVGGLGGYMLGSAMSRPLIHFGNDYEDRYYRENMYRYPNQVYYRPVDQYSNQNNFVHDCVNITVKQHTVTTTTKGENFTETDIKMMERVVEQMCITQYQRESQAYYQRGAS-

I M W N N I V V K EGRS

PrP sequence differs by 9 amino acids from the bank vole PrP (see in red)

No deletions

No insertions

No polymorphic variants found

Distinct primary sequence across species

76

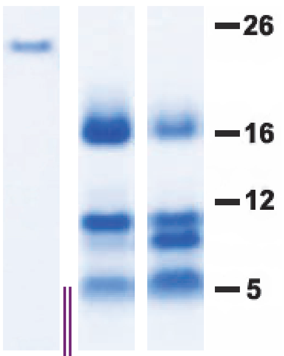

220 AA

2 Conformers

TmExp: ND

ΔΔG: -11.79

In vitro studies

TgVole (1x) Not tested

Maxwell's duiker Not tested

In vivo studies

TgVole (1x) Not tested

Maxwell's duiker Not tested

pLDDT [86.96]

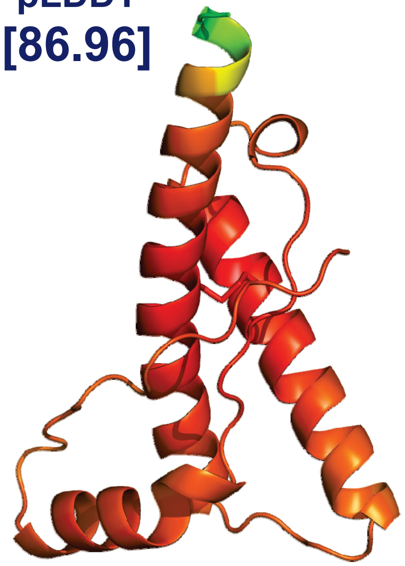

Protein code: 3307

Reindeer

GenBank: MW804583

Rangifer tarandus

Artiodactyla 46/50

Cervidae 6/6

P89%

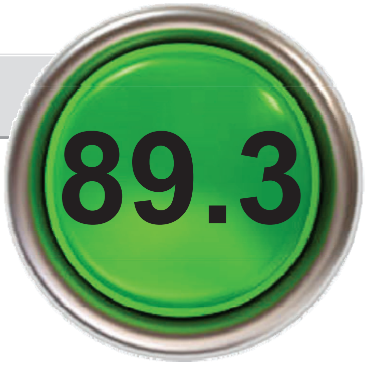

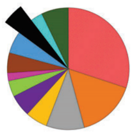

80-90

β2-α2 LOOP
NNQNTF
72

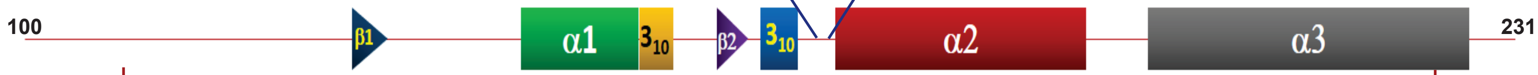

WNKPSKPKTNMKHVAGAAAAGAVVGGLGGYMLGSAMNRPLIHFGNDYEDRYYRENMYRYPNQVYYRPVDQYNNQNTFVHDCVNITVKQHTVTTTTKGENFTETDIKMMERVVEQMCITQYQRESQAYYQRGAS-

I S M W N N I V V K EGRS

PrP sequence differs by 10 amino acids from the bank vole PrP (see in red)

No deletions

No insertions

It shows polymorphic variants

Distinct primary sequence across species

135

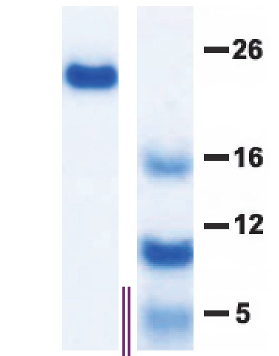

212 AA

1

Conformers

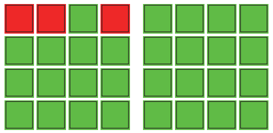

Tm^{Exp}: ND

ΔΔG: -9.46

In vitro studies

TgVole (1x) Not tested

Père David's deer Not tested

In vivo studies

TgVole (1x) Not tested

Père David's deer Not tested

pLDDT

[88.94]

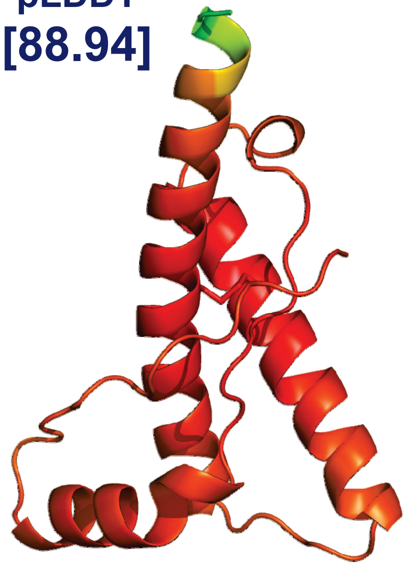

Protein code: 1450

Steenbok

GenBank: BK064134

Raphicerus campestris

Artiodactyla	46/50
Bovidae	26/30

P89%

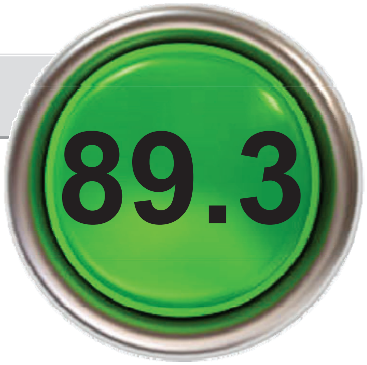

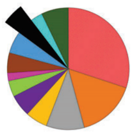

80-90

β2-α2 LOOP
SNQNNF
273

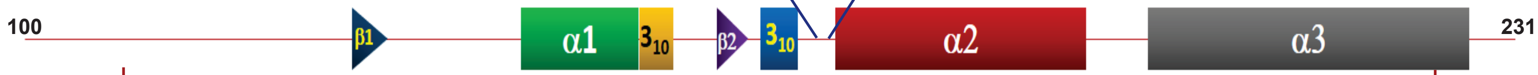


```
WNKPSKPKTSMKHVAGAAAAGAVVGGLGGYMLGSAMSRPLIHFGNDYEDRYYRENMYRYPNQVYYRPVDQYSNQNNFVHDCVNITVKQHTVTTTTKGENFTETDIKMMERVVEQMCITQYQRESQAYYQRGAS-
         NI                              M     W        N             N            I                V          V   K      EGRS
```

PrP sequence differs by 10 amino acids from the bank vole PrP (see in red)

No deletions
No insertions
No polymorphic variants found

Distinct primary sequence across species

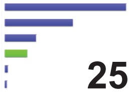

25

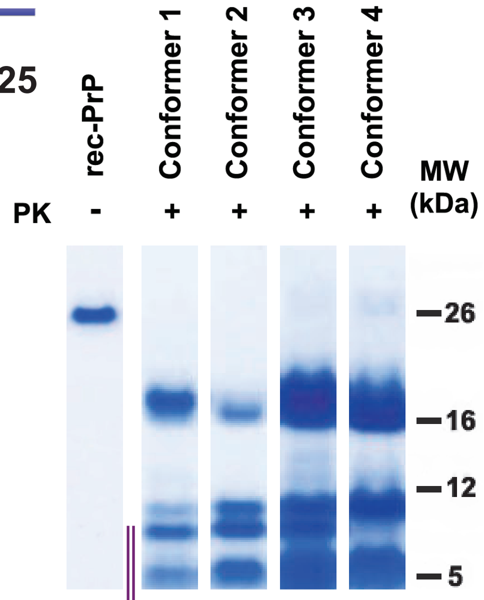

212 AA

4
Conformers

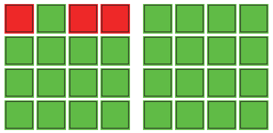

Tm^{Exp}: ND

ΔΔG: -11.02

In vitro studies

TgVole (1x) Not tested

Steenbok Not tested

In vivo studies

TgVole (1x) Not tested

Steenbok Not tested

pLDDT
[86.41]

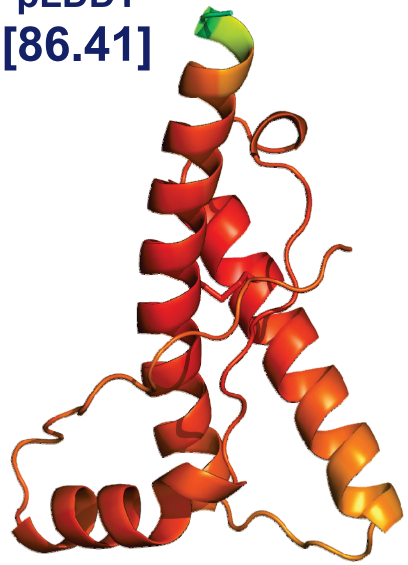

Protein code: 3318

Bohor reedbuck

GenBank: BK064135

Redunca redunca

Artiodactyla	46/50
Bovidae	26/30

P29%

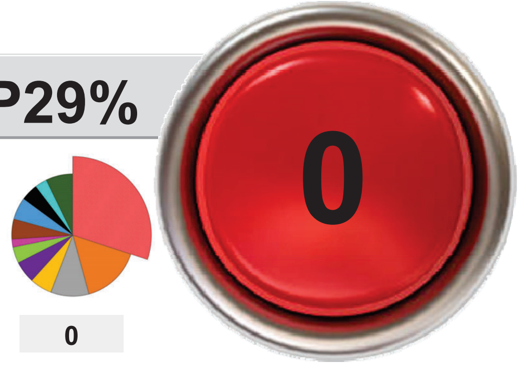

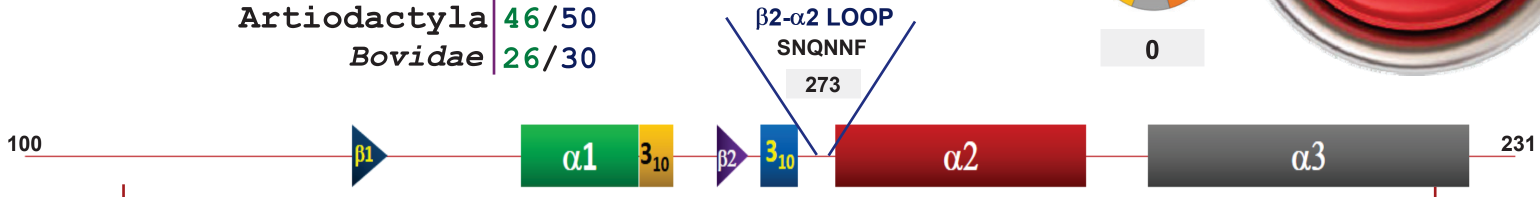

WNKPSKPKTNMKHVAGAAAAGAVVGGLGGYMLGSAMSRPLIHFGNDYEDRYYRENMYRYPNQVYYRPVDQYSNQNNFVHDCVNITVKQHTVTTTTKGENFTETDIKMMERVVEQMCITQYQRESEAYYQRGAS-

I M W N N I V V K Q EGRS

PrP sequence differs by 10 amino acids from the bank vole PrP (see in red)

No deletions

No insertions

No polymorphic variants found

Distinct primary sequence across species

Tm^{Exp}: ND

ΔΔG: -14.8

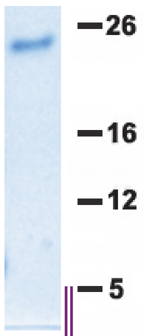

220 AA

NO MISFOLDING

0 Conformers

In vitro studies

NOT APPLICABLE

In vivo studies

NOT APPLICABLE

pLDDT [87.75]

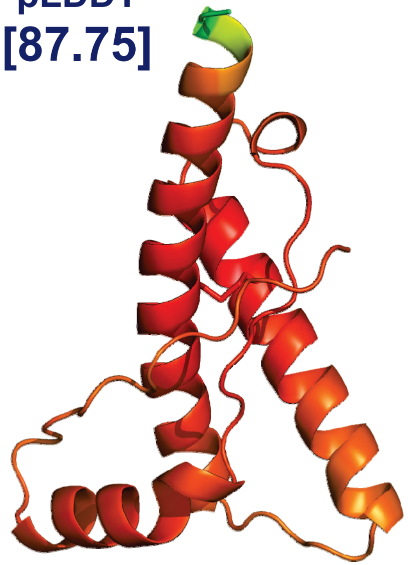

Protein code: 3319

Pig

GenBank: L07623

P78%

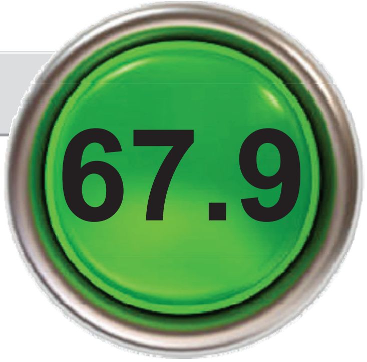

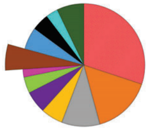

60-70

Sus scrofa

Artiodactyla 46/50

Suidae 3/3

β2-α2 LOOP

SNQNSF

36

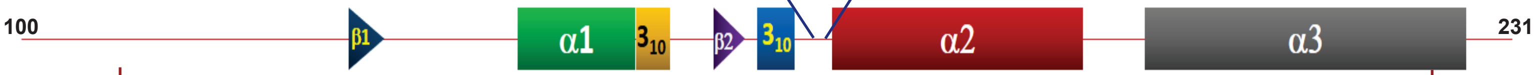

WNKPSKPKTNMKHVAGAAAAGAVVGGLGGYMLGSAMSRPLIHFGSDYEDRYYRENMYRYPNQVYYRPVDQYSNQNSFVHDCVNITVKQHTVTTTTKGENFTETDVKMIERVVEQMCITQYQKEYEAYAQRGAS-

I M N W N N N I M V SQ YEGRS

PrP sequence differs by 12 amino acids from the bank vole PrP (see in red)

No deletions

No insertions

No polymorphic variants found

33

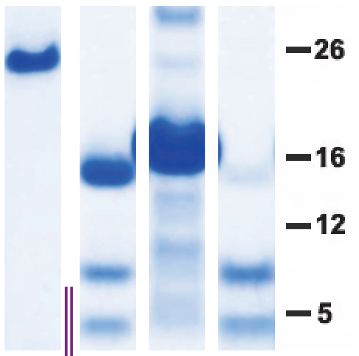

213 AA

3

Conformers

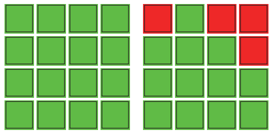

Tm^{Exp}: ND

ΔΔG: -15.44

In vitro studies

TgVole (1x) — Propagate

Pig — Ongoing

In vivo studies

TgVole (1x) — Infectious

TgPig — Not tested

pLDDT [85.4]

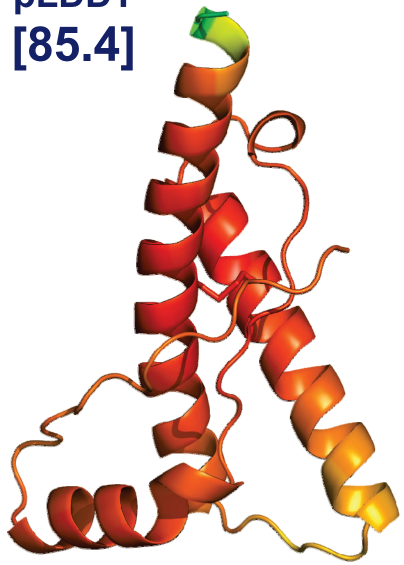

Distinct primary sequence across species

Protein code: 0243

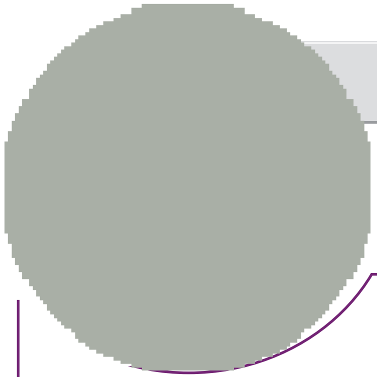

Common duiker

GenBank: BK064038

P58%

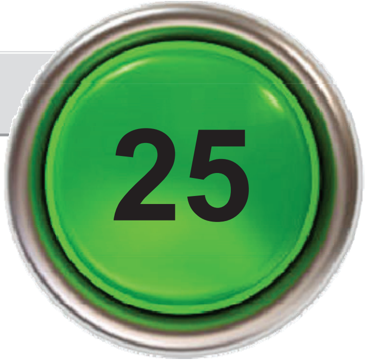

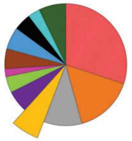

20-30

Sylvicapra grimmia

Artiodactyla 46/50

Bovidae 26/30

β2-α2 LOOP
SNQNNF
273

100 — β1 — α1 — 3_{10} — β2 — 3_{10} — α2 — α3 — 231

WNKPSKPKTNMKHMAGAAAAGAVVGGLGGYMLGSAMSRPLIHFGNDYEDRYYRENMYRYPNQVYYRPVDQYSNQNNFVHDCVNITVKQHTVTTTTKGENFTETDIKMMERVVEQMCITQYQRESQAYYQRGAS-

I V M W N N I V V K EGRS

PrP sequence differs by 10 amino acids from the bank vole PrP (see in red)

No deletions

No insertions

No polymorphic variants found

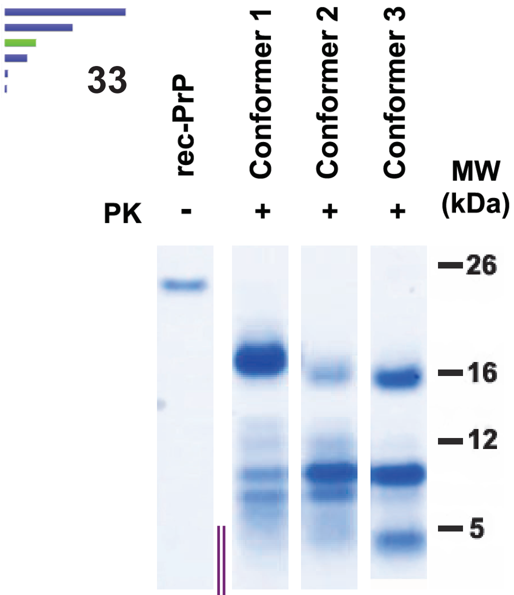

220 AA

3 Conformers

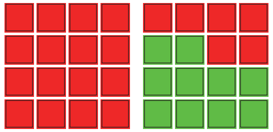

Tm[Exp]: ND

ΔΔG: -12.74

In vitro studies

TgVole (1x) Not tested

Common duiker Not tested

In vivo studies

TgVole (1x) Not tested

Common duiker Not tested

pLDDT [88.84]

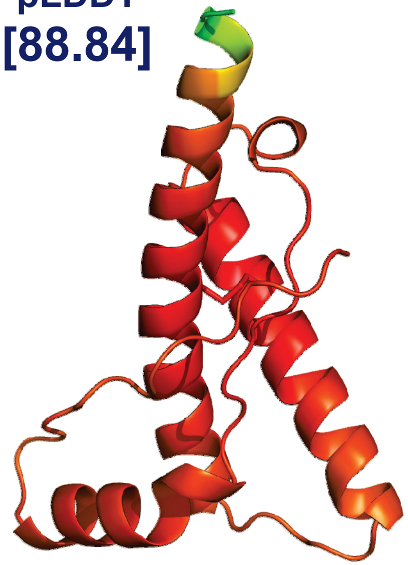

Distinct primary sequence across species

Protein code: 3204

African buffalo

GenBank: AY720686

Syncerus caffer

Artiodactyla 46/50

Bovidae 26/30

P83%

75

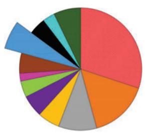

70-80

β2-α2 LOOP
SNQNNF
273

100

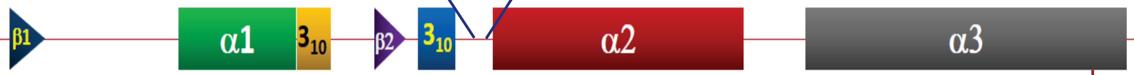

231

WNKPSKPKTNMKHVAGAAAAGAVVGGLGGYMLGSAMSRPLIHFGNDYEDRYYRENMHRYPNQVYYRPVDQYSNQNNFVHDCVNITVKEHTVTTTTKGENFTETDVKMMERVVEQMCITQYQRESEAYYQRGAS-

I M W N N I Q V K Q EGRS

PrP sequence differs by 10 amino acids from the bank vole PrP (see in red)

No deletions

No insertions

No polymorphic variants found

Distinct primary sequence across species

135

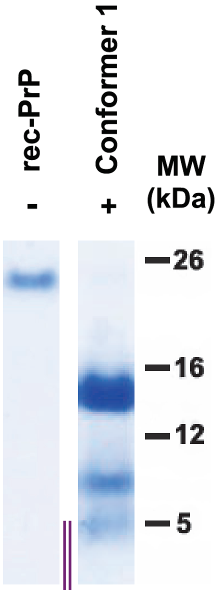

220 AA

1
Conformers

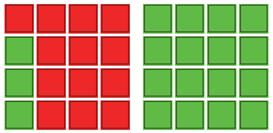

Tm^{Exp}: ND

ΔΔG: -10.06

In vitro studies

TgVole (1x) Not tested

African buffalo Not tested

In vivo studies

TgVole (1x) Not tested

African buffalo Not tested

pLDDT
[89.22]

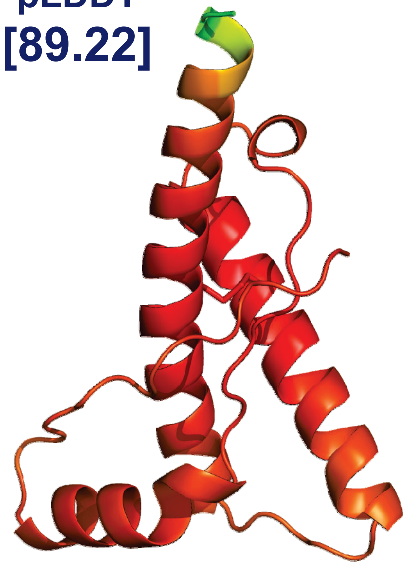

Protein code: 1434

Common eland

GenBank: EF165082

Taurotragus oryx

Artiodactyla 46/50
Bovidae 26/30

P81%

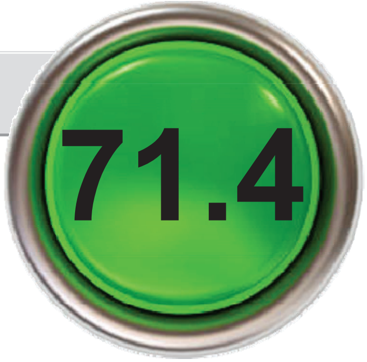

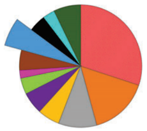

70-80

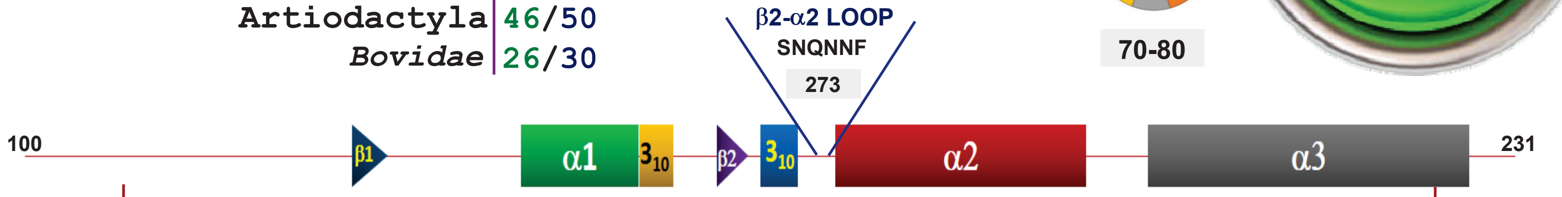

WNKPSKPKTNMKHVAGAAAAGAVVGGLGGYMLGSAMSRPLIHFGSDYEDRYYRENMYRYPNQVYYRPVDQYSNQNNFVHDCVNITVKQHTVTTTTKGENFTETDIKMMERVVEQMCITQYQRESEAYYQRGAS-

I M N W N N I V V K Q EGRS

PrP sequence differs by 11 amino acids from the bank vole PrP (see in red)

No deletions
No insertions
No polymorphic variants found

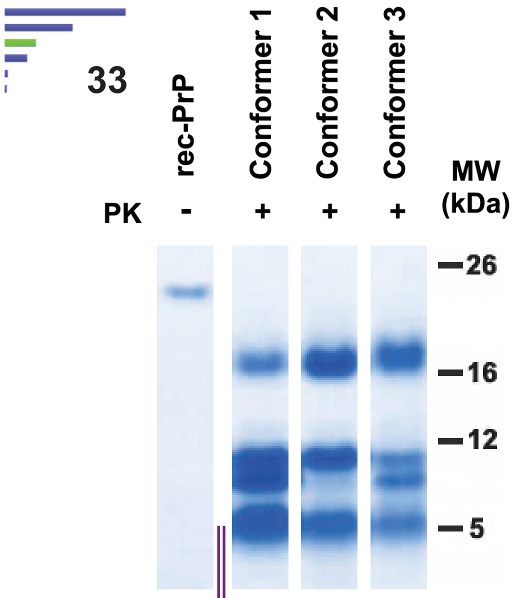

220 AA

3 Conformers

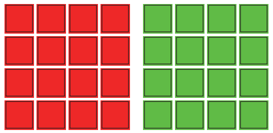

Tm[Exp]: ND

ΔΔG: -24.41

In vitro studies

TgVole (1x) Not tested

Common eland Not tested

In vivo studies

TgVole (1x) Not tested

Common eland Not tested

pLDDT [92.23]

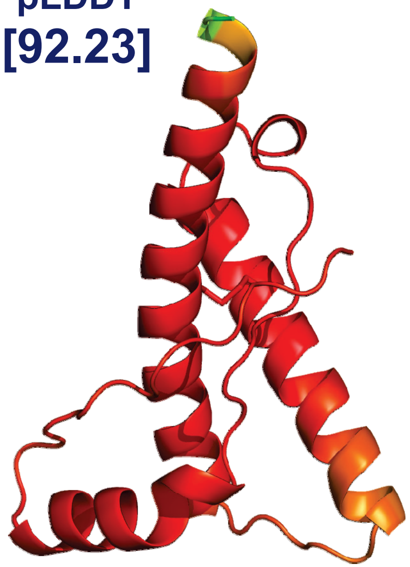

Distinct primary sequence across species

Protein code: 1379

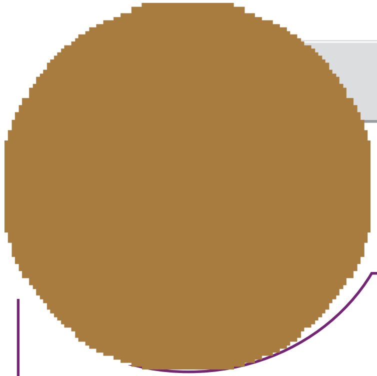

Lowland nyala

GenBank: EU032296

Tragelaphus angasii

Artiodactyla 46/50
Bovidae 26/30

P90%

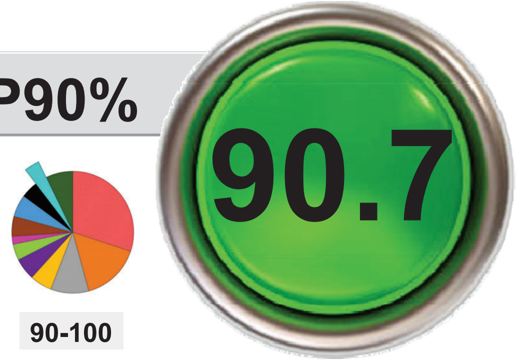

β2-α2 LOOP
SNQNNF
273

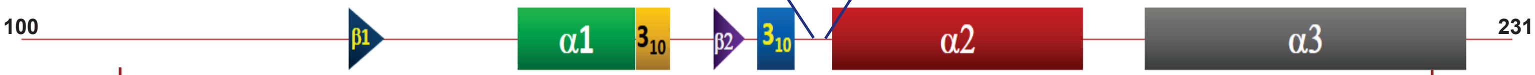

WNKPSKPKTNMKHVAGAAAAGAVVGGLGGYMLGSAMSRPLIHFGSDYEDRYYRENMYRYPNQVYYRPVDQYSNQNNFVHDCVNITVKQHTVTTTTKGENFTETDIKMMERVVEQMCITQYQRESEAYYQRGAS-

I M N W N N I V V K Q EGRS

PrP sequence differs by 11 amino acids from the bank vole PrP (see in red)

No deletions
No insertions
No polymorphic variants found

Distinct primary sequence across species

33

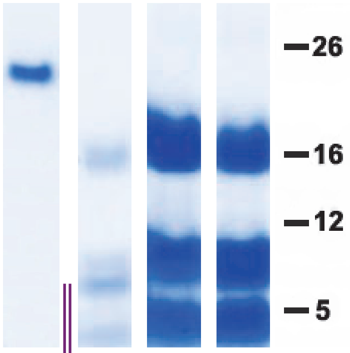

212 AA

3
Conformers

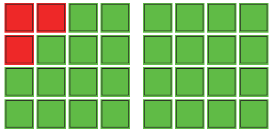

Tm^{Exp}: ND

ΔΔG: -10.1

In vitro studies

TgVole (1x) Not tested

Lowland nyala Not tested

In vivo studies

TgVole (1x) Not tested

Lowland nyala Not tested

pLDDT
[86.58]

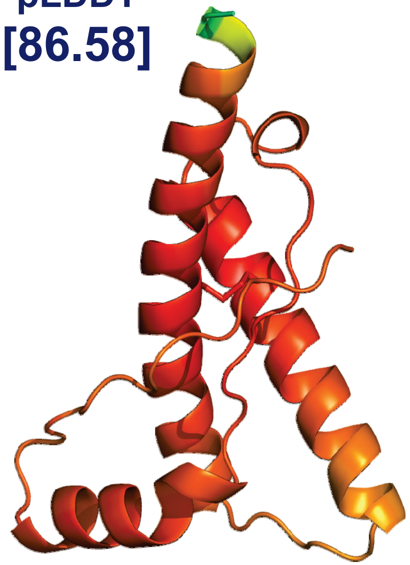

Protein code: 1327

Mountain nyala

GenBank: BK064065

Tragelaphus buxtoni

Artiodactyla 46/50
Bovidae 26/30

P29%

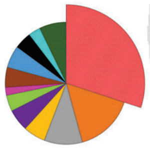

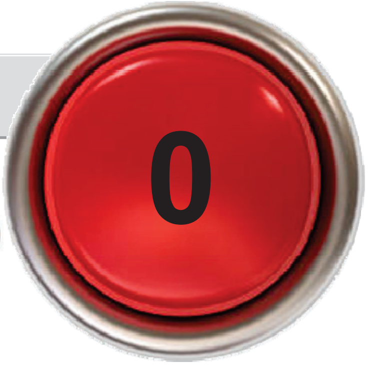

0

β2-α2 LOOP
SNQNNF
273

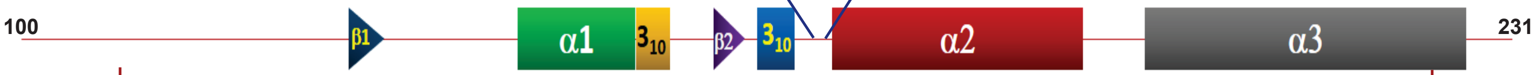

WNKPNKPKTNMKHVAGAAAAGAVVGGPGGYLLGSAMSRPLIHFGNDYEDRYYRENMYRYPNQVYYGPVDQFSNQNNFVHDCVNITVKQHTVTTTTKGENFTETDIKIMERVVEQMCITQYQRESEAYYQRGAS-

S I L M M W N R YN I V M V K Q EGRS

PrP sequence differs by 15 amino acids from the bank vole PrP (see in red)

No deletions
No insertions
No polymorphic variants found

Distinct primary sequence across species

Tm^{Exp}: ND

ΔΔG: -13.61

PK
rec-PrP
-
MW (kDa)

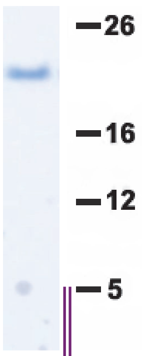

212 AA

NO MISFOLDING

0
Conformers

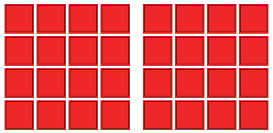

In vitro studies

NOT APPLICABLE

In vivo studies

NOT APPLICABLE

pLDDT
[87.79]

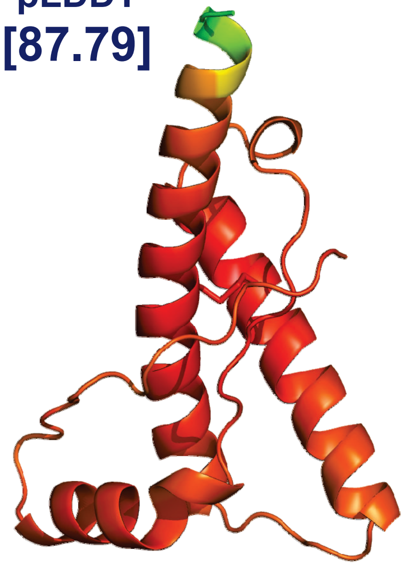

Protein code: 3241

Lesser kudu

GenBank: AY720704

P69%

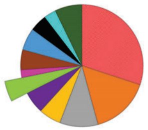

40-50

44.6

Tragelaphus imberbis

Artiodactyla 46/50

Bovidae 26/30

β2-α2 LOOP
SNQNNF
273

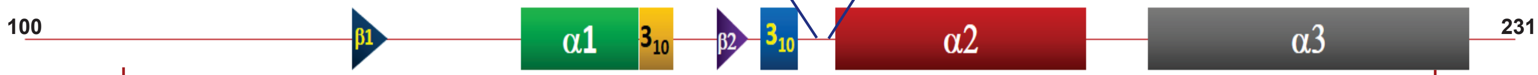

WNKPSKPKTNMKHVAGAAAAGAVVGGLGGYMLGSAMSRPLIHFGNDYEDRYYRENMYRYPNQVYYRPVDQYSNQNNFVHDCVNITVKQHTVTTTTKGENFTETDIKMMERVVEQMCITQYQRESEAYYQRGAS-

I M W N N I V V K Q EGRS

PrP sequence differs by 10 amino acids from the bank vole PrP (see in red)

No deletions

No insertions

No polymorphic variants found

Distinct primary sequence across species

76

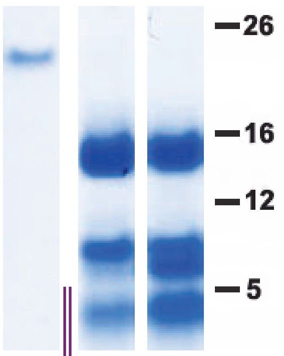

220 AA

2 Conformers

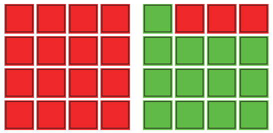

Tm^{Exp}: ND

ΔΔG: -12.32

In vitro studies

TgVole (1x) Not tested

Lesser kudu Not tested

In vivo studies

TgVole (1x) Not tested

Lesser kudu Not tested

pLDDT [86.01]

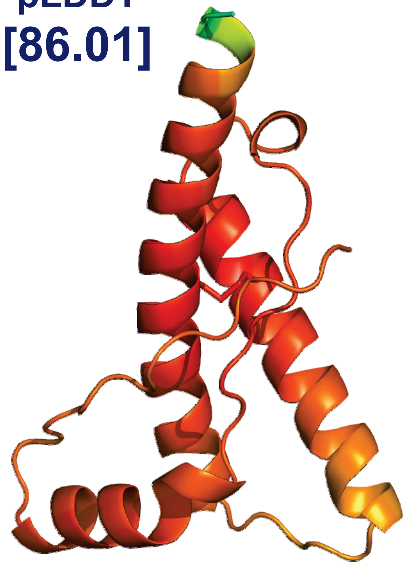

Protein code: 1380

Harnessed bushbuck

GenBank: BK063937

Tragelaphus scriptus

Artiodactyla 46/50
Bovidae 26/30

P66%

36.8

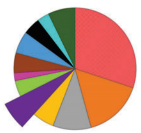

30-40

β2-α2 LOOP
SNQNNF
273

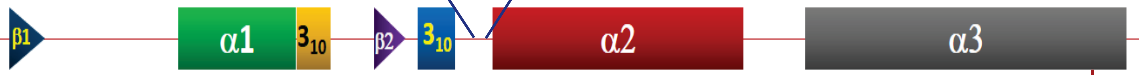

WNKPNKPKTNMKHVAGAAAAGAVVGGLGGYMLGSAMSRPLIHFGNDYEDRYYRENMYRYPNQVYYRPVDQYSNQNNFVHDCVNITVKQHTVTTTTKGENFTETDIKIMERVVEQMCITQYQRESEAYYQRGAS-
S I M W N N I V M V K Q EGRS

PrP sequence differs by 11 amino acids from the bank vole PrP (see in red)

No deletions
No insertions
No polymorphic variants found

Distinct primary sequence across species

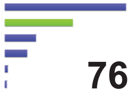

76

212 AA

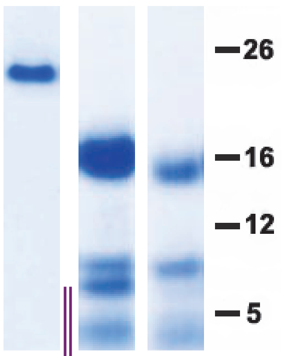

2
Conformers

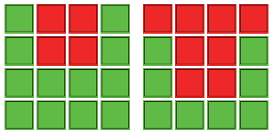

Tm^{Exp}: ND

ΔΔG: -15.56

In vitro studies

TgVole (1x) Not tested

Harnessed bushbuck Not tested

In vivo studies

TgVole (1x) Not tested

Harnessed bushbuck Not tested

pLDDT
[87.56]

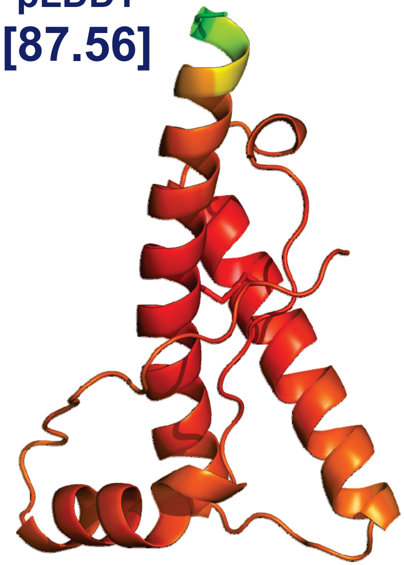

Protein code: 3060

Sitatunga

GenBank: EF165083

Tragelaphus spekii

Artiodactyla 46/50

Bovidae 26/30

1 species sharing the same primary sequence

Bongo
Tragelaphus eurycerus

P83%

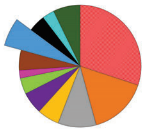

70-80

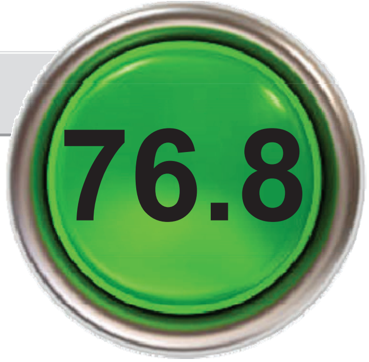

β2-α2 LOOP
SNQNNF
273

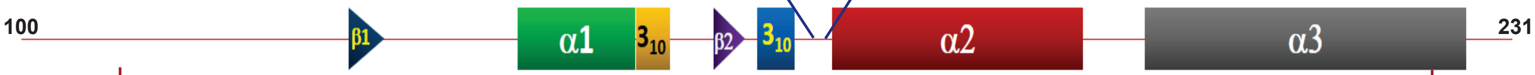

WNKPNKPKTNMKHVAGAAAAGAVVGGLGGYMLGSAMSRPLIHFGSDYEDRYYRENMYRYPNQVYYRPVDQYSNQNNFVHDCVNITVKQHTVTTTTKGENFTETDIKMMERVVEQMCITQYQRESEAYYQRGAS-

S I M N W N N I V V K Q EGRS

PrP sequence differs by 11 amino acids from the bank vole PrP (see in red)

No deletions

No insertions

No polymorphic variants found

25

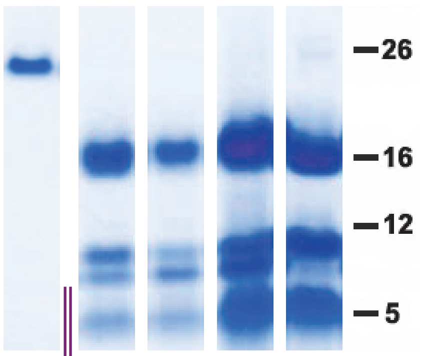

212 AA

4
Conformers

Tm^{Exp}: ND

ΔΔG: -10.2

In vitro studies

TgVole (1x) Not tested

Sitatunga Not tested

In vivo studies

TgVole (1x) Not tested

Sitatunga Not tested

pLDDT
[89.28]

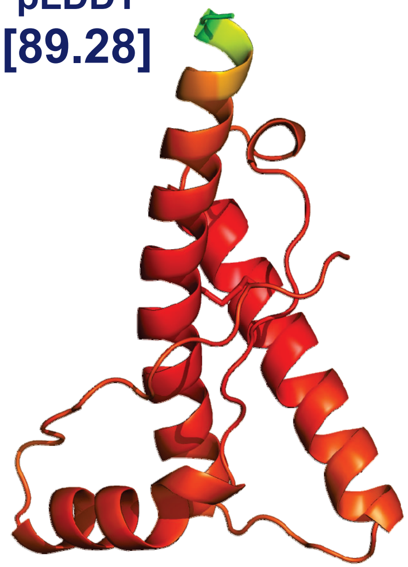

Protein code: 1381

Greater kudu

GenBank: EF165081

Tragelaphus strepsiceros

Artiodactyla 46/50

Bovidae 26/30

P64%

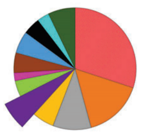

33.9

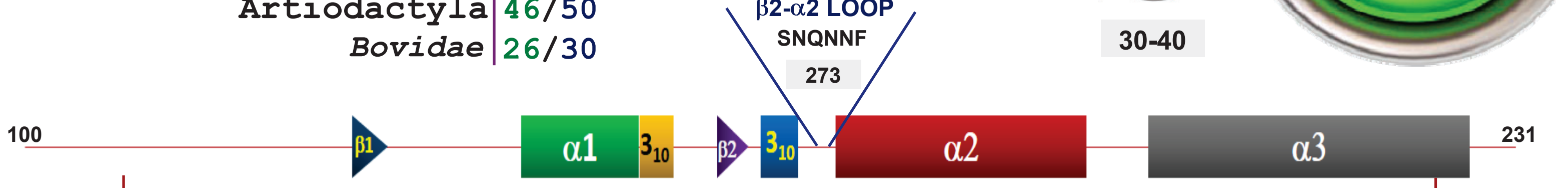

WNKPSKPKTNMKHVAGAAAAGAVVGGLGGYMLGSAMSRPLIHFGSDYEDRYYRENMYRYPNQVYYRPVDQYSNQNNFVHDCVNITVKQHTVTTTTKGENFTETDIKMMERVVEQMCITQYQRESEVYYQRGAS-

I M N W N N I V V K QA EGRS

PrP sequence differs by 12 amino acids from the bank vole PrP (see in red)

No deletions

No insertions

It shows polymorphic variants

Distinct primary sequence across species

135

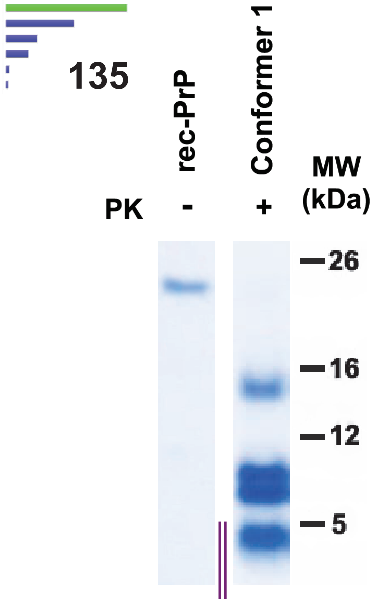

220 AA

1

Conformers

Tm^Exp: ND

ΔΔG: -14.67

In vitro studies

TgVole (1x) Not tested

Greater kudu Not tested

In vivo studies

TgVole (1x) Not tested

Greater kudu Not tested

pLDDT

[86.72]

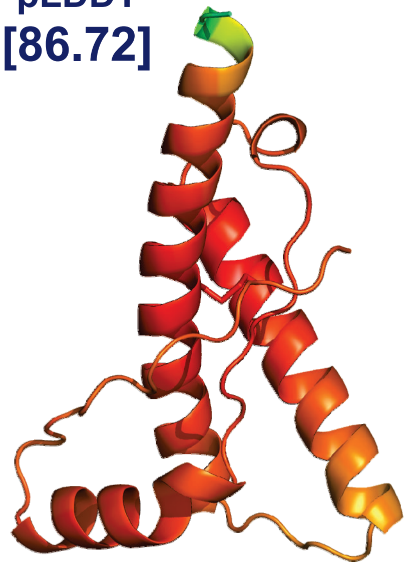

Protein code: 1482

Java mouse-deer

GenBank: BK064004

P100%

100

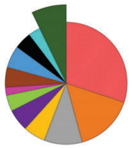

100

Tragulus javanicus

Artiodactyla 46/50

Tragulidae 1/1

1 species sharing the same primary sequence

Lesser mouse-deer
Tragulus kanchil

β2-α2 LOOP
SNQNNF
273

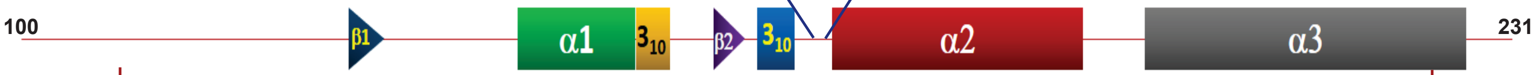

WNKPSKPKTNMKHVAGAAAAGAVVGGLGGYMLGSAMSRPLIHFGNDYEDRYYRENMYRYPNQVYYRPVDQYSNQNNFVHDCVNITIKQHTVTTTTKGENFTETDIKMMERVVEQMCITQYQQESQAYYQRGAS-

I M W N N V V K EGRS

PrP sequence differs by 8 amino acids from the bank vole PrP (see in red)

No deletions

No insertions

No polymorphic variants found

135

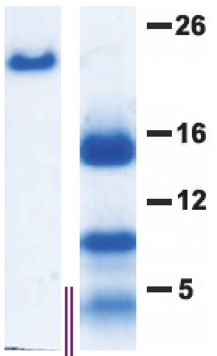

211 AA

1
Conformers

Tm[Exp]: ND

ΔΔG: -12.57

In vitro studies

TgVole (1x) Not tested

Java mouse-deer Not tested

In vivo studies

TgVole (1x) Not tested

Java mouse-deer Not tested

pLDDT
[85.83]

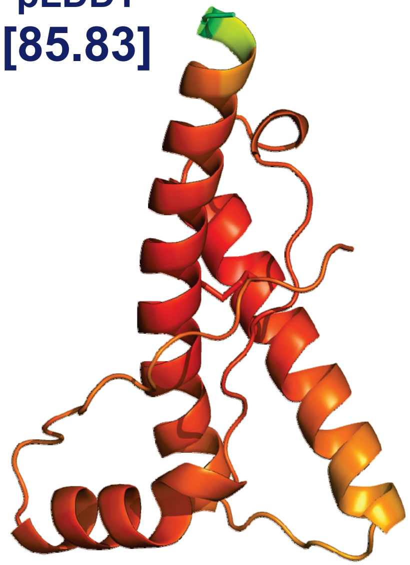

Protein code: 3165

Vicuna

GenBank: BK063925

P47%

12

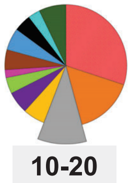

Vicugna vicugna

Artiodactyla 46/50
Camelidae 2/2

2 species sharing the same primary sequence

Guanaco *Lama glama cacsilensis*

Alpaca *Vicugna pacos*

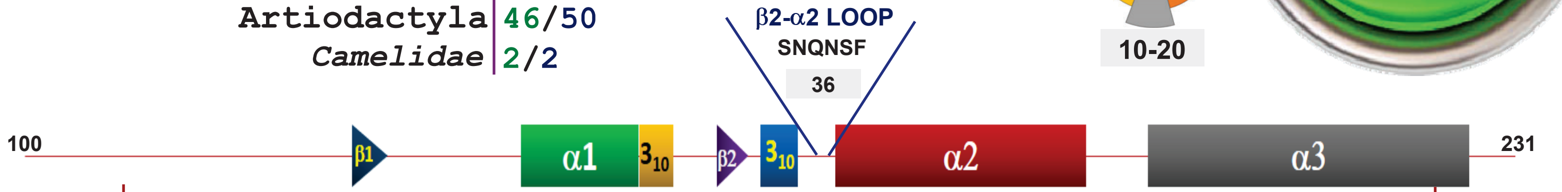


```
WNKPSKPKTSMKHVAGAAAAGAVVGGLGGYMLGSAMSRPLIHFGNDYEDRYYRENMYRYPNQVYYYKPVDQYSNQNSFVHDCVNITVKQHTVTTTTKGENFTETDVKMMERVVEQMCITQYQREYQASYGRGAS-
         NI                             M      W          N       R       N  N         I                              V    K S  Y EGRS
```

PrP sequence differs by 12 amino acids from the bank vole PrP (see in red)

No deletions
No insertions
No polymorphic variants found

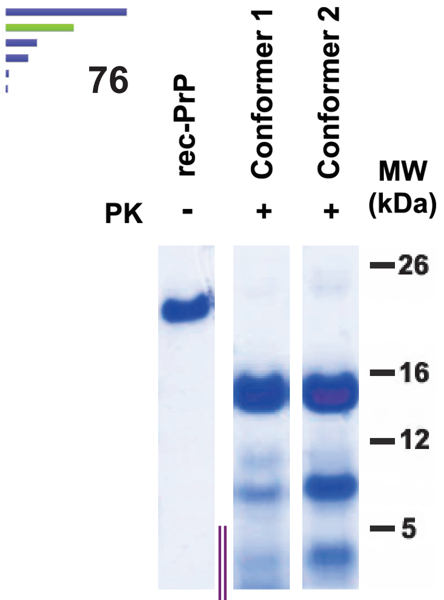

212 AA

2 Conformers

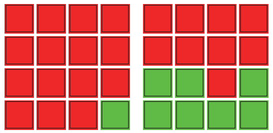

Tm^{Exp}: ND

ΔΔG: -7.28

In vitro studies

TgVole (1x) Not tested

Vicuna Not tested

In vivo studies

TgVole (1x) Not tested

Vicuna Not tested

pLDDT [85.91]

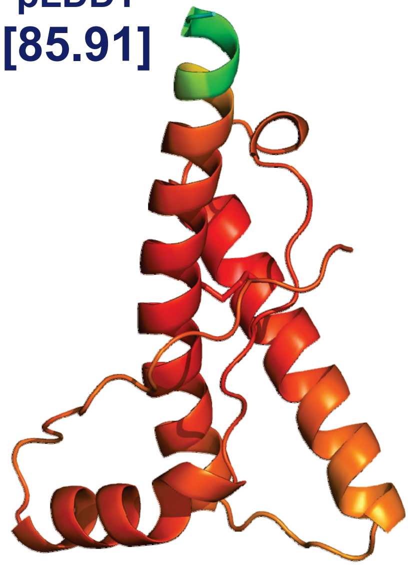

Protein code: 2088

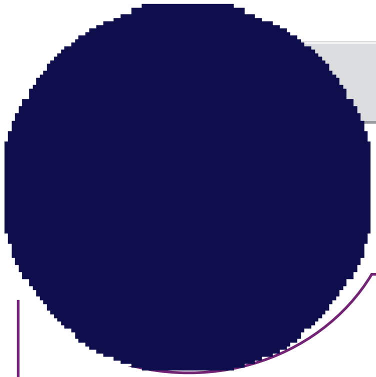

Dog

GenBank: KY649563

Canis lupus familiaris

Carnivora 36/41
Canidae 5/5

P87%

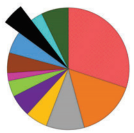

80-90

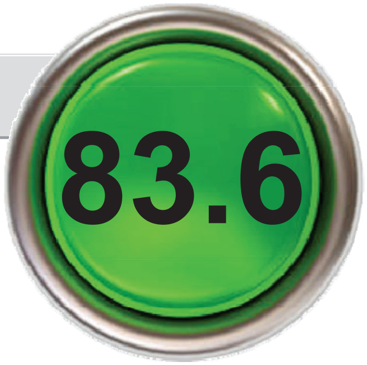

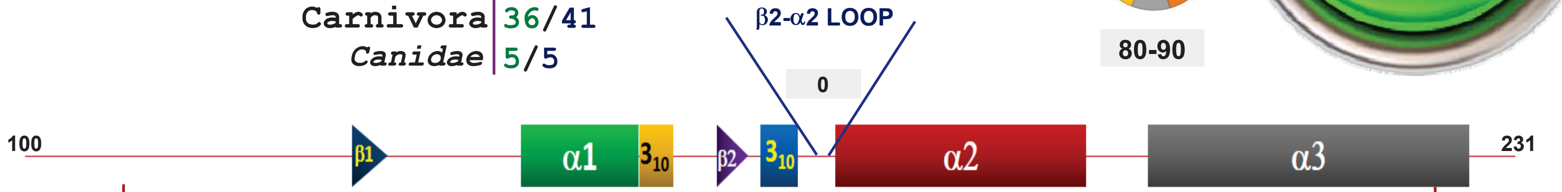

WGKPNKPKTNMKHVAGAAAAGAVVGGLGGYMLGSAMSRPLIHFGNDYEDRYYRENMYRYPEQVYYRPVDQYSNQNNFVRDCVNITVKQHTVTTTTKGENFTETDMKIMERVVEQMCVTQYQKESEAYYQRGAS-

PrP sequence differs by 0 amino acids from the bank vole PrP (see in red)

No deletions

No insertions

It shows polymorphic variants

76

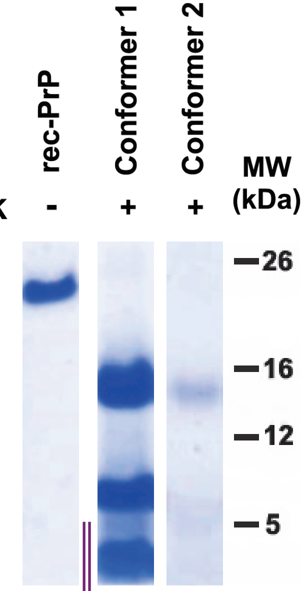

213 AA

2

Conformers

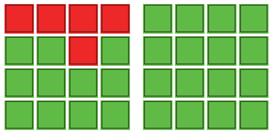

Tm^{Exp}: ND

ΔΔG: -2.04

In vitro studies

TgVole (1x)	Propagate
Dog	Ongoing

In vivo studies

TgVole (1x)	Infectious
TgDog	Ongoing

pLDDT

[84.93]

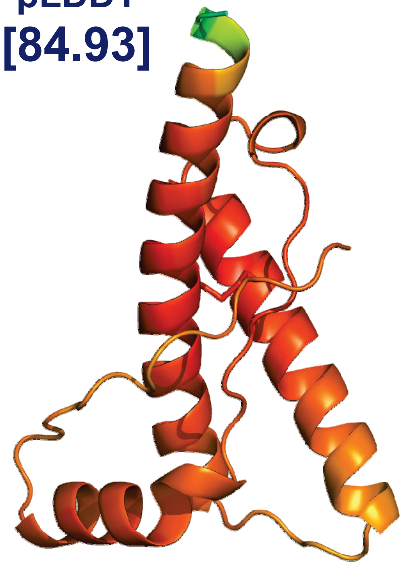

Protein code: 0139

Ferret

GenBank: BK064943

Mustela putorius

Carnivora	36/41
Mustelidae	5/5

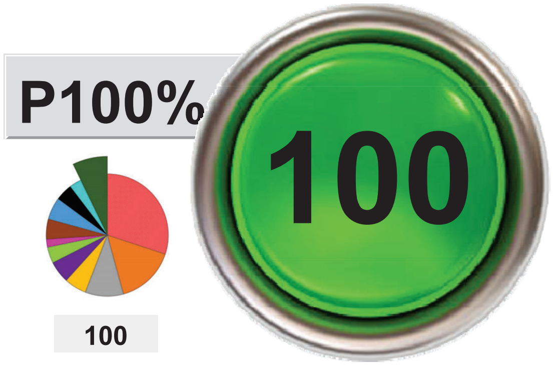

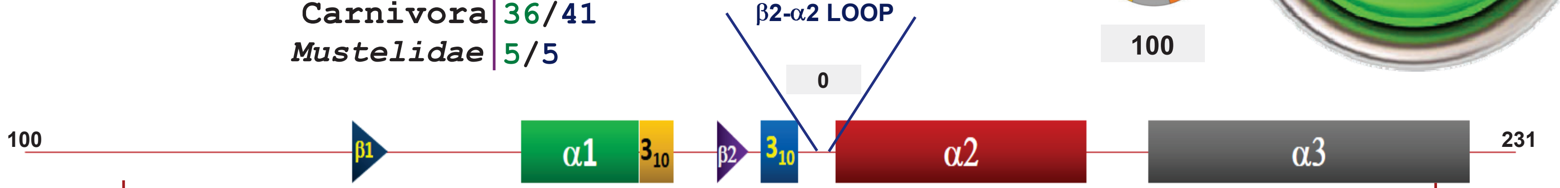

WGKPSKPKTNIKHVAGAAAAGAVVGGLGGYMLGSAMSRPLIHFGNDYEDRYYRENMYRYPNQVYYKPVDQYSNQNNFVHDCVNITVKQHTVTTTTKGENFTETDMKIMERVVEQMCVTQYQQESEAYYQRGAS-

PrP sequence differs by 0 amino acids from the bank vole PrP (see in red)

No deletions

No insertions

It shows polymorphic variants

76

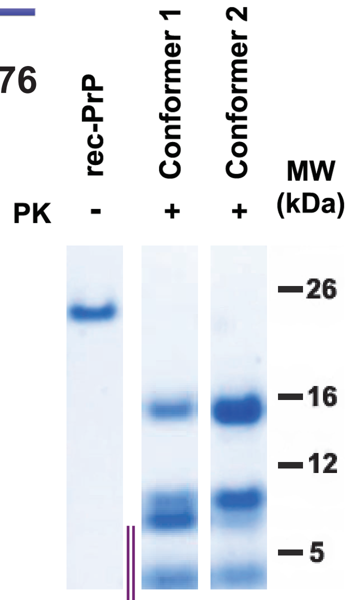

213 AA

2

Conformers

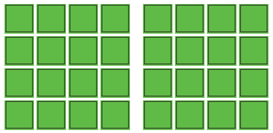

Tm^{Exp}: ND

ΔΔG: -8.29

In vitro studies

TgVole (1x)	Propagate
Ferret	Ongoing

In vivo studies

TgVole (1x)	Ongoing
Ferret	Not tested

pLDDT [85.37]

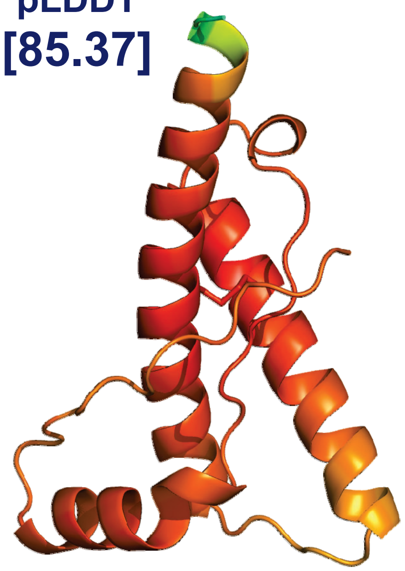

Protein code: 1072

Red panda

GenBank: EU341495

Ailurus fulgens

Carnivora 36/41
Ailuridae 1/1

P78%

67.9

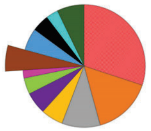

60-70

β2-α2 LOOP
SNQNNF
273

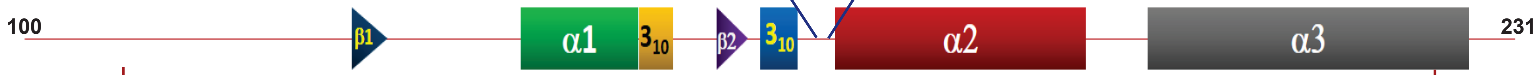

WGKPSKPKTNMKHVAGAAAAGAVVGGLGGYMLGSAMSRPLIHFGNDYEDRYYRENMYRYPNQVYYKPVDQYSNQNNFVHDCVNITVKQHTVTTTTKGENFTETDIKIMERVVEQMCITQYQREAEAYYQRGAS-
N I M W N R N I V M V K SQ EGRS

PrP sequence differs by 13 amino acids from the bank vole PrP (see in red)

No deletions
No insertions
No polymorphic variants found

76

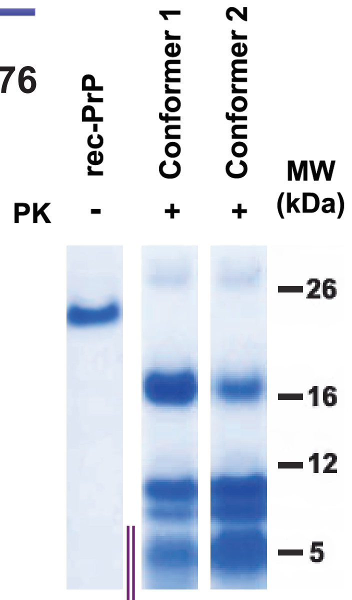

213 AA

2
Conformers

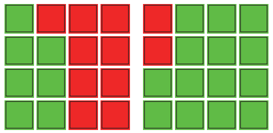

Tm^{Exp}: ND

ΔΔG: -12.32

In vitro studies

TgVole (1x)	Not tested
Red panda	Not tested

In vivo studies

TgVole (1x)	Not tested
Red panda	Not tested

pLDDT
[85.33]

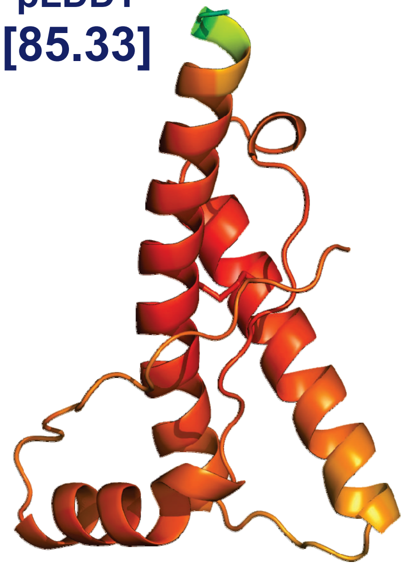

Distinct primary sequence across species

Protein code: 0948

Binturong

GenBank: JX218945

Arctictis binturong

Carnivora 36/41
Viverridae 1/1

P64%

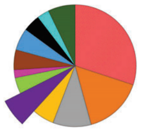

30-40

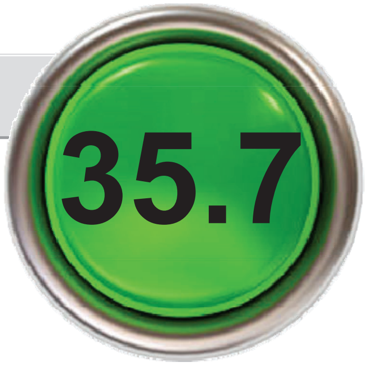

1 species sharing the same primary sequence

Asian palm civet
Paradoxurus hermaphroditus

β2-α2 LOOP
SNQNNF
273

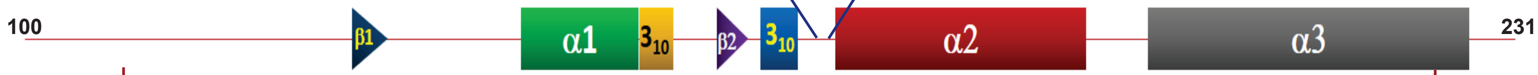

WGKPSKPKTNMKHVAGAAAAGAVVGGLGGYMLGSAMNRPLIHFGNDYEDRYYRENMYRYPNQVYYRPVDQYSNQNNFVHDCVNITVKQHTVTTTTKGENFTETDMKMIERVVEQMCVTQYQRESEAYYQRGAS-
N I S M W N N I V M K Q EGRS

PrP sequence differs by 11 amino acids from the bank vole PrP (see in red)

No deletions
No insertions
No polymorphic variants found

135

Tm^{Exp}: ND
ΔΔG: -5.22

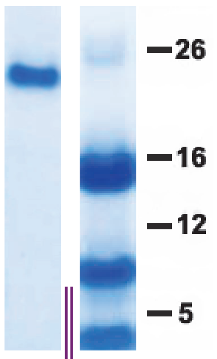

213 AA

1 Conformers

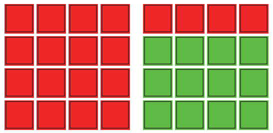

In vitro studies

TgVole (1x)	Not tested
Binturong	Not tested

In vivo studies

TgVole (1x)	Not tested
Binturong	Not tested

pLDDT
[85.54]

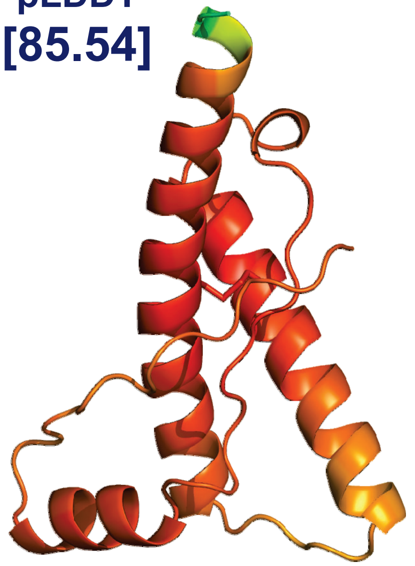

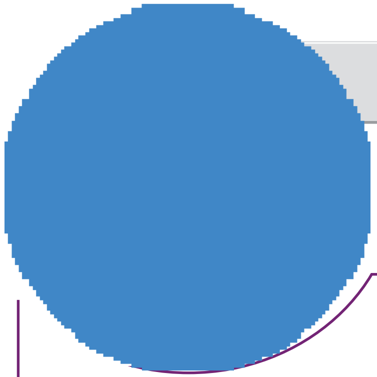

Protein code: 0925

Antarctic fur seal

GenBank: BK064071

Arctocephalus gazella

Carnivora 36/41
Otariidae 1/1

Distinct primary sequence across species

P62% **32.5**

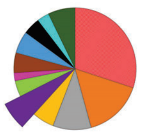

30-40

β2-α2 LOOP
SNQNNF
273

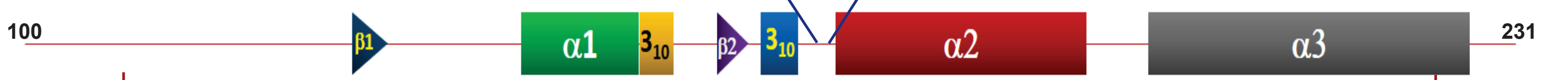


```
WGKPQKPKTNMKHMAGAAAAGAVVGGLGGYMLGSAMSRPLIHFGNDYEDRYYRENMNRYPNQVYYKPVDQYSNQNNFVHDCVNITVKQHTVTTTTKGENFTETDMKIMERVVEQMCVTQYQRESEAYYQRGAS-
 N S   I V                          M      W                  R    N         I                   V M               K Q  EGRS
```

PrP sequence differs by 11 amino acids from the bank vole PrP (see in red)

No deletions
No insertions
No polymorphic variants found

76

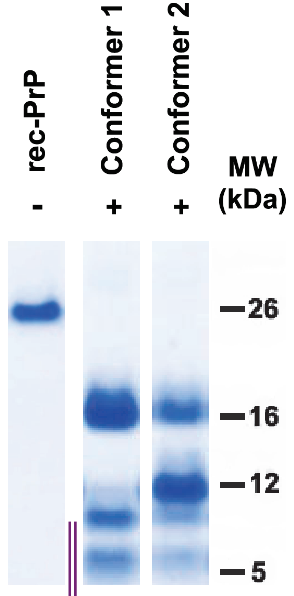

215 AA

2 Conformers

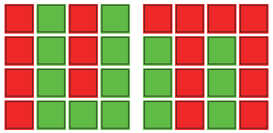

Tm^{Exp}: ND

ΔΔG: -11.5

In vitro studies

TgVole (1x) Not tested

Antarctic fur seal Not tested

In vivo studies

TgVole (1x) Not tested

Antarctic fur seal Not tested

pLDDT
[87.29]

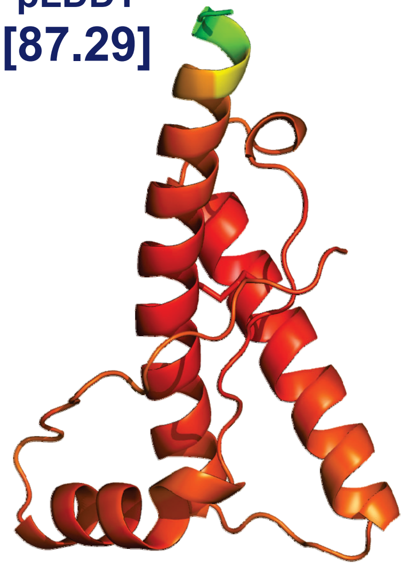

Protein code: 3248

Coyote

GenBank: JX218953

Canis latrans

Carnivora	36/41
Canidae	5/5

2 species sharing the same primary sequence

Maned wolf *Chrysocyon brachyurus*

Bush dog *Speothos venaticus*

P76%

64.3

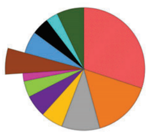

60-70

β2-α2 LOOP
SNQNNF
273

100

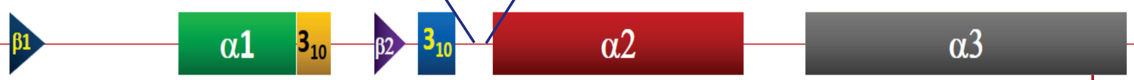

231

WGKPNKPKTNMKHVAGAAAAGAVVGGLGGYMLGSAMSRPLIHFGNDYEDRYYRENMYRYPDQVYYRPVDQYSNQNNFVRDCVNITVKQHTVTTTTKGENFTETDMKIMERVVEQMCVTQYQKESEAYYQRGAS-

N S I M W N N N H I V M Q EGRS

PrP sequence differs by 11 amino acids from the bank vole PrP (see in red)

No deletions

No insertions

No polymorphic variants found

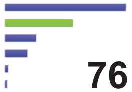

76

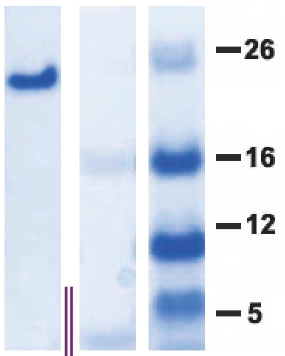

213 AA

2

Conformers

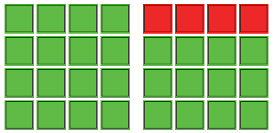

Tm[Exp]: ND

ΔΔG: -5.73

pLDDT [85.42]

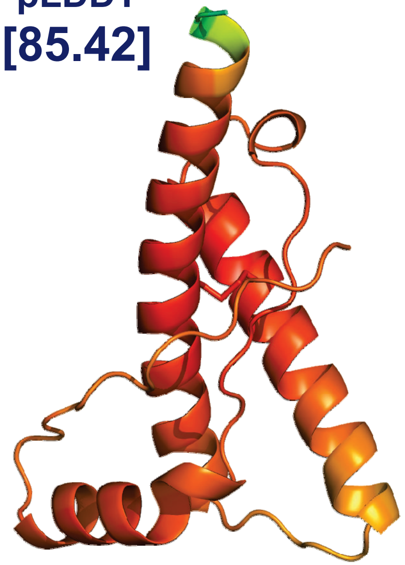

In vitro studies

TgVole (1x)	Not tested
Coyote	Not tested

In vivo studies

TgVole (1x)	Not tested
Coyote	Not tested

Protein code: 1353

Black-backed jackal

GenBank: BK064772

Canis mesomelas

Carnivora 36/41
Canidae 5/5

P57%

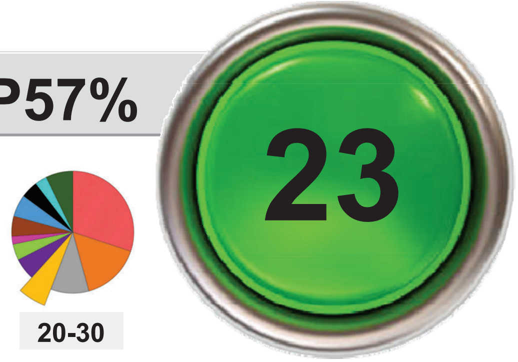

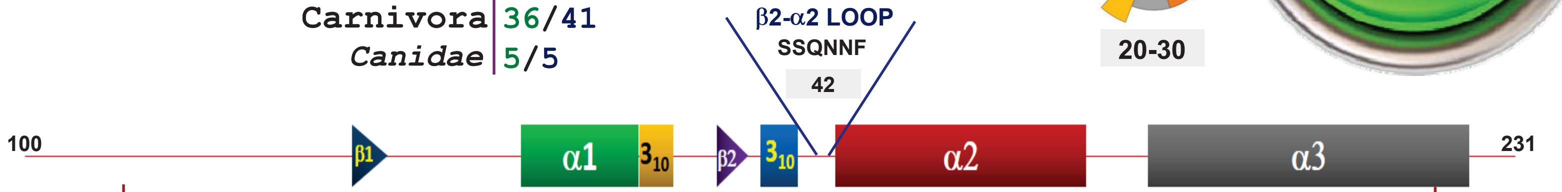

WGKPNKPKTNMKHVAGAAAAGAVVGGLGGYMLGSAMSRPLIHFGNDYEDRYYRENMYRYPDQVYYRPVDQYSSQNNFVRDCVNITVKQHTVTTTTKGENFTETDMKIMERVVEQMCVTQYQKESEAYYQRGAS-
N S I M W N N NN H I V M Q EGRS

PrP sequence differs by 12 amino acids from the bank vole PrP (see in red)

No deletions
No insertions
No polymorphic variants found

Distinct primary sequence across species

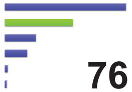

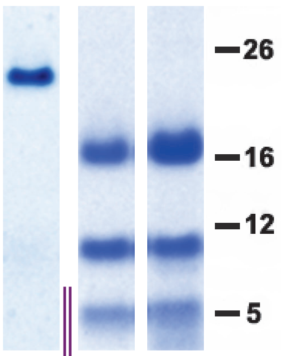

213 AA

2
Conformers

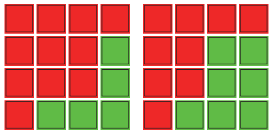

Tm^{Exp}: ND

ΔΔG: -8.24

In vitro studies

TgVole (1x) Not tested

Black-backed jackal Not tested

In vivo studies

TgVole (1x) Not tested

Black-backed jackal Not tested

pLDDT
[85.08]

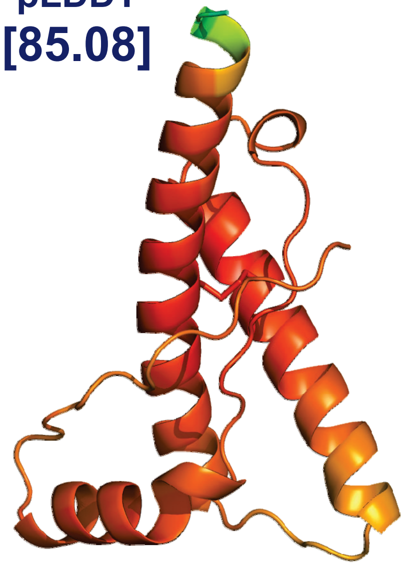

Protein code: 3927

Caracal

GenBank: BK063932

Caracal caracal

Carnivora 36/41

Felidae 7/9

P29%

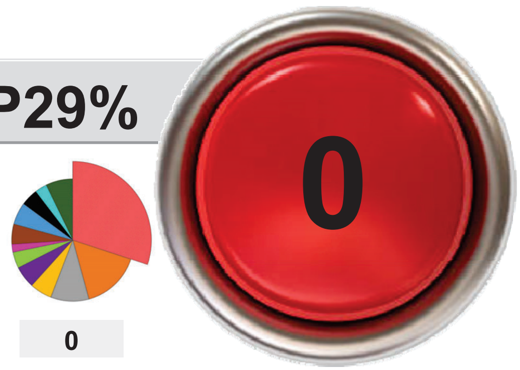

β2-α2 LOOP
SSQNNF
42

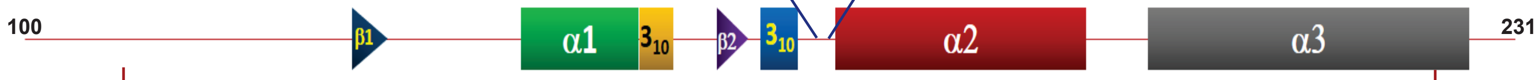

WGKPSKPKTNMKHMAGAAAAGAVVGGLGGYMLGSAMNRPLIHFGNDYEDRYYRENMYRYPNQVYYRPVDQYSSQNNFVHDCVNITVRQHTVTTTTKGENFTETDMKIMERVVEQMCVTQYQKESEAYYQRGAS-

N I V S M W N NN IK V M Q EGRS

PrP sequence differs by 13 amino acids from the bank vole PrP (see in red)

No deletions

No insertions

No polymorphic variants found

Tm^{Exp}: ND

ΔΔG: -6.41

Distinct primary sequence across species

PK

rec-PrP

-

MW (kDa)

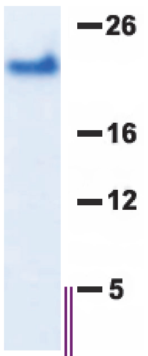

216 AA

NO MISFOLDING

0

Conformers

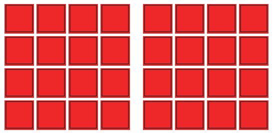

In vitro studies

NOT APPLICABLE

In vivo studies

NOT APPLICABLE

pLDDT

[85.46]

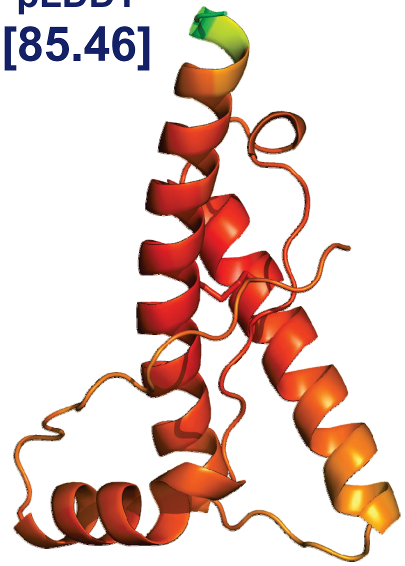

Protein code: 3054

Asian golden cat

GenBank: JX218947

Catopuma temminckii

Carnivora 36/41
Felidae 7/9

P76%

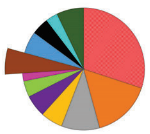

60-70

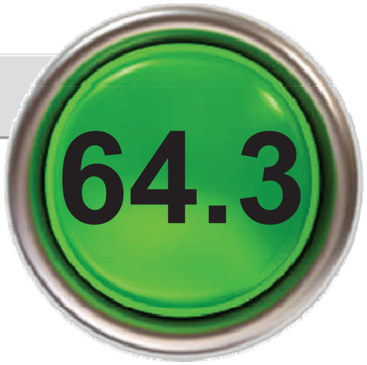

β2-α2 LOOP
SNQNSF
36

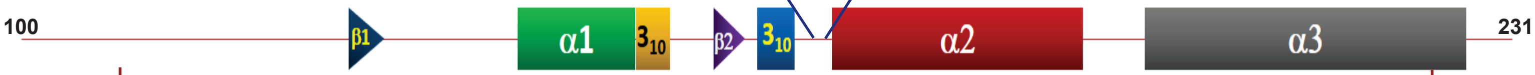

WGKPSKPKTNMKHMAGAAAAGAVVGGLGGYMLGSAMSRPLIHFGNDYEDRYYRENMYRYPNQVYYRPVDQYSNQNSFVHDCVNITVRQHTVTTTTKGENFTETDMKIMERVVEQMCVTQYQKESEAYYQRGAS-
N I V M W N N N IK V M Q EGRS

PrP sequence differs by 12 amino acids from the bank vole PrP (see in red)

No deletions
No insertions
No polymorphic variants found

Distinct primary sequence across species

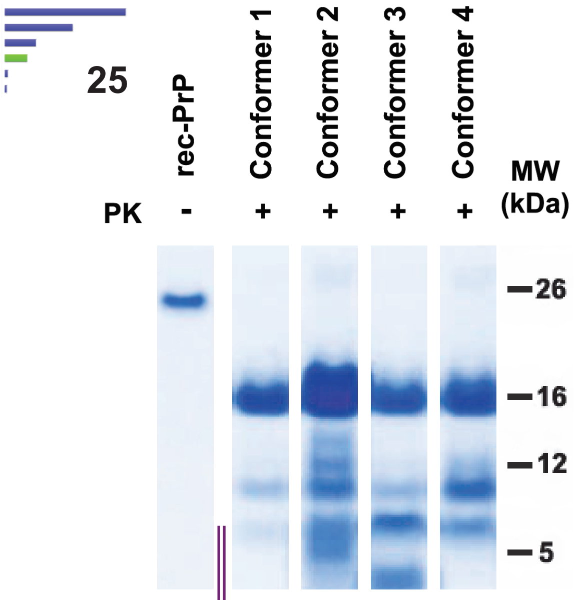

216 AA

4 Conformers

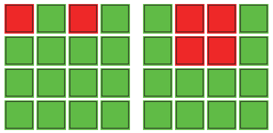

Tm[Exp]: ND

ΔΔG: -12.45

pLDDT [86.82]

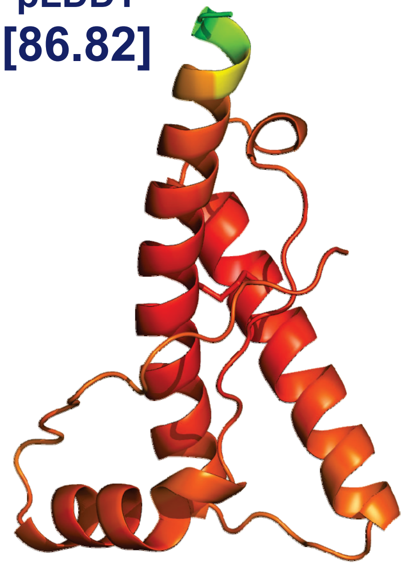

In vitro studies

TgVole (1x) Not tested

Asian golden cat Not tested

In vivo studies

TgVole (1x) Not tested

Asian golden cat Not tested

Protein code: 1408

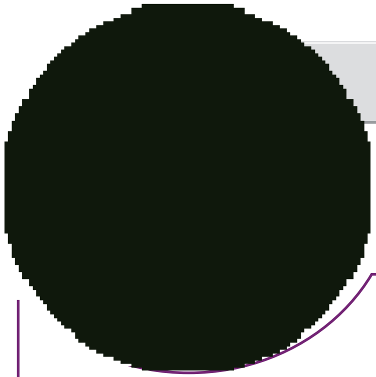

Spotted hyena

GenBank: JX218985

Crocuta crocuta

Carnivora 36/41

Hyaenidae 1/2

P29%

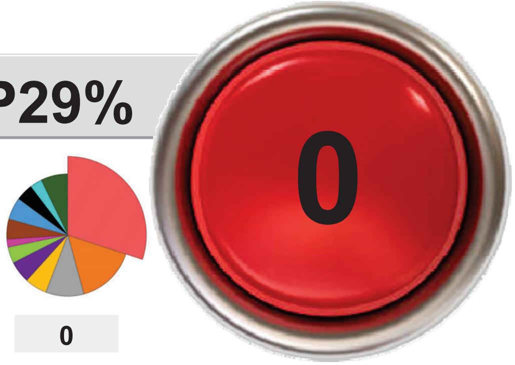

1 species sharing the same primary sequence

Striped hyena
Hyaena hyaena

β2-α2 LOOP
SSQNNF
42

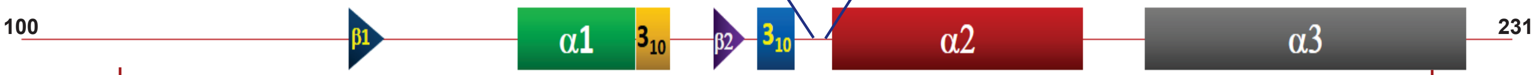

WGKPSKPKTNMKHMAGAAAAGAVVGGLGGYMLGSAMSRPLIHFGNDYEDRYYRQNMDRYPNQVYYKPVDQYSSQNNFVHDCVNITVRQHTVTTTTKGENFTETDMKIMERVVEQMCVTQYQRESEAYYQRGAS-

N I V M W E N R NN IK V M K Q EGRS

PrP sequence differs by 15 amino acids from the bank vole PrP (see in red)

No deletions

No insertions

No polymorphic variants found

Tm^{Exp}: ND

ΔΔG: -9.33

PK rec-PrP -

MW (kDa)

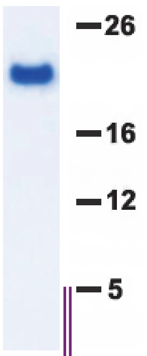

213 AAN

NO MISFOLDING

0 Conformers

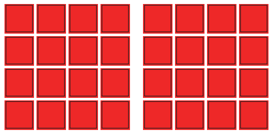

In vitro studies

NOT APPLICABLE

In vivo studies

NOT APPLICABLE

pLDDT [85.27]

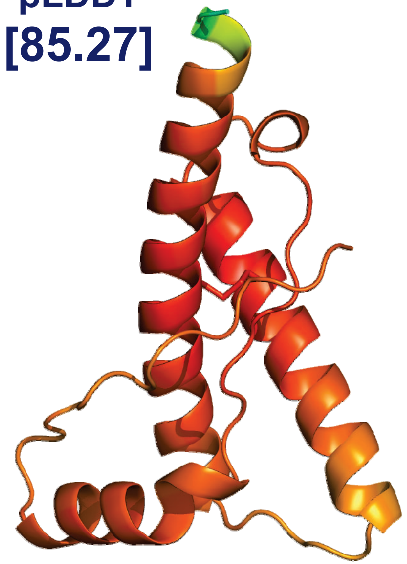

Protein code: 0531

Fossa

GenBank: BK063948

Cryptoprocta ferox

Carnivora	36/41
Eupleridae	1/1

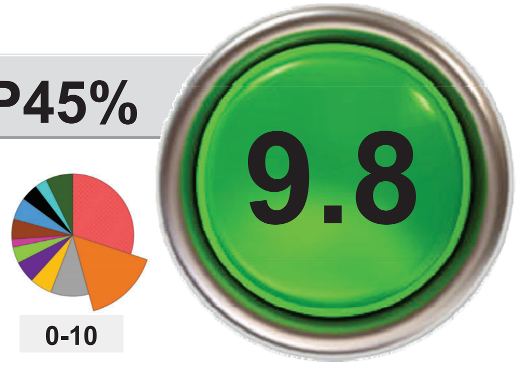

β2-α2 LOOP
SSQNNFF
42

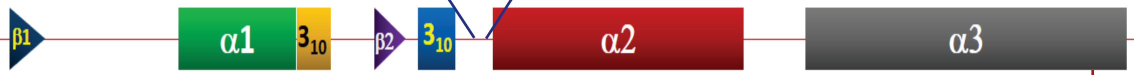

WGKPSKPKTNMKHMAGAAAAGAVVGGLGGYMLGSAMSRPLIHFGNDYEDRYYRENMYRYPNQVYYRPVDQYSSQNNFVHDCVNITVRQHTVTTTTKGENFTETDMKIMERVVEQMCVTQYQKESEAYYQRGAS-

N I V M W N NN IK V M Q EGRS

PrP sequence differs by 12 amino acids from the bank vole PrP (see in red)

No deletions
No insertions
No polymorphic variants found

Distinct primary sequence across species

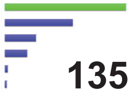

135

213 AA

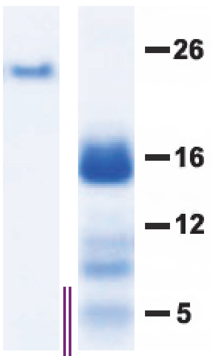

1
Conformers

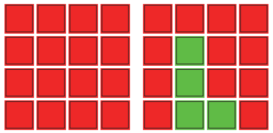

Tm^{Exp}: ND

ΔΔG: -8.09

In vitro studies

TgVole (1x) Not tested

Fossa Not tested

In vivo studies

TgVole (1x) Not tested

Fossa Not tested

pLDDT
[85.56]

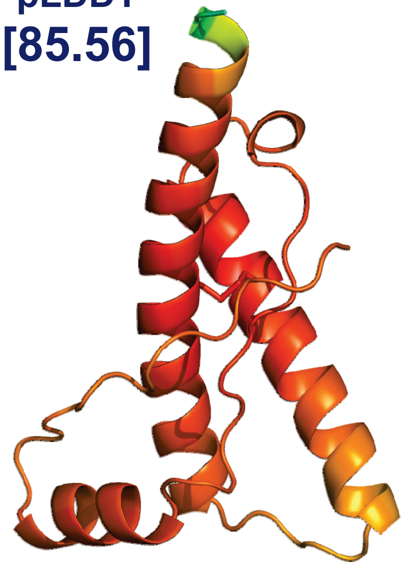

Protein code: 3075

Bearded seal

GenBank: BK064769

Erignathus barbatus

Carnivora 36/41
Phocidae 4/6

P29%

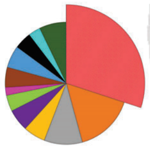

0

0

β2-α2 LOOP
SNQNNF
273

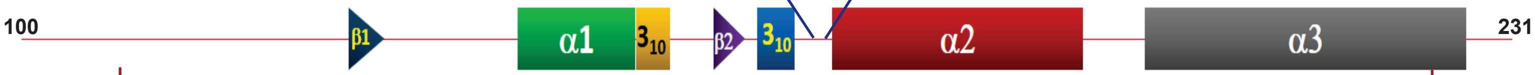

WGKPNKPKTNMKHMAGAAAAGAVVGGLGGYMLGSAMSRPLIHFGNDYEDRYYRENMYRYPNQVYYYKPVDQYSNQNNFVHDCVNITVKQHTVTTTTKGENFTETDMKIMERVVEQMCVTQYQRESEAYYQRGAS-
N S I V M W N R N I V M K Q EGRS

PrP sequence differs by 12 amino acids from the bank vole PrP (see in red)

No deletions
No insertions
No polymorphic variants found

Distinct primary sequence across species

Tm^{Exp}: ND

ΔΔG: -8.49

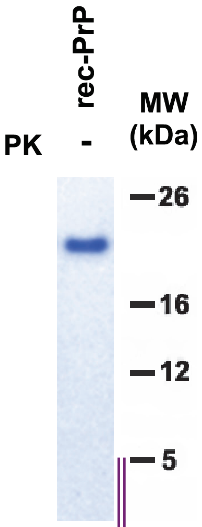

213 AA

NO MISFOLDING

0 Conformers

In vitro studies

NOT APPLICABLE

In vivo studies

NOT APPLICABLE

pLDDT [85.87]

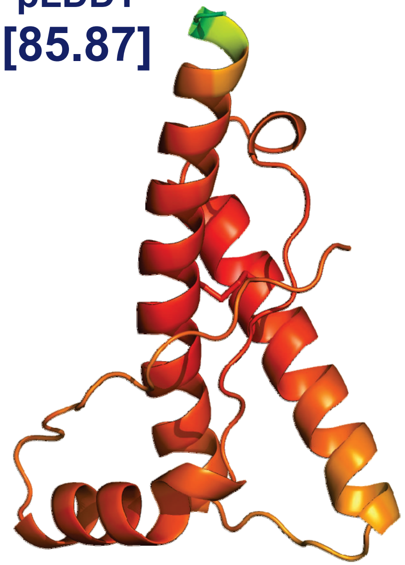

Protein code: 3919

Cat

GenBank: EU588730

Felis silvestris catus

Carnivora 36/41
Felidae 7/9

P49%

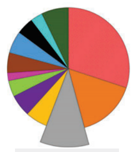

10-20

12.9

3 species sharing the same primary sequence

Jungle cat
Felis chaus

Black-footed cat
Felis nigripes

Pallas's cat
Otocolobus manul

β2-α2 LOOP
SNQNNF
273

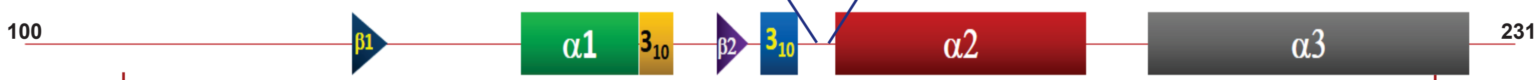

WGKPSKPKTNMKHMAGAAAAGAVVGGLGGYMLGSAMSRPLIHFGNDYEDRYYRENMYRYPNQVYYRPVDQYSNQNNFVHDCVNITVRQHTVTTTTKGENFTETDMKIMERVVEQMCVTQYQKESEAYYQRGAS-

N I V M W N N IK V M Q EGRS

PrP sequence differs by 11 amino acids from the bank vole PrP (see in red)

No deletions
No insertions
No polymorphic variants found

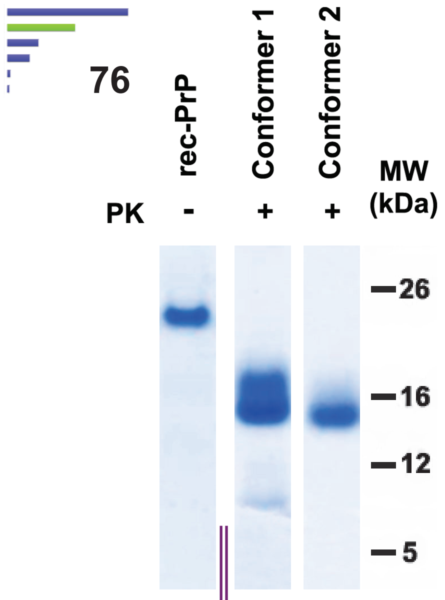

216 AA

2
Conformers

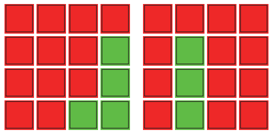

Tm^{Exp}: ND

ΔΔG: -8.92

In vitro studies

TgVole (1x) Propagate

Cat Ongoing

In vivo studies

TgVole (1x) Ongoing

Cat Not tested

pLDDT
[85.65]

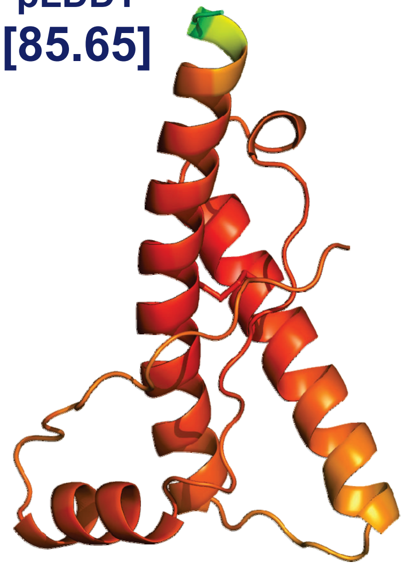

Protein code: 0173

Wolverine

GenBank: EU341500

P61% **29.8**

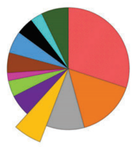

20-30

Gulo gulo

Carnivora 36/41

Mustelidae 5/5

β2-α2 LOOP
SNQNNF
273

100

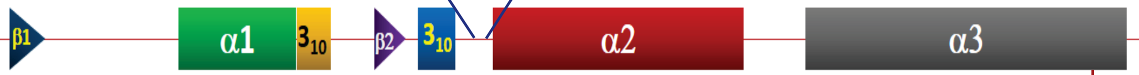

231

WGKPSKPKTNMKHVAGAAAAGAVVGGLGGYMLGSAMSRPLIHFGNDYEDRYYRENMHRYPDQVYYKPVDQYSNQNNFVHDCVNITVKQHTVTTTTKGENFTETDMKIMERVVEQMCVTQYQRESEAYYQRGAS-

N I M W N N R N I V M K Q EGRS

PrP sequence differs by 12 amino acids from the bank vole PrP (see in red)

No deletions

No insertions

No polymorphic variants found

6 species sharing the same primary sequence

Tayra *Eira barbara*

Yellow-throated marten *Martes flavigula*

Beech marten *Martes foina*

European pine marten *Martes martes*

Sable *Martes zibellina*

American badger *Taxidea taxus jeffersonii*

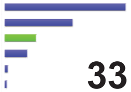

33

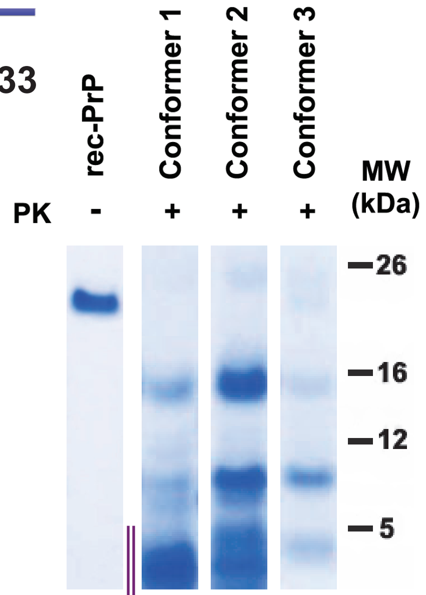

213 AA

3 Conformers

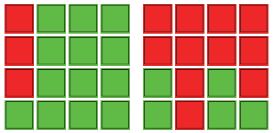

Tm^{Exp}: ND

ΔΔG: -4.08

In vitro studies

TgVole (1x)	Not tested
Wolverine	Not tested

In vivo studies

TgVole (1x)	Not tested
Wolverine	Not tested

pLDDT [85.02]

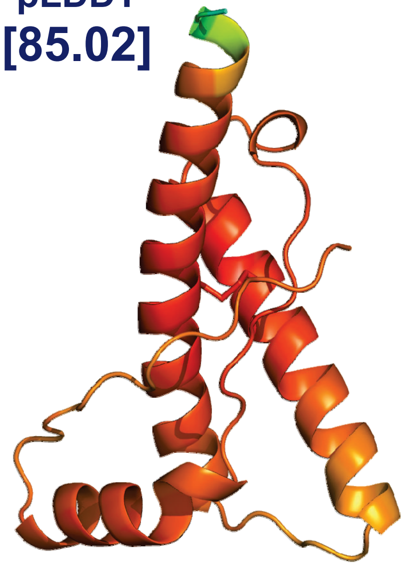

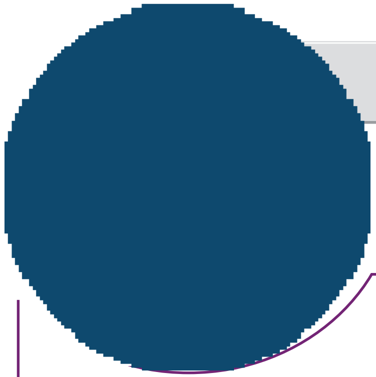

Common dwarf mongoose

GenBank: BK064051

Helogale parvula

Carnivora 36/41
Herpestidae 4/4

P34%

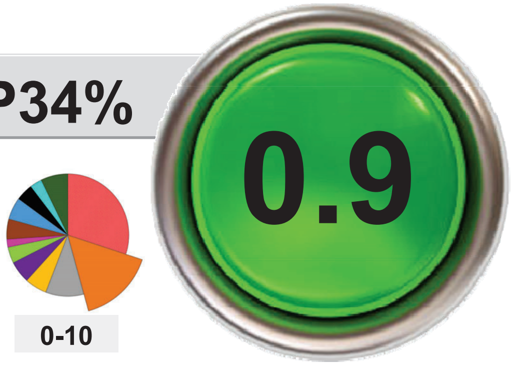

β2-α2 LOOP
SSQNNF
42

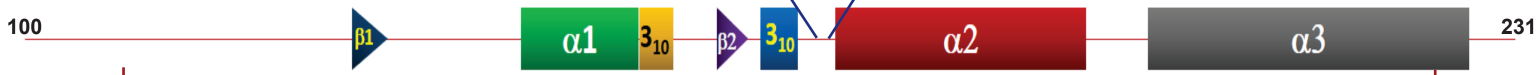

WGKPSKPKTNMKHVAGAAAAGAVVGGLGGYMLGSAMSRPIIHFGNDYEDRYYRQNMDRYPNQVYYRPVDQYSSQNNFVHDCVNITVRQHTVTTTTKGENFTETDMKIMERVVEQMCVTQYEKESQAYYQRGAS-
N I M W E N NN IK V M Q EGRS

PrP sequence differs by 12 amino acids from the bank vole PrP (see in red)

No deletions
No insertions
No polymorphic variants found

Distinct primary sequence across species

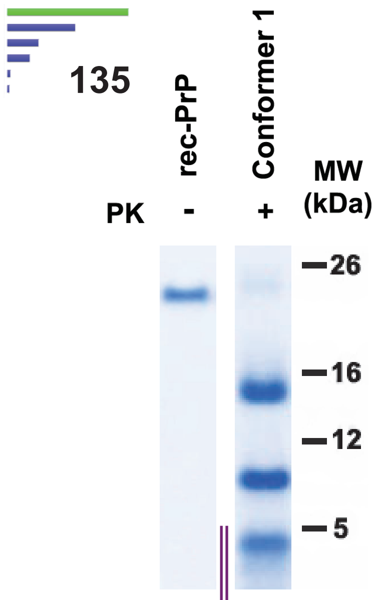

217 AA

1
Conformers

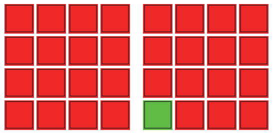

Tm[Exp]: ND

ΔΔG: -11.52

pLDDT
[84.99]

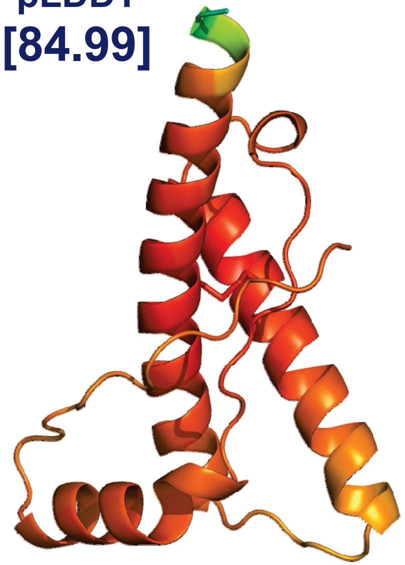

In vitro studies

Not tested
TgVole (1x)

Not tested
Common dwarf mongoose

In vivo studies

Not tested
TgVole (1x)

Not tested
Common dwarf mongoose

Protein code: 3220

Javan mongoose

GenBank: EU341501

Herpestes javanicus

Carnivora 36/41
Herpestidae 4/4

P66%

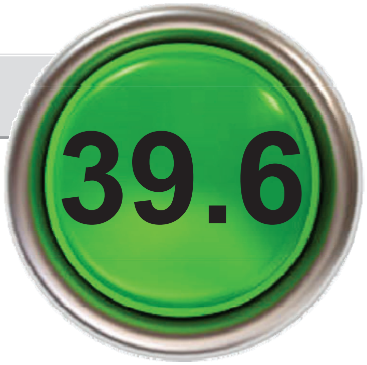

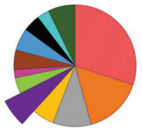

30-40

β2-α2 LOOP
SNQNNF
273

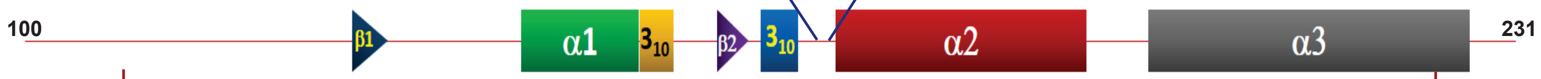

WGKPSKPKTNMKHVAGAAAAGAVVGGLGGYMLGSAMSRPIIHFGNDYEDRYYRQNMDRYPNQVYYRPVDQYSNQNNFVHDCVNITVRQHTVTTTTKGENFTETDMKIMERVVEQMCVTQYEKESQAYYQRGAS-
N I M W E N N IK V M Q EGRS

PrP sequence differs by 11 amino acids from the bank vole PrP (see in red)

No deletions
No insertions
No polymorphic variants found

Distinct primary sequence across species

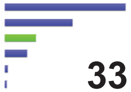

33

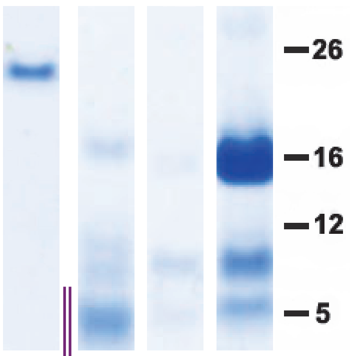

217 AA

3
Conformers

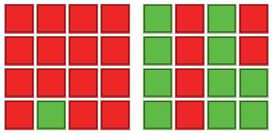

Tm^{Exp}: ND

ΔΔG: -6.95

In vitro studies

TgVole (1x) Not tested

Javan mongoose Not tested

In vivo studies

TgVole (1x) Not tested

Javan mongoose Not tested

pLDDT
[85.35]

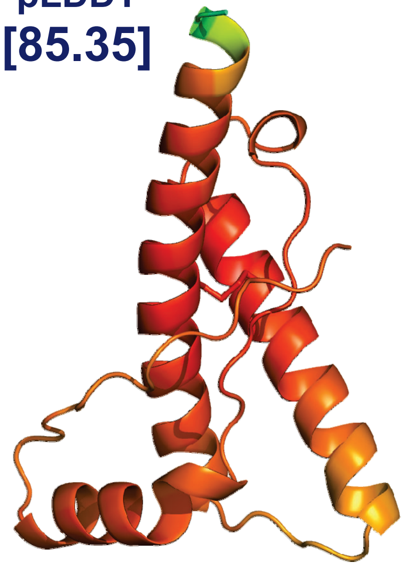

Protein code: 1345

Geoffroy's cat

GenBank: BK064182

Leopardus geoffroyi

Carnivora 36/41
Felidae 7/9

P39%

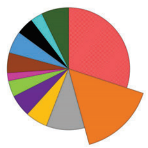

0-10

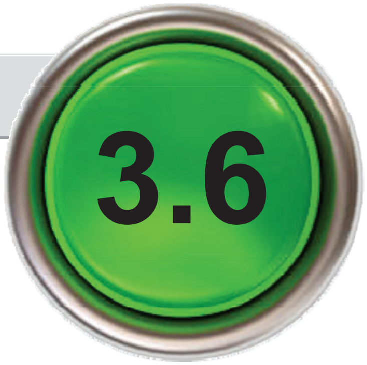

β2-α2 LOOP
SNQNNF
273

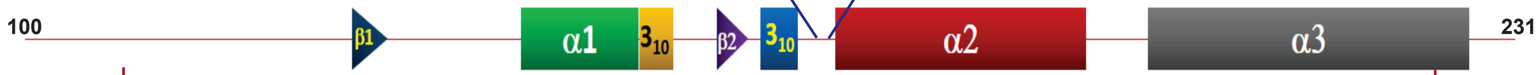

WGKPSKPKTNMKHMAGAAAAGAVVGGLGGYMLGSAMSRPLIHFGNDYEDRYYRENMYRYPNQVYYRPVDQYSNQNNFVHDCVNITVRQHTVTTTTKGENFTETDMKIMERVVEQMCVTQYQKESEAYYQRGAS-

N I V M W N N IK V M Q EGRS

PrP sequence differs by 11 amino acids from the bank vole PrP (see in red)

No deletions
No insertions
No polymorphic variants found

Distinct primary sequence across species

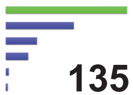

135

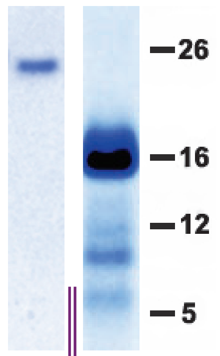

216 AA

1
Conformers

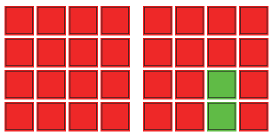

Tm^{Exp}: ND

ΔΔG: -7.1

In vitro studies

TgVole (1x) Not tested

Geoffroy's cat Not tested

In vivo studies

TgVole (1x) Not tested

Geoffroy's cat Not tested

pLDDT
[85.81]

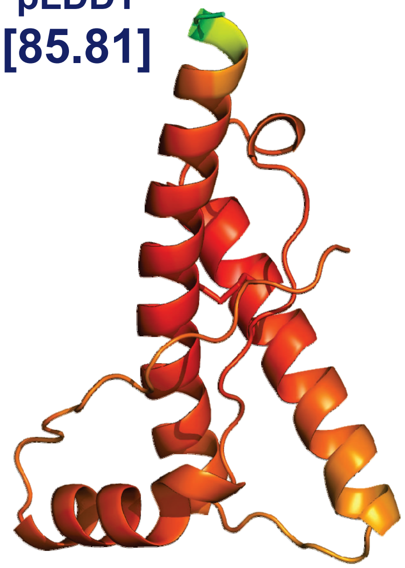

Protein code: 4011

Oncilla

GenBank: BK064829

Leopardus tigrinus

Carnivora 36/41
Felidae 7/9

P29%

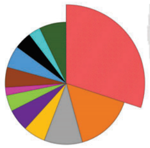

0

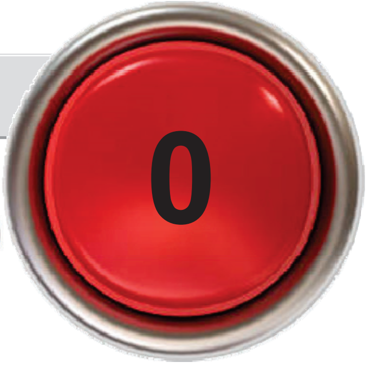

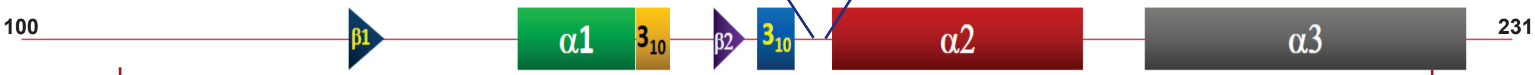

WGKPNKPKTNMKHMAGAAAAGAVVGGLGGYMLGSAMSRPLIHFGNDYEDRYYRENMYRYPNQVYYRPVDQYSNQNNFVHDCVNITVRQHTVTTTTKGENFTETDMKIMERVVEQMCVTQYQKESEAYYQRGAS-
N S I V M W N N IK V M Q EGRS

PrP sequence differs by 11 amino acids from the bank vole PrP (see in red)

No deletions
No insertions
No polymorphic variants found

Distinct primary sequence across species

Tm[Exp]: ND
ΔΔG: -14.52

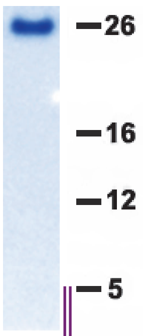

216 AA

NO MISFOLDING

0 Conformers

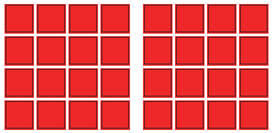

In vitro studies

NOT APPLICABLE

In vivo studies

NOT APPLICABLE

pLDDT [93.41]

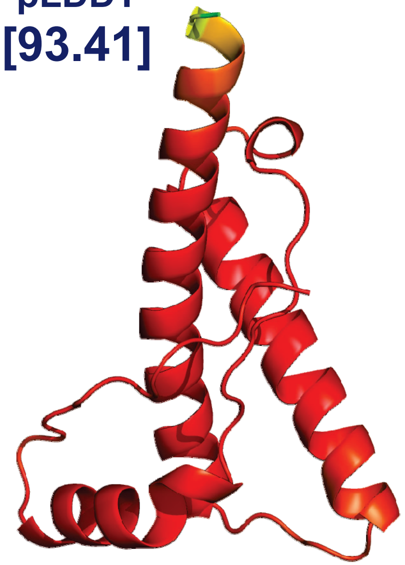

Protein code: 4815

Margay

GenBank: JX218979

Leopardus wiedii

Carnivora	36/41
Felidae	7/9

P34%

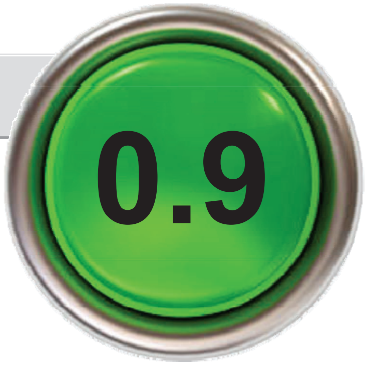

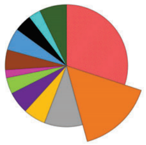

0-10

β2-α2 LOOP
SNQNSF
36

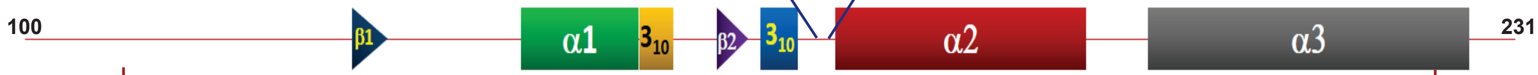


```
WGKPSKPKTNMKHMAGAAAAGAVVGGLGGYMLGSAMSRPLIHFGNDYEDRYYRENMYRYPNQVYYRPVDQYSNQNSFVHDCVNITVRQHTVTTTTKGENFTETDMKIMERVVEQMCVTQYQKESEAYYYQRGAS-
 N        I  V                         M      W         N              N   N          IK                      V M                  Q   EG SS
```

PrP sequence differs by 12 amino acids from the bank vole PrP (see in red)

No deletions
No insertions
No polymorphic variants found

Distinct primary sequence across species

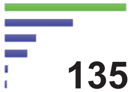

135

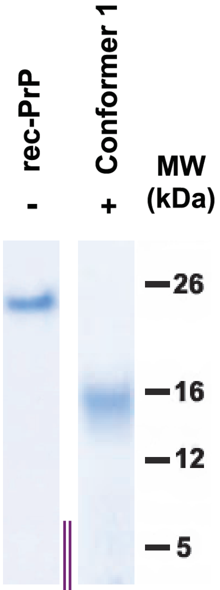

217 AA

1
Conformers

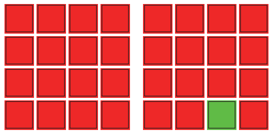

Tm^{Exp}: ND

ΔΔG: ND

In vitro studies

TgVole (1x)	Not tested
Margay	Not tested

In vivo studies

TgVole (1x)	Not tested
Margay	Not tested

pLDDT
[85.39]

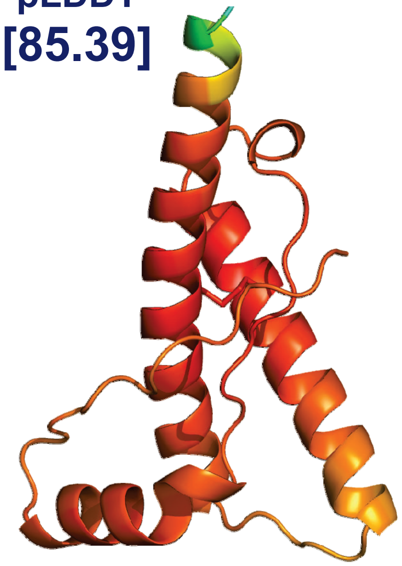

Protein code: 1443

Weddell seal

GenBank: XM_006744312

Leptonychotes weddellii

Carnivora 36/41
Phocidae 4/6

P76%

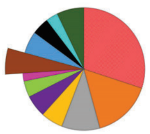

66.1

β2-α2 LOOP
SSQNNF
42

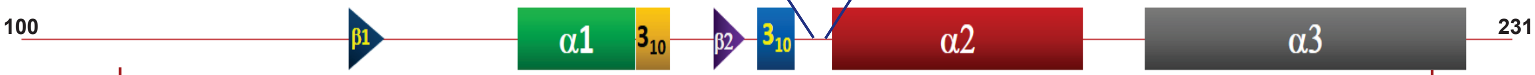

WGKPNKPKTNMKHMAGAAAAGAVVGGLGGYMLGSAMSRPLIHFGNDYEDRYYRENMHRYPNQVYYKPVDQYSSQNNFVHDCVNITVKQHTVTTTTKGENFTETDMKIMERVVEQMCVSQYQRESEAYYQRGAS-
N S I V M W N R NN I V M T K Q EGRS

PrP sequence differs by 14 amino acids from the bank vole PrP (see in red)

No deletions
No insertions
No polymorphic variants found

Distinct primary sequence across species

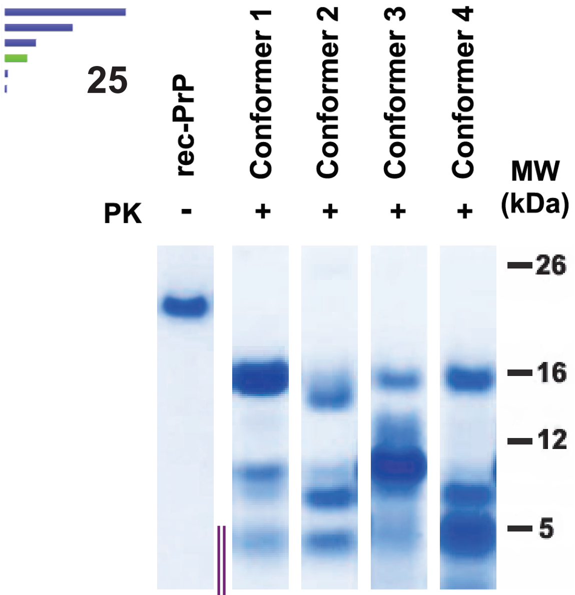

213 AA

4
Conformers

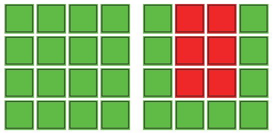

Tm^{Exp}: ND

ΔΔG: -6.26

In vitro studies

TgVole (1x) Not tested

Weddell seal Not tested

In vivo studies

TgVole (1x) Not tested

Weddell seal Not tested

pLDDT
[85.17]

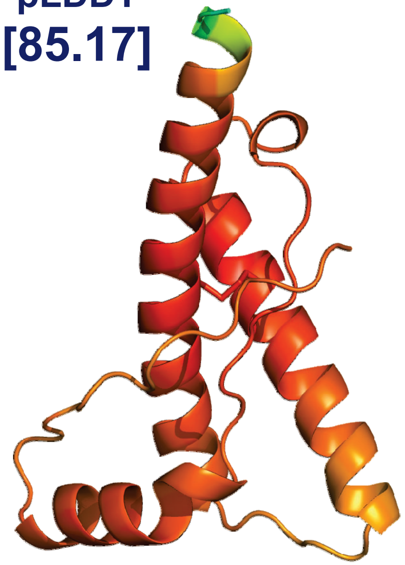

Protein code: 1081

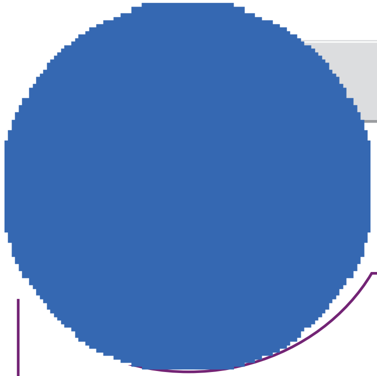

African wild dog

GenBank: BK064019

Lycaon pictus

Carnivora	36/41
Canidae	5/5

P100%

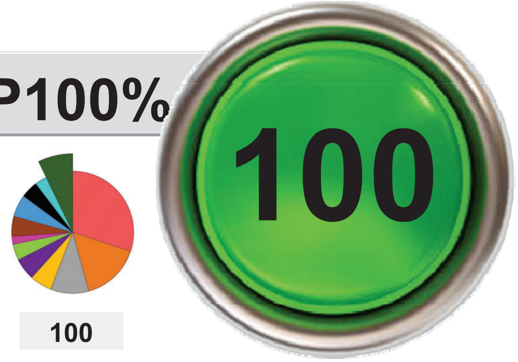

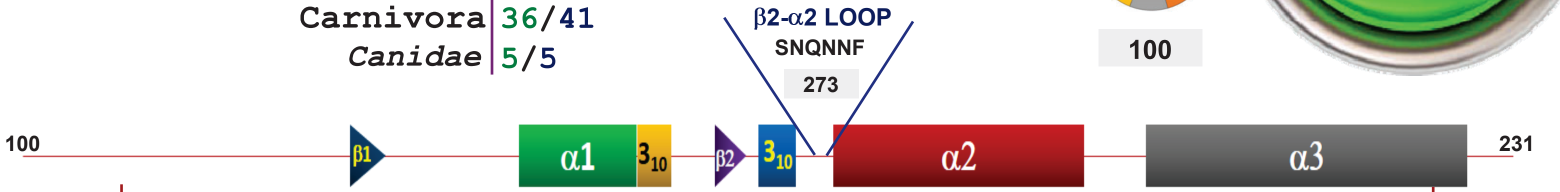

WGKPSKPKTNMKHVAGAAAAGAVVGGLGGYMLGSAMSRPLIHFGNDYEDRYYRENMYRYPDQVYYRPVDQYSNQNNFVRDCVNITVKQHTVTTTTKGENFTETDMKIMERVVEQMCVTQYQKESEAYYQRGAS-

N I M W N N N H I V M Q EGRS

PrP sequence differs by 11 amino acids from the bank vole PrP (see in red)

No deletions

No insertions

No polymorphic variants found

Distinct primary sequence across species

135

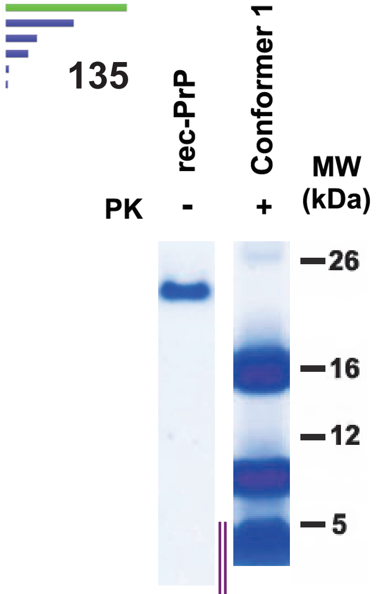

213 AA

1

Conformers

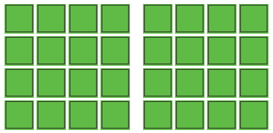

Tm^{Exp}: ND

ΔΔG: -6.06

In vitro studies

TgVole (1x) Not tested

African wild dog Not tested

In vivo studies

TgVole (1x) Not tested

African wild dog Not tested

pLDDT [85.29]

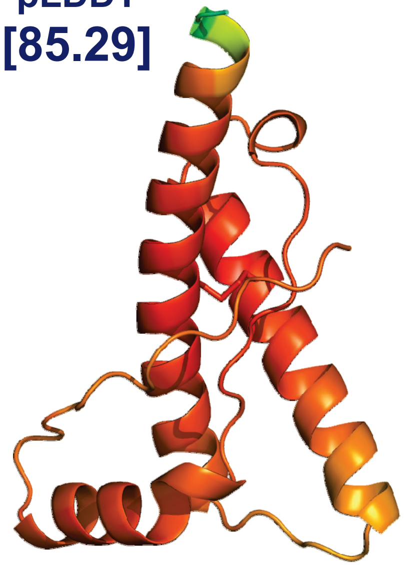

Protein code: 3183

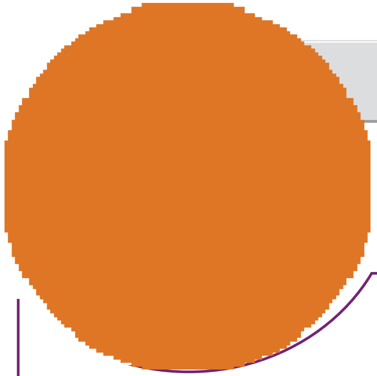

Iberian lynx

GenBank: **BK064941**

Lynx pardinus

Carnivora	36/41
Felidae	7/9

P31%

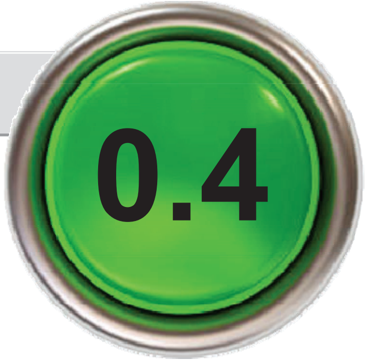

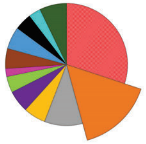

0-10

8 species sharing the same primary sequence

- **Cheetah** *Acinonyx jubatus*
- **Canada lynx** *Lynx canadensis*
- **Eurasian lynx** *Lynx lynx*
- **Leopard cat** *Prionailurus bengalensis euptilurus*
- **Iriomote cat** *Prionailurus iriomotensis*
- **Fishing cat** *Prionailurus viverrinus*
- **Cougar** *Puma concolor*
- **Jaguarundi** *Puma yagouaroundi*

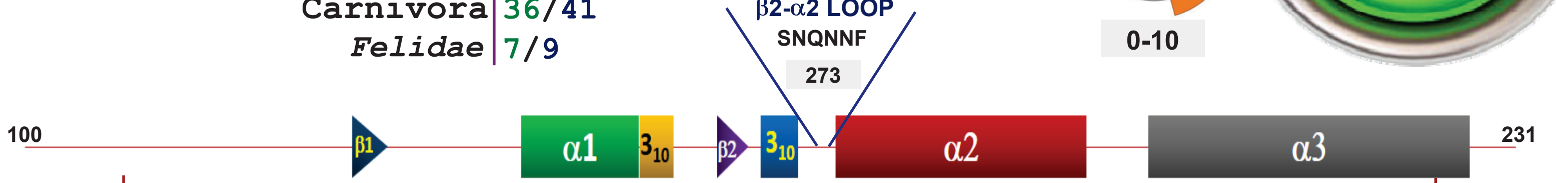

WGKPSKPKTNMKHMAGAAAAGAVVGGLGGYMLGSAMSRPLIHFGNDYEDRYYRENMYRYPNQVYYRPVDQYSNQNNFVHDCVNITVRQHTVTTTTKGENFTETDMKIMERVVEQMCVTQYQKESEAYYQRGAS-

N I V M W N N IK V M Q EGRS

PrP sequence differs by 11 amino acids from the bank vole PrP (see in red)

No deletions

No insertions

No polymorphic variants found

135

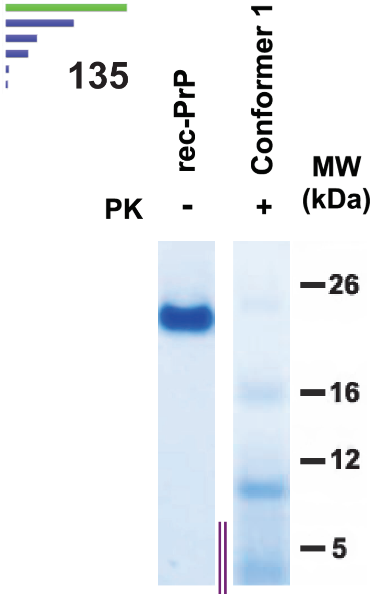

207 AA

1 Conformers

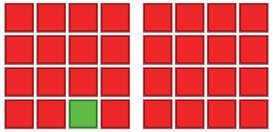

Tm^{Exp}: ND

ΔΔG: -8.12

In vitro studies

TgVole (1x)	Not tested
Iberian lynx	Not tested

In vivo studies

TgVole (1x)	Not tested
Iberian lynx	Not tested

pLDDT [85.48]

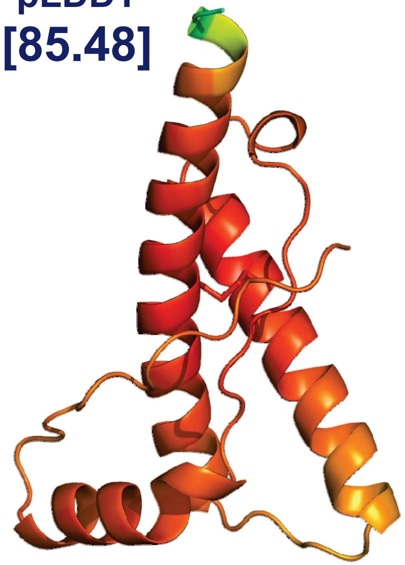

Protein code: 0977

Bobcat

GenBank: EU341503

Lynx rufus

Carnivora 36/41

Felidae 7/9

P87%

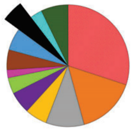

80-90

83.9

β2-α2 LOOP
SNQNNF
273

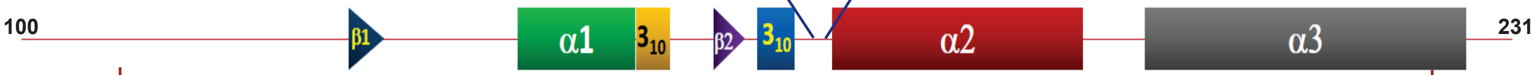

WGKPSKPKTNMKHMAGAAAAGAVVGGLGGYMLGSAMSRPLIHFGNDYEDRYYRENMYRYPNQVYYRPVDQYSNQNNFVHDCVNITVRQHTVTTTTKGENFTETDMKIMERVVEQMCVTQYQKEAEAYYQRGAS-

N I V M W N N IK V M SQ EGRS

PrP sequence differs by 12 amino acids from the bank vole PrP (see in red)

No deletions

No insertions

No polymorphic variants found

25

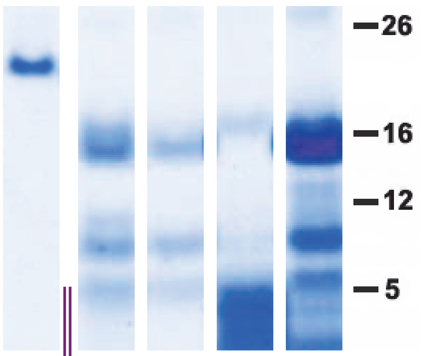

216 AA

4 Conformers

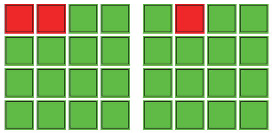

Tm^{Exp}: ND

ΔΔG: -6.74

pLDDT [85.81]

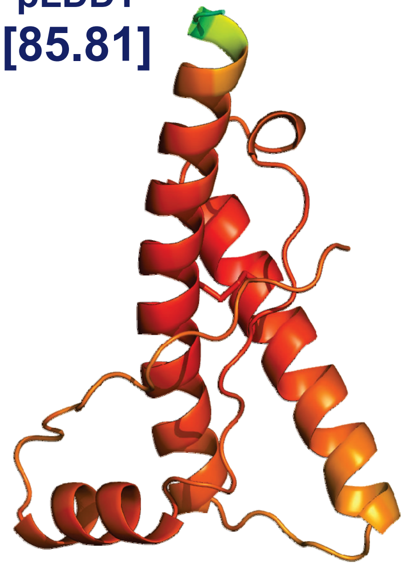

In vitro studies

TgVole (1x) Not tested

Bobcat Not tested

In vivo studies

TgVole (1x) Not tested

Bobcat Not tested

Distinct primary sequence across species

Protein code: 1333

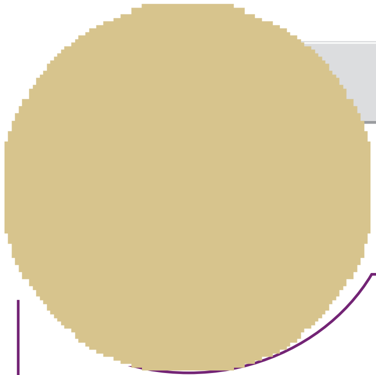

Honey badger

GenBank: BK064009

Mellivora capensis

Carnivora	36/41
Mustelidae	5/5

P100%

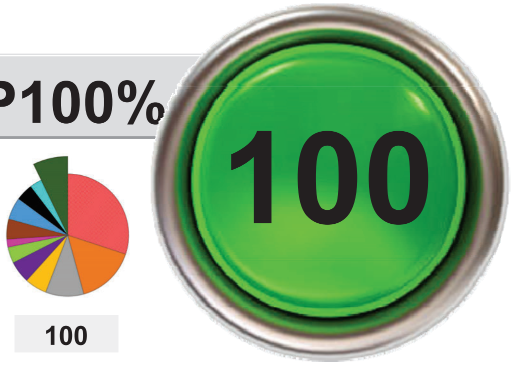

β2-α2 LOOP
SNQNNF
273

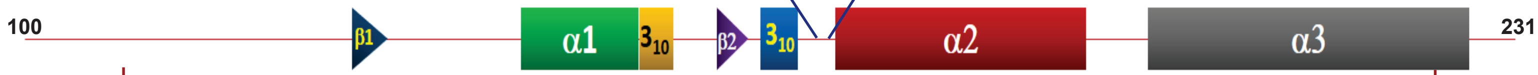

WGKPSKPKTNIKHMAGAAAAGAVVGGLGGYMLGSAMSRPLIHFGNDYEDRYYRENMHRYPDQVYYKPVDQYSNQNNFVHDCVNITVKQHTVTTTTKGENFTETDMKIMERVVEQMCVTQYQRESEAYYQRGAS-

N V M W N N R N I V M K Q EGRS

PrP sequence differs by 12 amino acids from the bank vole PrP (see in red)

No deletions
No insertions
No polymorphic variants found

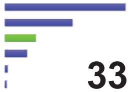

33

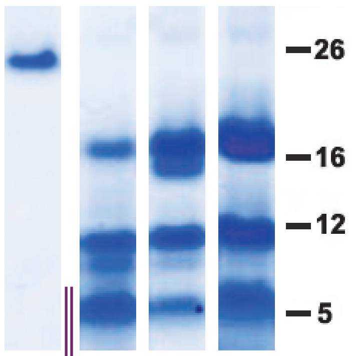

213 AA

3 Conformers

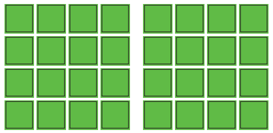

Tm[Exp]: ND

ΔΔG: -6.43

In vitro studies

TgVole (1x)	Not tested
Honey badger	Not tested

In vivo studies

TgVole (1x)	Not tested
Honey badger	Not tested

pLDDT [85.68]

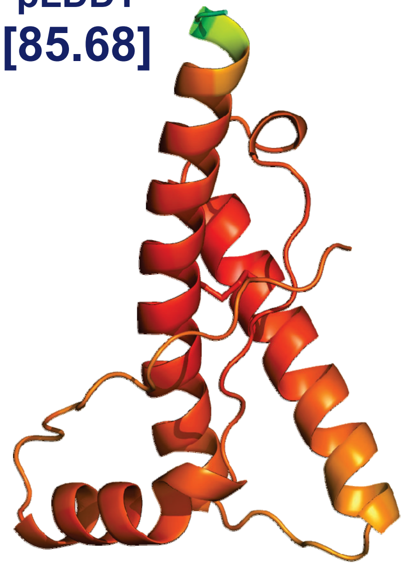

Distinct primary sequence across species

Protein code: 3170

Skunk

GenBank: EU341504

Mephitis mephitis

Carnivora 36/41
Mephitidae 2/2

P45%

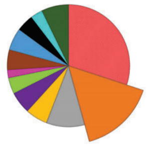

0-10

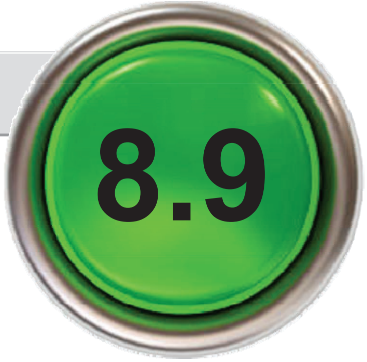

β2-α2 LOOP
SNQNNF
273

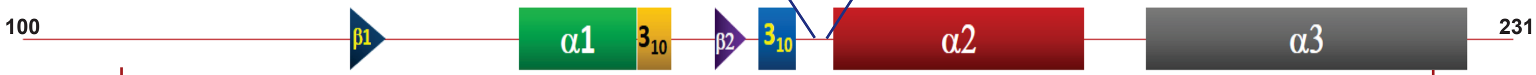

WGKPSKPKTNIKHMAGAAAAGAVVGGLGGYMLGSAMSRPLIHFGNDYEDRYYRDNMYRYPNQVYYKPVDQYSNQNNFVHDCVNITVRQHTVTTTTKGENFTETDMKIMERVVEQMCVTQYQRESEAYYQRGAS-
N V M W E N R N IK V M K Q EGRS

PrP sequence differs by 13 amino acids from the bank vole PrP (see in red)

No deletions
No insertions
No polymorphic variants found

Distinct primary sequence across species

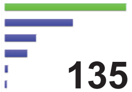

135

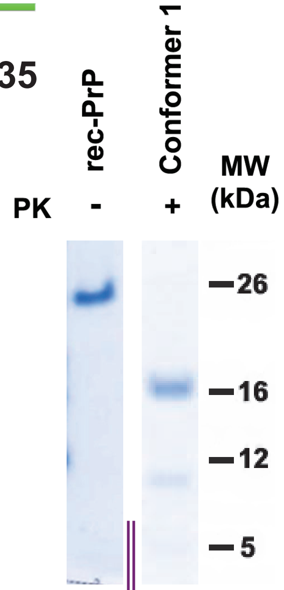

213 AA

1
Conformers

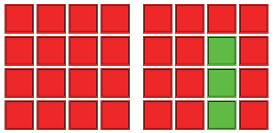

Tm[Exp]: ND

ΔΔG: -4.03

In vitro studies

TgVole (1x) Ongoing

Skunk Ongoing

In vivo studies

TgVole (1x) Ongoing

Skunk Not tested

pLDDT
[85.04]

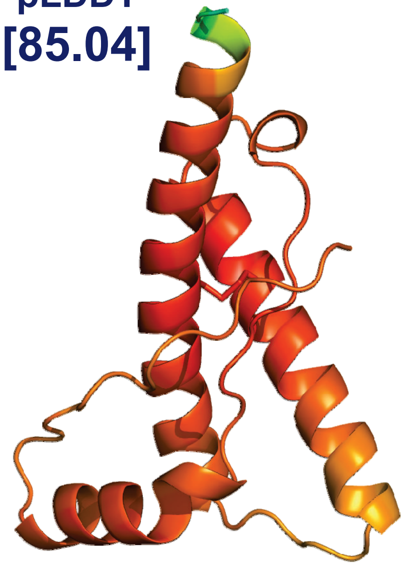

Protein code: 0538

Southern elephant seal

GenBank: XM_035005902

Mirounga leonina

Carnivora 36/41
Phocidae 4/6

P75% **62.5**

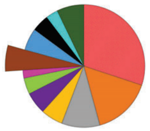

Northern elephant seal
Mirounga angustirostris

1 species sharing the same primary sequence

β2-α2 LOOP
SSQNNF
42

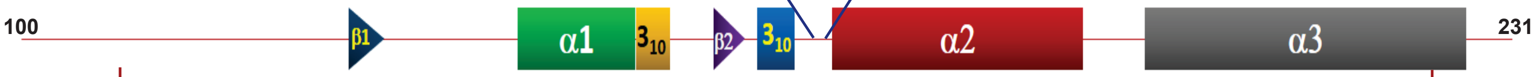

WGKPNKPKTNMKHMAGAAAAGAVVGGLGGYMLGSAMSRPLIHFGNDYEDRYYRENMYRYPNQVYYKPVDQYSSQNNFVHDCVNITVKQHTVTTTTKGENFTETDMKIMERVVEQMCVSQYQRESEAYYQRGAS-
N S I V M W N R NN I V M T K Q EGRS

PrP sequence differs by 14 amino acids from the bank vole PrP (see in red)

No deletions
No insertions
No polymorphic variants found

33

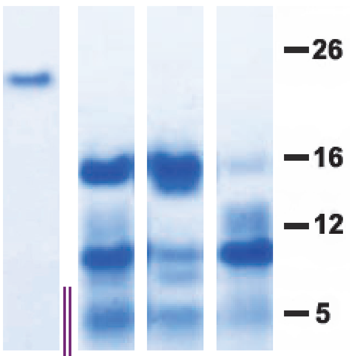

213 AA

3 Conformers

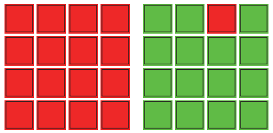

Tm^{Exp}: ND

ΔΔG: -12.06

In vitro studies

TgVole (1x) Not tested

Southern elephant seal Not tested

In vivo studies

TgVole (1x) Not tested

Southern elephant seal Not tested

pLDDT
[85.82]

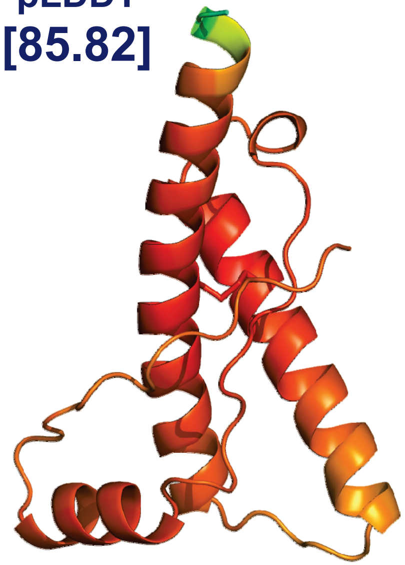

Protein code: 2188

Hawaiian monk seal

GenBank: XM_021689157

Monachus schauinslandi

Carnivora 36/41
Phocidae 4/6

P58%

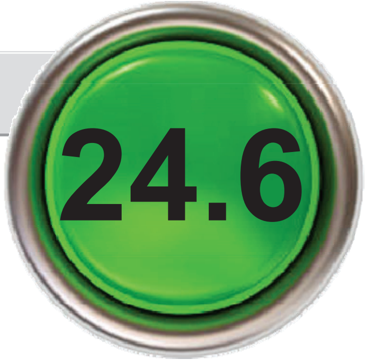

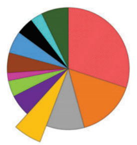

20-30

β2-α2 LOOP
SSQNNF
42

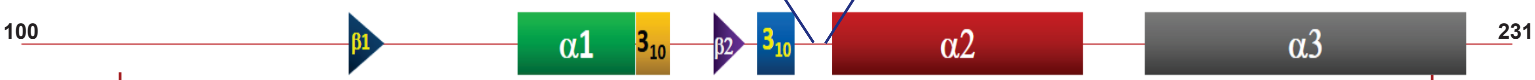


```
WGKPSKPKTNMKHMAGAAAAGAVVGGLGGYMLGSAMSRPLIHFGNDYEDRYYRENMYRYPNQVYYKPVDQYSSQNNFVHDCVNITVKQHTVTTTTKGENFTETDMKIMERVVEQMCVSQYQRESEAYYQRGAS-
 N         I  V                            M      W         N        R      NN           I                V M           T   K  Q   EGRS
```

PrP sequence differs by 14 amino acids from the bank vole PrP (see in red)

No deletions
No insertions
No polymorphic variants found

Distinct primary sequence across species

76

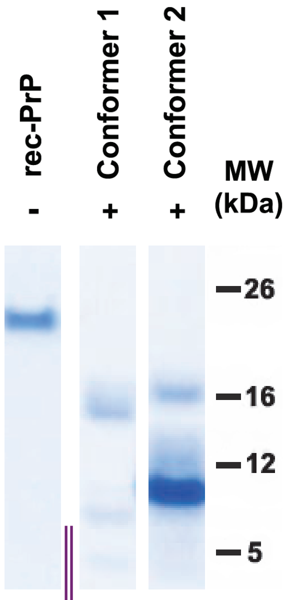

213 AA

2 Conformers

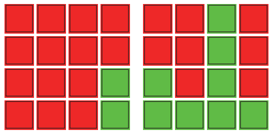

Tm[Exp]: ND

ΔΔG: -10.71

In vitro studies

TgVole (1x) — Not tested

Hawaiian monk seal — Not tested

In vivo studies

TgVole (1x) — Not tested

Hawaiian monk seal — Not tested

pLDDT [85.69]

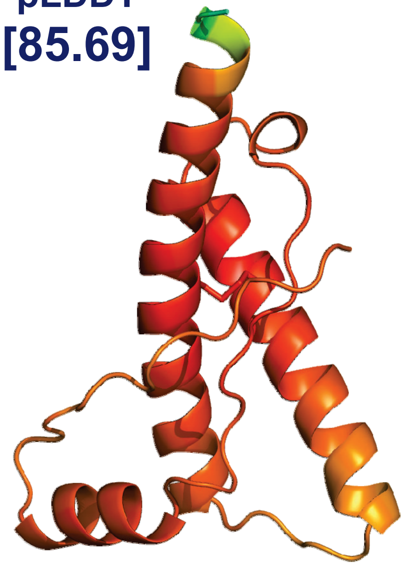

Protein code: 1112

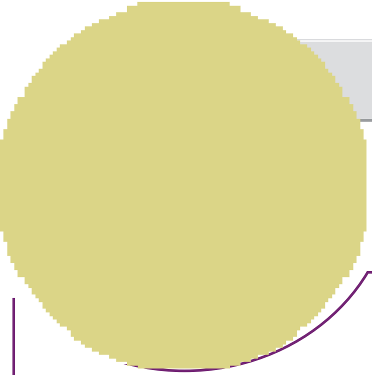

Banded mongoose

GenBank: BK064099

Mungos mungo

Carnivora	36/41
Herpestidae	4/4

β2-α2 LOOP
SSQNNF
42

P57%

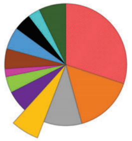

20-30

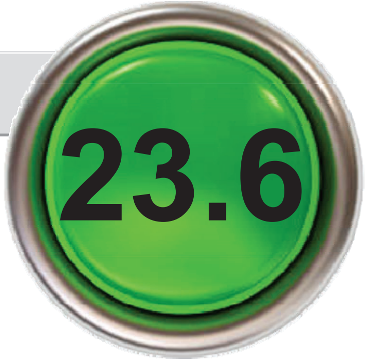

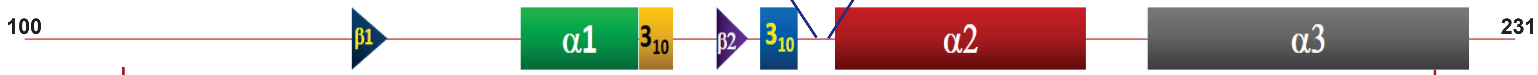

WGKPSKPKTSMKHVAGAAAAGAVVGGLGGYMLGSAMSRPIIHFGNDYEDRYYRQNMDRYPNQVYYRPVDQYSSQNNFVHDCVNITVRQHTVTTTTKGENFTETDMKIMERVVEQMCVTQYEKESQAYYQRGAS-

N NI M W E N NN IK V M Q EGRS

PrP sequence differs by 13 amino acids from the bank vole PrP (see in red)

No deletions
No insertions
No polymorphic variants found

Distinct primary sequence across species

76

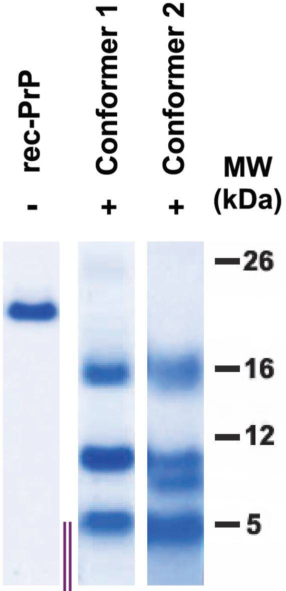

209 AA

2
Conformers

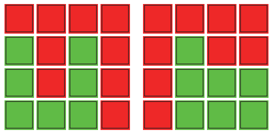

Tm^{Exp}: ND

ΔΔG: -9.1

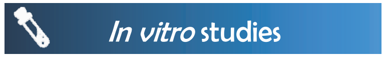

TgVole (1x) — Not tested

Banded mongoose — Not tested

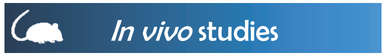

TgVole (1x) — Not tested

Banded mongoose — Not tested

pLDDT
[84.98]

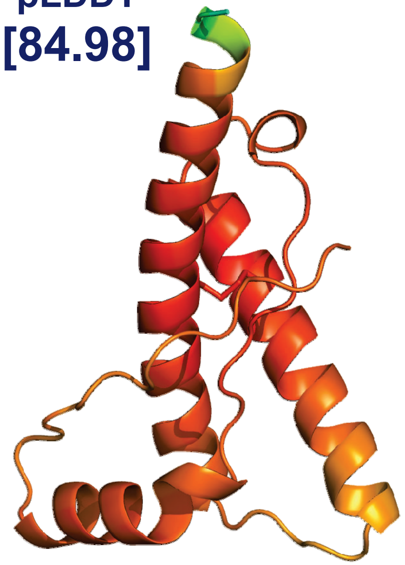

Stoat

GenBank: EU341505

Mustela erminea

Carnivora	36/41
Mustelidae	5/5

P92%

94.3

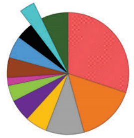

90-100

β2-α2 LOOP
SNQNNF
273

100 — β1 — α1 — 3_{10} — β2 — 3_{10} — α2 — α3 — 231

```
WGKPSKPKTNMKHVAGAAAAGAVVGGLGGYMLGSAMSRPLIHFGNDYEDRYYRENMYRYPNQVYYKPVDQYSNQNNFVHDCVNITVKQHTVTTTTKGENFTETDMKIMERVVEQMCVTQYQRESEAYYQRGAS-
 N        I                           M      W          N         R      N           I                      V M                 K  Q  EGRS
```

PrP sequence differs by 11 amino acids from the bank vole PrP (see in red)

No deletions

No insertions

No polymorphic variants found

14 species sharing the same primary sequence

- **Raccoon** *Procyon lotor*
- **Stoat** *Mustela erminea*
- **Sea otter** *Enhydra lutris*
- **European otter** *Lutra lutra*
- **North American river otter** *Lontra canadensis*
- **Giant otter** *Pteronura brasiliensis*
- **European mink** *Mustela lutreola*
- **Least weasel** *Mustela nivalis*
- **South American coati** *Nasua nasua*
- **Asian small-clawed otter** *Aonyx cinerea*
- **Ring-tailed cat** *Bassariscus astutus*
- **Cacomistle** *Bassariscus sumichrasti*
- **White-nosed coati** *Nasua narica*
- **Black-footed ferret** *Mustela nigripes*

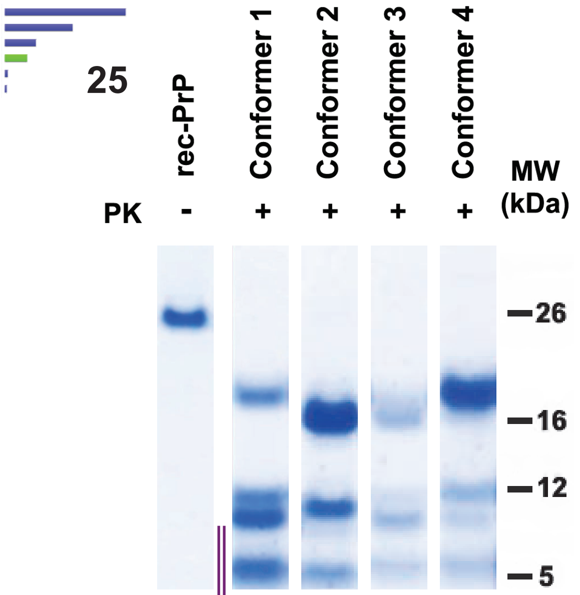

213 AA

4 Conformers

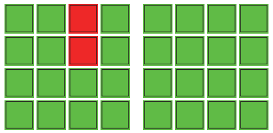

Tm^{Exp}: ND

ΔΔG: -8.8

***In vitro* studies**

TgVole (1x)	Not tested
Stoat	Not tested

***In vivo* studies**

TgVole (1x)	Not tested
Stoat	Not tested

pLDDT [87.3]

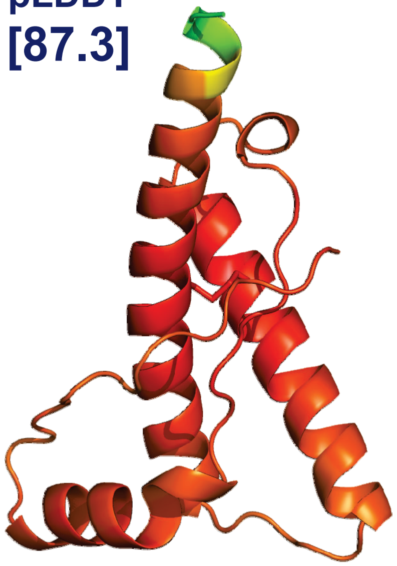

American mink

GenBank: EF508270

Neovison vison

Carnivora 36/41

Mustelidae 5/5

P81%

71.4

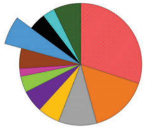

β2-α2 LOOP
SNQNNF
273

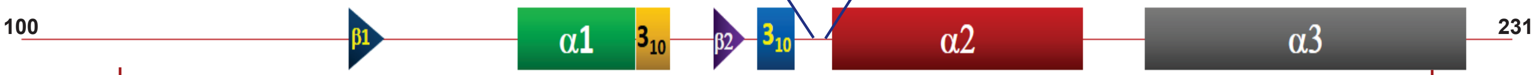

WGKPSKPKTNMKHVAGAAAAGAVVGGLGGYMLGSAMSRPLIHFGNDYEDRYYRENMYRYPNQVYYKPVDQYSNQNNFVHDCVNITVKQHTVTTTTKGENFTETDMKIMERVVEQMCVTQYQRESEAYYQKGAS-

N I M W N R N I V M K Q EGRS

PrP sequence differs by 11 amino acids from the bank vole PrP (see in red)

No deletions

No insertions

No polymorphic variants found

Distinct primary sequence across species

135

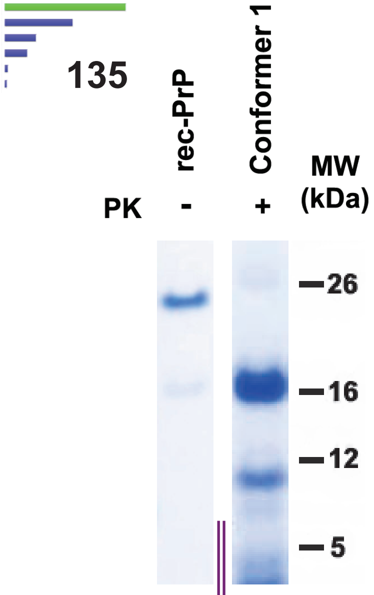

213 AA

1

Conformers

Tm^{Exp}: ND

ΔΔG: -6.64

In vitro studies

Propagate
TgVole (1x)

Ongoing
TgAmerican mink

In vivo studies

Infectious
TgVole (1x)

Not tested
TgAmerican mink

pLDDT [85.39]

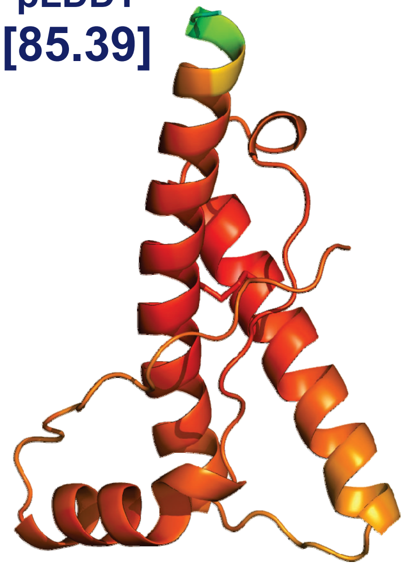

Protein code: 0180

Walrus

GenBank: EU341508

Odobenus rosmarus

Carnivora 36/41
Odobenidae 1/1

P42%

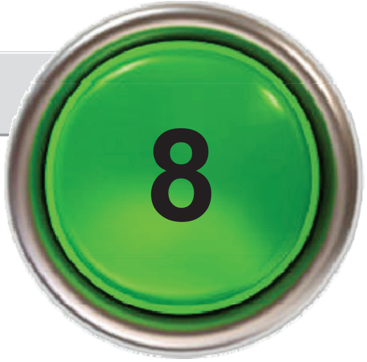

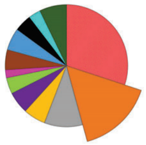

0-10

β2-α2 LOOP
SNQNNF
273

100

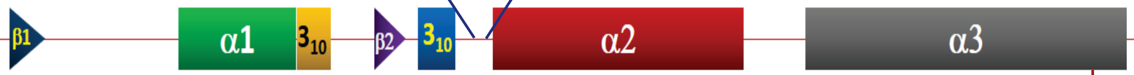

231

```
WGKPNKPKTNMKHMAGAAAAGAVVGGLGGYMLGSAMSRPLIHFGNDYEDRYYRENMNRYPNQVYYKPVDQYSNQNNFVHDCVNITVKQHTVTTTTKGENFTETDMKIMERVVEQMCVTQYQRESEAYYQRGAS-
 N  S    I  V                              M       W                    R     N               I                    V M                  K  Q  EGRS
```

PrP sequence differs by 11 amino acids from the bank vole PrP (see in red)

No deletions
No insertions
No polymorphic variants found

7 species sharing the same primary sequence

Australasian fur seal
Arctocephalus forsteri

Brown fur seal
Arctocephalus pusillus

Guadalupe fur seal
Arctocephalus townsendi

Northern fur seal
Callorhinus ursinus

Steller sea lion
Eumetopias jubatus

Sea lion
Otaria flavescens

California sea lion
Zalophus californianus

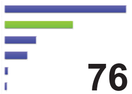

76

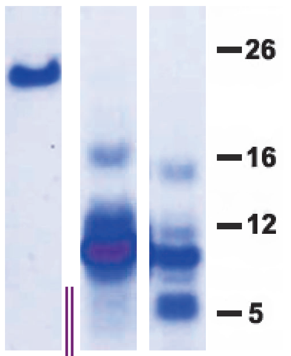

213 AA

2
Conformers

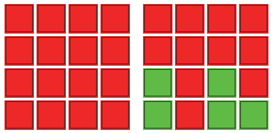

Tm^{Exp}: ND

ΔΔG: -12.13

In vitro studies

TgVole (1x)	Not tested
Walrus	Not tested

In vivo studies

TgVole (1x)	Not tested
Walrus	Not tested

pLDDT
[87.15]

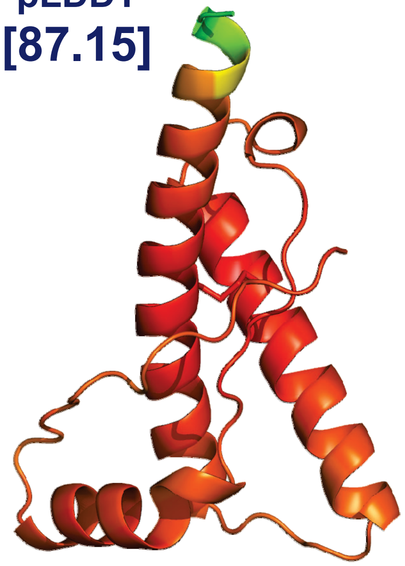

Protein code: 0547

Lion

GenBank: EU236260

Panthera leo

Carnivora 36/41
Felidae 7/9

P84%

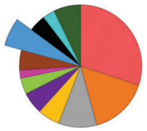

70-80

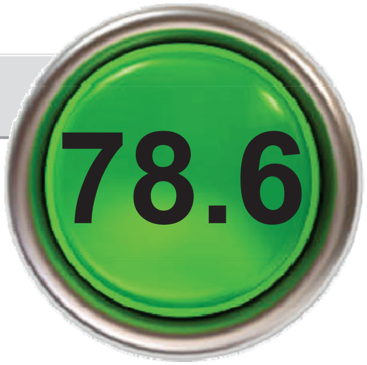

6 species sharing the same primary sequence

- **Sunda clouded leopard** *Neofelis diardi*
- **Clouded leopard** *Neofelis nebulosa*
- **Jaguar** *Panthera onca*
- **Leopard** *Panthera pardus*
- **Siberian tiger** *Panthera tigris*
- **Snow leopard** *Panthera uncia*

β2-α2 LOOP
SNQNNF
273

100 β1 α1 3_{10} β2 3_{10} α2 α3 231

WGKPSKPKTNMKHMAGAAAAGAVVGGLGGYMLGSAMNRPLIHFGNDYEDRYYRENMYRYPNQVYYRPVDQYSNQNNFVHDCVNITVRQHTVTTTTKGENFTETDMKIMERVVEQMCVTQYQKESEAYYQRGAS-
N I V S M W N N IK V M Q EGRS

PrP sequence differs by 12 amino acids from the bank vole PrP (see in red)

No deletions
No insertions
No polymorphic variants found

4

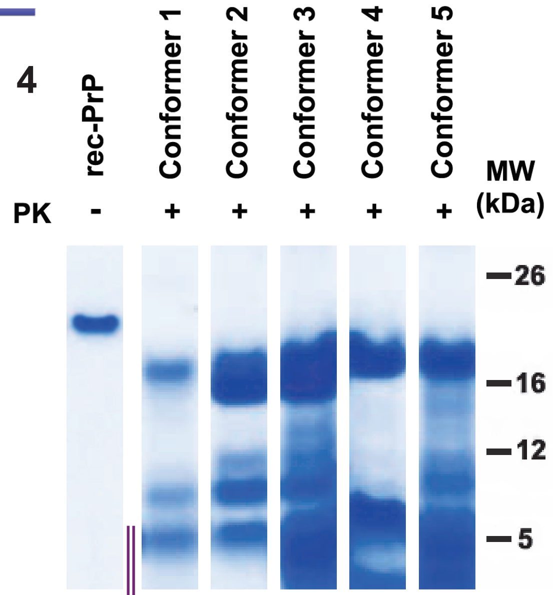

207 AA

5 Conformers

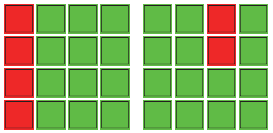

Tm^{Exp}: ND

ΔΔG: -6.42

***In vitro* studies**

TgVole (1x)	Not tested
Lion	Not tested

***In vivo* studies**

TgVole (1x)	Not tested
Lion	Not tested

pLDDT [85.76]

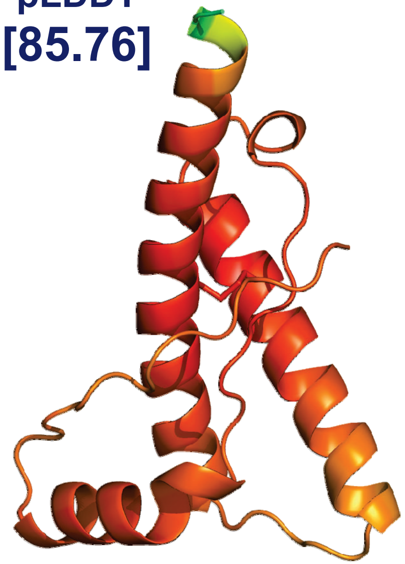

Protein code: 1085

Common seal

GenBank: EU341509

P47%

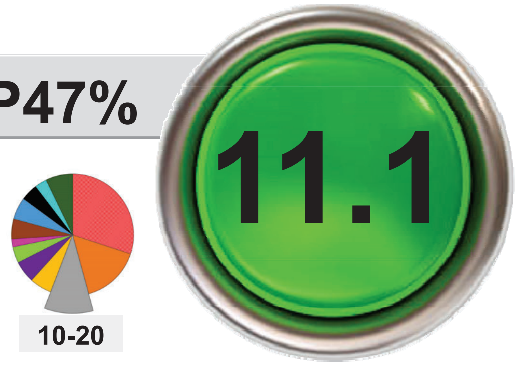

Phoca vitulina

Carnivora 36/41
Phocidae 4/6

5 species sharing the same primary sequence

Hooded seal *Cystophora cristata*

Grey seal *Halichoerus grypus*

Harp seal *Pagophilus groenlandicus*

Spotted seal *Phoca largha*

Baikal seal *Pusa sibirica*

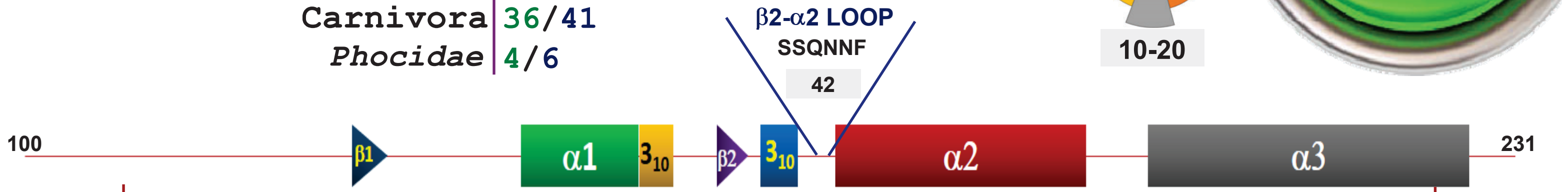

WGKPNKPKTNMKHMAGAAAAGAVVGGLGGYMLGSAMSRPLIHFGNDYEDRYYRENMYRYPNQVYYKPVDQYSSQNNFVHDCVNITVKQHTVTTTTKGENFTETDMKIMERVVEQMCVTQYQKESEAYYQRGAS-

N S I V M W N R NN I V M Q EGRS

PrP sequence differs by 12 amino acids from the bank vole PrP (see in red)

No deletions

No insertions

No polymorphic variants found

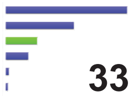

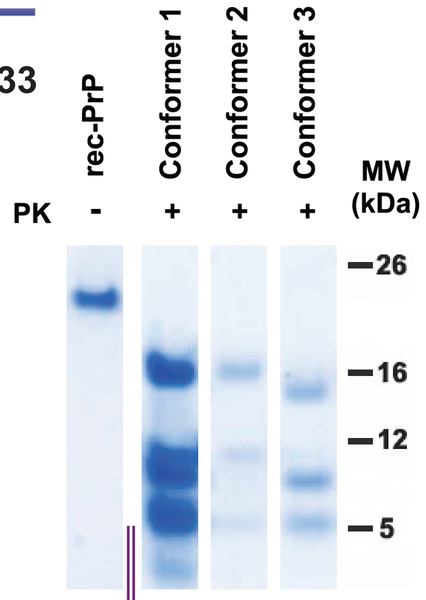

213 AA

3 Conformers

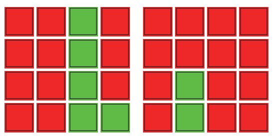

Tm^{Exp}: ND

ΔΔG: -9.87

pLDDT [85.59]

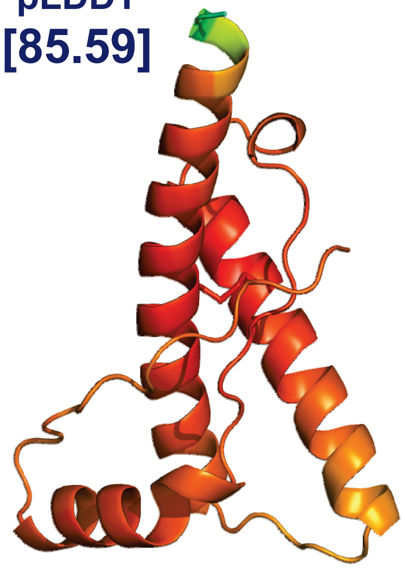

In vitro studies

TgVole (1x)	Not tested
Common seal	Not tested

In vivo studies

TgVole (1x)	Not tested
Common seal	Not tested

Protein code: 0929

Kinkajou

GenBank: BK063964

P64% **33.9**

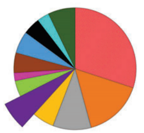

30-40

Potos flavus

Carnivora 36/41
Procyonidae 1/1

β2-α2 LOOP
SNQNNF
273

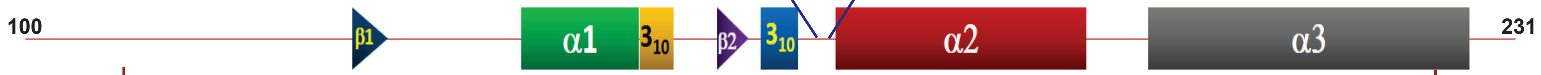

WGKPSKPKTNMKHVAGAAAAGAVVGGLGGYMLGSAMSRPLIHFGNDYEDRYYRENMYRYPNQVYYKPVDQYSNQNNFVHDCVNITVRQHTVTTTTKGENFTETDMKIMERVVEQMCVTQYQRESEAYYQRGAS-
N I M W N R N IK V M K Q EGRS

PrP sequence differs by 12 amino acids from the bank vole PrP (see in red)

No deletions
No insertions
No polymorphic variants found

Distinct primary sequence across species

33

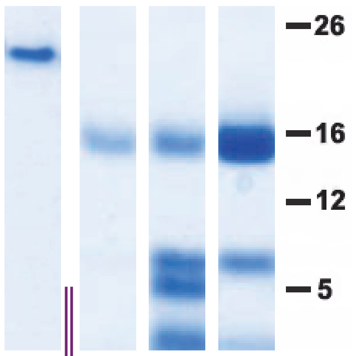

213 AA

3 Conformers

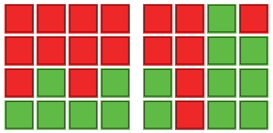

Tm^{Exp}: ND

ΔΔG: -6.27

In vitro studies

TgVole (1x)	Not tested
Kinkajou	Not tested

In vivo studies

TgVole (1x)	Not tested
Kinkajou	Not tested

pLDDT [86.17]

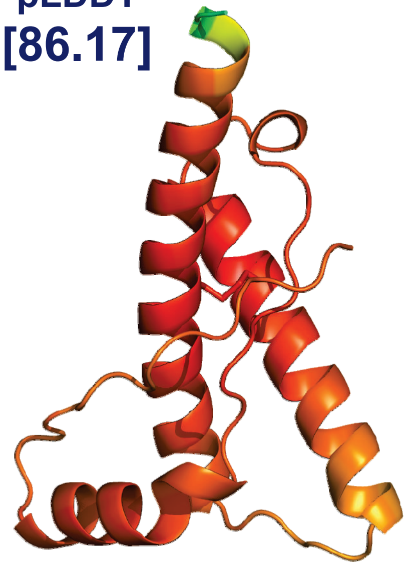

Protein code: 3094

Aardwolf

GenBank: BK064008

Proteles cristata

Carnivora 36/41

Hyaenidae 1/2

P73%

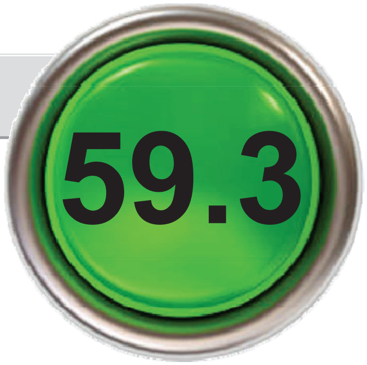

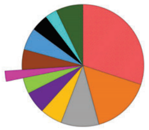

50-60

β2-α2 LOOP
SSQNNF
42

100 β1 α1 3_{10} β2 3_{10} α2 α3 231

```
WGKPSKPKTNMKHVAGAAAAGAVVGGLGGYMLGSAMSRPLIHFGNDYEDRYYRQNMDRYPNQVYYKPVDQYSSQNNFVHDCVNITVRQHTVTTTTKGENFTETDMKIMERVVEQMCVTQYQRESEAYYQRGAS-
 N        I                            M      W     E  N        R     NN            IK                 V M                K   Q  EGRS
```

PrP sequence differs by 14 amino acids from the bank vole PrP (see in red)

No deletions

No insertions

No polymorphic variants found

33

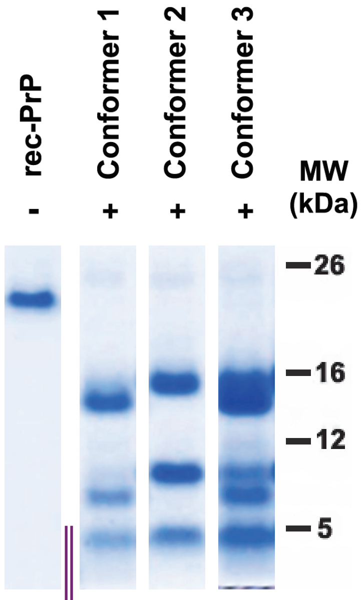

213 AA

3
Conformers

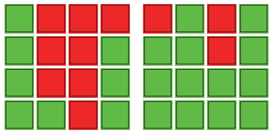

Tm^{Exp}: ND

ΔΔG: -10.08

In vitro studies

TgVole (1x) Not tested

Aardwolf Not tested

In vivo studies

TgVole (1x) Not tested

Aardwolf Not tested

pLDDT
[87.05]

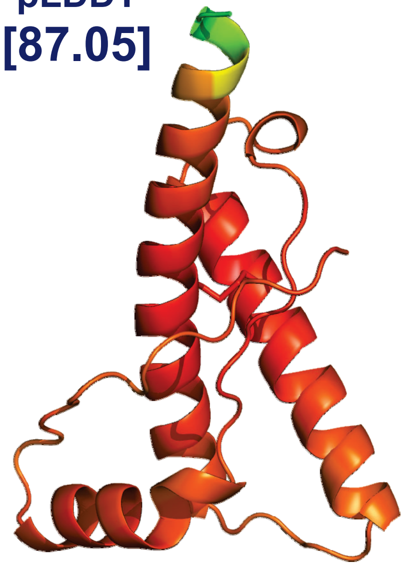

Distinct primary sequence across species

Protein code: 3169

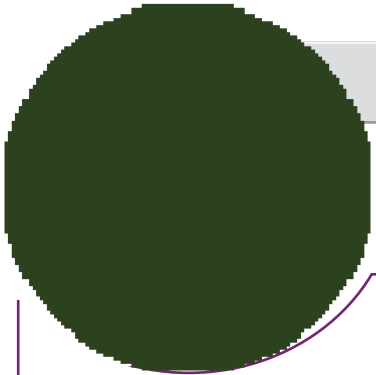

Ringed seal

GenBank: BK064218

Pusa hispida saimensis

Carnivora	36/41
Phocidae	4/6

P29%

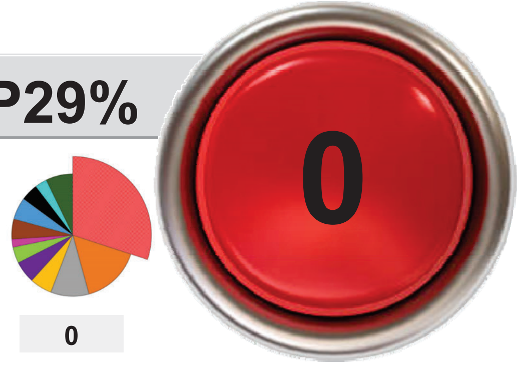

β2-α2 LOOP
SSQNNF
42

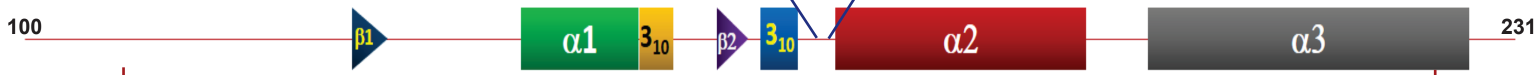


```
WGKPNKPKTNMKHMAGAAAAGAVVGGLGGYMLGSAMSRPLVHFGNDYEDRYYRENMYRYPNQVYYKPVDQYSSQNNFVHDCVNITVKQHTVTTTTKGENFTETDMKIMERVVEQMCVTQYQKESEAYYQRGAS-
 N  S     I  V                             MI     W          N         R     NN              I                    V M                 Q   EGRS
```

PrP sequence differs by 13 amino acids from the bank vole PrP (see in red)

No deletions
No insertions
No polymorphic variants found

Distinct primary sequence across species

Tm^{Exp}: ND

ΔΔG: -14.83

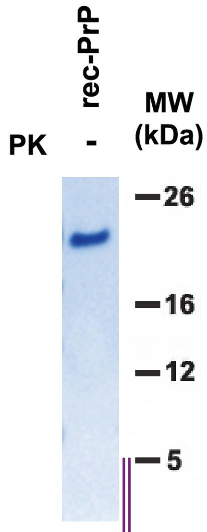

213 AA

NO MISFOLDING

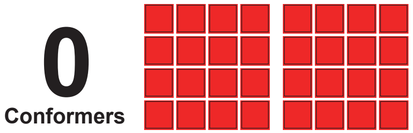

In vitro studies

NOT APPLICABLE

In vivo studies

NOT APPLICABLE

pLDDT
[92.7]

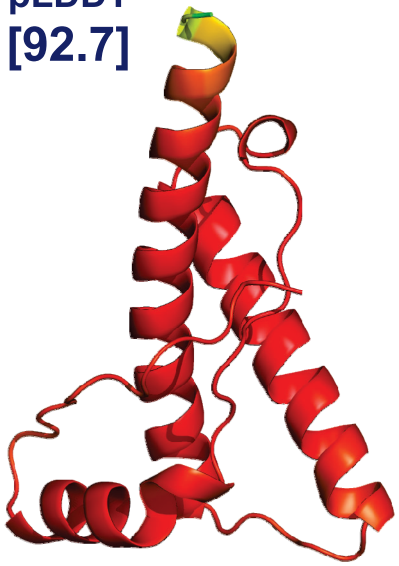

Protein code: 4786

Western spotted skunk

GenBank: BK064000

Spilogale gracilis

Carnivora 36/41

Mephitidae 2/2

P53% **18.2**

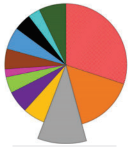

β2-α2 LOOP
SNQNNF
273

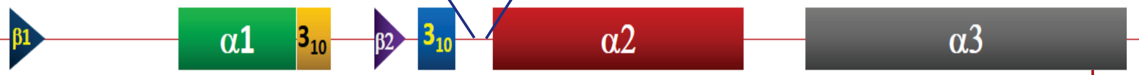

WGKPSKPKTNIKHVAGAAAAGAVVGGLGGYMLGSAMSRPLIHFGNDYEDRYYRDNMYRYPNQVYYKPVDQYSNQNNFVHDCVNITVRQHTVTTTTKGENFTETDMKIMERVVEQMCVTQYQRESEAYYQRGAS-

N M W E N R N IK V M K Q EGRS

PrP sequence differs by 12 amino acids from the bank vole PrP (see in red)

No deletions

No insertions

No polymorphic variants found

1 species sharing the same primary sequence

Eastern spotted skunk
Spilogale putorius interrupta

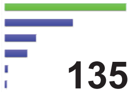

135

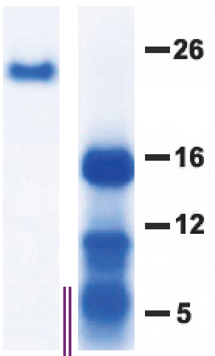

213 AA

1 Conformers

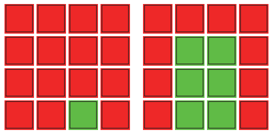

Tm^{Exp}: ND

ΔΔG: -1.74

In vitro studies

TgVole (1x) Not tested

Western spotted skunk Not tested

In vivo studies

TgVole (1x) Not tested

Western spotted skunk Not tested

pLDDT [85.27]

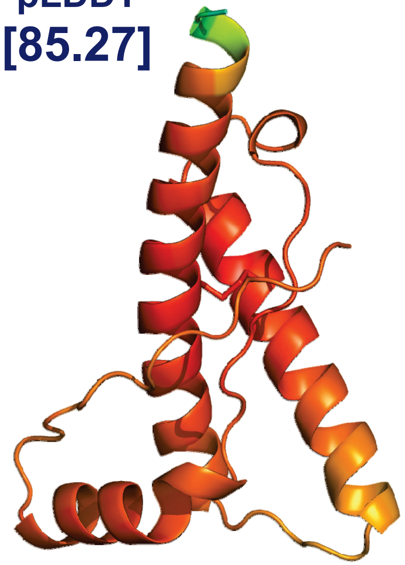

Protein code: 3161

Meerkat

GenBank: XP_029772432

Suricata suricatta

Carnivora	36/41
Herpestidae	4/4

P89%

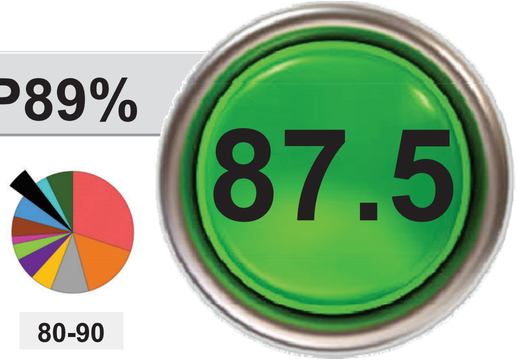

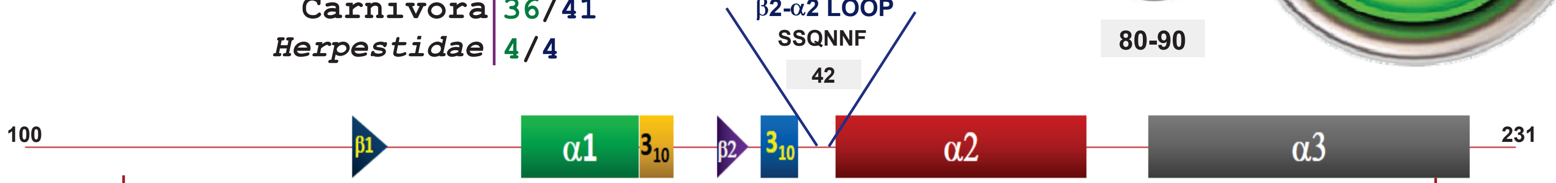

WGKPSKPKTNMKHVAGAAAAGAVVGGLGGYMLGSAMSRPIIHFGNDYEDRYYRQNMDRYPNQVYYRPVDQYSSQNNFVHDCVNITVRQHTVTTTTKGENFTETDMKIMERVVEQMCVTQYQKESQAYYQRGAS-

N I M W E N NN IK V M EGRS

PrP sequence differs by 11 amino acids from the bank vole PrP (see in red)

No deletions

No insertions

No polymorphic variants found

Distinct primary sequence across species

76

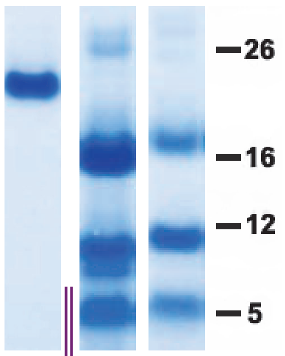

209 AA

2 Conformers

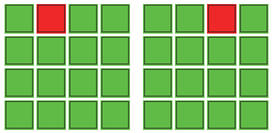

Tm^{Exp}: ND

ΔΔG: -9.09

pLDDT [85.16]

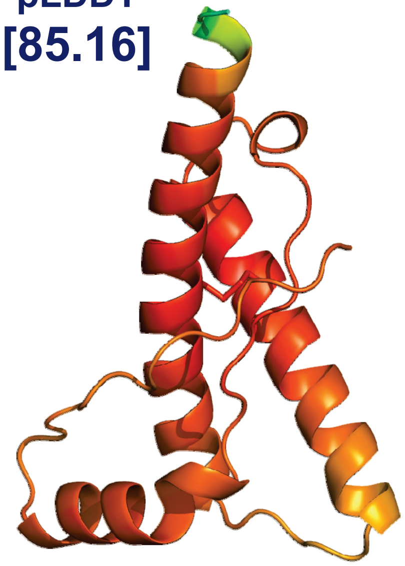

In vitro studies

TgVole (1x)	Not tested
Meerkat	Not tested

In vivo studies

TgVole (1x)	Not tested
Meerkat	Not tested

Protein code: 0939

Spectacled bear

GenBank: BK064152

Tremarctos ornatus

Carnivora	36/41
Ursidae	2/2

P73%

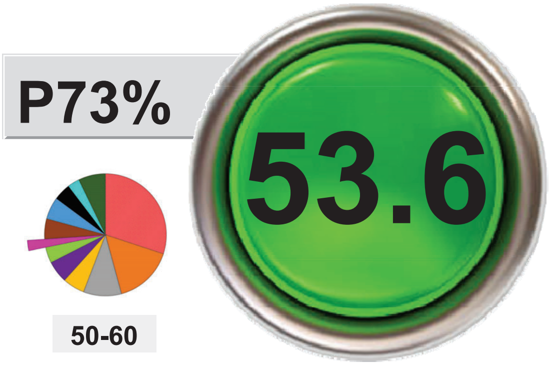

β2-α2 LOOP
SNQNSF
36

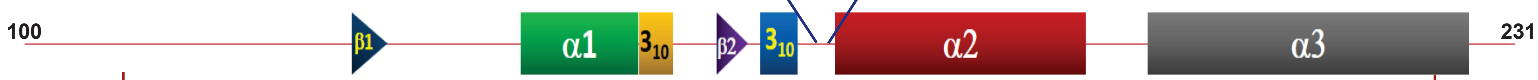

WGKPSKPKTNMKHVAGAAAAGAVVGGLGGYMLGSAMSRPLIHFGNDYEDRYYRENMYRYPNQVYYKPVDQYSNQNSFVHDCVNITVKQHTVTTTTKGENFTETDIKIMERVVEQMCVTQYQREAEAYYQRGAS-

N I M W N R N N I V M K SQ EGRS

PrP sequence differs by 13 amino acids from the bank vole PrP (see in red)

No deletions

No insertions

No polymorphic variants found

Distinct primary sequence across species

135

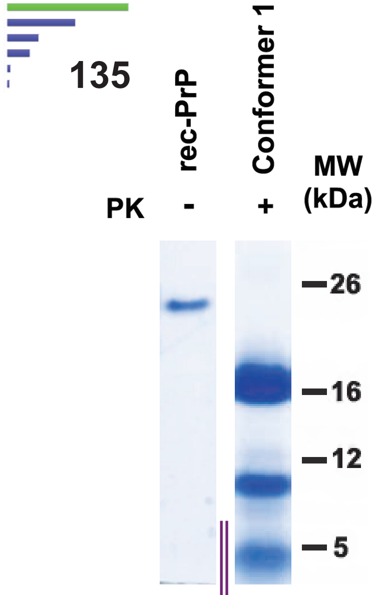

213 AA

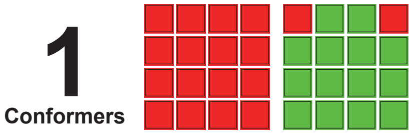

Tm[Exp]: ND

ΔΔG: -10.4

In vitro studies

TgVole (1x) Not tested

Spectacled bear Not tested

In vivo studies

TgVole (1x) Not tested

Spectacled bear Not tested

pLDDT
[86.62]

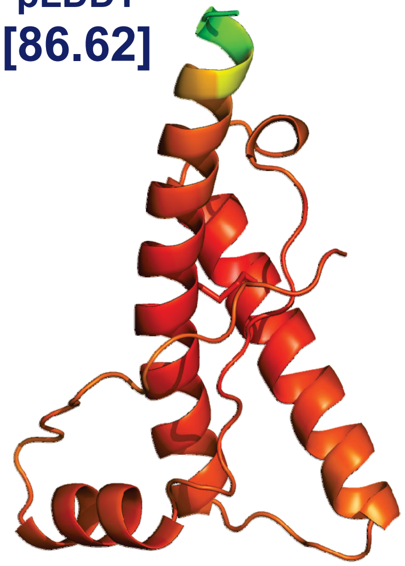

Protein code: 3583

Brown bear

GenBank: JX218961

Ursus arctos

Carnivora 36/41

Ursidae 2/2

5 species sharing the same primary sequence

Giant panda *Ailuropoda melanoleuca*

Sun bear *Helarctos malayanus*

American black bear *Ursus americanus*

Polar bear *Ursus maritimus*

Asian black bear *Ursus thibetanus thibetanus*

P69%

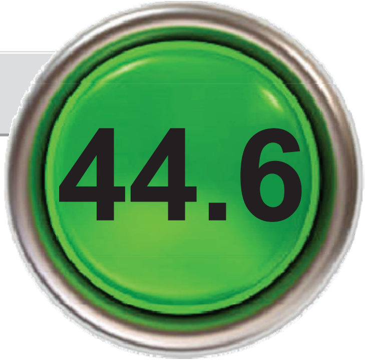

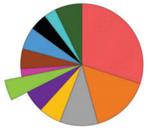

40-50

β2-α2 LOOP

SNQNSF

36

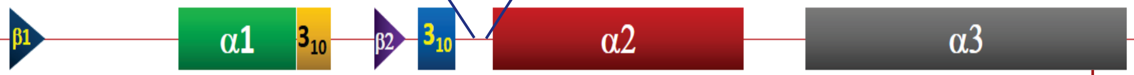


```
WGKPSKPKTNMKHVAGAAAAGAVVGGLGGYMLGSAMSRPLIHFGNDYEDRYYRENMYRYPNQVYYKPVDQYSNQNSFVHDCVNITVKQHTVTTTTKGENFTETDIKIMERVVEQMCVTQYQREAEAYYQRGAS-
  N          I                             M     W       N       R     N  N       I                      V M            K SQ  EGRS
```

PrP sequence differs by 13 amino acids from the bank vole PrP (see in red)

No deletions

No insertions

No polymorphic variants found

135

213 AA

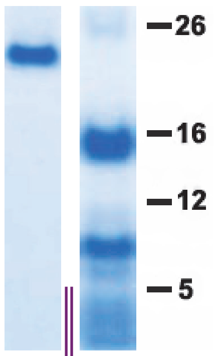

1 Conformers

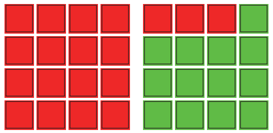

Tm^{Exp}: ND

ΔΔG: -10.67

pLDDT [84.84]

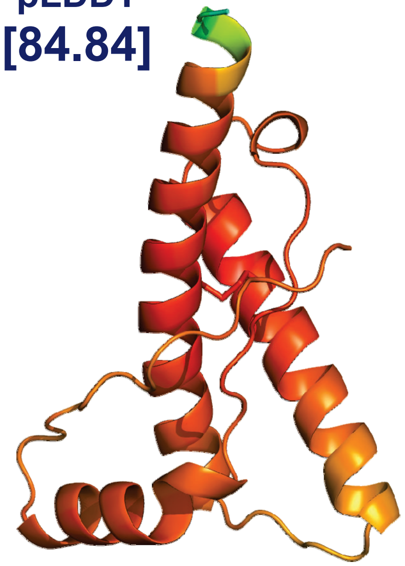

In vitro studies

TgVole (1x) Not tested

Brown bear Not tested

In vivo studies

TgVole (1x) Not tested

Brown bear Not tested

Protein code: 0927

Red fox

GenBank: XM_026012328

Vulpes vulpes

Carnivora 36/41
Canidae 5/5

P78% **67.9**

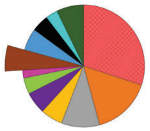

60-70

β2-α2 LOOP
SNQNNFF
273

100 β1 α1 3_{10} β2 3_{10} α2 α3 231

```
WGKPNKPKTNMKHVAGAAAAGAVVGGLGGYMLGSAMSRPLIHFGNDYEDRYYRENMYRYPDQVYYRPVDQYSNQNNFVRDCVNITVKQHTVTTTTKGENFTETDMKIMERVVEQMCVTQYQKESEAYYQRGAS-
 N S  I  M  W  N  N  N  H  I  V M  Q  EGRS
```

PrP sequence differs by 11 amino acids from the bank vole PrP (see in red)

No deletions
No insertions
No polymorphic variants found

9 species sharing the same primary sequence

- **Side-striped jackal** *Canis adustus*
- **Raccoon dog** *Nyctereutes procyonoides*
- **Bat-eared fox** *Otocyon megalotis*
- **Gray fox** *Urocyon cinereoargenteus*
- **Island fox** *Urocyon littoralis*
- **Corsac fox** *Vulpes corsac*
- **Arctic fox** *Vulpes lagopus*
- **Swift fox** *Vulpes velox*
- **Fennec fox** *Vulpes zerda*

76

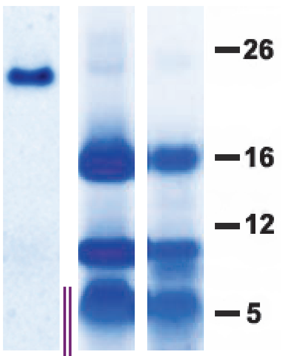

213 AA

2 Conformers

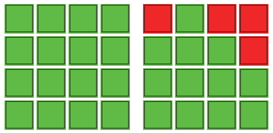

Tm[Exp]: ND
ΔΔG: -8.8

In vitro studies

TgVole (1x)	Not tested
Red fox	Not tested

In vivo studies

TgVole (1x)	Not tested
Red fox	Not tested

pLDDT [91.75]

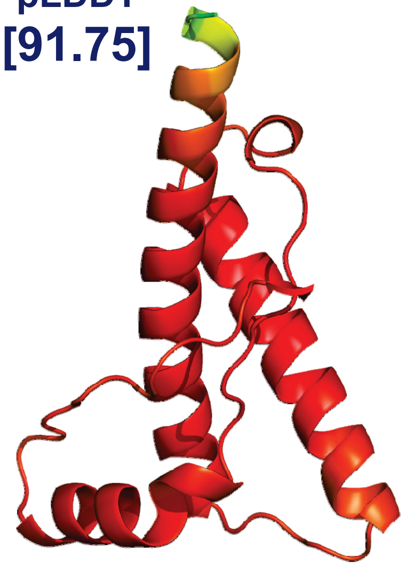

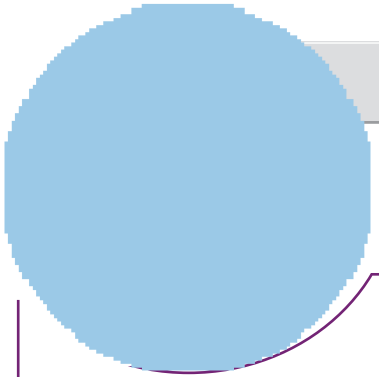

Whale

GenBank: DQ884475

Balaenoptera physalus

Cetacea	0/14
Balaenopteridae	0/1

Distinct primary sequence across species

P29%

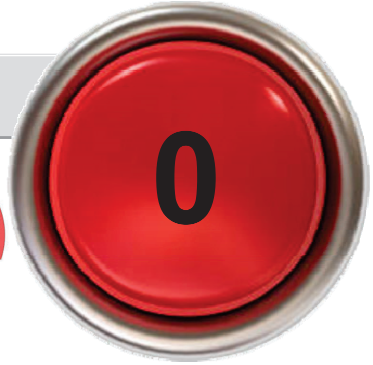

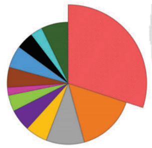

β2-α2 LOOP
NSQNSF
5

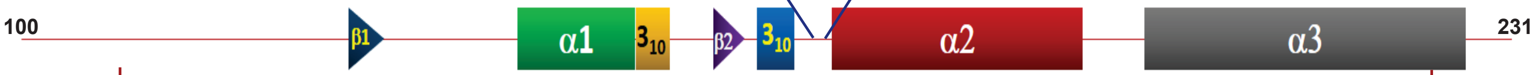

WXKPSKPKTNMKHVAGAAAAGAVVGGLGGYMLGSAMSRPLIHFGSDYEDRYYRENMYRYPSQVYYRPVDQYNSQNSFVHDCVNITVKQHTVTTTTTKGENFTETDIKMMERVVEQMCITQYQREYQAYYQRGAS-

N I M N W N N N N I X V V K S EGRS

PrP sequence differs by 14 amino acids from the bank vole PrP (see in red)

No deletions

One insertion

No polymorphic variants found

Tm^{Exp}: ND

ΔΔG: -16.96

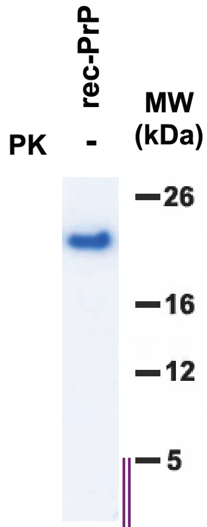

213 AA

NO MISFOLDING

0

Conformers

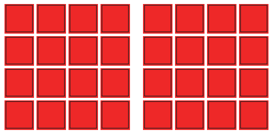

In vitro studies

NOT APPLICABLE

In vivo studies

NOT APPLICABLE

pLDDT

[86.98]

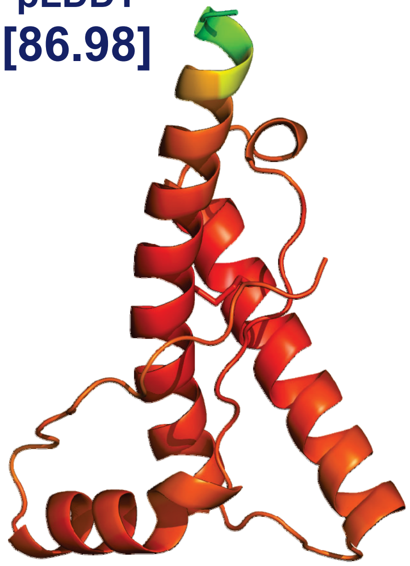

Protein code: 0960

Beluga whale

GenBank: XM_022556485

Delphinapterus leucas

Cetacea 0/14

Monodontidae 0/1

P29%

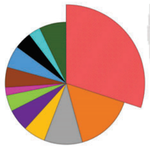

0

0

Distinct primary sequence across species

β2-α2 LOOP
NNQNSF
24

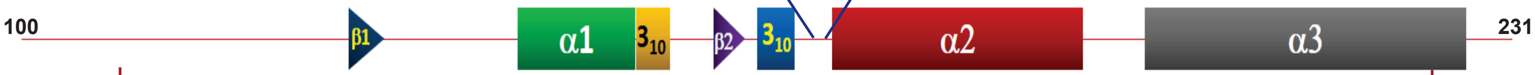

WXKPSKPKTNMKHVAGAAAAGAVVGGLGGYMLGSAMSRPLIHFGSDYEDRYYRENMYRYPNQVYYRPVDQYNNQNSFVHDCVNITVKQHTVTTTTTKGENFTETDIKMMERVVEQMCITQYQREYQAYYQRGAS-

N I M N W N N I X V V K S EGRS

PrP sequence differs by 12 amino acids from the bank vole PrP (see in red)

No deletions

One insertion

No polymorphic variants found

Tm^{Exp}: ND

ΔΔG: -17.39

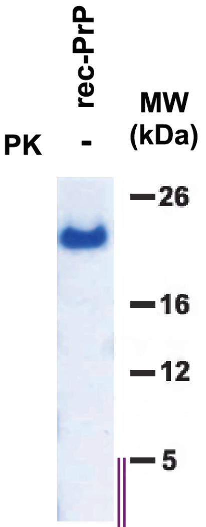

213 AA

NO MISFOLDING

0 Conformers

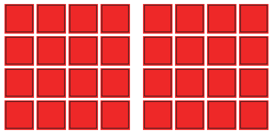

In vitro studies

NOT APPLICABLE

In vivo studies

NOT APPLICABLE

pLDDT [85.62]

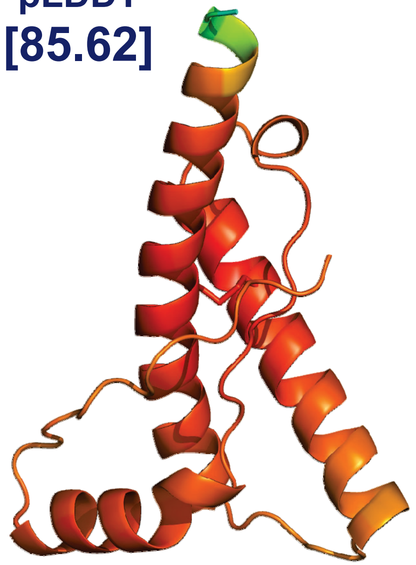

Protein code: 0924

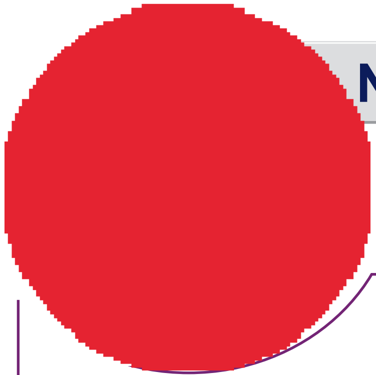

Northern bottlenose whale

GenBank: BK064204

Hyperoodon ampullatus

Cetacea 0/14

Ziphiidae 0/4

P29%

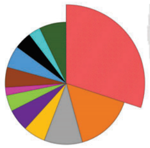

0

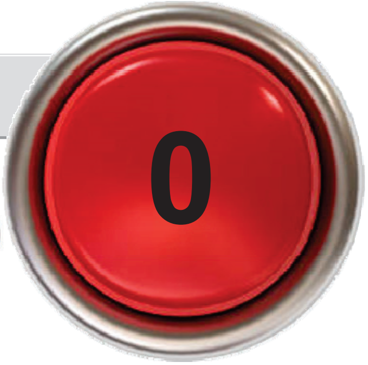

β2-α2 LOOP
NNQNSF
24

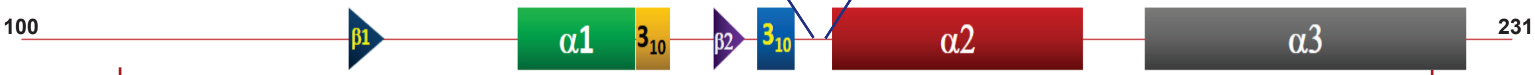

WXKPSKPKTNMKHMAGAAAAGAVVGGLGGYMLGSAMSRPLIHFGSDYEDRYYRENMYRYPNQVYYKPVDQYNNQNSFVHDCVNITVKQHTVTTTTTKGENFTETDIKMMERVVEEMCTTQYQREYQAYYQRGAS-

N I V M N W N R N I X V Q V K S EGRS

PrP sequence differs by 15 amino acids from the bank vole PrP (see in red)

No deletions

One insertion

No polymorphic variants found

Distinct primary sequence across species

Tm^{Exp}: ND

ΔΔG: -20.25

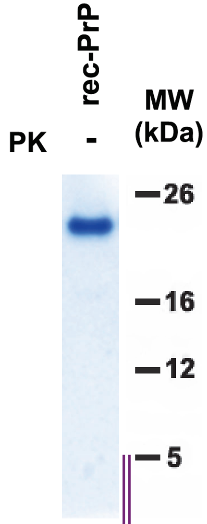

213 AA

NO MISFOLDING

0

Conformers

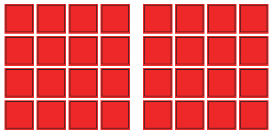

In vitro studies

NOT APPLICABLE

In vivo studies

NOT APPLICABLE

pLDDT
[87.26]

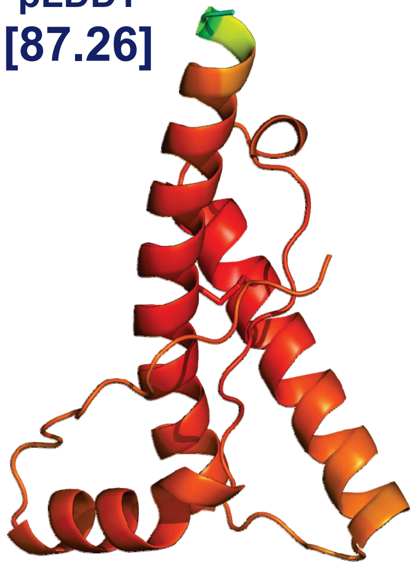

Protein code: 4556

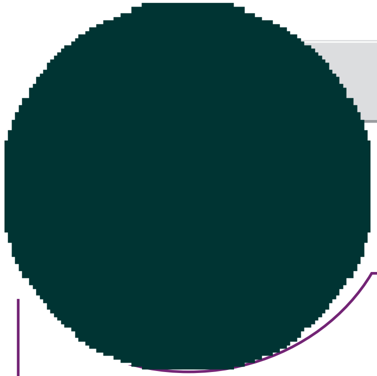

Amazon river dolphin

GenBank: BK064059

Inia geoffrensis

Distinct primary sequence across species

Cetacea	0/14
Iniidae	0/3

P29%

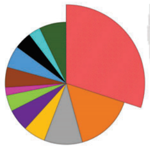

0

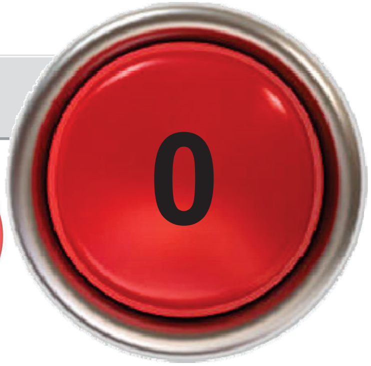

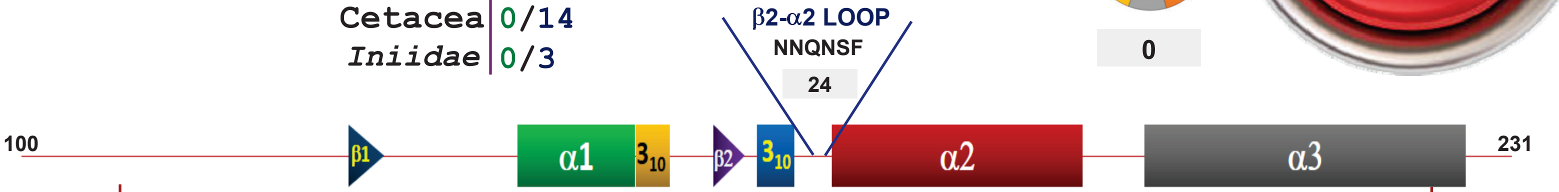

WXKPSKPKTNMKHVAGAAAAGAVVGGLGGYMLGSAMSRPLIHFGSDYEDRYYRENMYRYPNQVYYRPVDQYNNQNSFVHDCVNITVKQHTVTTTTTKGENFTETDIKMMERVVEQMCITQYQREYQAYYQRGAS-

N I M N W N N I X V V K S EGRS

PrP sequence differs by 12 amino acids from the bank vole PrP (see in red)

No deletions

One insertion

No polymorphic variants found

Tm^{Exp}: ND

ΔΔG: -17.25

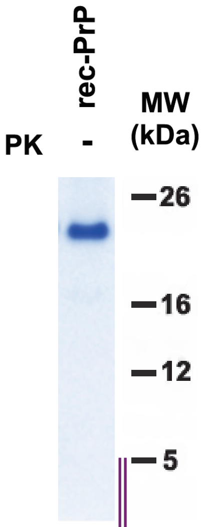

213 AA

NO MISFOLDING

0

Conformers

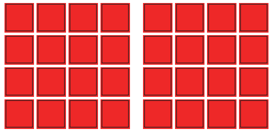

In vitro studies

NOT APPLICABLE

In vivo studies

NOT APPLICABLE

pLDDT

[86.97]

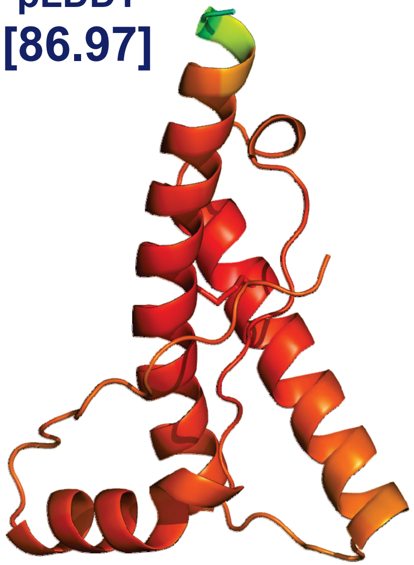

Protein code: 3228

Pacific white-sided dolphin

GenBank: XM_027118243

Lagenorhynchus obliquidens

Cetacea 0/14
Delphinidae 0/2

P29%

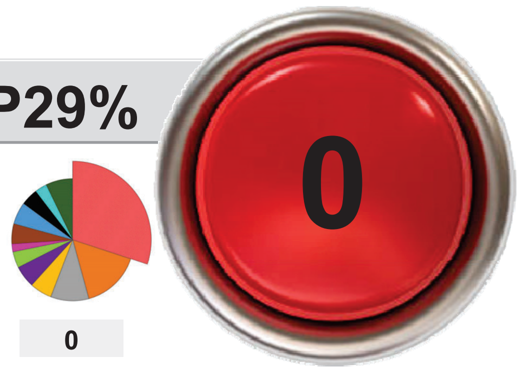

β2-α2 LOOP
NNQNSF
24

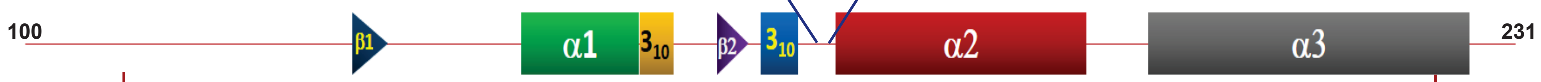

WXKPSKPKTNMKHVAGAAAAGAVVGGLGGYMLGSAMSRPLIHFGSDYEDRYYRENMYRYPNQVYYRPVDQYNNQNSFVHDCVNITVKQHTVTTTTTKGENFTETDIKMMERVVEQMCITQYQREYEAYYQRGYG-
N I M N W N N I X V V K SQ EGRSS

PrP sequence differs by 13 amino acids from the bank vole PrP (see in red)

No deletions
One insertion
No polymorphic variants found

Distinct primary sequence across species

Tm[Exp]: ND
ΔΔG: -30.33

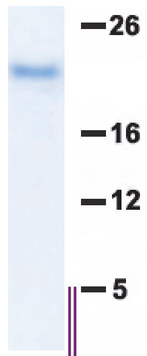

213 AA

NO MISFOLDING

0
Conformers

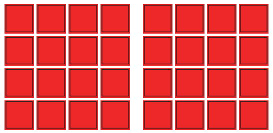

In vitro studies

NOT APPLICABLE

In vivo studies

NOT APPLICABLE

pLDDT
[87.41]

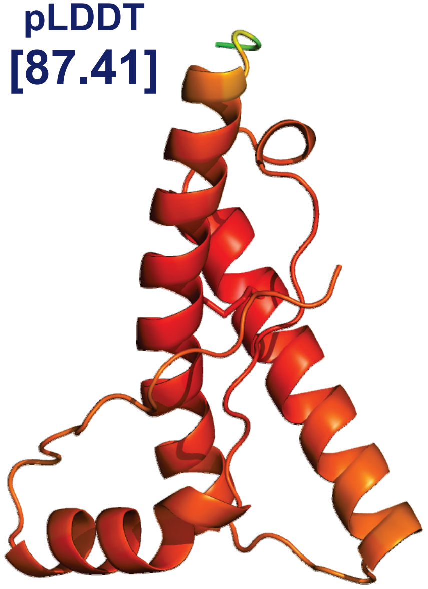

Protein code: 1416

Baiji

GenBank: XM_007463977

Lipotes vexillifer

Cetacea 0/14
Iniidae 0/3

Distinct primary sequence across species

P29%

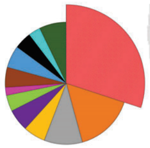

0

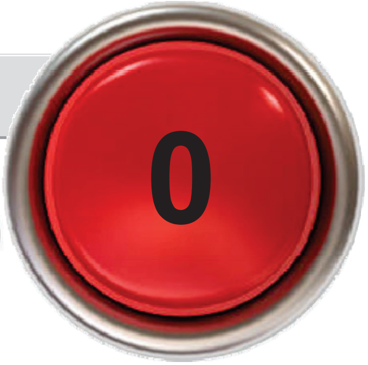

β2-α2 LOOP
NNQNSF
24

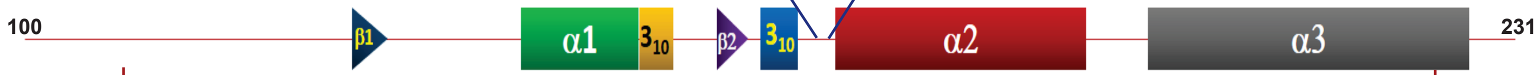

WXKPSKPKTNMKHVAGAAAAGAVVGGLGGYMLGSATSRPLIHFGSDYEDRYYRENMYRYPNQVYYRPVDQYNNQNSFVHDCVNITVKQHTVTTTTTKGENFTETDIKMMERVVEQMCITQYQREYQAYYQRGAS-

N I M M N W N N I X V V K S EGRS

PrP sequence differs by 13 amino acids from the bank vole PrP (see in red)

No deletions
One insertion
No polymorphic variants found

Tm^{Exp}: ND

ΔΔG: -10.64

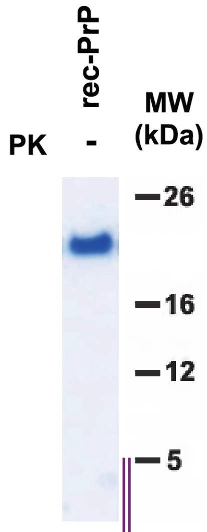

213 AA

NO MISFOLDING

0
Conformers

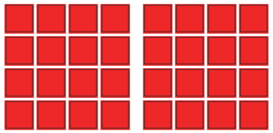

In vitro studies

NOT APPLICABLE

In vivo studies

NOT APPLICABLE

pLDDT
[85.68]

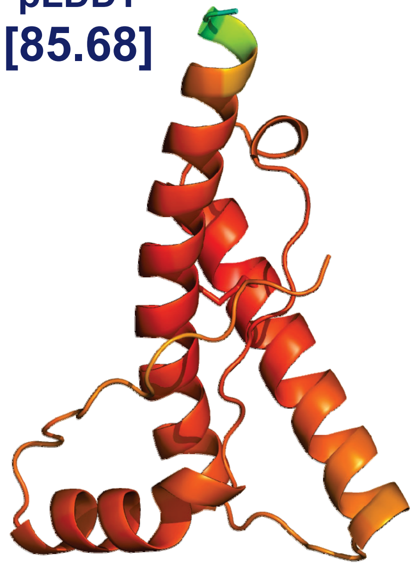

Protein code: 0922

Sowerby's beaked whale

GenBank: BK064083

Mesoplodon bidens

Cetacea	0/14
Ziphiidae	0/4

P29%

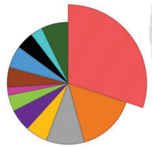

0

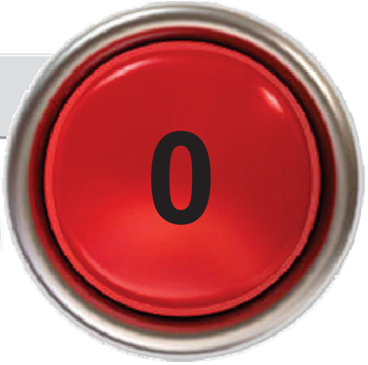

β2-α2 LOOP

NNQNSF

24

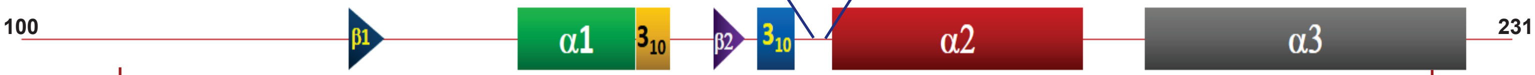

WXKPSKPKTNMKHVAGAAAAGAVVGGLGGYMLGSAMSRPLIHFGSDYEDRYYRENMYRYPNQVYYRPVDQYNNQNSFVHDCVNITVKQHTVTTTTTKGENFTETDIKMMERVVEEMCTTQYQREYQAYYQRGAS-

N I M N W N N I X V Q V K S EGRS

PrP sequence differs by 13 amino acids from the bank vole PrP (see in red)

No deletions

One insertion

No polymorphic variants found

Distinct primary sequence across species

Tm^{Exp}: ND

ΔΔG: -15.45

PK

rec-PrP

-

MW (kDa)

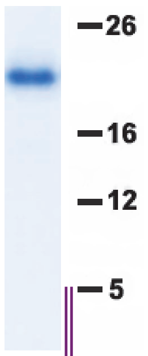

213 AA

NO MISFOLDING

0 Conformers

In vitro studies

NOT APPLICABLE

In vivo studies

NOT APPLICABLE

pLDDT [87.82]

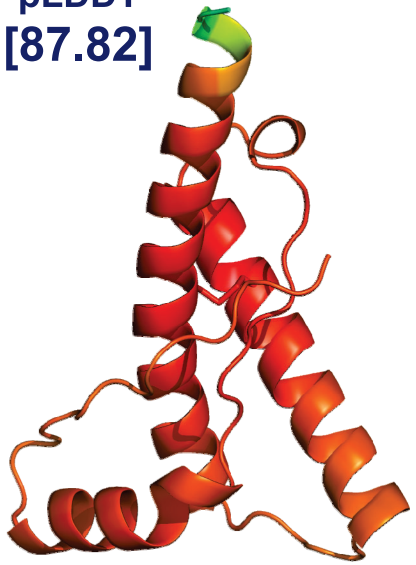

Protein code: 3262

Stejneger's beaked whale

GenBank: BK064780

Mesoplodon stejnegeri

Cetacea 0/14
Ziphiidae 0/4

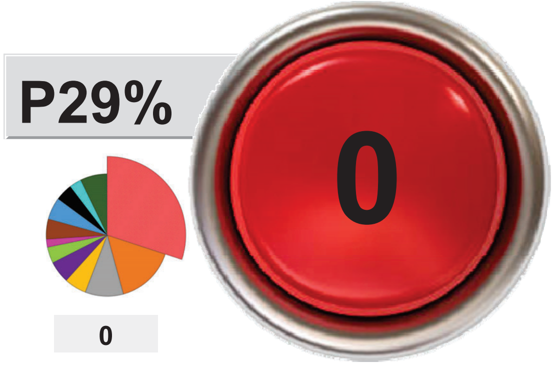

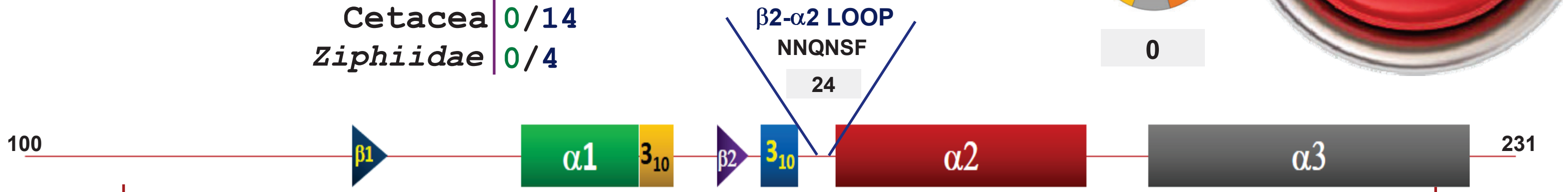

WXKPGKPKTNMKHVAGAAAAGAVVGGLGGYMLGSAMSRPLIHFGSDYEDRYYRENMYRYPNQVYYRPVDQYNNQNSFVHDCVNITVKQHTVTTTTTKGENFTETDIKMMERVVEEMCTTQYQREYQAYYQRGAS-
N S I M N W N N I X V Q V K S EGRS

PrP sequence differs by 13 amino acids from the bank vole PrP (see in red)

No deletions
One insertion
No polymorphic variants found

Distinct primary sequence across species

Tm^{Exp}: ND

ΔΔG: -16.57

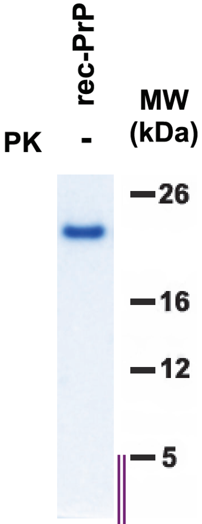

213 AA

NO MISFOLDING

0 Conformers

In vitro studies

NOT APPLICABLE

In vivo studies

NOT APPLICABLE

pLDDT [85.93]

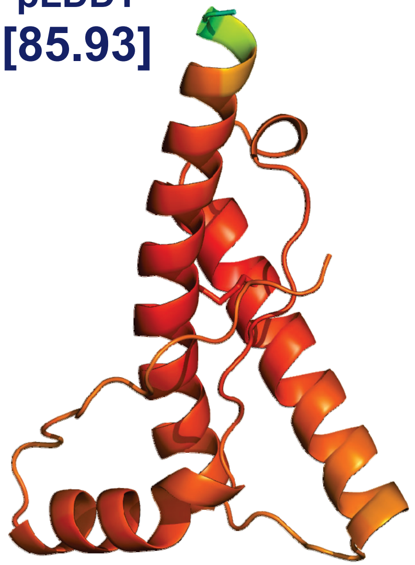

Protein code: 3937

Narrow-ridged finless porpoise

GenBank: XM_024764249

Neophocaena phocaenoides

Cetacea 0/14
Phocoenidae 0/1

Distinct primary sequence across species

P29%

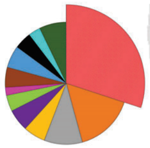

0

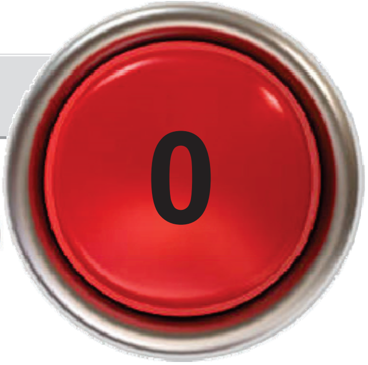

β2-α2 LOOP
NNQNSF
24

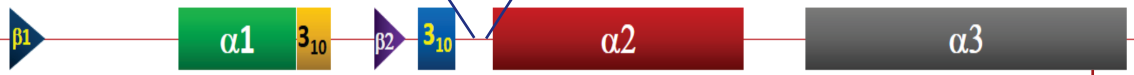

WXKPSKPKTNMKHVAGAAAAGAVVGGLGGYMLGSAMSRPLIHFGSDYEDRYYRENMYRYPNQVYYRPVDQYNNQNSFVHDCVNITVKQHTVTTTTTKGENFTETDIKMMERVVEQMCITQYQREYQAYYQRGYG-

N I M N W N N I X V V K S EGRSS

PrP sequence differs by 12 amino acids from the bank vole PrP (see in red)

No deletions
One insertion
No polymorphic variants found

Tm^{Exp}: ND

ΔΔG: -24.72

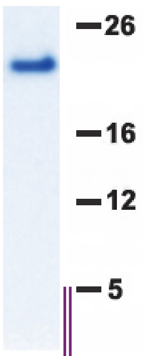

213 AA

NO MISFOLDING

0
Conformers

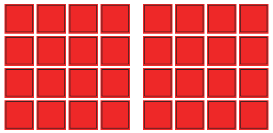

In vitro studies

NOT APPLICABLE

In vivo studies

NOT APPLICABLE

pLDDT
[86.94]

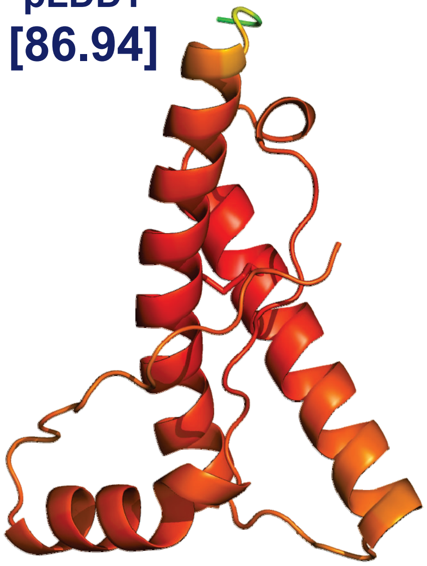

Protein code: 1126

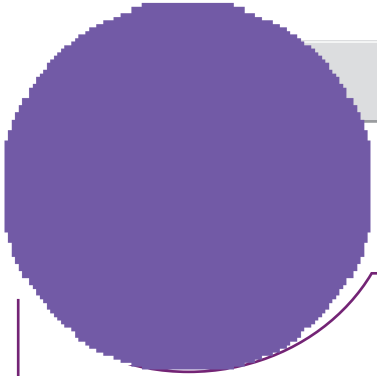

Sperm whale

GenBank: XM_007123285

Physeter catodon

Cetacea	0/14
Physeteridae	0/1

Distinct primary sequence across species

P29%

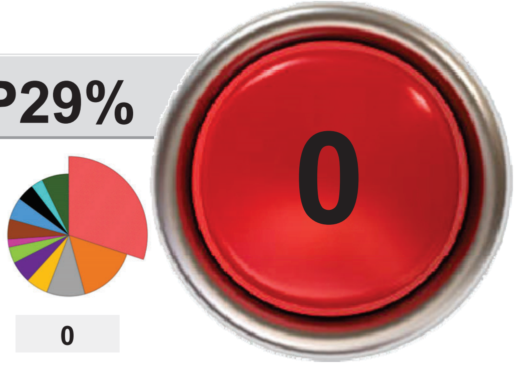

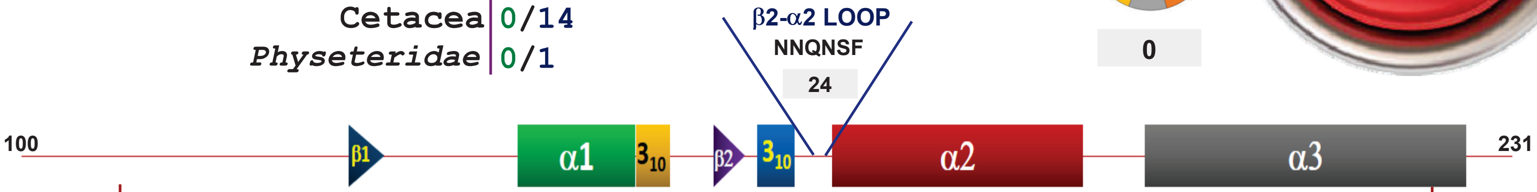

WXKPSKPKTNMKHVAGAAAAGAVVGGLGGYMLGSAMSRPLIHFGSDYEDRYYRENMYRYPNQVYYRPVDQYNNQNSFVHDCVNITVKQHTVTTTTTKGENFTETDIKMMERVVEQMCITQYQREYQAYYQRGAS-

N I M N W N N I X V V K S EGRS

PrP sequence differs by 12 amino acids from the bank vole PrP (see in red)

No deletions

One insertion

No polymorphic variants found

Tm^{Exp}: ND

ΔΔG: -16.05

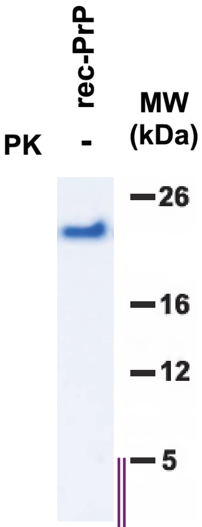

215 AA

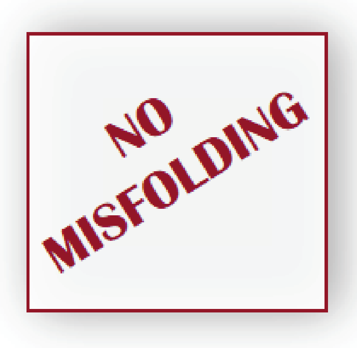

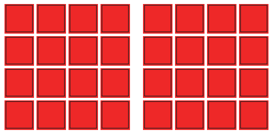

0

Conformers

In vitro studies

NOT APPLICABLE

In vivo studies

NOT APPLICABLE

pLDDT

[86.76]

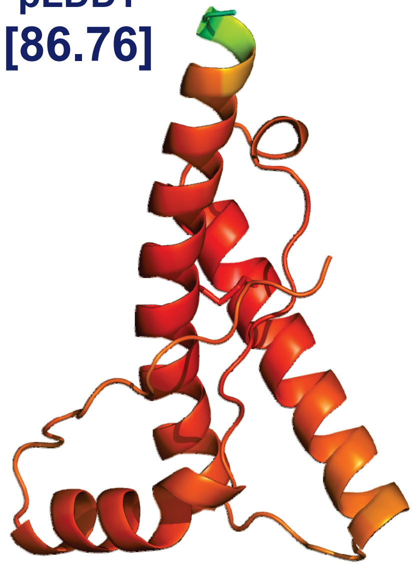

Protein code: 0956

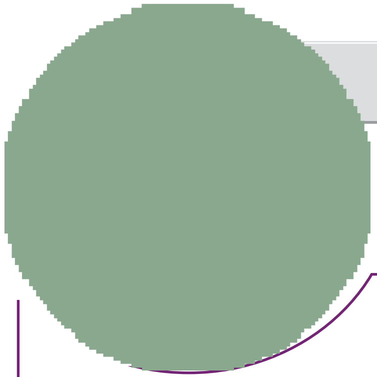

South Asian river dolphin

GenBank: BK064007

Platanista gangetica

Cetacea 0/14
Platanistidae 0/1

P29%

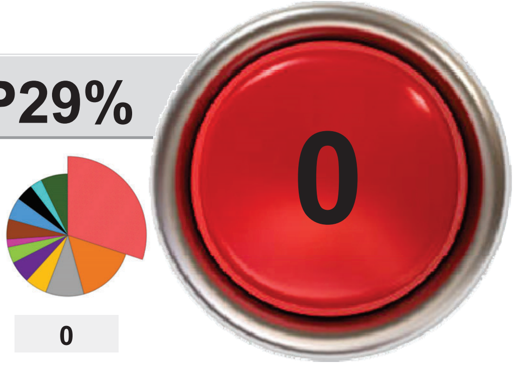

0

β2-α2 LOOP
NSQNSF
5

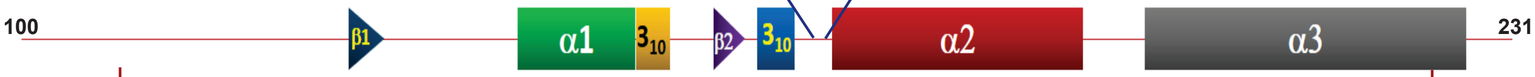

WXKPSKPKTNMKHVAGAAAAGAVVGGLGGYMLGSAMSRPLIHFGSDYEDRYYRENMYRYPNQVYYRPVDQYNSQNSFVHDCVNITVKQHTVTTTTTKGENFTETDIKMMERVVEQMCITQYQREYQAYYQRGAS-
N I M N W N N N I X V V K S EGRS

PrP sequence differs by 13 amino acids from the bank vole PrP (see in red)

No deletions
One insertion
No polymorphic variants found

Distinct primary sequence across species

Tm^{Exp}: ND

ΔΔG: -14.99

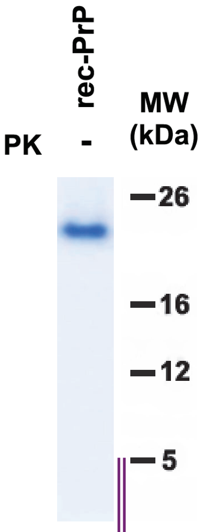

213 AA

NO MISFOLDING

0
Conformers

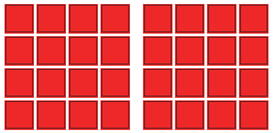

In vitro studies

NOT APPLICABLE

In vivo studies

NOT APPLICABLE

pLDDT
[86.35]

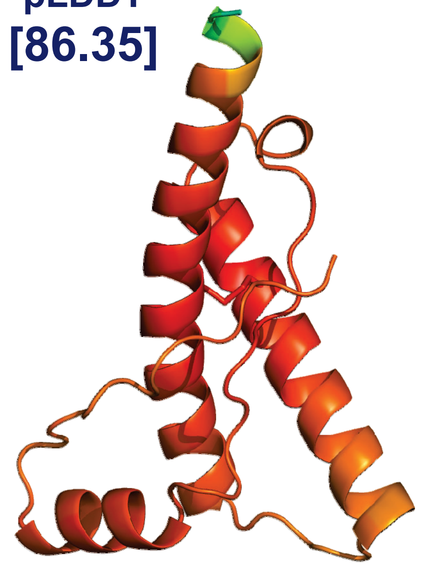

Protein code: 3168

La Plata dolphin

GenBank: BK064129

Pontoporia blainvillei

Cetacea 0/14
Iniidae 0/3

Distinct primary sequence across species

P29%

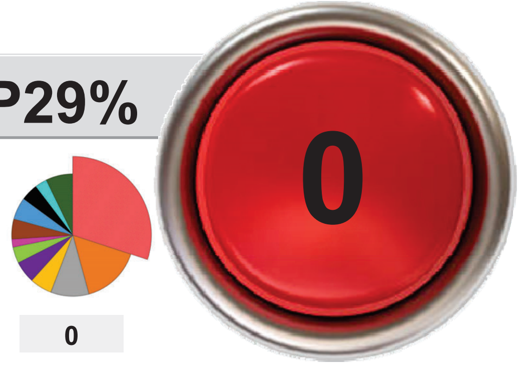

0

β2-α2 LOOP
NNQNSF
24

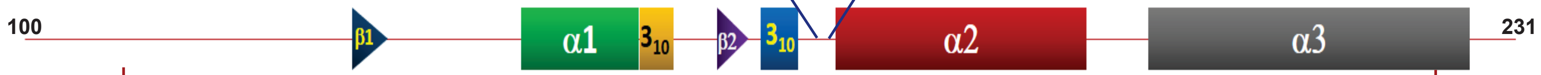


```
WXKPSKPKTNMKHVAGAAAAGAVVGGLGGYMLGSAMSRPLIHFGSDYEDRYYRENMYRYPNQVYYKPVDQYNNQNSFVHDCVNITVKQHTVTTTTTKGENFTETDIKMMERVVEQMCITQYQREYQAYYQRGAS-
 N       I                        M   N W      N     R      N    I    X    V      V   K S   EGRS
```

PrP sequence differs by 13 amino acids from the bank vole PrP (see in red)

No deletions
One insertion
No polymorphic variants found

Tm^{Exp}: ND

ΔΔG: -13.69

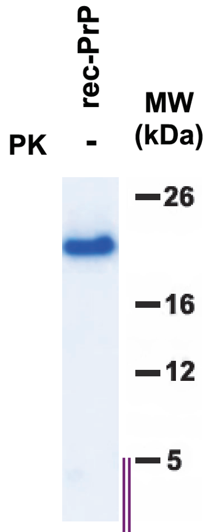

213 AA

NO MISFOLDING

0 Conformers

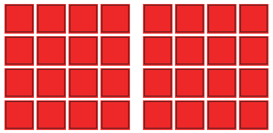

In vitro studies

NOT APPLICABLE

In vivo studies

NOT APPLICABLE

pLDDT [85.83]

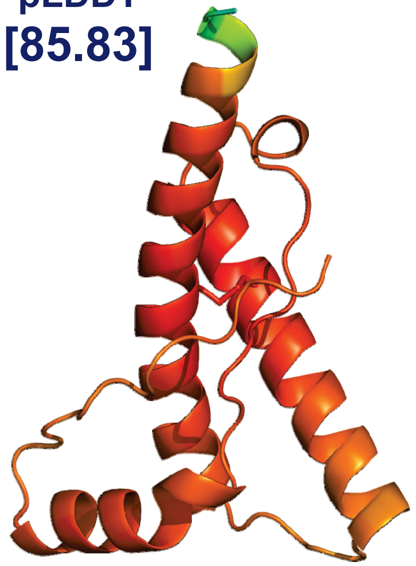

Protein code: 3311

Common bottlenose dolphin

GenBank: XM_019941481

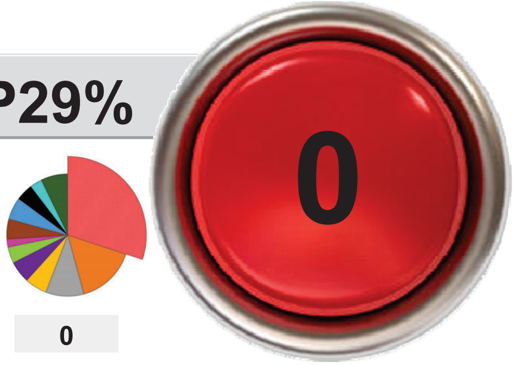

P29%

0

Tursiops truncatus

Cetacea	0/14
Delphinidae	0/2

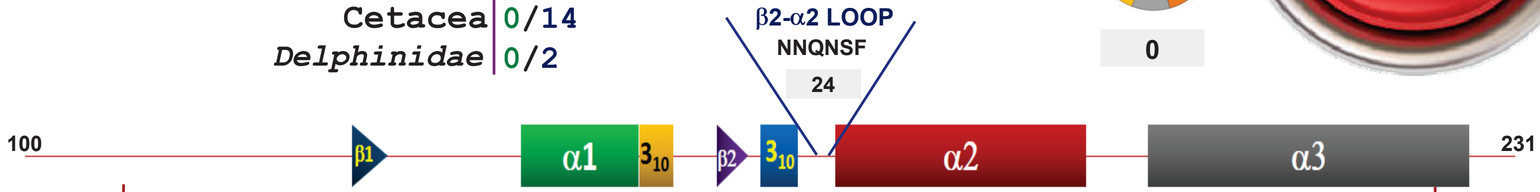

WXKPSKPKTNMKHVAGAAAAGAVVGGLGGYMLGSAMSRPLIHFGSDYEDRYYRENMYRYPNQVYYRPVDQYNNQNSFVHDCVNITVKQHTVTTTTTKGENFTETDIKMMERVVEQMCITQYQREYEAYYQRGAS-

N I M N W N N I X V V K SQ EGRS

PrP sequence differs by 13 amino acids from the bank vole PrP (see in red)

No deletions

One insertion

It shows polymorphic variants

Distinct primary sequence across species

Tm^{Exp}: 68.6 ºC

ΔΔG: -19.79

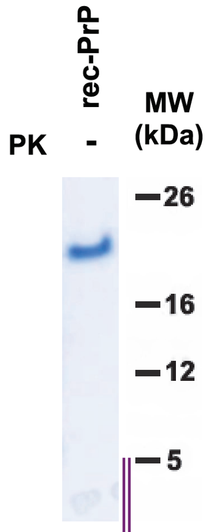

213 AA

NO MISFOLDING

0

Conformers

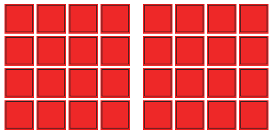

In vitro studies

NOT APPLICABLE

In vivo studies

NOT APPLICABLE

pLDDT

[86.43]

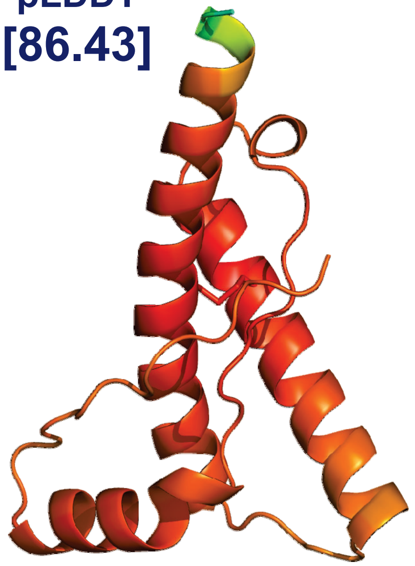

Protein code: 0409

Ziphius

GenBank: DQ884467

Ziphius cavirostris

Cetacea 0/14

Ziphiidae 0/4

P29%

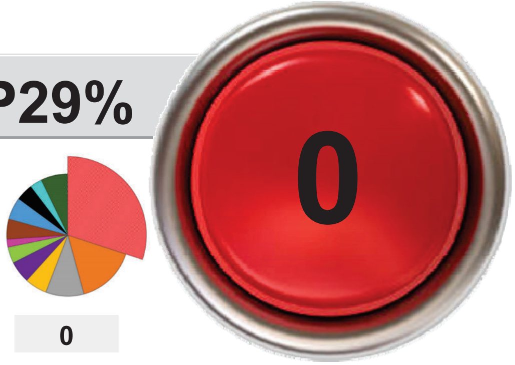

β2-α2 LOOP

NNQNSF

24

100 β1 α1 3_{10} β2 3_{10} α2 α3 231

WXKPSKPKTNMKHMAGAAAAGAVVGGLGGYMLGSAMSRPLIHFGSDYEDRYYRENMYRYPNQVYYRPVDQYNNQNSFVHDCVNITVKQHTVTTTTTKGENFTETDIKMMERVVEEMCTTQYQREYQAYYQRGAS-

N I V M N W N N I X V Q V K S EGRS

PrP sequence differs by 14 amino acids from the bank vole PrP (see in red)

No deletions

One insertion

No polymorphic variants found

Distinct primary sequence across species

Tm^{Exp}: ND

ΔΔG: -21.45

PK - rec-PrP

MW (kDa)

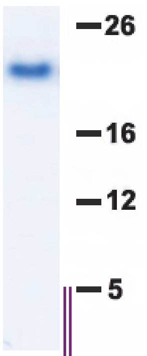

213 AA

NO MISFOLDING

0 Conformers

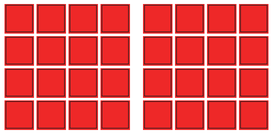

In vitro studies

NOT APPLICABLE

In vivo studies

NOT APPLICABLE

pLDDT [86.92]

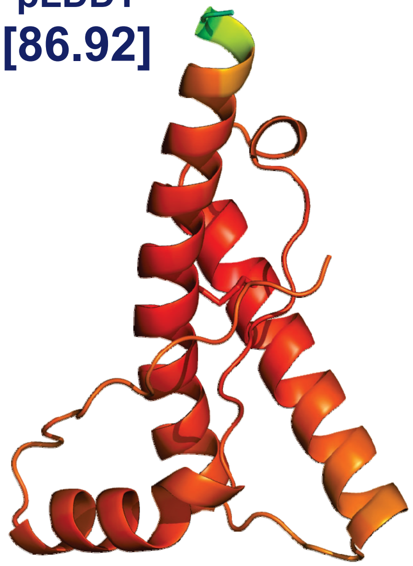

Protein code: 0961

Tailed tailless bat

GenBank: BK064067

P29%

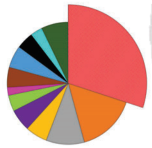

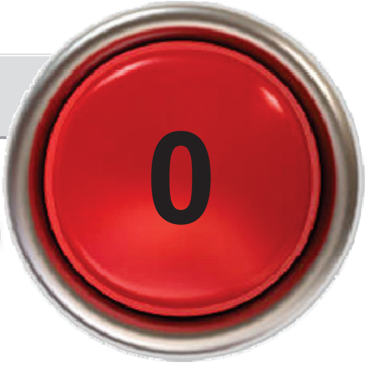

0

Anoura caudifer

Chiroptera 23/45
Phyllostomidae 2/10

β2-α2 LOOP
SNQNNF
273

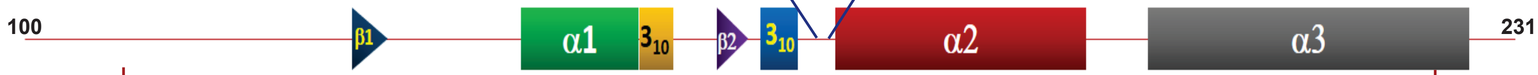

GGKPSKPKTNMKHVAGAAAAGAVVGGLGGYMLGSAMSRPLIHFGSDYEDRYYRENMNRFPNQVYYRPVDQYSNQNNFVRDCVNITVKEHTVTTTTKGENFTETDVKIMERVVEEMCTIQYQREYQAYQRGAS-
WN I M N W Y N H I Q M Q VT K S YE R S

PrP sequence differs by 15 amino acids from the bank vole PrP (see in red)

No deletions
No insertions
No polymorphic variants found

Distinct primary sequence across species

Tm^{Exp}: ND
ΔΔG: ND

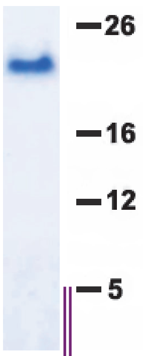

214 AA

NO MISFOLDING

0
Conformers

In vitro studies

NOT APPLICABLE

In vivo studies

NOT APPLICABLE

pLDDT
[87.61]

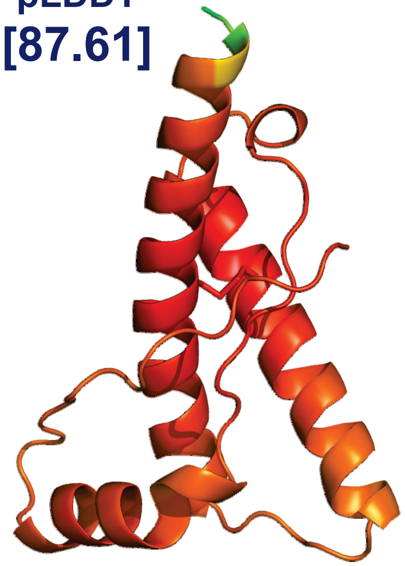

Protein code: 3243

Pallid bat

GenBank: BK064069

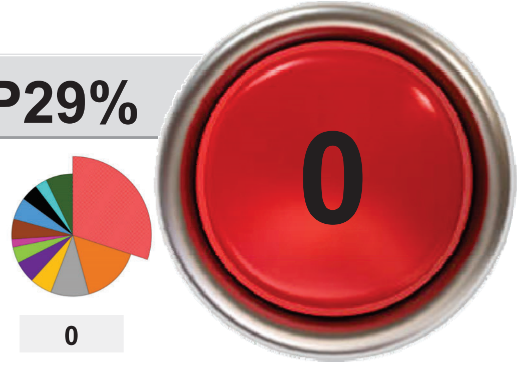

Antrozous pallidus

Chiroptera 23/45
Vespertilionidae 7/16

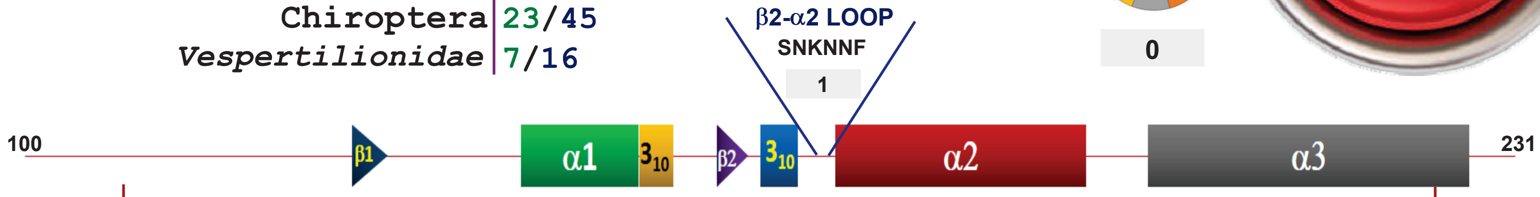

NKPNKPPKTNMKHVAGAAAAGAVVGGLGGYMLGSAMSRPAMHFGNEYEDRYYRENMNRFPEHVYYKPVDQYSNKNNFVRDCVNITVKQHTVTTTTKGENFTETDVKIMERVVEEMCTTQYQKEYQAAYQRGAS-
WNKPSK I MI DW Y NQ R N Q H I M Q V S Y EGRS

PrP sequence differs by 17 amino acids from the bank vole PrP (see in red)

No deletions
No insertions
No polymorphic variants found

Distinct primary sequence across species

Tm^{Exp}: ND

ΔΔG: -1.85

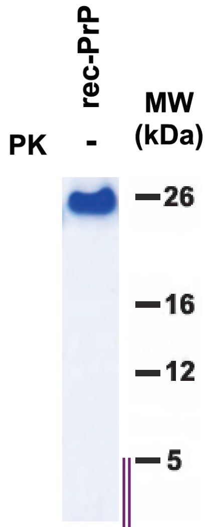

228 AA

NO MISFOLDING

0
Conformers

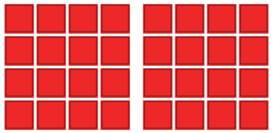

In vitro studies

NOT APPLICABLE

In vivo studies

NOT APPLICABLE

pLDDT
[87.3]

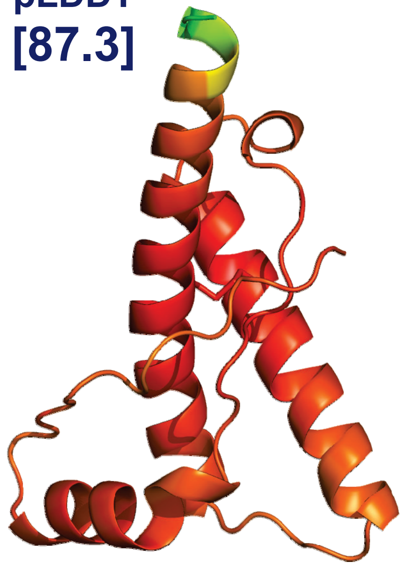

Protein code: 3246

Jamaican fruit bat

GenBank: BK064072

Artibeus jamaicensis

Chiroptera 23/45
Phyllostomidae 2/10

P29%

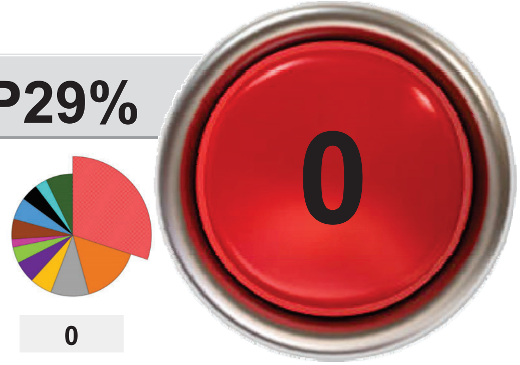

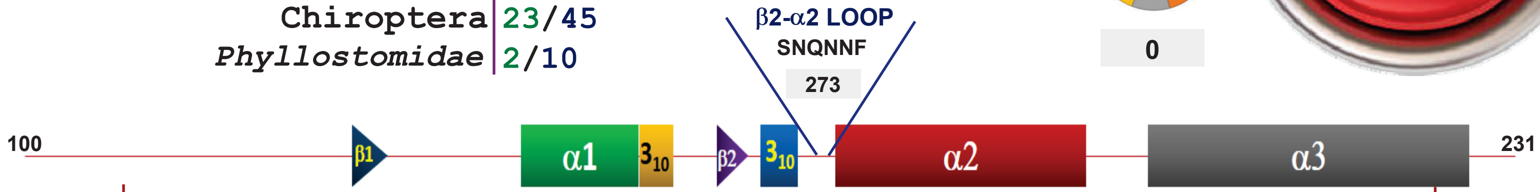

GGKPSKPKTNMKHVAGAAAAGAVVGGLGGYMLGSAMSRPLIHFGNDYEDRYYRENMNRFPNQVYYRPVDQYSNQNNFVRDCVNITVKEHTVTTTTKGENFTSTDIKIMERVVEEMCTVQYQREYQAYQRGAS-
WN I M W Y N H I Q E V M Q VT K S YE R S

PrP sequence differs by 16 amino acids from the bank vole PrP (see in red)

No deletions
No insertions
No polymorphic variants found

Distinct primary sequence across species

Tm^{Exp}: ND
ΔΔG: ND

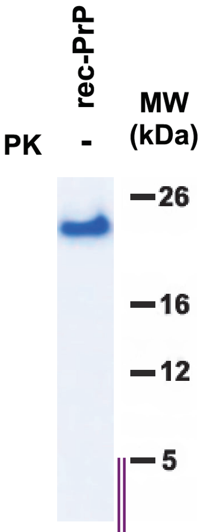

214 AA

NO MISFOLDING

0
Conformers

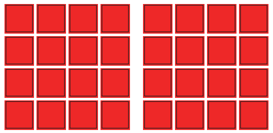

In vitro studies

NOT APPLICABLE

In vivo studies

NOT APPLICABLE

pLDDT
[86.75]

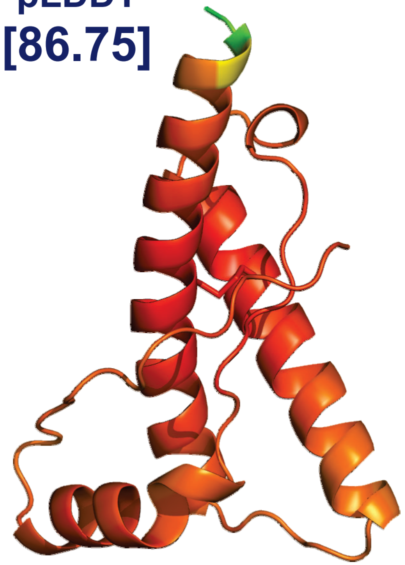

Protein code: 3249

Seba's short-tailed bat

GenBank: BK063935

Carollia perspicillata

Chiroptera 23/45

Phyllostomidae 2/10

P29%

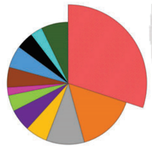

0

0

β2-α2 LOOP
SNQNNF
273

100 β1 α1 3_{10} β2 3_{10} α2 α3 231

GGKPSKPKTNMKHVAGAAAAGAVVGGLGGYMLGSAMSRPLIHFGNDYEDRYYRENMNRFPNQVYYRPVDQYSNQNNFVRDCVNITVKEHTVTTTTKGENFTATDVKIMERVVEEMCTMQYQREYQAYQRGAS-

WN I M W Y N H I Q E M Q VT K S YE R S

PrP sequence differs by 15 amino acids from the bank vole PrP (see in red)

No deletions

No insertions

No polymorphic variants found

Distinct primary sequence across species

Tm^{Exp}: ND

ΔΔG: ND

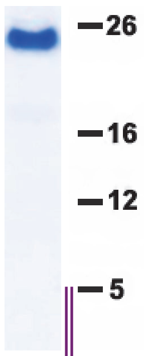

226 AA

NO MISFOLDING

0 Conformers

In vitro studies

NOT APPLICABLE

In vivo studies

NOT APPLICABLE

pLDDT [87.23]

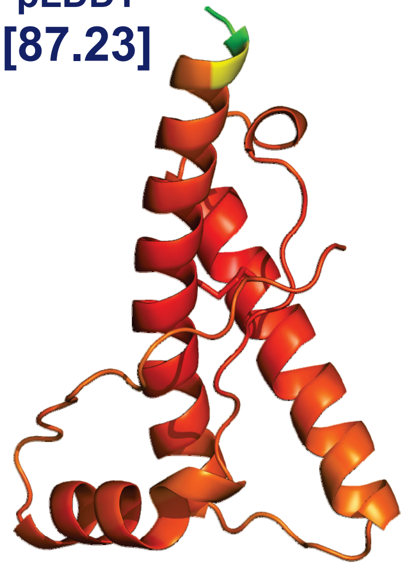

Protein code: 3058

Townsend's big-eared bat

GenBank: BK064211

Corynorhinus townsendii

Chiroptera 23/45
Vespertilionidae 7/16

P29%

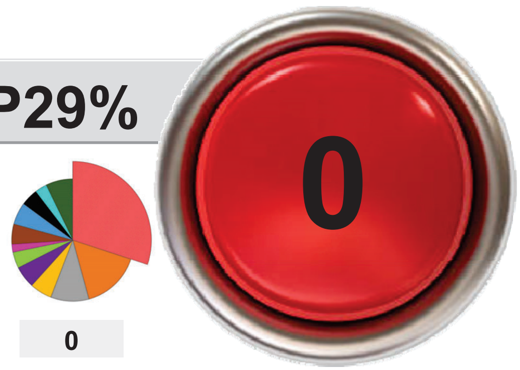

β2-α2 LOOP
NNQNNF
164

100 β1 α1 3_{10} β2 3_{10} α2 α3 231

WSKPNKPKTNMKHMAGAAAAGAVVGGLGGYMLGSAMSRPAMHFGNEYEDRYYRENMNRFPEQVYYKPVDQYNNQNNFVRDCVNITVKQHTVTTTTKGENFTETDIKIMERVVEEMCTTQYKKEYQASYQRGAS-
N S I V MI DW Y N R H I V M Q V Q S Y EGRS

PrP sequence differs by 17 amino acids from the bank vole PrP (see in red)

No deletions
No insertions
No polymorphic variants found

Tm^{Exp}: ND
ΔΔG: -17.27

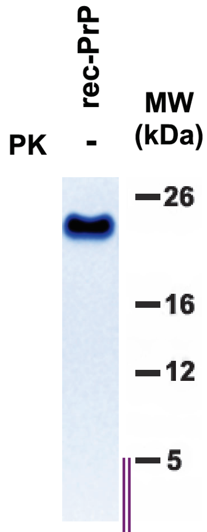

218 AA

NO MISFOLDING

0
Conformers

In vitro studies

NOT APPLICABLE

In vivo studies

NOT APPLICABLE

pLDDT
[91.52]

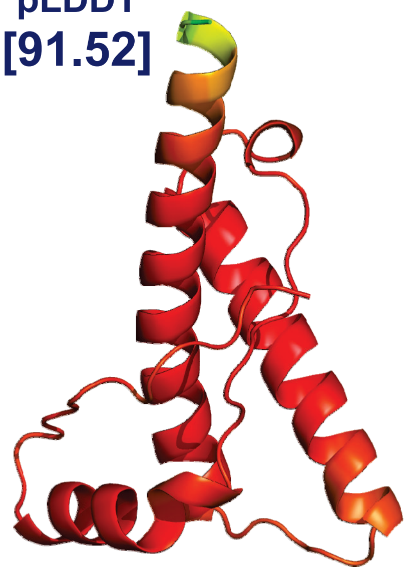

Protein code: 4684

Kitti's hog-nosed bat

GenBank: BK063940

Craseonycteris thonglongyai

Chiroptera 23/45
Craseonycteridae 1/1

P46%

10.7

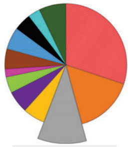

β2-α2 LOOP
SNQNNF
273

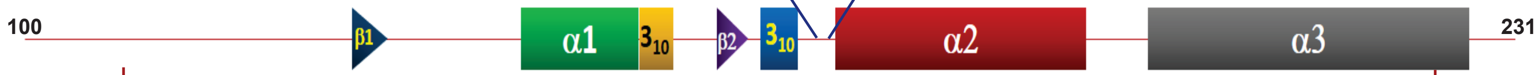

WNKPSKPKTNMKHVAGAAAAGAVVGGLGGYMLGSAMSRPMIHFGNDYEDRYYRENMYRYPNQVYYRPVDQYSNQNNFVHDCVNITVKQHTVTTTTKGENFTETDVKMMERVVEQMCITQYQRESHAAYQRGAS-
I W N N I V K Q Y EGRS

PrP sequence differs by 8 amino acids from the bank vole PrP (see in red)

No deletions
No insertions
No polymorphic variants found

Distinct primary sequence across species

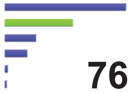

76

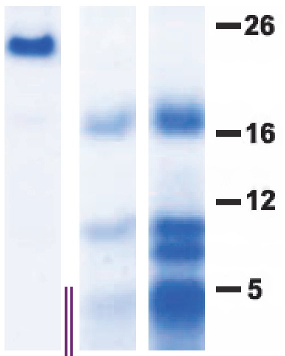

221 AA

2
Conformers

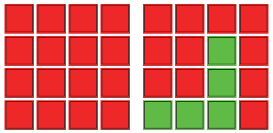

Tm^{Exp}: ND

ΔΔG: -6.77

In vitro studies

TgVole (1x) Not tested

Kitti's hog-nosed bat Not tested

In vivo studies

TgVole (1x) Not tested

Kitti's hog-nosed bat Not tested

pLDDT
[85.36]

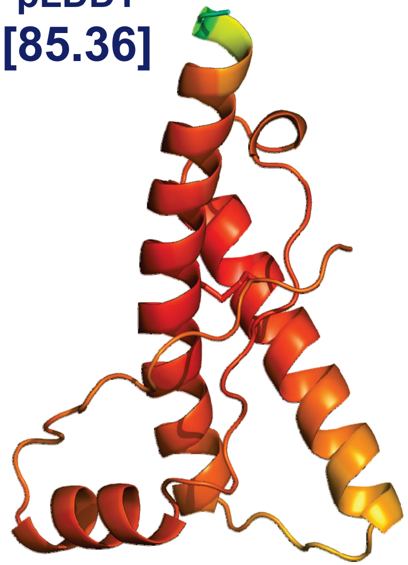

Protein code: 3066

Indian short-nosed fruit bat

GenBank: AY133046

Cynopterus sphinx

Chiroptera 23/45
Pteropodidae 8/8

1 species sharing the same primary sequence

Lesser short-nosed fruit bat
Cynopterus brachyotis

P89%

87.5

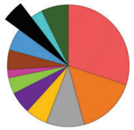

80-90

β2-α2 LOOP
SNQNSF
36

100 β1 α1 3_{10} β2 3_{10} α2 α3 231

```
WNKPSKPKTNLKHVAGAAAAGAVVGGLGGYMLGSAMSRPMIHFGNDYEDRYYRENMNRYPNQVYYRPVDQYSNQNSFVHDCVNITVKQHTVTTTTKGENFTETDVKIMERVVEQMCITQYQQESRAAYHRGAS-
           I                                   W                             N   N           I                       M         V     K   Q Y EGRS
```

PrP sequence differs by 9 amino acids from the bank vole PrP (see in red)

No deletions
No insertions
No polymorphic variants found

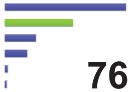

76

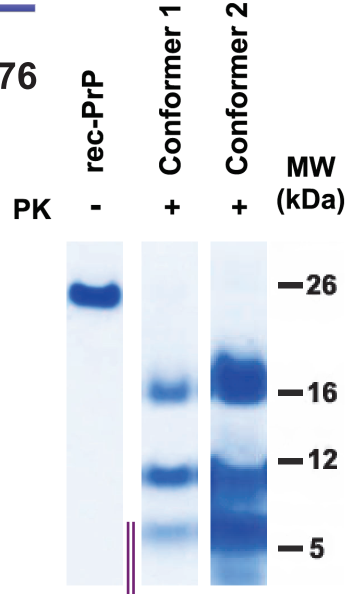

213 AA

2 Conformers

Tm^{Exp}: ND

ΔΔG: -7.08

***In vitro* studies**

TgVole (1x) — Not tested

Indian short-nosed fruit bat — Not tested

***In vivo* studies**

TgVole (1x) — Not tested

Indian short-nosed fruit bat — Not tested

pLDDT [84.43]

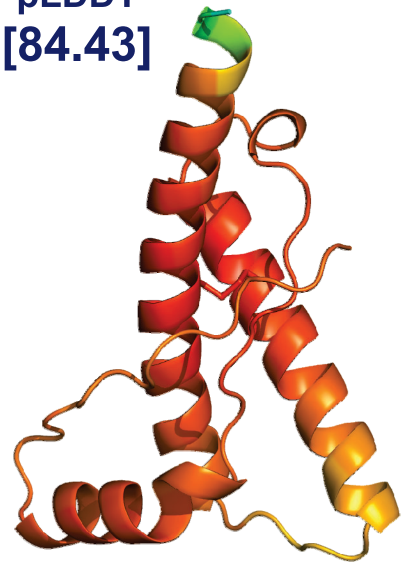

Protein code: 1156

Common vampire bat

GenBank: BK064947

Desmodus rotundus

Chiroptera 23/45

Phyllostomidae 2/10

P31%

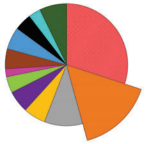

0-10

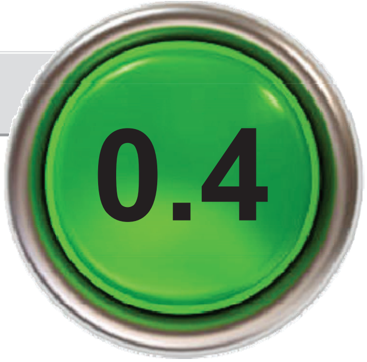

β2-α2 LOOP
SNQNSF
36

100 β1 α1 3_{10} β2 3_{10} α2 α3 231

```
GGKPSKPKTNMKHVAGAAAAGAVVGGLGGYMLGSAMSRPLIHFGNDYEDRYYRDNMNRFPNQVYYRPVDQYSNQNSFVRDCVNITVKEHTVTTTTKGENFTETDVKIMERVVEEMCTIQYQKEYQAYQRGAS-
WN        I                             M       W       E    Y            N    N  H       I Q                            M      Q  VT      S    YE R S
```

PrP sequence differs by 15 amino acids from the bank vole PrP (see in red)

No deletions

No insertions

No polymorphic variants found

Distinct primary sequence across species

135

216 AA

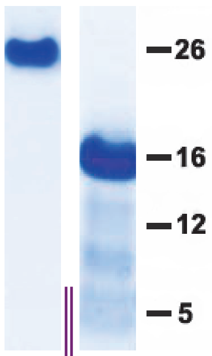

1

Conformers

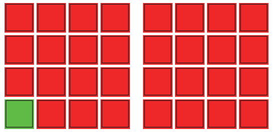

Tm^{Exp}: ND

ΔΔG: ND

In vitro studies

TgVole (1x) — Not tested

Common vampire bat — Not tested

In vivo studies

TgVole (1x) — Not tested

Common vampire bat — Not tested

pLDDT [85.91]

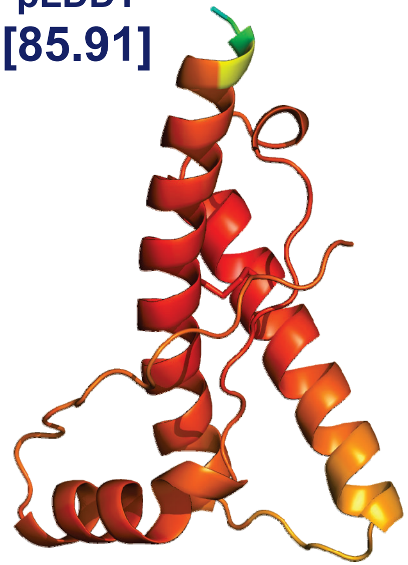

Protein code: 1472

Straw-coloured fruit bat

GenBank: BK064039

Eidolon helvum

Chiroptera 23/45

Pteropodidae 8/8

1 species sharing the same primary sequence

Madagascan fruit bat
Eidolon dupreanum

P86%

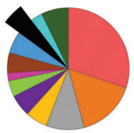

82.1

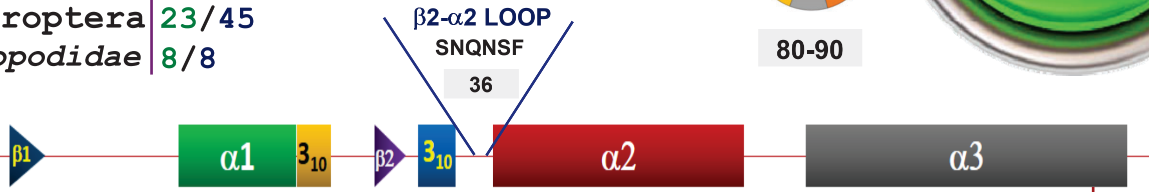


```
WNKPSKPKTNLKHVAGAAAAGAVVGGLGGYMLGSAMSRPMLHFGNDYEDRYYRENMYRYPNQVYYRPVDQYSNQNSFVHDCVNITVKQHTVTTTTKGENFTETDVKMMERVVEQMCITQYQQESRAAYHRGAS-
           I                                I     W          N               N   N         I                                           V     K   Q Y EGRS
```

PrP sequence differs by 10 amino acids from the bank vole PrP (see in red)

No deletions
No insertions
No polymorphic variants found

135

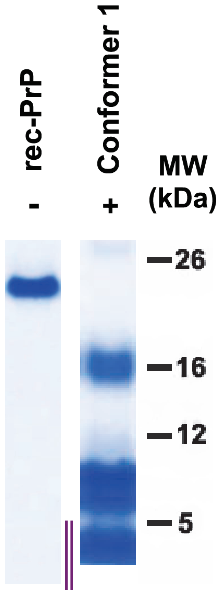

220 AA

1
Conformers

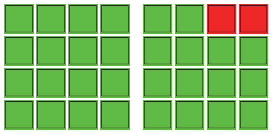

Tm^{Exp}: ND

ΔΔG: -16.93

In vitro studies

TgVole (1x) Not tested

Straw-coloured fruit bat Not tested

In vivo studies

TgVole (1x) Not tested

Straw-coloured fruit bat Not tested

pLDDT
[85.84]

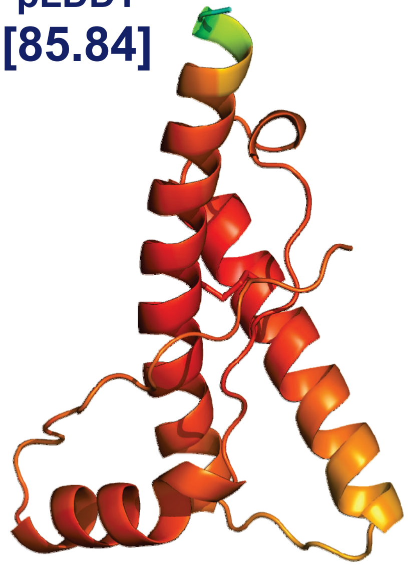

Protein code: 3205

Cave nectar bat

GenBank: BK064041

Eonycteris spelaea

Chiroptera 23/45
Pteropodidae 8/8

P56%

21.4

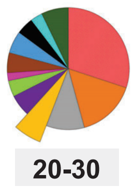

β2-α2 LOOP
NNQNSF
24

100 β1 α1 3_{10} β2 3_{10} α2 α3 231

```
WNKPSKPKTNLKHVAGAAAAGAVVGGLGGYMLGSAMSRPMLHFGNDYEDRYYRENMYRYPNQVYYRPVDQYNNQNSFVRDCVNITVKEHTVTTTTKGENFTETDVKMMERVVEQMCITQYQQESRAAYHRGAS-
           I                                 I     W           N                          N   H      I Q                               V     K   Q Y EGRS
```

PrP sequence differs by 11 amino acids from the bank vole PrP (see in red)

No deletions
No insertions
No polymorphic variants found

Distinct primary sequence across species

135

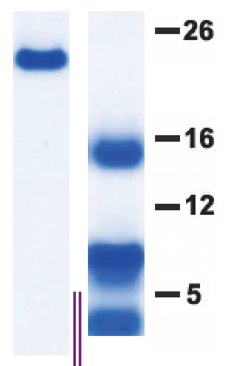

219 AA

1
Conformers

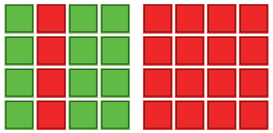

Tm^{Exp}: ND

ΔΔG: -13.08

In vitro studies

TgVole (1x) Not tested

Cave nectar bat Not tested

In vivo studies

TgVole (1x) Not tested

Cave nectar bat Not tested

pLDDT
[85.61]

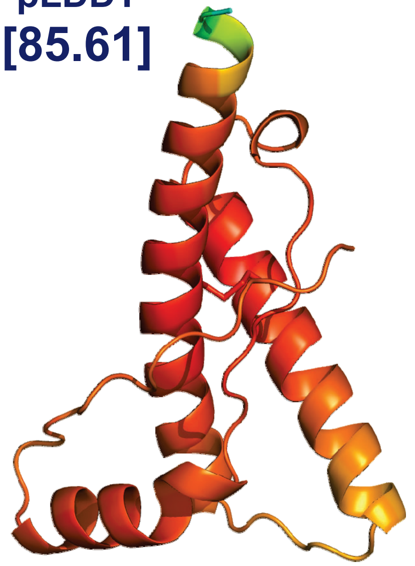

Protein code: 3207

Big brown bat

GenBank: BK064945

Eptesicus fuscus

Chiroptera 23/45

Vespertilionidae 7/16

P31% 0.4

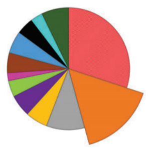

β2-α2 LOOP
NNQNNF
164

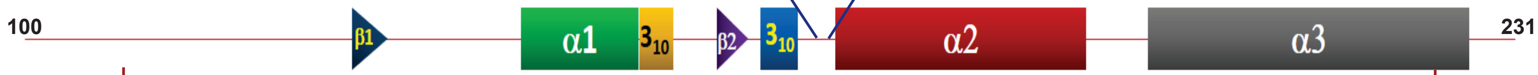

WNKPNKPKTNMKHMAGAAAAGAVVGGLGGYMLGSAMSRPAMHFGNEYEDRYYRENMNRFPERVYYKPVDQYNNQNNFVRDCVNITVKQHTVTTTTKGDNFTETDVKIMERVVEEMCTTQYQKEYQAAYQRGAS-

S I V MI DW Y NQ R H I E M Q V S Y EGRS

PrP sequence differs by 17 amino acids from the bank vole PrP (see in red)

No deletions
No insertions
No polymorphic variants found

Distinct primary sequence across species

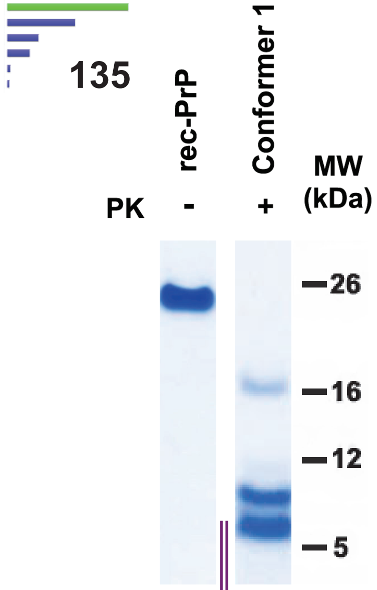

217 AA

1 Conformers

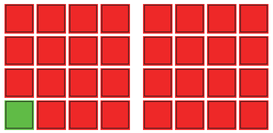

Tm[Exp]: ND

ΔΔG: -0.01

In vitro studies

TgVole (1x) Not tested

Big brown bat Not tested

In vivo studies

TgVole (1x) Not tested

Big brown bat Not tested

pLDDT [86.88]

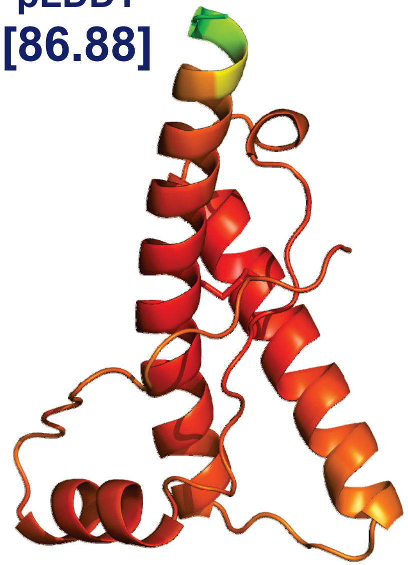

Protein code: 1139

Great roundleaf bat

GenBank: XM_019644637

Hipposideros armiger

Chiroptera 23/45
Hipposideridae 2/2

P86%

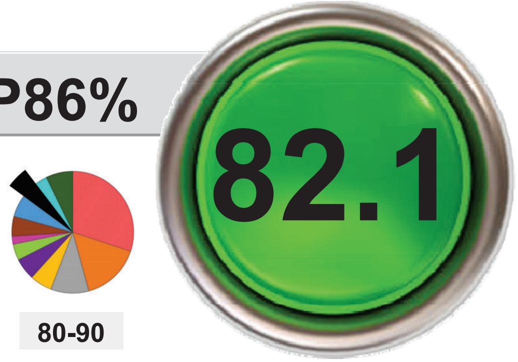

β2-α2 LOOP
NNQNNF
164

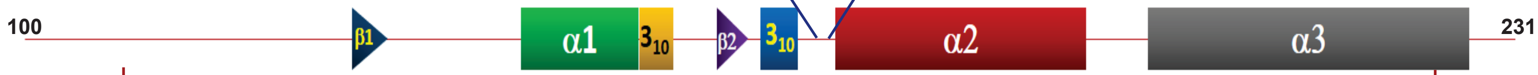


```
WNKPSKPKTNMKHVAGAAAAGAVVGGLGGYMLGSAMSRPLMHFGNDYEDRYYRENMYRYPNQVYYYRPVDQYNNQNNFVHDCVNITIKQHTVTTTTKGENFTETDVKMMERVVEQMCITQYQKESHAAYQRDAS-
           I                                    MI     W          N                                                                      V         Q Y EGRS
```

PrP sequence differs by 7 amino acids from the bank vole PrP (see in red)

No deletions
No insertions
No polymorphic variants found

1 species sharing the same primary sequence

Pendlebury's roundleaf bat
Hipposideros turpis pendleburyi

76

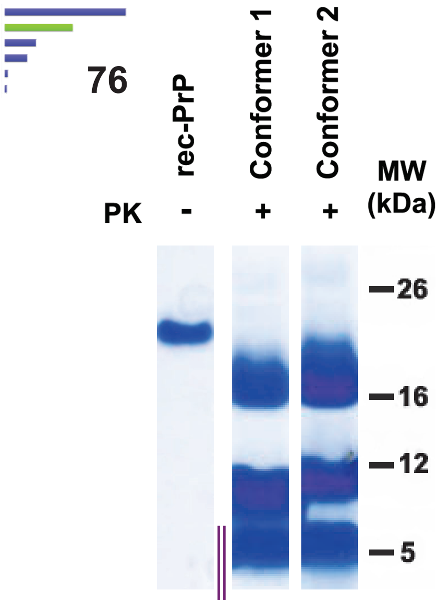

203 AA

2
Conformers

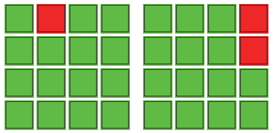

Tm^{Exp}: ND
ΔΔG: -2.36

In vitro studies

TgVole (1x) Not tested
Great roundleaf bat Not tested

In vivo studies

TgVole (1x) Not tested
Great roundleaf bat Not tested

pLDDT
[89.32]

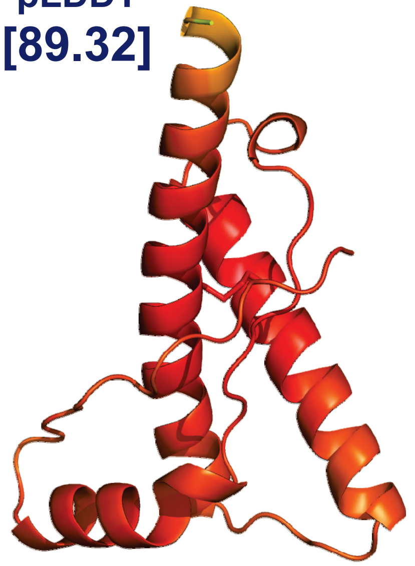

Cantor's roundleaf bat

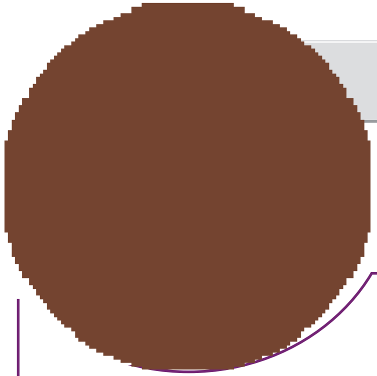

GenBank: BK064054

Hipposideros galeritus

Chiroptera 23/45
Hipposideridae 2/2

P65%

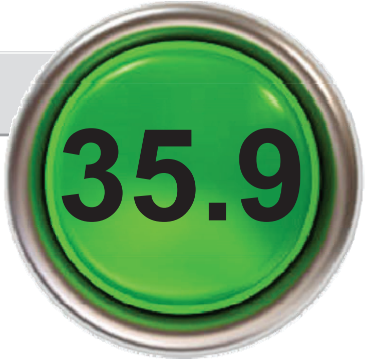

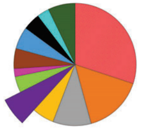

30-40

β2-α2 LOOP
NNQNNF
164

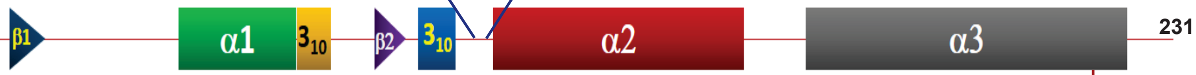


```
WNKPSKPKTNMKHVAGAAAAGAVVGGLGGYMLGSAMSRPLIHFGNDYEDRYYRENMYRYPNQVYYYRPVDQYNNQNNFVHDCVNITIKQHTVTTTTKGENFTETDVKMMERVVEQMCITQYQKEYQAAYQGSAS-
          I                           M      W         N                                                                          V      S   Y E RS
```

PrP sequence differs by 6 amino acids from the bank vole PrP (see in red)

No deletions
No insertions
No polymorphic variants found

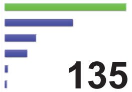

135

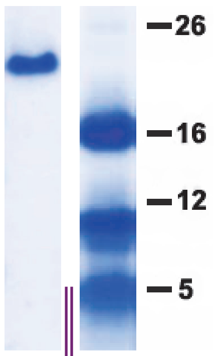

211 AA

1
Conformers

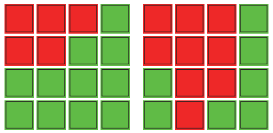

Tm^{Exp}: ND

ΔΔG: -8.51

In vitro studies

TgVole (1x) — Not tested

Cantor's roundleaf bat — Not tested

In vivo studies

TgVole (1x) — Not tested

Cantor's roundleaf bat — Not tested

pLDDT
[84.25]

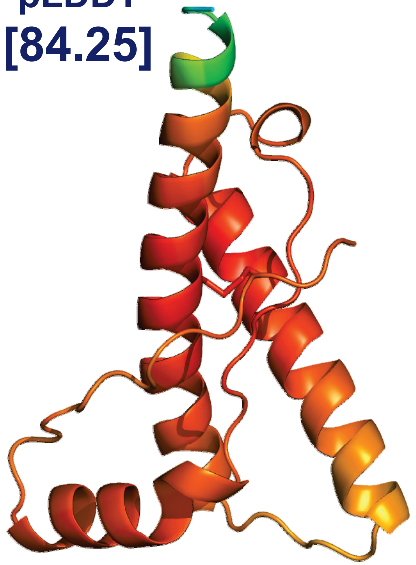

Distinct primary sequence across species

Protein code: 3223

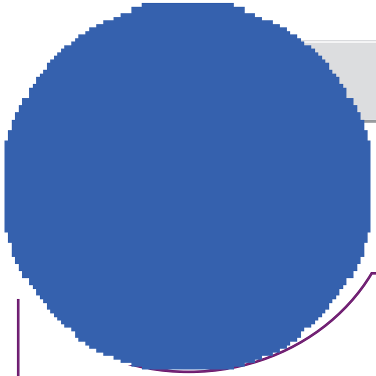

Great evening bat

GenBank: BK064208

Ia io

Chiroptera	23/45
Vespertilionidae	7/16

P29%

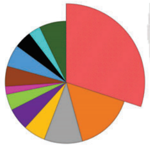

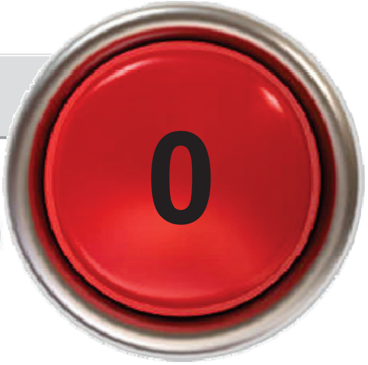

0

β2-α2 LOOP
SNQNNF
273

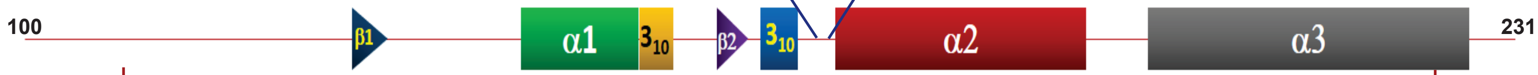

WNKPNKPKTNMKHVAGAAAAGAVVGGLGGYMLGSAMSRPAMHFGNEYEDRYYRENMNRFPEHVYYKPVDQYSNQNNFVRDCVNITVKQHTVTTTTKGENFTETDVKIMERVVEQMCTTQYQKEYQAAYQRGAS-

S I MI DW Y NQ R N H I M V S Y EGRS

PrP sequence differs by 15 amino acids from the bank vole PrP (see in red)

No deletions

No insertions

No polymorphic variants found

Distinct primary sequence across species

Tm^{Exp}: ND

ΔΔG: -13.71

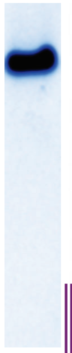

218 AA

NO MISFOLDING

0
Conformers

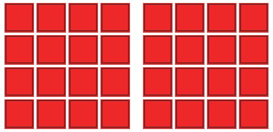

In vitro studies

NOT APPLICABLE

In vivo studies

NOT APPLICABLE

pLDDT
[92.15]

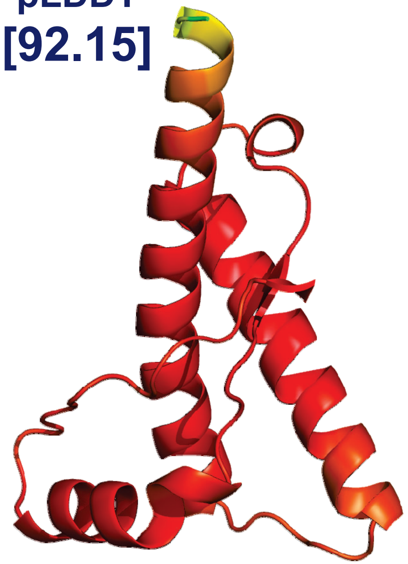

Protein code: 4617

Hoary bat

GenBank: BK063928

Lasiurus cinereus

Chiroptera 23/45

Vespertilionidae 7/16

P39%

3.6

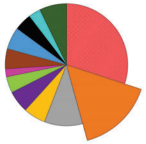

2 species sharing the same primary sequence

Rafinesque's big-eared bat
Corynorhinus rafinesquii

Eastern red bat
Lasiurus borealis

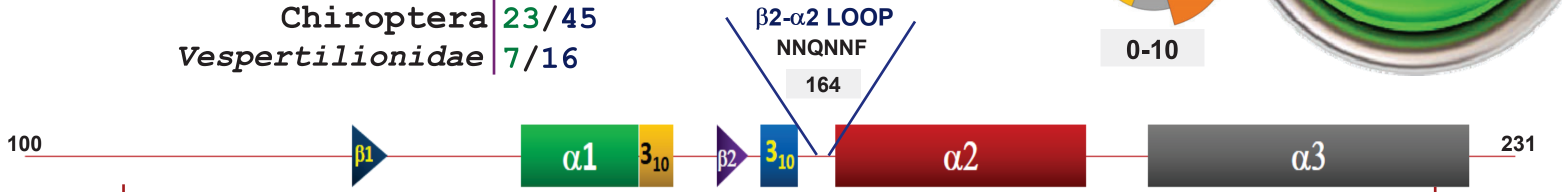


```
WNKPNKPKTNMKHVAGAAAAGAVVGGLGGYMLGSAMSRPAMHFGNEYEDRYYRENMNRFPEQVYYKPVDQYNNQNNFVRDCVNITVKQHTVTTTTKGENFTETDIKIMERVVEEMCTTQYKKEYQASYQRGAS-
     S    I                                MI    DW            Y N    R              H      I                        V M       Q  V    Q  S  Y EGRS
```

PrP sequence differs by 16 amino acids from the bank vole PrP (see in red)

No deletions

No insertions

No polymorphic variants found

135

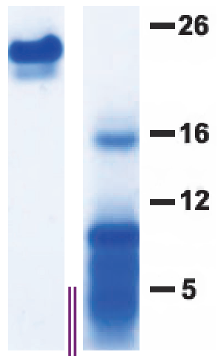

224 AA

1

Conformers

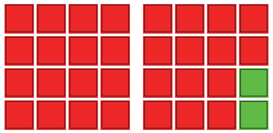

Tm^{Exp}: ND

ΔΔG: -4.45

In vitro studies

TgVole (1x) Not tested

Hoary bat Not tested

In vivo studies

TgVole (1x) Not tested

Hoary bat Not tested

pLDDT [85.37]

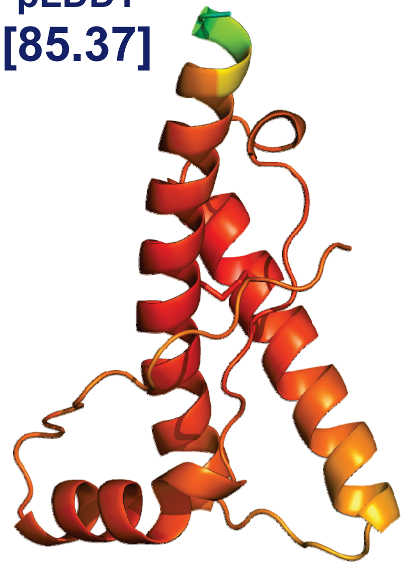

Protein code: 3046

Long-tongued fruit bat

GenBank: BK063991

Macroglossus sobrinus

Chiroptera 23/45
Pteropodidae 8/8

P86%

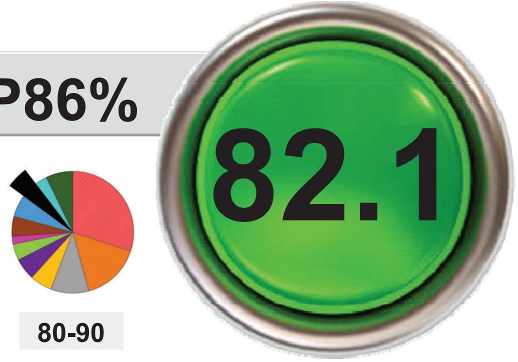

β2-α2 LOOP
NNQNSF
24

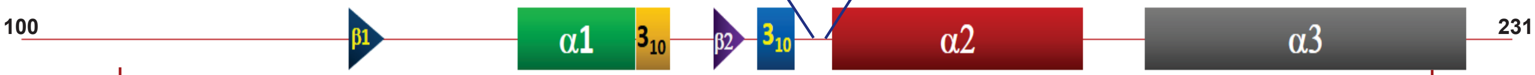

WNKPSKPKTNLKHVAGAAAAGAVVGGLGGYMLGSAMSRPMLHFGNDYEDRYYRENMYRYPNQVYYRPVDQYNNQNSFVHDCVNITVKQHTVTTTTKGENFTETDVKMMERVVEQMCITQYQQESRAAYHRGAS-
I I W N N I V K Q Y EGRS

PrP sequence differs by 9 amino acids from the bank vole PrP (see in red)

No deletions
No insertions
No polymorphic variants found

Distinct primary sequence across species

135

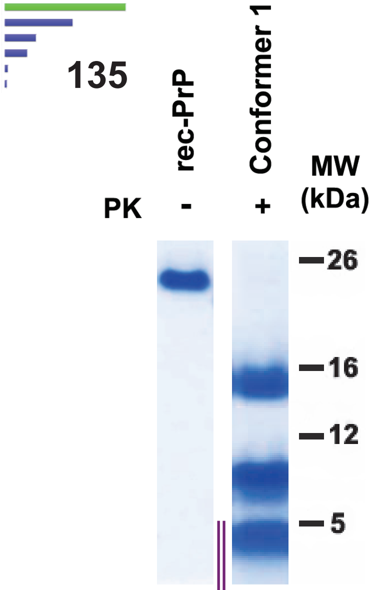

220 AA

1
Conformers

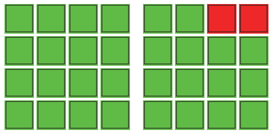

Tm[Exp]: ND

ΔΔG: -14.88

In vitro studies

TgVole (1x) Not tested

Long-tongued fruit bat Not tested

In vivo studies

TgVole (1x) Not tested

Long-tongued fruit bat Not tested

pLDDT
[85.45]

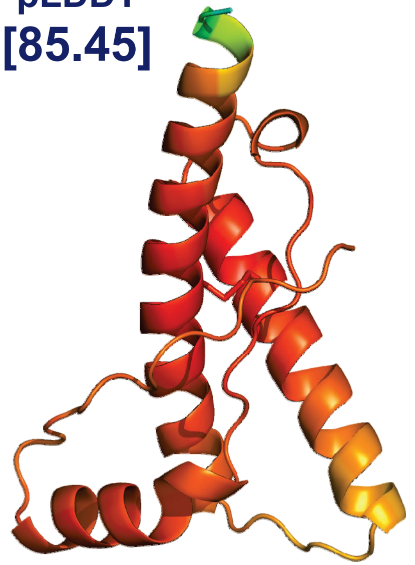

Protein code: 3149

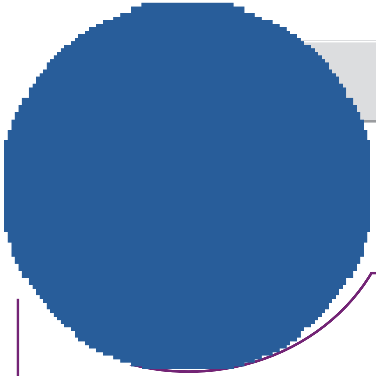

California leaf-nosed bat

GenBank: AY133049

Macrotus californicus

Chiroptera 23/45

Phyllostomidae 2/10

Distinct primary sequence across species

P35%

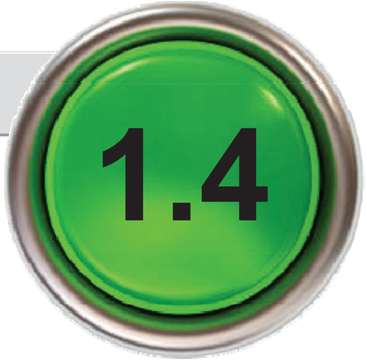

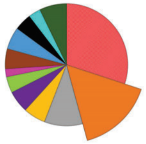

0-10

β2-α2 LOOP
SSQNNF
42

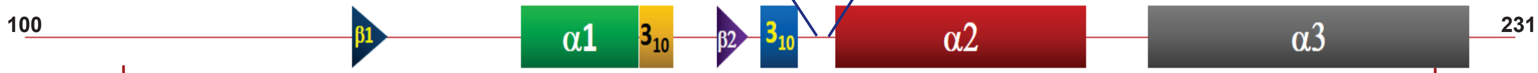

GGKPSKPKTNMKHVAGAAAAGAVVGGLGGYMLGSAMSRPLIHFGNDYEDRYYRENMNRFPNQVYYKPVDQYSSQNNFVRDCVNITVKEHTVTTTTKGENFTETDVKIMQRVVEEMCTIQYQQEFQAYQRGAS-

WN I M W Y R NN H I Q M E Q VT K S YE R S

PrP sequence differs by 17 amino acids from the bank vole PrP (see in red)

No deletions

No insertions

No polymorphic variants found

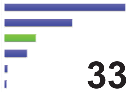

33

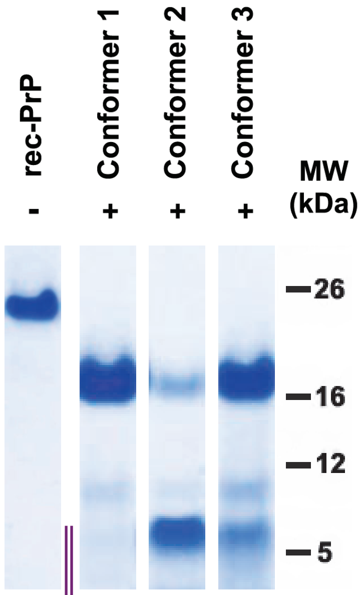

215 AA

3

Conformers

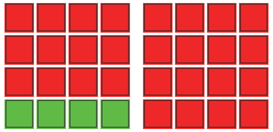

Tm^{Exp}: ND

ΔΔG: ND

In vitro studies

TgVole (1x) Not tested

California leaf-nosed bat Not tested

In vivo studies

TgVole (1x) Not tested

California leaf-nosed bat Not tested

pLDDT
[87.91]

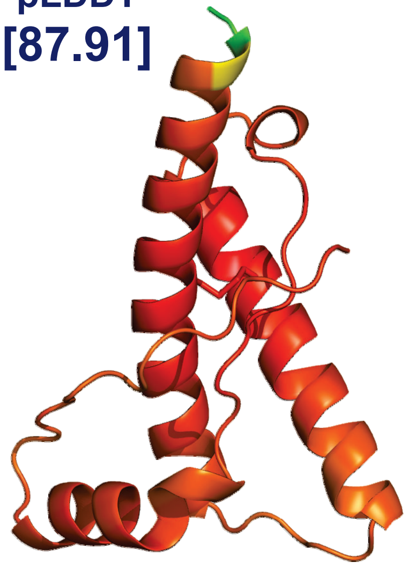

Protein code: 1151

Greater false vampire bat

GenBank: BK064080

Megaderma lyra

Chiroptera 23/45

Megadermatidae 0/1

P29%

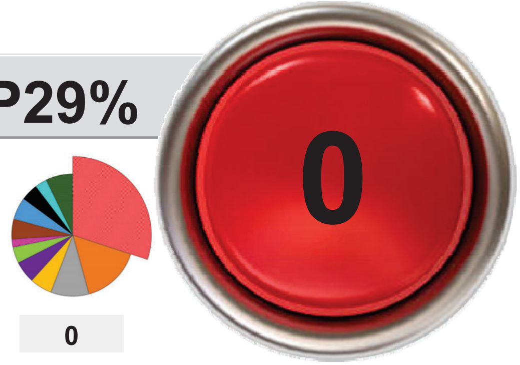

β2-α2 LOOP
SNQNNF
273

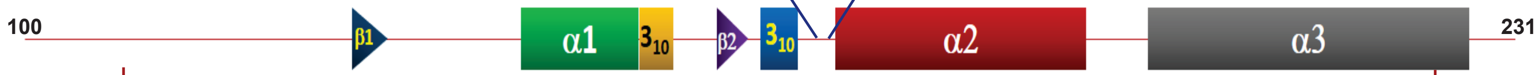


```
WGKPSKPKTNMKHVAGAAAAGAVVGGLGGYMLGSAMSRPMIHFGNDYEDRYYRENMYRYPNQVYYKPVDQYSNQNNFVHDCVNITVKQHTVTTTTKGENFTETDVKMMERVVEQMCITQYQRESQAAAYHRGAS-
 N       I                                  W          N         R      N            I                                          V    K    YYEG SS
```

PrP sequence differs by 8 amino acids from the bank vole PrP (see in red)

No deletions

No insertions

No polymorphic variants found

Distinct primary sequence across species

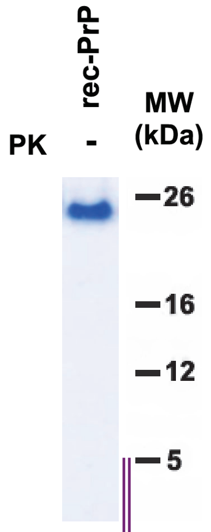

222 AA

NO MISFOLDING

0
Conformers

In vitro studies

NOT APPLICABLE

In vivo studies

NOT APPLICABLE

Tm^{Exp}: ND

ΔΔG: ND

pLDDT
[86.06]

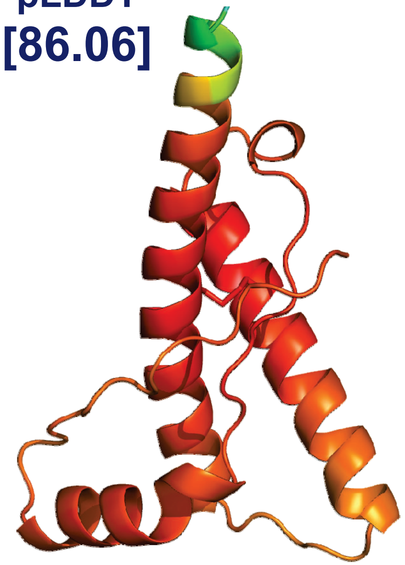

Protein code: 3258

Hairy big-eared bat

GenBank: BK064088

Micronycteris hirsuta

Chiroptera 23/45
Phyllostomidae 2/10

P29%

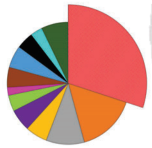

0

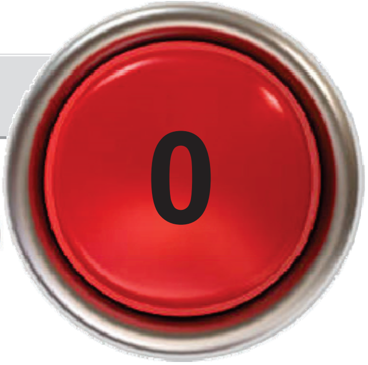

β2-α2 LOOP
SNQNNF
273

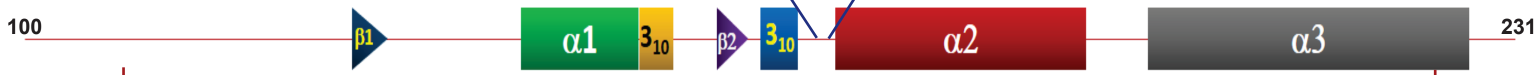

GGKPSKPKTNMKHVAGAAAAGAVVGGLGGYMLGSAMSRPLIHFGNDYEDRYYRDNMNRFPNQVYYRPVDQYSNQNNFVRDCVNITVKEHTVTTTTKGENFTETDVKIMQRVVEEMCTIQYQKEYQAYQSGAYYQRGA
WN I M W E Y N H I Q M E Q VT S YE RSS

PrP sequence differs by 15 amino acids from the bank vole PrP (see in red)

No deletions
No insertions
No polymorphic variants found

Distinct primary sequence across species

Tm[Exp]: ND
ΔΔG: ND

PK -
rec-PrP
MW (kDa)

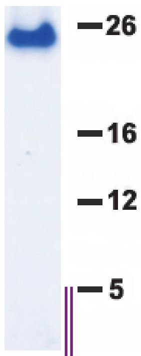

229 AA

NO MISFOLDING

0
Conformers

In vitro studies

NOT APPLICABLE

In vivo studies

NOT APPLICABLE

pLDDT
[86.43]

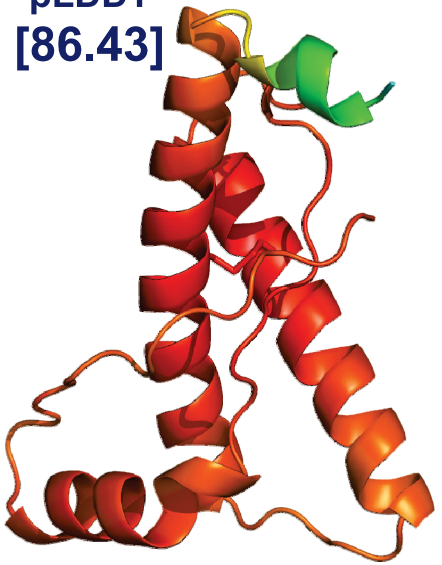

Protein code: 3267

Natal long-fingered bat

GenBank: XM_016212061

Miniopterus natalensis

Chiroptera 23/45
Vespertilionidae 7/16

P72%

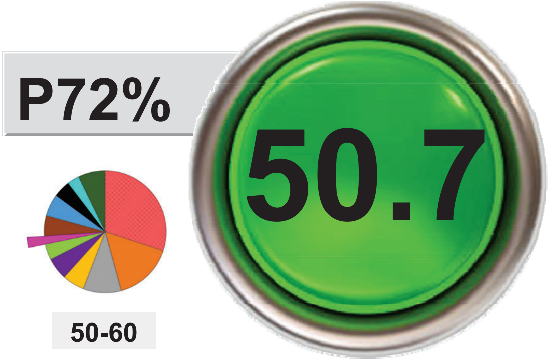

β2-α2 LOOP
NNQDNF
1

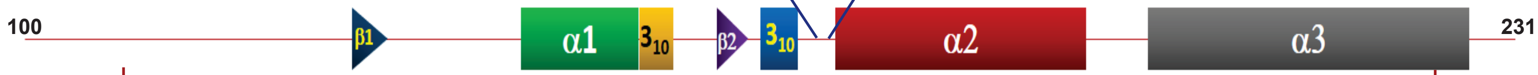


```
WNKPSKPKTNLKHMAGAAAAGAVVGGLGGYMVGSAMSRPLMHFGNDYEDRYYRENMNRFPNQVYYKPVNQYNNQDNFVRDCVNITVKQHTVTTTTKGENFTETDVKIMERVVEEMCTTQYQKEYQAYQSYNGMS-
           I  V          L      MI   W          Y    R  D    N  H     I                   M     Q  V     S   YEGRSS
```

PrP sequence differs by 16 amino acids from the bank vole PrP (see in red)

No deletions
No insertions
No polymorphic variants found

Distinct primary sequence across species

25

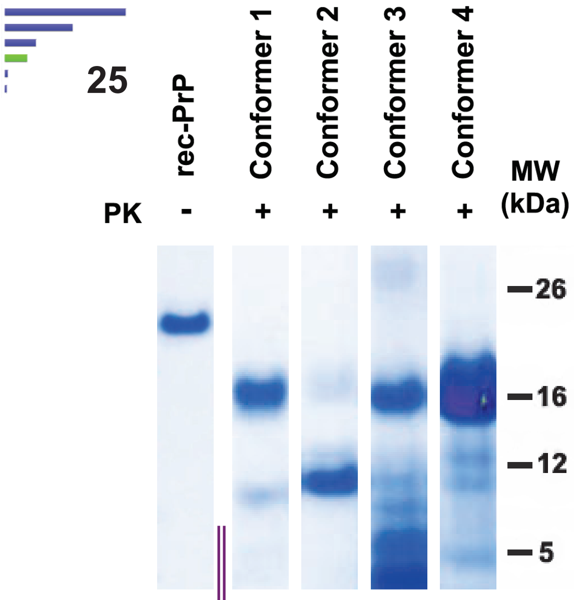

207 AA

4 Conformers

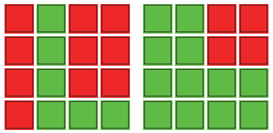

Tm[Exp]: ND
ΔΔG: ND

In vitro studies

TgVole (1x) Not tested
Natal long-fingered bat Not tested

In vivo studies

TgVole (1x) Not tested
Natal long-fingered bat Not tested

pLDDT [92.25]

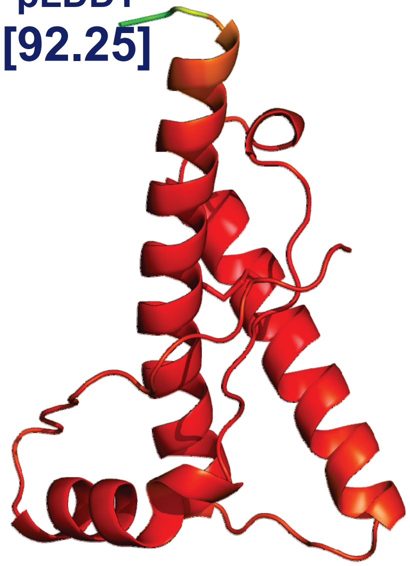

Protein code: 1125

Common bent-wing bat

GenBank: BK064092

Miniopterus schreibersii

Chiroptera 23/45

Vespertilionidae 7/16

P29%

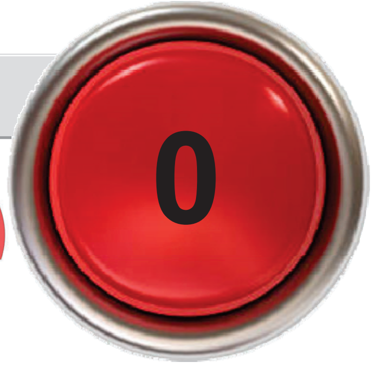

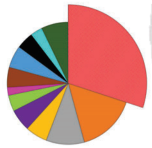

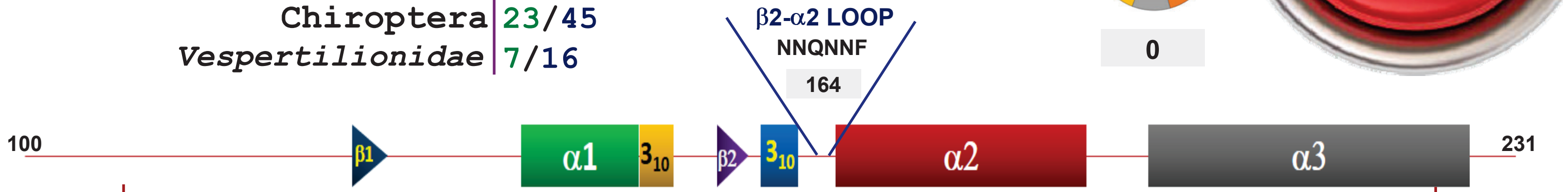

WNKPSKPKTNLKHMAGAAAAGAVVGGLGGYMLGSAMSRPLMHFGNDYEDRYYRENMNRFPNQVYYKPVDQYNNQNNFVRDCVNITVKQHTVTTTTKGENFTETDVKIMERVVEEMCTTQYQKEYQAYRSYNGMS-

I V MI W Y R H I M Q V S YEGRSS

PrP sequence differs by 13 amino acids from the bank vole PrP (see in red)

No deletions

No insertions

No polymorphic variants found

Tm^{Exp}: ND

ΔΔG: ND

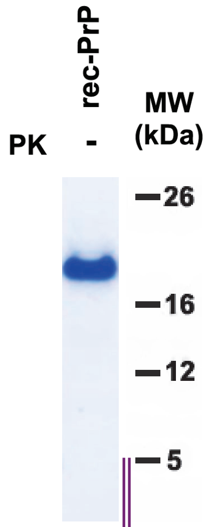

207 AA

NO MISFOLDING

0

Conformers

In vitro studies

NOT APPLICABLE

In vivo studies

NOT APPLICABLE

pLDDT [92.8]

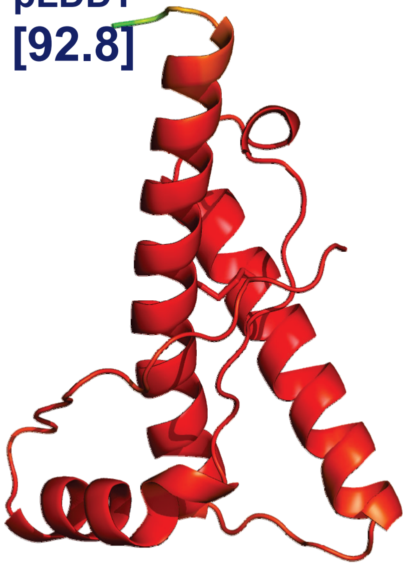

Distinct primary sequence across species

Protein code: 3271

Velvety free-tailed bat

GenBank: KAF6472180

Molossus molossus

Chiroptera 23/45
Molossidae 2/2

P55%

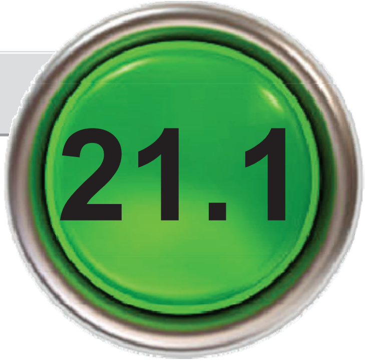

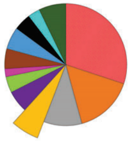

2 species sharing the same primary sequence

Alvarez's mastiff bat *Molossus alvarezi*

Northern black mastiff bat *Molossus rufus*

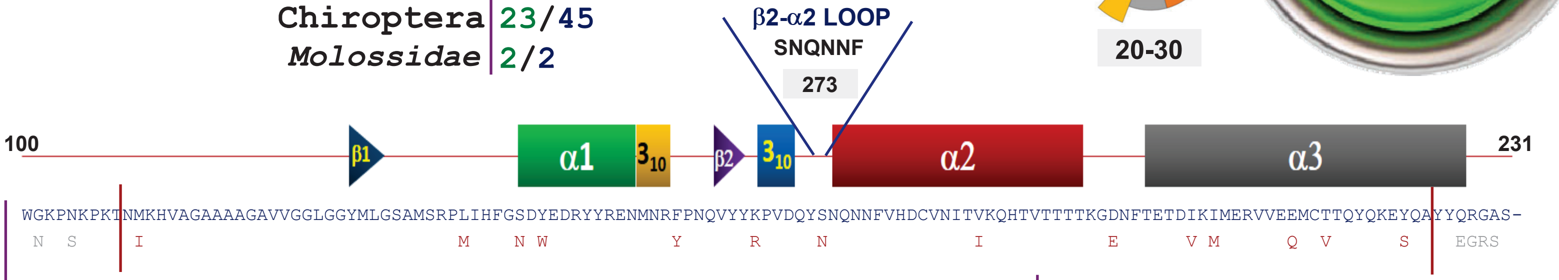

PrP sequence differs by 14 amino acids from the bank vole PrP (see in red)

No deletions
No insertions
No polymorphic variants found

76

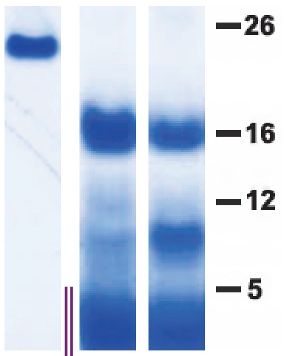

225 AA

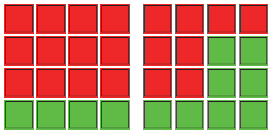

Tm^{Exp}: ND

ΔΔG: -7.17

In vitro studies

TgVole (1x) Not tested

Velvety free-tailed bat Not tested

In vivo studies

TgVole (1x) Not tested

Velvety free-tailed bat Not tested

pLDDT [85.78]

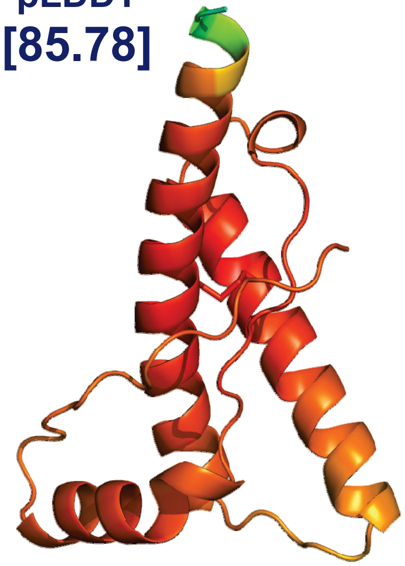

Protein code: 2386

Antillean ghost-faced bat

GenBank: BK063931

Mormoops blainvillei

Chiroptera	23/45
Mormoopidae	0/2

Distinct primary sequence across species

P29%

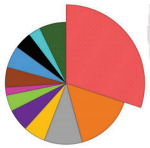

0

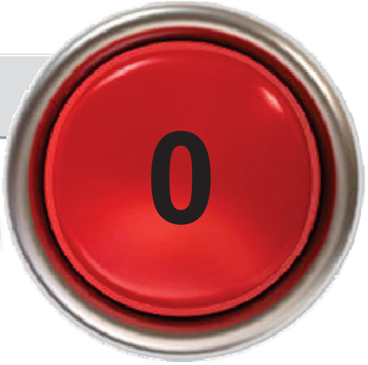

β2-α2 LOOP
SNQNNF
273

100 β1 α1 3_{10} β2 3_{10} α2 α3 231

GGKPSKPKTNMKHVAGAAAAGAVVGGLGGYMLGSAMSRPLIHFGNDYEDRYYRENMNRFPNQVYYKPVDQYSNQNNFVRDCVNITVKEHTVTTTTKGENFTETDVKIMERVVEEMCTIQYQKEYQTYYQRGAS-

WN I M W Y R N H I Q M Q VT S A EGRS

PrP sequence differs by 15 amino acids from the bank vole PrP (see in red)

No deletions

No insertions

No polymorphic variants found

Tm^{Exp}: ND

ΔΔG: -14.83

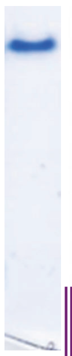

227 AA

NO MISFOLDING

0 Conformers

In vitro studies

NOT APPLICABLE

In vivo studies

NOT APPLICABLE

pLDDT [85.66]

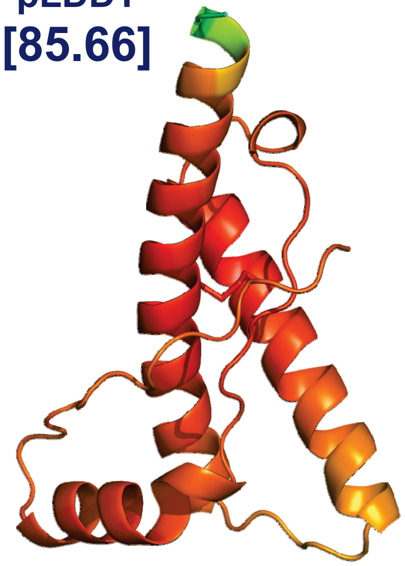

Protein code: 3049

Little tube-nosed bat

GenBank: BK064100

Murina aurata feae

Chiroptera 23/45
Vespertilionidae 7/16

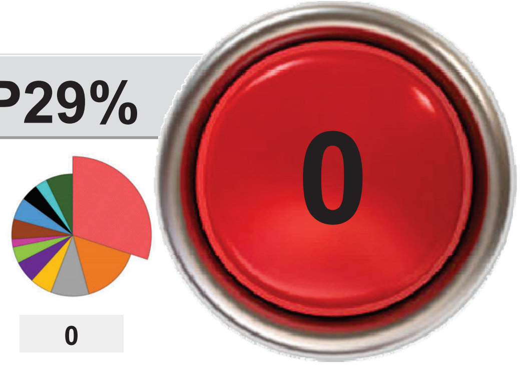

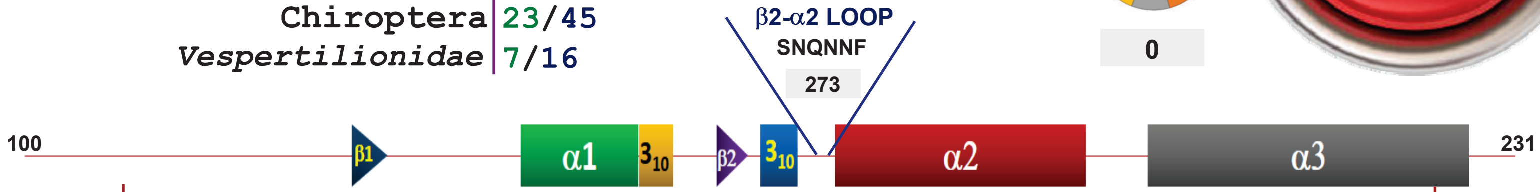

WNKPNKPKTNMKHVAGAAAAGAVVGGLGGYMLGSAMSRPAMHFGNEYEDRYYRENMNRFPNQVYYKPVDQYSNQNNFVRDCVNITIKEHTVTTITKGENITETDVKIMERVVEEMCTTQYQKEYQASYQRGAS-
S I MI DW Y R N H Q T F M Q V S Y EGRS

PrP sequence differs by 16 amino acids from the bank vole PrP (see in red)

No deletions
No insertions
No polymorphic variants found

Distinct primary sequence across species

Tm^{Exp}: ND

ΔΔG: -7.85

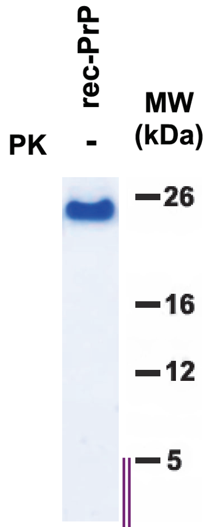

224 AA

NO MISFOLDING

0 Conformers

In vitro studies

NOT APPLICABLE

In vivo studies

NOT APPLICABLE

pLDDT [86.31]

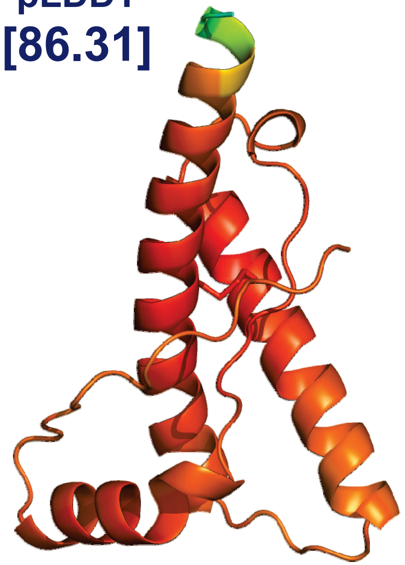

Protein code: 3279

Brandt's bat

GenBank: XM_005874678

Myotis brandtii

Chiroptera 23/45
Vespertilionidae 7/16

P41%

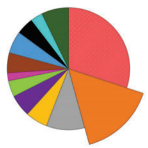

0-10

7.7

1 species sharing the same primary sequence

Little brown bat
Myotis lucifugus

β2-α2 LOOP
NNQNNF
164

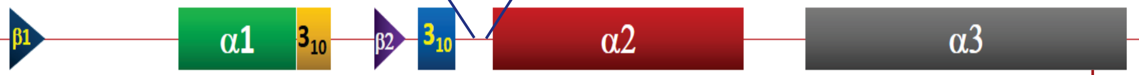

WNKPNKPKTNMKHVAGAAAAGAVVGGLGGYMLGSAMSRPPMHFGNEYEDRYYRENMNRFPDQVYYKPVDQYNNQNNFVRDCVNITVKQHTVTTITKGENITETDVKIMERVVEEMCTTQYQKEYQASYQRGAS-

S I MI DW Y N R H I T F M Q V S Y EGRS

PrP sequence differs by 16 amino acids from the bank vole PrP (see in red)

No deletions
No insertions
No polymorphic variants found

76

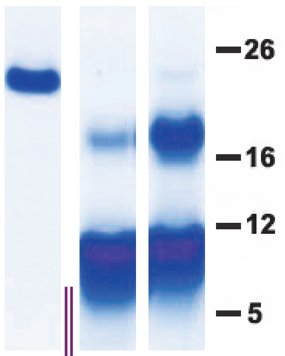

207 AA

2 Conformers

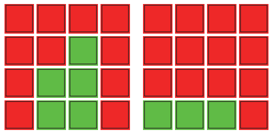

Tm^{Exp}: ND

ΔΔG: -12.24

In vitro studies

TgVole (1x)	Not tested
Brandt's bat	Not tested

In vivo studies

TgVole (1x)	Not tested
Brandt's bat	Not tested

pLDDT [86.64]

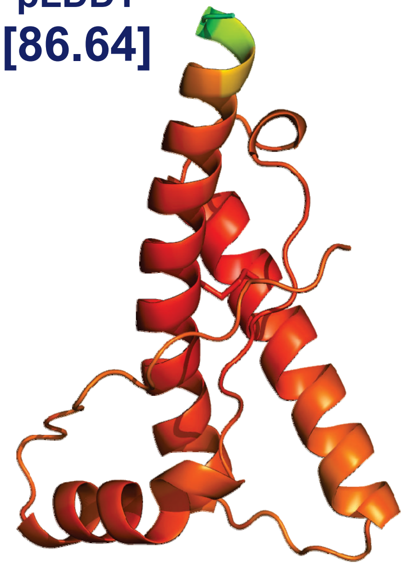

Protein code: 1102

David's myotis

GenBank: XM_006758058

P41%

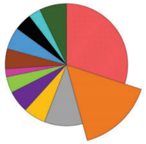

0-10

7.1

Myotis davidii

Chiroptera 23/45

Vespertilionidae 7/16

2 species sharing the same primary sequence

Daubenton's bat
Myotis daubentonii

Greater mouse-eared bat
Myotis myotis

β2-α2 LOOP
NNQNNF
164

100 β1 α1 3_{10} β2 3_{10} α2 α3 231

WNKPNKPKTNMKHVAGAAAAGAVVGGLGGYMLGSAMSRPPIHFGNEYEDRYYRENMNRFPNQVYYKPVDQYNNQNNFVRDCVNITVKQHTVTTTITKGENITETDVKIMERVVEEMCTTQYQKEYQASYQRGAS-

S I M DW Y R H I T F M Q V S Y EGRS

PrP sequence differs by 14 amino acids from the bank vole PrP (see in red)

No deletions
No insertions
No polymorphic variants found

135

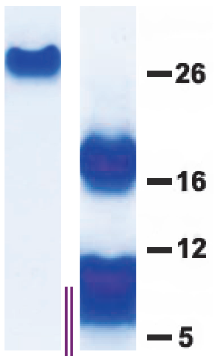

215 AA

1
Conformers

Tm^{Exp}: ND

ΔΔG: -13.43

In vitro studies

TgVole (1x) Not tested

David's myotis Not tested

In vivo studies

TgVole (1x) Not tested

David's myotis Not tested

pLDDT
[86.29]

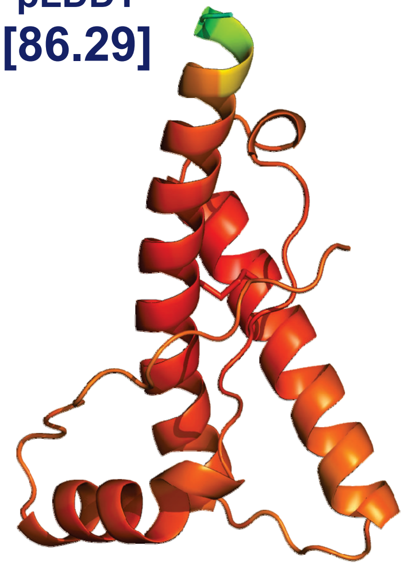

Protein code: 1132

Rickett's big-footed bat

GenBank: BK064146

P29%

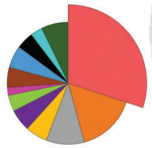

0

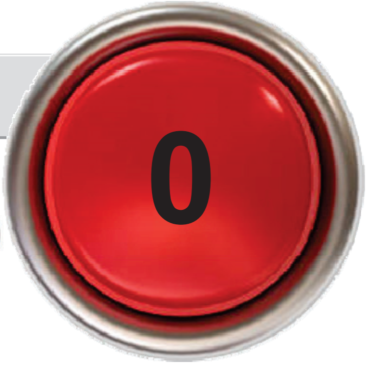

Myotis ricketti

Chiroptera 23/45
Vespertilionidae 7/16

β2-α2 LOOP
NNQNNF
164

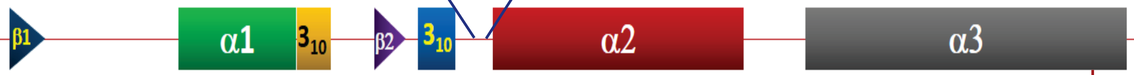


```
WNKPNKPKTNMKHMAGAAAAGAVVGGLGGYMLGSAMSRPPIHFGNEYEDRYYRENMNRFPNQVYYKPVDQYNNQNNFVRDCVNITVKQHTVTTITKGENITETDVKIMERVVEEMCTTQYQKEYQASYQRGAS-
      S    I  V                        M     DW           Y      R            H      I       T     F      M      Q  V        S  Y EGRS
```

PrP sequence differs by 15 amino acids from the bank vole PrP (see in red)

No deletions
No insertions
No polymorphic variants found

Distinct primary sequence across species

Tm^{Exp}: ND

ΔΔG: -12.32

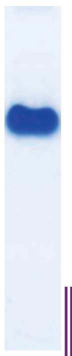

188 AA

NO MISFOLDING

0 Conformers

In vitro studies

NOT APPLICABLE

In vivo studies

NOT APPLICABLE

pLDDT
[86.53]

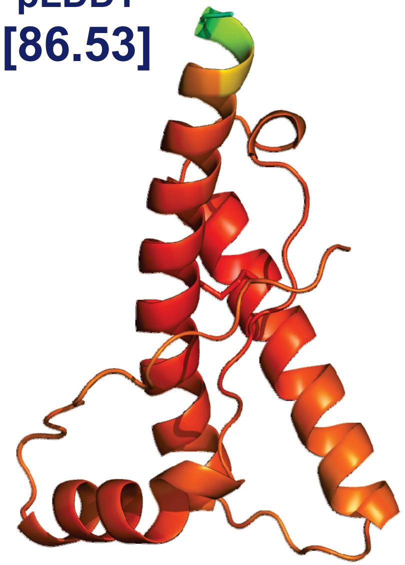

Protein code: 3338

Northern long-eared bat

GenBank: BK064803

Myotis septentrionalis

Chiroptera 23/45
Vespertilionidae 7/16

P45%

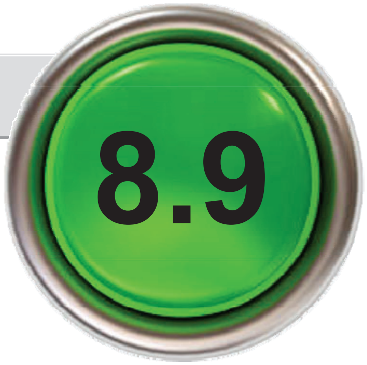

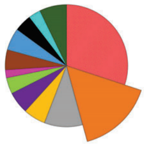

0-10

β2-α2 LOOP
NNQNNF
164

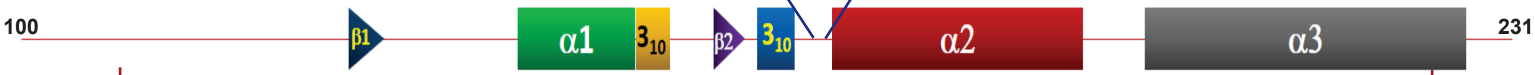

WNKPNKPKTNMKHVAGAAAAGAVVGGLGGYMLGSAMSRPPMHFGNEYEDRYYRENMNRFPNQVYYKPVDQYNNQNNFVRDCVNITVKQHTVTTITKGENITETDVKIMERVVEEMCTTQYQKEYQASYQRGAS-

S I MI DW Y R H I T F M Q V S Y EGRS

PrP sequence differs by 15 amino acids from the bank vole PrP (see in red)

No deletions
No insertions
No polymorphic variants found

Distinct primary sequence across species

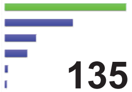

135

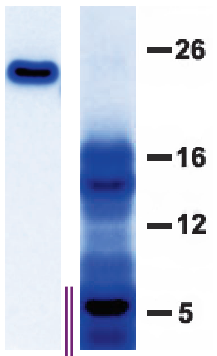

216 AA

1
Conformers

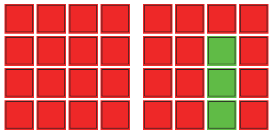

Tm^{Exp}: ND

ΔΔG: -10.06

In vitro studies

Not tested
TgVole (1x)

Not tested
Northern long-eared bat

In vivo studies

Not tested
TgVole (1x)

Not tested
Northern long-eared bat

pLDDT
[86.12]

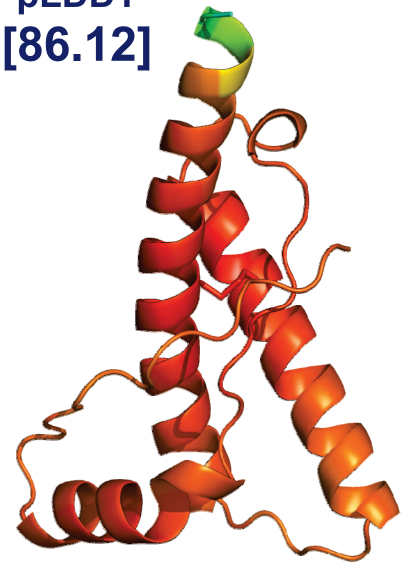

Protein code: 3970

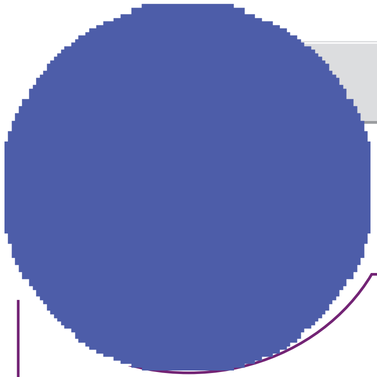

Yuma myotis

GenBank: BK064222

Myotis yumanensis

Chiroptera 23/45
Vespertilionidae 7/16

P29%

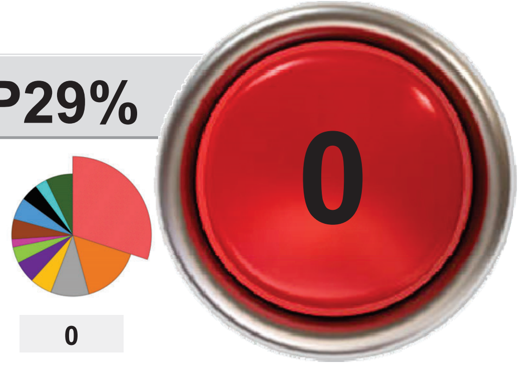

β2-α2 LOOP
NNQNNF
164

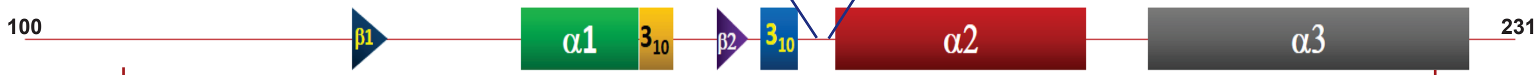

WNKPNKPKTNMKHVAGAAAAGAVVGGLGGYMLGSAMSRPPMHFGNEYEDRYYRENMNRFPNQVYYKPVDQYNNQNNFVRDCVNITVKQHMVTTTITKGENITETDVKIMERVVEEMCTTQYQKEYQASYQRGAS-

S I MI DW Y R H I T T F M Q V S Y EGRS

PrP sequence differs by 16 amino acids from the bank vole PrP (see in red)

No deletions
No insertions
No polymorphic variants found

Distinct primary sequence across species

Tm^{Exp}: ND

ΔΔG: -17.57

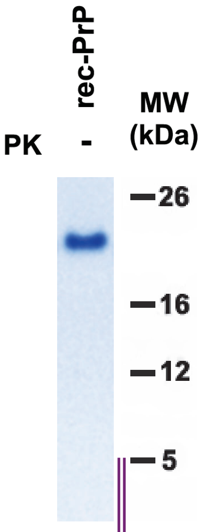

216 AA

NO MISFOLDING

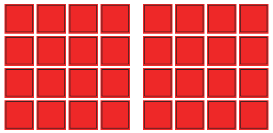

0
Conformers

In vitro studies

NOT APPLICABLE

In vivo studies

NOT APPLICABLE

pLDDT
[92.56]

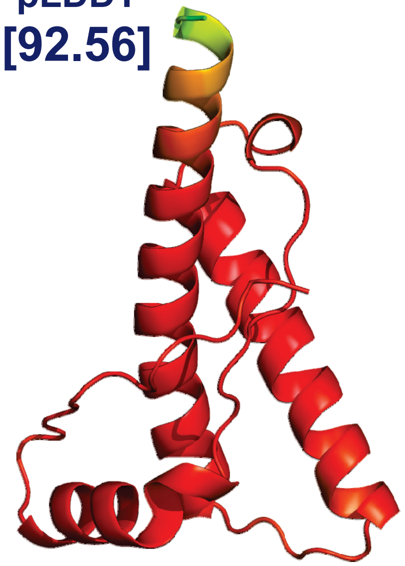

Protein code: 4819

Greater bulldog bat

GenBank: BK064113

Noctilio leporinus

Chiroptera 23/45

Noctilionidae 0/1

P29%

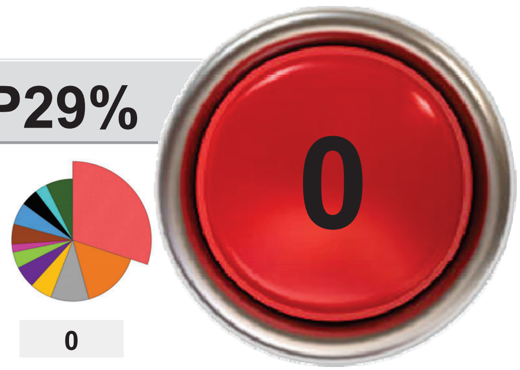

β2-α2 LOOP
NNQNNF
164

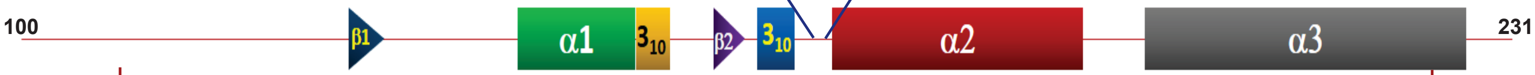

GGKPSKPKTNMKHVAGAAAAGAVVGGLGGYMLGSAMSRPLIHFGNDYEDRYYRENMNRFPNQVYYRPVDQYNNQNNFVRDCVNITVKEHTVTTTTKGDNFTETDVKIMERVVEEMCTIQYQKEYAAFQRGAS-

WN I M W Y H I Q E M Q VT SQ YYE R S

PrP sequence differs by 14 amino acids from the bank vole PrP (see in red)

No deletions

No insertions

No polymorphic variants found

Distinct primary sequence across species

Tm^{Exp}: ND

ΔΔG: ND

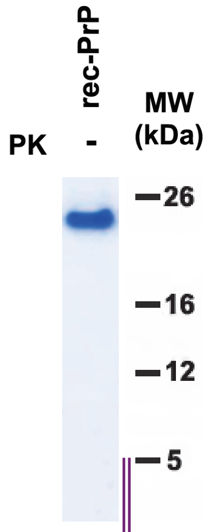

222 AA

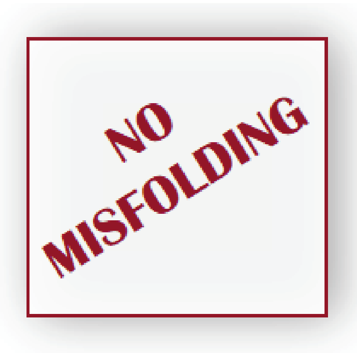

0

Conformers

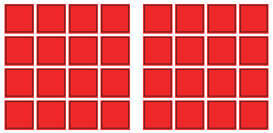

In vitro studies

NOT APPLICABLE

In vivo studies

NOT APPLICABLE

pLDDT

[86.94]

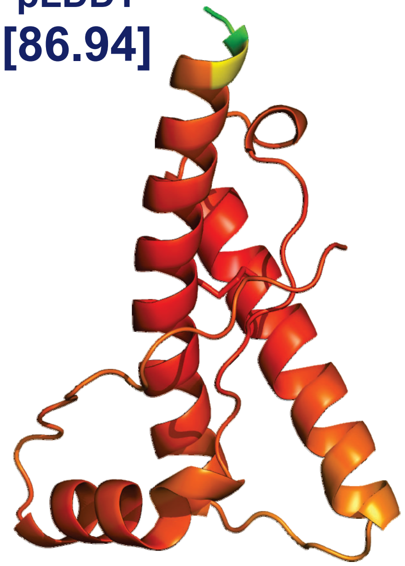

Protein code: 3293

Evening bat

GenBank: BK064115

Nycticeius humeralis

Chiroptera 23/45
Vespertilionidae 7/16

P29%

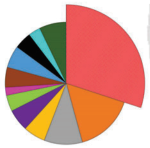

0

0

β2-α2 LOOP
NNQNNF
164

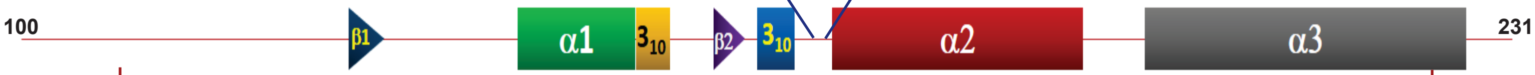


```
WNKPSKPKTNMKHVAGAAAAGAVVGGLGGYMLGSAMSRPTMHFGNEYEDRYYRENMNRFPEQVYYKPVDQYNNQNNFVRDCVNITVKQHTVTTTTKGENFTETDIKIMERVVEEMCTTQYQKEYRASYQRGAS-
          I                              MI    DW              Y N   R               H     I                      V M        Q  V      SQ Y EGRS
```

PrP sequence differs by 16 amino acids from the bank vole PrP (see in red)

No deletions
No insertions
No polymorphic variants found

Distinct primary sequence across species

Tm^{Exp}: ND

ΔΔG: -7.38

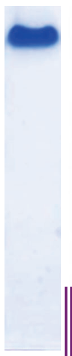

227 AA

NO MISFOLDING

0
Conformers

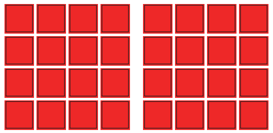

In vitro studies

NOT APPLICABLE

In vivo studies

NOT APPLICABLE

pLDDT
[85.14]

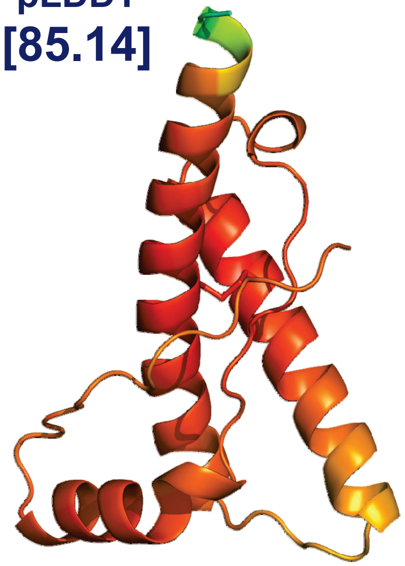

Protein code: 3295

Pale spear-nosed bat

GenBank: XP_035865486

Phyllostomus discolor

Chiroptera 23/45
Phyllostomidae 2/10

Distinct primary sequence across species

P29%

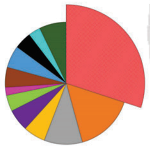

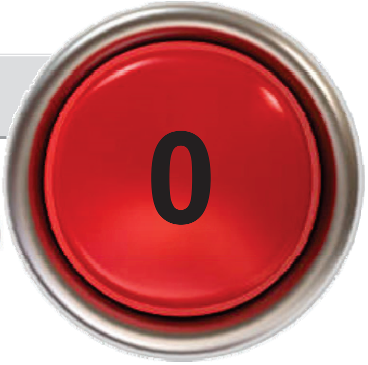

0

β2-α2 LOOP
SNQNNF
273

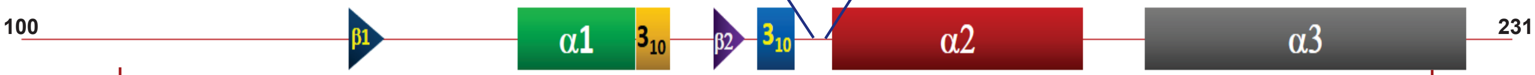


```
GGKPSKPKTNMKHVAGAAAAGAVVGGLGGYMLGSAMSRPLIHFGSDYEDRYYRENMNRFPNQVYYKPVDQYSNQNNFVRDCVNITVKEHTVTTTTKGENFTETDVKIMERVVEEMCTIQYQKEYQAYQRGAS-
WN        I                               M    N W          Y     R     N     H     I Q                     M     Q  VT    S  YE R S
```

PrP sequence differs by 15 amino acids from the bank vole PrP (see in red)

No deletions
No insertions
It shows polymorphic variants

Tm^{Exp}: ND

ΔΔG: ND

PK
rec-PrP
-
MW (kDa)

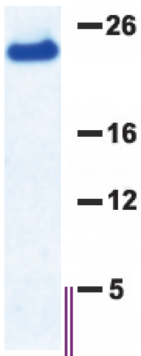

225 AA

NO MISFOLDING

0
Conformers

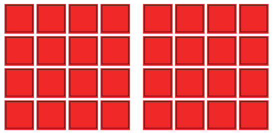

In vitro studies

NOT APPLICABLE

In vivo studies

NOT APPLICABLE

pLDDT
[87.53]

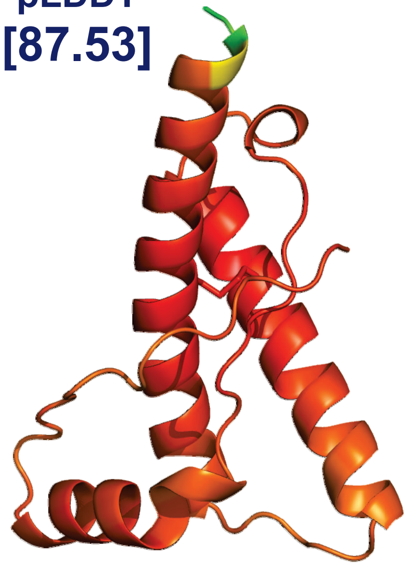

Protein code: 3765

Kuhl's pipistrelle

GenBank: XM_036417932

P39%

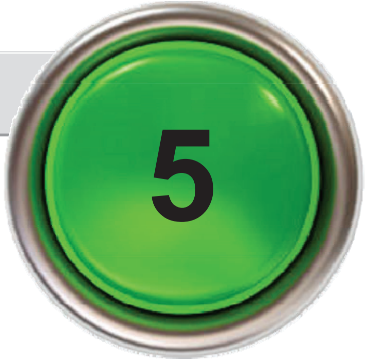

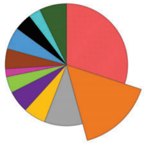

0-10

Pipistrellus kuhlii

Chiroptera 23/45

Vespertilionidae 7/16

β2-α2 LOOP
SNQNNF
273

100

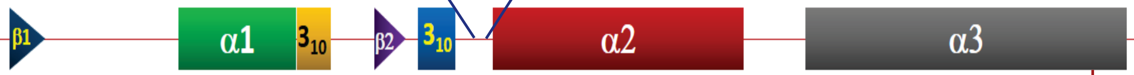

231

WNKPNKPKTNMKHVAGAAAAGAVVGGLGGYMLGSAMSRPAMHFGNEYEDRYYRENMNRFPEQVYYKPVNQYSNQNNFVRDCVNITVKQHTVTTTTKGENFTETDVKIMERVVEEMCTTQYQKEYQAAYQRGSS-

S I MI DW Y N R D N H I M Q V S Y EGR

PrP sequence differs by 16 amino acids from the bank vole PrP (see in red)

No deletions

No insertions

No polymorphic variants found

1 species sharing the same primary sequence

Common pipistrelle
Pipistrellus pipistrellus

135

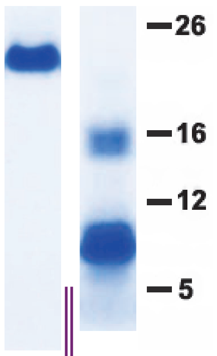

218 AA

1
Conformers

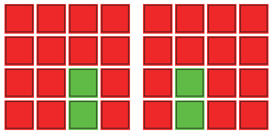

Tm[Exp]: ND

ΔΔG: -3.52

In vitro studies

TgVole (1x) Not tested

Kuhl's pipistrelle Not tested

In vivo studies

TgVole (1x) Not tested

Kuhl's pipistrelle Not tested

pLDDT
[85.05]

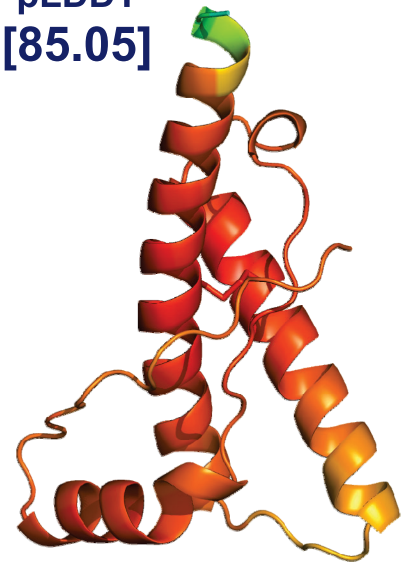

Protein code: 2535

Lump-nosed bat

GenBank: OR472453

Plecotus auritus

Chiroptera 23/45
Vespertilionidae 7/16

P29%

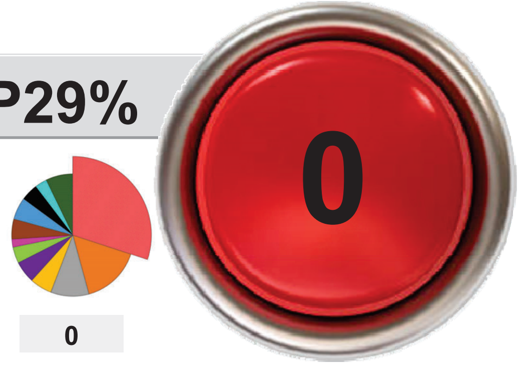

β2-α2 LOOP
NNQNNF
164

100 β1 α1 3_{10} β2 3_{10} α2 α3 231

WNKPNKPKTNMKHMAGAAAAGAVVGGLGGYMLGSAMSRPAMHFGNEYEDRYYRENMNRFPEQVYYKPVDQYNNQNNFVRDCVNITVKQHTVTTTTKGDNFTETDIKIMERVVEEMCTTQYQKEYQASYQRGAS-

S I V MI DW Y N R H I E V M Q V S Y EGRS

PrP sequence differs by 17 amino acids from the bank vole PrP (see in red)

No deletions
No insertions
No polymorphic variants found

Distinct primary sequence across species

Tm^{Exp}: ND

ΔΔG: -3.01

PK
rec-PrP
-
MW (kDa)

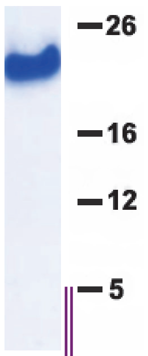

218 AA

NO MISFOLDING

0
Conformers

In vitro studies

NOT APPLICABLE

In vivo studies

NOT APPLICABLE

pLDDT
[84.99]

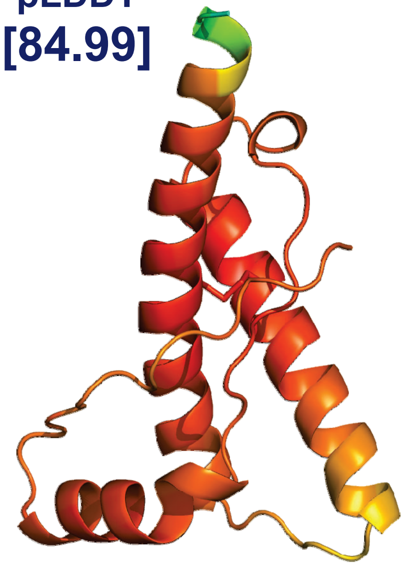

Protein code: 0114

Parnell's mustached bat

GenBank: BK063967

Pteronotus parnellii

Chiroptera 23/45

Mormoopidae 0/2

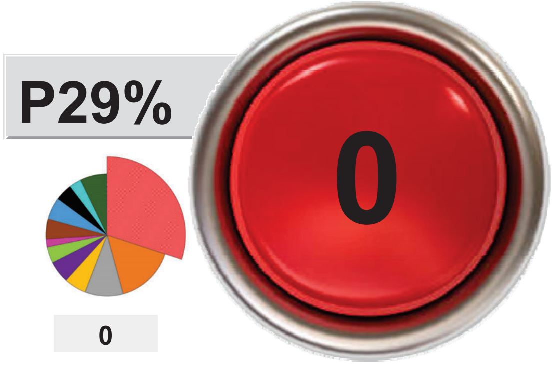

β2-α2 LOOP
SNQNTF
75

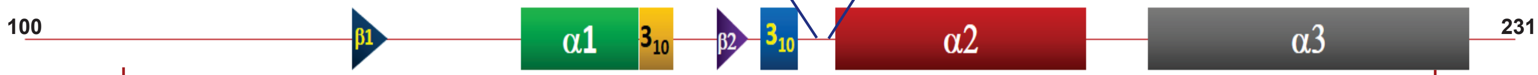

GGKPSKPKTNMKHVAGAAAAGAVVGGLGGYMLGSAMSRPLIHFGNDYEDRYYRENMHRFPDQVYYRPVDQY
SNQNTFVRDCVNITVKEHTVTTTTKGENFTETDVKIMQRVVEEMCTIQYQKEYQAYQRGAS-

WN I M W N Y N N N H I Q M E Q VT S YE R S

PrP sequence differs by 17 amino acids from the bank vole PrP (see in red)

No deletions

No insertions

No polymorphic variants found

Tm^{Exp}: ND

ΔΔG: ND

Distinct primary sequence across species

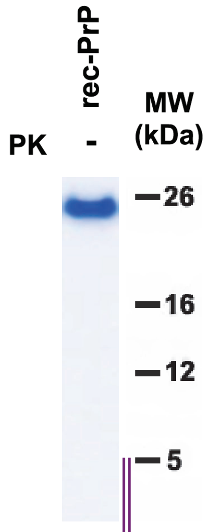

223 AA

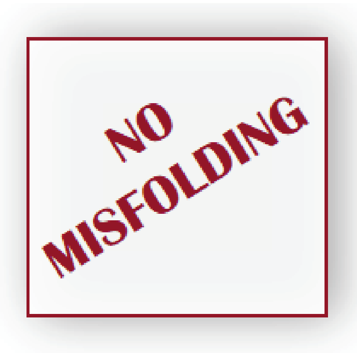

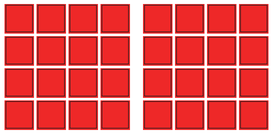

0

Conformers

In vitro studies

NOT APPLICABLE

In vivo studies

NOT APPLICABLE

pLDDT [87.17]

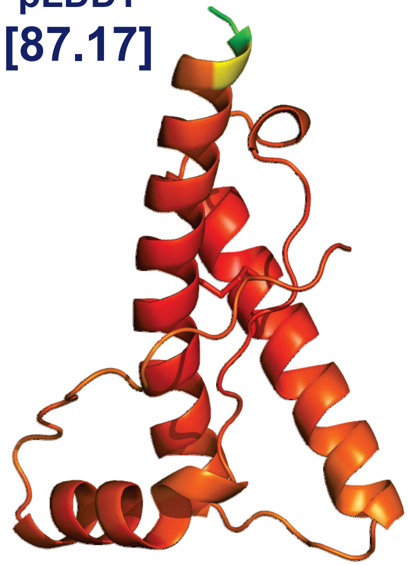

Protein code: 3097

Black flying fox

GenBank: XM_015597302

Pteropus alecto

Chiroptera 23/45
Pteropodidae 8/8

P92%

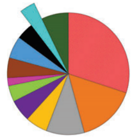

90-100

96.4

5 species sharing the same primary sequence

Indian flying fox *Pteropus medius*

Bonin flying fox *Pteropus pselaphon*

Rodrigues flying fox *Pteropus rodricensis*

Madagascan flying fox *Pteropus rufus*

Large flying fox *Pteropus vampyrus*

β2-α2 LOOP
NNQNSF
24

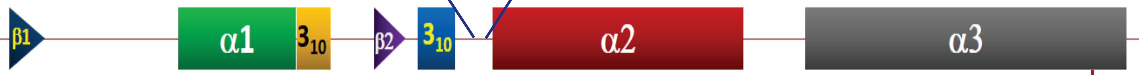


```
WNKPSKPKTNLKHVAGAAAAGAVVGGLGGYMLGSAMNRPLLHFGNDYEDRYYRENMYRYPNQVYYYRPVDQYNNQNSFVHDCVNITVKQHTVTTTTKGENFTETDVKMMERVVEQMCITQYQQESRAAYHRGAS-
           I                           S  MI    W         N                    N         I                                   V    K   Q Y EGRS
```

PrP sequence differs by 11 amino acids from the bank vole PrP (see in red)

No deletions
No insertions
No polymorphic variants found

135

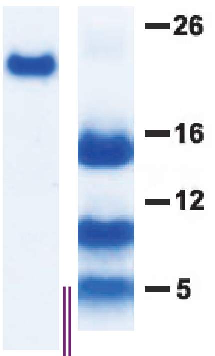

220 AA

1
Conformers

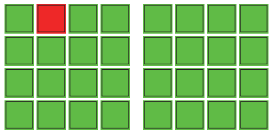

Tm^Exp: ND

ΔΔG: -6.57

In vitro studies

TgVole (1x) Not tested

Black flying fox Not tested

In vivo studies

TgVole (1x) Not tested

Black flying fox Not tested

pLDDT
[84.65]

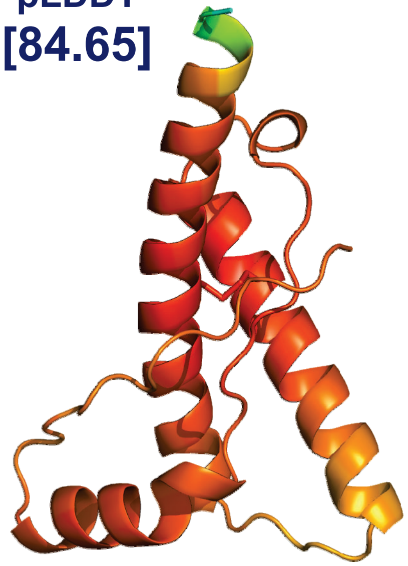

Protein code: 1101

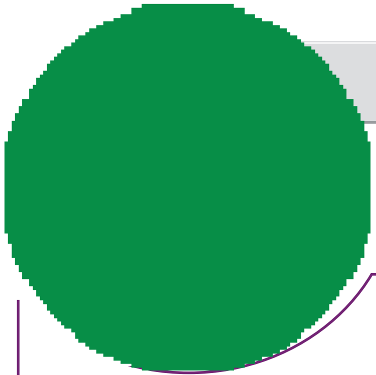

Greater horseshoe bat

GenBank: BK063922

Rhinolophus ferrumequinum

`Chiroptera` 23/45
Rhinolophidae 1/2

Distinct primary sequence across species

P29%

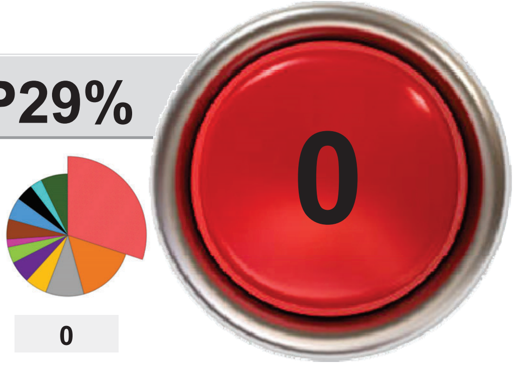

β2-α2 LOOP
NNQNNF
164

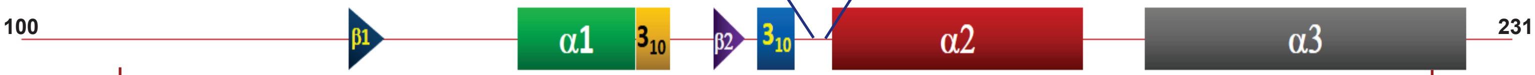

WNKPSKPKTSMKHMAGAAAAGAVAGGLGGYMLGSAMSRPLMHFGNDYEDRYYRENMYRYPNQVYYKPVEQYNNQNNFVHDCVNITIKQHTVTTTTKGENFTETDVKIMERVVEQMCITQYQKESHAAFRRDAS-
NI V V MI W N R D M V Q YYEGRS

PrP sequence differs by 13 amino acids from the bank vole PrP (see in red)

No deletions
No insertions
No polymorphic variants found

Tm[Exp]: ND

ΔΔG: -6.69

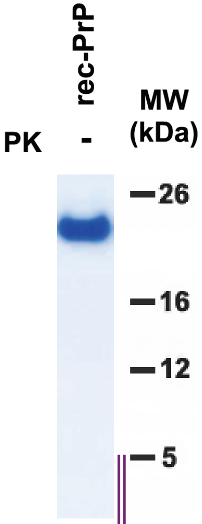

212 AA

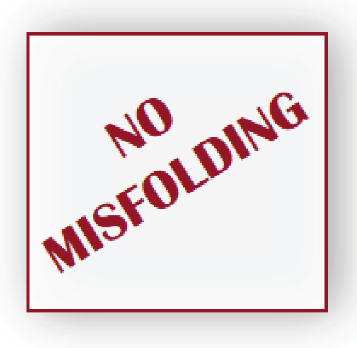

0
Conformers

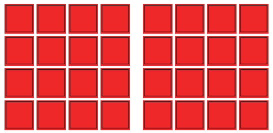

In vitro studies

NOT APPLICABLE

In vivo studies

NOT APPLICABLE

pLDDT
[88.61]

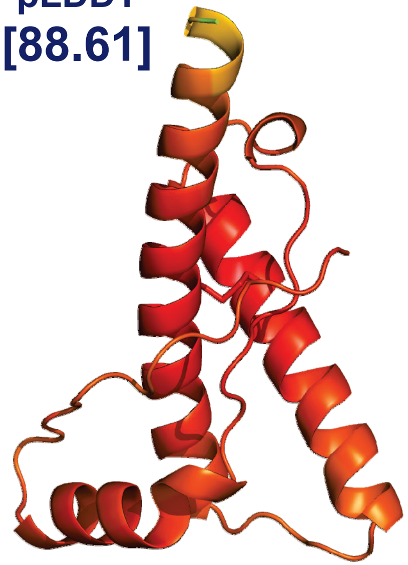

Protein code: 2030

Chinese rufous horseshoe bat

GenBank: BK063992

Rhinolophus sinicus

Chiroptera 23/45
Rhinolophidae 1/2

Distinct primary sequence across species

P34%

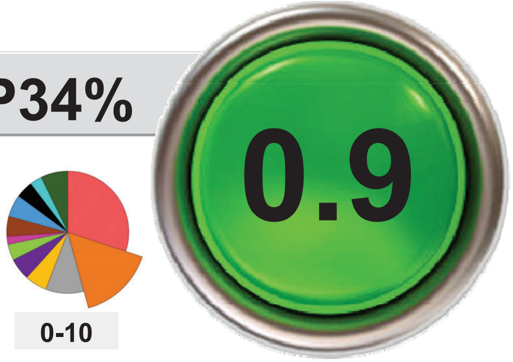

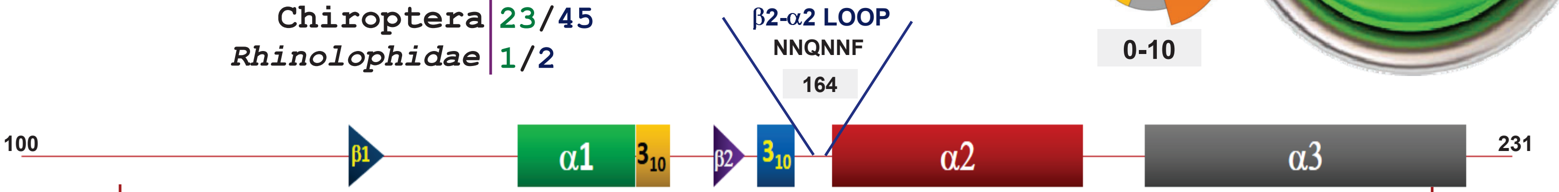

WNKPSKPKTSMKHMAGAAAAGAVAGGLGGYMLGSAMSRPPMHFGNDYEDRYYRENMYRYPNQVYYKPVEQYNNQNNFVHDCVNITIKQHTVTTTSKGENFTETDVKIMERVVEQMCITQYQKEYYASPHRDAS-

NI V V MI W N R D T M V SQ YYEGRS

PrP sequence differs by 15 amino acids from the bank vole PrP (see in red)

No deletions
No insertions
No polymorphic variants found

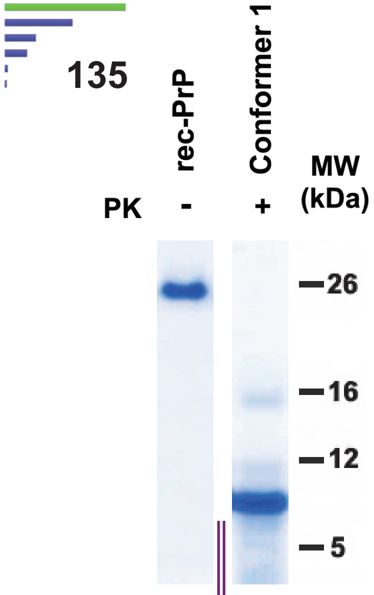

212 AA

1
Conformers

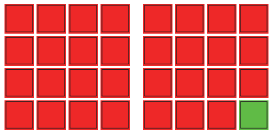

Tm^{Exp}: ND

ΔΔG: -3.92

In vitro studies

TgVole (1x) Not tested

Chinese rufous horseshoe bat Not tested

In vivo studies

TgVole (1x) Not tested

Chinese rufous horseshoe bat Not tested

pLDDT
[88.13]

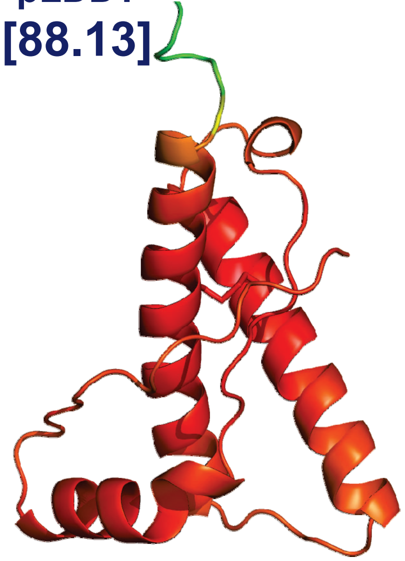

Protein code: 3151

Egyptian fruit bat

GenBank: XM_016135122

Rousettus aegyptiacus

Chiroptera 23/45
Pteropodidae 8/8

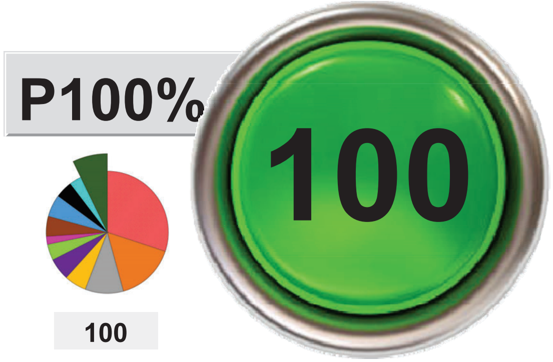

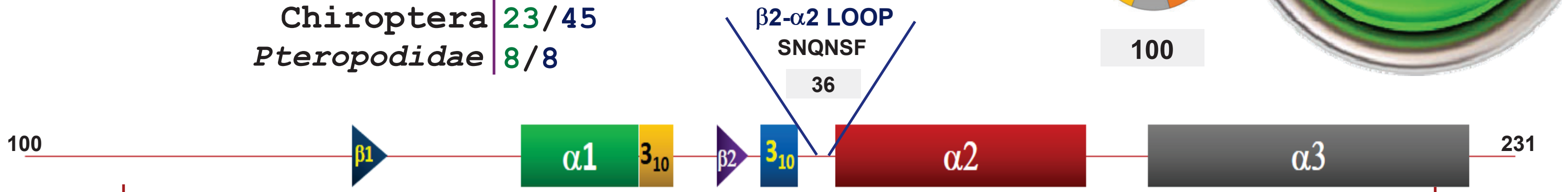


```
WNKPSKPKTNLKHVAGAAAAGAVVGGLGGYMLGSAMSRPMLHFGNDYEDRYYRENMYRYPNQVYYRPMEQYSNQNSFVHDCVNITIKQHTVTTTTKGENFTETDVKMMERVVEQMCITQYQQESRAAYHRGAS-
           I                                 I     W         N          VD  N   N                                  V    K  Q Y EGRS
```

PrP sequence differs by 11 amino acids from the bank vole PrP (see in red)

No deletions
No insertions
It shows polymorphic variants

135

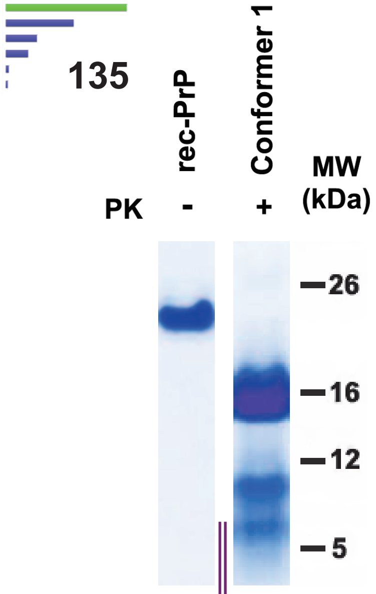

203 AA

1
Conformers

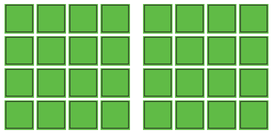

Tm^{Exp}: 67.2 ºC

ΔΔG: -20.62

In vitro studies

TgVole (1x) — Propagate

Egyptian fruit bat — Ongoing

In vivo studies

TgVole (1x) — Infectious

Egyptian fruit bat — Not tested

pLDDT [85.55]

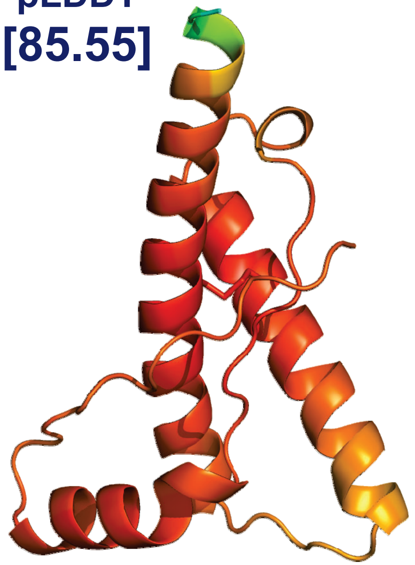

Distinct primary sequence across species

Protein code: 1107

Leschenault's rousette

GenBank: BK063997

Rousettus leschenaultii

Chiroptera 23/45
Pteropodidae 8/8

P54%

19.3

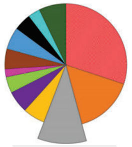

10-20

β2-α2 LOOP
NNQNSF
24

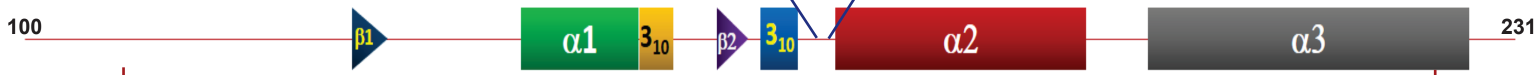

WNKPSKPKTSLKHVAGAAAAGAVVGGLGGYMLGSAMSRPMLHFGNDYEDRYYRENMYRYPNQVYYRPMEQYNNQNSFVHDCVNITVKQHTVTTTTKGENFTETDVKMMERVVEQMCITQYQQESRAAYHRGAS-
NI I W N VD N I V K Q Y EGRS

PrP sequence differs by 12 amino acids from the bank vole PrP (see in red)

No deletions
No insertions
No polymorphic variants found

Distinct primary sequence across species

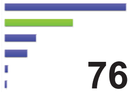

76

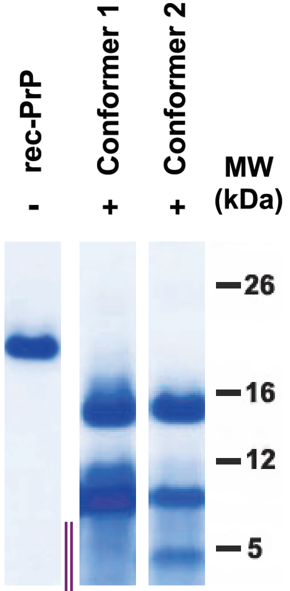

195 AA

2
Conformers

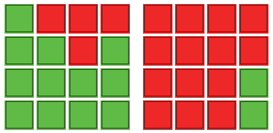

Tm[Exp]: ND

ΔΔG: -20.82

In vitro studies

Not tested
TgVole (1x)

Not tested
Leschenault's rousette

In vivo studies

Not tested
TgVole (1x)

Not tested
Leschenault's rousette

pLDDT
[86.87]

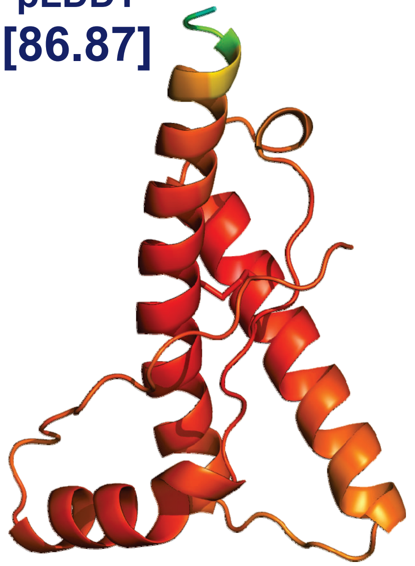

Protein code: 3157

Madagascan rousette

GenBank: BK064805

Rousettus madagascariensis

Distinct primary sequence across species

Chiroptera 23/45
Pteropodidae 8/8

P71%

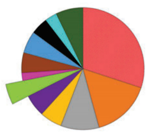

40-50

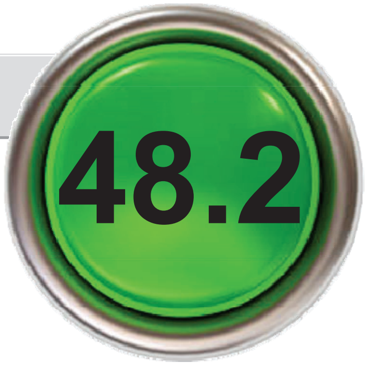

β2-α2 LOOP
NNQNSF
24

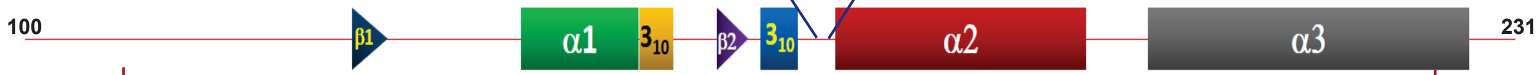

WNKPSKPKTNLKHVAGAAAAGAVVGGLGGYMLGSAMSRPMLHFGNDYEDRYYRENMYRYPNQVYYRPMEQYNNQNSFVHDCVNITVKQHTVTTTTKGENFTETDVKMMERVVEQMCITQYQQESRAAYHRGAS-

I I W N VD N I V K Q Y EGRS

PrP sequence differs by 11 amino acids from the bank vole PrP (see in red)

No deletions
No insertions
No polymorphic variants found

135

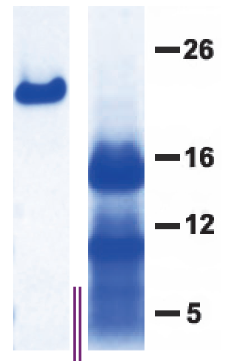

203 AA

1
Conformers

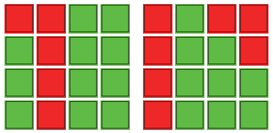

Tm^{Exp}: ND

ΔΔG: -18.77

In vitro studies

TgVole (1x) — Not tested

Madagascan rousette — Not tested

In vivo studies

TgVole (1x) — Not tested

Madagascan rousette — Not tested

pLDDT
[85.48]

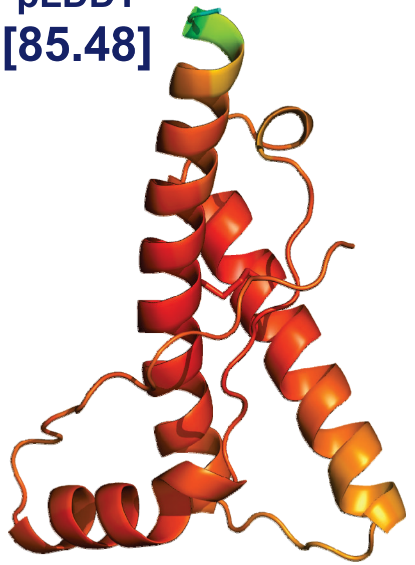

Protein code: 3973

Honduran yellow-shouldered bat

GenBank: BK063970

Sturnira ludovici hondurensis

Chiroptera 23/45
Phyllostomidae 2/10

P29%

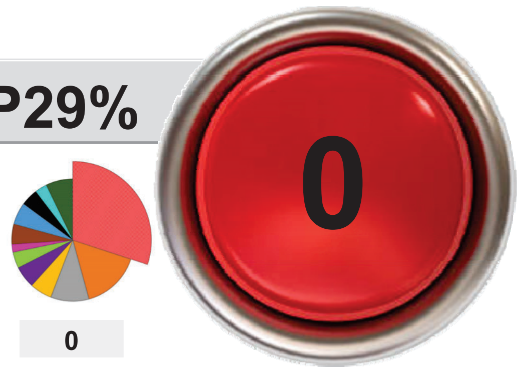

β2-α2 LOOP
SNQNNF
273

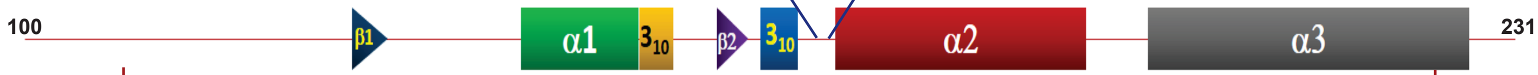

GGKPSKPKTNMKHVAGAAAAGAVVGGLGGYMLGSAMSRPLVHFGNDYEDRYYRENMNRFPNQVYYRPVDQYSNQNNFVRDCVNITVKEHTVTTTTKGENFTSTDVKIMERVVEEMCTIQYQKEYQAYQRGAS-

WN I MI W Y N H I Q E M Q VT S YE R S

PrP sequence differs by 15 amino acids from the bank vole PrP (see in red)

No deletions
No insertions
No polymorphic variants found

Distinct primary sequence across species

Tm^{Exp}: ND
ΔΔG: ND

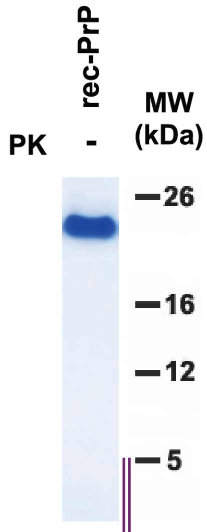

216 AA

NO MISFOLDING

In vitro studies

NOT APPLICABLE

In vivo studies

NOT APPLICABLE

pLDDT [84.95]

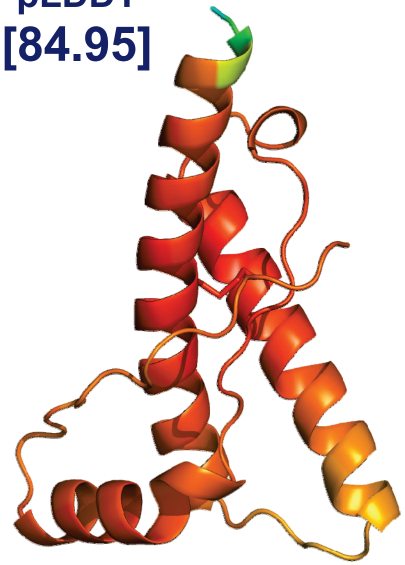

0
Conformers

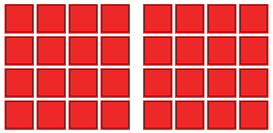

Protein code: 3126

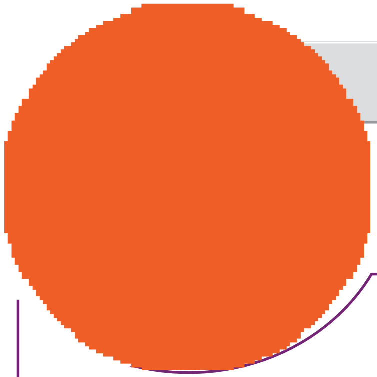

Mexican free-tailed bat

GenBank: BK064001

Tadarida brasiliensis

Chiroptera 23/45
Molossidae 2/2

P41%

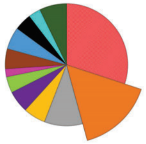

0-10

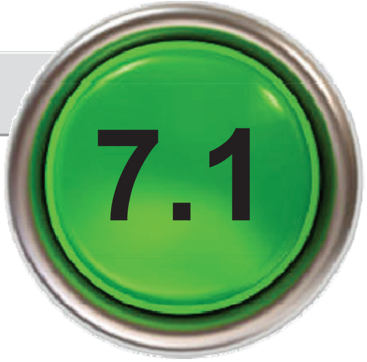

β2-α2 LOOP
SNQNNF
273

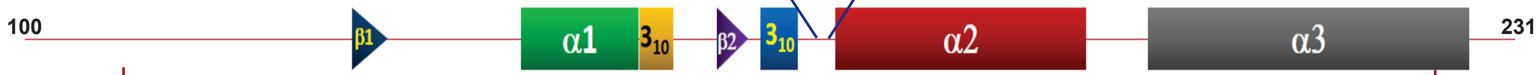

WNKPSKPKTNMKHVAGAAAAGAVVGGLGGYMLGSAMSRPPIHFGSDYEDRYYRENMNRFPNQVYYKPVDQYSNQNNFVHDCVNITVKQHTITTTTKGDNFTETDIKIMERVVEEMCTTQYQKEYQAYQRGAS-
I M N W Y R N I V E V M Q V S YE R S

PrP sequence differs by 15 amino acids from the bank vole PrP (see in red)

No deletions
No insertions
No polymorphic variants found

Distinct primary sequence across species

135

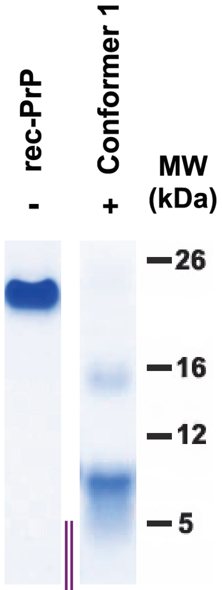

224 AA

1
Conformers

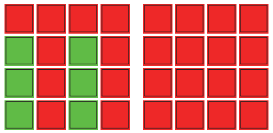

Tm^{Exp}: ND

ΔΔG: ND

In vitro studies

TgVole (1x) Not tested

Mexican free-tailed bat Not tested

In vivo studies

TgVole (1x) Not tested

Mexican free-tailed bat Not tested

pLDDT
[86.35]

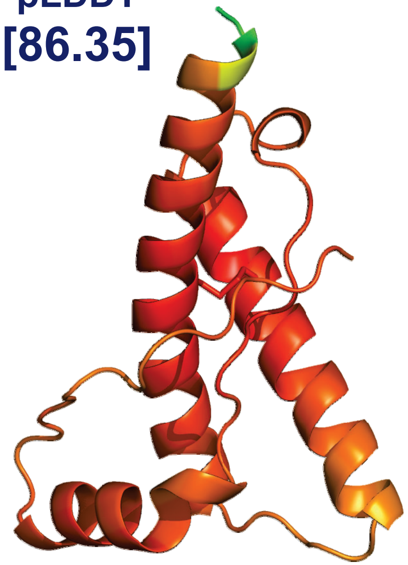

Protein code: 3162

Stripe-headed round-eared bat

GenBank: BK064003

Tonatia saurophila

Chiroptera 23/45

Phyllostomidae 2/10

P29%

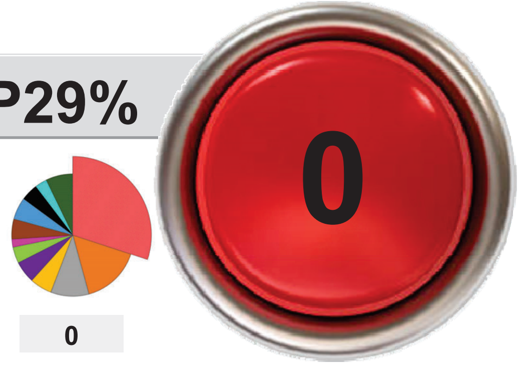

1 species sharing the same primary sequence

Greater spear-nosed bat
Phyllostomus hastatus

β2-α2 LOOP
SNQNNF
273

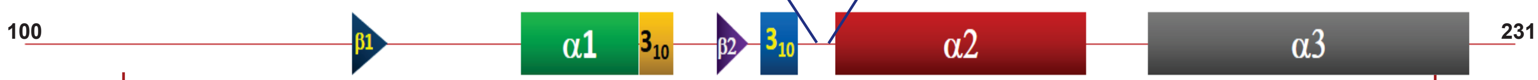

GGKPSKPKTNMKHVAGAAAAGAVVGGLGGYMLGSAMSRPLIHFGNDYEDRYYRENMNRFPNQVYYKPVDQYSNQNNFVRDCVNITVKEHTVTTTTKGENFTETDVKIMERVVEEMCTIQYQREYQAYQRGAS-

WN I M W Y R N H I Q M Q VT K S YE R S

PrP sequence differs by 15 amino acids from the bank vole PrP (see in red)

No deletions

No insertions

No polymorphic variants found

Tm^{Exp}: ND

ΔΔG: ND

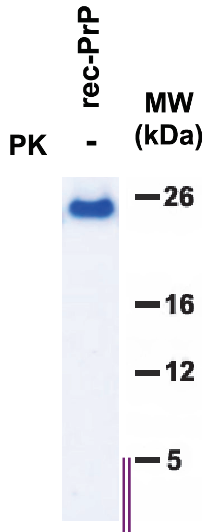

225 AA

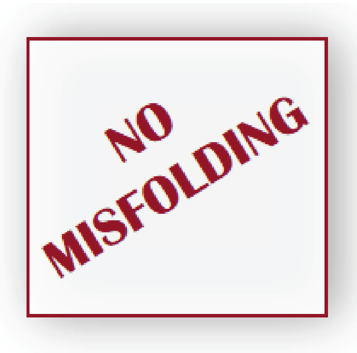

0 Conformers

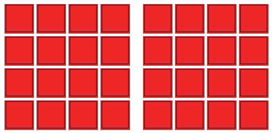

In vitro studies

NOT APPLICABLE

In vivo studies

NOT APPLICABLE

pLDDT [86.72]

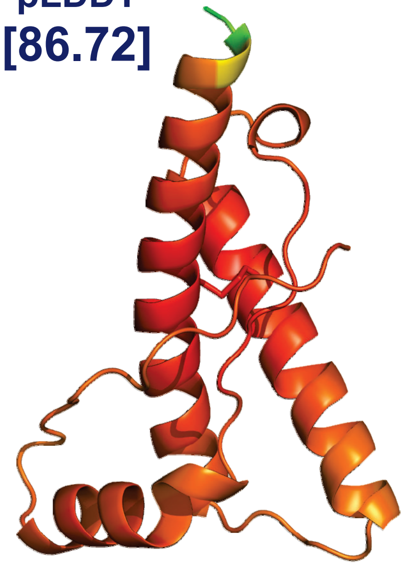

Protein code: 3164

Distinct primary sequence across species

Fringe-lipped bat

GenBank: BK064958

Trachops cirrhosus

Chiroptera 23/45
Phyllostomidae 2/10

P29%

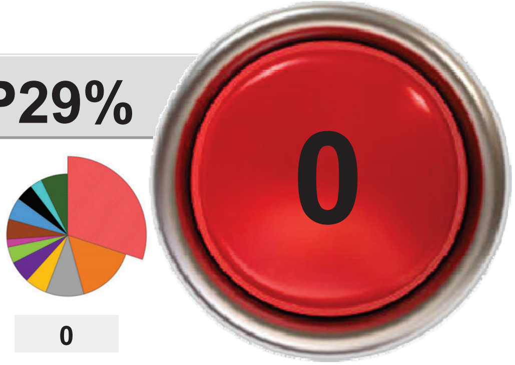

β2-α2 LOOP
SNQNNF
273

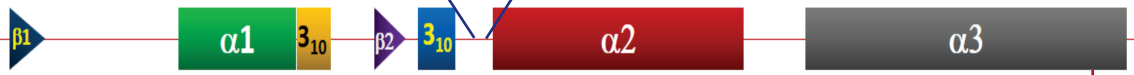

GGKPSKPKTNMKHVAGAAAAGAVVGGLGGYMLGSAMSRPLIHFGNDYEDRYYRENMNRFPNQVYYRPVDQYSNQNNFVRDCVNITVKEHTVTTTTKGENFTETDVKIMERVVEEMCTIQYQRESQAYYQRGAS-
WN I M W Y N H I Q M Q VT K EGRS

PrP sequence differs by 13 amino acids from the bank vole PrP (see in red)

No deletions
No insertions
No polymorphic variants found

Tm^{Exp}: ND

ΔΔG: -13.99

rec-PrP
PK -
MW (kDa)
26
16
12
5

226 AA

NO MISFOLDING

0
Conformers

In vitro studies

NOT APPLICABLE

In vivo studies

NOT APPLICABLE

pLDDT [86.53]

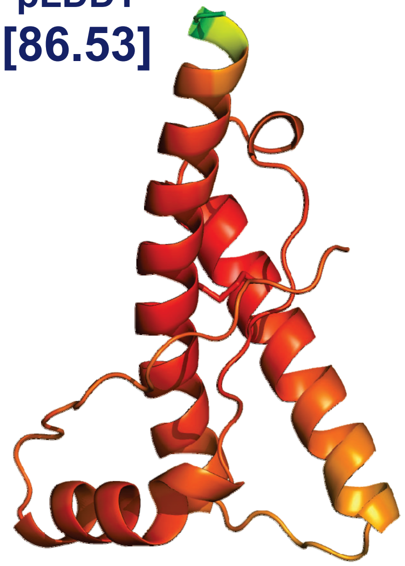

Protein code: 4512

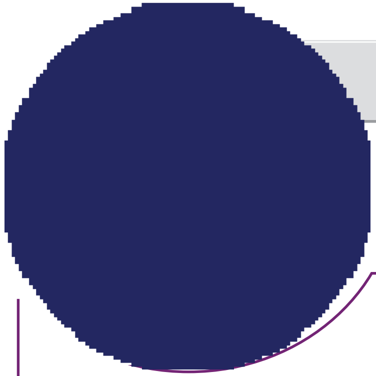

Southern naked-tailed armadillo

GenBank: BK064228

Cabassous unicinctus

Cingulata 1/4

Dasypodidae 1/4

Distinct primary sequence across species

P29%

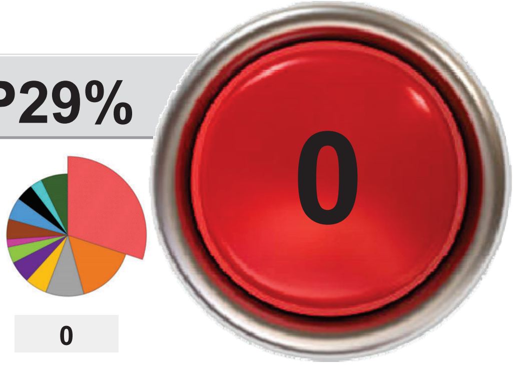

β2-α2 LOOP
GSEQSF
2

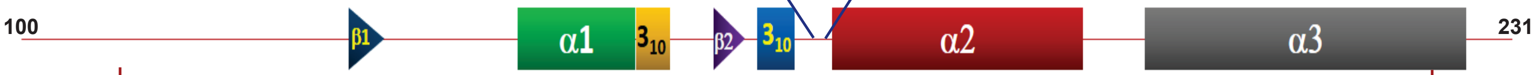

WNKPSKPKTNMKHVAGAAAAGAVVGGLGGYLVGSAMSRPLIHFSNDYDDRYYRENMYRYPNQVYYRPLEQYGSEQSFVRDCVNVTIKEHTATTTTKGENFTETDVRVMERVVEQMCVTQYQRESQAAYQRGAG-

I ML M G W N VD NNQNN H I Q V KM K Y EGRSS

PrP sequence differs by 22 amino acids from the bank vole PrP (see in red)

No deletions

No insertions

No polymorphic variants found

Tm^{Exp}: ND

ΔΔG: -14.75

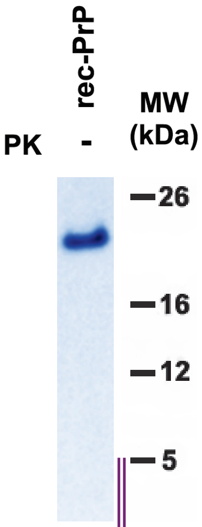

213 AA

NO MISFOLDING

0 Conformers

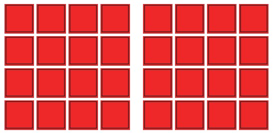

In vitro studies

NOT APPLICABLE

In vivo studies

NOT APPLICABLE

pLDDT [91.82]

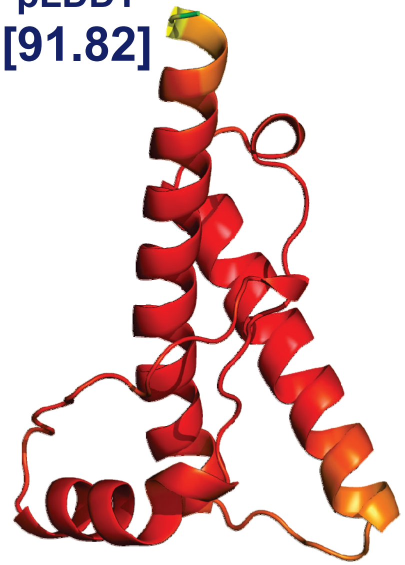

Protein code: 4842

Screaming hairy armadillo

GenBank: BK064063

Chaetophractus vellerosus

Cingulata 1/4
Dasypodidae 1/4

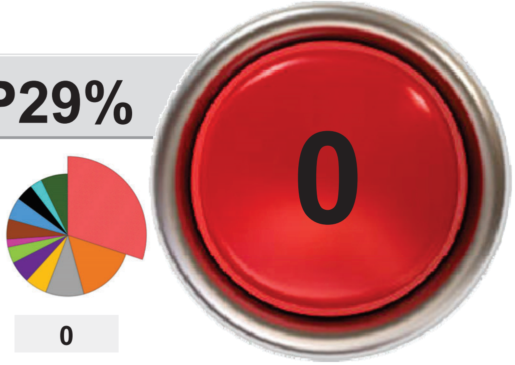

Six-banded armadillo
Euphractus sexcinctus

1 species sharing the same primary sequence

β2-α2 LOOP
SSERNF
1

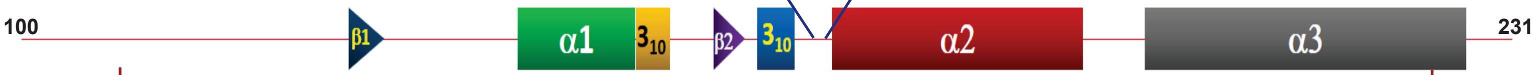

WNKPSKPKTNMKHVAGAAAAGAVVGGLGGYLVGSAMSRPLIHFGNDYEDRYYRENMYRYPNQVYYRPVEQYSSERNFVQDCVNITVKEHTVTTTTKGENFTETDVRMMERVVEQMCITQYQRESQAAFQRGSG-

I ML M W N D NNQN H I Q K V K YYEGR S

PrP sequence differs by 17 amino acids from the bank vole PrP (see in red)

No deletions
No insertions
No polymorphic variants found

Tm^{Exp}: ND

ΔΔG: -17.17

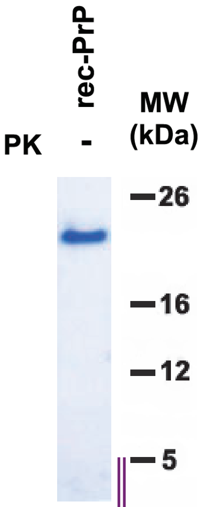

211 AA

NO MISFOLDING

0
Conformers

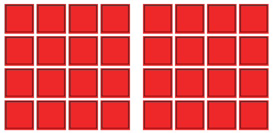

In vitro studies

NOT APPLICABLE

In vivo studies

NOT APPLICABLE

pLDDT
[86.09]

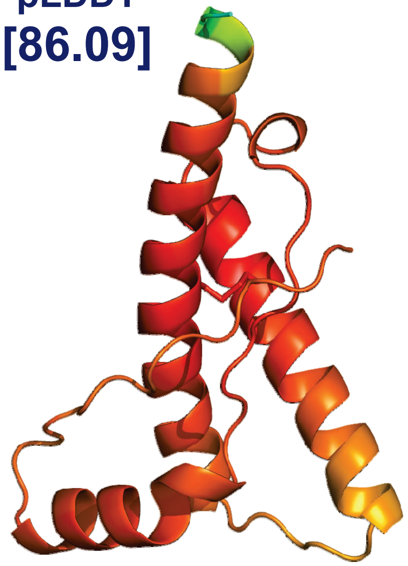

Protein code: 3238

Nine-banded armadillo

GenBank: EU559337

Dasypus novemcinctus

Cingulata 1/4

Dasypodidae 1/4

P31%

0.4

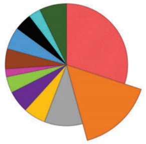

β2-α2 LOOP
SSEKNF
1

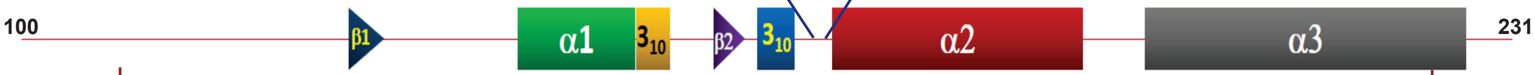

WNKPSKPKTNMKHVAGAAAAGAVVGGLGGYLVGSAMSRPLIHFGNDYEDRYYRENMYRYPNQVYYRPVEQYSSEKNFVHDCVNITIKEHTVTTTTKGENFTETDVRMMERVVEQMCITQYQREAQAAYQRGAG-

I ML M W N D NNQN Q K V K S Y EGRSS

PrP sequence differs by 16 amino acids from the bank vole PrP (see in red)

No deletions

No insertions

No polymorphic variants found

Distinct primary sequence across species

135

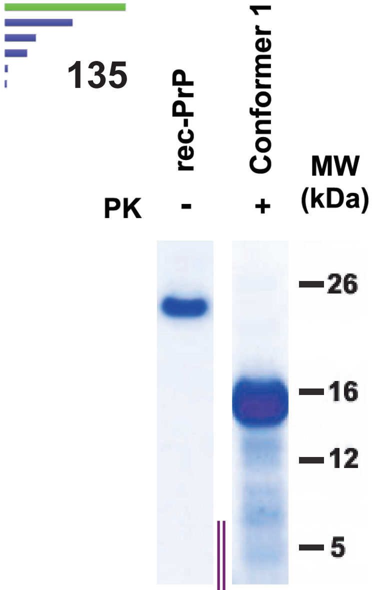

210 AA

1

Conformers

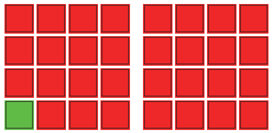

Tm^{Exp}: ND

ΔΔG: -20.08

In vitro studies

TgVole (1x) Not tested

Nine-banded armadillo Not tested

In vivo studies

TgVole (1x) Not tested

Nine-banded armadillo Not tested

pLDDT

[86.09]

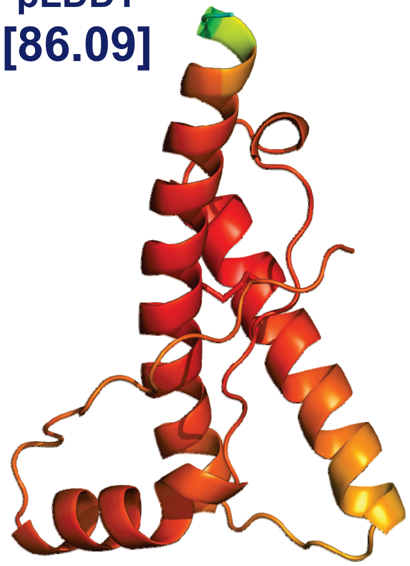

Protein code: 0968

Southern three-banded armadillo

GenBank: BK064002

Tolypeutes matacus

Cingulata 1/4

Dasypodidae 1/4

Distinct primary sequence across species

P29%

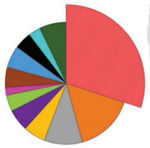

0

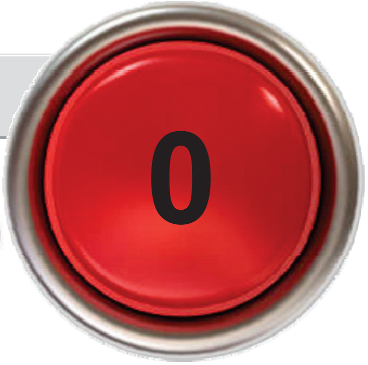

β2-α2 LOOP
GSEQSF
2

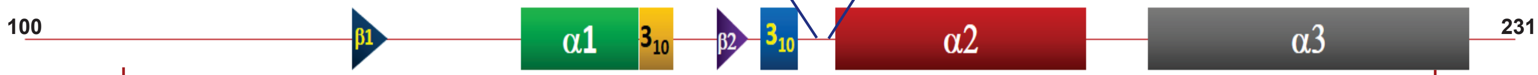


```
WNKPSKPKTNMKHVAGAAAAGAVVGGLGGYLVGSAMNRPLIHFGNDYDDRYYRENMYRYPNQVYYRPLEQYGSEQSFVRDCVNITIKEHTATTTTRGENFTETDVRVMERVVEQMCVTQYQRESQAAYQRGAG-
          I                 ML    S  M       W          N         VD  NNQNN  H         Q  V              KM                K      Y EGRSS
```

PrP sequence differs by 21 amino acids from the bank vole PrP (see in red)

No deletions

No insertions

No polymorphic variants found

Tm[Exp]: ND

ΔΔG: -8.09

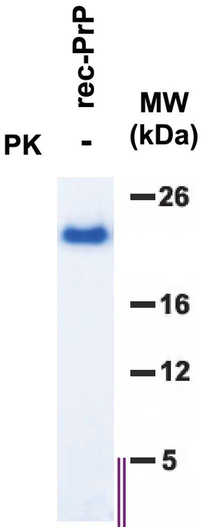

211 AA

NO MISFOLDING

0
Conformers

In vitro studies

NOT APPLICABLE

In vivo studies

NOT APPLICABLE

pLDDT
[87.05]

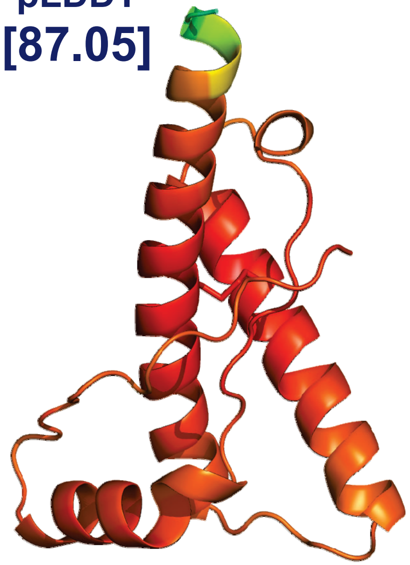

Protein code: 3163

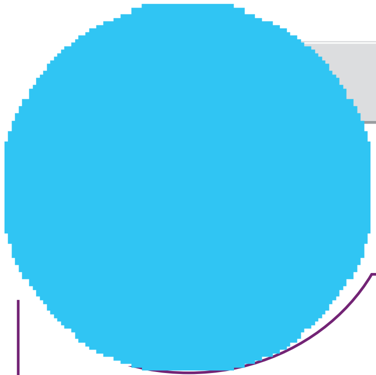

Yellow-footed antechinus

GenBank: BK063971

Antechinus flavipes

Dasyuromorphia 4/9

Dasyuridae 3/7

P29%

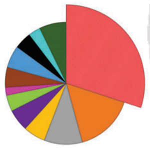

0

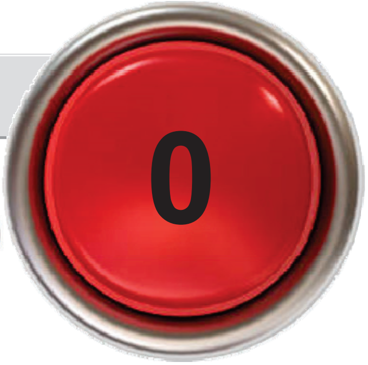

β2-α2 LOOP
NNQNNF
164

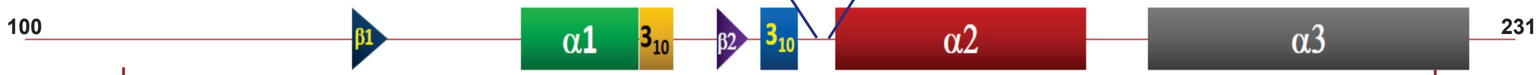


```
KWKPDKPKTNMKHVAGAAAAGAVLGGVGGYMLGSAMSRPVMHFGNEWEDRYYRENQYRYPNQVMYRPIDQYNNQNNFVHDCVNITVKQHTTTTTTKGENFTETDIKIMERVVEQMCITQYQNEYRAAQYSYNLA-
WN  S      I           V  L           MI    D        MN     Y  V               I    V             V M         V    K SQ YYEGRSS
```

PrP sequence differs by 18 amino acids from the bank vole PrP (see in red)

No deletions

No insertions

No polymorphic variants found

Distinct primary sequence across species

Tm^{Exp}: ND

ΔΔG: ND

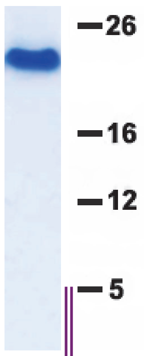

218 AA

NO MISFOLDING

0 Conformers

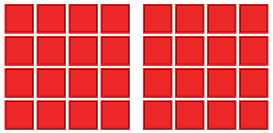

In vitro studies

NOT APPLICABLE

In vivo studies

NOT APPLICABLE

pLDDT [92.27]

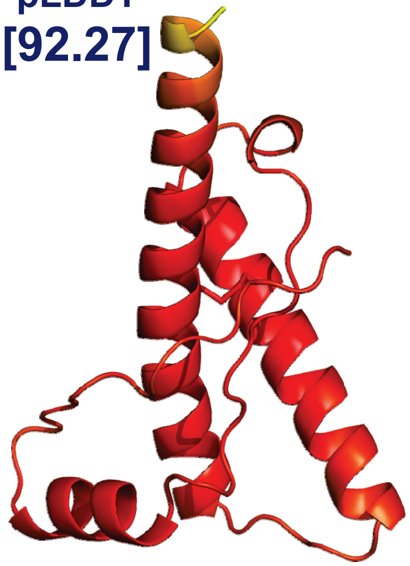

Protein code: 3128

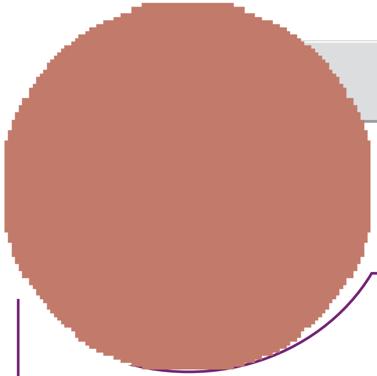

Brown antechinus

GenBank: BK063930

Antechinus stuartii

Dasyuromorphia 4/9

Dasyuridae 3/7

P29%

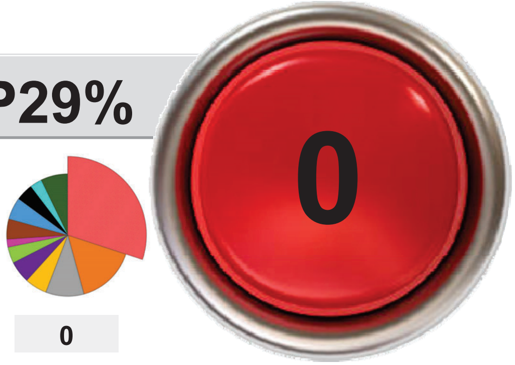

β2-α2 LOOP
NNQNNF
164

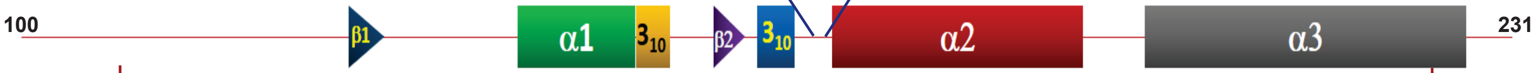


```
KWKPDKPKTNMKHVAGAAAAGAVLGGVGGYMLGSAMSRPVMHFGSDWEDRYYRENQYRYPNQVMYRPIDQYNNQNNFVHDCVNITVKQHTTTTTTKGENFTETDIRIMERVVEQMCITQYQNEYRAAQYSYNLA-
WN  S    I           V  L             MI   N              MN       Y   V                    I    V               VKM        V    K SQ YYEGRSS
```

PrP sequence differs by 19 amino acids from the bank vole PrP (see in red)

No deletions

No insertions

No polymorphic variants found

Distinct primary sequence across species

Tm^{Exp}: ND

ΔΔG: ND

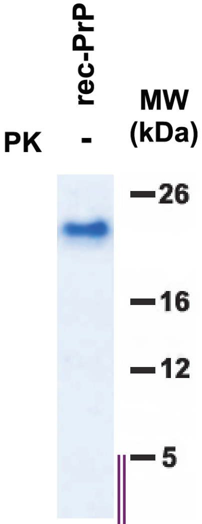

218 AA

NO MISFOLDING

0 Conformers

In vitro studies

NOT APPLICABLE

In vivo studies

NOT APPLICABLE

pLDDT [92.82]

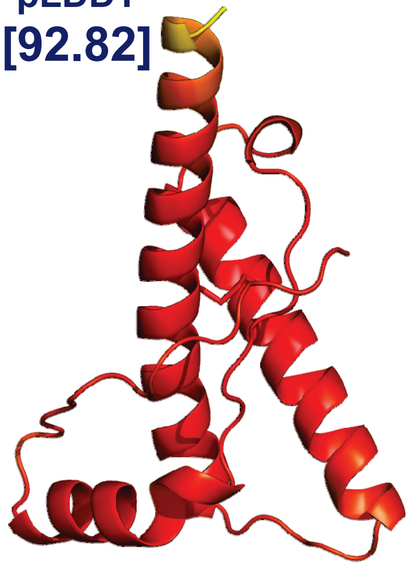

Protein code: 3048

Northern quoll

GenBank: BK064760

Dasyurus hallucatus

Dasyuromorphia 4/9

Dasyuridae 3/7

P45%

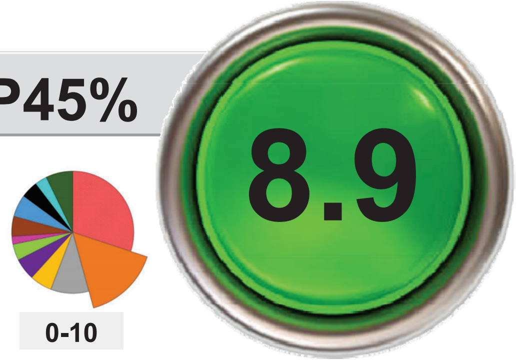

β2-α2 LOOP
SNQNNF
273

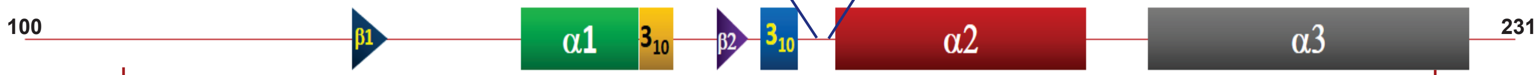

KWKPDKPKTNMKQMAGAAAAGAVLGGVSGYVLGSAMSRPIMHFGNDYEDRYYRENQYRYPNQVMYRPIDQYSNQNNFVHDCVNITVKQHTTTTTTKGENFTETDIKIMERVVEQMCITQYQNEYRAAQYSYNMA-

WN S I HV V LG M MI W MN Y V N I V V M V K SQ YYEGRSS

PrP sequence differs by 23 amino acids from the bank vole PrP (see in red)

No deletions

No insertions

No polymorphic variants found

Distinct primary sequence across species

Tm^{Exp}: ND

ΔΔG: ND

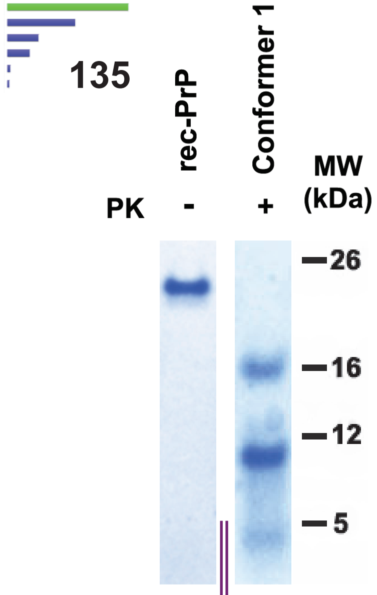

228 AA

1

Conformers

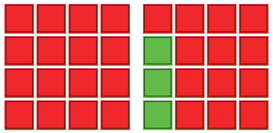

In vitro studies

TgVole (1x) Not tested

Northern quoll Not tested

In vivo studies

TgVole (1x) Not tested

Northern quoll Not tested

pLDDT

[91.53]

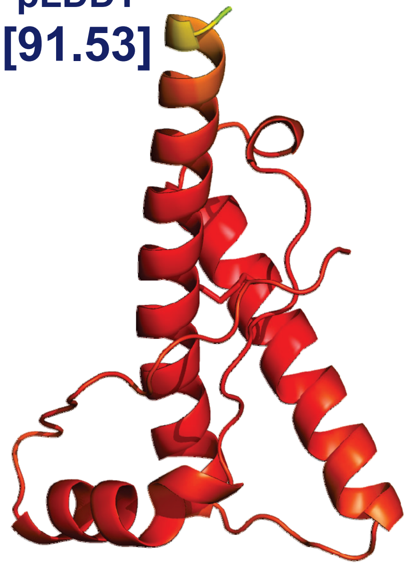

Protein code: 3849

Eastern quoll

GenBank: BK064810

Dasyurus viverrinus

Dasyuromorphia 4/9

Dasyuridae 3/7

P35%

1.4

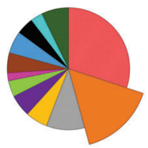

0-10

β2-α2 LOOP

SNQNNF

273

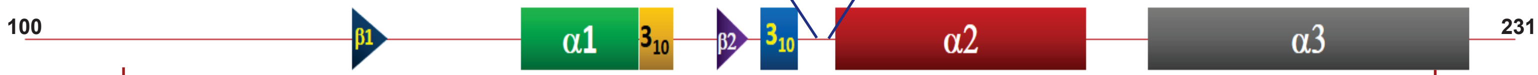

KWKPDKPKTNMKQMAGAAAAGAVLGGVGGYVLGSAMSRPVMHFGNDYEDRYYRENQYRYPNQVMYRPIDQYSNQNNFVHDCVNITVKQHTTTTTTKGENFTETDIKIMERVVEQMCITQYQNEYRAAQYSYNMA-

WN S I HV V L M MI W MN Y V N I V V M V K SQ YYEGRSS

PrP sequence differs by 22 amino acids from the bank vole PrP (see in red)

No deletions

No insertions

No polymorphic variants found

Distinct primary sequence across species

135

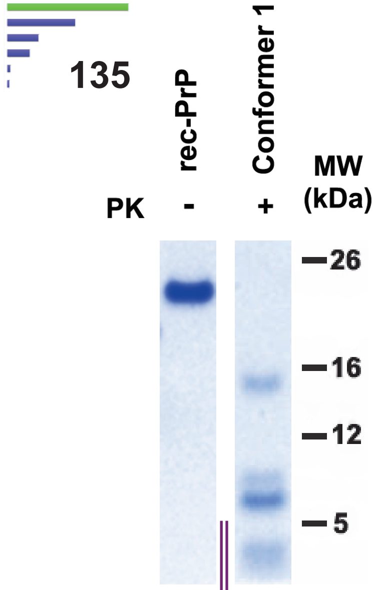

218 AA

1

Conformers

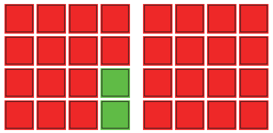

Tm[Exp]: ND

ΔΔG: ND

In vitro studies

TgVole (1x) — Not tested

Eastern quoll — Not tested

In vivo studies

TgVole (1x) — Not tested

Eastern quoll — Not tested

pLDDT

[91.97]

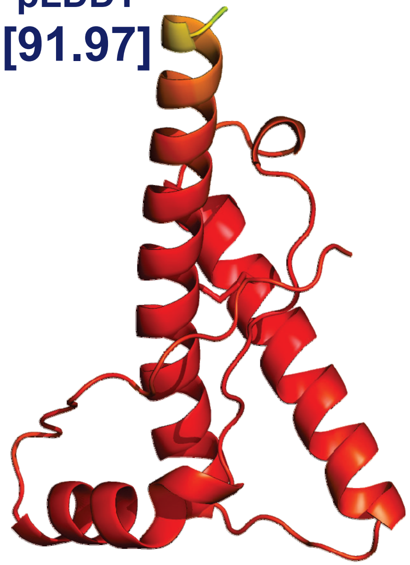

Protein code: 4008

Numbat

GenBank: BK064811

Myrmecobius fasciatus

Dasyuromorphia 4/9
Myrmecobiidae 0/1

P29%

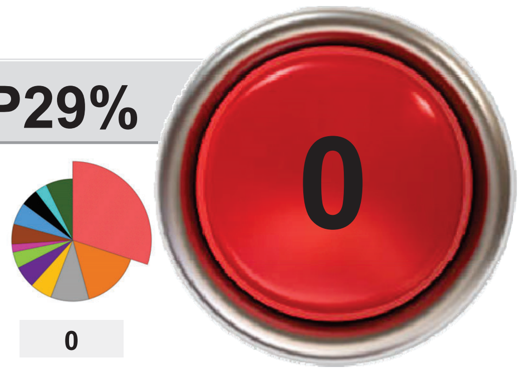

β2-α2 LOOP
SNQNNF
273

100 — β1 — α1 — 3_{10} — β2 — 3_{10} — α2 — α3 — 231

```
KWKPDKPKTNMKHVAGAAAAGAVVGGLGGYVLGSAMSRPIMHFGNEYEDRYYRENQYRYPNQVMYRPIDQYSNQNNFVHDCVNITVKQHTTTTTTKGENFTETDIKIMERVVEQMCITQYQNEYRAAQYSYNMA-
WN  S    I                    M         MI     DW          MN       Y   V   N               I    V              V M          V    K SQ YYEGRSS
```

PrP sequence differs by 19 amino acids from the bank vole PrP (see in red)

No deletions
No insertions
No polymorphic variants found

Tm^{Exp}: ND
ΔΔG: ND

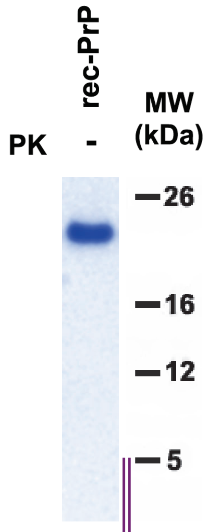

213 AA

NO MISFOLDING

0
Conformers

In vitro studies

NOT APPLICABLE

In vivo studies

NOT APPLICABLE

pLDDT [92.19]

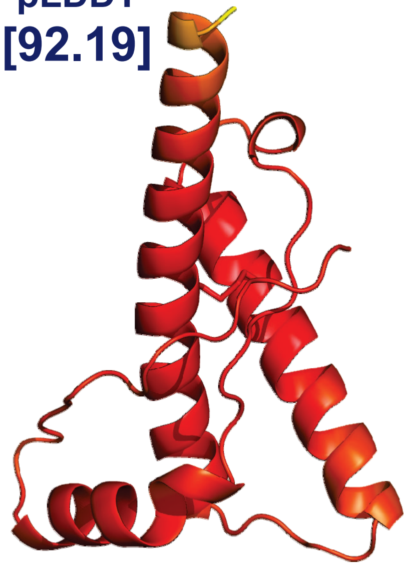

Distinct primary sequence across species

Protein code: 4079

Brush-tailed phascogale

GenBank: BK064816

Phascogale tapoatafa

Dasyuromorphia 4/9

Dasyuridae 3/7

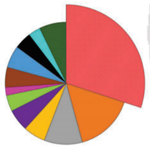

P29%

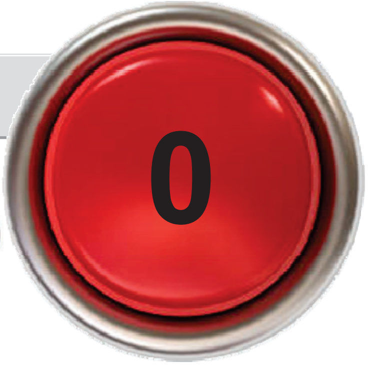

0

1 species sharing the same primary sequence

Red-tailed phascogale
Phascogale calura

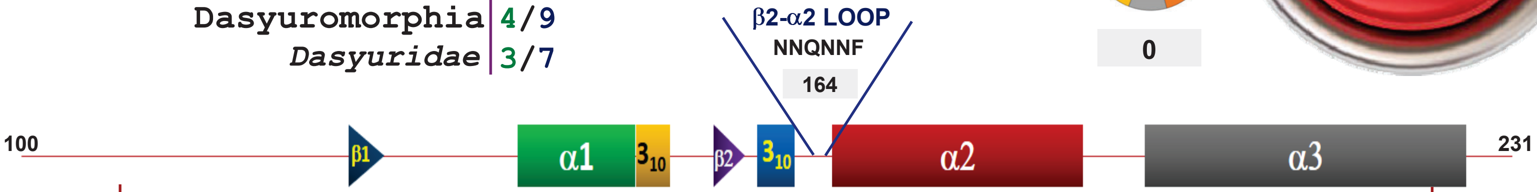


```
KWKPDKPKTNMKQMAGAAAAGAVLGGVGGYMLGSAMSRPVMHFGNEYEDRYYRENQYRYPNQVMYRPIDQYNNQNNFVHDCVNITVKQHTTTTTTKGENFTETDIKIMERVVEQMCITQYQNEYRAAQYSYNLA-
WN  S    I HV        V  L             MI     DW        MN      Y   V                I    V                 V M         V     K SQ YYEGRSS
```

PrP sequence differs by 21 amino acids from the bank vole PrP (see in red)

No deletions

No insertions

No polymorphic variants found

Tm^{Exp}: ND

ΔΔG: ND

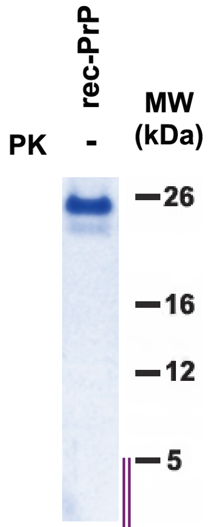

228 AA

NO MISFOLDING

0 Conformers

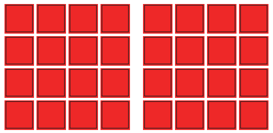

In vitro studies

NOT APPLICABLE

In vivo studies

NOT APPLICABLE

pLDDT [92.71]

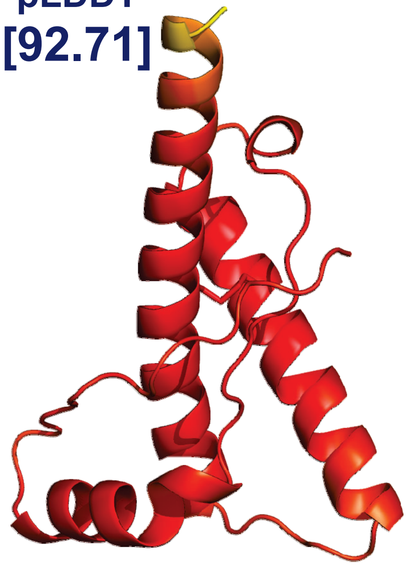

Protein code: 4470

Tasmanian devil

GenBank: XM_003757949

Sarcophilus harrisii

Dasyuromorphia 4/9

Dasyuridae 3/7

P29%

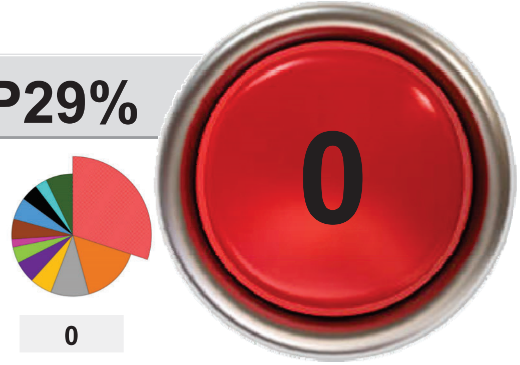

0

β2-α2 LOOP
SSQNNF
42

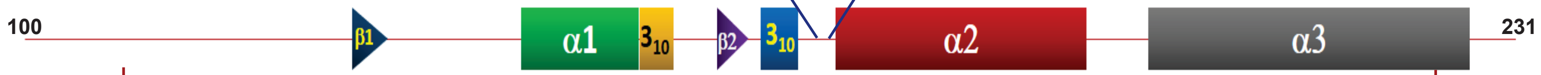

KWKPDKPKTNMKHMAGAAAAGAVLGGVGGYVLGSAMSRPIMHFGNDYEDRYYRENQYRYPNQVMYRPIDQYSSQNNFVHDCVNITVKQHTTTTTTKGENFTETDIKIMERVVEQMCITQYQNEYRAAQYSYNMA-

WN S I V V L M MI W MN Y V NN I V V M V K SQ YYEGRSS

PrP sequence differs by 22 amino acids from the bank vole PrP (see in red)

No deletions

No insertions

No polymorphic variants found

Distinct primary sequence across species

Tm^{Exp}: ND

ΔΔG: ND

PK

rec-PrP

-

MW (kDa)

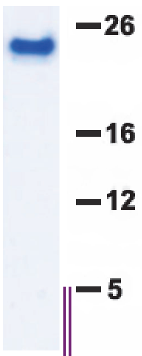

218 AA

NO MISFOLDING

0 Conformers

In vitro studies

NOT APPLICABLE

In vivo studies

NOT APPLICABLE

pLDDT [90.1]

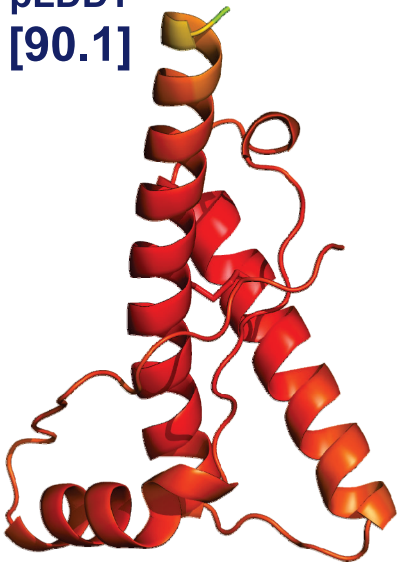

Protein code: 0970

Fat-tailed dunnart

GenBank: BK064171

P55%

19.6

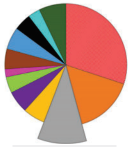

10-20

Sminthopsis crassicaudata

Dasyuromorphia 4/9
Dasyuridae 3/7

Distinct primary sequence across species

β2-α2 LOOP
NNQNNF
164

100

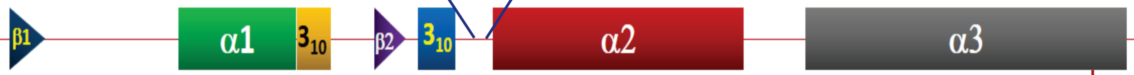

231

```
KWKPDKTKTNMKQMAGAAAVGAVVGGVGGYMLGSAMSRPVMHFGNDYEDRYYRENQYRYPNQVMYRPIDQYNNQNNFVHDCVNITVKQHTTTTTSKGENFTETDIKIMERVVEQMCITQYQNEYRAAQYSYNMA-
WN  S P  I HV     A     L          MI    W      MN     Y  V              I   V  T          V M          V   K SQ YYEGRSS
```

PrP sequence differs by 21 amino acids from the bank vole PrP (see in red)

No deletions
No insertions
No polymorphic variants found

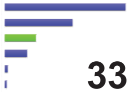

33

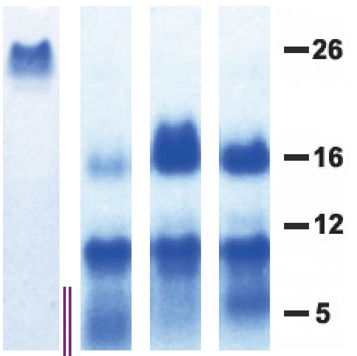

228 AA

3 Conformers

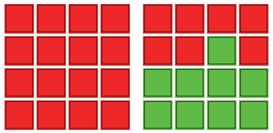

Tm^{Exp}: ND

ΔΔG: ND

In vitro studies

TgVole (1x) Not tested

Fat-tailed dunnart Not tested

In vivo studies

TgVole (1x) Not tested

Fat-tailed dunnart Not tested

pLDDT [92.93]

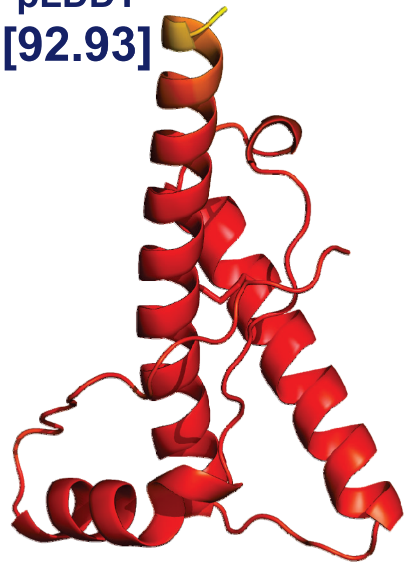

Protein code: 3850

Thylacine

GenBank: BK064189

P39%

3.6

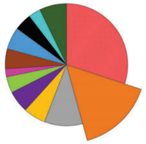

0-10

Thylacinus cynocephalus

Dasyuromorphia 4/9

Thylacinidae 1/1

β2-α2 LOOP
SNQNNF
273

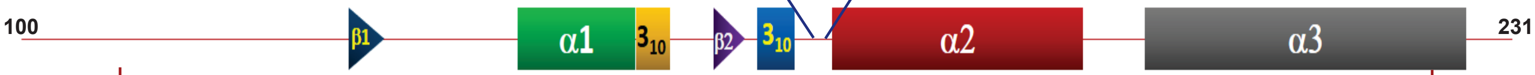

KWKPDKPKTNMKHVAGAAAAGAVVGGLGGYMLGSAMSRPIMHFGNEYEDRYYRENQYRYPNQVMYRPIDQYSNQNNFVHDCVNITVKQHTTTTTTKGENFTETDIKIMERVVEQMCITQYQTEYRAAQYTYNMA-

WN S I MI DW MN Y V N I V V M V K SQ YYEGRSS

PrP sequence differs by 18 amino acids from the bank vole PrP (see in red)

No deletions

No insertions

No polymorphic variants found

135

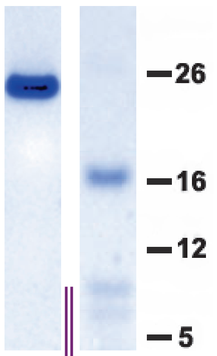

208 AA

1

Conformers

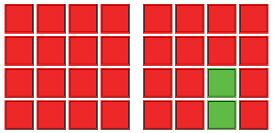

Tm^{Exp}: ND

ΔΔG: ND

In vitro studies

TgVole (1x) Ongoing

Thylacine Not tested

In vivo studies

TgVole (1x) Ongoing

Thylacine Not tested

pLDDT [91.45]

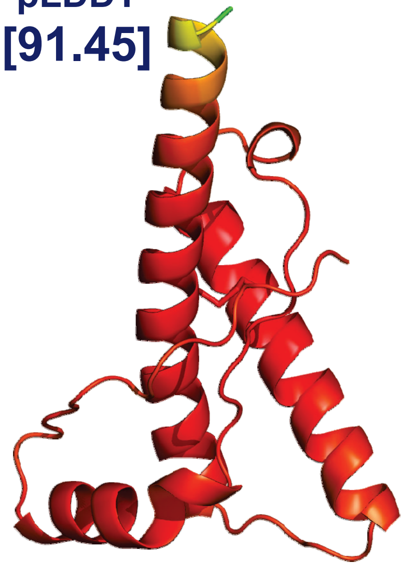

Distinct primary sequence across species

Protein code: 4339

Sunda flying lemur

GenBank: AY133034

Galeopterus variegatus

Dermoptera 1/1

Cynocephalidae 1/1

Philippine flying lemur
Cynocephalus volans

1 species sharing the same primary sequence

P35%

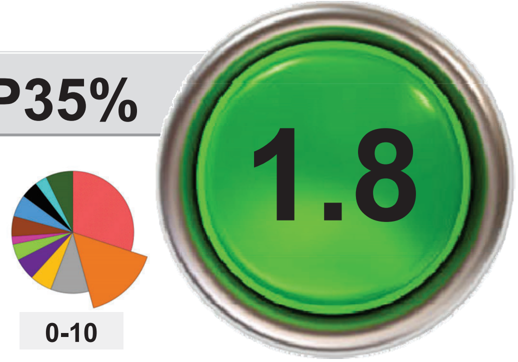

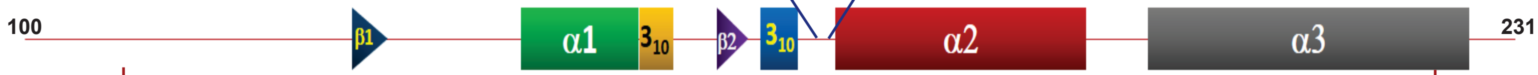

WGKPSKPKTNLKQMAGAAAAGAVVGGLGGYMLGSAMSRPLIHFGNDYEDRYYRENMYRYPNQVYYRPVDQYSNQNNFVHDCVNITVKQHTVTTTTKGENFTETDIKIMERVVEQMCITQYQKEQQAYYQRGSS-

N I HV M W N N I V M V S EGR

PrP sequence differs by 12 amino acids from the bank vole PrP (see in red)

No deletions

No insertions

No polymorphic variants found

135

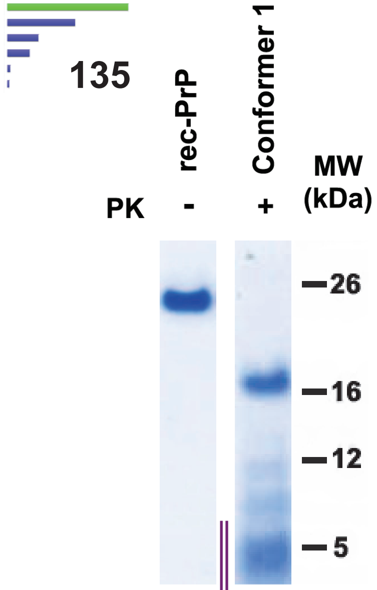

212 AA

1 Conformers

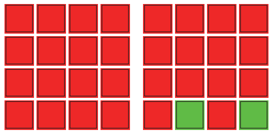

Tm^{Exp}: ND

ΔΔG: -8.07

In vitro studies

No propagation
TgVole (1x)

Not tested
Sunda flying lemur

In vivo studies

Ongoing
TgVole (1x)

Not tested
Sunda flying lemur

pLDDT
[85.82]

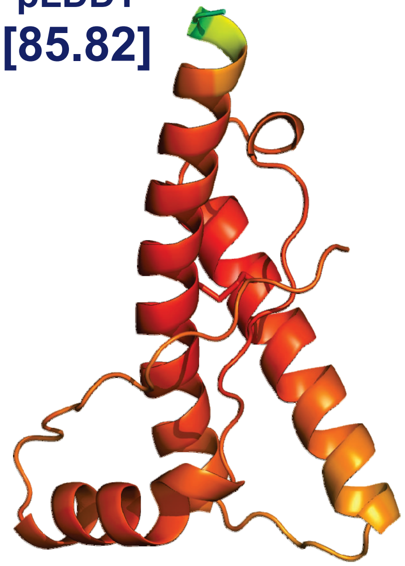

Protein code: 0957

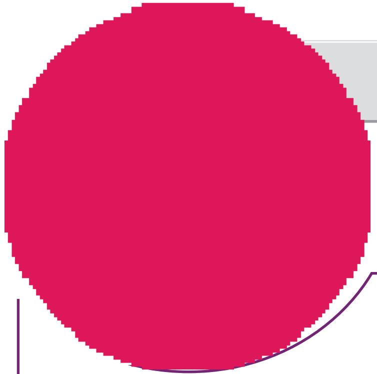

Virginia opossum

GenBank: BK064768

Didelphis virginiana

Didelphimorphia 2/3

Didelphidae 2/3

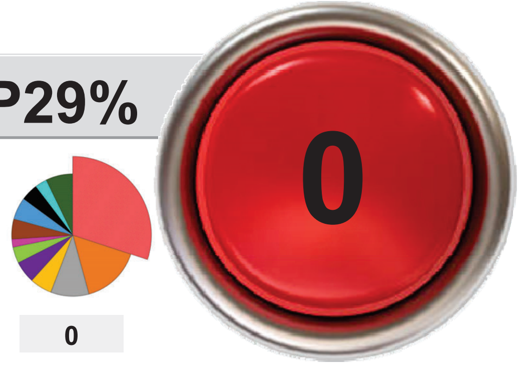

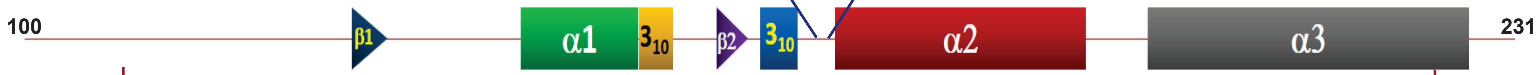


```
KWKPDKPKTNMKHVAGAAAAGAVVGGLGGYMLGSAMSRPIMHFGNDYEDRYYRENQYRYPNQVMYRPIDQYSNQNNFVHDCVNITVKQHTTTTTTKGENFTETDIKIMERVVEQMCITQYQNEYRSAYN-
WN  S    I                           MI     W        MN     Y   V   N              I    V                V M         V    K SQAY EG
```

PrP sequence differs by 18 amino acids from the bank vole PrP (see in red)

No deletions

No insertions

No polymorphic variants found

Distinct primary sequence across species

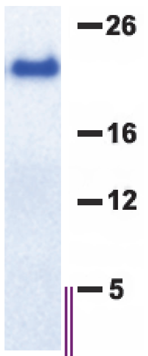

215 AA

NO MISFOLDING

0 Conformers

Tm^{Exp}: ND

ΔΔG: ND

In vitro studies

NOT APPLICABLE

In vivo studies

NOT APPLICABLE

pLDDT [90.58]

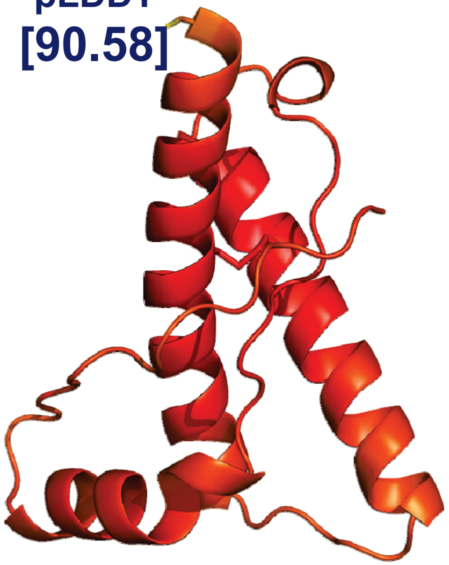

Protein code: 3918

Agile gracile opossum

GenBank: BK063958

P57%

22.9

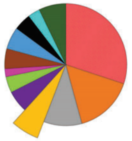

20-30

Gracilinanus agilis

Didelphimorphia 2/3

Didelphidae 2/3

β2-α2 LOOP
NNQNNF
164

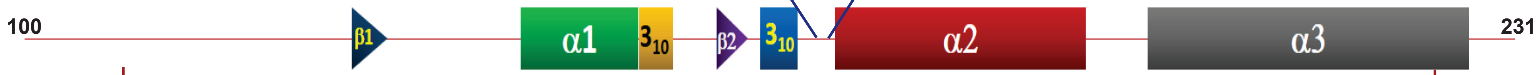

KWKPDKPKTNMKHVAGAAAAGAVVGGLGGYMLGSAMSRPIMHFGNDYEDRYYRENQYRYPNQVMYRPIEQYNNQNNFVHDCVNITVKQHTTTTTTKGENFTETDIKIMERVVEQMCITQYQNEQRAAYS-

WN S I MI W MN Y VD I V V M V K SQ Y EG

PrP sequence differs by 17 amino acids from the bank vole PrP (see in red)

No deletions

No insertions

No polymorphic variants found

Distinct primary sequence across species

33

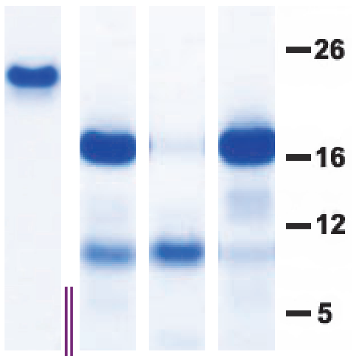

214 AA

3 Conformers

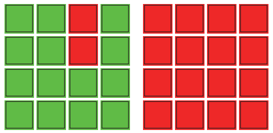

Tm^{Exp}: ND

ΔΔG: ND

In vitro studies

TgVole (1x) Ongoing

Agile gracile opossum Not tested

In vivo studies

TgVole (1x) Ongoing

Agile gracile opossum Not tested

pLDDT [87.25]

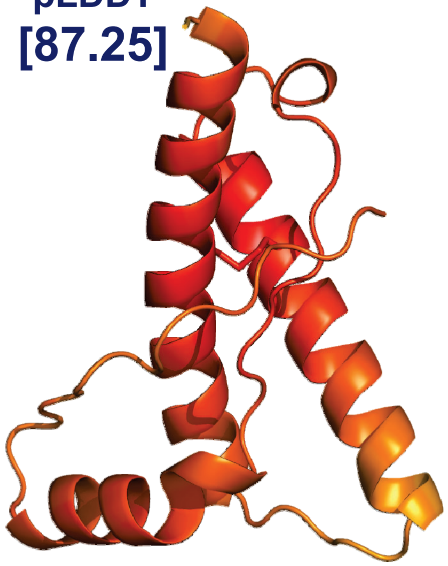

Protein code: 3086

Opossum

GenBank: NM_001040028

Monodelphis domestica

Didelphimorphia 2/3

Didelphidae 2/3

P39%

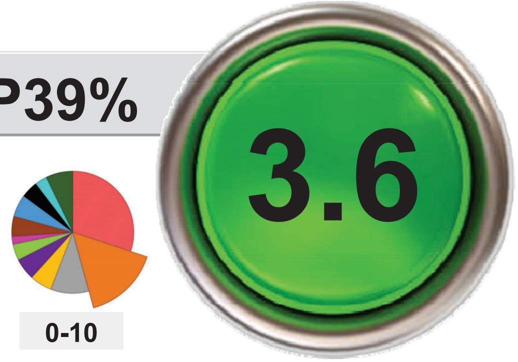

Distinct primary sequence across species

β2-α2 LOOP
NNQNNF
164

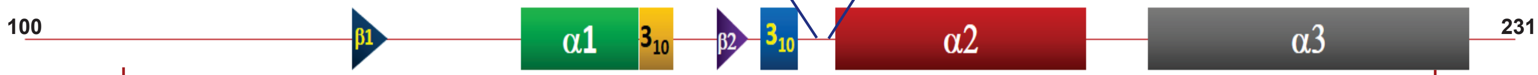

KWKPDKPKTNMKHVAGAAAAGAVVGGLGGYMLGSAMSRPIMHFGNDYEDRYYRENQYRYPNQVMYRPIDQYNNQNNFVHDCVNITVKQHTTTTTTKGENFTETDIKIMERVVEQMCITQYQNEYRSAYS-

WN S I MI W MN Y V I V V M V K SQAY EG

PrP sequence differs by 17 amino acids from the bank vole PrP (see in red)

No deletions

No insertions

It shows polymorphic variants

135

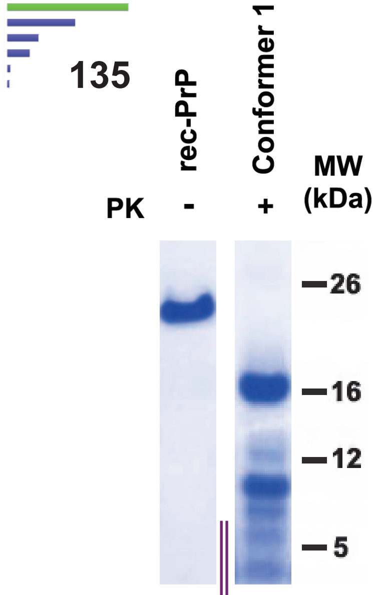

215 AA

1
Conformers

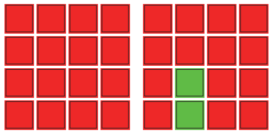

Tm[Exp]: ND

ΔΔG: ND

In vitro studies

No propagation
TgVole (1x)

Not tested
Opossum

In vivo studies

Non infectious
TgVole (1x)

Not tested
Opossum

pLDDT
[93.24]

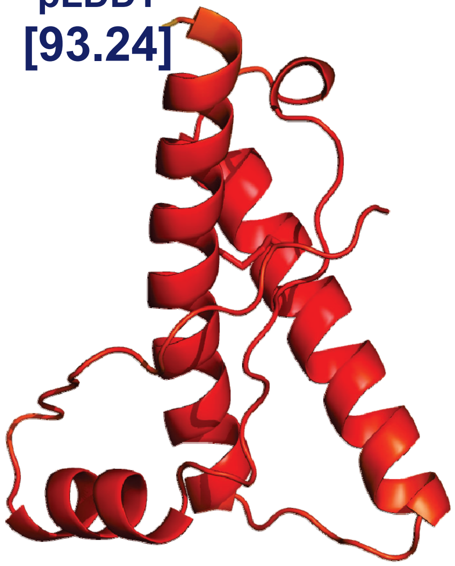

Protein code: 0398

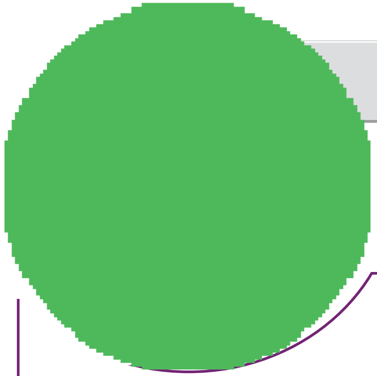

Woylie

GenBank: **BK064808**

Bettongia penicillata ogilbyi

Diprotodontia 9/18

Potoroidae 1/2

P45%

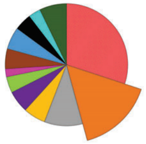

9.8

Distinct primary sequence across species

β2-α2 LOOP
NGQNSF
1

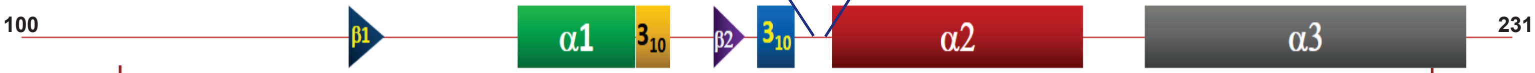

KWKPDKPKTNLKHVAGAAAAGAVVGGLGGYMLGSAMSRPVIHFGNEYEDRYYRENQYRYPNQVMYRPIDQYNGQNSFVHDCVNITVKQHTTTTTTKGENFTETDIKIMERVVEQMCITQYQNEYQAAQRYYNM-

WN S I M DW MN Y V N N I V V M V K S YYEGRSS

PrP sequence differs by 17 amino acids from the bank vole PrP (see in red)

No deletions

No insertions

No polymorphic variants found

135

216 AA

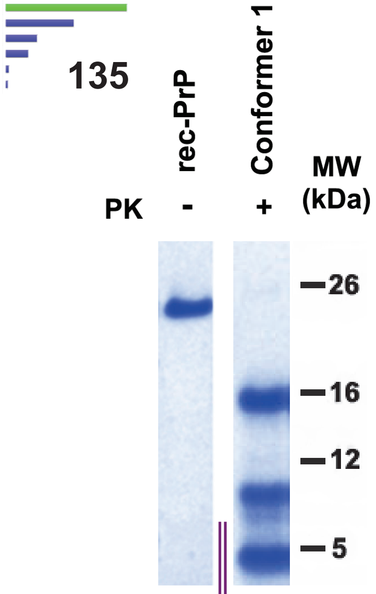

1 Conformers

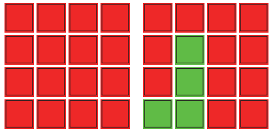

Tm[Exp]: ND

ΔΔG: ND

In vitro studies

TgVole (1x)	Not tested
Woylie	Not tested

In vivo studies

TgVole (1x)	Not tested
Woylie	Not tested

pLDDT [92.82]

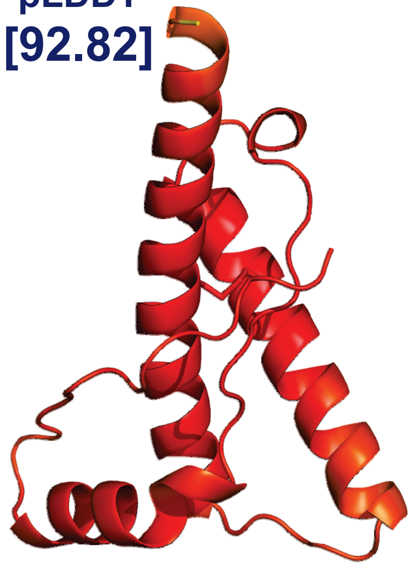

Protein code: 3976

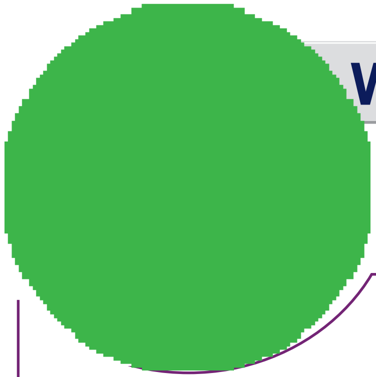

Western pygmy possum

GenBank: BK064813

Cercartetus concinnus

Diprotodontia 9/18

Burramyidae 1/1

P46%

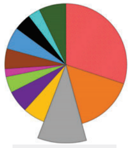

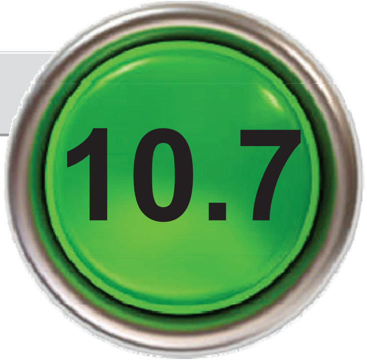

β2-α2 LOOP
NNQNNF
164

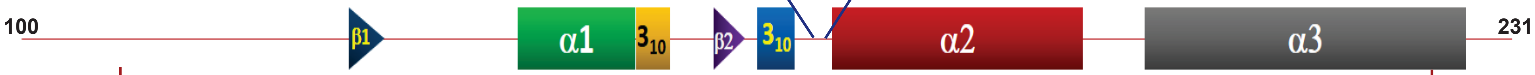

KWKPDKPKTNLKHVAGAAAAGAVVGGLGGYMLGNAMSRPPIHFGNEYEDRYYRENQNRYPNQVMYRPIDQYNNQNNFVHDCVNITVKQHTTTTTTKGENFTETDIKIMERVVEQMCITQYQAEYQAAQRGYN-

WN S I S M DW M Y V I V V M V K S YYE RSS

PrP sequence differs by 15 amino acids from the bank vole PrP (see in red)

No deletions
No insertions
No polymorphic variants found

Distinct primary sequence across species

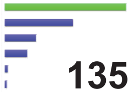

135

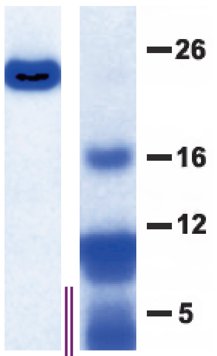

212 AA

1
Conformers

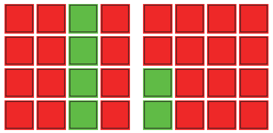

Tm^{Exp}: ND

ΔΔG: ND

In vitro studies

TgVole (1x) Propagate

Western pygmy possum Not tested

In vivo studies

TgVole (1x) Infectious

Western pygmy possum Not tested

pLDDT
[86.86]

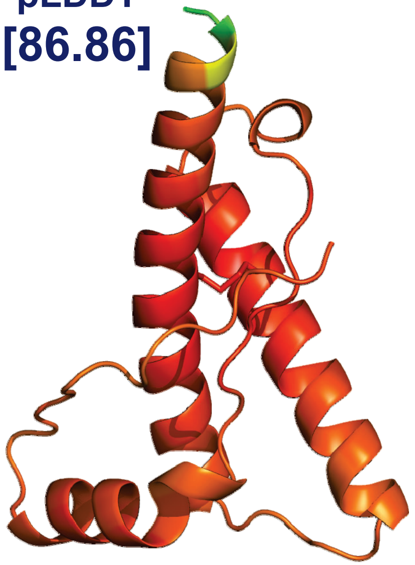

Protein code: 4104

Matschie's tree-kangaroo

GenBank: BK064823

Dendrolagus matschiei

Diprotodontia 9/18
Macropodidae 1/7

P29%

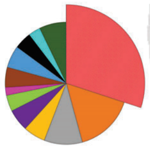

0

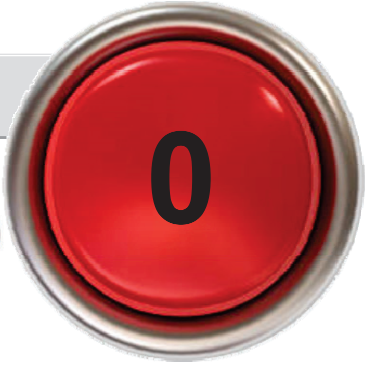

β2-α2 LOOP
SGQSSF
4

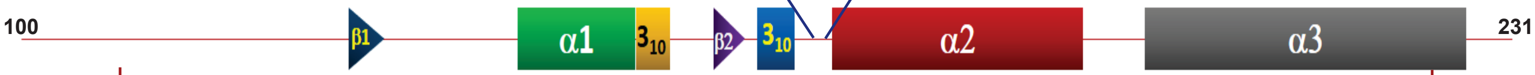

KWKPDKPKTNLKHVAGAAAAGAVVGGLGGYMLGSAMSRPVIHFGNEYEDRYYRENQYRYPNQVMYRPIDQYSGQSSFVHDCVNITVKQHTTTTTTKGENFTETDIKIMERVVEQMCITQYQNEYQAAQRYYNM-
WN S I M DW MN Y V NN NN I V V M V K S YYEGRSS

PrP sequence differs by 19 amino acids from the bank vole PrP (see in red)

No deletions
No insertions
No polymorphic variants found

Distinct primary sequence across species

Tm^{Exp}: ND

ΔΔG: -23.84

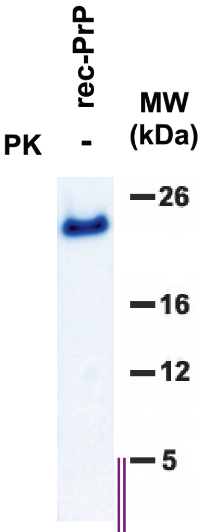

216 AA

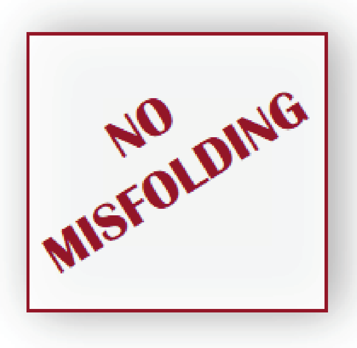

0
Conformers

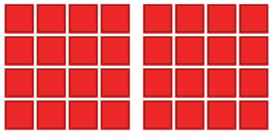

In vitro studies

NOT APPLICABLE

In vivo studies

NOT APPLICABLE

pLDDT
[94.09]

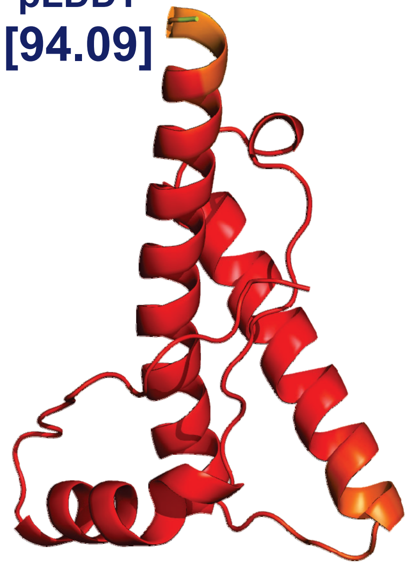

Protein code: 4627

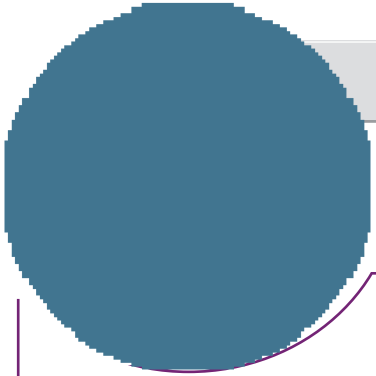

Leadbeater's possum

GenBank: BK064074

P74%

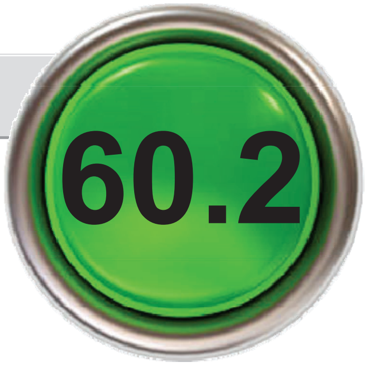

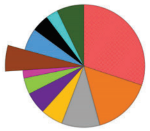

60-70

Gymnobelideus leadbeateri

Diprotodontia 9/18
Petauridae 1/2

β2-α2 LOOP
SSQSNF
1

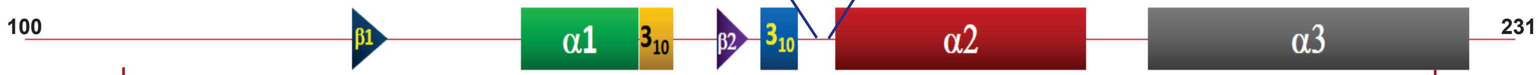

KWKPDKPKTNLKHVAGAAAAGAVVGGLGGYMLGSAMSRPVMHFGNEYEDRYYRENQYRYPNQVMYRPIDQYSSQSNFVHDCVNITVKQHTTTTTTKGENFTETDIKIMERVVEQMCITQYQNEYQAAAQRGYN-
WN S I MI DW MN Y V NN N I V V M V K S YYEGRSS

PrP sequence differs by 19 amino acids from the bank vole PrP (see in red)

No deletions
No insertions
No polymorphic variants found

Distinct primary sequence across species

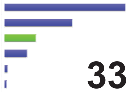

33

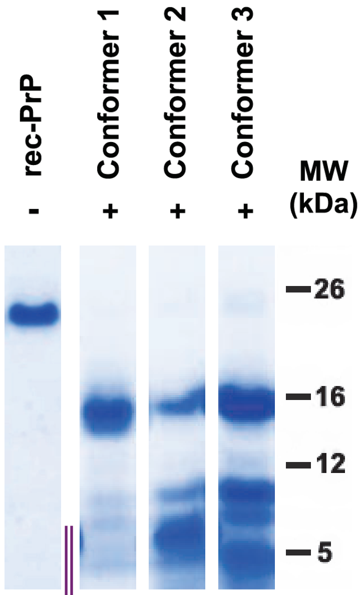

215 AA

3 Conformers

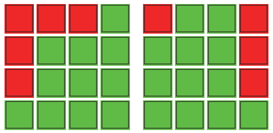

Tm[Exp]: ND
ΔΔG: ND

In vitro studies

TgVole (1x) Not tested
Leadbeater's possum Not tested

In vivo studies

TgVole (1x) Not tested
Leadbeater's possum Not tested

pLDDT
[89.44]

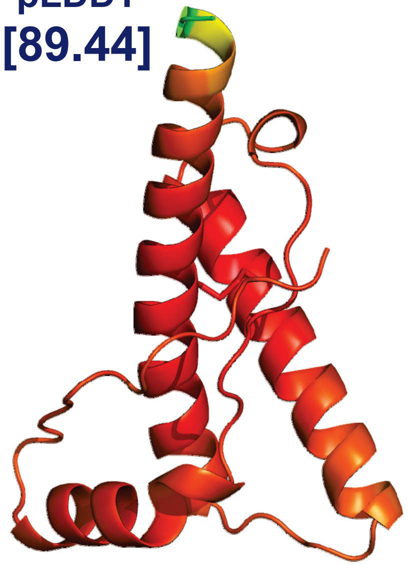

Protein code: 3252

Rufous hare-wallaby

GenBank: BK064777

P48%

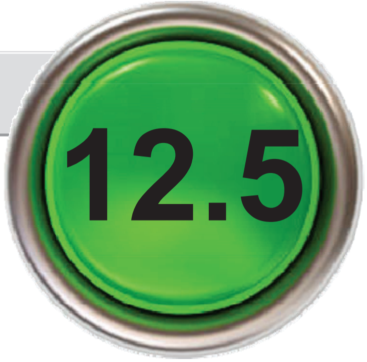

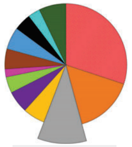

10-20

Lagorchestes hirsutus

Diprotodontia 9/18
Macropodidae 1/7

β2-α2 LOOP
SGQNSF
3

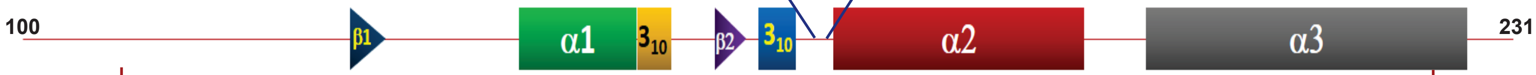

KWKPDKPKTNLKHVAGAAAAGAVVGGLGGYMLGSAMSRPVMHFGNEYEDRYYRENQYRYPNQVMYRPIDQYSGQNSFVHDCVNITVKQHTTTTTTKGENFTETDIKIMERVVEQMCITQYQNEYQAAQRYYNM-
WN S I MI DW MN Y V NN N I V V M V K S YYEGRSS

PrP sequence differs by 19 amino acids from the bank vole PrP (see in red)

No deletions
No insertions
No polymorphic variants found

Distinct primary sequence across species

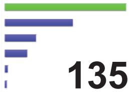

135

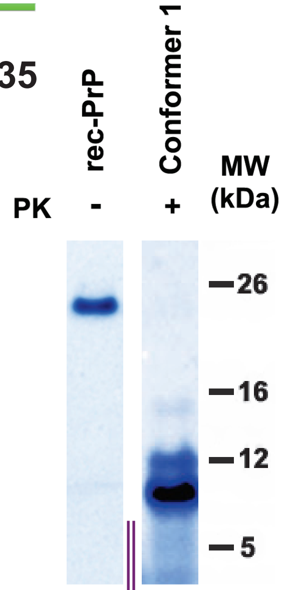

216 AA

1
Conformers

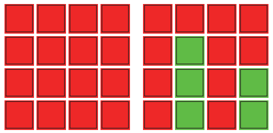

Tm^{Exp}: ND

ΔΔG: ND

In vitro studies

Not tested
TgVole (1x)

Not tested
Rufous hare-wallaby

In vivo studies

Not tested
TgVole (1x)

Not tested
Rufous hare-wallaby

pLDDT
[92.38]

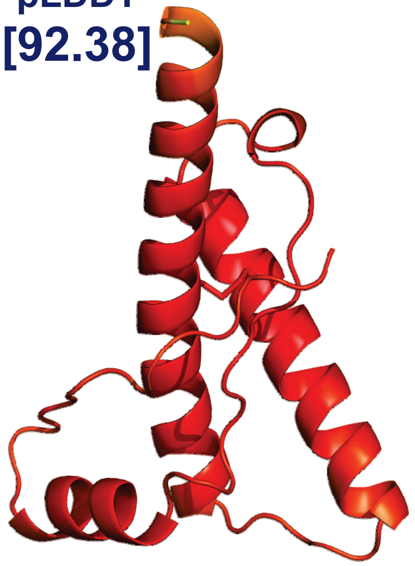

Protein code: 3934

Wallaby

GenBank: AY659988

Macropus eugenii

Diprotodontia	9/18
Macropodidae	1/7

2 species sharing the same primary sequence

Western Brush Wallaby
Macropus irma

Red-necked wallaby
Macropus rufogriseus

P29%

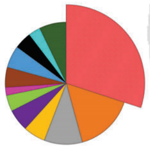

0

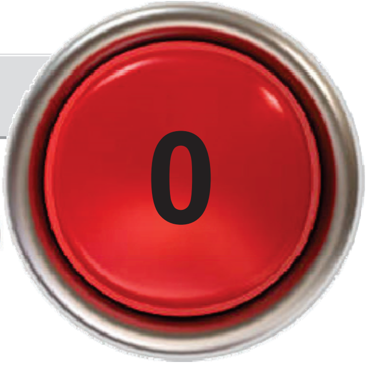

β2-α2 LOOP
GSQNSF
2

100

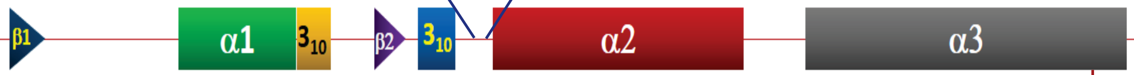

231

```
KWKPDKPKTNLKHVAGAAAAGAVVGGLGGYMLGSAMSRPVMHFGNEYEDRYYRENQYRYPNQVMYRPIDQYGSQNSFVHDCVNITVKQHTTTTTTKGENFTETDIKIMERVVEQMCITQYQNEYQAAQRYYNM-
WN  S     I                               MI    DW         MN     Y   V   NN  N         I    V                   V M         V    K S YYEGRSS
```

PrP sequence differs by 19 amino acids from the bank vole PrP (see in red)

No deletions

No insertions

No polymorphic variants found

Tm^{Exp}: ND

$\Delta\Delta G$: ND

PK | rec-PrP -

MW (kDa)

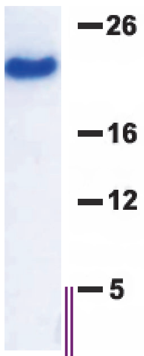

216 AA

NO MISFOLDING

0 Conformers

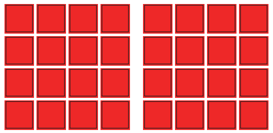

In vitro studies

NOT APPLICABLE

In vivo studies

NOT APPLICABLE

pLDDT [92.2]

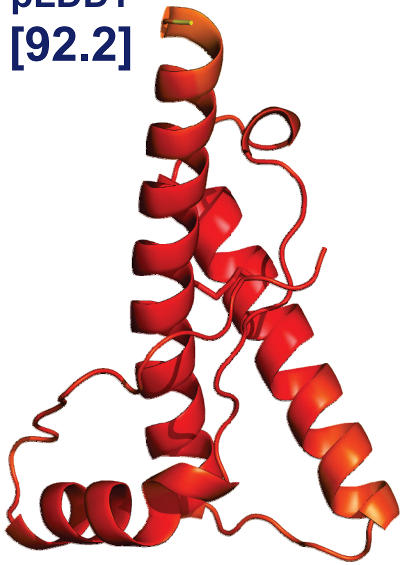

Protein code: 3333

Eastern grey kangaroo

GenBank: BK064781

Macropus giganteus

Diprotodontia 9/18
Macropodidae 1/7

P29%

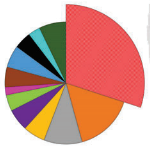

0

0

1 species sharing the same primary sequence

Western grey kangaroo
Macropus fuliginosus

β2-α2 LOOP
SGQSGF
1

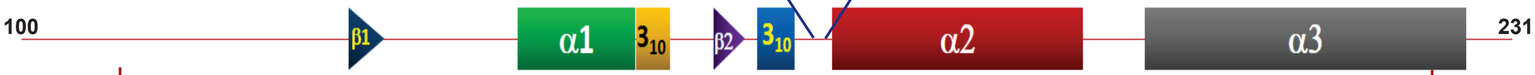

KWKPDKPKTNLKHVAGAAAAGAVVGGLGGYMLGSAMSRPVMHFGNEYEDRYYRENQYRYPNQVMYRPIDQYSGQSGFVHDCVNITVKQHTTTTTTKGENFTETDIKIMERVVEQMCITQYQNEYQAAQRYYNM-
WN S I MI DW MN Y V NN NN I V V M V K S YYEGRSS

PrP sequence differs by 20 amino acids from the bank vole PrP (see in red)

No deletions
No insertions
No polymorphic variants found

Tm^{Exp}: ND
ΔΔG: ND

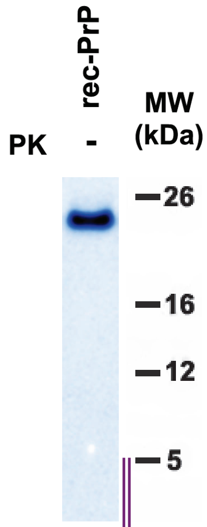

216 AA

NO MISFOLDING

0
Conformers

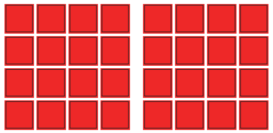

In vitro studies

NOT APPLICABLE

In vivo studies

NOT APPLICABLE

pLDDT
[92.16]

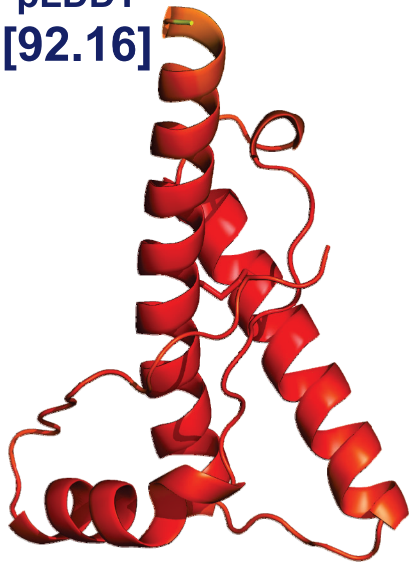

Red kangaroo

GenBank: OR47271

Macropus rufus

Diprotodontia 9/18
Macropodidae 1/7

P29%

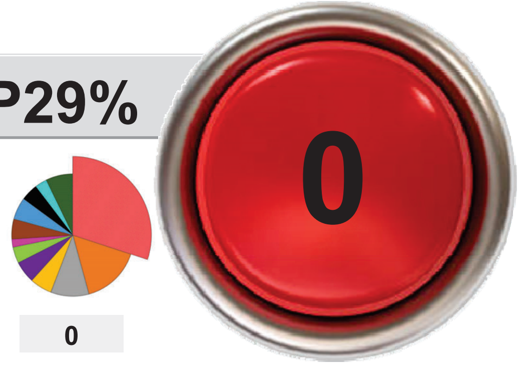

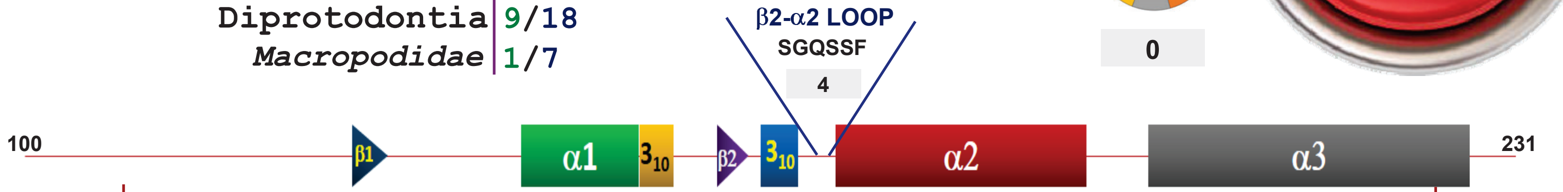

KWKPDKPKTNLKHVAGAAAAGAVVGGLGGYVLGSAMSRPVMHFGNEYEDRYYRENQYRYPNQVMYRPIDQYSGQSSFVHDCVNITVKEHTTTTTTKGENFTETDIKIMERVVEQMCITQYQNEYQAAQRYYNM-
WN S I M MI DW MN Y V NN NN I Q V V M V K S YYEGRSS

PrP sequence differs by 22 amino acids from the bank vole PrP (see in red)

No deletions
No insertions
No polymorphic variants found

Distinct primary sequence across species

Tm^{Exp}: ND

ΔΔG: ND

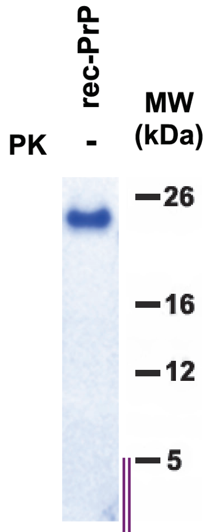

216 AA

NO MISFOLDING

0
Conformers

In vitro studies

NOT APPLICABLE

In vivo studies

NOT APPLICABLE

pLDDT
[91.7]

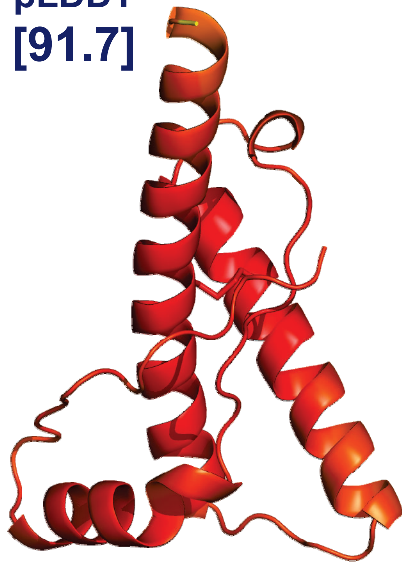

Protein code: 3775

Sugar glider

GenBank: BK064219

Petaurus breviceps

Diprotodontia 9/18
Petauridae 1/2

P39%

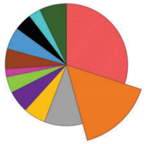

0-10

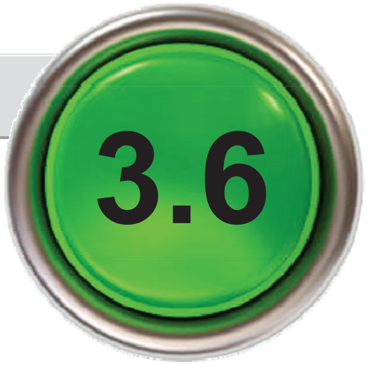

β2-α2 LOOP
SNQNNF
273

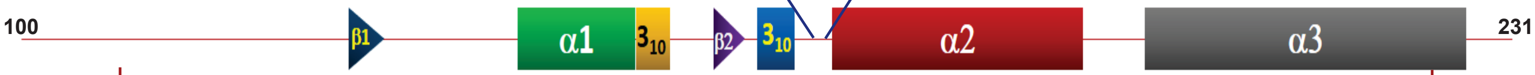

KWKPDKPKTNLKHVAGAAAAGAVVGGLGGYMLGSAMSRPVMHFGNEYEDRYYRENQYRYPNQVMYRPIDQYSNQNNFVHDCVNITVKQHTTTTTTKGENFTETDIKIMERVVEQMCITQYQTEYQAAAQRGYN-
WN S I MI DW MN Y V N I V V M V K S YYEGRSS

PrP sequence differs by 17 amino acids from the bank vole PrP (see in red)

No deletions
No insertions
No polymorphic variants found

135

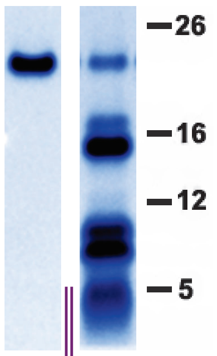

216 AA

1
Conformers

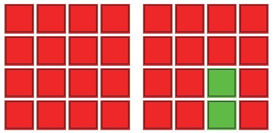

Tm^{Exp}: ND
ΔΔG: -22.28

In vitro studies

TgVole (1x) Not tested

Sugar glider Not tested

In vivo studies

TgVole (1x) Not tested

Sugar glider Not tested

pLDDT
[93.64]

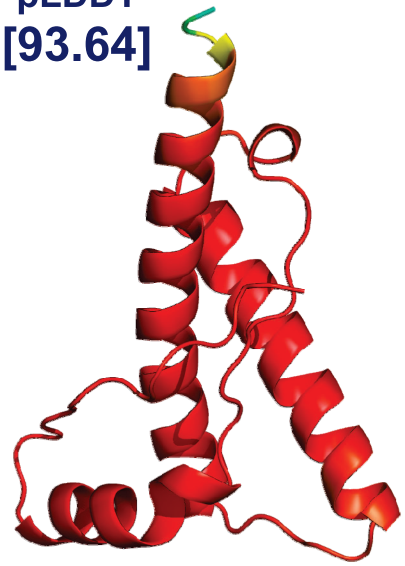

Distinct primary sequence across species

Protein code: 4816

Yellow-footed rock-wallaby

GenBank: OR47266

Petrogale xanthopus

Diprotodontia 9/18
Macropodidae 1/7

P29%

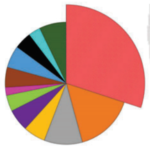

0

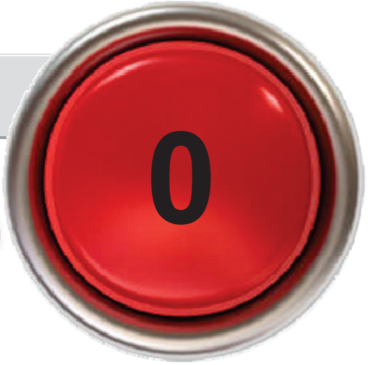

β2-α2 LOOP
SGQSSF
4

100 β1 α1 3_{10} β2 3_{10} α2 α3 231

KWKPDKPKTNLKHVAGAAAAGAVVGGLGGYMLGSAMSRPVMHFGNEYEDRYYRENQYRYPNQVMYRPIDQYSGQSSFVHDCVNITVKQHTTTTTTKGENFTETDIKIMERVVEQMCITQYQNEYQAAQRYYNM-
WN S I MI DW MN Y V NN NN I V V M V K S YYEGRSS

PrP sequence differs by 20 amino acids from the bank vole PrP (see in red)

No deletions
No insertions
No polymorphic variants found

1 species sharing the same primary sequence

Swamp wallaby
Wallabia bicolor

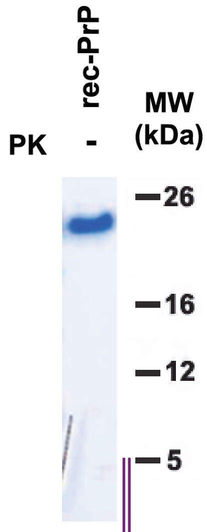

216 AA

NO MISFOLDING

0
Conformers

In vitro studies

NOT APPLICABLE

In vivo studies

NOT APPLICABLE

Tm^{Exp}: ND

ΔΔG: ND

pLDDT
[92.57]

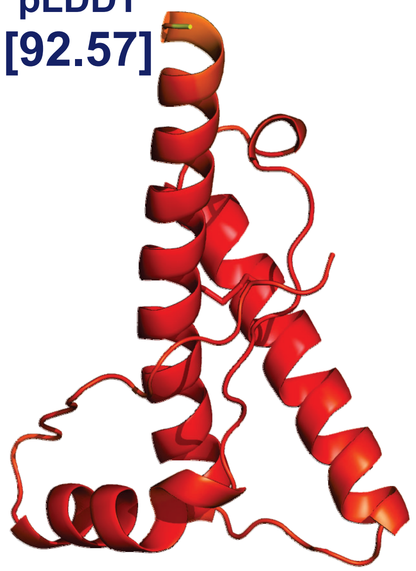

Protein code: 3762

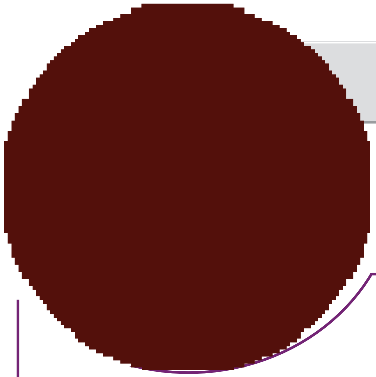

Ground cuscus

GenBank: BK064791

Phalanger gymnotis

Diprotodontia 9/18

Phalangeridae 2/2

P60%

28.6

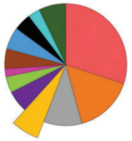

20-30

β2-α2 LOOP
SNQNNF
273

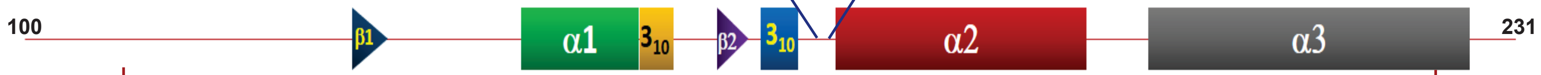

KWKPDKPKTNLKHVAGAAAAGAVVGGLGGYMLGSAMSRPVIHFGNEYEDRYYRENQYRYPNQVMYRPIDQYSNQNNFVHDCVNITVKQHTTTTTTKGENFTETDIKIMERVVEQMCITQYQAEYQAAQRGYN-

WN S I M DW MN Y V N I V V M V K S YYE RSS

PrP sequence differs by 16 amino acids from the bank vole PrP (see in red)

No deletions

No insertions

No polymorphic variants found

Distinct primary sequence across species

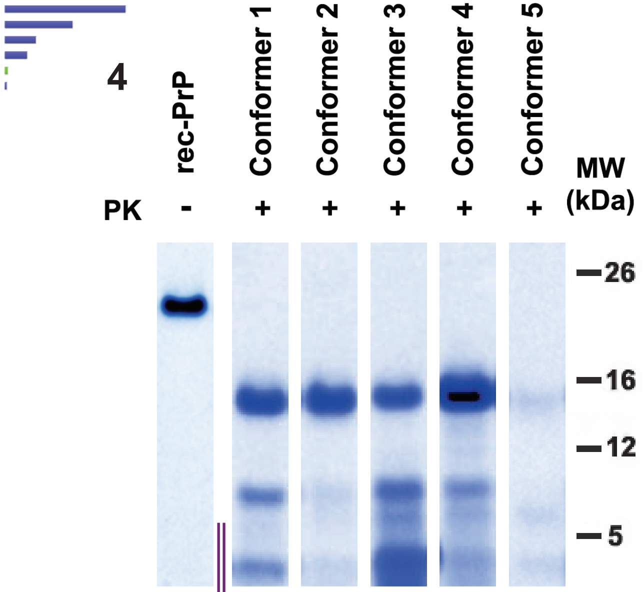

213 AA

5 Conformers

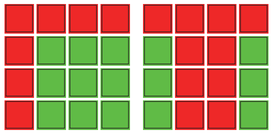

Tm^{Exp}: ND

ΔΔG: ND

In vitro studies

TgVole (1x) Not tested

Ground cuscus Not tested

In vivo studies

TgVole (1x) Not tested

Ground cuscus Not tested

pLDDT [89.54]

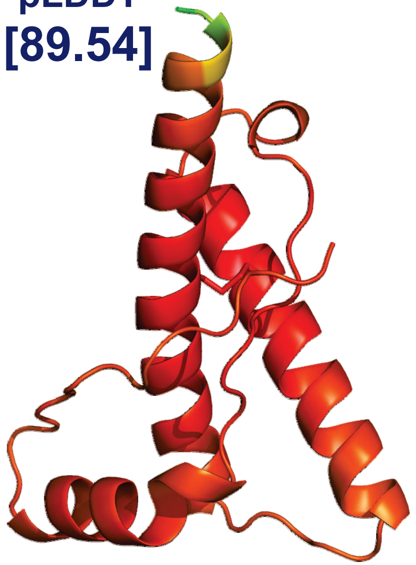

Protein code: 3956

Koala

GenBank: XM_020985342

Phascolarctos cinereus

Diprotodontia 9/18
Phascolarctidae 0/1

P29%

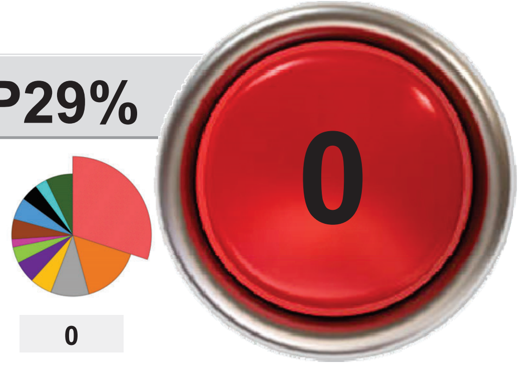

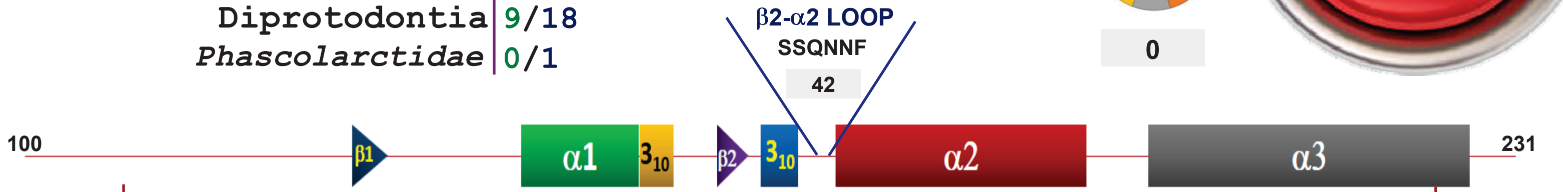

KWKPDKPKTNLKHVAGAAAAGAVVGGLGGYMLGSAMSRPIMHFGNEYEDRYYRENQYRYPNQVMYRPIDQYSSQNNFVHDCVNITVKQHTTTTTTKGENFTETDIKIMERVVEQMCITQYQNEYRAAQQGYGY-

WN S I MI DW MN Y V NN I V V M V K SQ YYE RSS

PrP sequence differs by 19 amino acids from the bank vole PrP (see in red)

No deletions
No insertions
No polymorphic variants found

Distinct primary sequence across species

Tm^{Exp}: ND

ΔΔG: ND

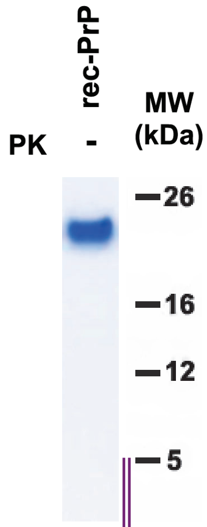

217 AA

NO MISFOLDING

0 Conformers

In vitro studies

NOT APPLICABLE

In vivo studies

NOT APPLICABLE

pLDDT [85.52]

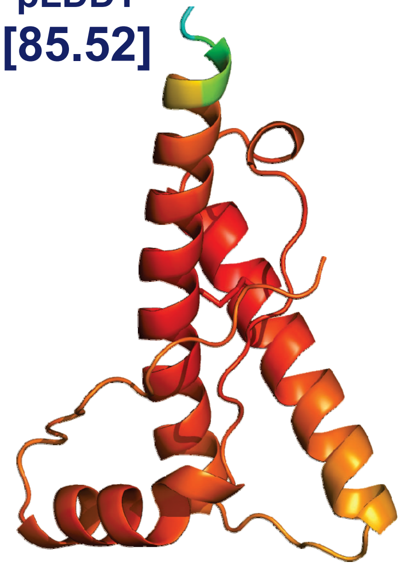

Protein code: 0935

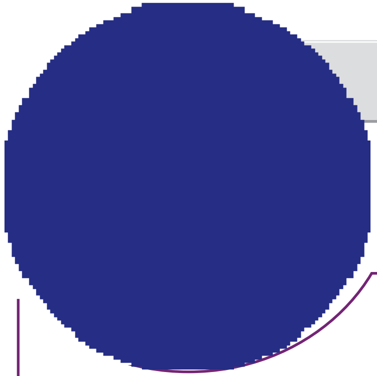

Gilbert's potoroo

GenBank: BK064793

Potorous gilbertii

Diprotodontia 9/18

Potoroidae 1/2

P29%

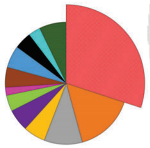

0

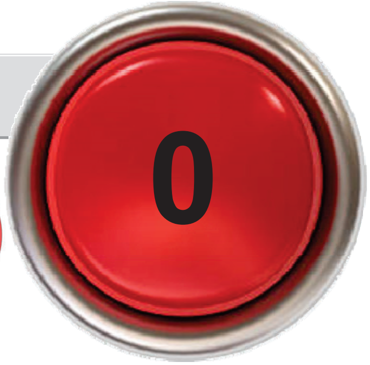

β2-α2 LOOP
SGQNSF
3

100 — β1 — α1 — 3_{10} — β2 — 3_{10} — α2 — α3 — 231

KWKPDKPKTNLKHVAGAAAAGAVVGGLGGYMLGSAMSRPVIHFGNEYEDRYYRENQYRYPNQVMYRPIDQYSGQNSFVHDCVNITVKQHTTTTTTKGENFTETDIKIMERVVEQMCITQYQNEYQAAQRYYNM-

WN S I M DW MN Y V NN N I V V M V K S YYEGRSS

PrP sequence differs by 18 amino acids from the bank vole PrP (see in red)

No deletions

No insertions

No polymorphic variants found

Tm^{Exp}: ND

ΔΔG: ND

PK

rec-PrP -

MW (kDa)

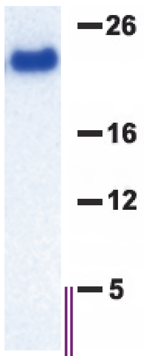

216 AA

NO MISFOLDING

0 Conformers

In vitro studies

NOT APPLICABLE

In vivo studies

NOT APPLICABLE

pLDDT [92.95]

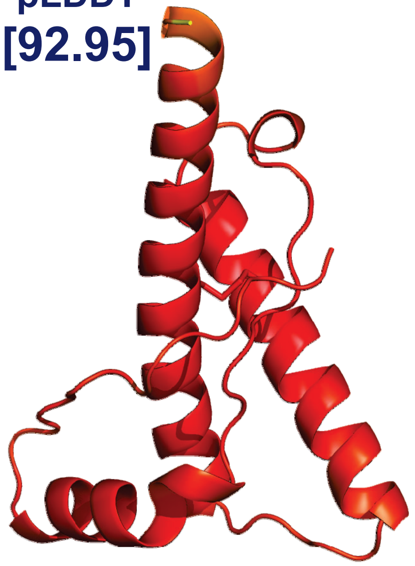

Distinct primary sequence across species

Protein code: 3958

Western ringtail

GenBank: BK064794

Pseudocheirus peregrinus

Diprotodontia 9/18
Pseudocheiridae 2/2

Distinct primary sequence across species

P70%

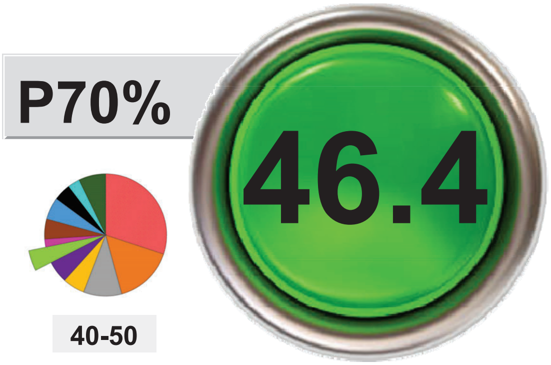

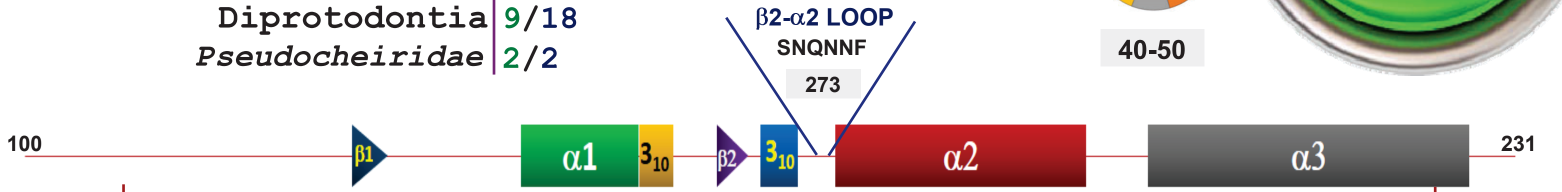

KWKPDKPKTNLKHVAGAAAAGAVVGGLGGYMLGSAMSRPVMHFGNEYEDRYYRENQYRYPNQVMYRPIDQYSNQNNFVHDCVNITVKQHTTTTTTKGENFTETDIKIMERVVEQMCITQYQNEYQAAAQRGYN-
WN S I MI DW MN Y V N I V V M V K S YYEGRSS

PrP sequence differs by 17 amino acids from the bank vole PrP (see in red)

No deletions
No insertions
No polymorphic variants found

33

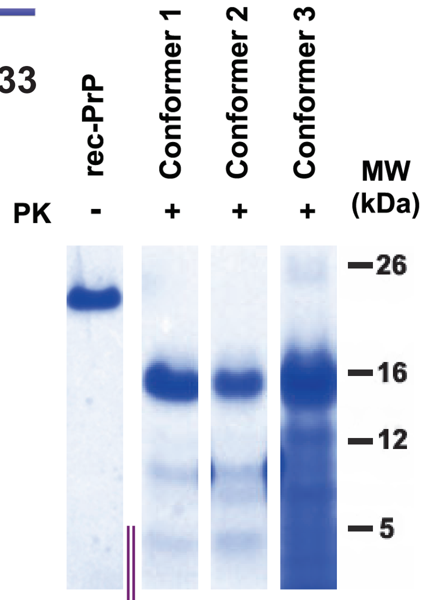

216 AA

3 Conformers

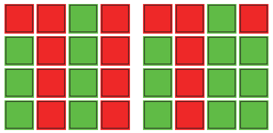

Tm^{Exp}: ND

ΔΔG: ND

In vitro studies

TgVole (1x) Not tested

Western ringtail Not tested

In vivo studies

TgVole (1x) Not tested

Western ringtail Not tested

pLDDT [88.55]

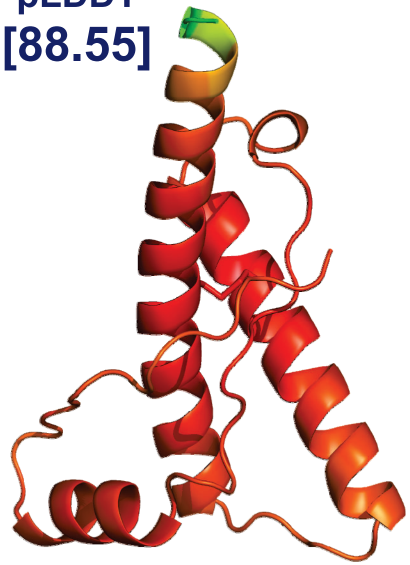

Protein code: 3959

Plush-coated ringtail possum

GenBank: BK064795

Pseudochirops corinnae

Diprotodontia 9/18
Pseudocheiridae 2/2

P49%

12.9

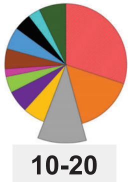

1 species sharing the same primary sequence

Coppery ringtail possum
Pseudochirops cupreus

β2-α2 LOOP
GSQNSF
2

100 — β1 — α1 — 3_{10} — β2 — 3_{10} — α2 — α3 — 231

```
KWKPDKPKTNLKHVAGAAAAGAVVGGLGGYMLGSAMSRPVMHFGNEYEDRYYRENQYRYPNQVMYRPIDQYGSQNSFVHDCVNITVKQHTTTTTTKGENFTETDIKIMERVVEQMCITQYQNEYQAAAQRGYN-
WN  S     I                           MI     DW         MN       Y   V   NN  N         I    V                    V M        V    K S  YYEGRSS
```

PrP sequence differs by 19 amino acids from the bank vole PrP (see in red)

No deletions
No insertions
No polymorphic variants found

76

216 AA

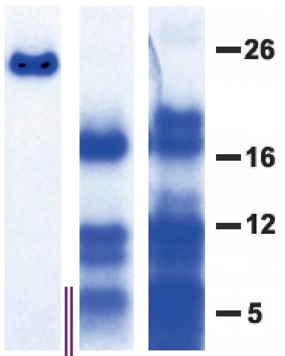

2 Conformers

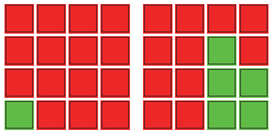

Tm^{Exp}: ND

ΔΔG: ND

In vitro studies

TgVole (1x) — Not tested

Plush-coated ringtail possum — Not tested

In vivo studies

TgVole (1x) — Not tested

Plush-coated ringtail possum — Not tested

pLDDT [88.63]

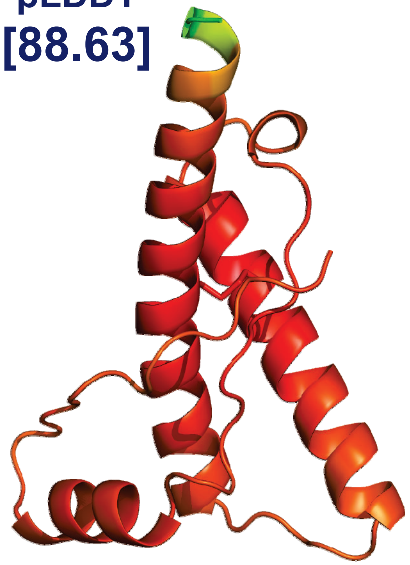

Protein code: 3960

Quokka

GenBank: BK064765

Setonix brachyurus

Diprotodontia 9/18
Macropodidae 1/7

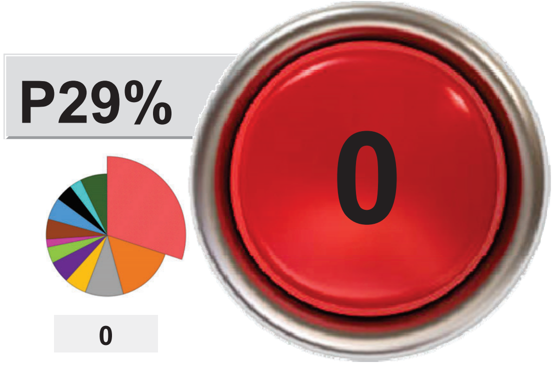

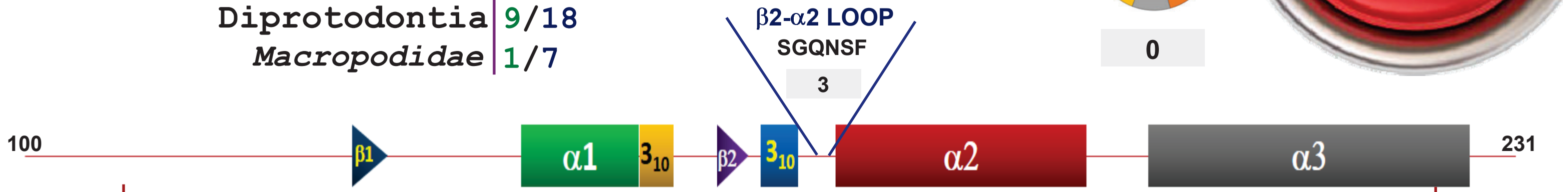

KWKPDKPKTNLKHVAGAAAAGAVVGGLGGYVLGSAMSRPVMHFGNEYEDRYYRENQYRYPNQVMYRPIDQYSGQNSFVHDCVNITVKQHTTTTTTKGENFTETDIKIMERVVEQMCITQYQNEYQAAQRYYNM-
WN S I M MI DW MN Y V NN N I V V M V K S YYEGRSS

PrP sequence differs by 20 amino acids from the bank vole PrP (see in red)

No deletions
No insertions
No polymorphic variants found

Distinct primary sequence across species

Tm[Exp]: ND
ΔΔG: ND

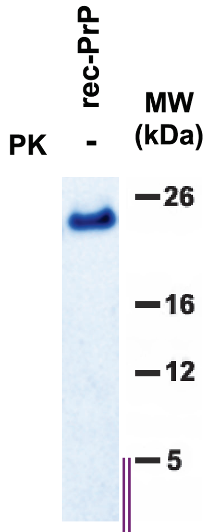

216 AA

NO MISFOLDING

0
Conformers

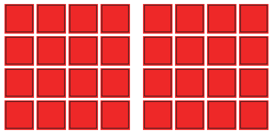

In vitro studies

NOT APPLICABLE

In vivo studies

NOT APPLICABLE

pLDDT
[93.34]

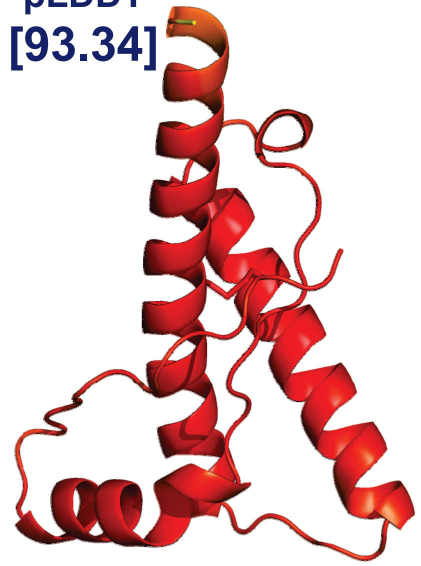

Protein code: 3862

Common brushtail possum

GenBank: L38993

P64%

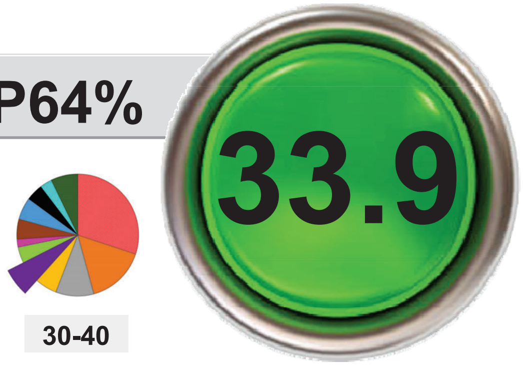

Trichosurus vulpecula

Diprotodontia 9/18
Phalangeridae 2/2

Distinct primary sequence across species

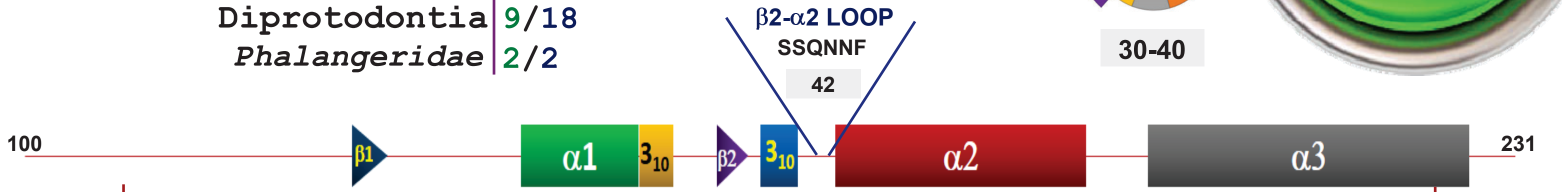

KWKPDKPKTNLKHVAGAAAAGAVVGGLGGYMLGSAMSRPVIHFGNEYEDRYYRENQYRYPNQVMYRPIDQYSSQNNFVHDCVNITVKQHTTTTTTKGENFTETDIKIMERVVEQMCITQYQAEYEAAAQRAYN-

WN S I M DW MN Y V NN I V V M V K SQ YYEGRSS

PrP sequence differs by 18 amino acids from the bank vole PrP (see in red)

No deletions

No insertions

No polymorphic variants found

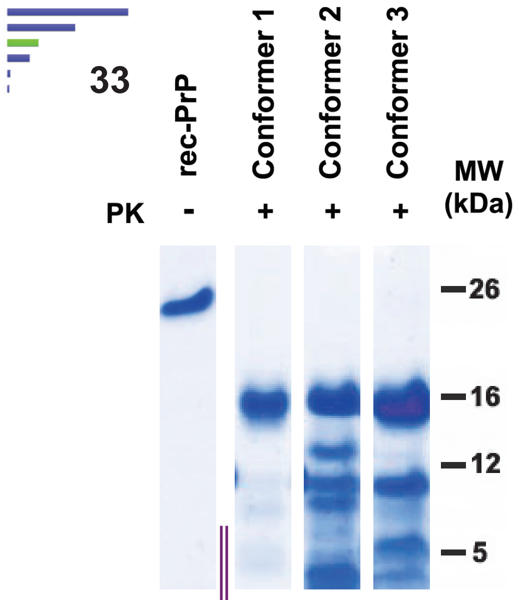

214 AA

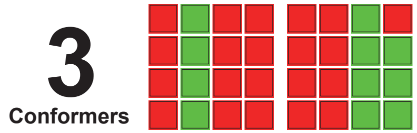

Tm^{Exp}: ND

ΔΔG: ND

In vitro studies

TgVole (1x) Not tested

Common brushtail possum Not tested

In vivo studies

TgVole (1x) Not tested

Common brushtail possum Not tested

pLDDT [92.87]

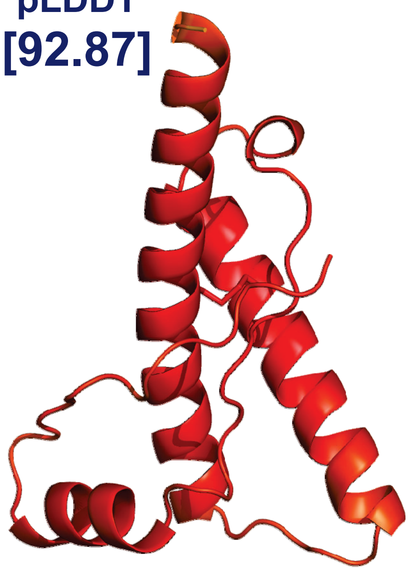

Protein code: 1335

Common wombat

GenBank: XM_027865324

Vombatus ursinus

Diprotodontia 9/18
Vombatidae 1/1

P45%

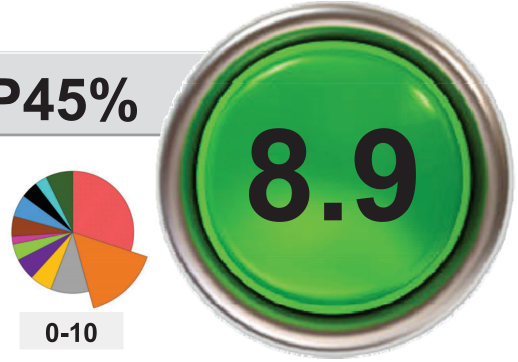

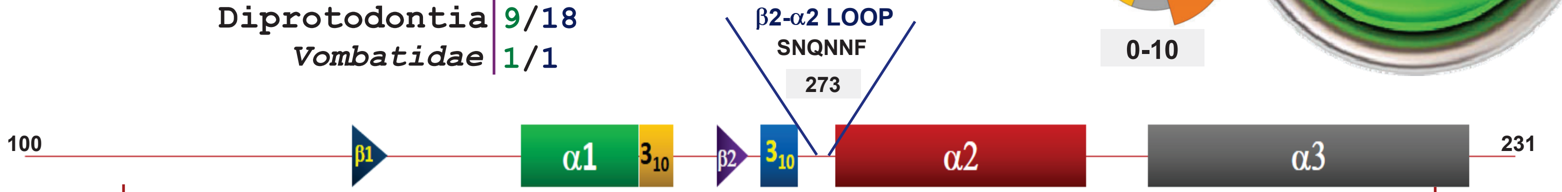

KWKPDKPKTNLKHVAGAAAAGAVVGGLGGYMLGSAMSRPVIHFGNDYEDRYYRENQYRYPNQVMYRPIDQYSNQNNFVHDCVNITVKQHTTTTTTKGENFTETDIKIMERVVEQMCITQYQNEYRAAQQGYYY-
WN S I M W MN Y V N I V V M V K SQ YYE RSS

PrP sequence differs by 16 amino acids from the bank vole PrP (see in red)

No deletions
No insertions
No polymorphic variants found

Distinct primary sequence across species

135

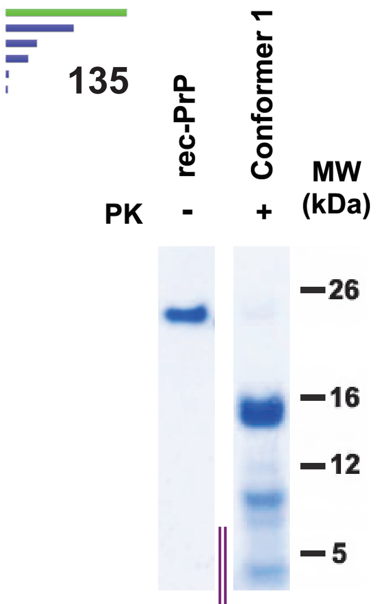

214 AA

1
Conformers

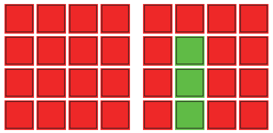

Tm^{Exp}: ND

ΔΔG: ND

In vitro studies

TgVole (1x) Not tested

Common wombat Not tested

In vivo studies

TgVole (1x) Not tested

Common wombat Not tested

pLDDT [90.42]

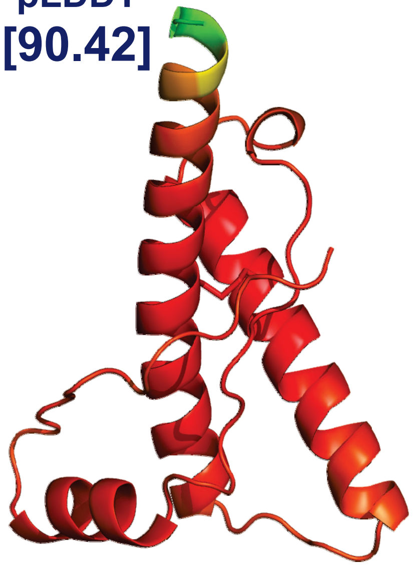

Protein code: 1100

Four-toed hedgehog

GenBank: OR47273

P58%

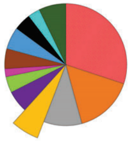

25.4

20-30

Atelerix albiventris

Erinaceomorpha 3/3
Erinaceidae 3/3

Distinct primary sequence across species

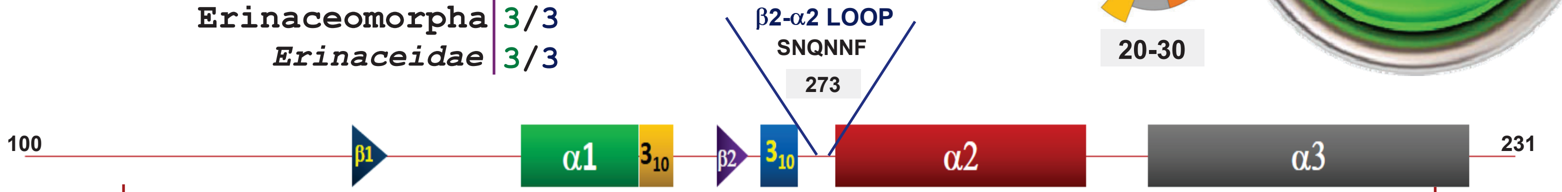

WNKPNKPKTNIKHVAGAAAAGAVVGGLGGYLVGSAMSRPPIHFGNDYEDRYYRENINRYPNQVYYKPVDQYSNQNNFVHDCVNITVKQHTVTTTTKGENFTETDIKIMERVVEQMCITQYQQARAYHNGAS-
S ML M W M R N I V M V KESQAYYEGRS

PrP sequence differs by 16 amino acids from the bank vole PrP (see in red)

No deletions
No insertions
No polymorphic variants found

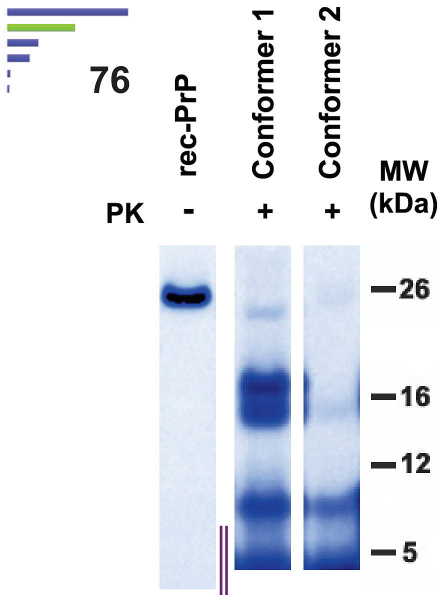

226 AA

2 Conformers

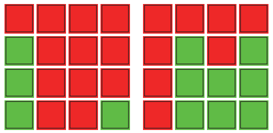

Tm^{Exp}: ND

ΔΔG: ND

In vitro studies

TgVole (1x) Not tested

Four-toed hedgehog Not tested

In vivo studies

TgVole (1x) Not tested

Four-toed hedgehog Not tested

pLDDT [88.74]

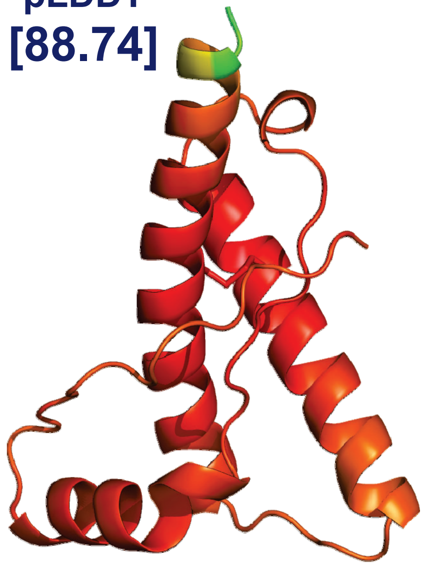

Protein code: 4029

European hedgehog

GenBank: EU572708

Erinaceus europaeus

Erinaceomorpha 3/3

Erinaceidae 3/3

Distinct primary sequence across species

P34%

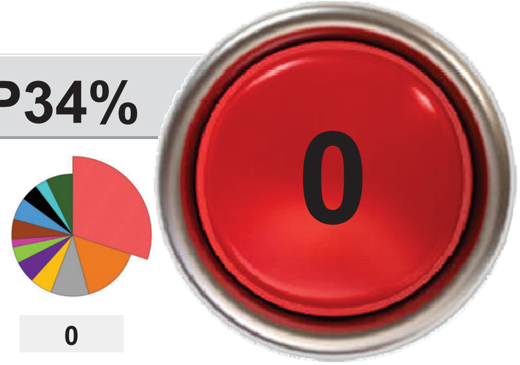

β2-α2 LOOP
SNQNNF
273

100 β1 α1 3_{10} β2 3_{10} α2 α3 231

WNKPNKPKTNMKHVAGAAAAGAVVGGLGGYLVGSAMSRPPIHFGNDYEDRYYRENINRYPNQVYYKPVDQYSNQNNFVHDCVNITVKQHTVTTTTKGENFTETDVKIMERVVEQMCITQYQQARAYHDGAS-

PrP sequence differs by 16 amino acids from the bank vole PrP (see in red)

No deletions

No insertions

No polymorphic variants found

Tm^{Exp}: ND

ΔΔG: ND

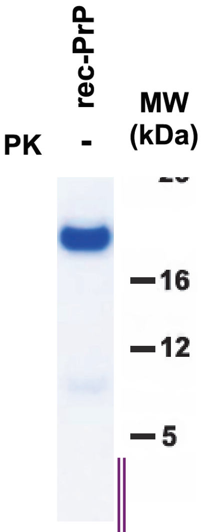

216 AA

NO MISFOLDING

0 Conformers

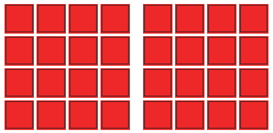

In vitro studies

NOT APPLICABLE

In vivo studies

NOT APPLICABLE

pLDDT [90.76]

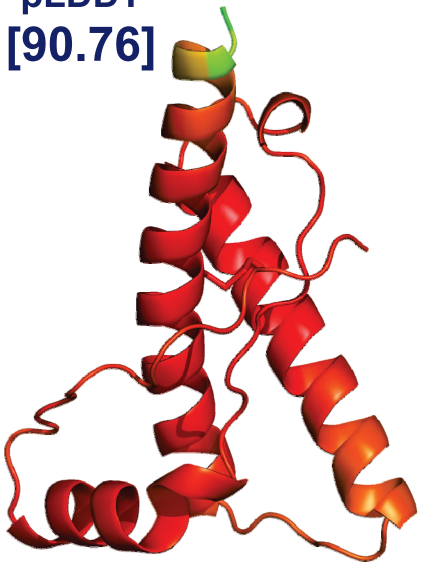

Protein code: 0931

Short-tailed gymnure

GenBank: AY133044

Hylomys suillus

Erinaceomorpha 3/3

Erinaceidae 3/3

P65% **36.1**

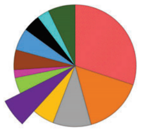

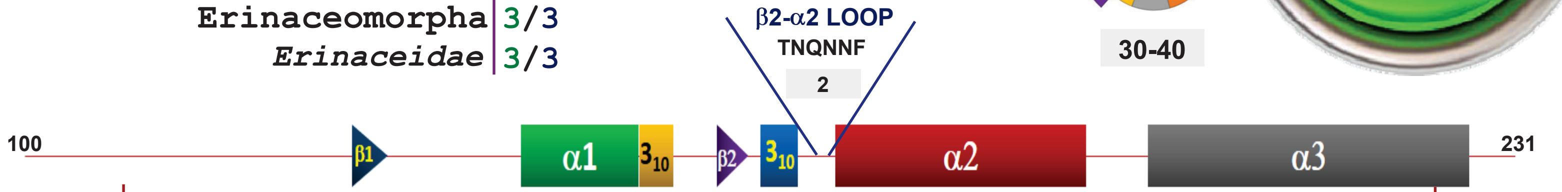

WGKPSKPKTNMKHVAGAAAAGAVVGGLGGYMLGSAMSRPIIHFGNDYEDRYYRENMNRYPNQVYYRPVDQYTNQNNFVHDCTNITIKQHTVTTTTKGENFTETDIKIMERVVEQMCITQYQEAYRAYQQNHGT-

N I M W N V V M V KESQ YEGRSS

PrP sequence differs by 12 amino acids from the bank vole PrP (see in red)

No deletions

No insertions

No polymorphic variants found

Distinct primary sequence across species

76

223 AA

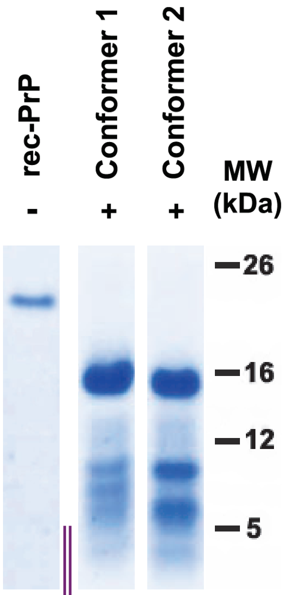

2 Conformers

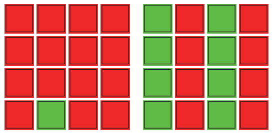

Tm[Exp]: ND

ΔΔG: -10.37

pLDDT [90.72]

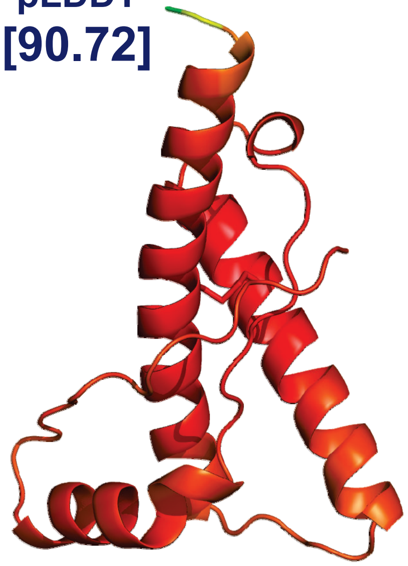

In vitro studies

TgVole (1x) — Not tested

Short-tailed gymnure — Not tested

In vivo studies

TgVole (1x) — Not tested

Short-tailed gymnure — Not tested

Protein code: 1344

Rock hyrax

GenBank: AY133057

Procavia capensis

Hyracoidea 0/1
Procaviidae 0/1

P29%

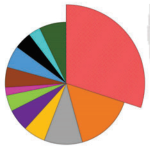

0

0

1 species sharing the same primary sequence

Yellow-spotted rock hyrax
Heterohyrax brucei

β2-α2 LOOP
SSENNF
8

100 — β1 — α1 — 3_{10} — β2 — 3_{10} — α2 — α3 — 231

```
WGKPSKPKTNMKHVAGAAAAGAVVGGLGGYMLGSAMSRPLIHFGNDYEDRYYRENMYRYPNQVYYRPVNEYSSENNFVHDCVNITVKQHTVTTTTKGENFTETDIKIMERVVEQMCMTQYQKEFQAYQRGSS-
 N        I                              M      W          N          DQ NNQ          I                 V M         V      S  YE R S
```

PrP sequence differs by 14 amino acids from the bank vole PrP (see in red)

No deletions
No insertions
No polymorphic variants found

Tm^{Exp}: ND
ΔΔG: ND

PK / **rec-PrP** -
MW (kDa)

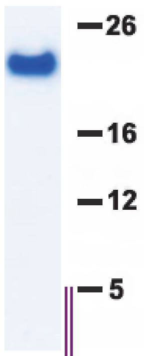

211 AA

NO MISFOLDING

0 Conformers

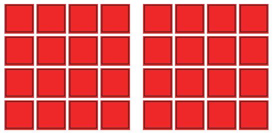

In vitro studies

NOT APPLICABLE

In vivo studies

NOT APPLICABLE

pLDDT [85.66]

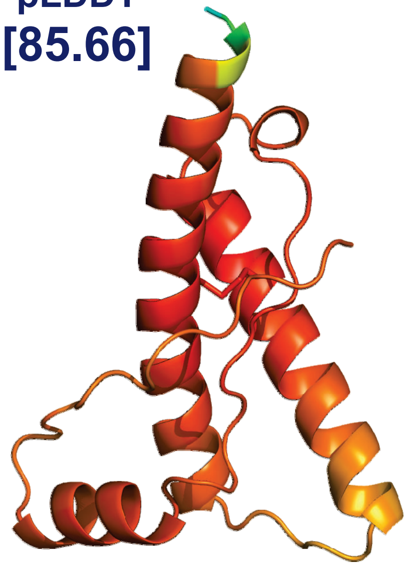

Protein code: 0952

Brown hare

GenBank: OR472454

Lepus europaeus

Lagomorpha 4/5

Leporidae 3/4

P40%

5.9

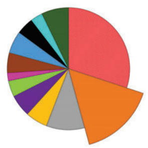

0-10

β2-α2 LOOP

SNQNSF

36

100 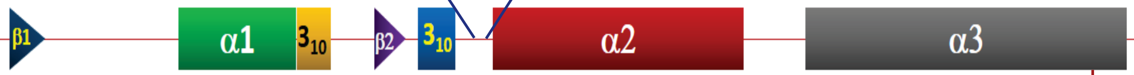

231

WGKPSKPKTSMKHVAGAAAAGAVVGGLGGYMLGSAMSRPLIHFGNDYEDRYYRENMYRYPNQVYYRPVDQYSNQNSFVHDCVNITVKQHTVTTTTKGENFTETDIKIMERVVEQMCITQYQQEYQAAYQRAAG-

N NI M W N N N I V M V K S Y EGRSS

PrP sequence differs by 13 amino acids from the bank vole PrP (see in red)

No deletions

No insertions

No polymorphic variants found

3 species sharing the same primary sequence

Snowshoe hare *Lepus americanus*

Mountain hare *Lepus timidus*

White-tailed jackrabbit *Lepus townsendii*

135

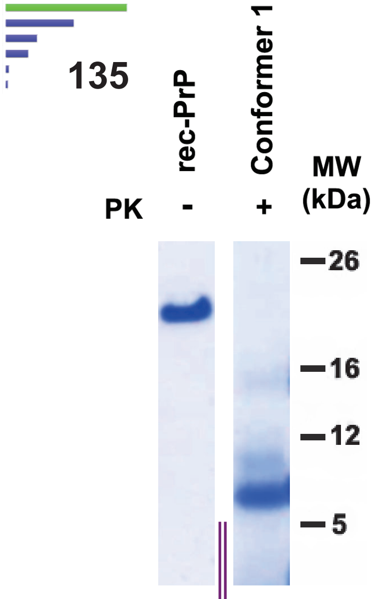

210 AA

1

Conformers

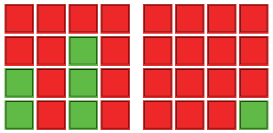

Tm^{Exp}: ND

ΔΔG: -17.76

In vitro studies

TgVole (1x) Not tested

Brown hare Not tested

In vivo studies

TgVole (1x) Not tested

Brown hare Not tested

pLDDT

[90.29]

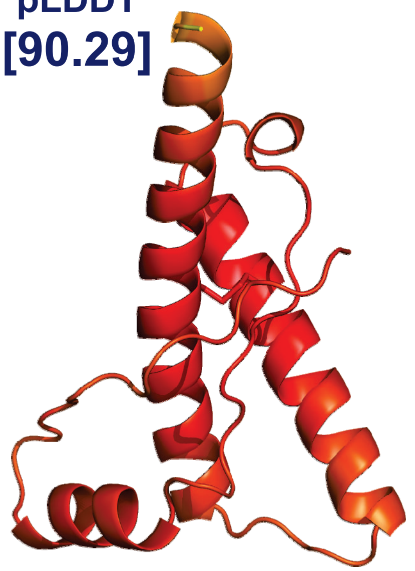

Protein code: 0533

American pika

GenBank: EU555402

Ochotona princeps

Lagomorpha 4/5
Ochotonidae 1/1

Plateau pika
Ochotona curzoniae

1 species sharing the same primary sequence

P53%

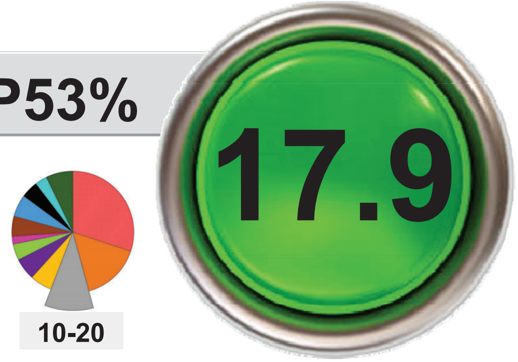

β2-α2 LOOP
SNQNNF
273

100 β1 α1 3_{10} β2 3_{10} α2 α3 231

WGKPSKPKTNMKHVAGAAAAGAVVGGLGGYMLGSAMSRPIIHFGNEYEDRYYRDNMYRYPNQVYYKPVDQYSNQNNFVHDCVNITIKQHTVTTTTKGENFTETDIKMMERVVEQMCVTQYQQEYQASYQRAAS-

N I M DW E N R N V K S Y EGRS

PrP sequence differs by 11 amino acids from the bank vole PrP (see in red)

No deletions
No insertions
No polymorphic variants found

135

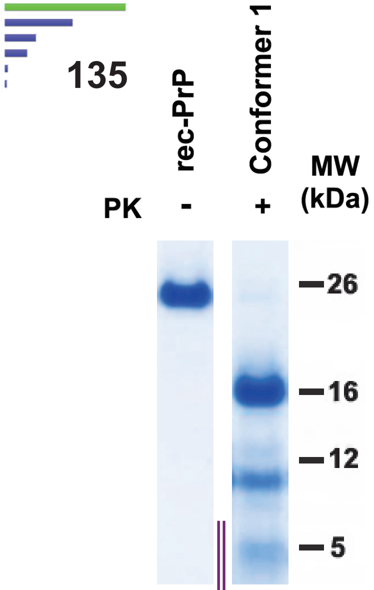

210 AA

1
Conformers

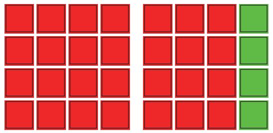

Tm^{Exp}: ND

ΔΔG: -12.67

In vitro studies

TgVole (1x) Not tested

American pika Not tested

In vivo studies

TgVole (1x) Not tested

American pika Not tested

pLDDT
[88.07]

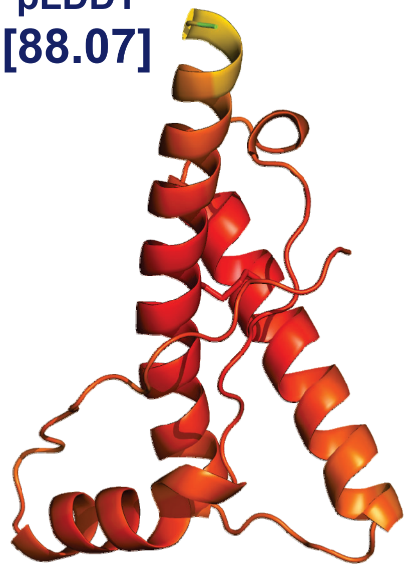

Protein code: 1140

Rabbit

GenBank: NM_001082021

Oryctolagus cuniculus

Lagomorpha 4/5
Leporidae 3/4

P34%

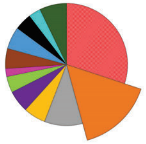

0-10

0.9

β2-α2 LOOP
SNQNSF
36

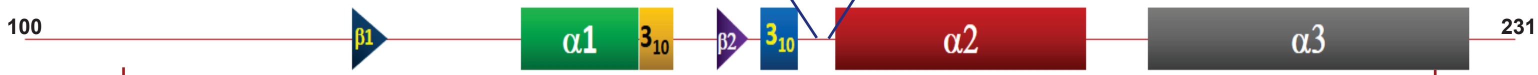

WGKPSKPKTSMKHVAGAAAAGAVVGGLGGYMLGSAMSRPLIHFGNDYEDRYYRENMYRYPNQVYYRPVDQYSNQNSFVHDCVNITVKQHTVTTTTKGENFTETDIKIMERVVEQMCITQYQQESQAAYQRAAG-
N NI M W N N N I V M V K Y EGRSS

PrP sequence differs by 12 amino acids from the bank vole PrP (see in red)

No deletions
No insertions
No polymorphic variants found

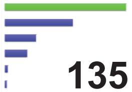

135

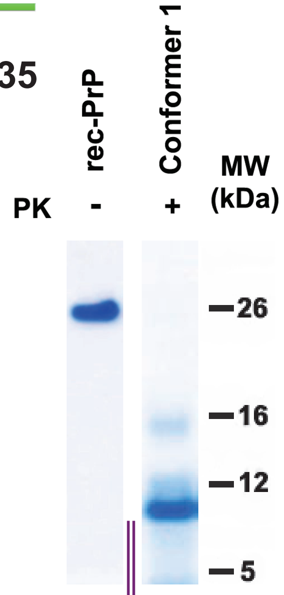

210 AA

1
Conformers

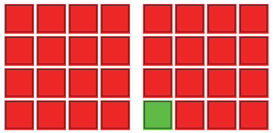

Tm[Exp]: ND

ΔΔG: -13.56

In vitro studies

TgVole (1x) Propagate

Rabbit Ongoing

In vivo studies

TgVole (1x) Infectious

TgRabbit Ongoing

pLDDT
[89.85]

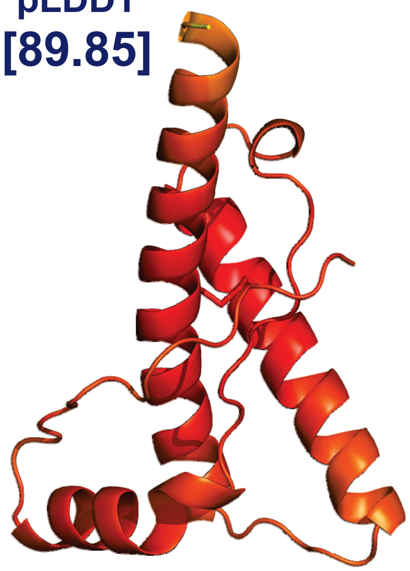

Distinct primary sequence across species

Protein code: 0135

Brush rabbit

GenBank: BK063974

P49%

12.5

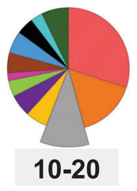

Sylvilagus bachmani

Lagomorpha 4/5

Leporidae 3/4

β2-α2 LOOP
SNQNSF
36

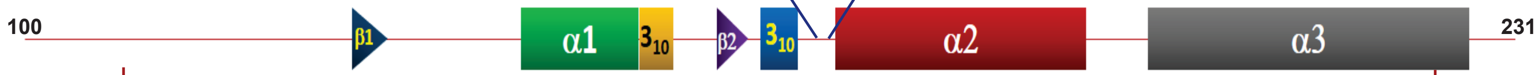


```
WGKPSKPKTSMKHVAGAAAAGAVVGGLGGYMLGSAMSRPLIHFGNDYEDRYYRENMYRYPNQVYYRPVEQYSNQNSFVHDCVNITVKQHTVTTTTKGENFTETDIKIMERVVEQMCITQYQQEYQAAYQRAAG-
 N       NI                               M      W         N           D  N   N         I                       V M          V     K S   Y EGRSS
```

PrP sequence differs by 14 amino acids from the bank vole PrP (see in red)

No deletions

No insertions

No polymorphic variants found

76

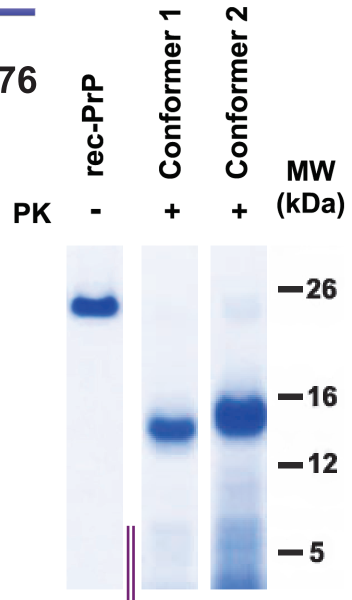

210 AA

2
Conformers

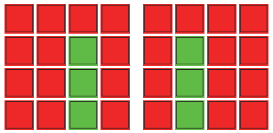

Tm^{Exp}: ND

ΔΔG: -15.86

In vitro studies

TgVole (1x) Not tested

Brush rabbit Not tested

In vivo studies

TgVole (1x) Not tested

Brush rabbit Not tested

pLDDT [89.61]

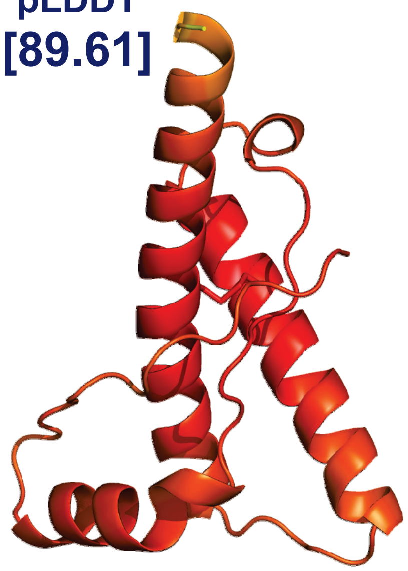

Distinct primary sequence across species

Protein code: 3131

New England cottontail

GenBank: BK064962

Sylvilagus transitionalis

Lagomorpha 4/5

Leporidae 3/4

Distinct primary sequence across species

P29%

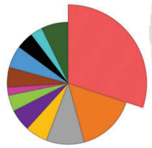

0

0

β2-α2 LOOP
SNQNSF
36

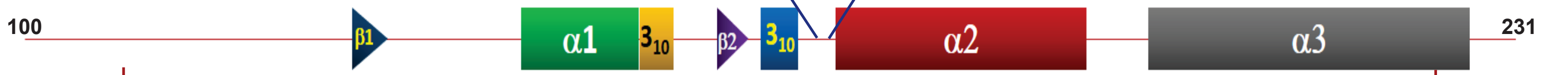

WGKPSKPKTSMKHVAGAAAAGAVVGGLGGYMLGSAMSRPLIHFGNDYEDRYYRENMYRYPNQVYYRPVDQYSNQNSFVHDCVNITVKQHTVTTTTKGENFTETDIKIMERVVEQMCITQYQQEYQAAYQRGAS-

N NI M W N N N I V M V K S Y EGRS

PrP sequence differs by 13 amino acids from the bank vole PrP (see in red)

No deletions

No insertions

No polymorphic variants found

Tm^{Exp}: ND

ΔΔG: -18.5

PK

rec-PrP

-

MW (kDa)

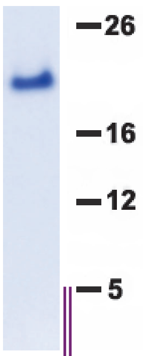

210 AA

NO MISFOLDING

0

Conformers

In vitro studies

NOT APPLICABLE

In vivo studies

NOT APPLICABLE

pLDDT

[92.35]

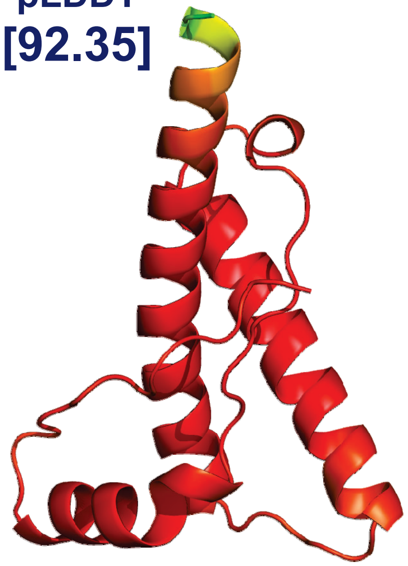

Protein code: 4742

Cape elephant shrew

GenBank: XM_006894222

Elephantulus edwardii

Macroscelidea 1/2

Macroscelididae 1/2

P29%

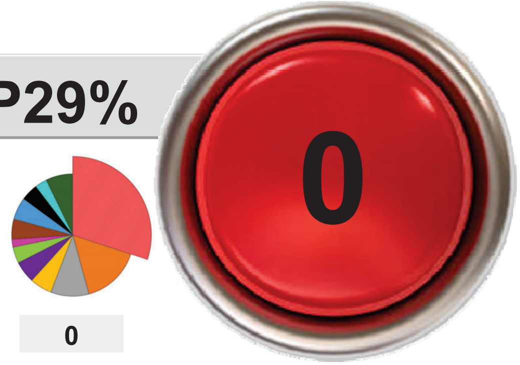

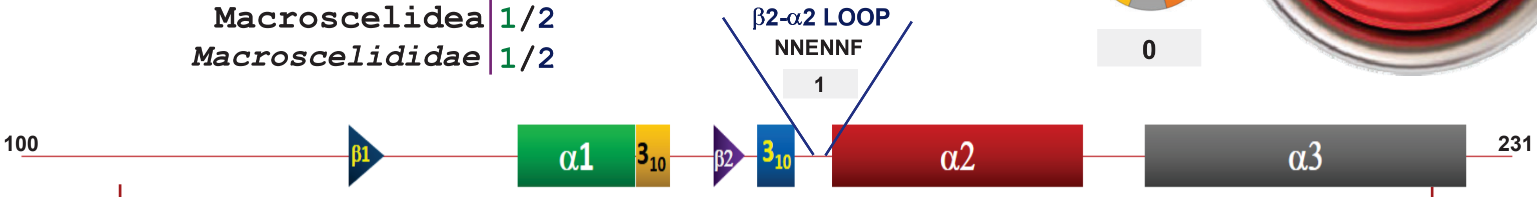

WGKPNKPKTNMKNMAGAAAAGAVVGGLGGYMLGSAMSRPPMHFGNDYEDRYYRENMNRYPNQVYYYKPVNQYNNENNFVHDCVNITVKQHTTTTTTKGENFTETDIKIMERVVEQMCITQYQKESMAYQRGSS-

N S I HV MI W R D Q I V V M V Q YE R S

PrP sequence differs by 15 amino acids from the bank vole PrP (see in red)

No deletions

No insertions

No polymorphic variants found

Tm^{Exp}: ND

ΔΔG: ND

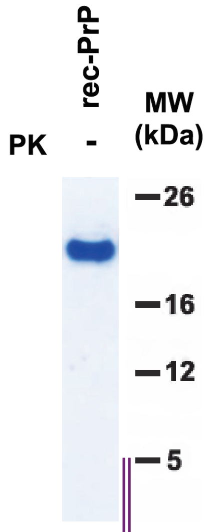

213 AA

NO MISFOLDING

0 Conformers

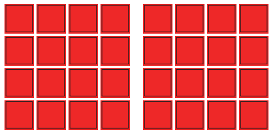

In vitro studies

NOT APPLICABLE

In vivo studies

NOT APPLICABLE

pLDDT [88.74]

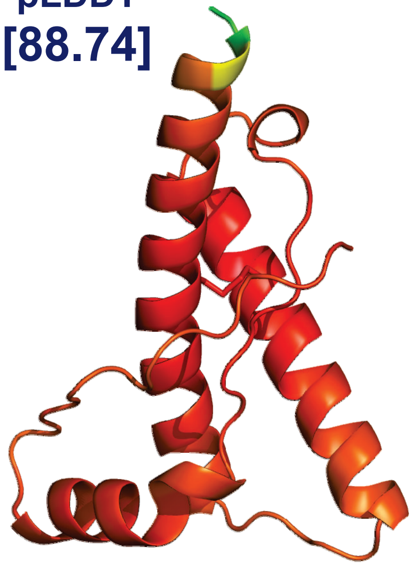

Distinct primary sequence across species

Protein code: 0972

Round-eared elephant shrew

GenBank: AY133059

Macroscelides proboscideus

Macroscelidea 1/2
Macroscelididae 1/2

P58%

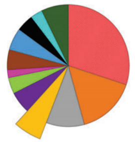

20-30

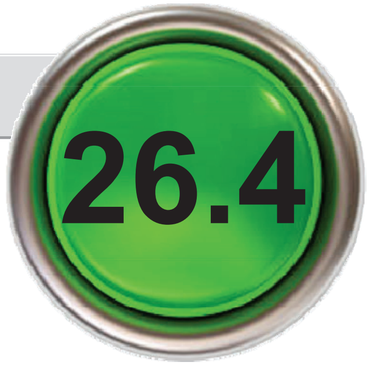

β2-α2 LOOP
SNENNF
2

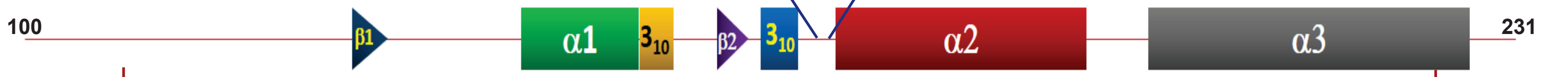

WGKPNKPKTNLKNMAGAAAAGAVVGGLGGYMLGSAMSRPIMHFGNDYEDRYYRENMNRYPNQVYYKPVNQYSNENNFVHDCVNITIKQHTTTTTTKGENFTETDIKIMERVVEQMCTTQYQKESMAYQLGSNA-
N S I HV MI W R D N Q V V M V Q YE RSS

PrP sequence differs by 15 amino acids from the bank vole PrP (see in red)

No deletions
No insertions
No polymorphic variants found

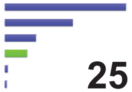

25

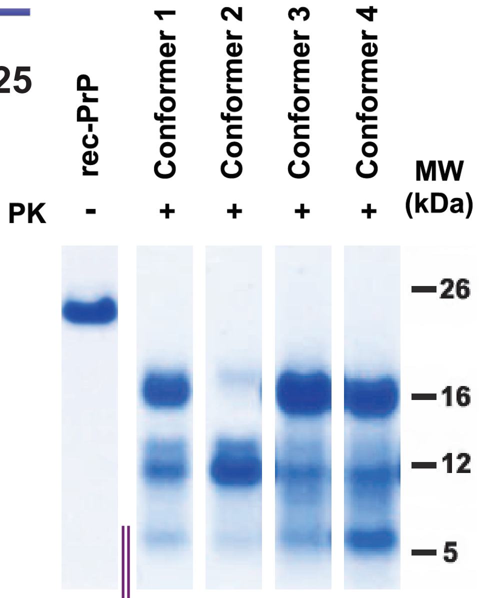

215 AA

4 Conformers

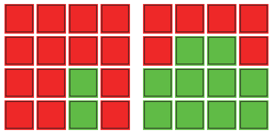

Tm^{Exp}: ND

ΔΔG: -9.98

In vitro studies

Not tested
TgVole (1x)

Not tested
Round-eared elephant shrew

In vivo studies

Not tested
TgVole (1x)

Not tested
Round-eared elephant shrew

pLDDT
[89.61]

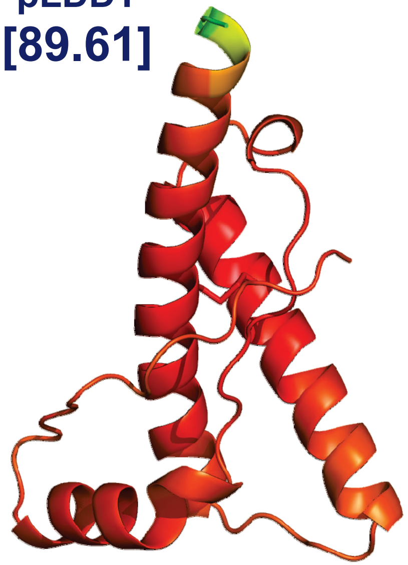

Distinct primary sequence across species

Protein code: 1343

Colocolo opossum

GenBank: BK064161

P39%

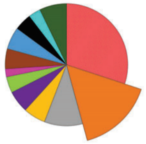

0-10

3.6

1 species sharing the same primary sequence

Pancho's colocolo opossum
Dromiciops bozinovici

Dromiciops gliroides

Microbiotheria 1/1
Microbiotheriidae 1/1

β2-α2 LOOP
NNQNNF
164

100 β1 α1 3_{10} β2 3_{10} α2 α3 231

```
KWKPDKPKTNMKHVAGAAAAGAVVGGLGGYMLGSAMSRPAMHFGNDYEDRYYRENQNRYPNQVMYRPVDQYNNQNNFVHDCVNITVKQHTTTTTTKGENFTETDIKIMERVVEQMCITQYQNEQRAAQYNYNI-
WN  S    I                              MI     W        M       Y                       I    V                V M        V    K SQ YYEGRSS
```

PrP sequence differs by 14 amino acids from the bank vole PrP (see in red)

No deletions
No insertions
No polymorphic variants found

135

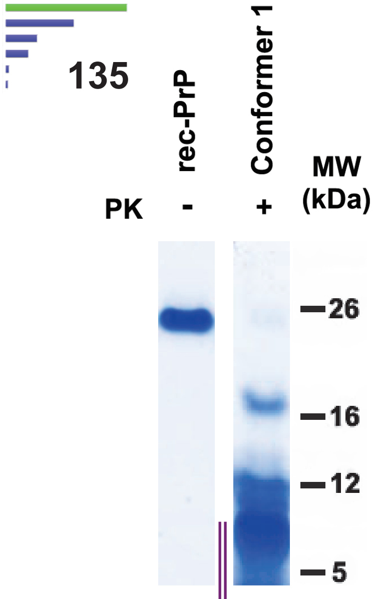

202 AA

1
Conformers

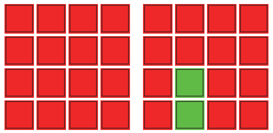

Tm[Exp]: ND
ΔΔG: ND

In vitro studies

No propagation
TgVole (1x)

Not tested
Colocolo opossum

In vivo studies

Ongoing
TgVole (1x)

Not tested
Colocolo opossum

pLDDT
[88.92]

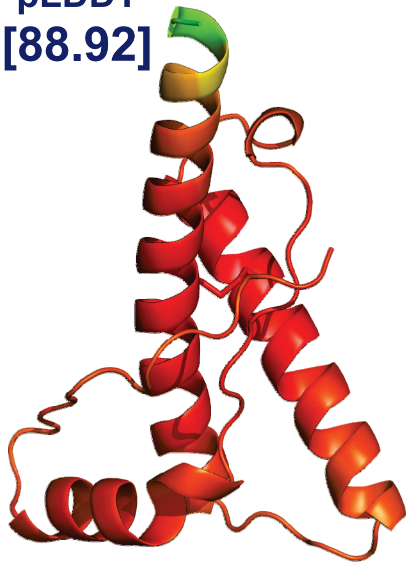

Protein code: 3732

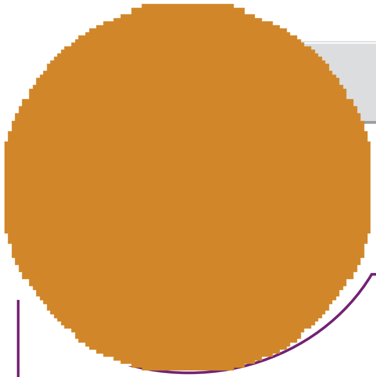

Platypus

GenBank: XM_029065640

Ornithorhynchus anatinus

Monotremata 0/2

Ornithorhynchidae 0/1

P29%

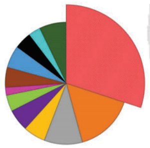

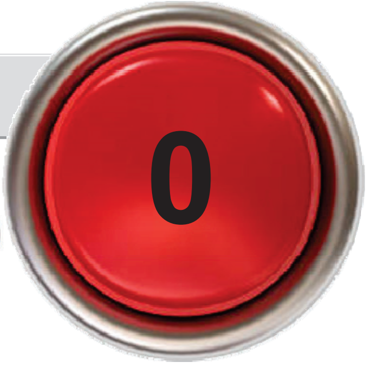

0

β2-α2 LOOP
GSQDGF
14

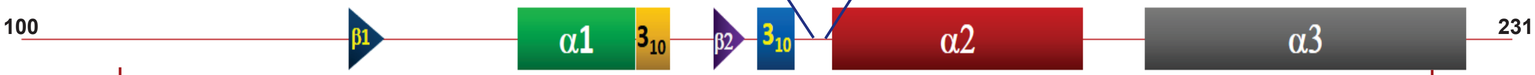

KYKPDKPKTGMKHVAGAAAAGAVVGGLGGYMIGSAMSRPPMHFGNEFEDRYYRENQNRYPNQVYYRPVDQYGSQDGFVRDCVNITVTQHTVTTTE GKNLNETDVKIMTRVVEQMCVNQYRAEYQARSS-

WN S NI L MI DW M NN NN H IK TK E FT M E T QK S YYEG

PrP sequence differs by 26 amino acids from the bank vole PrP (see in red)

One deletion

No insertions

No polymorphic variants found

Distinct primary sequence across species

PK -

rec-PrP

MW (kDa)

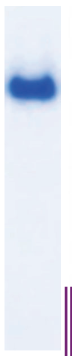

208 AA

NO MISFOLDING

0

Conformers

In vitro studies

NOT APPLICABLE

In vivo studies

NOT APPLICABLE

Tm^{Exp}: ND

ΔΔG: ND

pLDDT [86.68]

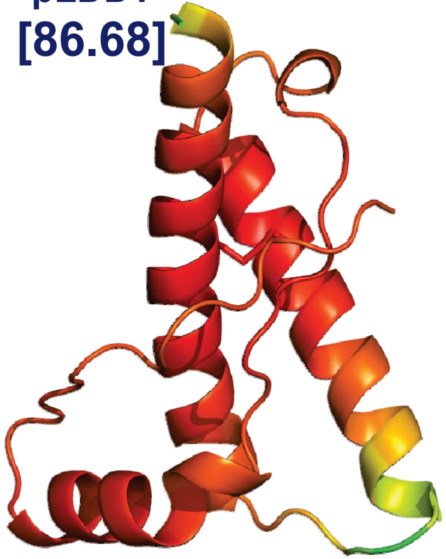

Protein code: 0944

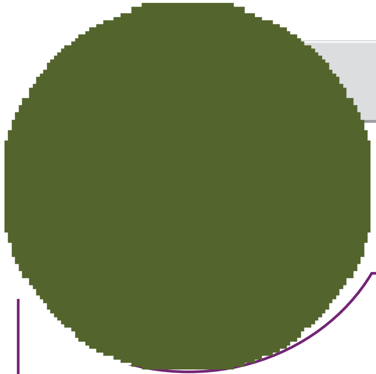

Short-beaked echidna

GenBank: BK063950

Tachyglossus aculeatus

Monotremata 0/2
Tachyglossidae 0/1

Distinct primary sequence across species

P29%

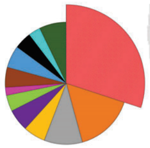

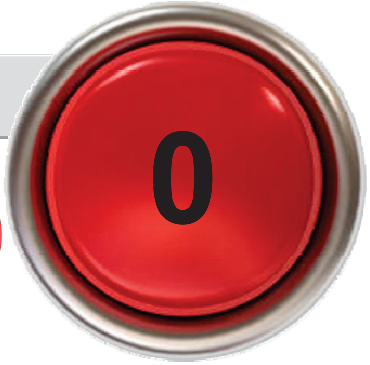

0

β2-α2 LOOP
GSQDGF
14

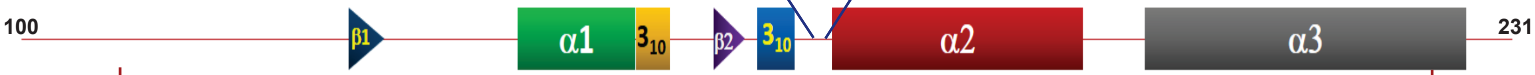


```
KYKPDKPKTSMKHVAGAAAAGAVVGGLGGYMLGSAMSRPPMHFGNDYEDRYYRENQYRYPNQVYYYRPVDQYGSQDGFVRDCVNITVTQHTVTTTE GKNFNETDVKIMTRVVEQMCVNQYQAEYRARS-
WN  S    NI                          MI    W         MN                NN NN  H      IK        TK E  T     M E         T   K SQ YYE
```

PrP sequence differs by 24 amino acids from the bank vole PrP (see in red)

One deletion
No insertions
No polymorphic variants found

Tm^{Exp}: ND

ΔΔG: ND

rec-PrP
PK -
MW (kDa)

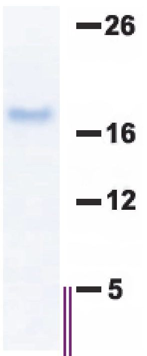

197 AA

NO MISFOLDING

0
Conformers

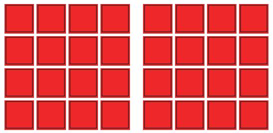

In vitro studies

NOT APPLICABLE

In vivo studies

NOT APPLICABLE

pLDDT
[88.52]

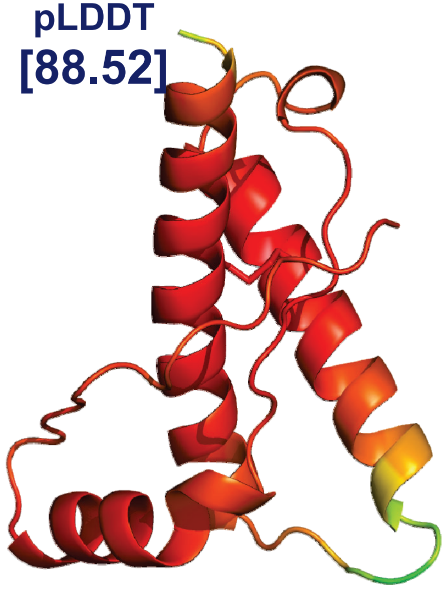

Protein code: 3077

Southern marsupial mole

GenBank: BK064812

Notoryctes typhlops

Notoryctemorphia 0/1

Notoryctidae 0/1

P29%

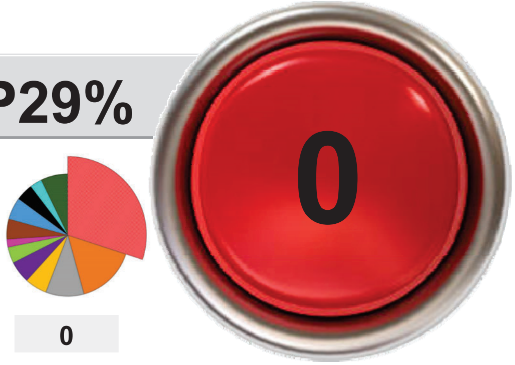

β2-α2 LOOP
SNQNNF
273

100 β1 α1 3_{10} β2 3_{10} α2 α3 231

KWKPDKPKTNLKHVAGAAAAGAVVGGLGGYMLGSAMSRPIMHFGNDYEDRYYRENQYRYPNQVMYRPIDQYSNQNNFVHDCVNITVKEHTTTTTTKGENFTETDIKIMERVVEQMCITQYQNEYAAQYRYNMA-

WN S I MI W MN Y V N I Q V V M V K SQ Y EGRSS

PrP sequence differs by 18 amino acids from the bank vole PrP (see in red)

No deletions

No insertions

No polymorphic variants found

Distinct primary sequence across species

Tm^{Exp}: ND

ΔΔG: -15.2

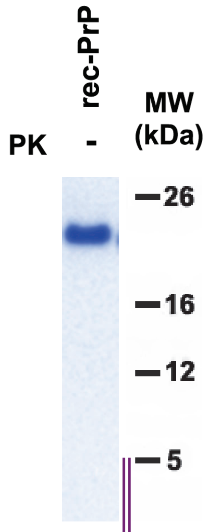

215 AA

NO MISFOLDING

0 Conformers

In vitro studies

NOT APPLICABLE

In vivo studies

NOT APPLICABLE

pLDDT [93.3]

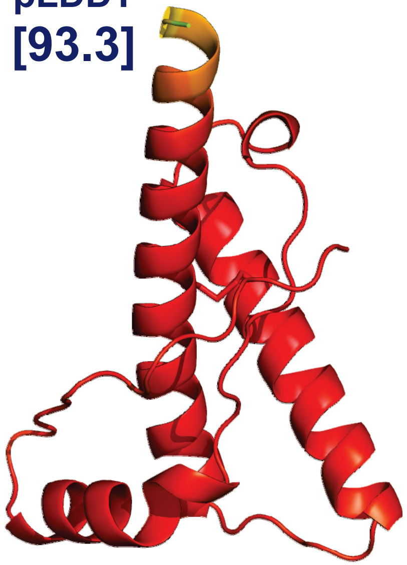

Protein code: 4081

Southern white rhinoceros

GenBank: XM_014790100

Ceratotherium simum simum

Perissodactyla 6/6
Rhinocerotidae 1/1

P36%

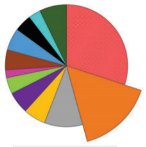

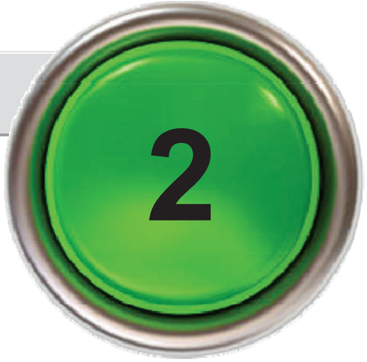

3 species sharing the same primary sequence

Bornean rhinoceros
Dicerorhinus sumatrensis harrissoni

Black rhinoceros
Diceros bicornis

White rhinoceros
Rhinoceros unicornis

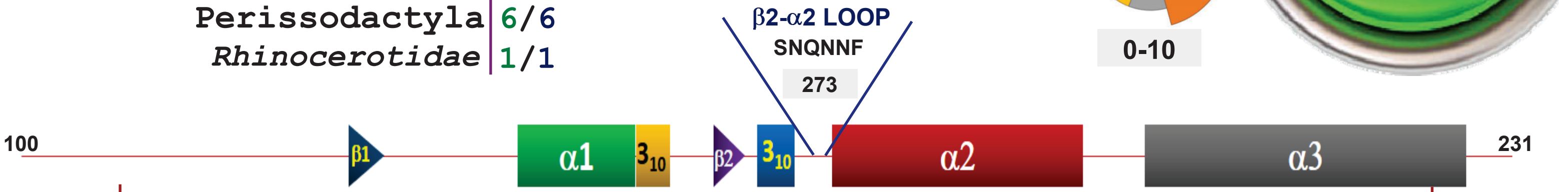

WNKPSKPKTNMKHMAGAAAAGAVVGGLGGYMLGSAMSRPLIHFGNDYEDRYYRENMYRYPNQVYYRPVSEYSNQNNFVHDCVNITVKQHTVTTTTKGENFTETDVKMMERVVEQMCITQYQREYEAFQQRGAS-
I V M W N DQ N I V K SQ YYEGRS

PrP sequence differs by 13 amino acids from the bank vole PrP (see in red)

No deletions
No insertions
No polymorphic variants found

135

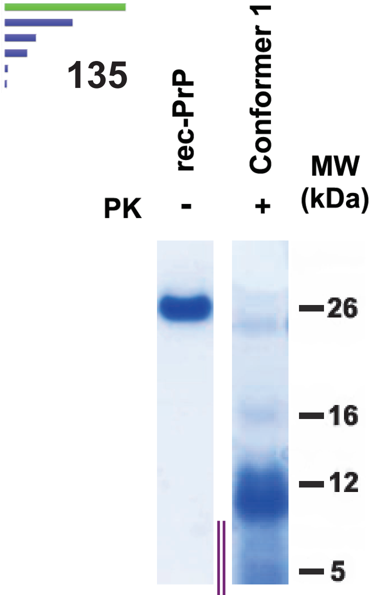

212 AA

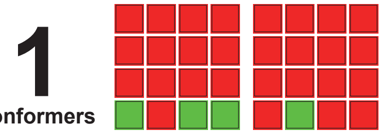

Tm^{Exp}: ND

ΔΔG: -11.01

pLDDT [88.31]

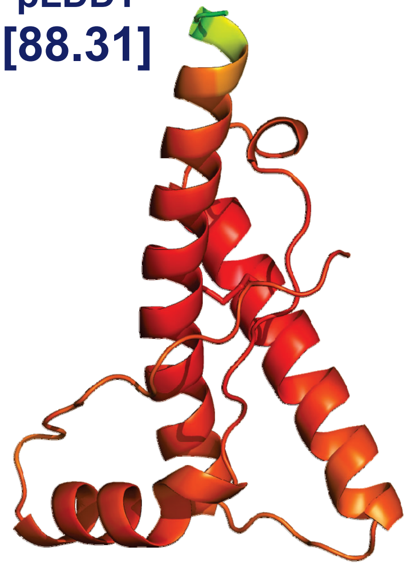

In vitro studies

TgVole (1x)	Not tested
Southern white rhinoceros	Not tested

In vivo studies

TgVole (1x)	Not tested
Southern white rhinoceros	Not tested

Protein code: 0955

Asinus

GenBank: BK064942

Equus asinus

Perissodactyla 6/6
Equidae 3/3

1 species sharing the same primary sequence

African wild ass
Equus africanus

P39%

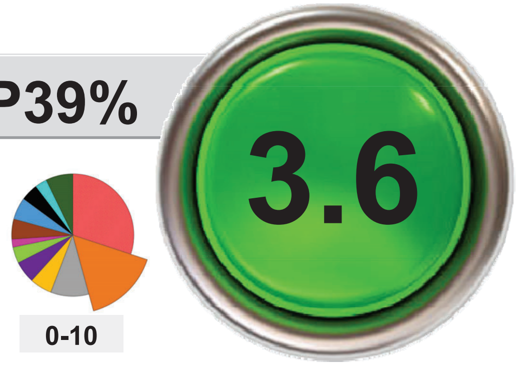

β2-α2 LOOP
SNQNNF
273

100 β1 α1 3_{10} β2 3_{10} α2 α3 231

WNKPSKPKTNMKHVAGAAAAGAVVGGLGGYMLGSAMSRPLIHFGNDYEDRYYRENMYRYPNQVYYRPVNEYSNQNNFVHDCVNITVKQHTVTTTTKGENFTETDVKIMERVVEQMCITQYQKEYEAFQQRGAS-
I M W N DQ N I M V SQ YYEGRS

PrP sequence differs by 12 amino acids from the bank vole PrP (see in red)

No deletions
No insertions
It shows polymorphic variants

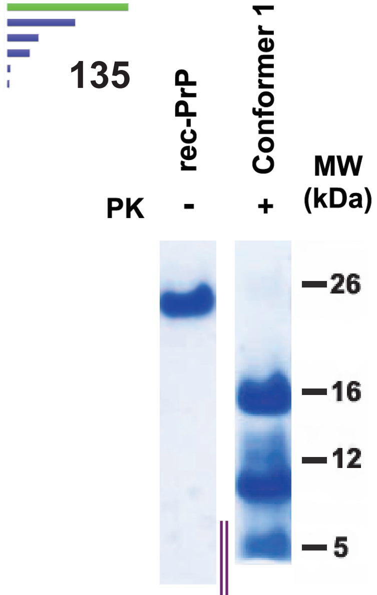

211 AA

1
Conformers

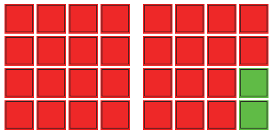

Tm[Exp]: ND

ΔΔG: -2.76

In vitro studies

TgVole (1x) Not tested

Asinus Not tested

In vivo studies

TgVole (1x) Not tested

Asinus Not tested

pLDDT
[86.98]

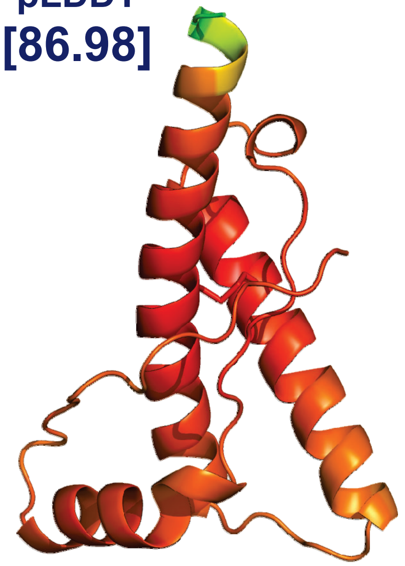

Protein code: 1070

Horse

GenBank: EU887260

Equus caballus ferus

Perissodactyla 6/6

Equidae 3/3

P41%

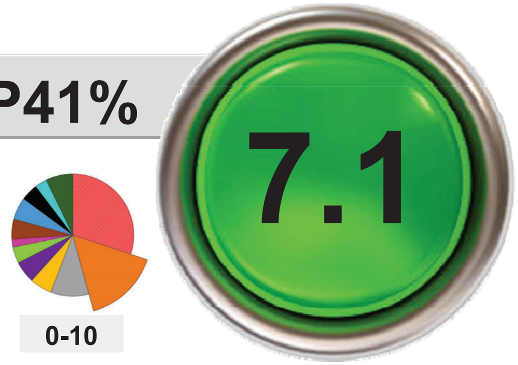

β2-α2 LOOP
SNQNNF
273

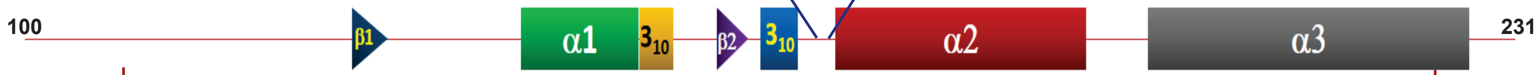

WNKPSKPKTNMKHVAGAAAAGAVVGGLGGYMLGSAMSRPLIHFGNDYEDRYYRENMYRYPNQVYYRPVSEYSNQNNFVHDCVNITVKQHTVTTTTKGENFTETDVKIMERVVEQMCITQYQKEYEAFQQRGAS-

I M W N DQ N I M V SQ YYEGRS

PrP sequence differs by 12 amino acids from the bank vole PrP (see in red)

No deletions

No insertions

It shows polymorphic variants

5 species sharing the same primary sequence

Burchell's zebra *Equus burchellii*

Grévy's zebra *Equus grevyi*

Kiang *Equus kiang*

Grant's zebra *Equus quagga boehmi*

Mountain zebra *Equus zebra hartmannae*

135

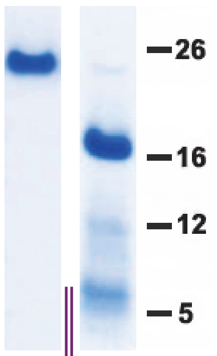

211 AA

1 Conformers

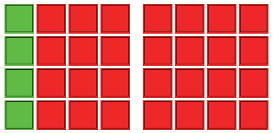

Tm^{Exp}: ND

ΔΔG: -4.71

In vitro studies

TgVole (1x) Propagate

Horse Ongoing

In vivo studies

TgVole (1x) Ongoing

Horse Not tested

pLDDT [88.55]

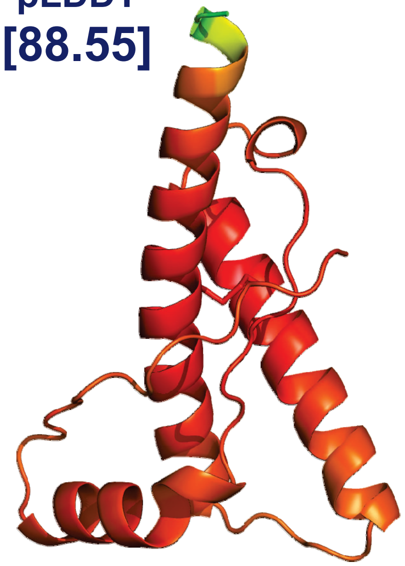

Protein code: 0115

Przewalski's horse

GenBank: XM_008519788

Equus caballus przewalskii

`Perissodactyla` 6/6
`Equidae` 3/3

P51% **14.3**

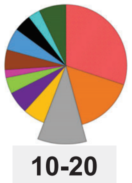

β2-α2 LOOP
SNQNNF
273

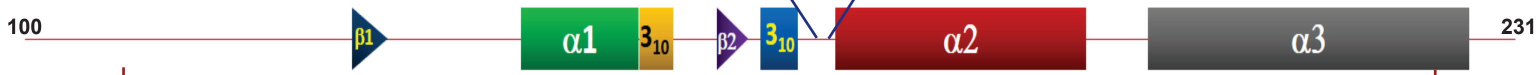

WNKPSKPKTNIKHVAGAAAAGAVVGGLGGYMLGSAMSRPLIHFGNDYEDRYYRENMYRYPNQVYYRPVSEYSNQNNFVHDCVNITVKQHTVTTTTKGENFTETDVKIMERVVEQMCITQYQKEYEAFQQRGAS-
M W N DQ N I M V SQ YYEGRS

PrP sequence differs by 11 amino acids from the bank vole PrP (see in red)

No deletions
No insertions
No polymorphic variants found

Distinct primary sequence across species

135

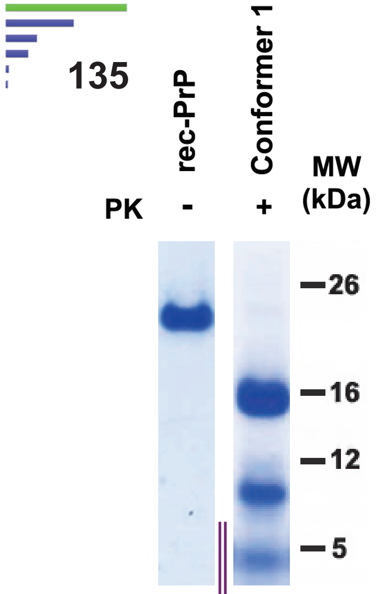

211 AA

1
Conformers

Tm^{Exp}: ND

ΔΔG: -8.75

In vitro studies

TgVole (1x) Not tested

Przewalski's horse Not tested

In vivo studies

TgVole (1x) Not tested

Przewalski's horse Not tested

pLDDT
[87.91]

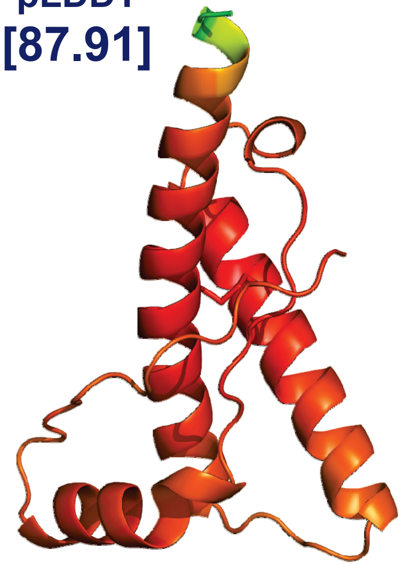

Malayan tapir

GenBank: BK063977

Tapirus indicus

Perissodactyla 6/6

Tapiridae 2/2

P51%

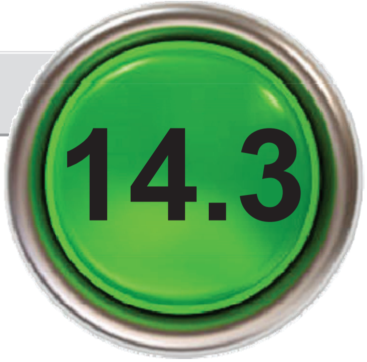

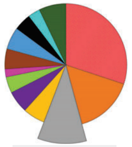

10-20

β2-α2 LOOP
SNQNNF
273

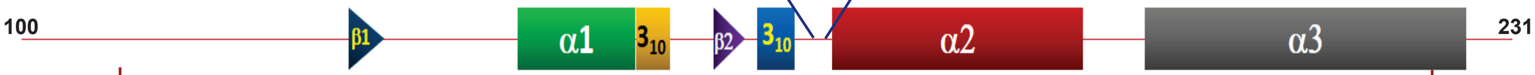

WNKPSKPKTNMKHVAGAAAAGAVVGGLGGYMLGSAMSRPLIHFGNDYEDRYYRENMYRYPNQVYYRPVSEYSNQNNFVHDCVNITVKQHTVTTTTKGENFTETDVKMMERVVEQMCITQYQREYEAFQQRGAS-

I M W N DQ N I V K SQ YYEGRS

PrP sequence differs by 12 amino acids from the bank vole PrP (see in red)

No deletions

No insertions

No polymorphic variants found

Distinct primary sequence across species

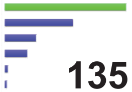

135

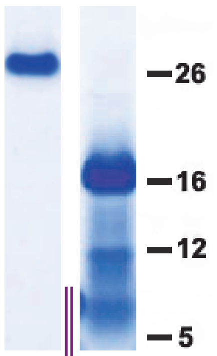

212 AA

1

Conformers

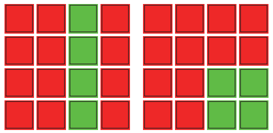

Tm^{Exp}: ND

ΔΔG: -9.07

In vitro studies

TgVole (1x) Not tested

Malayan tapir Not tested

In vivo studies

TgVole (1x) Not tested

Malayan tapir Not tested

pLDDT [88.49]

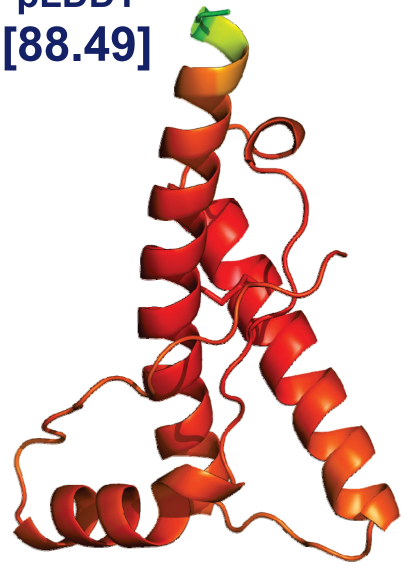

Protein code: 3134

South American tapir

GenBank: BK063978

Tapirus terrestris

Perissodactyla 6/6
Tapiridae 2/2

1 species sharing the same primary sequence

Baird's tapir
Tapirus bairdii

P51%

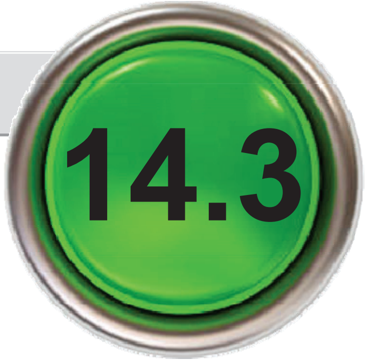

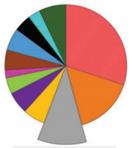

10-20

β2-α2 LOOP
SNQNNF
273

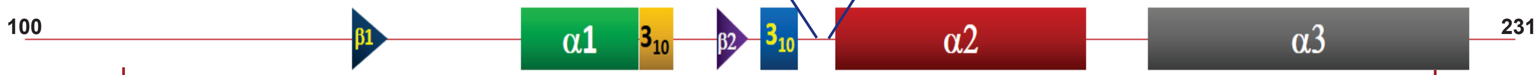


```
WNKPSKPKTNMKHMAGAAAAGAVVGGLGGYMLGSAMSRPLIHFGSDYEDRYYRENMHRYPNQVYYYRPVSEYSNQNNFVHDCVNITVKQHTVTTTTKGENFTETDVKIMERVVEQMCITQYQREYEAFQQRGAS-
  I  V                            M     N W          N            DQ N                I                          M           V     K SQ YYEGRS
```

PrP sequence differs by 15 amino acids from the bank vole PrP (see in red)

No deletions
No insertions
No polymorphic variants found

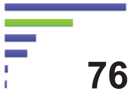

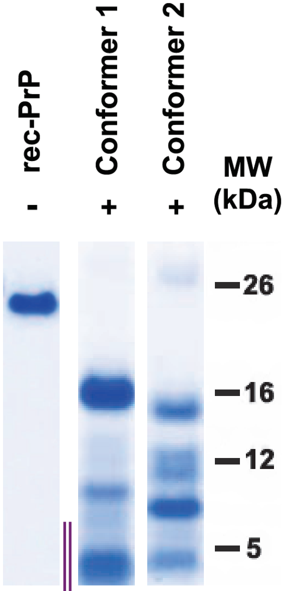

212 AA

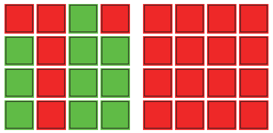

Tm^{Exp}: ND

ΔΔG: -7.74

In vitro studies

Not tested
TgVole (1x)

Not tested
South American tapir

In vivo studies

Not tested
TgVole (1x)

Not tested
South American tapir

pLDDT
[88.48]

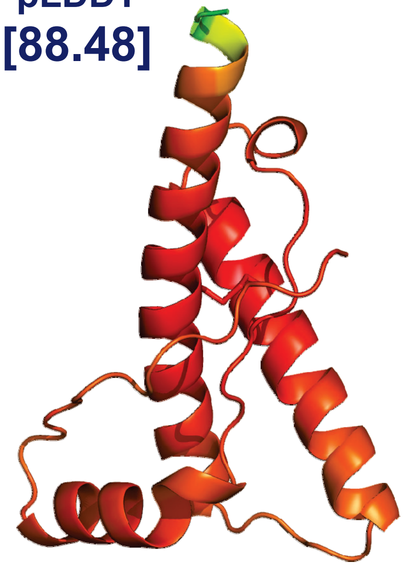

Protein code: 3135

Pangolin

GenBank: XM_017663893

P66%

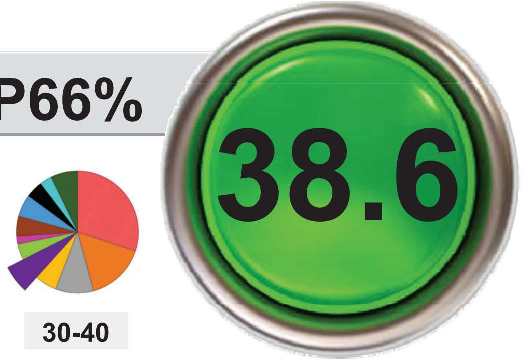

Manis javanica

Pholidota 3/3
Manidae 3/3

β2-α2 LOOP
NNQNNF
164

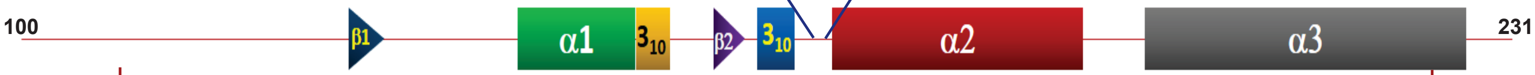


```
WGKPSKPKTNMKHVAGAAAAGAVVGGLGGYMLGSAMSRPLIHFGNDYEDRYYRENMYRYPNQVYYRPVDQYNNQNNFVHDCVNITVKQHTVTTTTKGENFTETDIKMMERVVEQMCITQYQKEYQAASYQRGAS-
 N        I                        M      W         N                                       I                  V          V       S  YYEG SS
```

PrP sequence differs by 8 amino acids from the bank vole PrP (see in red)

No deletions
No insertions
No polymorphic variants found

1 species sharing the same primary sequence

Indian pangolin
Manis crassicaudata

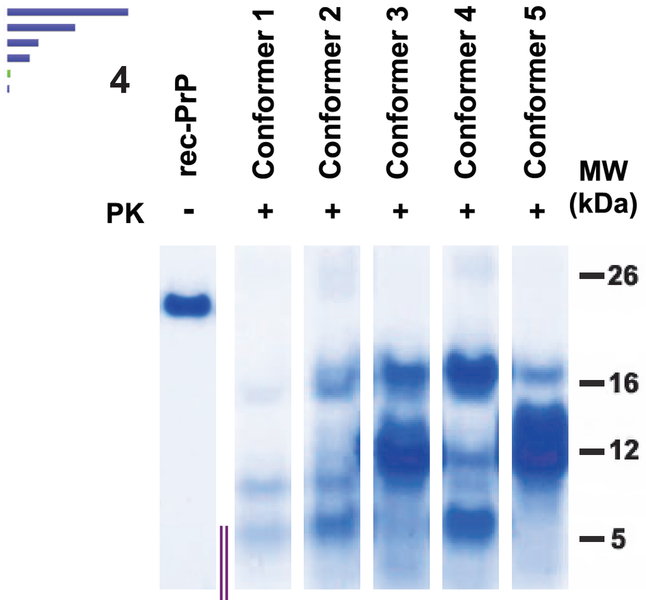

215 AA

5
Conformers

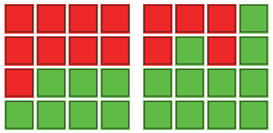

Tm^{Exp}: ND

ΔΔG: ND

In vitro studies

TgVole (1x)	Ongoing
Pangolin	Not tested

In vivo studies

TgVole (1x)	Ongoing
Pangolin	Not tested

pLDDT
[87.58]

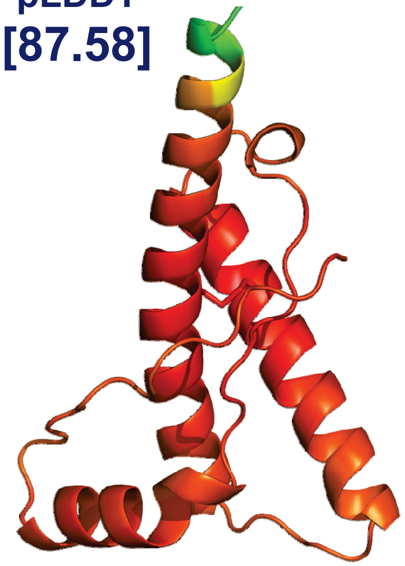

Protein code: 0942

Chinese pangolin

GenBank: BK063962

P56%

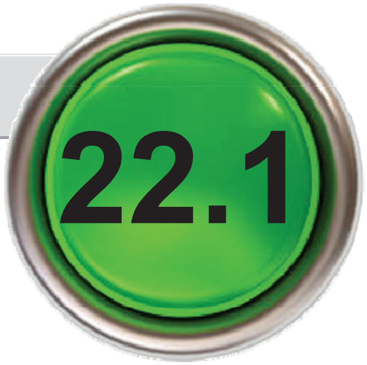

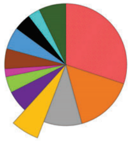

20-30

Manis pentadactyla

Pholidota	3/3
Manidae	3/3

β2-α2 LOOP
NNQNNF
164

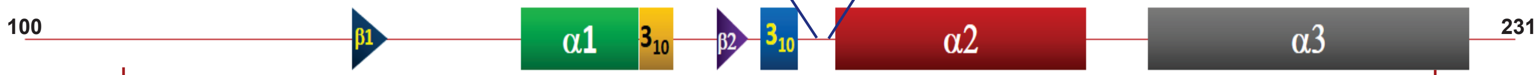

WGKPSKPKTNMKHVAGAAAAGAVVGGLGGYMLGSAMSRPLIHFGNDYEDRYYRENMYRYPNQVYYRPVDQYNNQNNFVHDCVNITVKQHTVTTTTKGENFTETDIKMMERVVEQMCITQYQKEYQATSYQRGAS-

N I M W N I V V S YYEG SS

PrP sequence differs by 8 amino acids from the bank vole PrP (see in red)

No deletions

No insertions

No polymorphic variants found

Distinct primary sequence across species

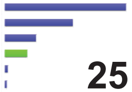

25

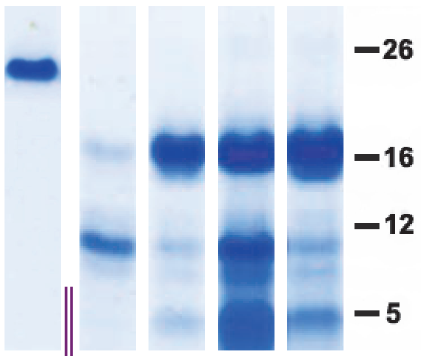

215 AA

4
Conformers

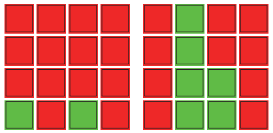

Tm^{Exp}: ND

ΔΔG: ND

In vitro studies

TgVole (1x)	Not tested
Chinese pangolin	Not tested

In vivo studies

TgVole (1x)	Not tested
Chinese pangolin	Not tested

pLDDT
[85.06]

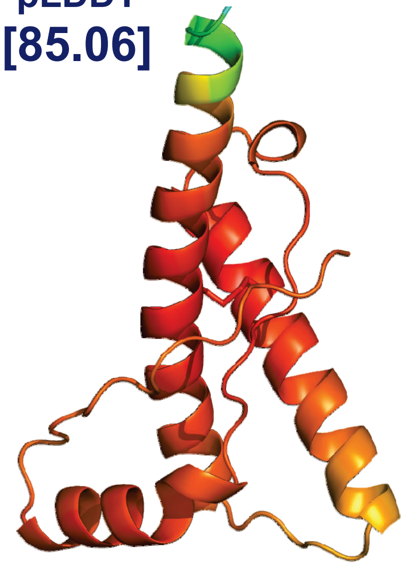

Protein code: 3092

Tree pangolin

GenBank: BK063960

Manis tricuspis

Pholidota 3/3

Manidae 3/3

P52%

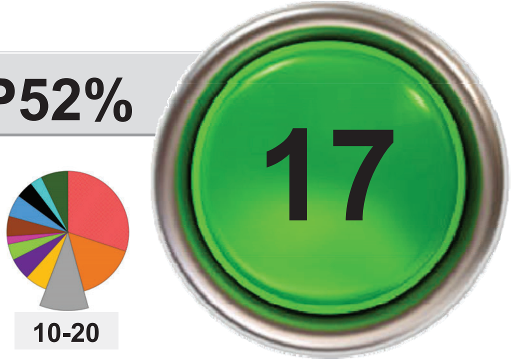

β2-α2 LOOP
SNQNNF
273

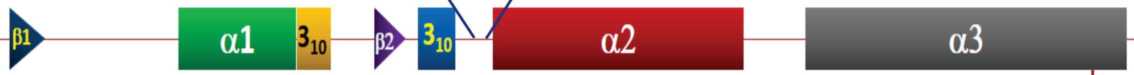


```
WGKPSKPKTNMKHVAGAAAAGAVVGGLGGYMLGSAMSRPLIHFGNDYEDRYYRENMYRYPNQVYYRPVDQYSNQNNFVHDCVNITVKQHTVTTTTKGENFTETDIKMMERVVEQMCVTQYQKEYQATSYQRGAS-
 N       I                          M      W         N               N         I           V                 S YYEG SS
```

PrP sequence differs by 8 amino acids from the bank vole PrP (see in red)

No deletions
No insertions
No polymorphic variants found

Distinct primary sequence across species

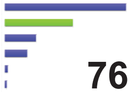

76

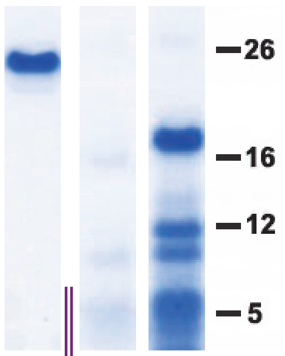

215 AA

2
Conformers

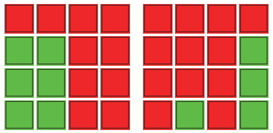

Tm^{Exp}: ND

ΔΔG: ND

In vitro studies

TgVole (1x) Not tested

Tree pangolin Not tested

In vivo studies

TgVole (1x) Not tested

Tree pangolin Not tested

pLDDT
[85.51]

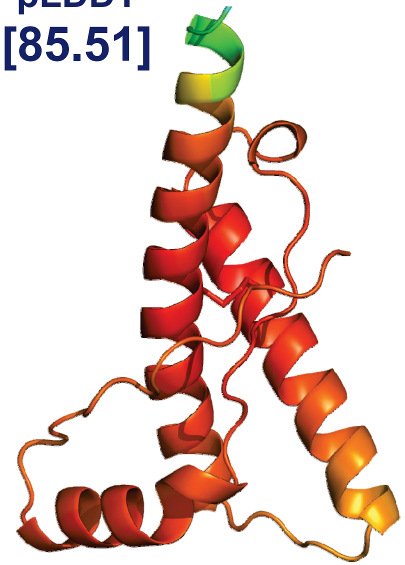

Protein code: 3089

Brown-throated sloth

GenBank: BK063934

Bradypus variegatus

Pilosa 1/5
Bradypodidae 0/1

P29%

0

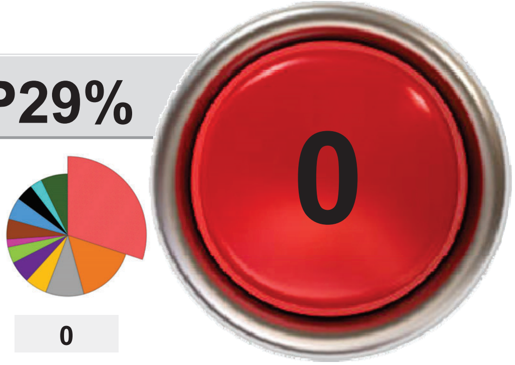

β2-α2 LOOP
SSENNF
8

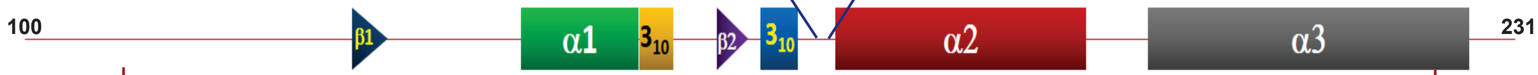

WNKPSKPKTNMKHVAGAAAAGAVVGGLGGYLVGSAMSRPLIHFGNDYEDRYYRENMYRYPNQVYYKPVDQYSSENNFVRDCVNITIKEHTVTTTTKGENFTETDIKMMERVVEQMCVTQYKTEYQAAYRQGSS-
I ML M W N R NNQ H Q V QK S Y EGR

PrP sequence differs by 16 amino acids from the bank vole PrP (see in red)

No deletions
No insertions
No polymorphic variants found

Tm[Exp]: ND

ΔΔG: -11.57

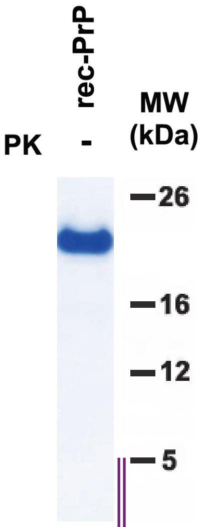

217 AA

NO MISFOLDING

0
Conformers

In vitro studies

NOT APPLICABLE

In vivo studies

NOT APPLICABLE

pLDDT
[85.17]

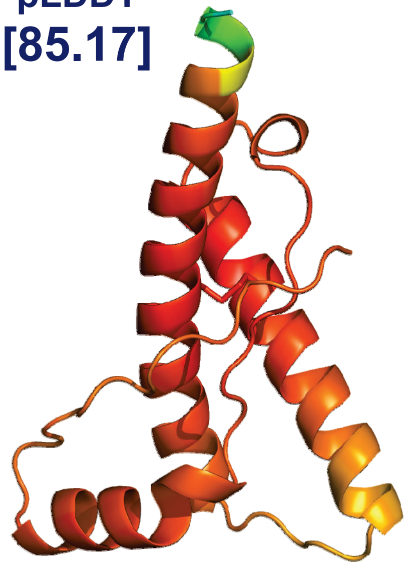

Protein code: 3057

Linnaeus's two-toed sloth

GenBank: BK063945

Choloepus didactylus

Pilosa 1/5
Megalonychidae 0/1

P29%

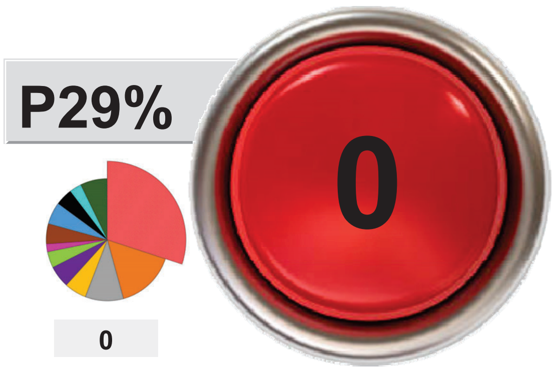

1 species sharing the same primary sequence

Hoffmann's two-toed sloth
Choloepus hoffmanni

β2-α2 LOOP
SSENNF
8

100 — β1 — α1 — 3_{10} — β2 — 3_{10} — α2 — α3 — 231

```
WNKPSKPKTNMKHVAGAAAAGAVVGGLGGYLVGSAMSRPLIHFGSDYEDRYYRENMYRYPNQVYYKPVDQYSSENNFVRDCVNITIKEHTVTTTTKGENFTETDIKMMERVVEQMCITQYKTEYQAAYQRGSS-
          I                ML        M    N W         N        R     NNQ   H        Q                 V           V   QK S  Y EGR
```

PrP sequence differs by 18 amino acids from the bank vole PrP (see in red)

No deletions
No insertions
No polymorphic variants found

Tm[Exp]: ND
ΔΔG: -14.14

PK | -
rec-PrP
MW (kDa)

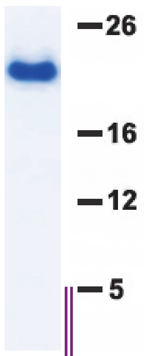

217 AA

NO MISFOLDING

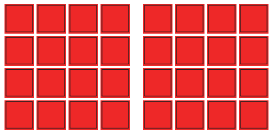

0
Conformers

In vitro studies

NOT APPLICABLE

In vivo studies

NOT APPLICABLE

pLDDT
[86.49]

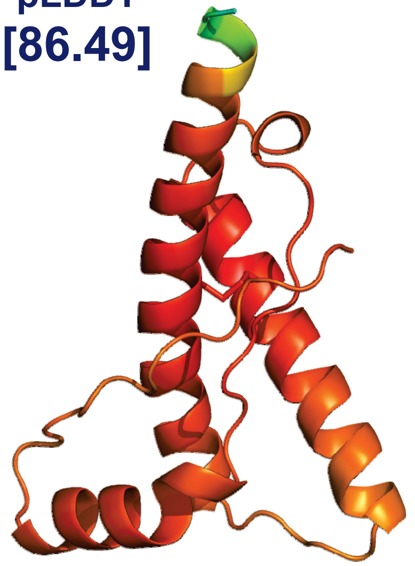

Protein code: 3071

Silky anteater

GenBank: AY133063

Cyclopes didactylus

Pilosa 1/5
Cyclopedidae 0/1

P29%

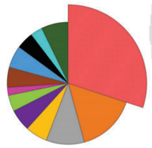

0

0

β2-α2 LOOP
SSENNF
8

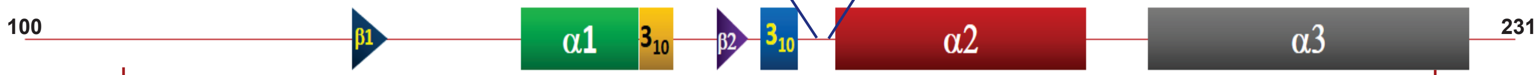

WNKPSKPKTNMKHVAGAAAAGAVVGGLGGYLVGSAMSRPLIHFGNDYEDRYYRENMYRYPNQVYYKPVNQYSSENNFVRDCVNITIKEHTVTTTTKGENFTETDVKMMERVVEQMCITQYKQESQAAYYQRGSS-
I ML M W N R D NNQ H Q V QK Y EG S

PrP sequence differs by 16 amino acids from the bank vole PrP (see in red)

No deletions
No insertions
No polymorphic variants found

Distinct primary sequence across species

Tm^{Exp}: ND

ΔΔG: ND

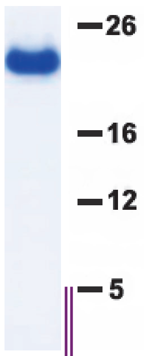

216 AA

NO MISFOLDING

0 Conformers

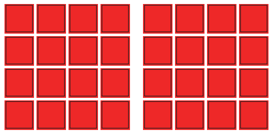

In vitro studies

NOT APPLICABLE

In vivo studies

NOT APPLICABLE

pLDDT [87.28]

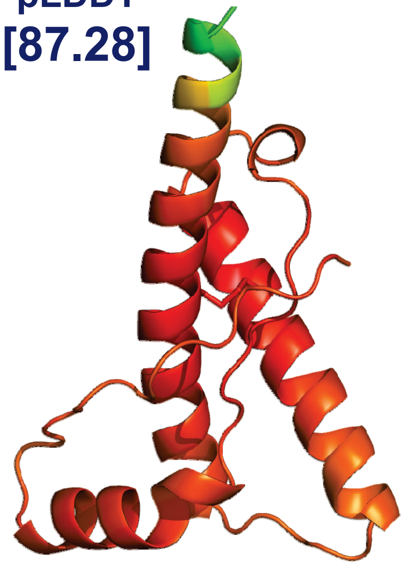

Protein code: 0923

Giant anteater

GenBank: BK063979

Myrmecophaga tridactyla

Pilosa 1/5

Myrmecophagidae 1/2

Distinct primary sequence across species

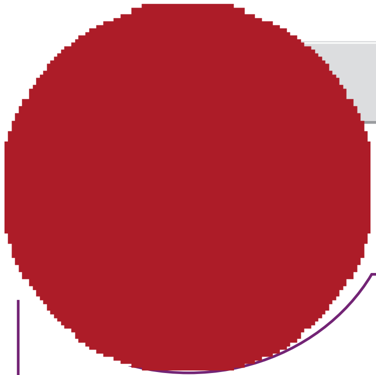

P29%

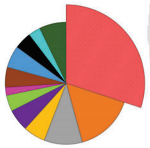

0

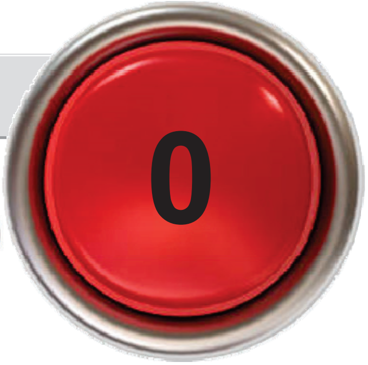

β2-α2 LOOP
SSENNF
8

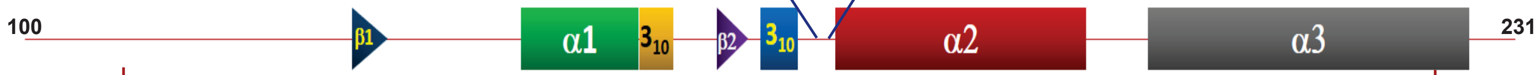

WNKPSKPKTNMKHMAGAAAAGAVVGGLGGYLVGSAMSRPLIHFGNDYEDRYYRENMYRYPNQVYYKPVNQYSSENNFVHDCVNITIKEHTVTTTTKGENFTETDIKMMERVVEQMCITQYQRESQAAYFHGGS-

I V ML M W N R D NNQ Q V V K Y EGRS

PrP sequence differs by 16 amino acids from the bank vole PrP (see in red)

No deletions

No insertions

No polymorphic variants found

Tm^{Exp}: ND

ΔΔG: -15.61

PK

rec-PrP

-

MW (kDa)

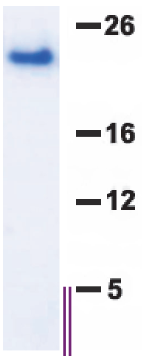

214 AA

NO MISFOLDING

0

Conformers

In vitro studies

NOT APPLICABLE

In vivo studies

NOT APPLICABLE

pLDDT

[86.38]

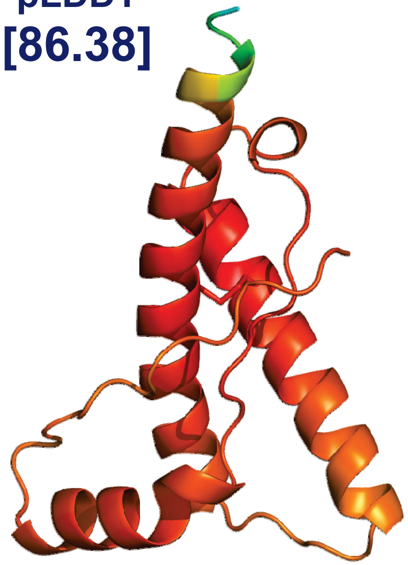

Protein code: 3136

Southern tamandua

GenBank: BK063980

Tamandua tetradactyla

Pilosa 1/5
Myrmecophagidae 1/2

P65%

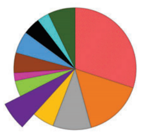

30-40

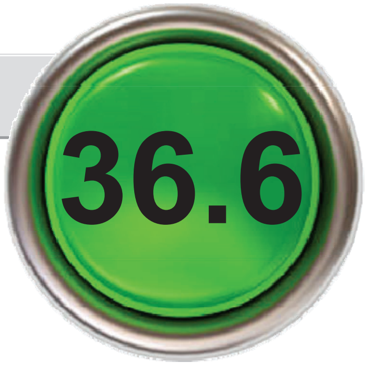

β2-α2 LOOP
SSENNF
8

100

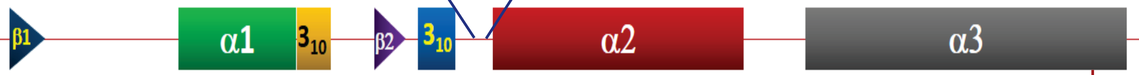

231

WNKPSKPKTNMKHVAGAAAAGAVVGGLGGYLVGSAMSRPLIHFGNDYEDRYYRENMYRYPNQVYYKPVNQYSSENNFVHDCVNITIKEHTVTTTTKGENFTETDIKMMERVVEQMCITQYQRESQAAYFHGGS-
I ML M W N R D NNQ Q V V K Y EGRS

PrP sequence differs by 15 amino acids from the bank vole PrP (see in red)

No deletions
No insertions
No polymorphic variants found

Distinct primary sequence across species

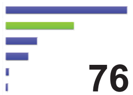

76

PK | rec-PrP - | Conformer 1 + | Conformer 2 + | MW (kDa)

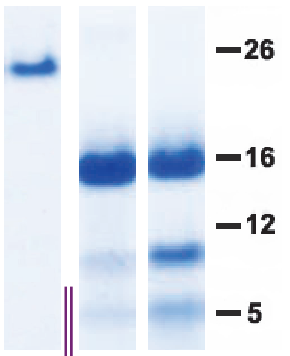

214 AA

2
Conformers

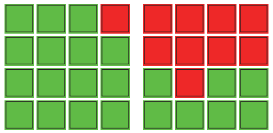

Tm^{Exp}: ND

ΔΔG: -10.89

In vitro studies

TgVole (1x) Not tested

Southern tamandua Not tested

In vivo studies

TgVole (1x) Not tested

Southern tamandua Not tested

pLDDT
[86.33]

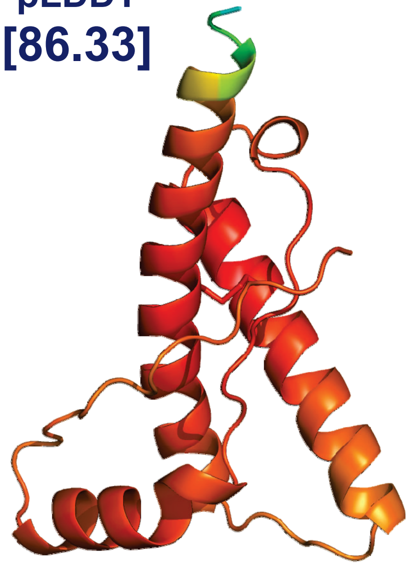

Protein code: 3137

Red-handed howler

GenBank: AY765382

P75%

63.6

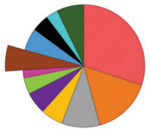

60-70

Alouatta belzebul

Primates	35/45
Atelidae	3/5

Mantled howler
Alouatta palliata

1 species sharing the same primary sequence

β2-α2 LOOP
SNQNNF
273

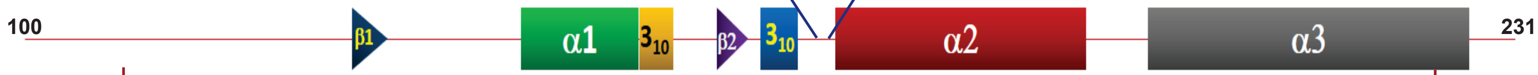

WNKPSKPKTNMKHMAGAAAAGAVVGGLGGYMLGSAMSRPLIHFGNDYEDRYYRENMYRYPNQVYYRPVDQYSNQNNFVHDCVNITIKQHTVTTTTKGENFTETDVKMMERVVEQMCITQYEKESQAYYQRGSS-

I V M W N N V Q EGR

PrP sequence differs by 8 amino acids from the bank vole PrP (see in red)

No deletions
No insertions
No polymorphic variants found

25

PK	rec-PrP	Conformer 1	Conformer 2	Conformer 3	Conformer 4	MW (kDa)
	-	+	+	+	+	

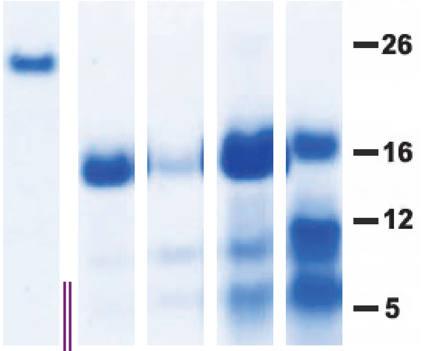

210 AA

4 Conformers

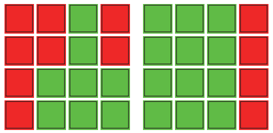

Tm^{Exp}: ND

ΔΔG: -0.92

***In vitro* studies**

TgVole (1x) — Not tested

Red-handed howler — Not tested

***In vivo* studies**

TgVole (1x) — Not tested

Red-handed howler — Not tested

pLDDT
[87.82]

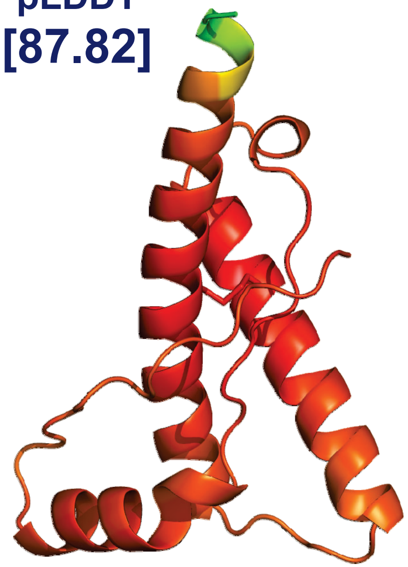

Protein code: 1383

Gray-bellied night monkey

GenBank: AY765387

Aotus lemurinus

Primrates 35/45
Aotidae 1/2

1 species sharing the same primary sequence

Nancy Ma's night monkey
Aotus nancymaae

P29%

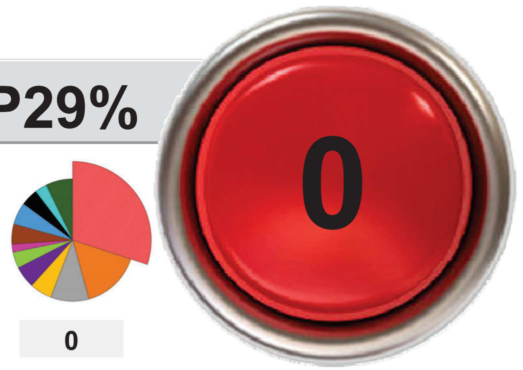

β2-α2 LOOP
SNQNNF
273

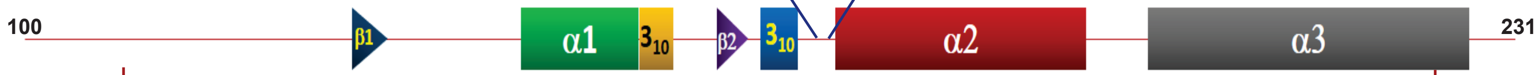

WNKPSKPKTNMKHMAGAAAAGAVVGGLGGYMLGSAMSRPLIHFGNDYEDRYYRENMYRYPNQVYYRPVDQYSNQNNFVHDCVNITIKQHTVTTTTKGENFTETDVKIMERVVEQMCITQYEKESQAYYQRGSS-
I V M W N N M V Q EGR

PrP sequence differs by 9 amino acids from the bank vole PrP (see in red)

No deletions
No insertions
No polymorphic variants found

Tm^{Exp}: ND
ΔΔG: 1.07

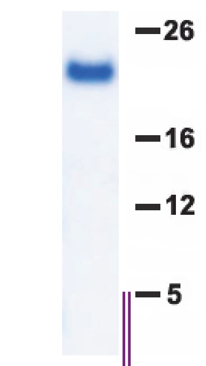

210 AA

NO MISFOLDING

0 Conformers

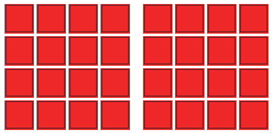

In vitro studies

NOT APPLICABLE

In vivo studies

NOT APPLICABLE

pLDDT
[84.39]

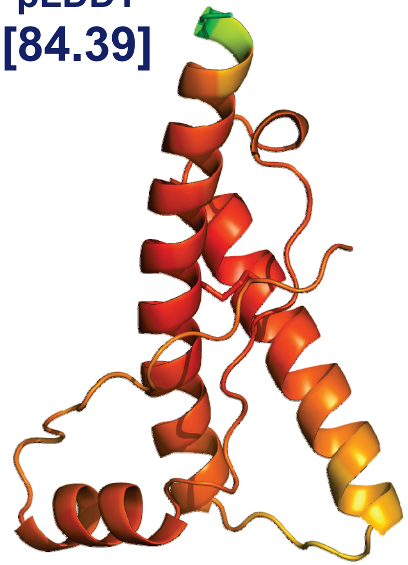

Protein code: 1386

Three-striped night monkey

GenBank: U08293

Aotus trivirgatus

Primates 35/45
Aotidae 1/2

P70% **46.1**

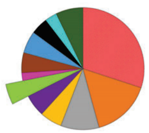

β2-α2 LOOP
SNQNNF
273

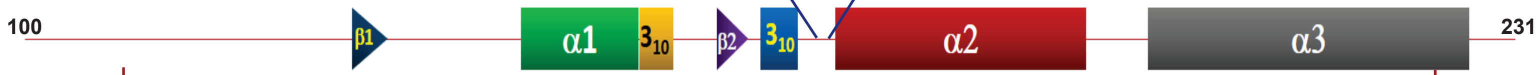

WNKPSKPKTNMKHMAGAAAAGAVVGGLGGYMLGSAMSRPLIHFGNDYEDRYYRENMYRYPNQVYYRPVDQYSNQNNFVHDCVNITIKQHTVTTTTKGENFTETDVKIMERVVEQMCITQYEKESQAYYQRGSS-
I V M W N N M V Q EGR

PrP sequence differs by 9 amino acids from the bank vole PrP (see in red)

No deletions
No insertions
No polymorphic variants found

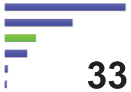

33

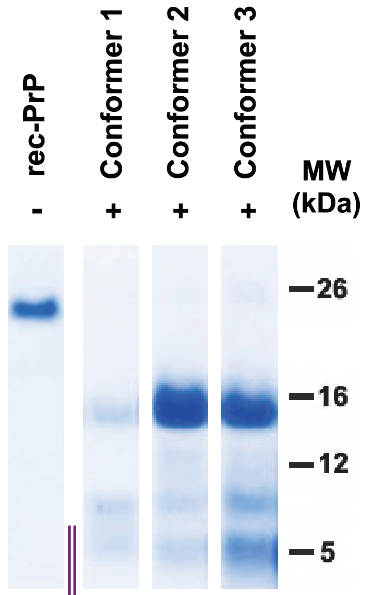

210 AA

3 Conformers

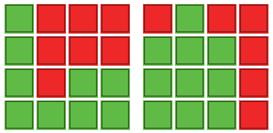

Tm[Exp]: ND
ΔΔG: -5.4

In vitro studies

Not tested
TgVole (1x)

Not tested
Three-striped night monkey

In vivo studies

Not tested
TgVole (1x)

Not tested
Three-striped night monkey

pLDDT
[86.79]

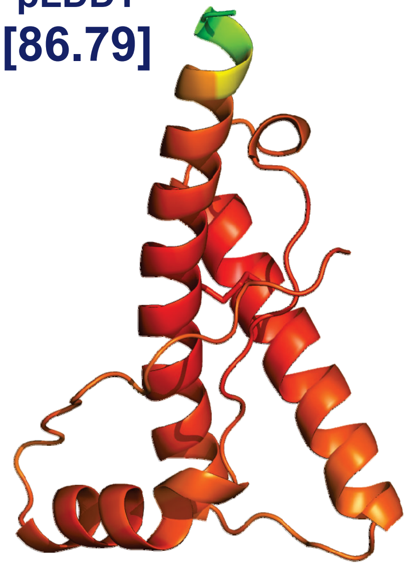

Distinct primary sequence across species

Protein code: 1385

Geoffroy's spider monkey

GenBank: U08309

P56%

21.4

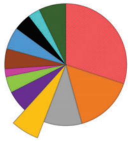

20-30

Ateles geoffroyi

Primates 35/45
Atelidae 3/5

1 species sharing the same primary sequence

Brown spider monkey
Ateles hybridus

β2-α2 LOOP
NNQNNF
164

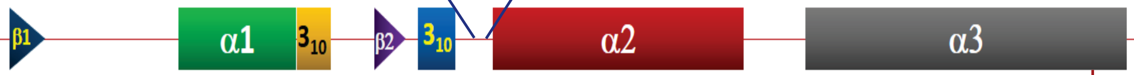

WNKPSKPKTNMKHMAGAAAAGAVVGGLGGYMLGSAMSRPLIHFGNDYEDRYYRENMYRYPNQVYYRPVDQYNNQNNFVHDCVNITIKQHTVTTTTKGENFTETDVKMMERVVEQMCITQYERESQAYYQRGSS-

I V M W N V QK EGR

PrP sequence differs by 8 amino acids from the bank vole PrP (see in red)

No deletions
No insertions
No polymorphic variants found

135

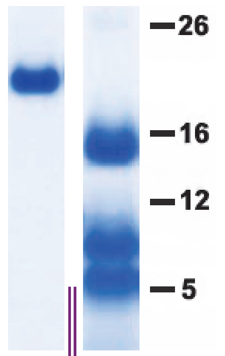

202 AA

1
Conformers

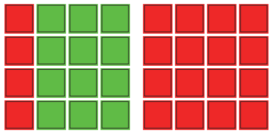

Tm[Exp]: ND

ΔΔG: -6.77

pLDDT
[85.45]

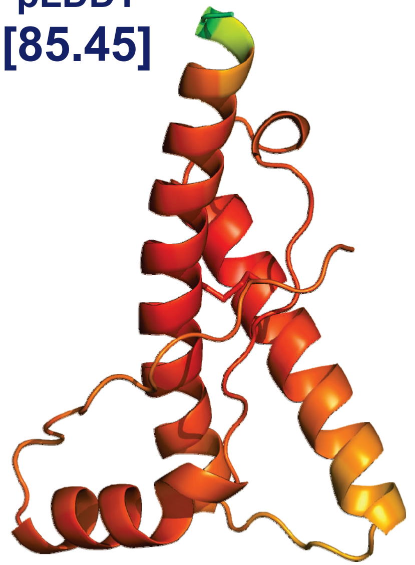

In vitro studies

TgVole (1x) — Not tested

Geoffroy's spider monkey — Not tested

In vivo studies

TgVole (1x) — Not tested

Geoffroy's spider monkey — Not tested

Protein code: 1417

Red-faced spider monkey

GenBank: U15164

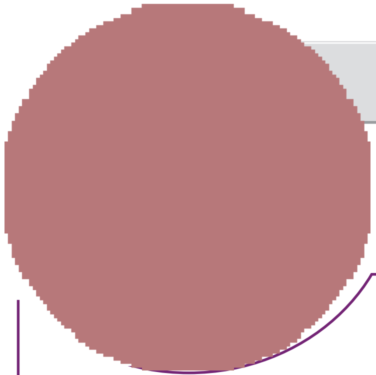

P29%

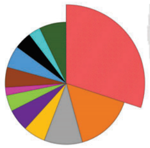

0

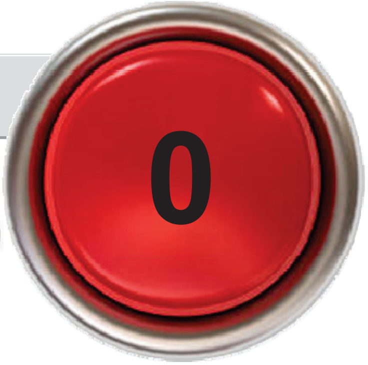

Ateles paniscus

Primates 35/45
Atelidae 3/5

β2-α2 LOOP
NNQNNF
164

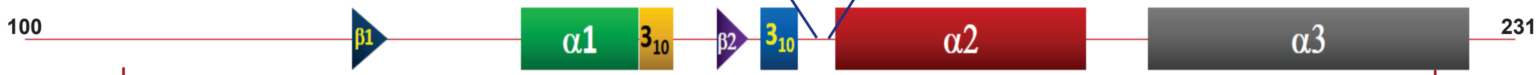

WNKPSKPKTNMKHMAGAAAAGAVVGGLGGYMLGSAMSRPLIHFGNDYEDRYYRENMYRYPNQVYYRPVDQYNNQNNFVHDCVNITIKQHTVTTTTKGENLTETDVKMMERVVEQMCITQYERESQAYYQRGSS-

I V M W N F V QK EGR

PrP sequence differs by 9 amino acids from the bank vole PrP (see in red)

No deletions
No insertions
No polymorphic variants found

Distinct primary sequence across species

Tm^{Exp}: ND

ΔΔG: -5.68

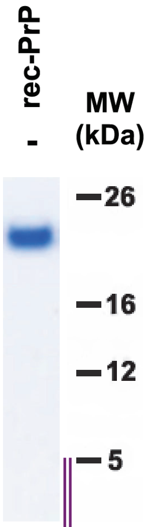

210 AA

NO MISFOLDING

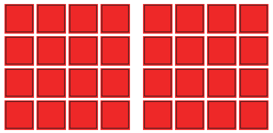

0
Conformers

In vitro studies

NOT APPLICABLE

In vivo studies

NOT APPLICABLE

pLDDT
[86.16]

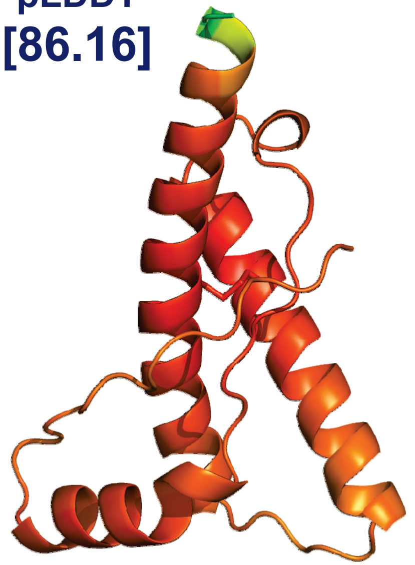

Protein code: 1415

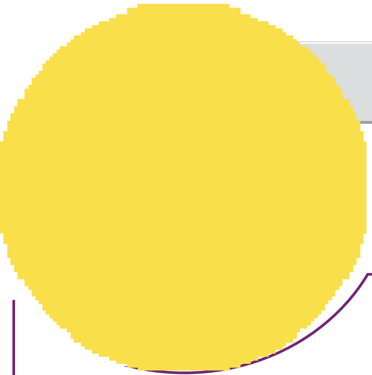

Southern muriqui

GenBank: AY765383

Brachyteles arachnoides

Primates	35/45
Atelidae	3/5

P29%

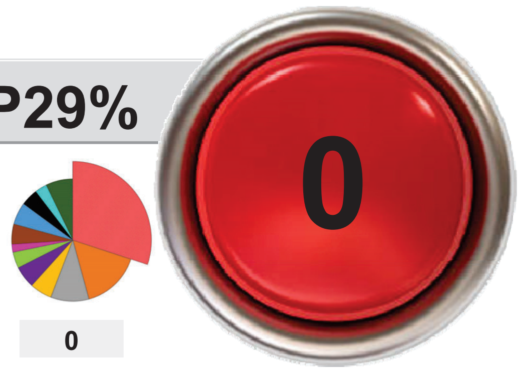

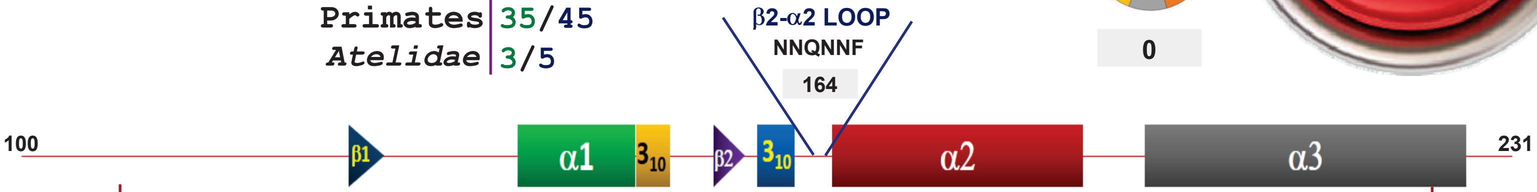

WSKPSKPKTNMKHMAGAAAAGAVVGGLGGYMLGSAMSRPLIHFGNDYEDRYYRENMYRYPNQVYYRPVDQYNNQNNFVHDCVNITIKQHTVTTTTKGENFTETDVKMMERVVEQMCITQYEKESQAYYQRGSS-

N I V M W N V Q EGR

PrP sequence differs by 7 amino acids from the bank vole PrP (see in red)

No deletions

No insertions

No polymorphic variants found

Distinct primary sequence across species

Tm^{Exp}: ND

ΔΔG: -4.43

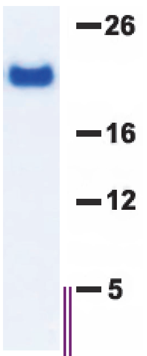

210 AA

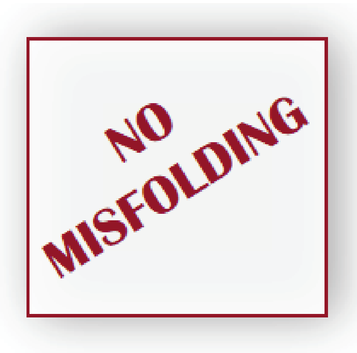

0

Conformers

In vitro studies

NOT APPLICABLE

In vivo studies

NOT APPLICABLE

pLDDT

[85.32]

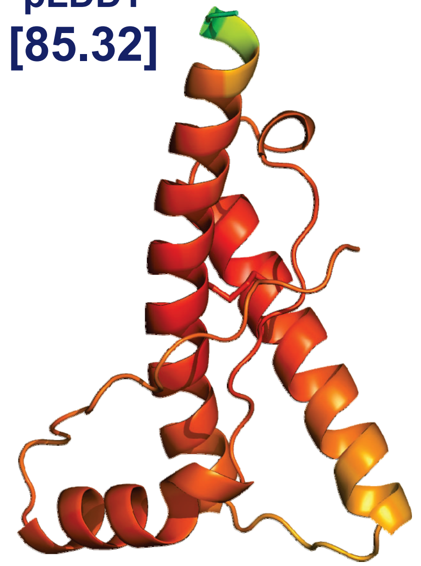

Protein code: 1337

White-eared titi

GenBank: BK064142

Callicebus donacophilus

Primates 35/45
Pitheciidae 3/4

P82%

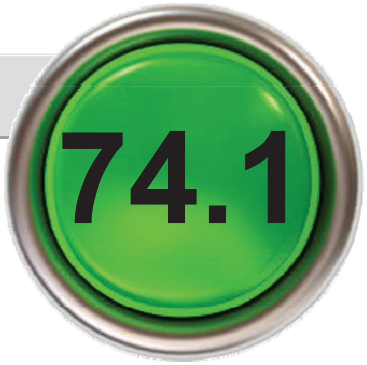

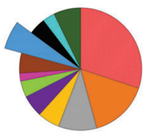

70-80

β2-α2 LOOP
SNQNNF
273

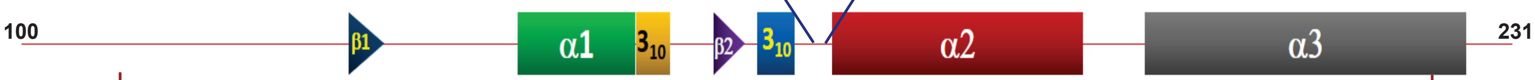

WNKPSKPKTNMKHVAGAAAAGAVVGGLGGYMLGSAMSRPLIHFGNDYEDRYYRENMYRYPNQVYYRPVDQYSNQNNFVHDCVNITIKQHTVTTTTKGDNFTETDVKMMERVVEQMCITQYEKESQAYYQRGSS-
I M W N N E V Q EGR

PrP sequence differs by 8 amino acids from the bank vole PrP (see in red)

No deletions
No insertions
No polymorphic variants found

Distinct primary sequence across species

25

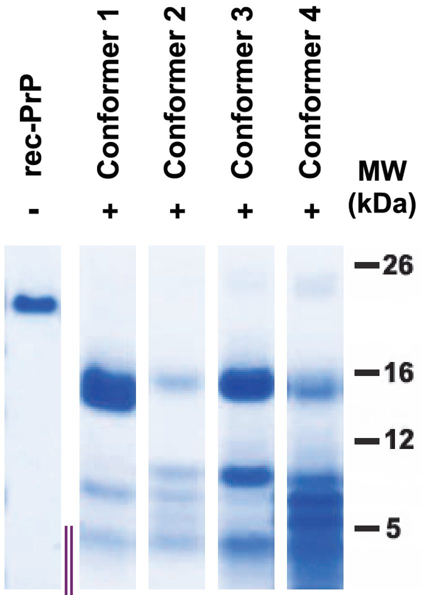

211 AA

4 Conformers

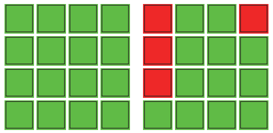

Tm^{Exp}: ND

ΔΔG: 4.73

In vitro studies

TgVole (1x) Not tested

White-eared titi Not tested

In vivo studies

TgVole (1x) Not tested

White-eared titi Not tested

pLDDT [84.42]

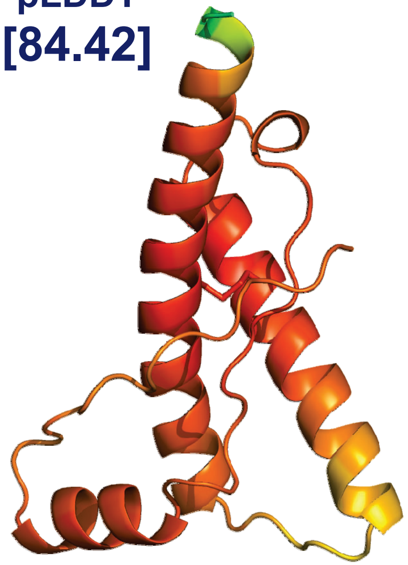

Protein code: 3329

Red-bellied titi

GenBank: U08312

Callicebus moloch

Primates 35/45
Pitheciidae 3/4

P76%

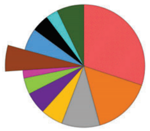

60-70

64.3

β2-α2 LOOP
SNQNNF
273

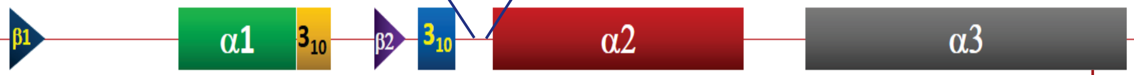

WNKPSKPKTNMKHVAGAAAAGAVVGGLGGYMLGSAMSRPLIHFGNDYEDRYYRENMYRYPNQVYYRPVDQYSNQNNFVHDCVNITIKQHTVTTTTKGENFTETDVKMMERVVEQMCITQYEKESQAYYQRGSS-
I M W N N V Q EGR

PrP sequence differs by 7 amino acids from the bank vole PrP (see in red)

No deletions
No insertions
No polymorphic variants found

Distinct primary sequence across species

2

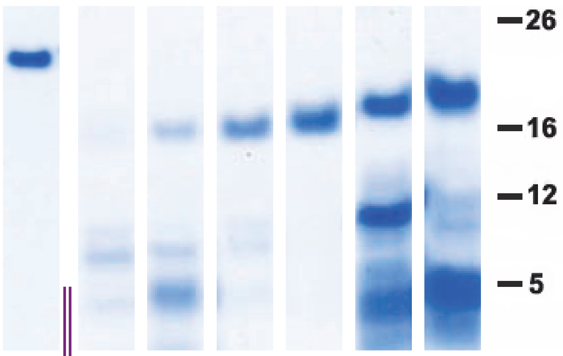

211 AA

6 Conformers

Tm^{Exp}: ND

ΔΔG: 3.98

In vitro studies

TgVole (1x) Not tested

Red-bellied titi Not tested

In vivo studies

TgVole (1x) Not tested

Red-bellied titi Not tested

pLDDT [85.07]

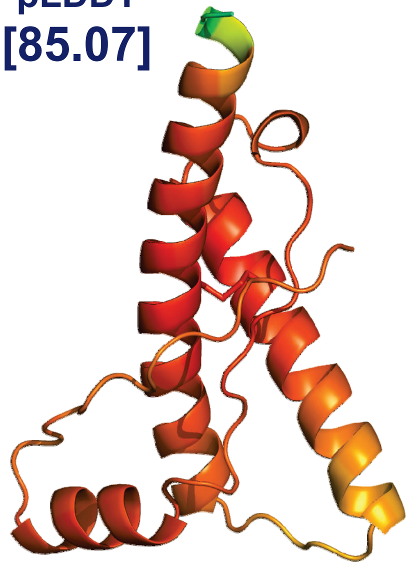

Common marmoset

GenBank: XM_008995703

Callithrix jacchus

Primates 35/45
Cebidae 7/8

P77%

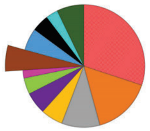

67.5

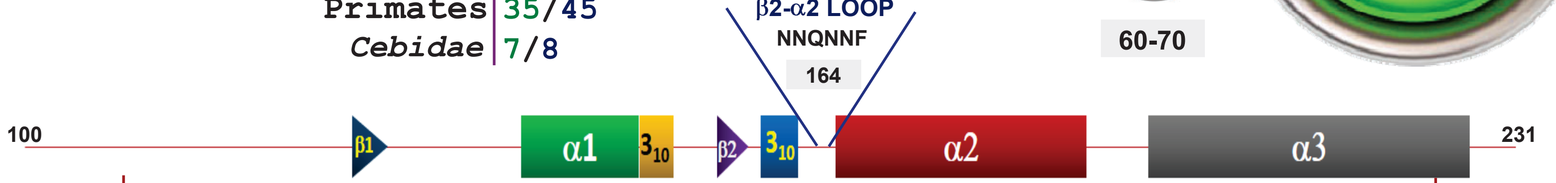

WNKPSKPKTNMKHVAGAAAAGAVVGGLGGYMLGSAMSRPLIHFGNDYEDRYYRENMYRYPNQVYYRPVDQYNNQNNFVHDCVNITIKQHTVTTTTKGENFTETDVKMMERVVEQMCITQYERESQAYYQRGSS-
I M W N V QK EGR

PrP sequence differs by 7 amino acids from the bank vole PrP (see in red)

No deletions
No insertions
No polymorphic variants found

4 species sharing the same primary sequence

Goeldi's marmoset *Callimico goeldii*
Silvery marmoset *Callithrix argentata*
White-headed marmoset *Callithrix geoffroyi*
Pygmy marmoset *Callithrix pygmaea*

135

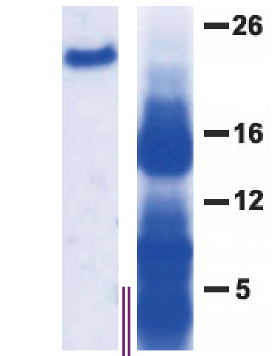

210 AA

1 Conformers

Tm^{Exp}: ND
ΔΔG: -4.16

In vitro studies

TgVole (1x) Not tested
Common marmoset Not tested

In vivo studies

TgVole (1x) Not tested
Common marmoset Not tested

pLDDT [85.76]

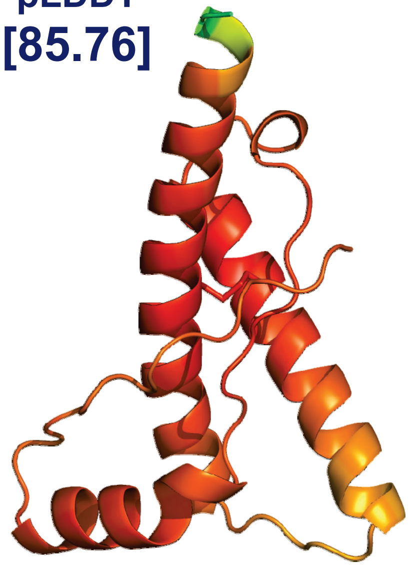

Protein code: 1121

Panamanian white-faced capuchin

GenBank: XM_017524036

Cebus capucinus

P39%

3.9

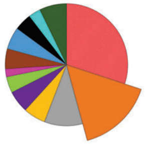

0-10

2 species sharing the same primary sequence

H white-fronted capuchin
Cebus albifrons

Tufted capuchin
Cebus apella

Primates 35/45
Cebidae 7/8

β2-α2 LOOP
SNQNNF
273

100 β1 α1 3_{10} β2 3_{10} α2 α3 231

WNKPSKPKTSMKHVAGAAAAGAVVGGLGGYMLGSAMSRPLIHFGNDYEDRYYRENMYRYPNQVYYRPVDQYSNQNNFVHDCVNITIKQHTVTTTTKGENFTETDVKMMERVVEQMCITQYERESQAYYQRGSS-

NI M W N N V QK EGR

PrP sequence differs by 9 amino acids from the bank vole PrP (see in red)

No deletions
No insertions
No polymorphic variants found

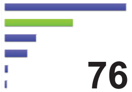

76

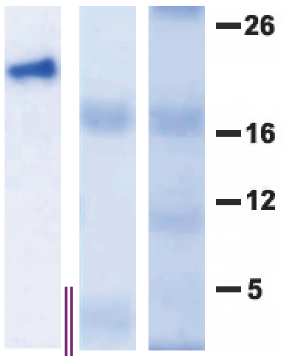

210 AA

2
Conformers

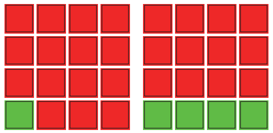

Tm^{Exp}: ND

ΔΔG: -2.68

In vitro studies

TgVole (1x) Not tested

Panamanian white-faced Not tested

In vivo studies

TgVole (1x) Not tested

Panamanian white-faced Not tested

pLDDT
[86.8]

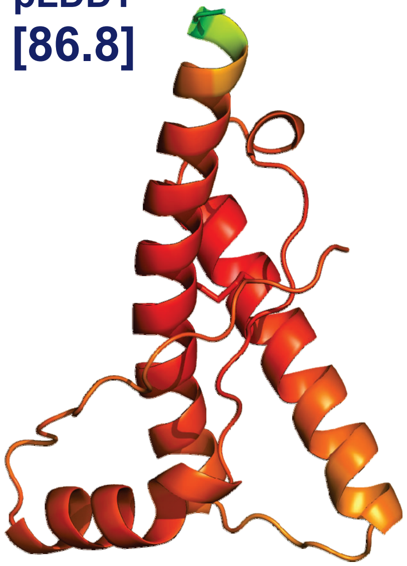

Protein code: 1084

Sooty mangabey

GenBank: XM_012052159

Cercocebus atys

Primates 35/45
Cercopithecidae 7/7

P45%

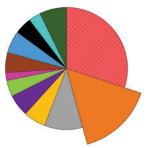

0-10

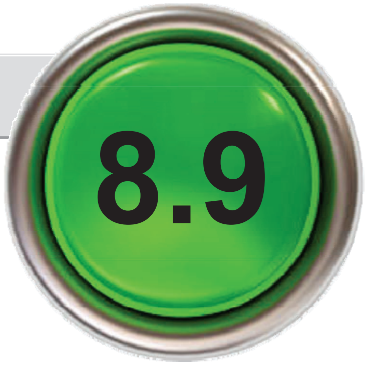

1 species sharing the same primary sequence

Collared mangabey
Cercocebus torquatus

β2-α2 LOOP
SNQNNF
273

100
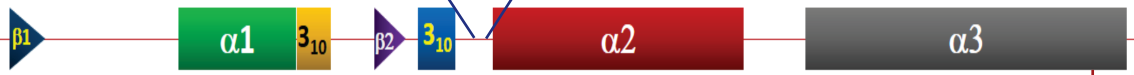

231

WHKPSKPKTSMKHMAGAAAAGAVVGGLGGYMLGSAMSRPLIHFGNEYEDRYYRENMYRYPNQVYYRPVDQYSNQNNFVHDCVNITIKQHTVTTTTKGENFTETDVKMMERVVEQMCITQYEKESQAYYQRGSS-

N NI V M DW N N V Q EGR

PrP sequence differs by 10 amino acids from the bank vole PrP (see in red)

No deletions
No insertions
No polymorphic variants found

135

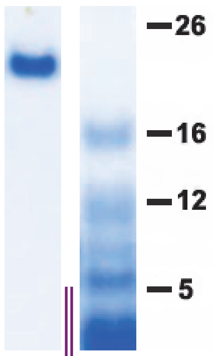

211 AA

1
Conformers

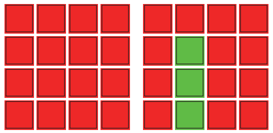

Tm^{Exp}: ND

ΔΔG: -9.39

In vitro studies

TgVole (1x) Not tested

Sooty mangabey Not tested

In vivo studies

TgVole (1x) Not tested

Sooty mangabey Not tested

pLDDT
[85.75]

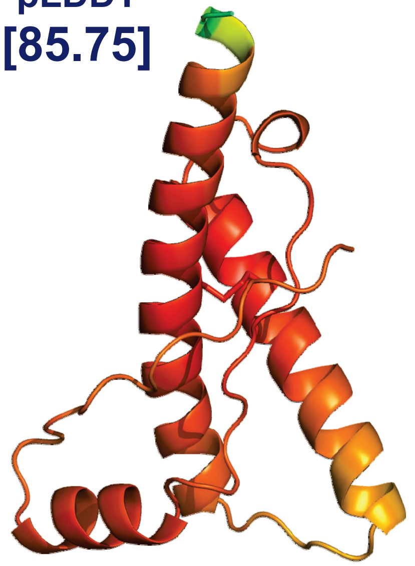

Protein code: 2057

Fat-tailed dwarf lemur

GenBank: BK063936

P29%

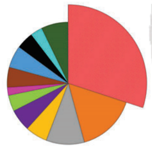

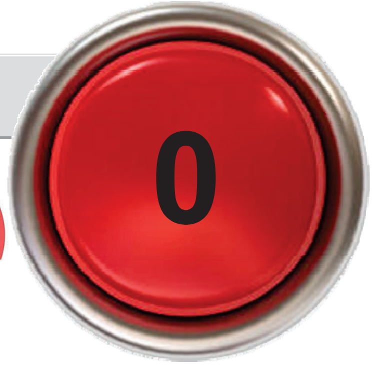

Cheirogaleus medius

Primates	35/45
Cheirogaleidae	1/4

Distinct primary sequence across species

β2-α2 LOOP
SNQNNF
273

100 — β1 — α1 — 3_{10} — β2 — 3_{10} — α2 — α3 — 231

```
WNKPSKPKTNMKHVAGAAAAGAVVGGLGGYMLGSAMSRPLMHFGNDYEDRYYRENMNRYPNQVYYKPVDQYSNQNNFVHDCVNITIKQHTVTTTTKGENFTETDVKMMERVVEQMCVTQYQKEYQAAYQRGSS-
           I                               MI     W                        R     N                                                              S   Y EGR
```

PrP sequence differs by 7 amino acids from the bank vole PrP (see in red)

No deletions
No insertions
No polymorphic variants found

Tm[Exp]: ND

ΔΔG: 10.05

PK | rec-PrP -

MW (kDa)

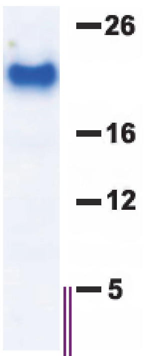

212 AA

NO MISFOLDING

0 Conformers

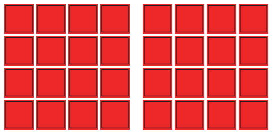

In vitro studies

NOT APPLICABLE

In vivo studies

NOT APPLICABLE

pLDDT [84.91]

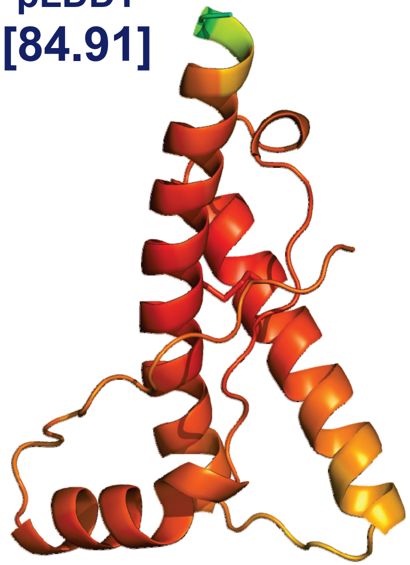

Protein code: 3059

Black bearded saki

GenBank: AY765380

Chiropotes satanas

Primates	35/45
Pitheciidae	3/4

P31%

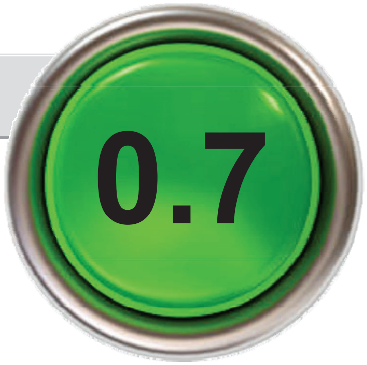

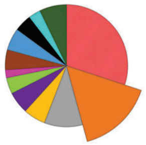

0-10

1 species sharing the same primary sequence

Bald uakari
Cacajao calvus

β2-α2 LOOP
SNQNNF
273

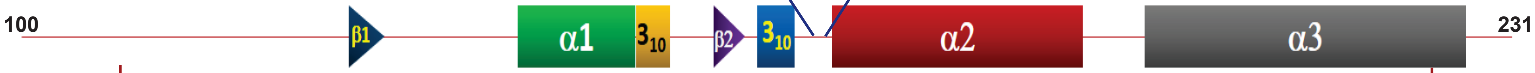

WNKPSKPKTNMKHMAGAAAAGAVVGGLGGYMLGSAMSRPLIHFGNDYEDRYYRENMYRYPNQVYYRPVDQYSNQNNFVHDCVNITIKQHTVTTTTKGENFTETDVKMMERVVEQMCITQYEKESQAYYQRGSS-

I V M W N N V Q EGR

PrP sequence differs by 8 amino acids from the bank vole PrP (see in red)

No deletions
No insertions
No polymorphic variants found

135

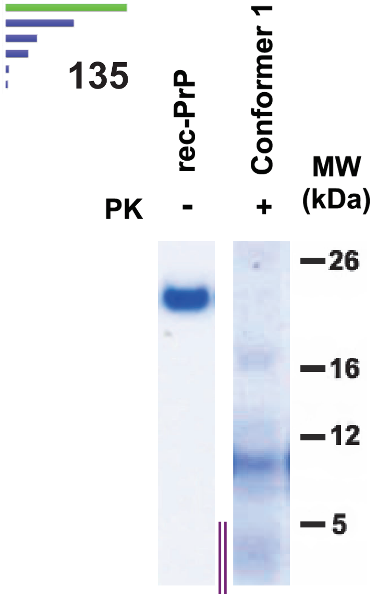

211 AA

1
Conformers

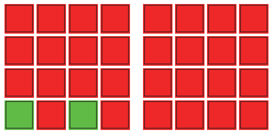

Tm[Exp]: ND

ΔΔG: -3.52

In vitro studies

TgVole (1x) — Not tested

Black bearded saki — Not tested

In vivo studies

TgVole (1x) — Not tested

Black bearded saki — Not tested

pLDDT
[85.14]

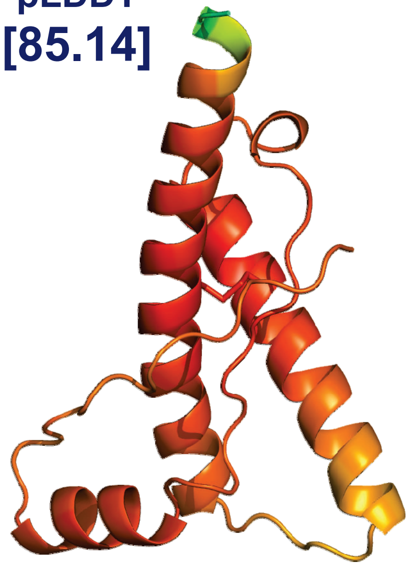

Angola colobus

GenBank: XM_011941048

Colobus angolensis palliatus

Primates 35/45
Cercopithecidae 7/7

11 species sharing the same primary sequence

- **Black snub-nosed monkey** *Rhinopithecus bieti*
- **Angola colobus** *Colobus angolensis palliatus*
- **Mantled guereza** *Colobus guereza*
- **Francois' langur** *Trachypithecus francoisi*
- **Golden snub-nosed monkey** *Rhinopithecus roxellana*
- **Northern plains gray langur** *Semnopithecus entellus*
- **Proboscis monkey** *Nasalis larvatus*
- **Red-shanked douc** *Pygathrix nemaeus*
- **Phayre's leaf monkey** *Trachypithecus phayrei crepuscula*
- **Black-shanked douc** *Pygathrix nigripes*
- **Myanmar snub-nosed monkey** *Rhinopithecus strykeri*

P42%

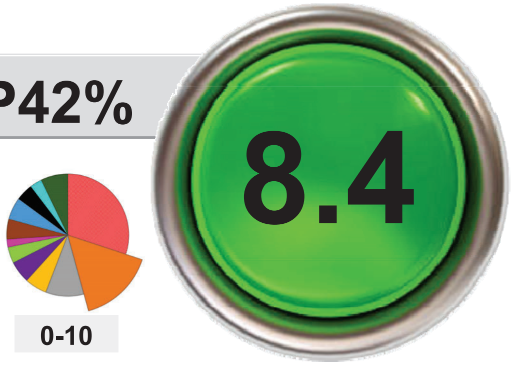

β2-α2 LOOP
SNQNNF
273

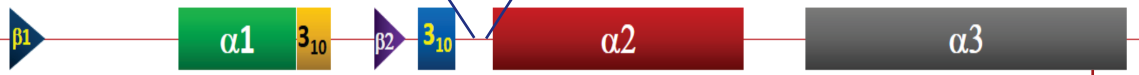


```
WNKPSKPKTSMKHMAGAAAAGAVVGGLGGYMLGSAMSRPLIHFGNDYEDRYYRENMYRYPNQVYYRPVDQYSNQNNFVHDCVNITIKQHTVTTTTKGENFTETDVKMMERVVEQMCITQYEKESQAYYQRGSS-
          NI  V                            M      W         N               N                                         V   Q              EGR
```

PrP sequence differs by 9 amino acids from the bank vole PrP (see in red)

No deletions
No insertions
No polymorphic variants found

76

211 AA

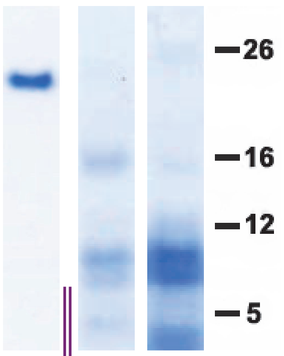

2 Conformers

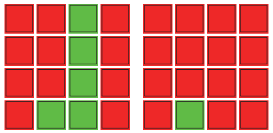

Tm^{Exp}: ND

ΔΔG: -1.54

In vitro studies

TgVole (1x) — Not tested

Angola colobus — Not tested

In vivo studies

TgVole (1x) — Not tested

Angola colobus — Not tested

pLDDT [86.04]

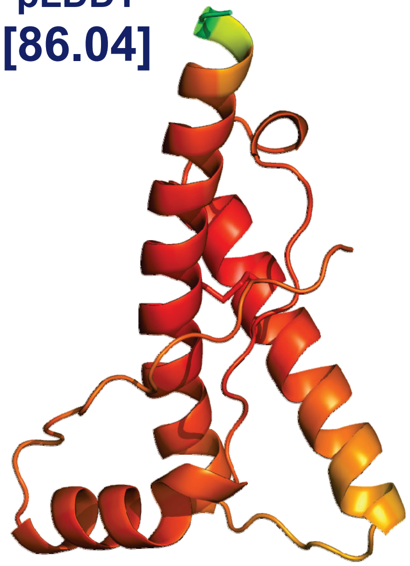

Aye-aye

GenBank: BK064012

Daubentonia madagascariensis

Primates 35/45
Daubentoniidae 1/1

Distinct primary sequence across species

P63%

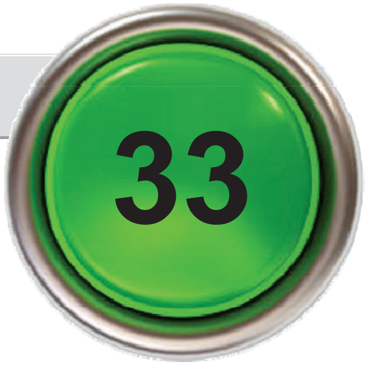

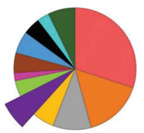

30-40

β2-α2 LOOP
SSQNSF
6

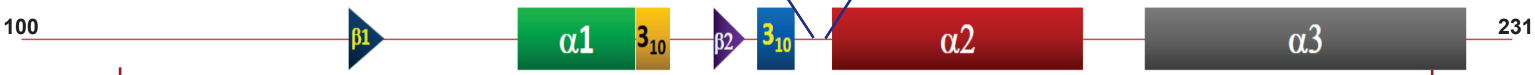

WNKPSKPKTNMKHVAGAAAAGAVVGGLGGYMLGSAMSRPLIHFGNDYEDRYYRENMYRYPNQVYYKPVDQYSSQNSFVHDCVNITIKQHTVTTTTKGENFTETDVKMMERVVEQMCVTQYQREYEAAAYQRGSS-
I M W N R NN N K SQ YYEG S

PrP sequence differs by 11 amino acids from the bank vole PrP (see in red)

No deletions
No insertions
No polymorphic variants found

33

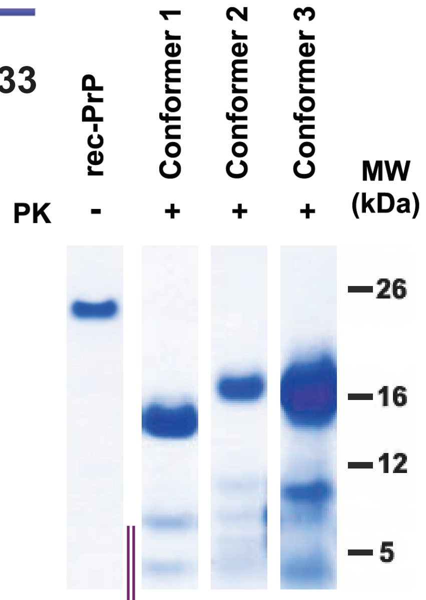

213 AA

3
Conformers

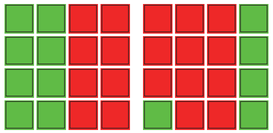

Tm^{Exp}: ND

ΔΔG: ND

pLDDT
[86.65]

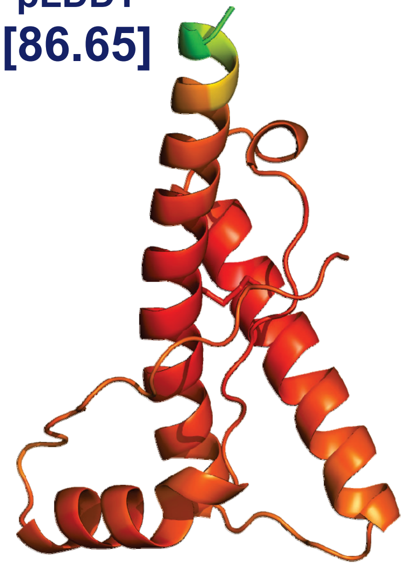

In vitro studies

TgVole (1x) Not tested

Aye-aye Not tested

In vivo studies

TgVole (1x) Not tested

Aye-aye Not tested

Protein code: 3173

Mohol bushbaby

GenBank: BK064194

Galago moholi

Primates 35/45

Galagidae 1/2

P29%

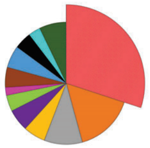

0

0

β2-α2 LOOP
SNQNNF
273

100 — β1 — α1 — 3_{10} — β2 — 3_{10} — α2 — α3 — 231

```
WNKPSKPKTNMKHVAGAAAAGAVVGGLGGYMLGSAMSRPLMHFGNDYEDRYYRENMNRYPSQVYYYRPVDQYSNQNNFVHDCVNITIKQHTVTTTTKGENFTETDVKIMERMVEQMCVTQYQREYQAAYQRGSS-
           I                            MI    W             N          N                                   M   V           K S  Y EGR
```

PrP sequence differs by 10 amino acids from the bank vole PrP (see in red)

No deletions

No insertions

No polymorphic variants found

Distinct primary sequence across species

Tm^{Exp}: ND

ΔΔG: -7.11

PK rec-PrP -

MW (kDa)

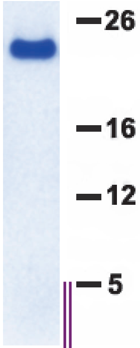

217 AA

NO MISFOLDING

0 Conformers

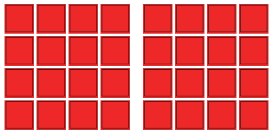

In vitro studies

NOT APPLICABLE

In vivo studies

NOT APPLICABLE

pLDDT [84.35]

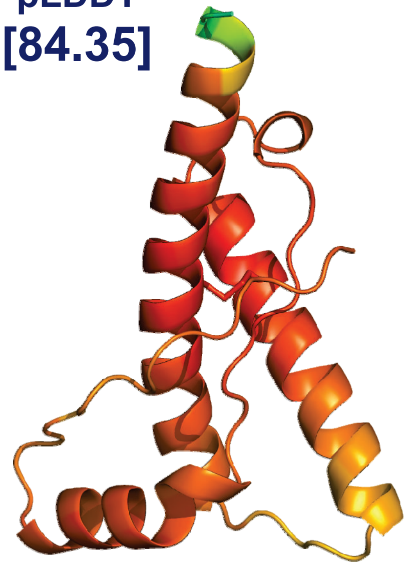

Protein code: 4523

Western gorilla

GenBank: U08300

Gorilla gorilla

Primates 35/45
Hominidae 4/4

P51%

14.3

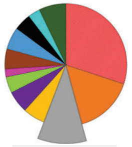

10-20

β2-α2 LOOP
SNQNNF
273

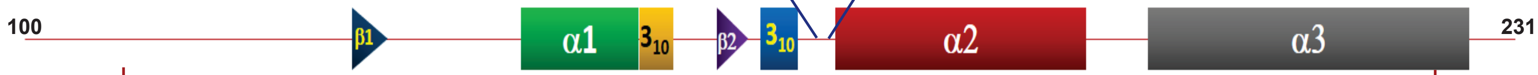

WNKPSKPKTNMKHMAGAAAAGAVVGGLGGYMLGSAMSRPIIHFGSDYEDRYYRENMHRYPNQVYYRPMDQYSNQNNFVHDCVNITIKQHTVTTTTKGENFTETDVKMMERVVEQMCITQYERESQAYYQRGSS-
I V M N W N V N V QK EGR

PrP sequence differs by 11 amino acids from the bank vole PrP (see in red)

No deletions
No insertions
No polymorphic variants found

Distinct primary sequence across species

135

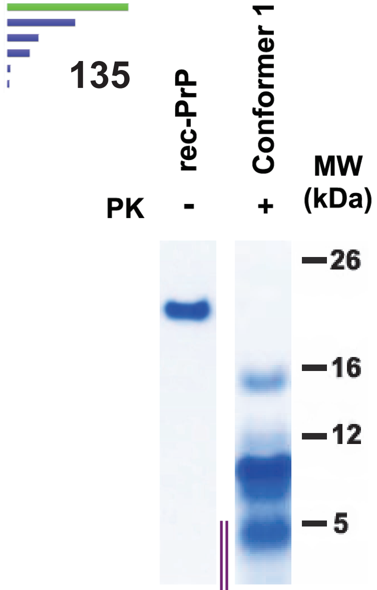

211 AA

1
Conformers

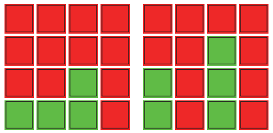

Tm^{Exp}: ND

ΔΔG: -5.87

In vitro studies

Not tested
TgVole (1x)

Not tested
Western gorilla

In vivo studies

Not tested
TgVole (1x)

Not tested
Western gorilla

pLDDT
[85.92]

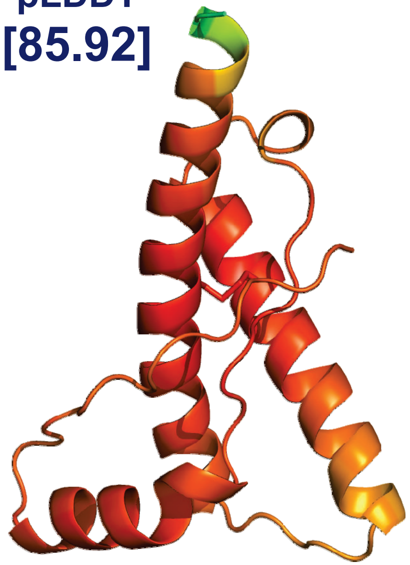

Protein code: 1148

Human

GenBank: NM_001080123

P61% **30.4**

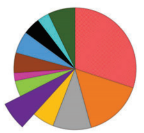

30-40

Homo sapiens

Primates 35/45
Hominidae 4/4

β2-α2 LOOP
SNQNNF
273

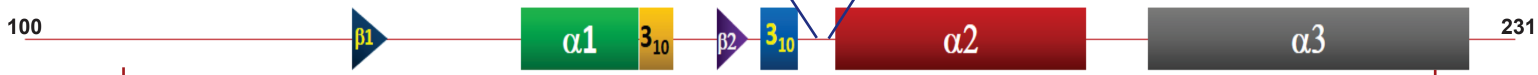

WNKPSKPKTNMKHMAGAAAAGAVVGGLGGYMLGSAMSRPIIHFGSDYEDRYYRENMHRYPNQVYYRPMDEYSNQNNFVHDCVNITIKQHTVTTTTKGENFTETDVKMMERVVEQMCITQYERESQAYYQRGSS-
I V M N W N V Q N V QK EGR

PrP sequence differs by 12 amino acids from the bank vole PrP (see in red)

No deletions
No insertions
It shows polymorphic variants

Distinct primary sequence across species

76

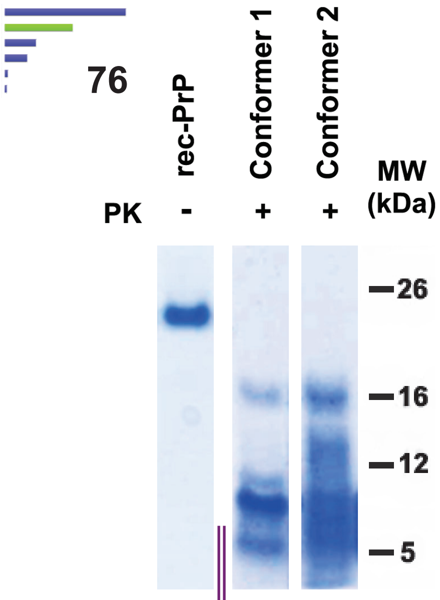

211 AA

2
Conformers

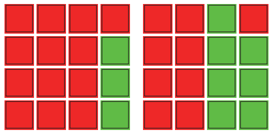

Tm[Exp]: ND
ΔΔG: -11.6

In vitro studies

TgVole (1x) Propagate
Human Ongoing

In vivo studies

TgVole (1x) Infectious
TgHuman Ongoing

pLDDT
[86.1]

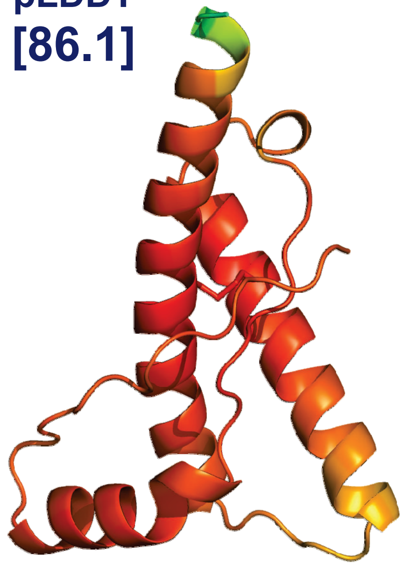

Protein code: 0178

Eastern hoolock gibbon

GenBank: BK064201

Hoolock leuconedys

Primates 35/45
Hylobatidae 1/1

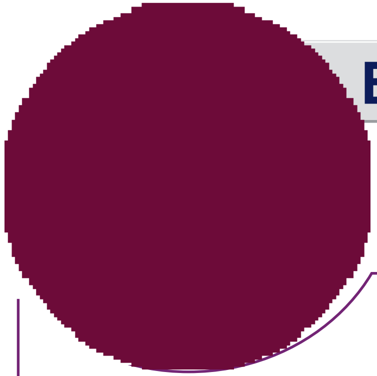

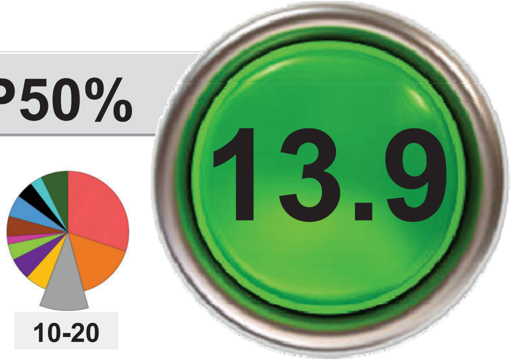

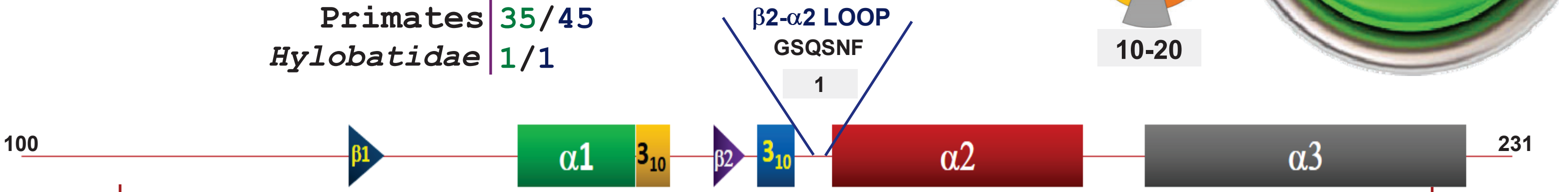

WNKPSKPKTNMKHMAGAAAAGAVVGGLGGYMLGSAMSRPIIHFGSDYEDRYYRENMHRYPNQVYYRPVDQYGSQSNFVHDCVNITIKQHTVTTTTKGENFTETDVKMMERVVEQMCITQYERESQAYYQRGSS-

I V M N W N NN N V QK EGR

PrP sequence differs by 12 amino acids from the bank vole PrP (see in red)

No deletions
No insertions
No polymorphic variants found

Distinct primary sequence across species

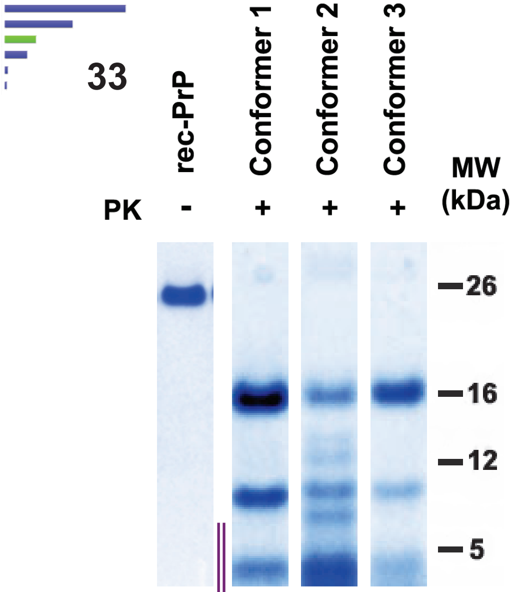

211 AA

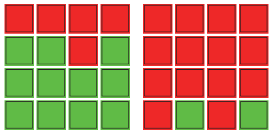

Tm[Exp]: ND

ΔΔG: -11.24

In vitro studies

Not tested
TgVole (1x)

Not tested
Eastern hoolock gibbon

In vivo studies

Not tested
TgVole (1x)

Not tested
Eastern hoolock gibbon

pLDDT
[85.7]

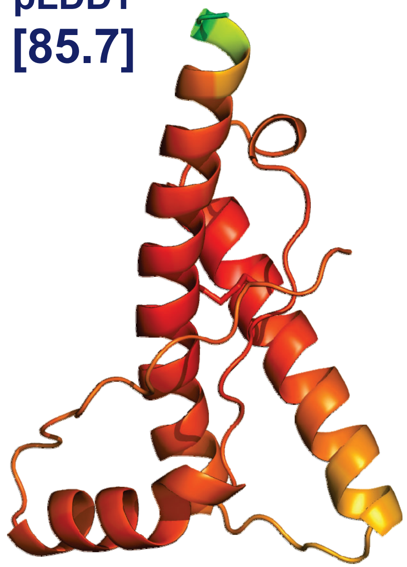

Protein code: 4530

Indri

GenBank: BK064058

P70% **47.9**

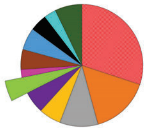
40-50

Indri indri

Primates	35/45
Indridae	2/2

β2-α2 LOOP
SNQNSF
36

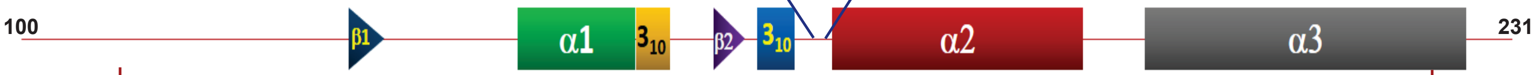

WNKPSKPKTSMKHVAGAAAAGAVVGGLGGYMLGSAMSRPLIHFGNDYEDRYYRENMYRYPNQVYYKPVDQYSNQNSFVHDCVNITIKQHTVTTTTKGENFTETDVKMMERVVEQMCVTQYQREYEAAYQRGSS-

NI M W N R N N K SQ Y EGR

PrP sequence differs by 11 amino acids from the bank vole PrP (see in red)

No deletions
No insertions
No polymorphic variants found

Distinct primary sequence across species

25

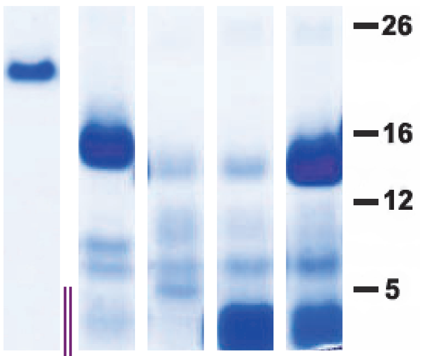

212 AA

4 Conformers

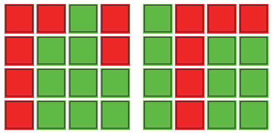

Tm[Exp]: ND

ΔΔG: -10.04

In vitro studies

TgVole (1x)	Not tested
Indri	Not tested

In vivo studies

TgVole (1x)	Not tested
Indri	Not tested

pLDDT [87.65]

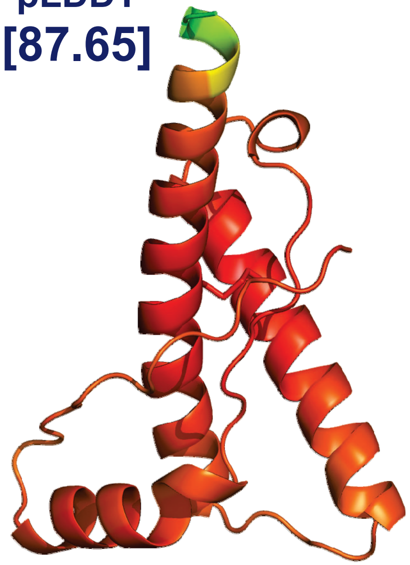

Brown woolly monkey

GenBank: AY765384

Lagothrix lagotricha

Primates 35/45
Atelidae 3/5

P61%

30

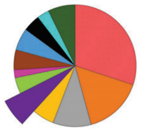

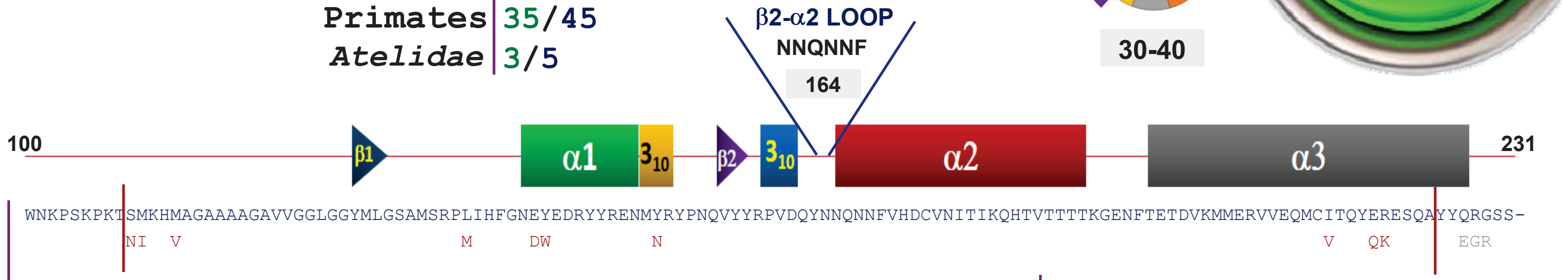

PrP sequence differs by 10 amino acids from the bank vole PrP (see in red)

No deletions
No insertions
No polymorphic variants found

Distinct primary sequence across species

25

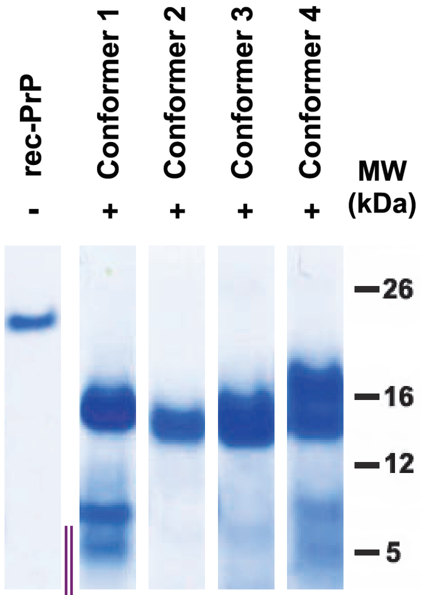

210 AA

4 Conformers

Tm^{Exp}: ND

ΔΔG: -7.89

pLDDT [86.89]

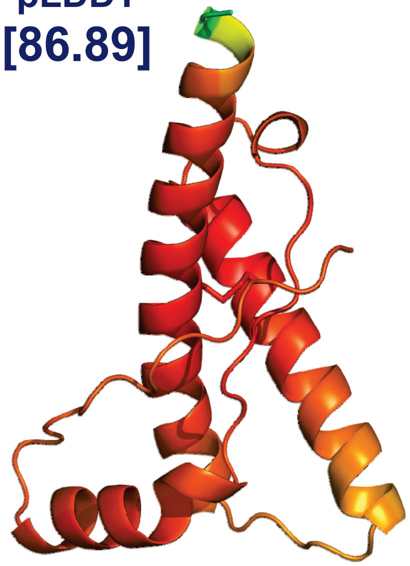

In vitro studies

Not tested
TgVole (1x)

Not tested
Brown woolly monkey

In vivo studies

Not tested
TgVole (1x)

Not tested
Brown woolly monkey

Protein code: 1389

Golden lion tamarin

GenBank: BK063917

Leontopithecus rosalia

Primates 35/45
Cebidae 7/8

P34%

0.9

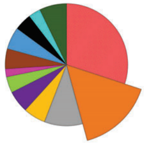

0-10

1 species sharing the same primary sequence

Golden-headed lion tamarin
Leontopithecus chrysomelas

β2-α2 LOOP
NNQNNF
164

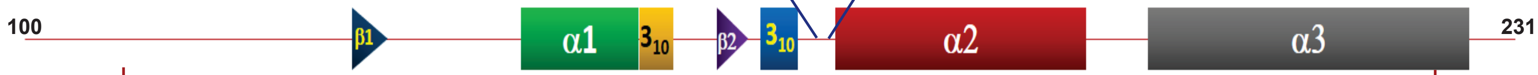

WNKPSKPKTNMKHVAGAAAAGAVVGGLGGYMLGSAMSRPLIHFGNDYEDRYYRENMYRYPNQVYYRPVDQYNNQNNFVHDCVNITIKQHTVTTTTKGENFTETDVKMMERVVEQMCITQYEKESQAYYQRGSS-
I M W N V Q EGR

PrP sequence differs by 6 amino acids from the bank vole PrP (see in red)

No deletions
No insertions
No polymorphic variants found

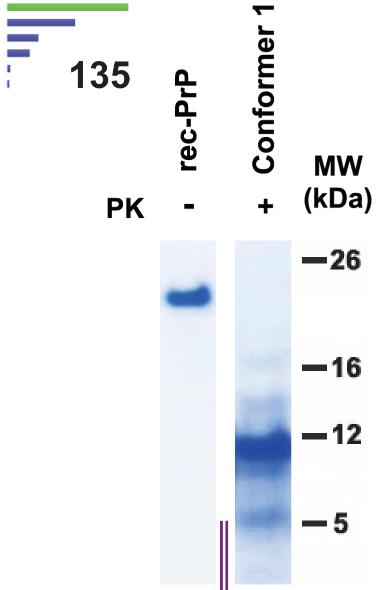

210 AA

1
Conformers

Tm^{Exp}: ND

ΔΔG: -4.23

In vitro studies

TgVole (1x) Not tested

Golden lion tamarin Not tested

In vivo studies

TgVole (1x) Not tested

Golden lion tamarin Not tested

pLDDT
[87.28]

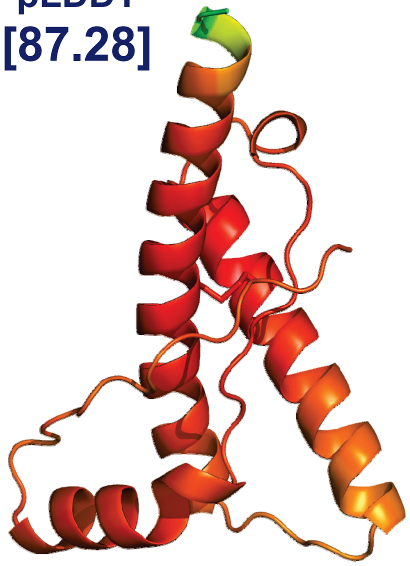

Protein code: 1437

Red slender loris

GenBank: BK064198

Loris tardigradus

Primates	35/45
Lorisidae	2/3

P39%

4.8

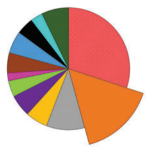

0-10

β2-α2 LOOP
SNQNNF
273

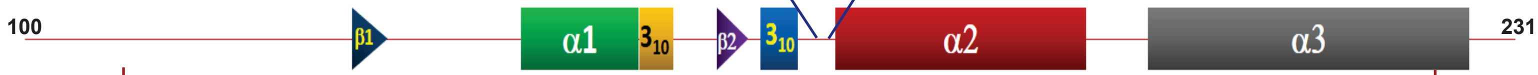

WNKPSKPKTSMKHVAGAAAAGAVVGGLGGYMLGSAMSRPLIHFGNDYEDRYYRENMNRYPGQVYYRPVDQYSNQNNFVHDCVNITIKQHTVTTTTKGENFTETDVKIMERMVEQMCVTQYQREYQAAYQRGSS-

NI M W N N M V K S Y EGR

PrP sequence differs by 10 amino acids from the bank vole PrP (see in red)

No deletions
No insertions
No polymorphic variants found

135

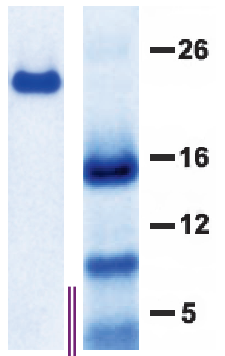

211 AA

1 Conformers

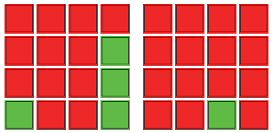

Tm^{Exp}: ND

ΔΔG: 10.2

In vitro studies

TgVole (1x) Not tested

Red slender loris Not tested

In vivo studies

TgVole (1x) Not tested

Red slender loris Not tested

pLDDT [88.22]

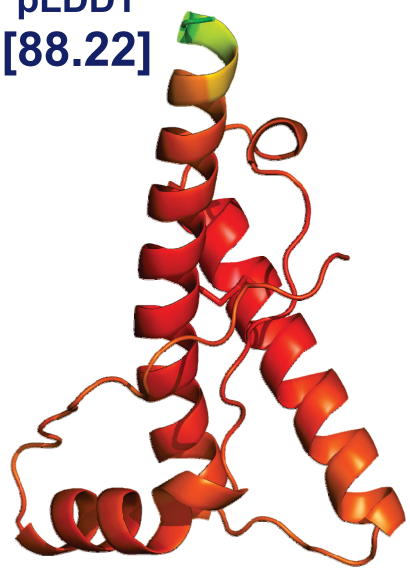

Distinct primary sequence across species

Protein code: 4527

Pigtail macaque

GenBank: XM_011741222

Macaca nemestrina

Primates 35/45
Cercopithecidae 7/7

P59%

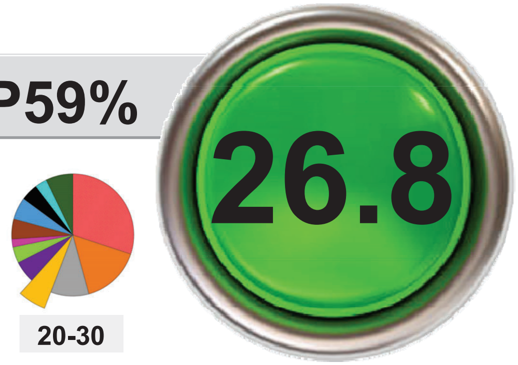

β2-α2 LOOP
SNQNNF
273

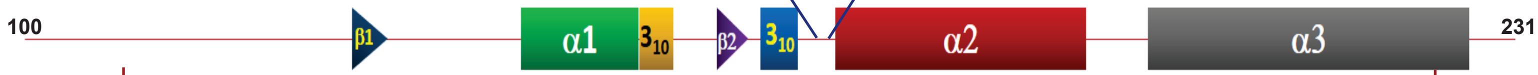

WHKPSKPKTSMKHMAGAAAAGAVVGGLGGYMLGSAMSRPLIHFGNDYEDRYYRENMYRYPNQVYYRPVDQYSNQNNFVHDCVNITIKQHTVTTTTKGENFTETDVKMMERVVEQMCITQYEKESQAYYQRGSS-
N NI V M W N N V Q EGR

PrP sequence differs by 9 amino acids from the bank vole PrP (see in red)

No deletions
No insertions
No polymorphic variants found

20 species sharing the same primary sequence

- **Hamadryas baboon** *Papio hamadryas*
- **Olive baboon** *Papio anubis*
- **Assam macaque** *Macaca assamensis*
- **Barbary macaque** *Macaca sylvanus*
- **Stump-tailed macaque** *Macaca arctoides*
- **Rhesus macaque** *Macaca mulatta*
- **Japanese macaque** *Macaca fuscata*
- **Grivet** *Chlorocebus aethiops*
- **Black crested mangabey** *Lophocebus aterrimus*
- **DeBrazza's monkey** *Cercopithecus neglectus*
- **Guenons** *Cercopithecus diana*
- **Mona monkey** *Cercopithecus mona*
- **Green monkey** *Chlorocebus sabaeus*
- **Pigtail macaque** *Macaca nemestrina*
- **Allen's swamp monkey** *Allenopithecus nigroviridis*
- **Patas monkey** *Erythrocebus patas*
- **Guinea baboon** *Papio papio*
- **Sykes' monkey** *Cercopithecus albogularis*
- **Lion-tailed macaque** *Macaca silenus*
- **Formosan rock macaque** *Macaca cyclopis*

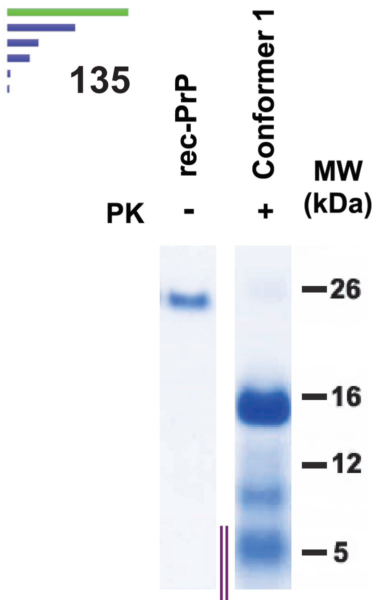

219 AA

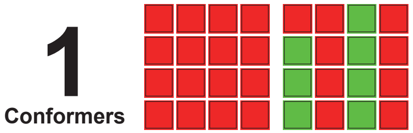

Tm^{Exp}: ND

ΔΔG: -6.33

In vitro studies

TgVole (1x) — No propagation

Pigtail macaque — Not tested

In vivo studies

TgVole (1x) — Ongoing

Pigtail macaque — Not tested

pLDDT [85.9]

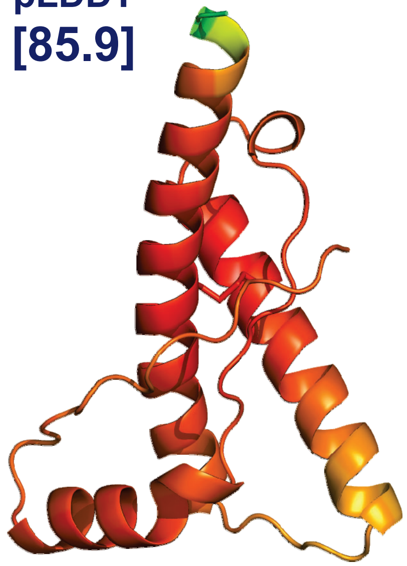

Protein code: 1970

Drill

GenBank: XM_011975232

Mandrillus leucophaeus

Primates 35/45
Cercopithecidae 7/7

P64% **35.7**

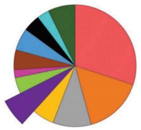

β2-α2 LOOP
SNQNNF
273

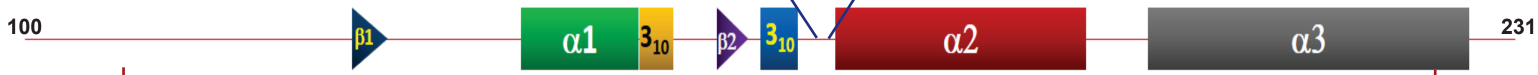

WHKPNKPKTSMKHMAGAAAAGAVVGGLGGYMLGSAMSRPLIHFGNEYEDRYYRENMYRYPNQVYYRPVDQYSNQNNFVHDCVNITIKQHTVTTTTKGENFTETDVKMMERVVEQMCITQYEKESQAYYQRGSS-
N S NI V M DW N N V Q EGR

PrP sequence differs by 10 amino acids from the bank vole PrP (see in red)

No deletions
No insertions
No polymorphic variants found

Distinct primary sequence across species

33

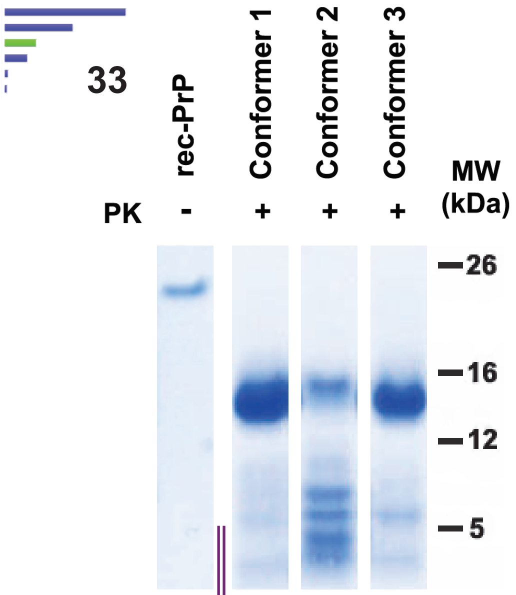

219 AA

3 Conformers

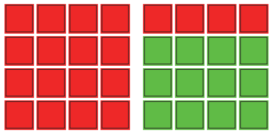

Tm^{Exp}: ND
ΔΔG: -10.7

In vitro studies

TgVole (1x) Not tested
Drill Not tested

In vivo studies

TgVole (1x) Not tested
Drill Not tested

pLDDT
[86.25]

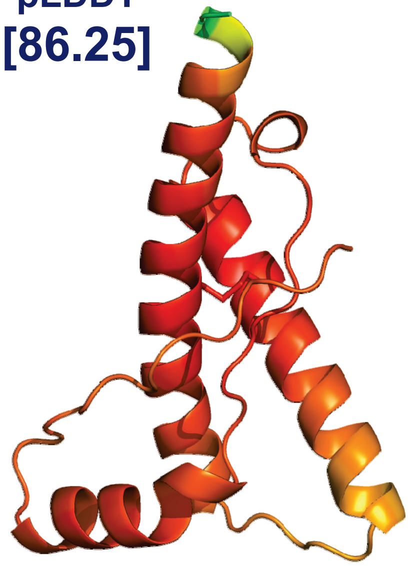

Protein code: 2078

Mandrill

GenBank: U08303

Mandrillus sphinx

Primates	35/45
Cercopithecidae	7/7

P34%

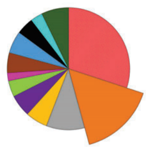

0.9

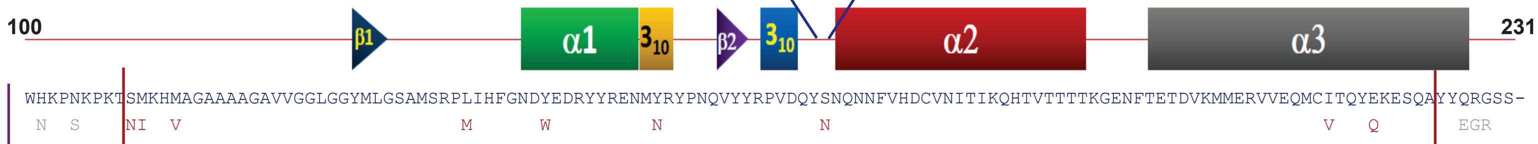

PrP sequence differs by 9 amino acids from the bank vole PrP (see in red)

No deletions

No insertions

No polymorphic variants found

Distinct primary sequence across species

135

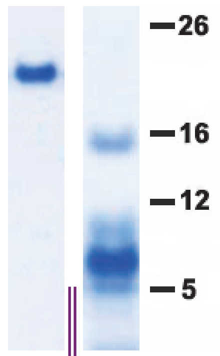

211 AA

1

Conformers

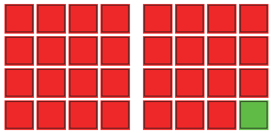

Tm^{Exp}: ND

ΔΔG: -6.05

In vitro studies

TgVole (1x)	Not tested
Mandrill	Not tested

In vivo studies

TgVole (1x)	Not tested
Mandrill	Not tested

pLDDT [85.56]

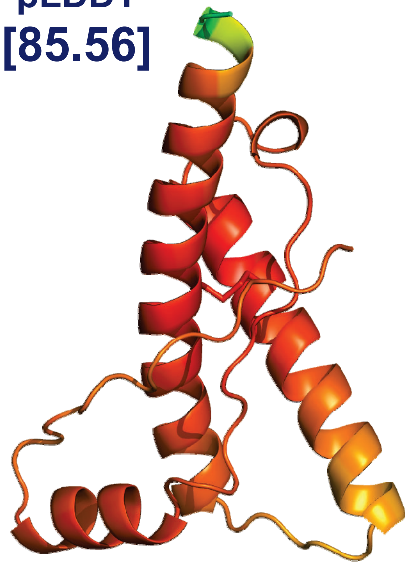

Protein code: 1208

Gray mouse lemur

GenBank: NM_001309920

P29%

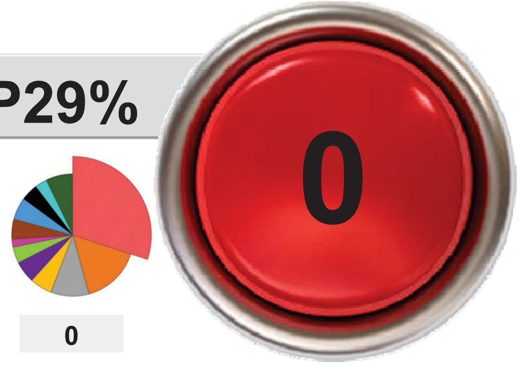

Microcebus murinus

Primates	35/45
Cheirogaleidae	1/4

3 species sharing the same primary sequence

Reddish-gray mouse lemur
Microcebus griseorufus

Mittermeier's mouse lemur
Microcebus mittermeieri

Northern rufous mouse lemur
Microcebus tavaratra

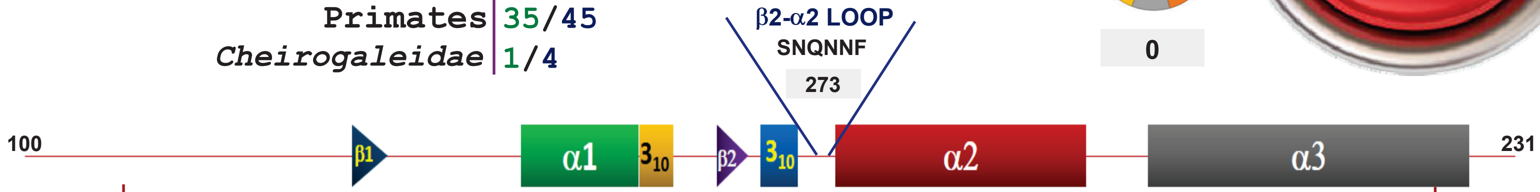

WNKPSKPKTNMKHMAGAAAAGAVVGGLGGYMLGSAMSRPLMHFGNDYEDRYYRENMNRYPNQVYYKPVDQYSNQNNFVHDCVNITIKQHTVTTTTKGENFTETDVKMMERVVEQMCVTQYQKEYQAAYQRGSS-

I V MI W R N S Y EGR

PrP sequence differs by 8 amino acids from the bank vole PrP (see in red)

No deletions

No insertions

No polymorphic variants found

Tm^{Exp}: ND

ΔΔG: 1.62

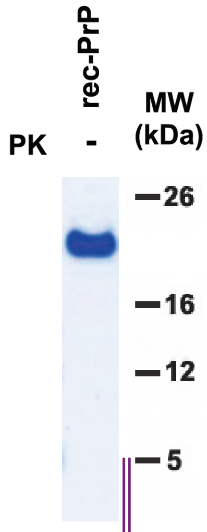

212 AA

NO MISFOLDING

0 Conformers

In vitro studies

NOT APPLICABLE

In vivo studies

NOT APPLICABLE

pLDDT [85.44]

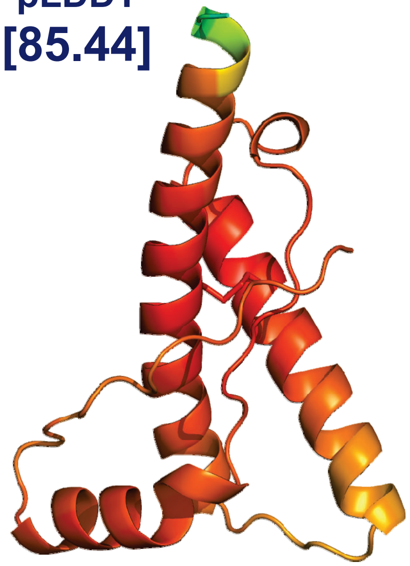

Protein code: 0933

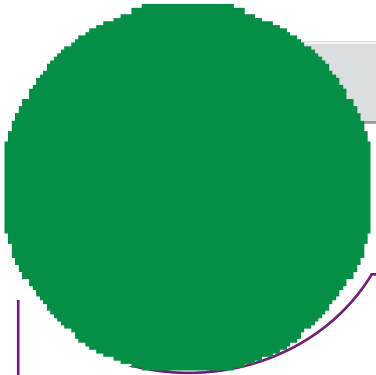

Golden-brown mouse lemur

GenBank: BK064086

Microcebus ravelobensis

Primates	35/45
Cheirogaleidae	1/4

P29%

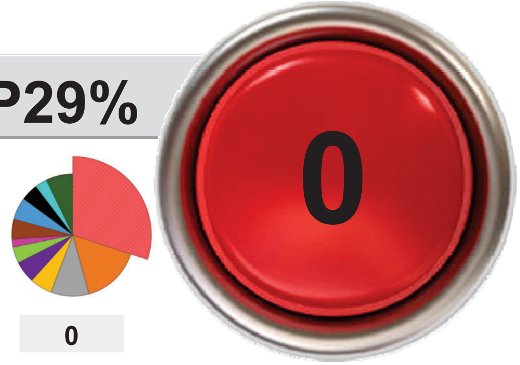

β2-α2 LOOP
SNQNNF
273

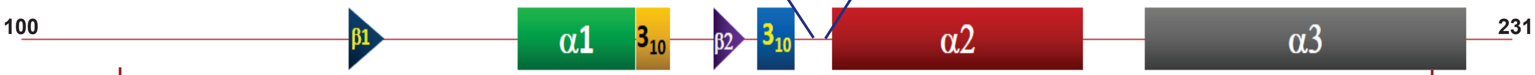

WNKPSKPKTNMKHMAGAAAAGAVVGGLGGYMLGSAMSRPLMHFGNDYEDRYYRENMYRYPNQVYYKPVDQYSNQNNFVHDCVNITIKQHTVTTTTKGENFTETDVKMMERVVEQMCVTQYQKEYQAAYQRGSS-
I V MI W N R N S Y EGR

PrP sequence differs by 9 amino acids from the bank vole PrP (see in red)

No deletions
No insertions
No polymorphic variants found

Tm^{Exp}: ND

ΔΔG: -0.56

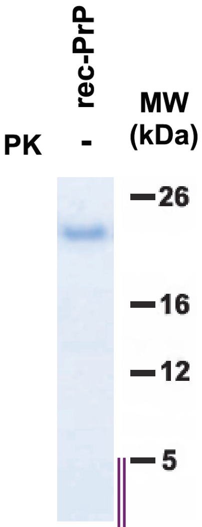

212 AA

NO MISFOLDING

0
Conformers

In vitro studies

NOT APPLICABLE

In vivo studies

NOT APPLICABLE

pLDDT
[85.56]

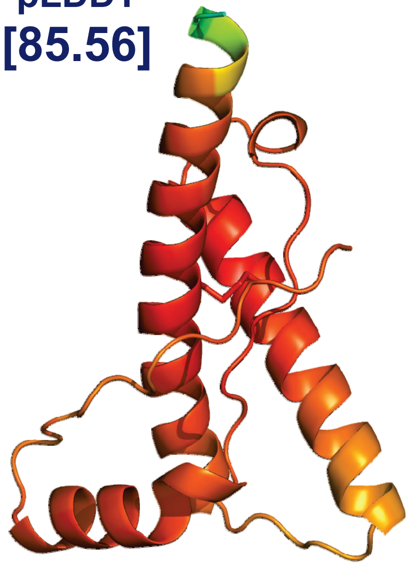

Distinct primary sequence across species

Angolan talapoin

GenBank: BK064818

Miopithecus talapoin

Primates	35/45
Cercopithecidae	7/7

P41%

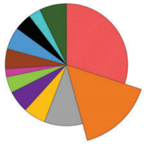

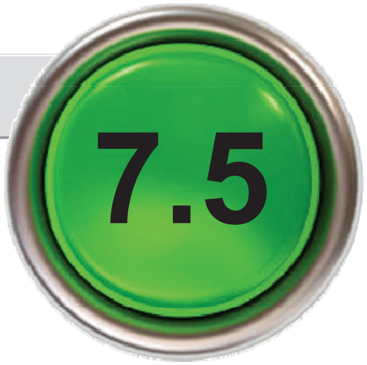

0-10

β2-α2 LOOP
SSQNNF
42

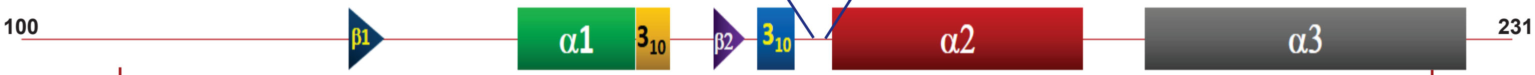

WHKPSKPKTSMKHMAGAAAAGAVVGGLGGYMLGSAMSRPLIHFGNDYEDRYYRENMYRYPNQVYYRPVDQYSSQNNFVHDCVNITIKQHTVTTTTKGENFTETDVKMMERVVEQMCITQYEKESQAYYQRGSS-

N NI V M W N NN V Q EGR

PrP sequence differs by 10 amino acids from the bank vole PrP (see in red)

No deletions
No insertions
No polymorphic variants found

Distinct primary sequence across species

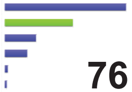

76

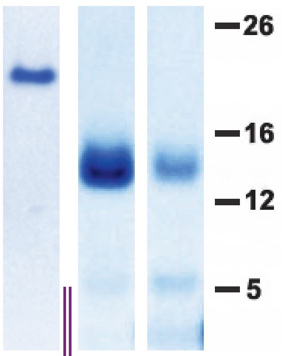

211 AA

2
Conformers

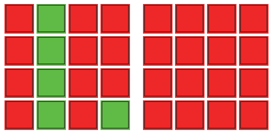

Tm[Exp]: ND

ΔΔG: -10.99

In vitro studies

TgVole (1x) Not tested

Angolan talapoin Not tested

In vivo studies

TgVole (1x) Not tested

Angolan talapoin Not tested

pLDDT
[85.96]

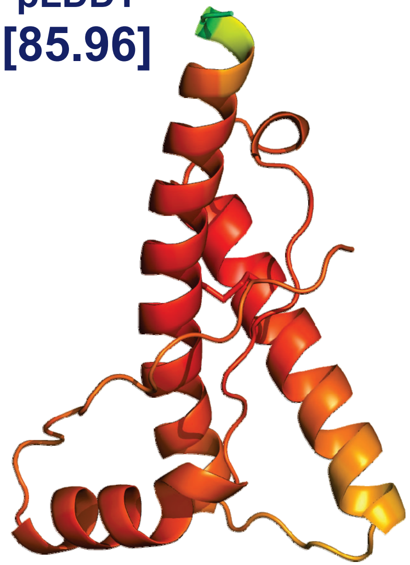

Protein code: 4508

Coquerel's giant mouse lemur

GenBank: BK064094

P60% **27.9**

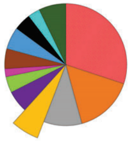

20-30

Mirza coquereli

Primates 35/45
Cheirogaleidae 1/4

β2-α2 LOOP
SNQNNF
273

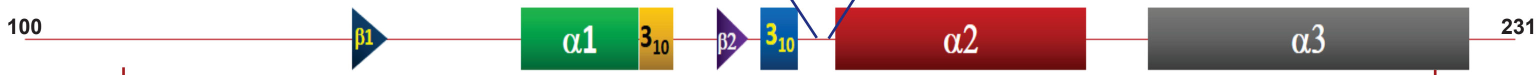

WNKPNKPKTNMKHVAGAAAAGAVVGGLGGYMLGSAMSRPLIHFGNDYEDRYYRENMYRYPNQVYYRPVDQYSNQNNFVHDCVNITIKQHTVTTTTKGENFTETDVKMMERVVEQMCVTQYQKEYQAAYQRGSS-
S I M W N N S Y EGR

PrP sequence differs by 6 amino acids from the bank vole PrP (see in red)

No deletions
No insertions
No polymorphic variants found

1 species sharing the same primary sequence

Northern giant mouse lemur
Mirza zaza

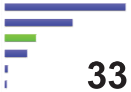

33

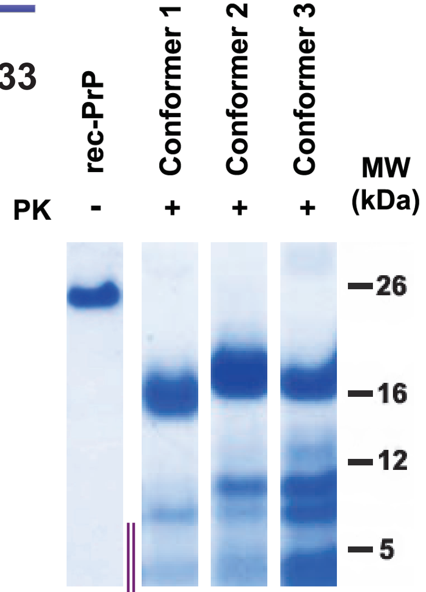

212 AA

3 Conformers

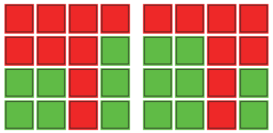

Tm[Exp]: ND

ΔΔG: 4.23

In vitro studies

TgVole (1x) Not tested

Coquerel's giant mouse lemur Not tested

In vivo studies

TgVole (1x) Not tested

Coquerel's giant mouse lemur Not tested

pLDDT
[85.02]

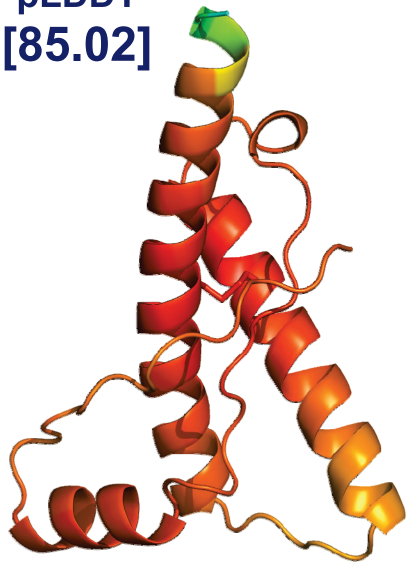

Protein code: 3273

Sunda slow loris

GenBank: BK064114

Nycticebus coucang

Primates 35/45
Lorisidae 2/3

P46%

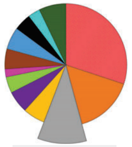

10-20

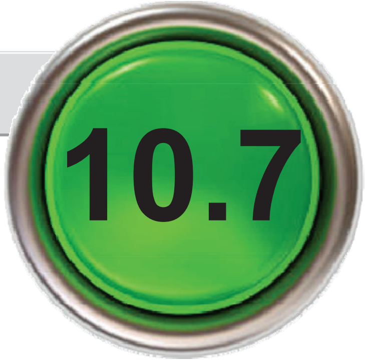

Bengal slow loris
Nycticebus bengalensis

1 species sharing the same primary sequence

β2-α2 LOOP
SNQNNF
273

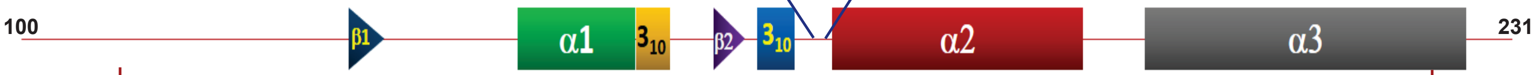

WNKPSKPKTSMKHMAGAAAAGAVVGGLGGYMLGSAMSRPLIHFGNDYEDRYYRENMNRYPSQVYYRPVDQYSNQNNFVHDCVNITIKQHTVTTTTKGENFTETDVKIMERMVEQMCVTQYQREYQAAYQRGSS-
NI V M W N N M V K S Y EGR

PrP sequence differs by 11 amino acids from the bank vole PrP (see in red)

No deletions
No insertions
No polymorphic variants found

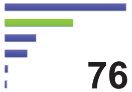

76

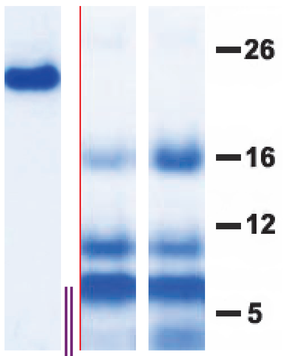

211 AA

2
Conformers

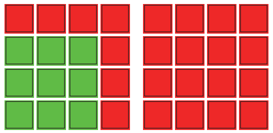

Tm^{Exp}: ND

ΔΔG: -1.87

In vitro studies

TgVole (1x) Not tested

Sunda slow loris Not tested

In vivo studies

TgVole (1x) Not tested

Sunda slow loris Not tested

pLDDT
[87.46]

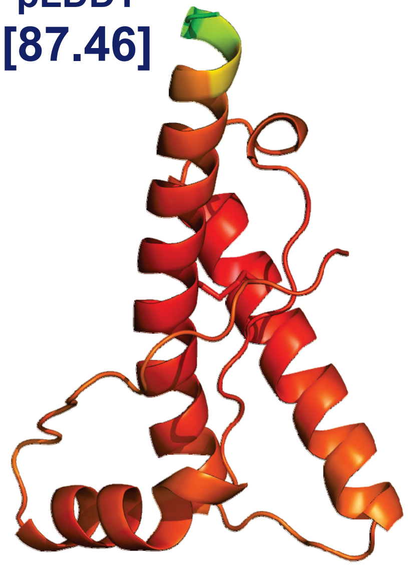

Protein code: 3294

Pygmy slow loris

GenBank: OR47281

Nycticebus pygmaeus

Primates	35/45
Lorisidae	2/3

P29%

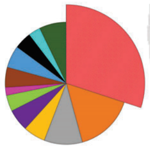

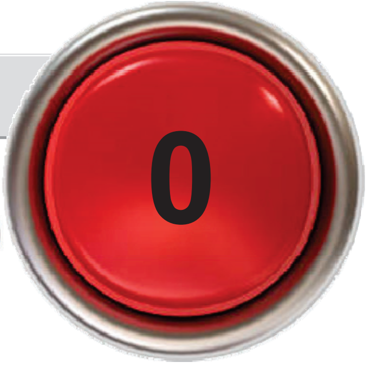

0

β2-α2 LOOP
SNQNNF
273

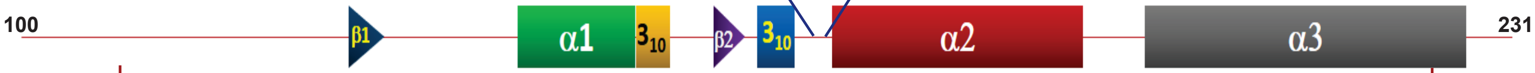

WNKPSKPKTSMKHMAGAAAAGAVVGGLGGYMLGSAMSRPLIHFGNDYEDRYYRENMNRYPSQVYYRPVDQYSNQNNFVHECVNITIKQHTVTTTTKGDNFTEMDVKIMERMVEQMCVTQYQREYQAAYQRGSS-
NI V M W N N D E T M V K S Y EGR

PrP sequence differs by 14 amino acids from the bank vole PrP (see in red)

No deletions

No insertions

No polymorphic variants found

Distinct primary sequence across species

PK | rec-PrP -

MW (kDa)

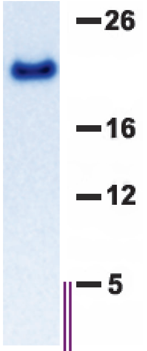

211 AA

NO MISFOLDING

0 Conformers

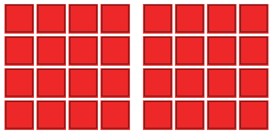

In vitro studies

NOT APPLICABLE

In vivo studies

NOT APPLICABLE

Tm^{Exp}: ND

ΔΔG: -6.58

pLDDT [90.2]

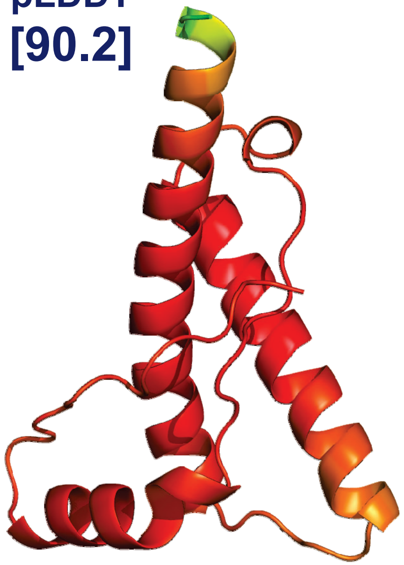

Protein code: 4835

Northern greater galago

GenBank: XM_003788036

Otolemur garnettii

P35%

1.8

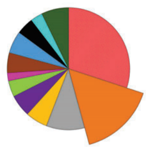

Primates 35/45
Galagidae 1/2

β2-α2 LOOP
SNQNNF
273

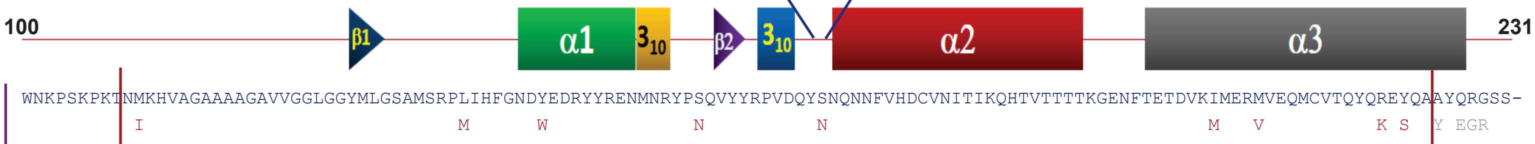

PrP sequence differs by 9 amino acids from the bank vole PrP (see in red)

No deletions
No insertions
No polymorphic variants found

Tm[Exp]: ND

ΔΔG: -2.15

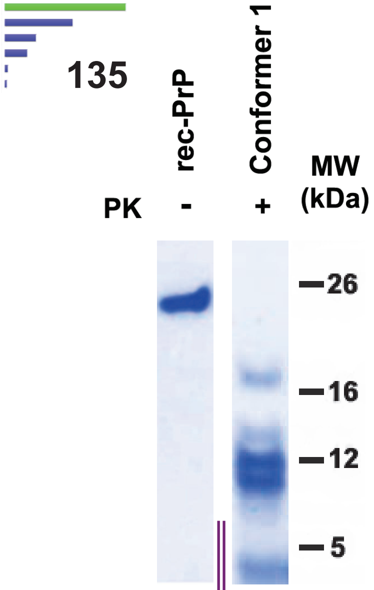

217 AA

1 Conformers

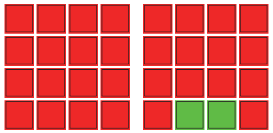

In vitro studies

Not tested
TgVole (1x)

Not tested
Northern greater galago

In vivo studies

Not tested
TgVole (1x)

Not tested
Northern greater galago

pLDDT [84.32]

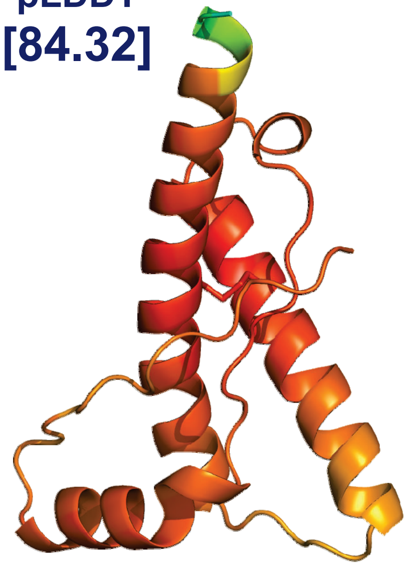

Distinct primary sequence across species

Protein code: 1105

Chimpanzee

GenBank: U08296

Pan troglodytes

Primates 35/45
Hominidae 4/4

P55% **20.7**

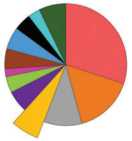

20-30

β2-α2 LOOP
SSQNNF
42

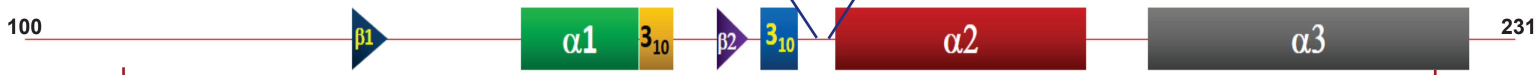

WNKPSKPKTNMKHMAGAAAAGAVVGGLGGYMLGSAMSRPIIHFGSDYEDRYYRENMHRYPNQVYYRPMDQYSSQNNFVHDCVNITIKQHTVTTTTKGENFTETDVKMMERVVEQMCITQYERESQAYYQRGSS-

I V M N W N V NN V QK EGR

PrP sequence differs by 12 amino acids from the bank vole PrP (see in red)

No deletions
No insertions
No polymorphic variants found

9 species sharing the same primary sequence

Agile gibbon *Hylobates agilis*
Lar gibbon *Hylobates lar*
Silvery gibbon *Hylobates moloch*
Müller's gibbon *Hylobates muelleri*
Pileated gibbon *Hylobates pileatus*
N white-cheeked gibbon *Nomascus leucogenys*
S white-cheeked gibbon *Nomascus siki*
Bonobo *Pan paniscus*
Siamang *Symphalangus syndactylus*

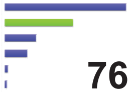

76

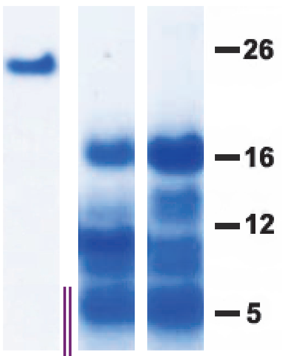

211 AA

2 Conformers

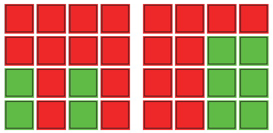

Tm^{Exp}: ND

ΔΔG: -9.88

In vitro studies

TgVole (1x)	Not tested
Chimpanzee	Not tested

In vivo studies

TgVole (1x)	Not tested
Chimpanzee	Not tested

pLDDT [85.22]

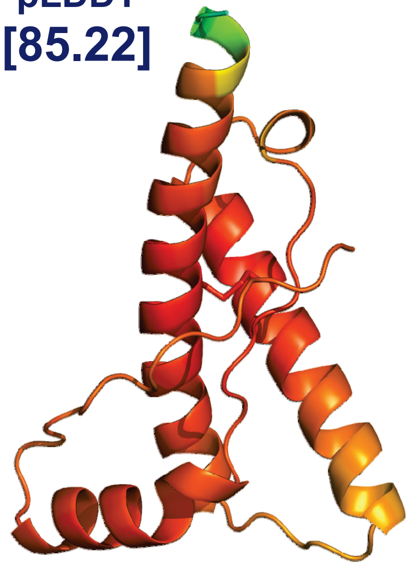

Rio Tapajós saki

GenBank: AY765379

Pithecia irrorata

Primates 35/45
Pitheciidae 3/4

White-faced saki
Pithecia pithecia

1 species sharing the same primary sequence

P29%

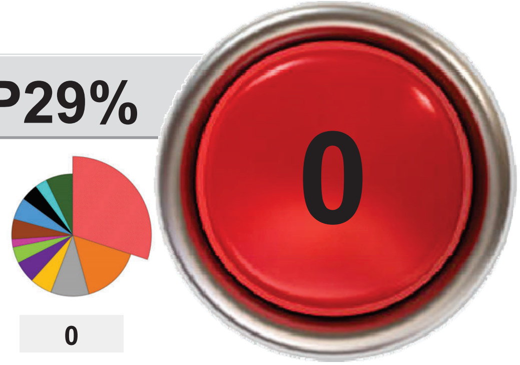

β2-α2 LOOP
SNQNNF
273

100 β1 α1 3 10 β2 3 10 α2 α3 231

WNKPSKPKTNMKHMAGAAAAGAVVGGLGGYMLGSAMSRPLIHFGNDYEDRYYRENMYRYPNQVYYRPVDQYSNQNNFVHDCVNITIKQHTVTTTTKGENFTETDVKMMERVVEQMCITQYERESQAYYQRGSS-
I V M W N N V QK EGR

PrP sequence differs by 9 amino acids from the bank vole PrP (see in red)

No deletions
No insertions
No polymorphic variants found

Tm^{Exp}: ND

ΔΔG: -6.48

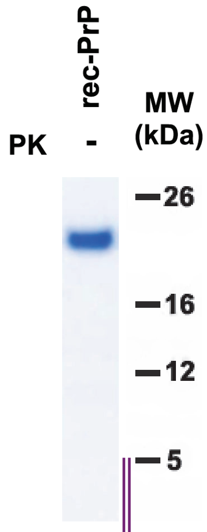

211 AA

NO MISFOLDING

0
Conformers

In vitro studies

NOT APPLICABLE

In vivo studies

NOT APPLICABLE

pLDDT
[85.46]

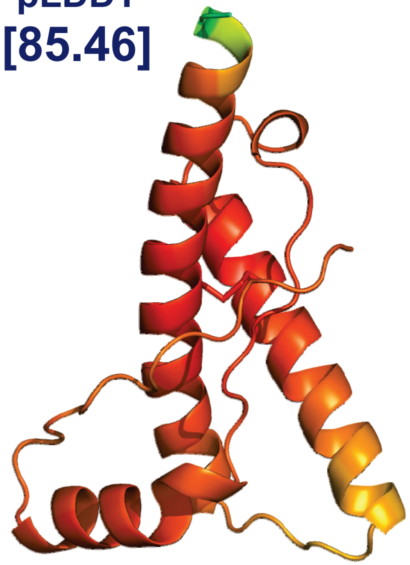

Protein code: 1411

Bornean orangutan

GenBank: U08305

Pongo pygmaeus

Primates	35/45
Hominidae	4/4

P47%

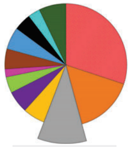

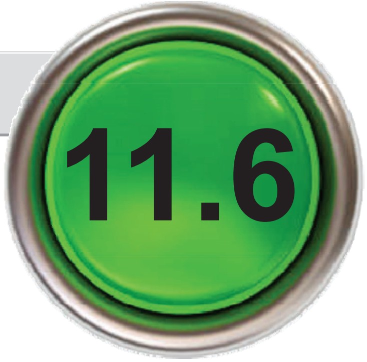

1 species sharing the same primary sequence

Sumatran orangutan
Pongo abelii

β2-α2 LOOP
SNQNNF
273

100

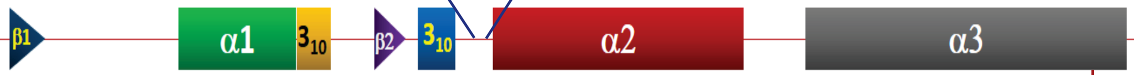

231

WNKPSKPKTNMKHMAGAAAAGAVVGGLGGYMLGSAMSRPIIHFGNDYEDRYYRENMYRYPNQVYYRPVDQYSNQNNFVHDCVNITIKQHTVTTTTKGENFTETDVKMMERVVEQMCITQYERESQAYYQRGSS-

I V M W N N V QK EGR

PrP sequence differs by 9 amino acids from the bank vole PrP (see in red)

No deletions
No insertions
No polymorphic variants found

135

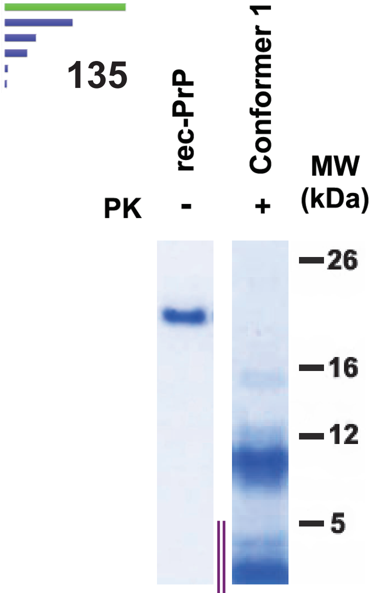

211 AA

1
Conformers

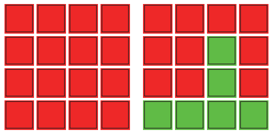

Tm^{Exp}: ND

ΔΔG: -10.55

In vitro studies

TgVole (1x) Not tested

Bornean orangutan Not tested

In vivo studies

TgVole (1x) Not tested

Bornean orangutan Not tested

pLDDT
[86.48]

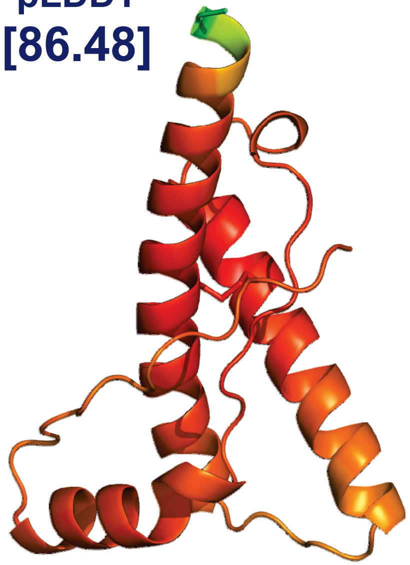

Protein code: 0926

Greater bamboo lemur

GenBank: BK063921

P34%

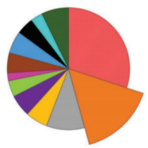

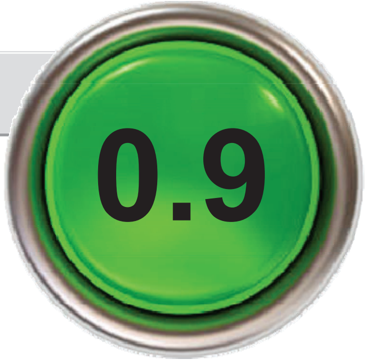

Prolemur simus

Primates 35/45
Lemuridae 1/1

β2-α2 LOOP
SSQNNF
42

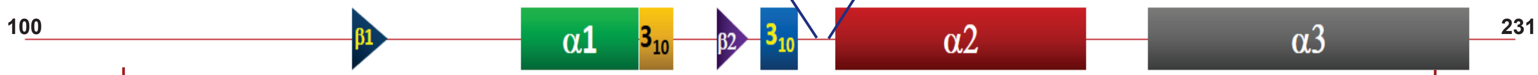

WNKPSKPKTNMKHVAGAAAAGAVVGGLGGYMLGSAMSRPLIHFGNDYEDRYYRENMYRYPNQVYYKPVDQYSSQNNFVHDCVNITIKQHTVTTTTKGENFTETDVKMMERVVEQMCVTQYQREYEAAAYQRGSS-

I M W N R NN K SQ Y EGR

PrP sequence differs by 10 amino acids from the bank vole PrP (see in red)

No deletions
No insertions
No polymorphic variants found

5 species sharing the same primary sequence

White-headed lemur *Eulemur albifrons*
Common brown lemur *Eulemur fulvus*
Black lemur *Eulemur macaco*
Mongoose lemur *Eulemur mongoz*
Ring-tailed lemur *Lemur catta*

Tm^{Exp}: ND
ΔΔG: -6.96

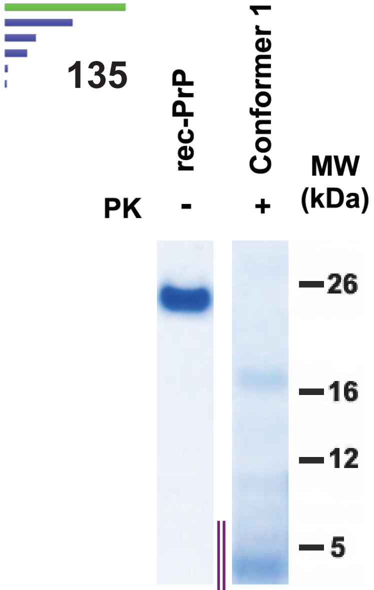

211 AA

1
Conformers

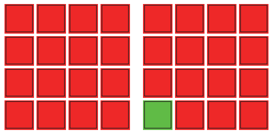

In vitro studies

TgVole (1x) Not tested
Greater bamboo lemur Not tested

In vivo studies

TgVole (1x) Not tested
Greater bamboo lemur Not tested

pLDDT
[85.48]

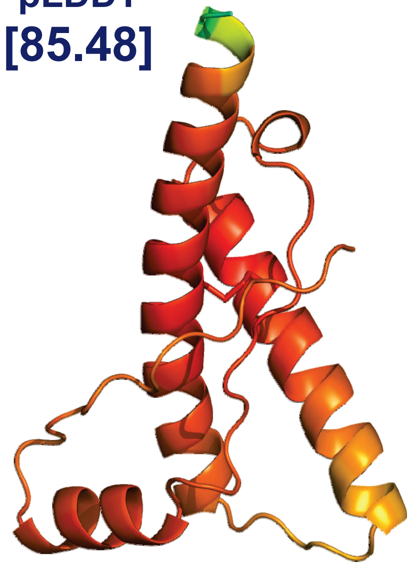

Protein code: 2028

Coquerel's sifaka

GenBank: XM_012639270

Propithecus coquereli

Primates 35/45
Indridae 2/2

P64%

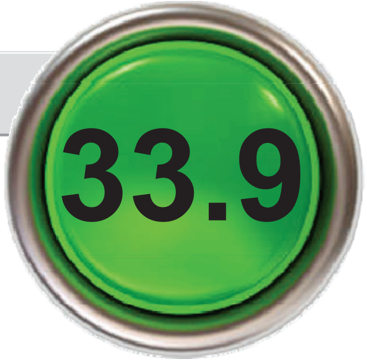

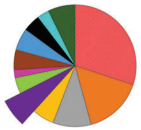

30-40

β2-α2 LOOP
SSQNSF
6

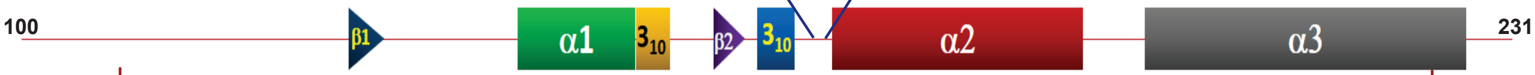


```
WNKPSKPKTNMKHVAGAAAAGAVVGGLGGYMLGSAMSRPLIHFGNDYEDRYYRENMYRYPNQVYYKPVDQYSSQNSFVHDCVNITIKQHTVTTTTKGENFTETDVKMMERVVEQMCVTQYQREYEAAYQRGSS-
          I                       M     W        N       R     NN  N                                                      K SQ Y EGR
```

PrP sequence differs by 11 amino acids from the bank vole PrP (see in red)

No deletions
No insertions
No polymorphic variants found

Distinct primary sequence across species

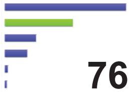

76

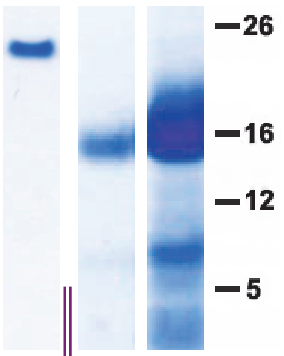

212 AA

2
Conformers

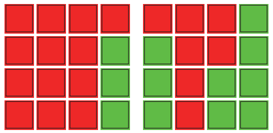

Tm^{Exp}: ND

ΔΔG: -3.69

In vitro studies

TgVole (1x) — Not tested

Coquerel's sifaka — Not tested

In vivo studies

TgVole (1x) — Not tested

Coquerel's sifaka — Not tested

pLDDT
[84.91]

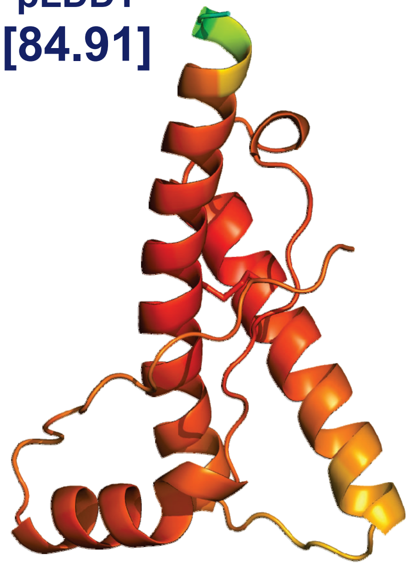

Protein code: 1127

Pied tamarin

GenBank: AY765388

Saguinus bicolor

Primates 35/45
Cebidae 7/8

P54%

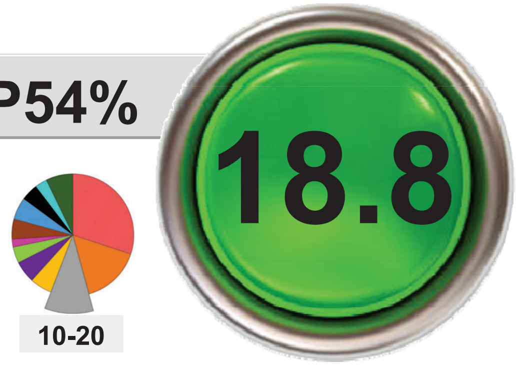

β2-α2 LOOP
NNQNNF
164

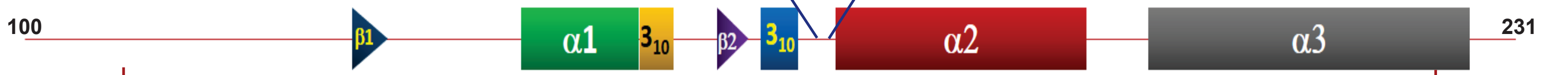

WNKPSKPKTNMKHVAGAAAAGAVVGGLGGYMLGSAMSRPLIHFGNDYEGRYYRENMYRYPNQVYYRPVDQYNNQNNFVHDCVNITIKQHTVTTTTKGENFTETDVKMMERVVEQMCITQYEKESQAYYQRGSS-
I M WD N V Q EGR

PrP sequence differs by 7 amino acids from the bank vole PrP (see in red)

No deletions
No insertions
No polymorphic variants found

Distinct primary sequence across species

76

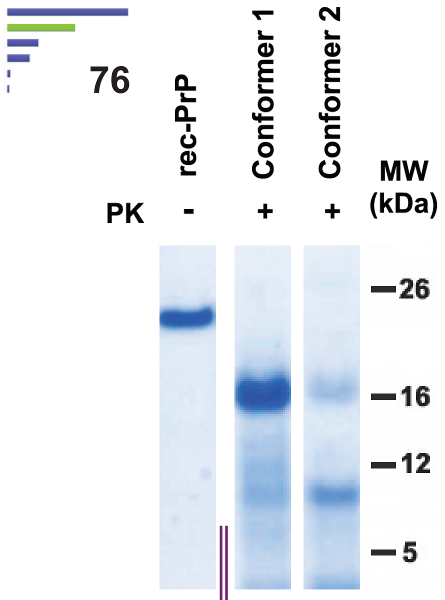

210 AA

2
Conformers

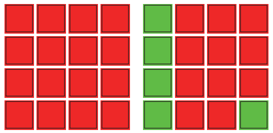

Tm^{Exp}: ND

ΔΔG: 6.16

In vitro studies

TgVole (1x) — Not tested

Pied tamarin — Not tested

In vivo studies

TgVole (1x) — Not tested

Pied tamarin — Not tested

pLDDT
[84.8]

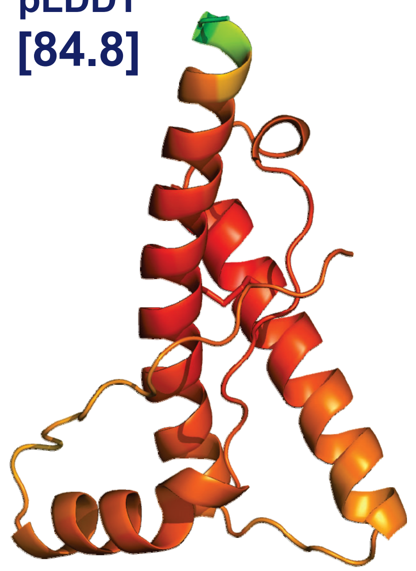

Emperor tamarin

GenBank: BK064034

Saguinus imperator

Primates 35/45
Cebidae 7/8

P73%

53.6

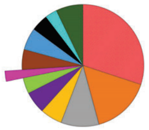

50-60

β2-α2 LOOP
NNQNNF
164

100

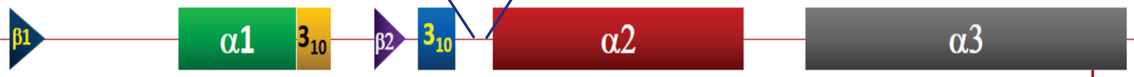

231

WNKPSKPKTNMKHVAGAAAAGAVVGGLGGYMLGSAMSRPLIHFGNDYEDRYYRENMYRYPNQVYYRPVDQYNNQNNFVHDCVNITIKQHTVTTTTKGENFTETDVKMMERVVEQMCITQYEKESQAYYQRGSS-

I M W N V Q EGR

PrP sequence differs by 6 amino acids from the bank vole PrP (see in red)

No deletions
No insertions
No polymorphic variants found

Distinct primary sequence across species

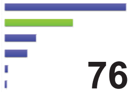

76

210 AA

PK	rec-PrP	Conformer 1	Conformer 2	MW (kDa)
	-	+	+	

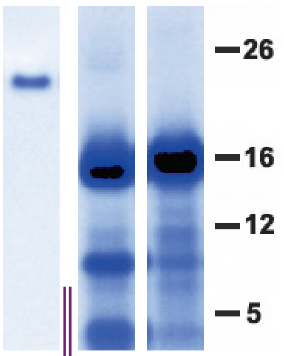

2
Conformers

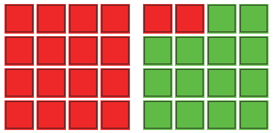

Tm^{Exp}: ND

ΔΔG: 4.37

In vitro studies

TgVole (1x) — Not tested

Emperor tamarin — Not tested

In vivo studies

TgVole (1x) — Not tested

Emperor tamarin — Not tested

pLDDT
[86.12]

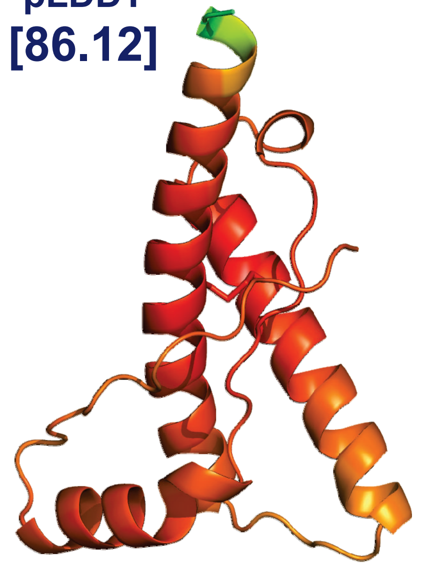

Golden-handed tamarin

GenBank: BK064187

P41%

7.1

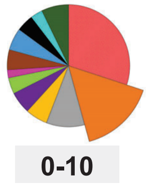

Saguinus midas

Primates	35/45
Cebidae	7/8

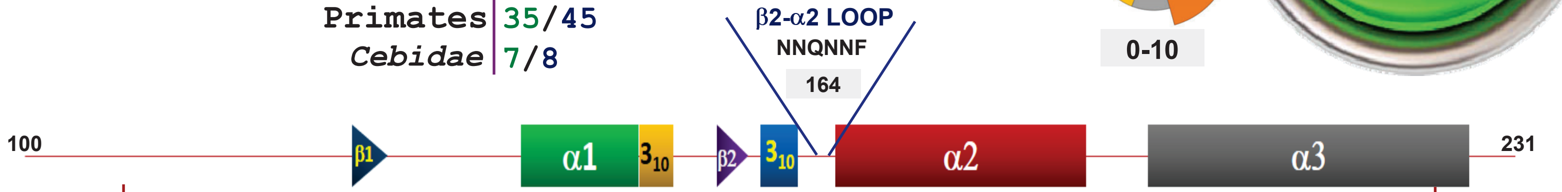

WNKPSKPKTNMKHMAGAAAAGAVVGGLGGYMLGSAMSRPLIHFGNDYEDRYYRENMYRYPNQVYYRPVDQYNNQNNFVHDCVNITIKQHTVTTTTKGENFTETDVKMMERVVEQMCITQYEKESQAYYQRGSS-

I V M W N V Q EGR

PrP sequence differs by 7 amino acids from the bank vole PrP (see in red)

No deletions
No insertions
No polymorphic variants found

Distinct primary sequence across species

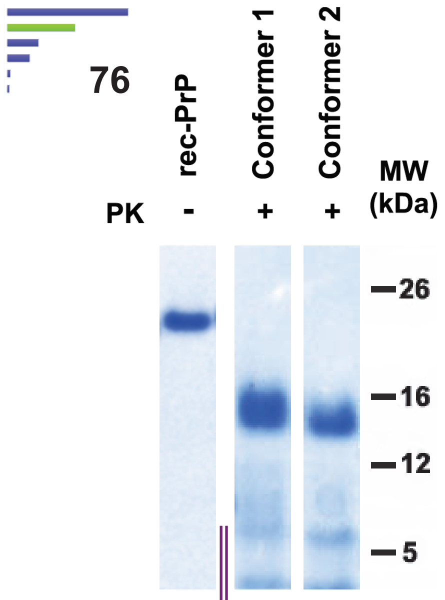

210 AA

2
Conformers

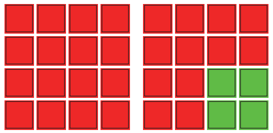

Tm^Exp: ND

ΔΔG: -4.49

In vitro studies

TgVole (1x) — Not tested

Golden-handed tamarin — Not tested

In vivo studies

TgVole (1x) — Not tested

Golden-handed tamarin — Not tested

pLDDT [86.75]

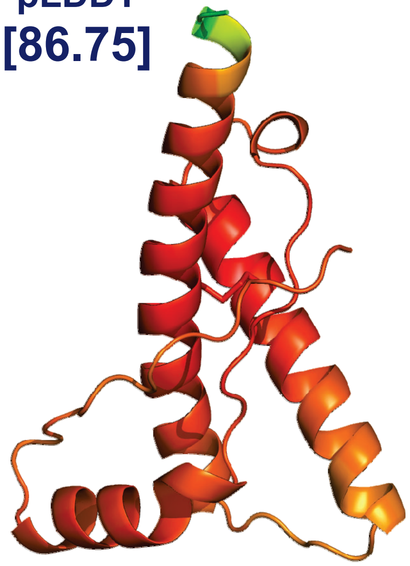

Protein code: 4093

Black-capped squirrel monkey

GenBank: BK064035

Saimiri boliviensis

Primates	35/45
Cebidae	7/8

P34%

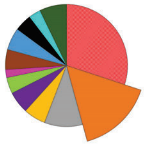

0-10

0.9

β2-α2 LOOP
SNQNNF
273

100 — β1 — α1 — 3_{10} — β2 — 3_{10} — α2 — α3 — 231

WNKPSKPKTNMKHMAGAAAAGAVVGGLGGYMLGSAMSRPLIHFGNDYEDRYYRENMYRYPSQVYYKPVDQYSNQNNFVHDCVNVTIKQHTVTTTTKGENFTETDVKMMERVVEQMCITQYEKESQAYYQRGSS-
I V M W N N R N I V Q EGR

PrP sequence differs by 11 amino acids from the bank vole PrP (see in red)

No deletions
No insertions
No polymorphic variants found

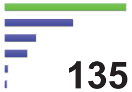

135

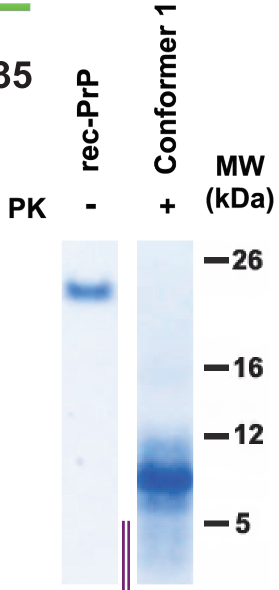

218 AA

1
Conformers

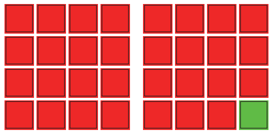

Tm^{Exp}: ND

ΔΔG: -5.19

In vitro studies

TgVole (1x) — Not tested

Black-capped squirrel monkey — Not tested

In vivo studies

TgVole (1x) — Not tested

Black-capped squirrel monkey — Not tested

pLDDT
[86.67]

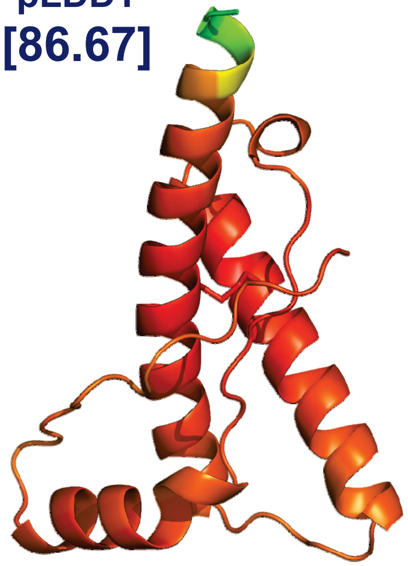

Distinct primary sequence across species

Protein code: 1975

Squirrel monkey

GenBank: U15165

Saimiri sciureus

Primates	35/45
Cebidae	7/8

P29%

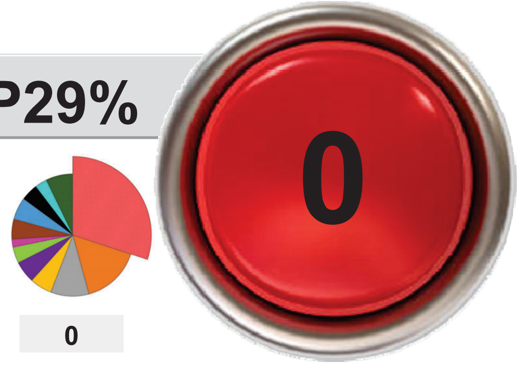

0

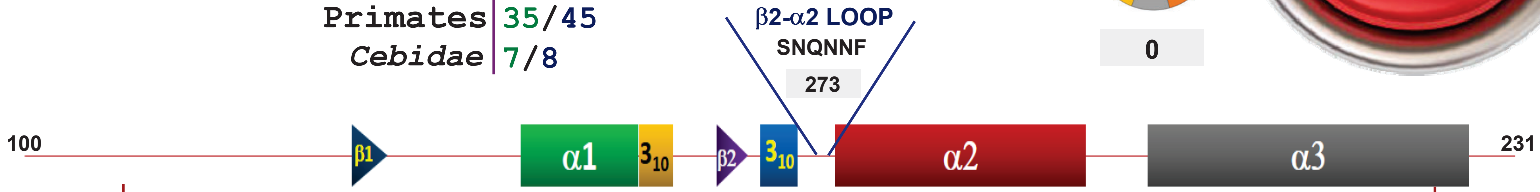

WNKPSKPKTNMKHMAGAAAAGAVVGGLGGYMLGSAMSRPLIHFGNDYEDRYYRENMYRYPSQVYYRPVDQYSNQNNFVHDCVNVTIKQHTVTTTTKGENFTETDVKMMERVVEQMCITQYEKESQAYYQRGSS-

I V M W N N N I V Q EGR

PrP sequence differs by 10 amino acids from the bank vole PrP (see in red)

No deletions

No insertions

It shows polymorphic variants

Distinct primary sequence across species

Tm^{Exp}: ND

ΔΔG: -5.63

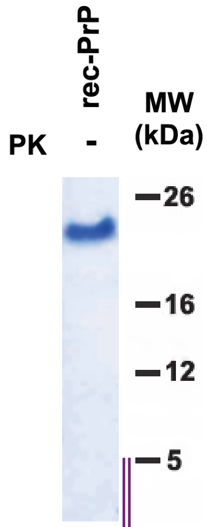

218 AA

NO MISFOLDING

0

Conformers

In vitro studies

NOT APPLICABLE

In vivo studies

NOT APPLICABLE

pLDDT [84.64]

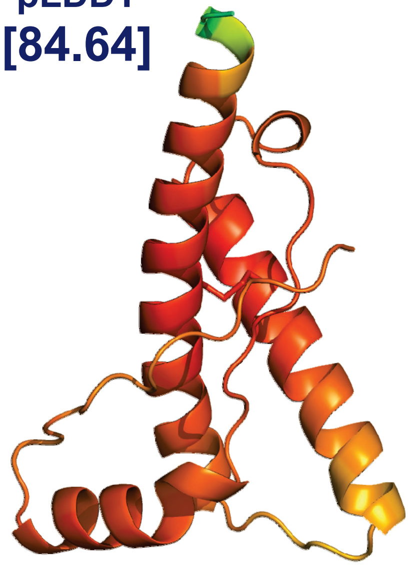

Protein code: 0362

Philippine tarsier

GenBank: XM_021710036

Tarsius syrichta

Primates 35/45
Tarsiidae 1/1

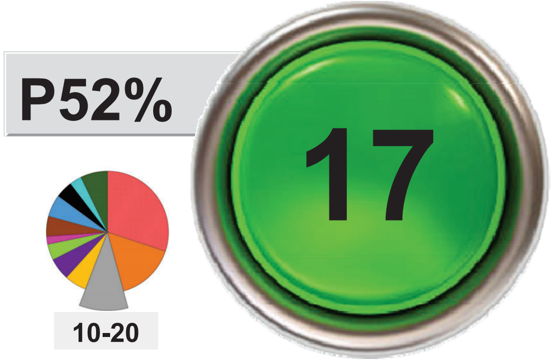

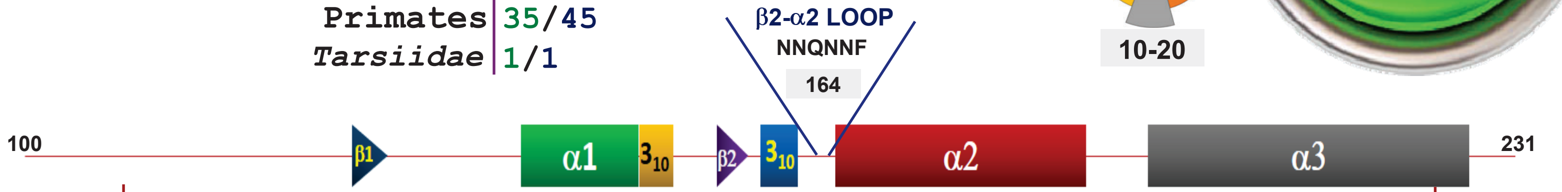

WSKPSKPKTNMKHVAGAAAAGAVVGGLGGYMLGSAMSRPLIHFGNDYEDRYYRENMYRYPNQVYYRPMDQYNNQNNFVHDCVNITIKQHTVTTTTKGENFTETDIKMMERVVEQMCITQYQKEYQASYHSGSN-
N I M W N V V V S Y EGR S

PrP sequence differs by 8 amino acids from the bank vole PrP (see in red)

No deletions
No insertions
No polymorphic variants found

1 species sharing the same primary sequence

Horsfield's tarsier
Cephalopachus bancanus

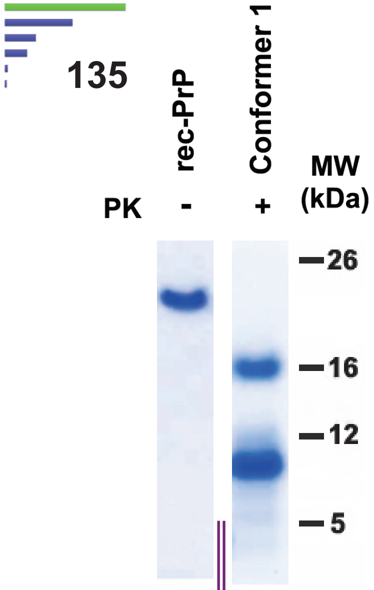

211 AA

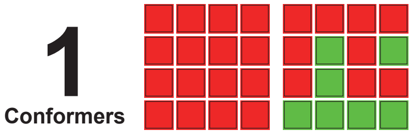

Tm^{Exp}: ND

ΔΔG: -9.49

In vitro studies

TgVole (1x) Not tested

Philippine tarsier Not tested

In vivo studies

TgVole (1x) Not tested

Philippine tarsier Not tested

pLDDT [84.65]

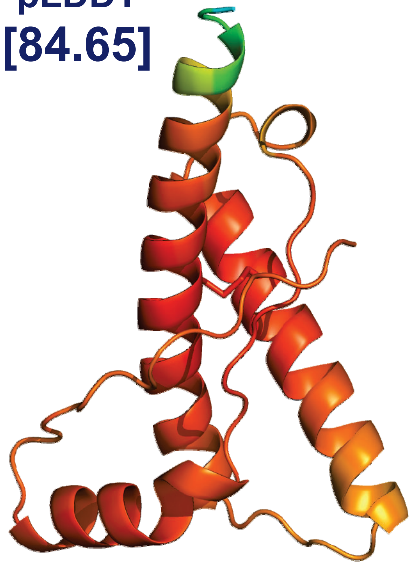

Protein code: 0943

Gelada

GenBank: XM_025399776

P78%

67.9

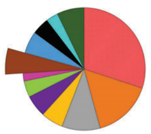

60-70

Theropithecus gelada

Primates	35/45
Cercopithecidae	7/7

β2-α2 LOOP
SNQNNF
273

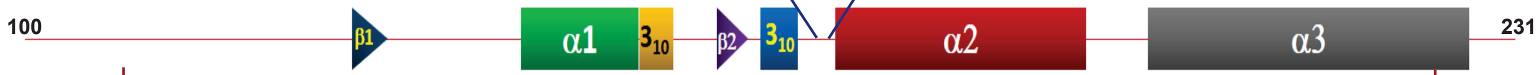

WHKPSKPKTSMKHMAGAAAAGAVVGGLGGYMLGSAMSRPLIHFGNDYEDRYYRENMYRYPNQVYYRPVDQYSNQNNFVHDCVNITIKQHTVTTTTKGENFTETDVKMMERVVEQMCITQYQKESQAYYQRGSS-
N NI V M W N N V EGR

PrP sequence differs by 8 amino acids from the bank vole PrP (see in red)

No deletions
No insertions
No polymorphic variants found

Distinct primary sequence across species

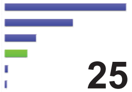

25

211 AA

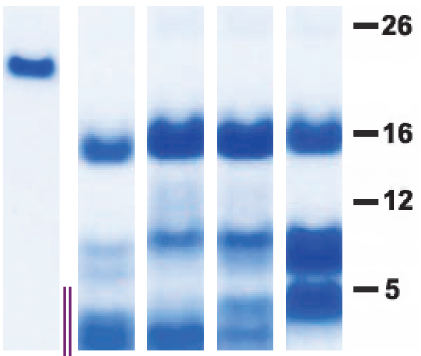

4
Conformers

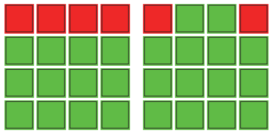

Tm[Exp]: ND

ΔΔG: -8.22

pLDDT
[85.79]

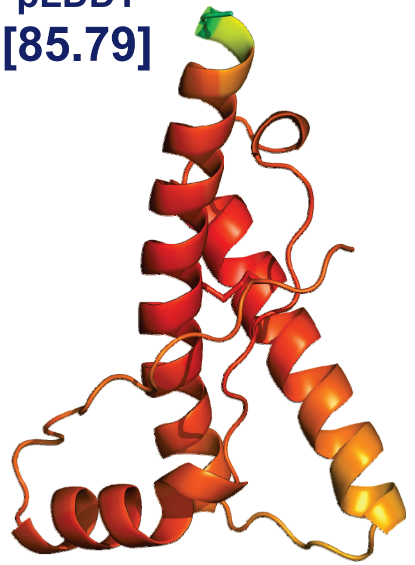

In vitro studies

TgVole (1x)	Not tested
Gelada	Not tested

In vivo studies

TgVole (1x)	Not tested
Gelada	Not tested

Protein code: 1204

Elephant

GenBank: EU588731

P29%

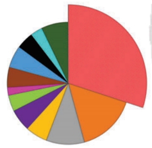

0

0

Loxodonta africana

Proboscidea 0/1

Elephantidae 0/1

Asian elephant
Elephas maximus

1 species sharing the same primary sequence

β2-α2 LOOP
SNENNF
2

100 β1 α1 3_{10} β2 3_{10} α2 α3 231

WNKPSKPKTNLKHVAGAAAAGAVVGGLGGYMLGSAMSRPLIHFGNDYEDRYYRENMYRYPNQVYYRPVNEYSNENNFVHDCVNITVKQHTTTTITKGENFTETDTKIMERMVEQMCVTQYRKEFQAYQRGSS-

I M W N DQ N Q I V T V M V Q S YE R S

PrP sequence differs by 16 amino acids from the bank vole PrP (see in red)

No deletions

No insertions

No polymorphic variants found

Tm^{Exp}: ND

ΔΔG: ND

rec-PrP

PK -

MW (kDa)

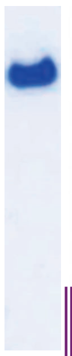

26
16
12
5

212 AA

NO MISFOLDING

0
Conformers

In vitro studies

NOT APPLICABLE

In vivo studies

NOT APPLICABLE

pLDDT
[88.02]

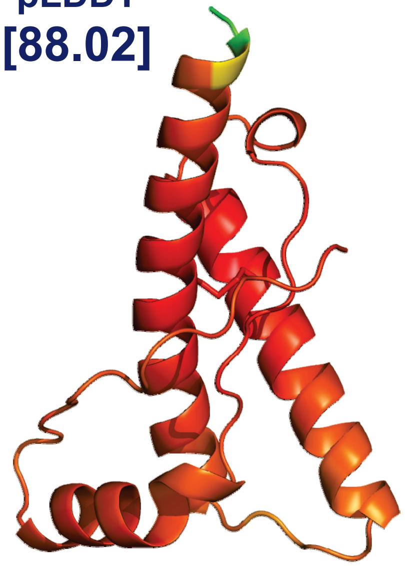

Protein code: 0930

Cairo spiny mouse

GenBank: EF467171

Acomys cahirinus

Rodentia 81/101
Muridae 16/18

P90%

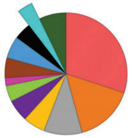

90-100

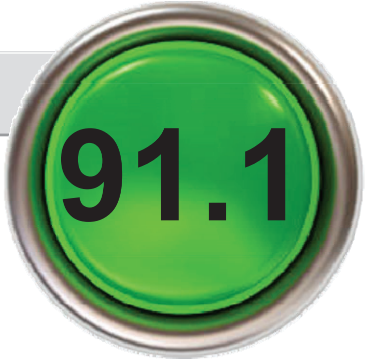

β2-α2 LOOP
SNQNNF
273

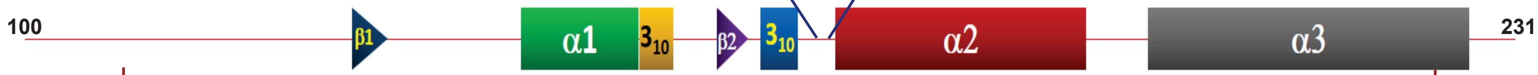

WGKPSKPKTNMKHVAGAAAAGAVVGGLGGYMLGSAMSRPMIHFGNDWEDRYYRENMYRYPNQVYYRPVDQYSNQNNFVHDCVNITIKQHTVTTTTKGENFTETDVKMMERVVEQMCVTQYQKESQAYYDGRRS-

N I N N E S

PrP sequence differs by 3 amino acids from the bank vole PrP (see in red)

No deletions
No insertions
No polymorphic variants found

Distinct primary sequence across species

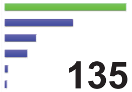

135

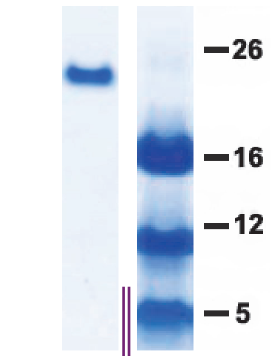

211 AA

1
Conformers

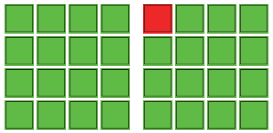

Tm^{Exp}: ND

ΔΔG: -2.04

In vitro studies

TgVole (1x) Not tested

Cairo spiny mouse Not tested

In vivo studies

TgVole (1x) Not tested

Cairo spiny mouse Not tested

pLDDT
[86.25]

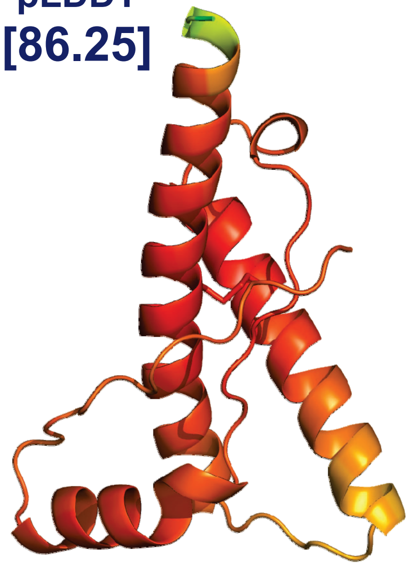

Protein code: 1370

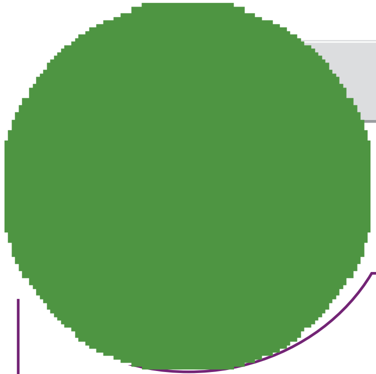

Gobi jerboa

GenBank: BK064143

Allactaga bullata

Rodentia 81/101

Dipodidae 3/3

P84%

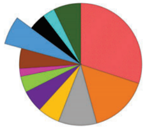

70-80

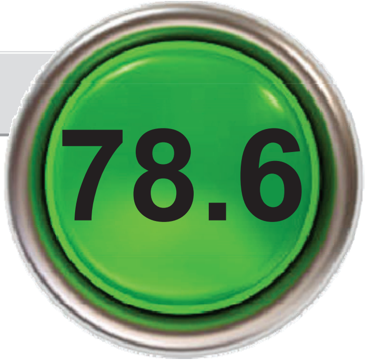

β2-α2 LOOP

NNQNNF

164

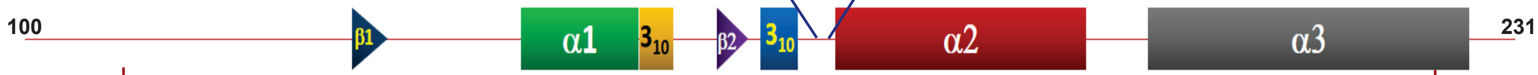

WNKPNKPKTSMKHMAGAAAAGAVAGGLGGYMLGSAMSRPSMNFGNDYEDRYYRENMNRYPSQVYYKPVDQYNNQNNFVHDCVNITVKQHTVTTTTKGENFTETDVKIMERVVEQMCITQYQKESQAYLEGRRS-

S NI V V MIH W N R I M V Y S

PrP sequence differs by 13 amino acids from the bank vole PrP (see in red)

No deletions

No insertions

No polymorphic variants found

Distinct primary sequence across species

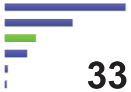

33

rec-PrP | Conformer 1 | Conformer 2 | Conformer 3 | MW (kDa)

PK - + + +

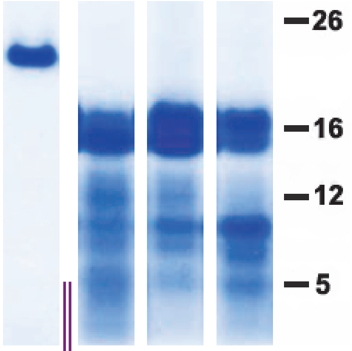

213 AA

3

Conformers

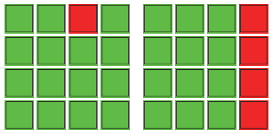

Tm[Exp]: ND

ΔΔG: -10.12

In vitro studies

TgVole (1x) Not tested

Gobi jerboa Not tested

In vivo studies

TgVole (1x) Not tested

Gobi jerboa Not tested

pLDDT

[85.38]

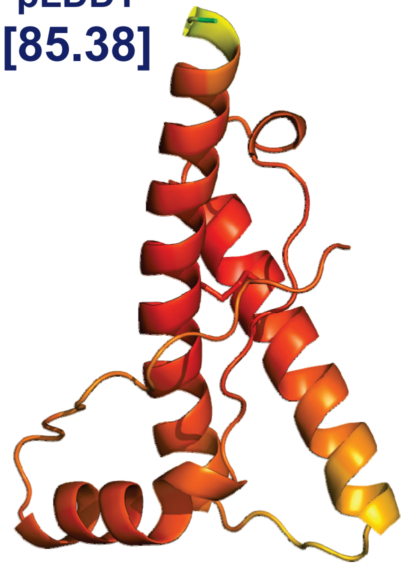

Protein code: 3330

Mountain beaver

GenBank: BK064070

Aplodontia rufa

Rodentia 81/101
Aplodontiidae 1/1

P79%

71.1

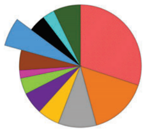

70-80

β2-α2 LOOP
SNQNNF
273

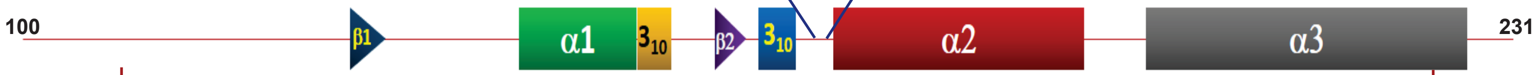

WNKPKKPKTNMKHVAGAAAAGAVVGGLGGYMLGSAMSRPLIHFGNDYEDRYYRENMYRYPNQVYYKPVDQYSNQNNFVHDCVNITVKQHTVTTTTKGENFTETDIKIMERVVEQMCITQYQQEAQAYYQRGSS-

S I M W N R N I V M V K S EGR

PrP sequence differs by 12 amino acids from the bank vole PrP (see in red)

No deletions
No insertions
No polymorphic variants found

Distinct primary sequence across species

135

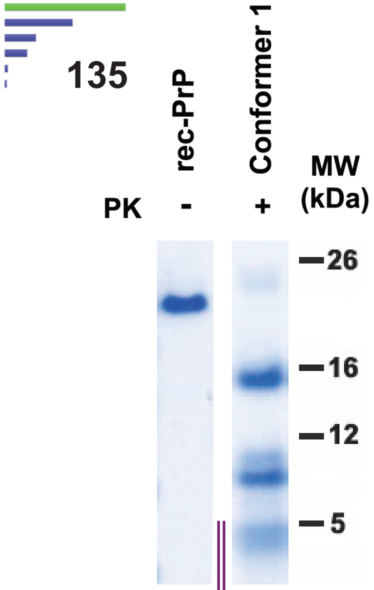

213 AA

1
Conformers

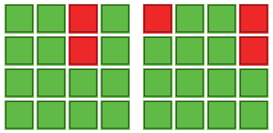

Tm^{Exp}: ND

ΔΔG: -11.28

In vitro studies

TgVole (1x) — Not tested

Mountain beaver — Not tested

In vivo studies

TgVole (1x) — Not tested

Mountain beaver — Not tested

pLDDT
[85.02]

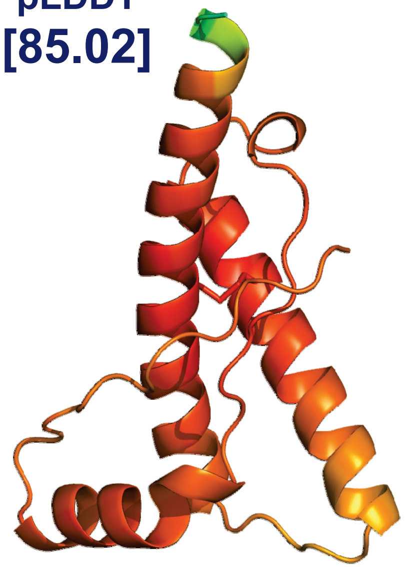

Protein code: 3247

Steppe field mouse

GenBank: KF466953

Apodemus fulvipectus

Rodentia 81/101
Muridae 16/18

P61%

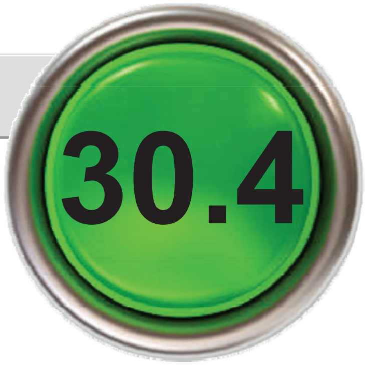

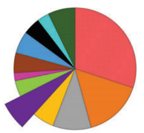

30-40

β2-α2 LOOP
SNQNNF
273

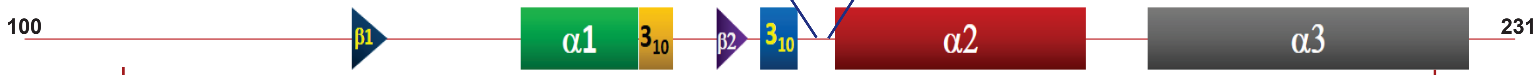

WNKPSKPKTNLKHVAGAAAAGAVVGGLGGYMLGSAMSRPMIHFGNDWEDRYYRENMYRYPNQVYYRPVDQYSNQNNFVHDCVNITIKQHTVTTTTNGENFTETDVKMMERVVDQMCVTQYHKESQAYYDGRRS-
I N N E Q E S

PrP sequence differs by 5 amino acids from the bank vole PrP (see in red)

No deletions
No insertions
No polymorphic variants found

Distinct primary sequence across species

135

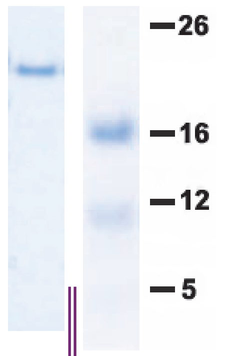

211 AA

1
Conformers

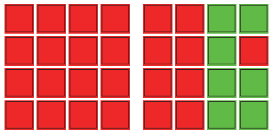

Tm^{Exp}: ND

ΔΔG: -11.57

pLDDT
[86.31]

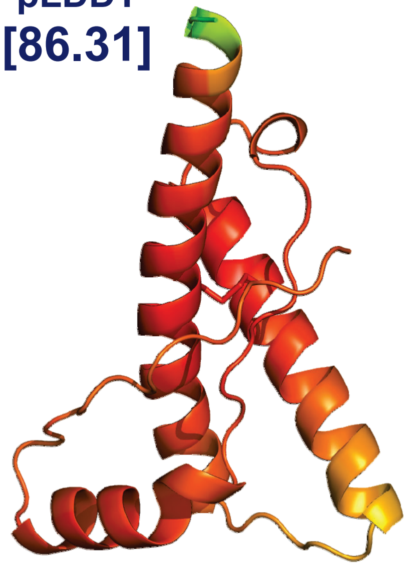

In vitro studies

TgVole (1x) Not tested

Steppe field mouse Not tested

In vivo studies

TgVole (1x) Not tested

Steppe field mouse Not tested

Protein code: 1395

African grass rat

GenBank: XM_034493892

Arvicanthis niloticus

Rodentia 81/101
Muridae 16/18

P100%

100

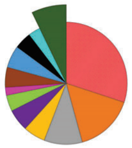

100

β2-α2 LOOP
SNQNSF
36

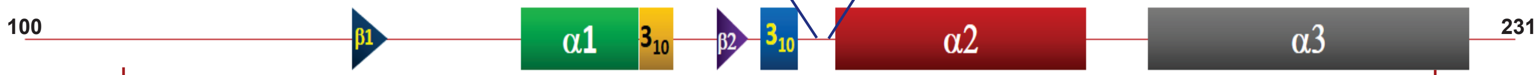

WNKPSKPKTNLKHVAGAAAAGAVVGGLGGYMLGSAMSRPMIHFGNDWEDRYYRENMYRYPNQVYYRPVDQYSNQNSFVHDCVNITIKQHTVTTTTKGENFTETDVKMMERVVEQMCTTQYQKESQAYYDGRRS-
I N N N V E S

PrP sequence differs by 5 amino acids from the bank vole PrP (see in red)

No deletions
No insertions
No polymorphic variants found

76

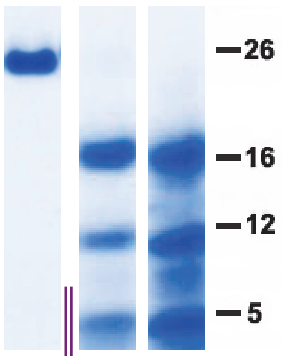

211 AA

2
Conformers

Tm^{Exp}: ND

ΔΔG: -5.57

In vitro studies

TgVole (1x) Not tested

African grass rat Not tested

In vivo studies

TgVole (1x) Not tested

African grass rat Not tested

pLDDT
[86.26]

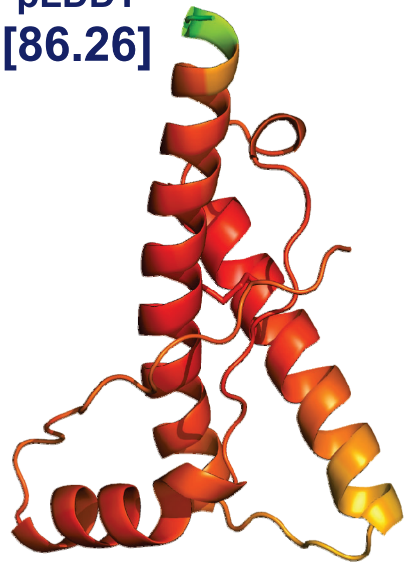

Distinct primary sequence across species

Protein code: 2544

European water vole

GenBank: BK063953

P60% **27.7**

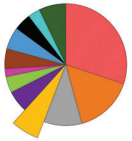

20-30

Arvicola amphibius

Rodentia 81/101
Cricetidae 25/27

1 species sharing the same primary sequence

Gerbe's vole
Microtus gerbei

β2-α2 LOOP
NNQNNF
164

100 β1 α1 3_{10} β2 3_{10} α2 α3 231

WNKPSKPKTNMKHVAGAAAAGAVVGGLGGYMLGSAMSRPMIHFGNDWEDRYYRENMNRYPNQVYYRPVDQYNNQNNFVHDCVNITIKQHTVTTTTKGENFTETDVKMMERVVEQMCVTQYQKESQAYYEGRYS-
I S

PrP sequence differs by 1 amino acids from the bank vole PrP (see in red)

No deletions
No insertions
No polymorphic variants found

135

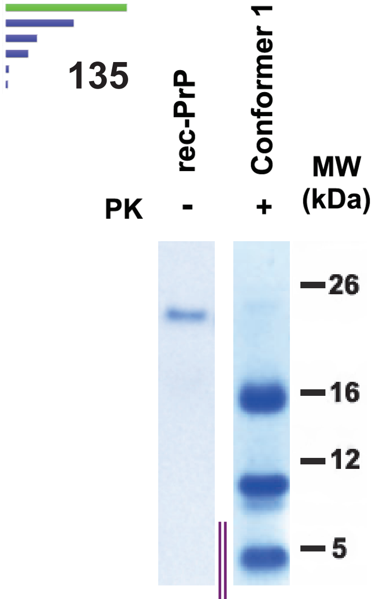

210 AA

1
Conformers

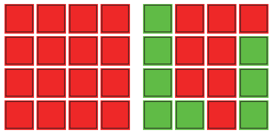

Tm^{Exp}: ND

ΔΔG: -0.51

In vitro studies

TgVole (1x) Not tested

European water vole Not tested

In vivo studies

TgVole (1x) Not tested

European water vole Not tested

pLDDT [88.37]

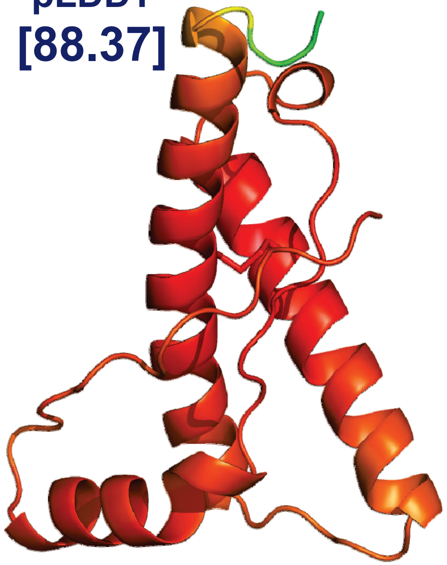

Protein code: 3080

Greater bandicoot rat

GenBank: KF466950

Bandicota indica

Rodentia 81/101
Muridae 16/18

P100%

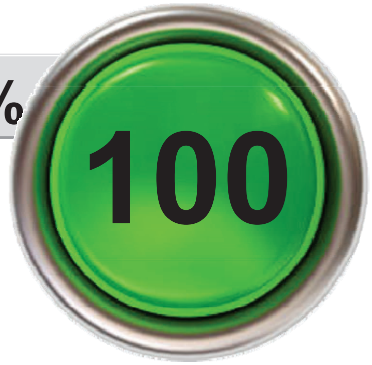

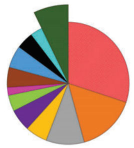

100

β2-α2 LOOP
SNQNNF
273

WNKPSKPKTNLKHVAGAAAAGAVVGGLGGYMLGSAMSRPMLHFGNDWEDRYYRENMYRYPNQVYYRPVDQYSNQNNFVHDCVNITIKQHTVTTTTKGENFTETDVKMMERVVEQMCVTQYQKESQAYYDGRRS-

I I N N E S

PrP sequence differs by 4 amino acids from the bank vole PrP (see in red)

No deletions
No insertions
No polymorphic variants found

5 species sharing the same primary sequence

Savile's bandicoot rat *Bandicota savilei*

Small white-toothed rat *Berylmys berdmorei*

Edwards's long-tailed giant rat *Leopoldamys edwardsi*

Long-tailed giant rat *Leopoldamys sabanus*

Polynesian rat *Rattus exulans*

135

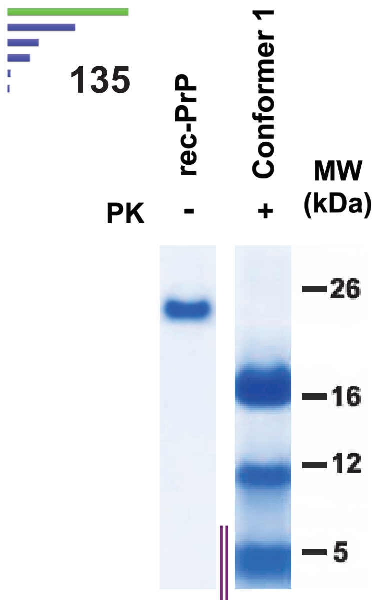

210 AA

1
Conformers

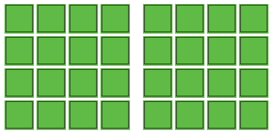

Tm[Exp]: ND

ΔΔG: -9.35

In vitro studies

TgVole (1x) — Not tested

Greater bandicoot rat — Not tested

In vivo studies

TgVole (1x) — Not tested

Greater bandicoot rat — Not tested

pLDDT
[86.4]

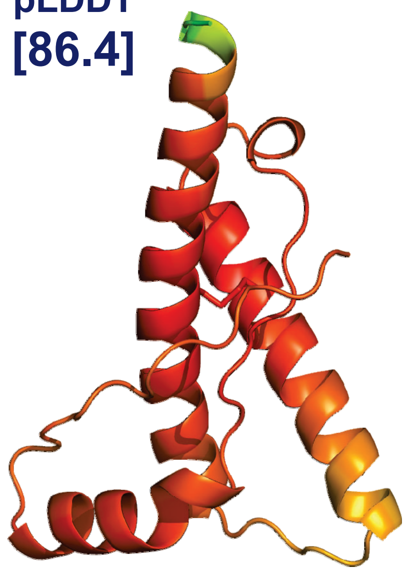

Protein code: 1340

Desmarest's hutia

GenBank: BK064046

Capromys pilorides

Rodentia 81/101
Capromyidae 0/1

P29%

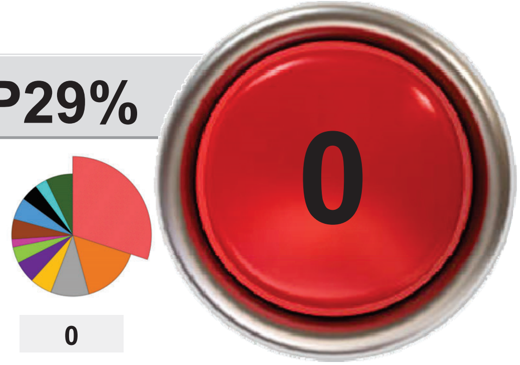

β2-α2 LOOP
SNQNSF
36

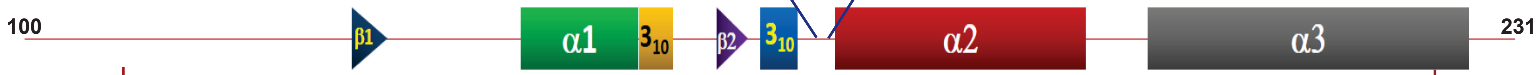

WNKPSKPKTNMKHVAGAAAAGAVVGGLGGYMLGSAMSRPLIHFGNDYEDRYYRENMYRYPNQVYYRPVDQYSNQNSFVQDCVNITIKQHTVTTTTKGENFTETDVKIMERVLQQMCTTQYQKESQAYYYHGRAG-
I M W N N N H M VE V EGRSS

PrP sequence differs by 11 amino acids from the bank vole PrP (see in red)

No deletions
No insertions
No polymorphic variants found

Distinct primary sequence across species

Tm^{Exp}: ND

ΔΔG: ND

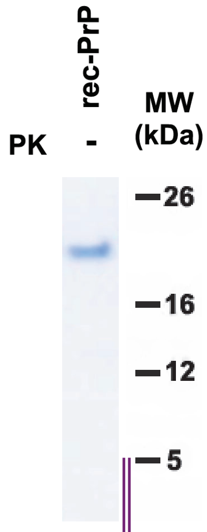

202 AA

NO MISFOLDING

0
Conformers

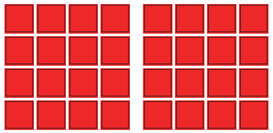

In vitro studies

NOT APPLICABLE

In vivo studies

NOT APPLICABLE

pLDDT
[83.65]

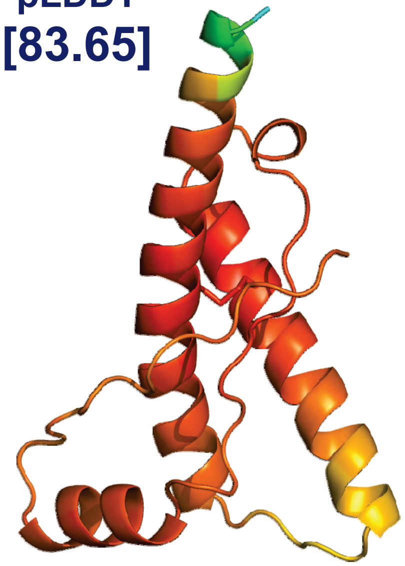

Protein code: 3213

North American beaver

GenBank: XM_020184027

Castor canadensis

Rodentia 81/101
Castoridae 1/1

P84% **78.2**

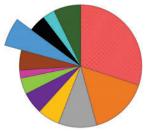

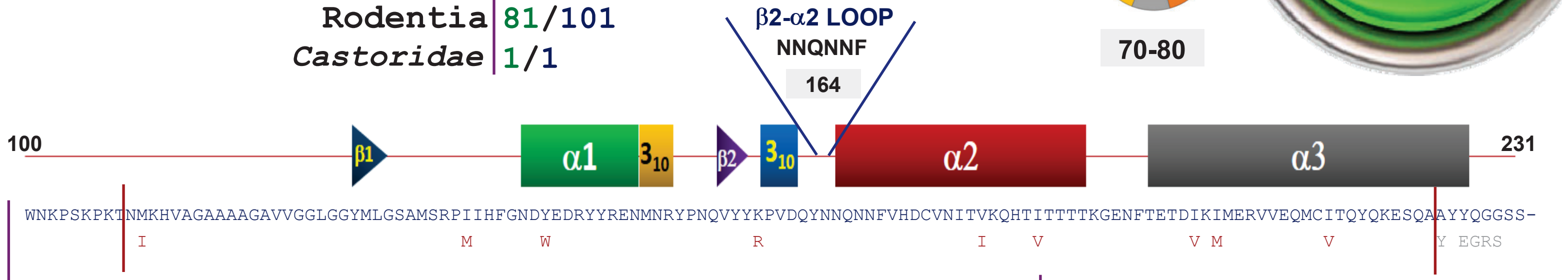

PrP sequence differs by 9 amino acids from the bank vole PrP (see in red)

No deletions
No insertions
No polymorphic variants found

Distinct primary sequence across species

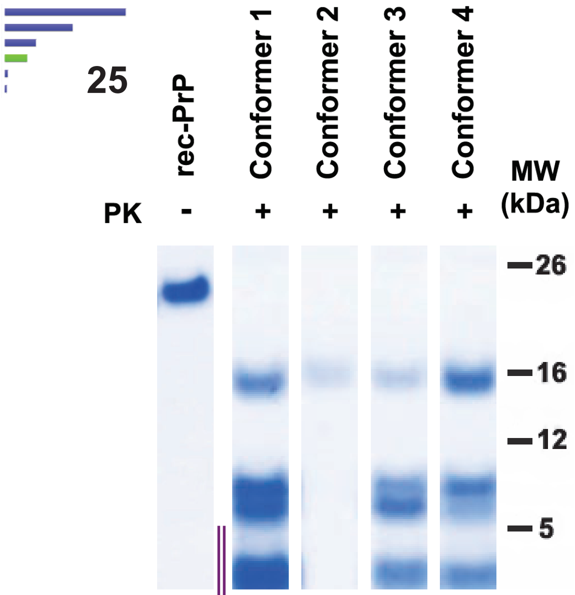

225 AA

4 Conformers

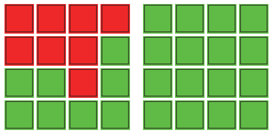

Tm[Exp]: ND
ΔΔG: ND

pLDDT [84.13]

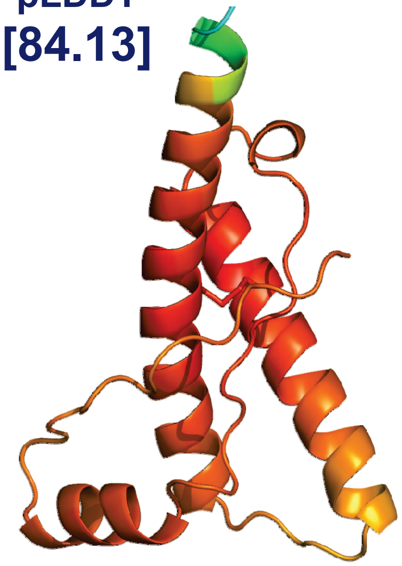

In vitro studies

TgVole (1x) — Not tested
North American beaver — Not tested

In vivo studies

TgVole (1x) — Not tested
North American beaver — Not tested

Protein code: 0940

Brazilian guinea pig

GenBank: KM357834

Cavia aperea

Rodentia 81/101
Caviidae 1/5

Distinct primary sequence across species

P29%

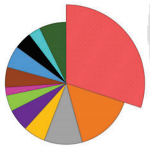

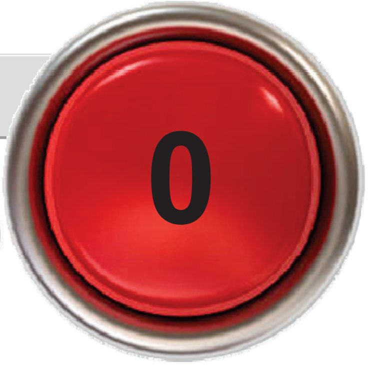

0

β2-α2 LOOP
SNQNSF
36

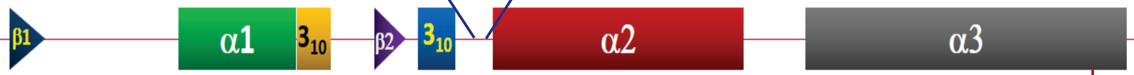

WNKPSKPKTNMKHMAGAAAAGAVVGGLGGYMLGSAMSRPLIHFGSDYEDRYYRENMYRYPNQVYYKPVDQYSNQNSFVQDCVNITIKQHTVTTTTKGENFTETDVKIMERVLEQMCTTQYQKESQAYYYHGRAG-

I V M N W N R N N H M V V EGRSS

PrP sequence differs by 13 amino acids from the bank vole PrP (see in red)

No deletions
No insertions
No polymorphic variants found

Tm^{Exp}: ND

ΔΔG: ND

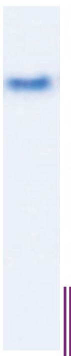

202 AA

NO MISFOLDING

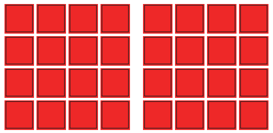

0
Conformers

In vitro studies

NOT APPLICABLE

In vivo studies

NOT APPLICABLE

pLDDT
[84.41]

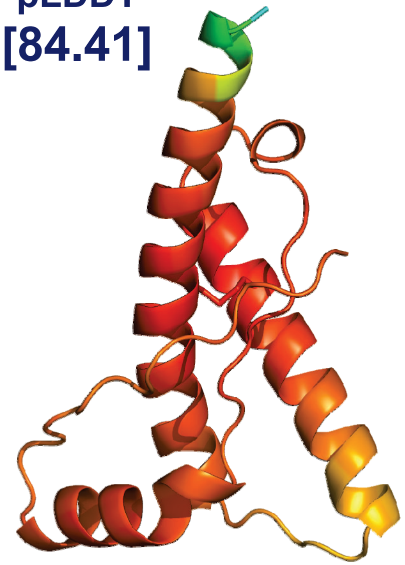

Protein code: 1470

Guinea pig

GenBank: XM_003476602

Cavia porcellus

Rodentia 81/101

Caviidae 1/5

P29%

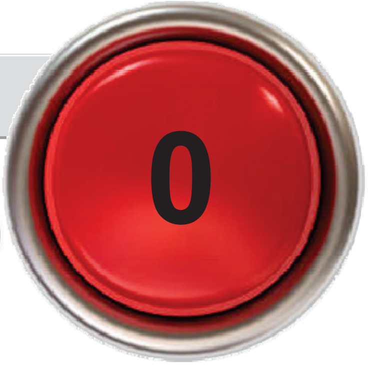

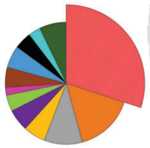

0

β2-α2 LOOP

SNQNSF

36

100 β1 α1 3_{10} β2 3_{10} α2 α3 231

WNKPSKPKTNMKHMAGAAAAGAVVGGLGGYMLGSAMSRPLIHFGSDYEDRYYRENMYRYPNQVYYKPVDQYSNQNSFVQDCVNITIKQHTVTTTTKGENFTETDVKIMERVLEQMCTTQYQKESQAYYYHGRA-

I V M N W N R N N H M V V EGRSS

PrP sequence differs by 13 amino acids from the bank vole PrP (see in red)

No deletions

No insertions

No polymorphic variants found

Distinct primary sequence across species

Tm^{Exp}: ND

ΔΔG: -6.62

PK -

rec-PrP

MW (kDa)

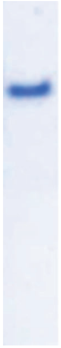

26

16

12

5

201 AA

NO MISFOLDING

0

Conformers

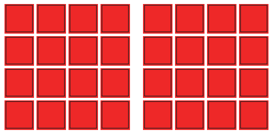

In vitro studies

NOT APPLICABLE

In vivo studies

NOT APPLICABLE

pLDDT

[85.43]

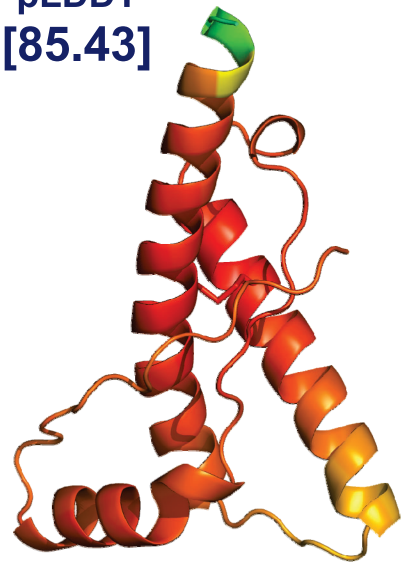

Protein code: 0815

Montane guinea pig

GenBank: BK063938

P72%

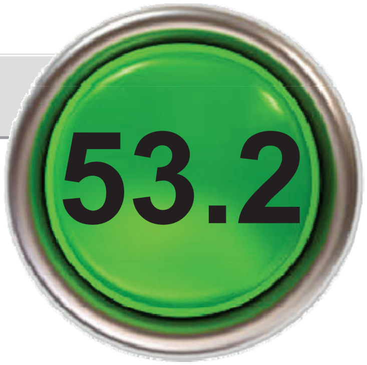

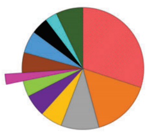

50-60

Cavia tschudii

Rodentia 81/101
Caviidae 1/5

β2-α2 LOOP
SNQNSF
36

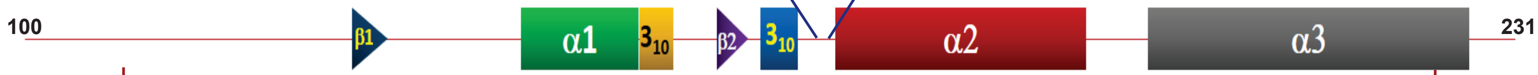

WNKPSKPKTNMKHVAGAAAAGAVVGGLGGYMLGSAMSRPLIHFGSDYEDRYYRENMYRYPNQVYYKPVDQYSNQNSFVQDCVNITIKQHTVTTTTKGENFTETDVKIMERVLEQMCTTQYQKESQAYYYHGRAG-
I M N W N R N N H M V V EGRSS

PrP sequence differs by 12 amino acids from the bank vole PrP (see in red)

No deletions
No insertions
No polymorphic variants found

Distinct primary sequence across species

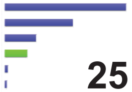

25

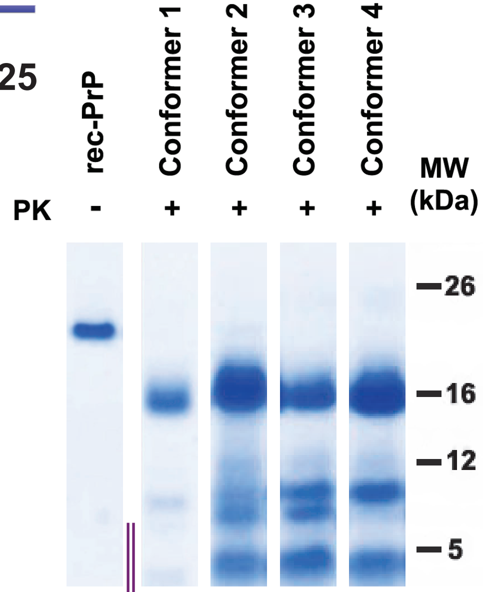

202 AA

4
Conformers

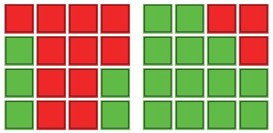

Tm[Exp]: ND
ΔΔG: ND

In vitro studies

TgVole (1x) — Propagate
Montane guinea pig — Ongoing

In vivo studies

TgVole (1x) — Ongoing
Montane guinea pig — Not tested

pLDDT
[84.32]

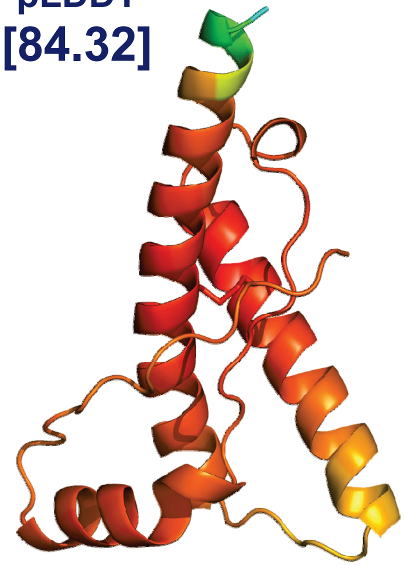

Protein code: 3061

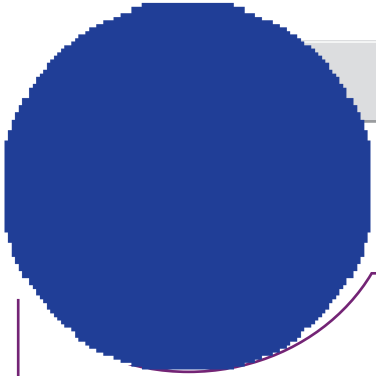

Long-tailed chinchilla

GenBank: XM_005380818

P54%

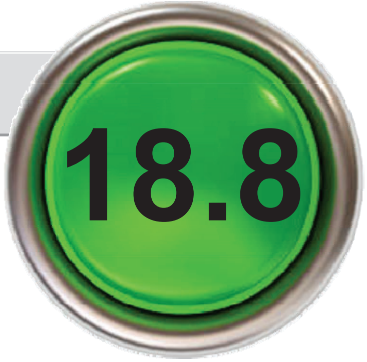

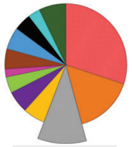

10-20

Chinchilla lanigera

Rodentia	81/101
Chinchillidae	1/1

β2-α2 LOOP
NSQNSF
5

100

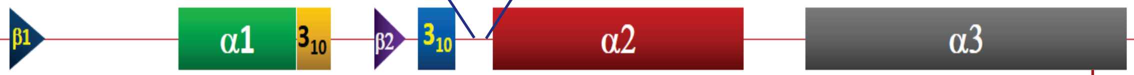

231

WNKPSKPKTNMKHVAGAAAAGAVVGGLGGYMLGSAMSRPLIHFSNDYEDRYYRENMYRYPNQVYYKPVDQYNSQNSFVQDCVNITIKQHTVTTTTKGENFTETDVKIMERVLEQMCTTQYQKESRAYYYHGRAG-

I M G W N R N N H M V V Q EGRSS

PrP sequence differs by 13 amino acids from the bank vole PrP (see in red)

No deletions
No insertions
No polymorphic variants found

Distinct primary sequence across species

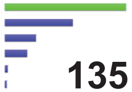

135

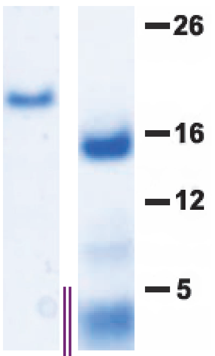

202 AA

1
Conformers

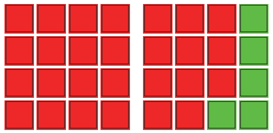

Tm^Exp: ND

ΔΔG: ND

***In vitro* studies**

Propagate
TgVole (1x)

Ongoing
Long-tailed chinchilla

***In vivo* studies**

Ongoing
TgVole (1x)

Not tested
Long-tailed chinchilla

pLDDT
[83.93]

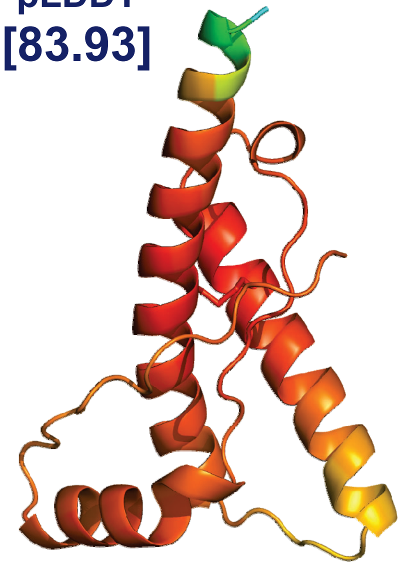

Protein code: 1136

Brazilian porcupine

GenBank: OR47272

Coendou prehensilis

Rodentia	81/101
Erethizontidae	0/2

P29%

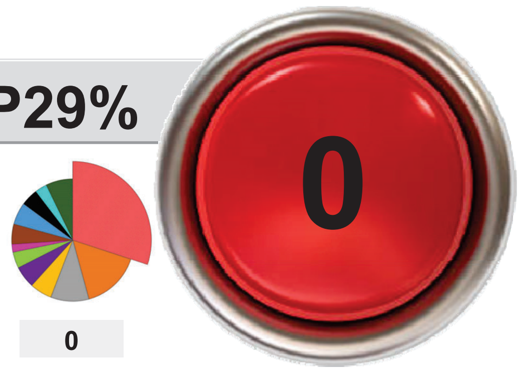

β2-α2 LOOP
SGQKSF
2

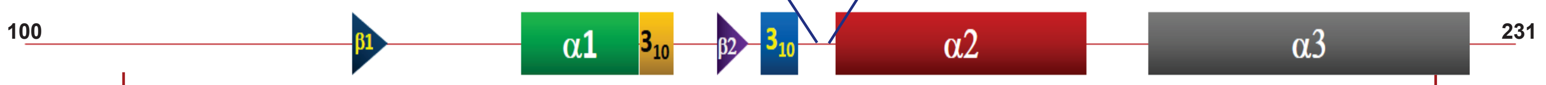

WNKPSKPKTNMKHMAGAAAAGAVVGGLGGYMLGSAMSRPLIHFGSDYEDRYYRENMYRYPDQVYYRPVDQYSGQKSFVQDCVNITIKQHTVTTTTKGENFTETDVKIMERVLEQMCTTQYQKESQAYYYRGRAG-

I V M N W N N NN NN H M V V EGRSS

PrP sequence differs by 15 amino acids from the bank vole PrP (see in red)

No deletions

No insertions

No polymorphic variants found

Distinct primary sequence across species

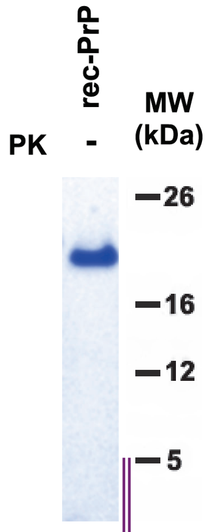

202 AA

NO MISFOLDING

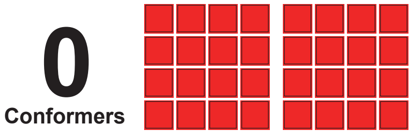

In vitro studies

NOT APPLICABLE

In vivo studies

NOT APPLICABLE

Tm^{Exp}: ND

ΔΔG: ND

pLDDT [86.71]

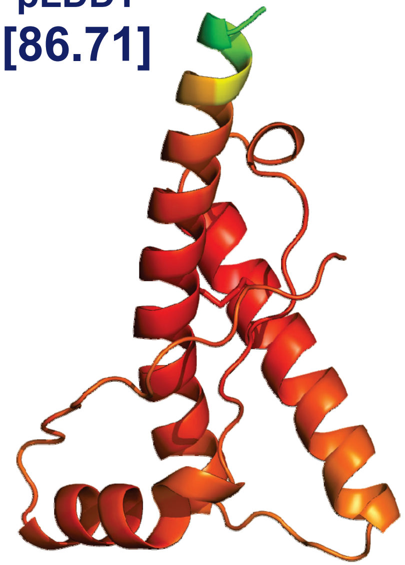

Protein code: 3785

Gambian pouched rat

GenBank: BK063941

Cricetomys gambianus

Rodentia 81/101

Nesomyidae 1/1

P40%

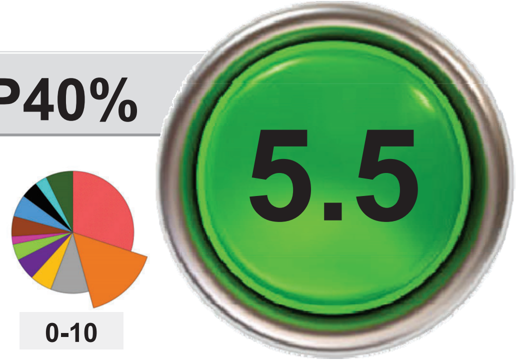

1 species sharing the same primary sequence

Southern giant pouched rat
Cricetomys ansorgei

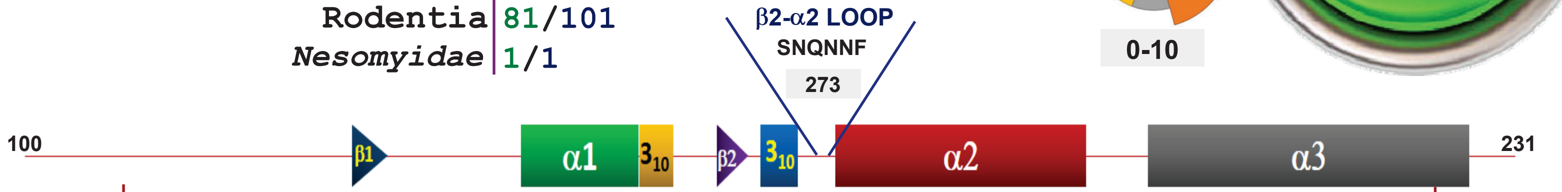

WNKPSKPKTNMKQMAGAAAAGAVVGGLGGYMLGSAMSRPMIHFGSDWEDRYYRENMYRYPNQVYYRPVDQYSNQNNFVHDCVNITIKQHTVTTTTKGENFTETDVKMMERVVEEMCTTQYQKESQAYYEGRRS-

I HV N N N Q V S

PrP sequence differs by 8 amino acids from the bank vole PrP (see in red)

No deletions

No insertions

No polymorphic variants found

135

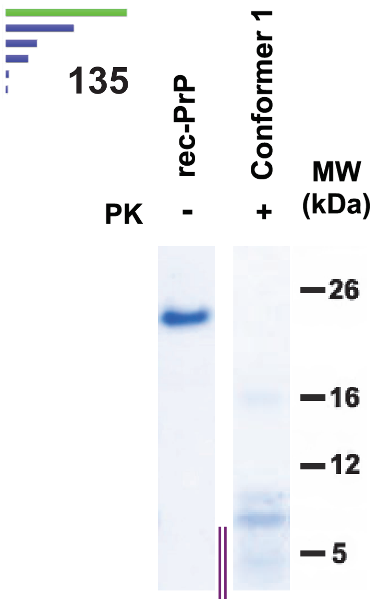

214 AA

1

Conformers

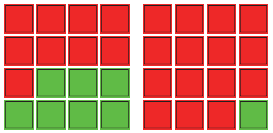

Tm^{Exp}: ND

ΔΔG: -4.31

In vitro studies

Not tested
TgVole (1x)

Not tested
Gambian pouched rat

In vivo studies

Not tested
TgVole (1x)

Not tested
Gambian pouched rat

pLDDT [88.39]

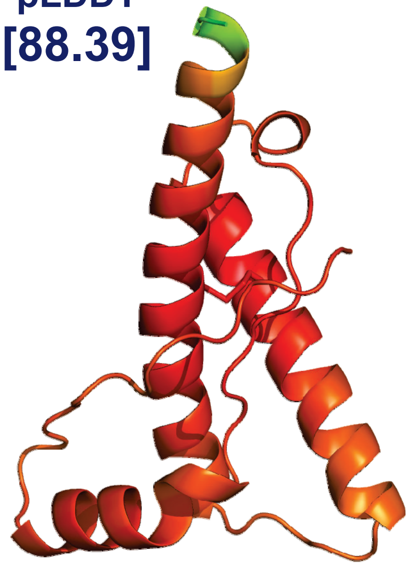

Protein code: 3067

Chinese hamster

GenBank: M33958

Cricetulus barabensis

Rodentia 81/101
Cricetidae 25/27

P88%

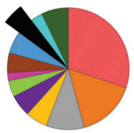

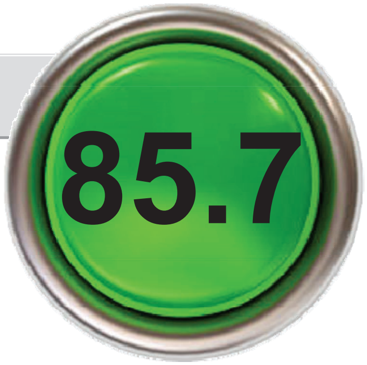

β2-α2 LOOP
NNQNNF
164

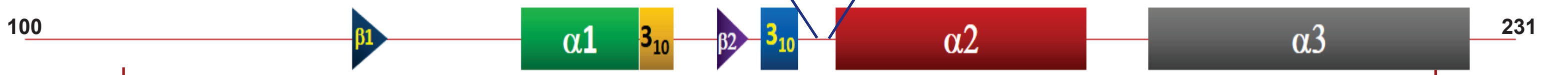

WNKPSKPKTNMKHVAGAAAAGAVVGGLGGYMLGSAMSRPMLHFGNDWEDRYYRENMNRYPNQVYYRPVDQYNNQNNFVHDCVNITIKQHTVTTTTKGENFTETDVKMMERVVEQMCVTQYQKESQAYYDGRRS-
I I E S

PrP sequence differs by 2 amino acids from the bank vole PrP (see in red)

No deletions
No insertions
No polymorphic variants found

Distinct primary sequence across species

76

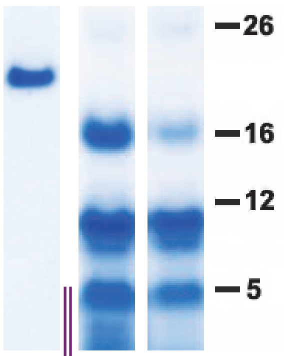

211 AA

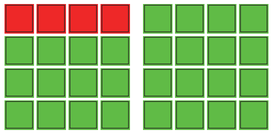

Tm^{Exp}: ND

ΔΔG: -4.97

In vitro studies

Not tested
TgVole (1x)

Not tested
Chinese hamster

In vivo studies

Not tested
TgVole (1x)

Not tested
Chinese hamster

pLDDT
[86.17]

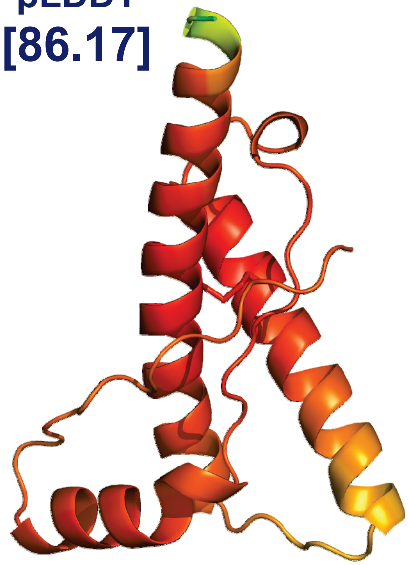

Protein code: 0835

Armenian hamster

GenBank: M33959

Cricetulus migratorius

`Rodentia` 81/101
`Cricetidae` 25/27

P91% **92.9**

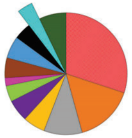

90-100

β2-α2 LOOP
NNQNNF
164

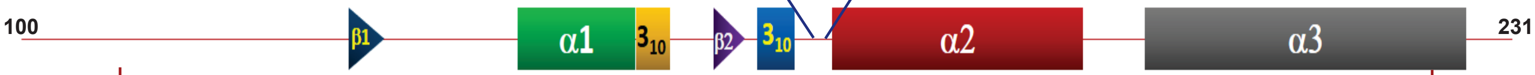


```
WNKPNKPKTSMKHMAGAAAAGAVVGGLGGYMLGSAMSRPMLHFGNDWEDRYYRENMNRYPNQVYYRPVDQYNNQNNFVHDCVNITIKQHTVTTTTKGENFTETDVKMMERVVEQMCVTQYQKESQAYYDGRRS-
    S    NI  V                          I                                                                                            E  S
```

PrP sequence differs by 4 amino acids from the bank vole PrP (see in red)

No deletions
No insertions
No polymorphic variants found

Distinct primary sequence across species

135

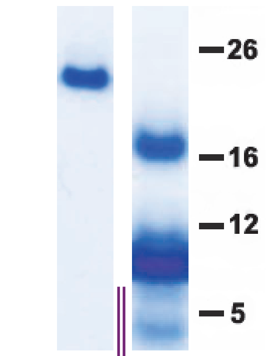

211 AA

1 Conformers

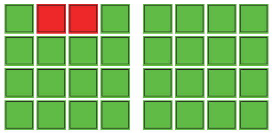

Tm^{Exp}: ND

ΔΔG: -7.18

In vitro studies

TgVole (1x) Not tested

Armenian hamster Not tested

In vivo studies

TgVole (1x) Not tested

Armenian hamster Not tested

pLDDT [86.22]

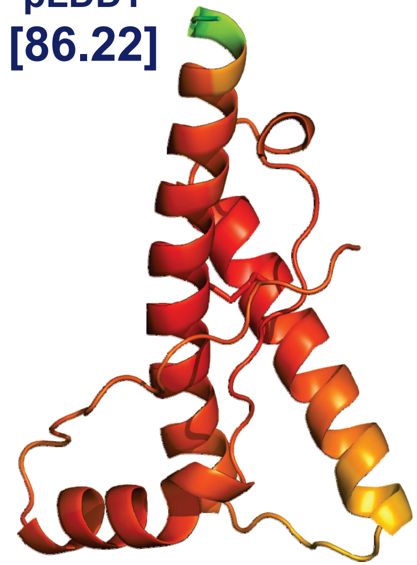

Protein code: 0824

Damaraland mole-rat

GenBank: XM_010607544

Cryptomys damarensis

Rodentia 81/101
Bathyergidae 2/3

Distinct primary sequence across species

P49%

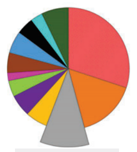

13

β2-α2 LOOP
SSQNNF
42

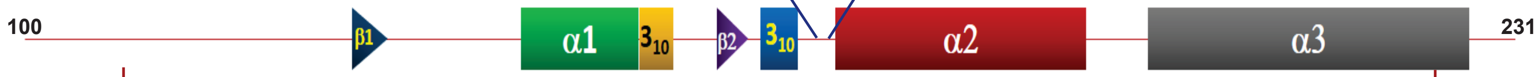


```
WNKPNKPKTNMKHMAGAAAAGAVVGGLGGYMLGSAMSRPLIHFSSDYEDRYYRENMYRYPNQVYYRPVDQYSSQNNFVQDCVNITIKQHTVTTTTKGENFTETDVKIMERVLEQMCTTQYQKESQAHYYRGRAG-
      S     I  V                            M    GN W          N                NN     H                                 M    V    V           Y EGRSS
```

PrP sequence differs by 13 amino acids from the bank vole PrP (see in red)

No deletions

No insertions

No polymorphic variants found

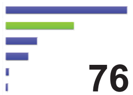

76

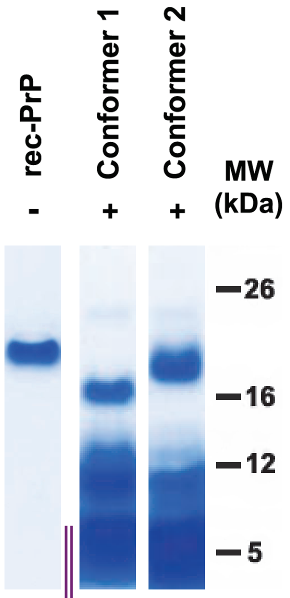

194 AA

2

Conformers

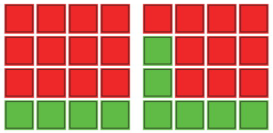

Tm^{Exp}: ND

ΔΔG: ND

In vitro studies

TgVole (1x) — Not tested

Damaraland mole-rat — Not tested

In vivo studies

TgVole (1x) — Not tested

Damaraland mole-rat — Not tested

pLDDT [87.29]

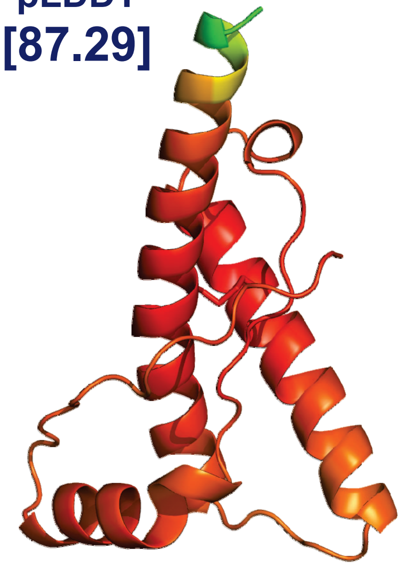

Protein code: 1130

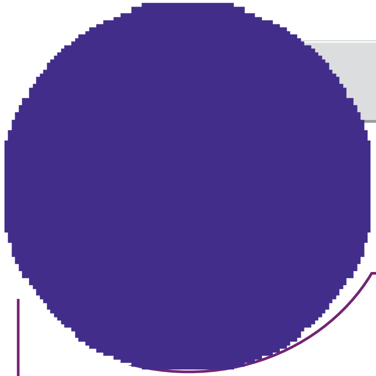

Mashona mole-rat

GenBank: BK064181

P46%

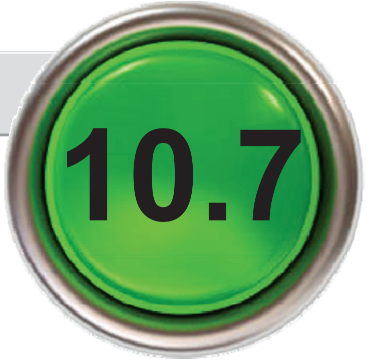

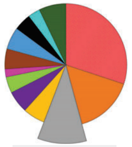

Cryptomys darlingi

Rodentia	81/101
Bathyergidae	2/3

β2-α2 LOOP
SSQNNFF
42

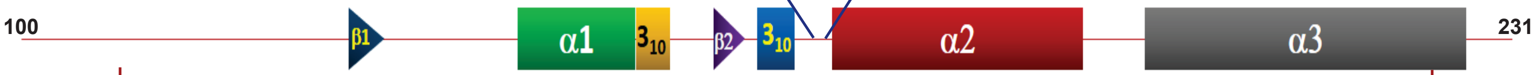

WNKPNKPKTNMKHMAGAAAAGAGVGGLGGYMLGSAMSRPLIHFSSDYEDRYYRENMYRYPNQVYYRPVDQYSSQNNFVQDCVNITIKQHTVTTTTKGENFTETDVKIMERVLEQMCTTQYQKESQAHYYRGRAG-
S I V V M GN W N NN H M V V Y EGRSS

PrP sequence differs by 14 amino acids from the bank vole PrP (see in red)

No deletions
No insertions
No polymorphic variants found

Distinct primary sequence across species

33

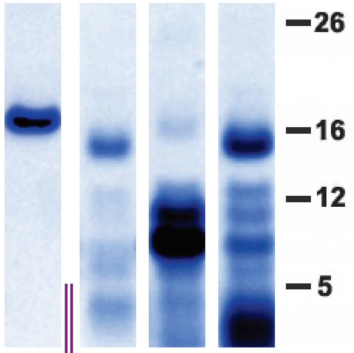

178 AA

3 Conformers

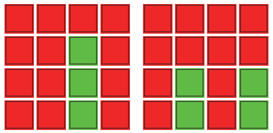

Tm[Exp]: ND

ΔΔG: ND

In vitro studies

TgVole (1x) Not tested

Mashona mole-rat Not tested

In vivo studies

TgVole (1x) Not tested

Mashona mole-rat Not tested

pLDDT
[87.08]

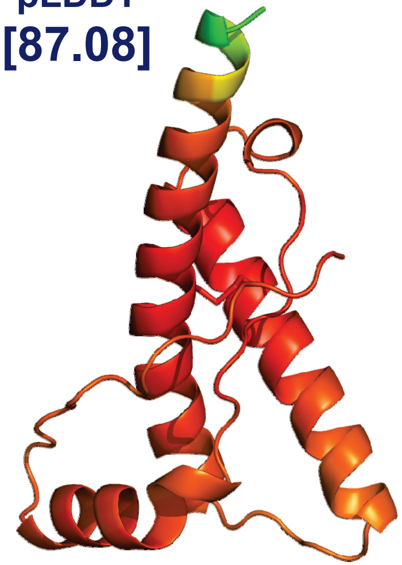

Protein code: 4004

Common gundi

GenBank: BK063949

Ctenodactylus gundi

Rodentia 81/101
Ctenodactylidae 1/1

P79%

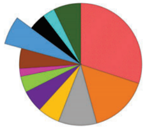

71.1

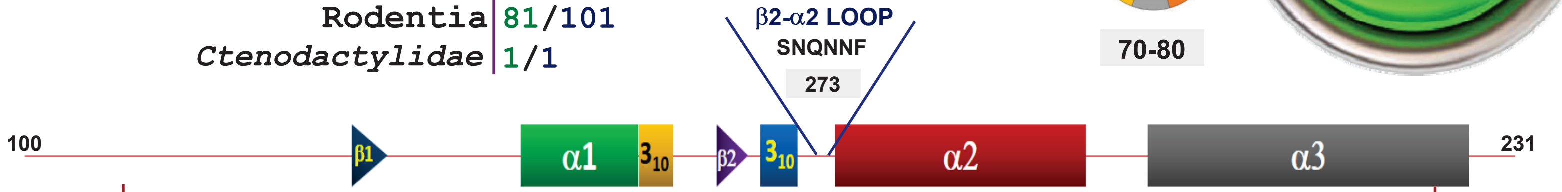

WNKPSKPKTSMKHVAGAAAAGAVVGGLGGYMLGSAMSRPLIHFGNDYEDRYYRENMNRYPNQVYYRPIDQYSNQNNFVHDCVNITIKQHTVTTTTKGDNFTETDIKIMERVVEQMCITQYQQEAQAYQYQGRS-
NI M W V N E V M V K S YEGRS

PrP sequence differs by 12 amino acids from the bank vole PrP (see in red)

No deletions
No insertions
No polymorphic variants found

Distinct primary sequence across species

76

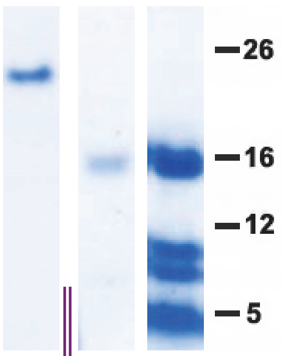

210 AA

2
Conformers

Tm^{Exp}: ND

ΔΔG: -6.5

In vitro studies

TgVole (1x) Not tested

Common gundi Not tested

In vivo studies

TgVole (1x) Not tested

Common gundi Not tested

pLDDT [86.49]

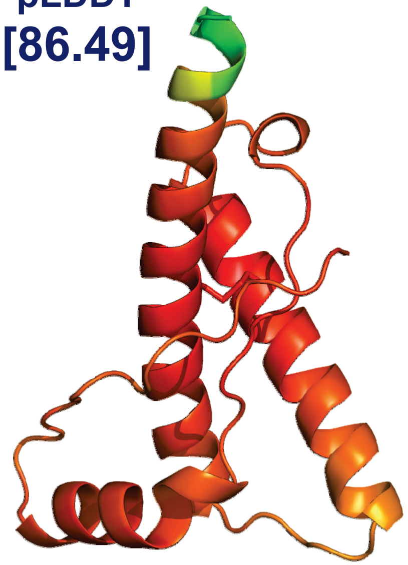

Social tuco-tuco

GenBank: BK063951

Ctenomys sociabilis

Rodentia 81/101

Ctenomyidae 1/1

P60% 28.6

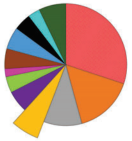

β2-α2 LOOP
ANENNF
1

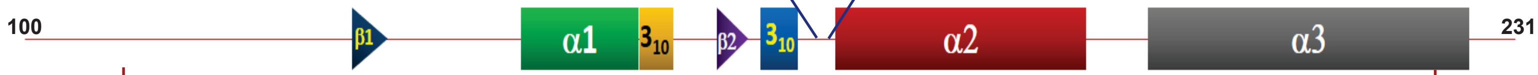

WNKPSKPKTNMKHVAGAAAAGAVVGGLGGYMLGSAMSRPLIHFGNDYEDRYYRDNMYRYPNQVYYKPVDQYANENNFVQDCVNITIKQHTTTTTTKGENFTETDVKIMERVLQQMCTTQYQKESQAYYYQPRGG-

I M W E N R N Q H V M VE V EGRSS

PrP sequence differs by 14 amino acids from the bank vole PrP (see in red)

No deletions

No insertions

No polymorphic variants found

Distinct primary sequence across species

76

203 AA

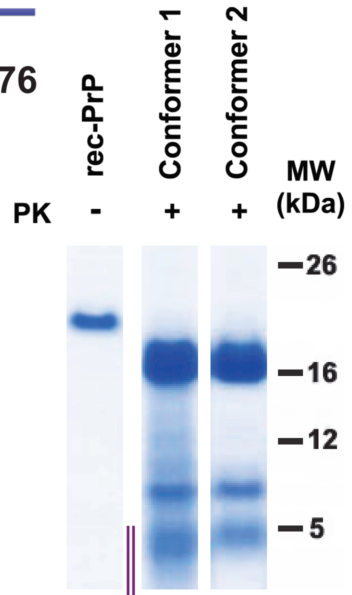

2 Conformers

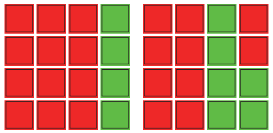

Tm^Exp: ND

ΔΔG: ND

In vitro studies

TgVole (1x) Not tested

Social tuco-tuco Not tested

In vivo studies

TgVole (1x) Not tested

Social tuco-tuco Not tested

pLDDT [83.95]

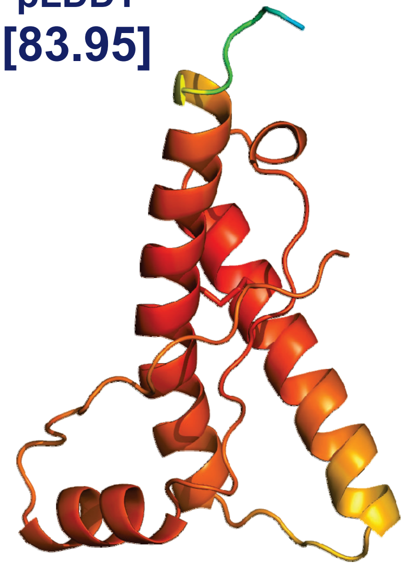

Protein code: 3078

Central American agouti

GenBank: BK063998

Dasyprocta punctata

Rodentia 81/101

Dasyproctidae 1/1

Distinct primary sequence across species

P45%

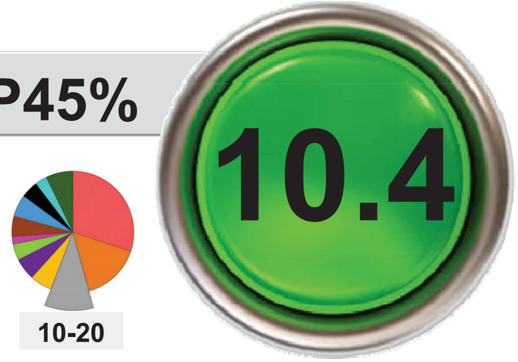

β2-α2 LOOP
SNQNNF
273

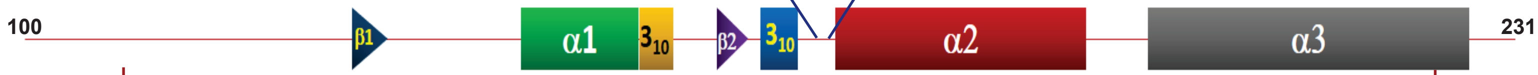

WNKPSKPKTNMKHMAGAAAAGAVVGGLGGYMLGSAMSRPLIHFGNDFEDRYYRENMYRYPNQVYYKPVDQYSNQNNFVQDCVNITIKQHTVTTTTKGENFTETDVKIMERVLEQMCTTQYQKESQAYYSPGRAG-

I V M W N R N H M V V EGRSS

PrP sequence differs by 11 amino acids from the bank vole PrP (see in red)

No deletions

No insertions

No polymorphic variants found

25

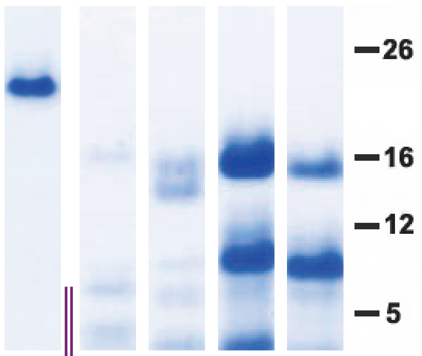

202 AA

4

Conformers

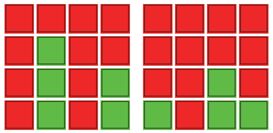

Tm^{Exp}: ND

ΔΔG: ND

In vitro studies

TgVole (1x) Not tested

Central American agouti Not tested

In vivo studies

TgVole (1x) Not tested

Central American agouti Not tested

pLDDT [83.85]

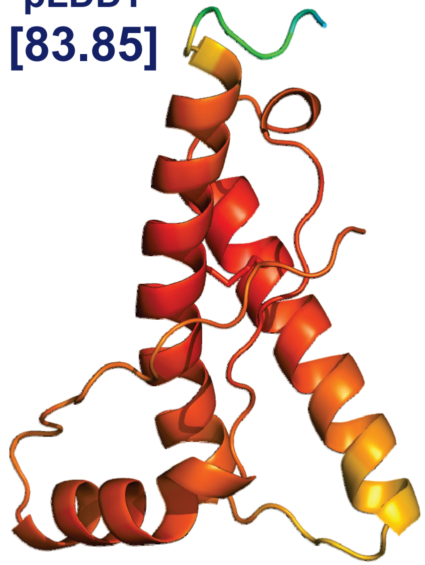

Protein code: 3158

Pacarana

GenBank: BK063954

P59%

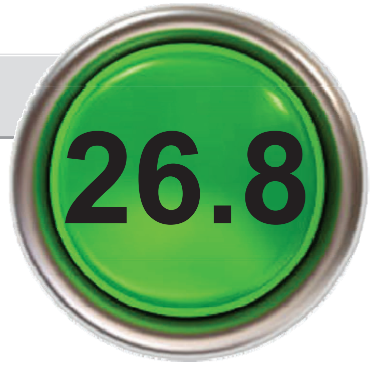

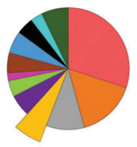

20-30

Dinomys branickii

Rodentia 81/101
Dinomyidae 1/1

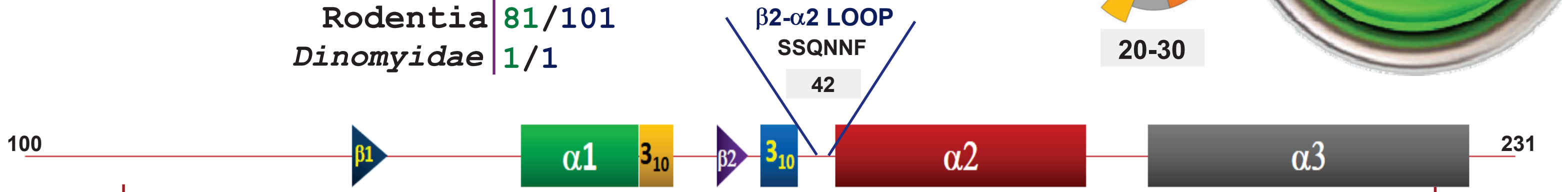


```
WNKPSKPKTNMKHVAGAAAAGAVVGGLGGYMLGSAMSRPLIHFGSDYEDRYYRENMYRYPNQVYYKPVDQYSSQNNFVQDCVNITIKQHTVTTTTKGENFTETDVKIMERVLEQMCATQYQKESQAYYYHGRAG-
           I                      M    N W       N       R     NN    H                         M   V   V               EGRSS
```

PrP sequence differs by 12 amino acids from the bank vole PrP (see in red)

No deletions
No insertions
No polymorphic variants found

Distinct primary sequence across species

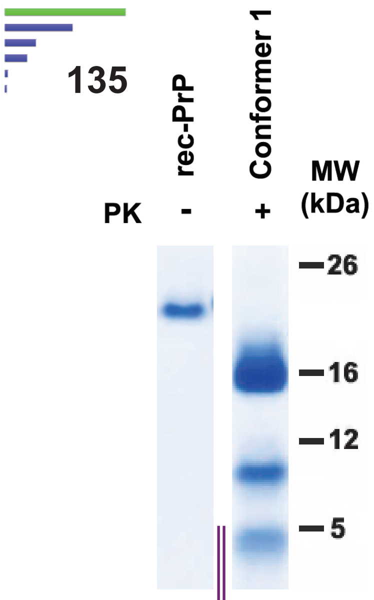

204 AA

1
Conformers

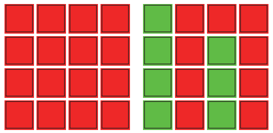

Tm^{Exp}: ND

ΔΔG: ND

In vitro studies

TgVole (1x) Not tested

Pacarana Not tested

In vivo studies

TgVole (1x) Not tested

Pacarana Not tested

pLDDT
[84.46]

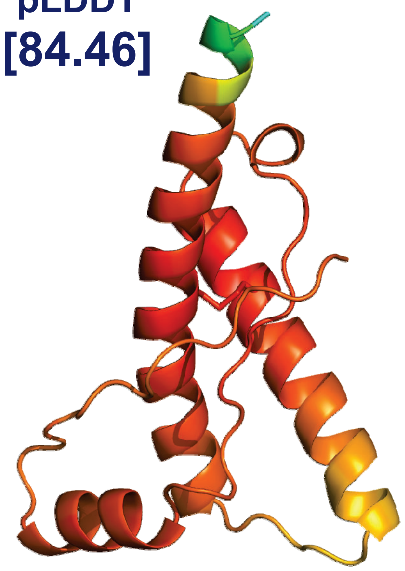

Protein code: 3082

Merriam's kangaroo rat

GenBank: BK064205

Dipodomys merriami

Rodentia	81/101
Heteromyidae	5/5

P91%

92.9

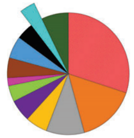

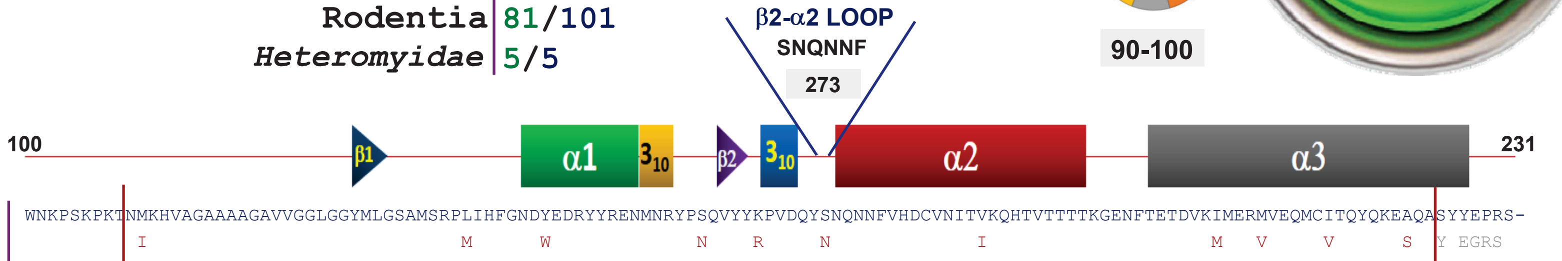

PrP sequence differs by 11 amino acids from the bank vole PrP (see in red)

No deletions

No insertions

No polymorphic variants found

Distinct primary sequence across species

135

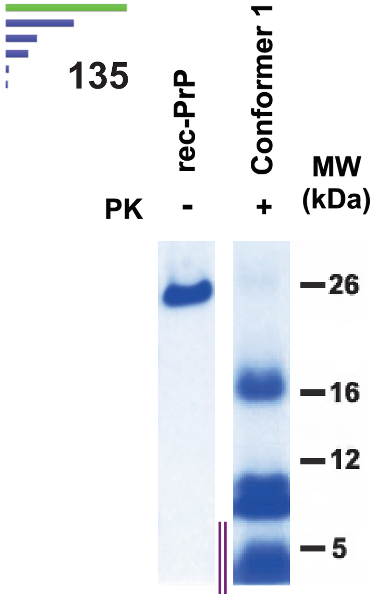

211 AA

1 Conformers

Tm^{Exp}: ND

ΔΔG: 1.98

In vitro studies

TgVole (1x) Not tested

Merriam's kangaroo rat Not tested

In vivo studies

TgVole (1x) Not tested

Merriam's kangaroo rat Not tested

pLDDT [84.87]

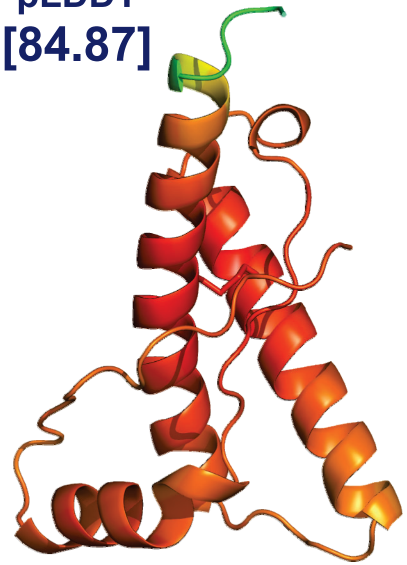

Protein code: 4560

Ord's kangaroo rat

GenBank: XM_013010955

Dipodomys ordii

Rodentia	81/101
Heteromyidae	5/5

P85%

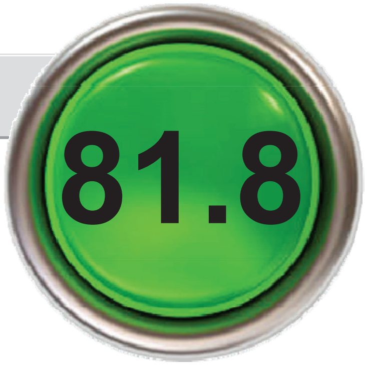

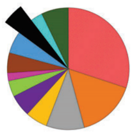

80-90

β2-α2 LOOP
NNQNNF
164

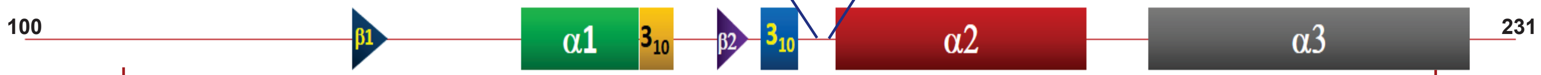

WNKPSKPKTNMKHVAGAAAAGAVVGGLGGYMLGSAMSRPLIHFGNDYEDRYYRENMNRYPSQVYYKPVDQYNNQNNFVHDCVNITIKQHTVTTTTKGENFTETDVKIMERMVEQMCITQYQKEAQASYYEPRS-

I M W N R M V V S Y EGRS

PrP sequence differs by 9 amino acids from the bank vole PrP (see in red)

No deletions
No insertions
No polymorphic variants found

Distinct primary sequence across species

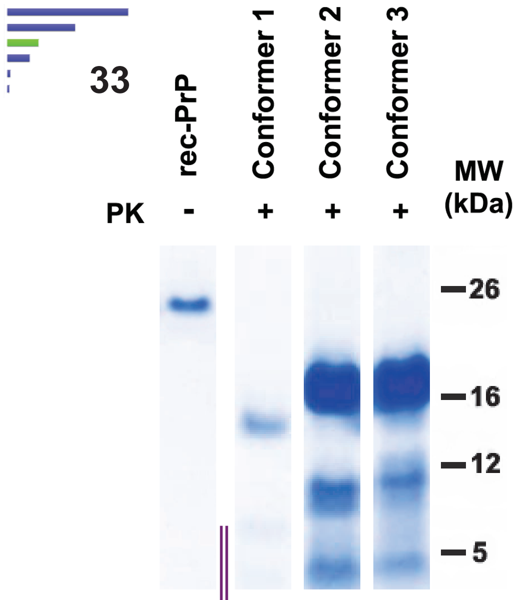

219 AA

3
Conformers

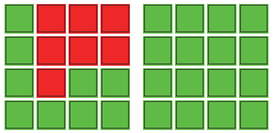

Tm^{Exp}: ND

ΔΔG: 2.83

In vitro studies

TgVole (1x) Not tested

Ord's kangaroo rat Not tested

In vivo studies

TgVole (1x) Not tested

Ord's kangaroo rat Not tested

pLDDT
[86.28]

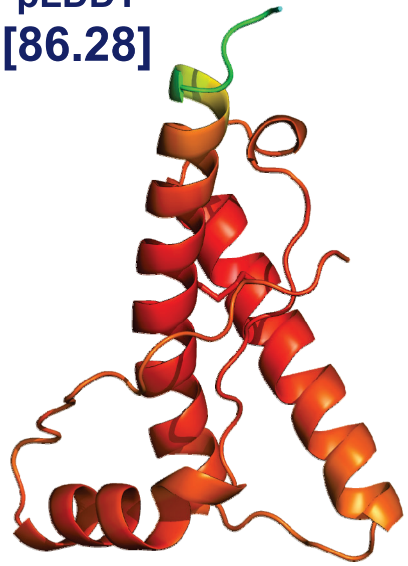

Protein code: 2568

Banner-tailed kangaroo rat

GenBank: BK064155

Dipodomys spectabilis

Rodentia	81/101
Heteromyidae	5/5

P67%

39.8

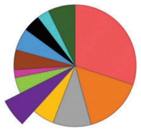

30-40

β2-α2 LOOP
SNQNNF
273

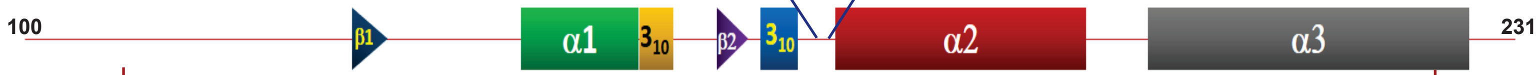

WNKPSKPKTNMKHMAGAAAAGAVVGGLGGYMLGSAMSRPLIHFGNDYEDRYYRENMNRYPSQVYYKPVDQYSNQNNFVHDCVNITVKQHTVTTTTKGENFTETDVKIMERMVEQMCITQYQKEAQASYYEPRS-

I V M W N R N I M V V S Y EGRS

PrP sequence differs by 12 amino acids from the bank vole PrP (see in red)

No deletions

No insertions

No polymorphic variants found

Distinct primary sequence across species

25

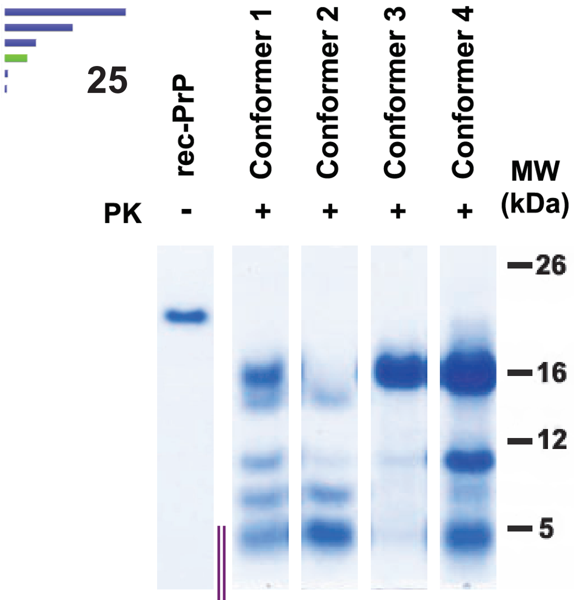

219 AA

4

Conformers

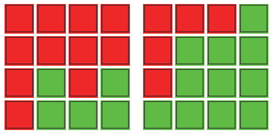

Tm[Exp]: ND

ΔΔG: -8.72

In vitro studies

TgVole (1x)	Not tested
Banner-tailed kangaroo rat	Not tested

In vivo studies

TgVole (1x)	Not tested
Banner-tailed kangaroo rat	Not tested

pLDDT [86.11]

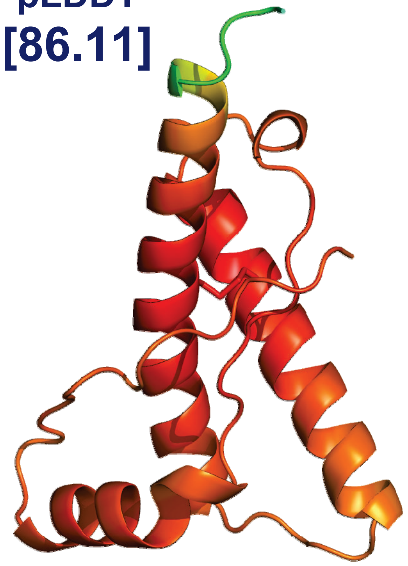

Protein code: 3688

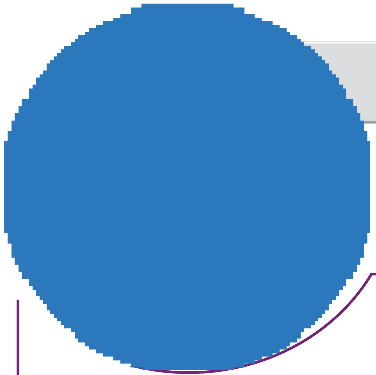

Stephens' kangaroo rat

GenBank: BK064064

Dipodomys stephensi

Rodentia 81/101

Heteromyidae 5/5

P100%

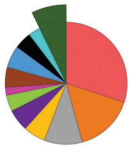

100

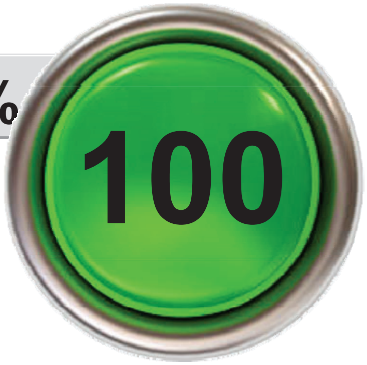

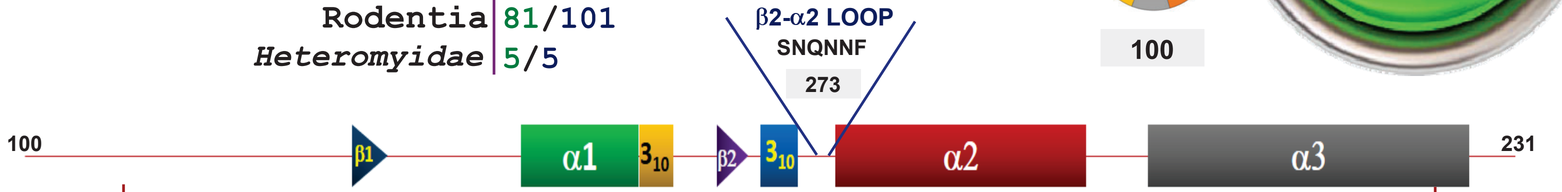

WNKPSKPKTNMKHVAGAAAAGAVVGGLGGYMLGSAMSRPLIHFGNDYEDRYYRENMNRYPSQVYYKPVDQYSNQNNFVHDCVNITVKQHTVTTTTKGENFTETDVKIMERMVEQMCITQYQKEAQAYYEPRS-

I M W N R N I M V V S G S

PrP sequence differs by 11 amino acids from the bank vole PrP (see in red)

No deletions

No insertions

No polymorphic variants found

Distinct primary sequence across species

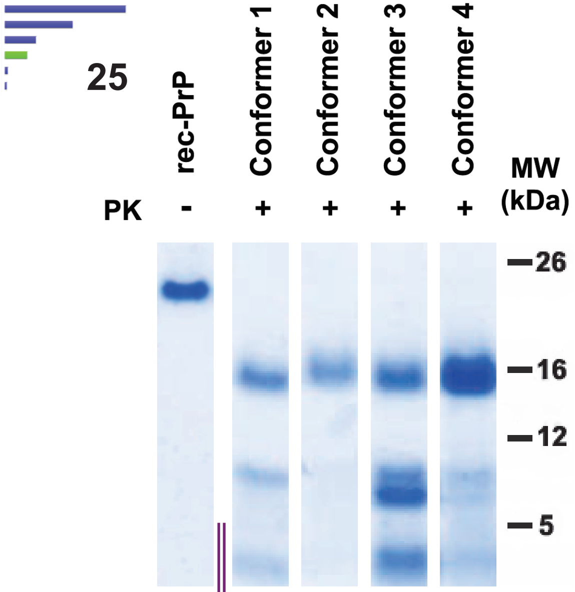

218 AA

4

Conformers

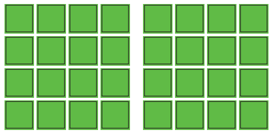

Tm[Exp]: ND

ΔΔG: ND

In vitro studies

TgVole (1x) Not tested

Stephens' kangaroo rat Not tested

In vivo studies

TgVole (1x) Not tested

Stephens' kangaroo rat Not tested

pLDDT [86.16]

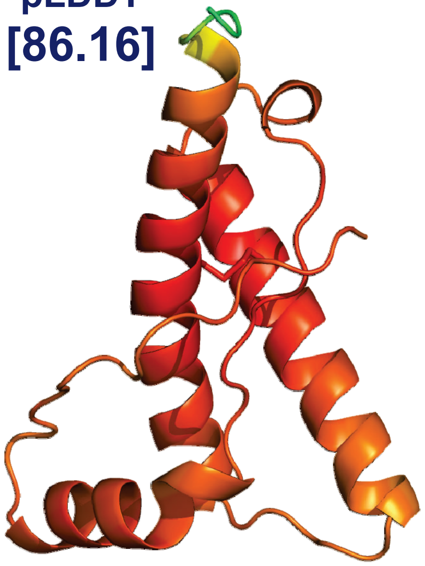

Protein code: 3240

Distinct primary sequence across species

Patagonian mara

GenBank: KM357833

Dolichotis patagonum

Rodentia	81/101
Caviidae	1/5

P29%

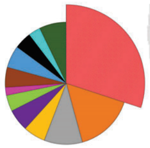

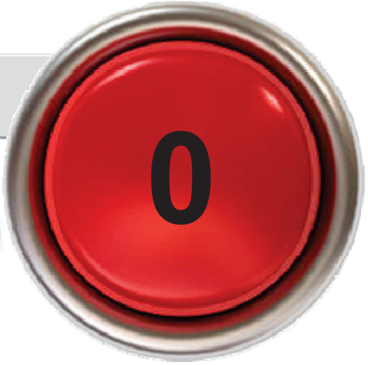

0

β2-α2 LOOP
NSQNSF
5

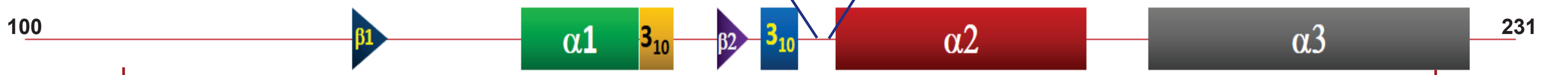

WNKPSKPKTNMKHMAGAAAAGAVVGGLGGYMLGSAMSRPLIHFGSDYEDRYYRENMYRYPNQVYYKPVDQYNSQNSFVQDCVNITIKQHTVTTTTKGENFTETDVKIMERVLEQMCTTQYQKESQAYYYHGRAG-

I V M N W N R N N H M V V EGRSS

PrP sequence differs by 13 amino acids from the bank vole PrP (see in red)

No deletions

No insertions

No polymorphic variants found

Tm^{Exp}: ND

ΔΔG: ND

PK
rec-PrP -
MW (kDa)

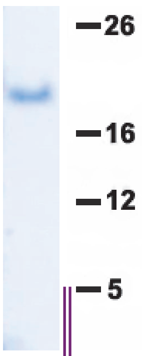

202 AA

NO MISFOLDING

0 Conformers

In vitro studies

NOT APPLICABLE

In vivo studies

NOT APPLICABLE

pLDDT
[84.68]

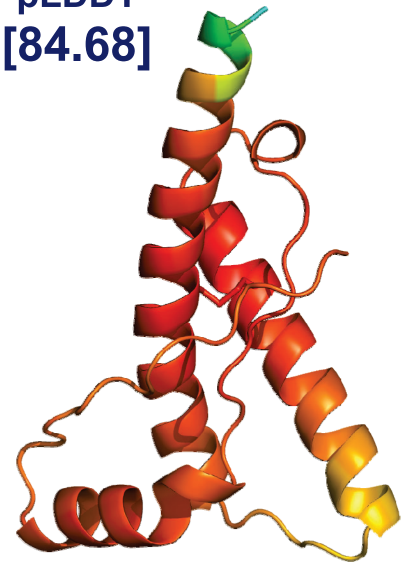

Protein code: 1087

Transcaucasian mole vole

GenBank: BK064014

Ellobius lutescens

Rodentia 81/101
Cricetidae 25/27

P100%

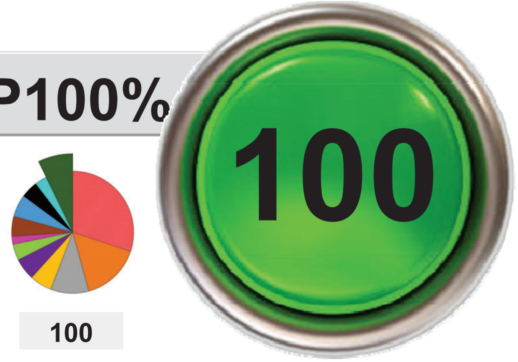

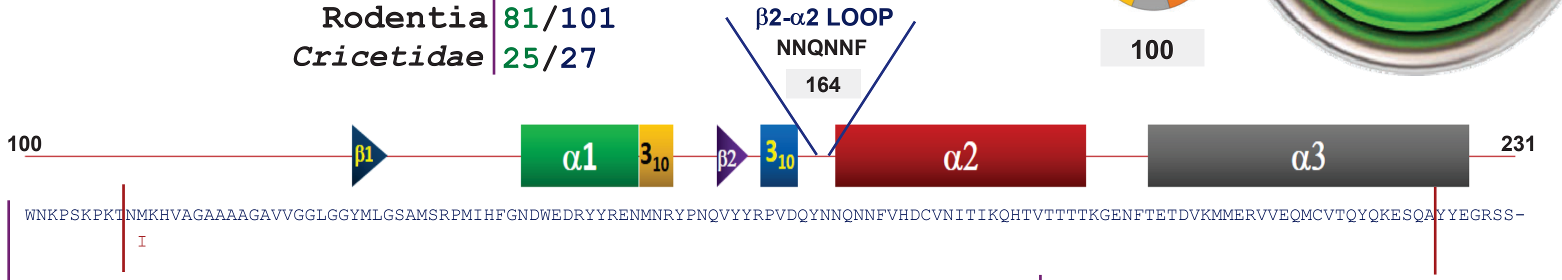

PrP sequence differs by 1 amino acids from the bank vole PrP (see in red)

No deletions
No insertions
No polymorphic variants found

Distinct primary sequence across species

135

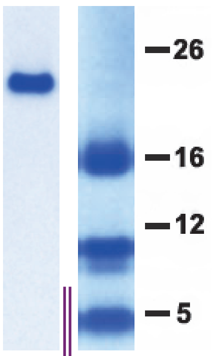

202 AA

1 Conformers

Tm^{Exp}: ND

ΔΔG: 6.65

In vitro studies

TgVole (1x) — Not tested

Transcaucasian mole vole — Not tested

In vivo studies

TgVole (1x) — Not tested

Transcaucasian mole vole — Not tested

pLDDT [85.48]

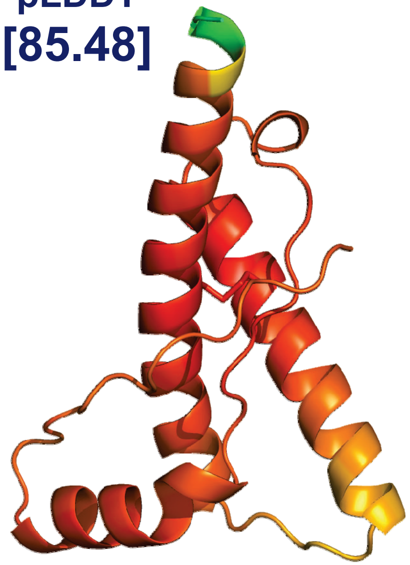

Northern mole vole

GenBank: BK064040

P100%

100

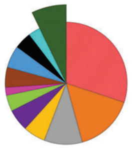

Ellobius talpinus

Rodentia 81/101
Cricetidae 25/27

β2-α2 LOOP
NNQNNF
164

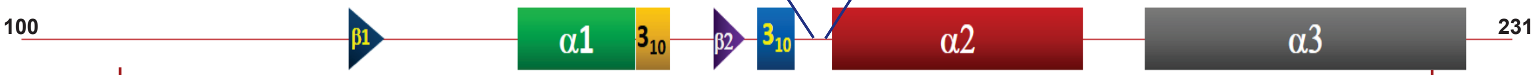

WNKPSKPKTNMKHVAGAAAAGAVVGGLGGYMLGSAMSRPMIHFGNDWEDRYYRENMNRYPNQVYYRPVDQYNNQNNFVHDCVNITIKQHTVTTTTKGENFTETDVKMMERVVEQMCVTQYQKESQAYYEGRSS-
I

PrP sequence differs by 1 amino acids from the bank vole PrP (see in red)

No deletions
No insertions
No polymorphic variants found

Distinct primary sequence across species

135

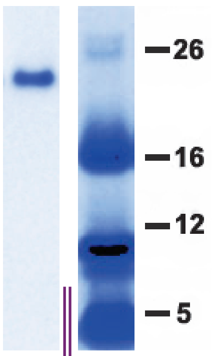

210 AA

1
Conformers

Tm^{Exp}: ND
ΔΔG: 3.73

In vitro studies

TgVole (1x) Not tested
Northern mole vole Not tested

In vivo studies

TgVole (1x) Not tested
Northern mole vole Not tested

pLDDT
[85.45]

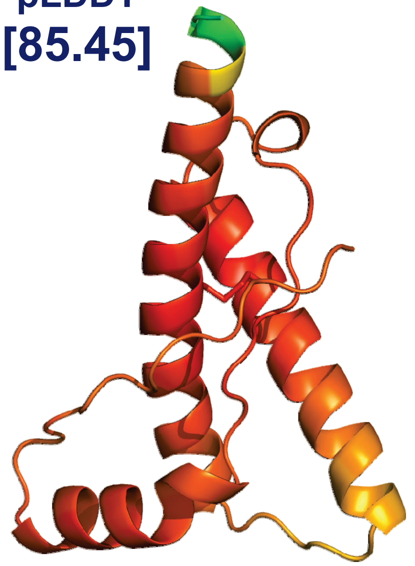

Protein code: 3206

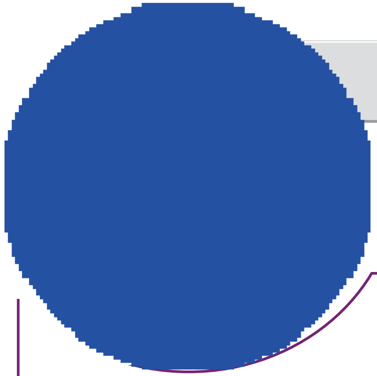

Chinese zokor

GenBank: BK064949

P78%

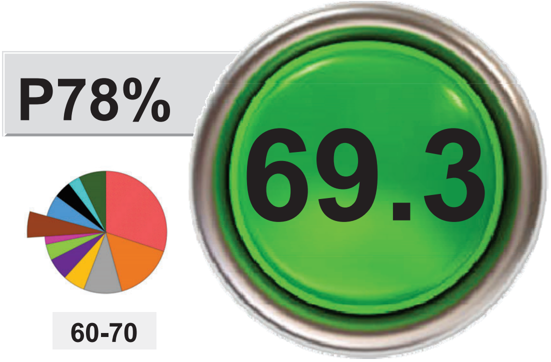

Eospalax fontanierii

Rodentia	81/101
Spalacidae	3/3

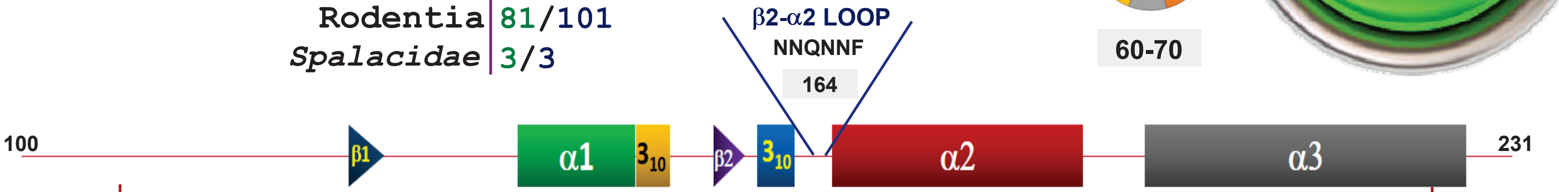

WNKPSKPKTNMKHVAGAAAAGAVVGGLGGYMLGSAMSRPLIHFGNDYEDRYYRENMYRYPNQVYYRPVDQYNNQNNFVHDCVNITVKQHTVTTTTKGENFTETDIKIMERVVEQMCITQYQKESQAYYAGRSS-

I M W N I V M V E

PrP sequence differs by 8 amino acids from the bank vole PrP (see in red)

No deletions
No insertions
No polymorphic variants found

Distinct primary sequence across species

25

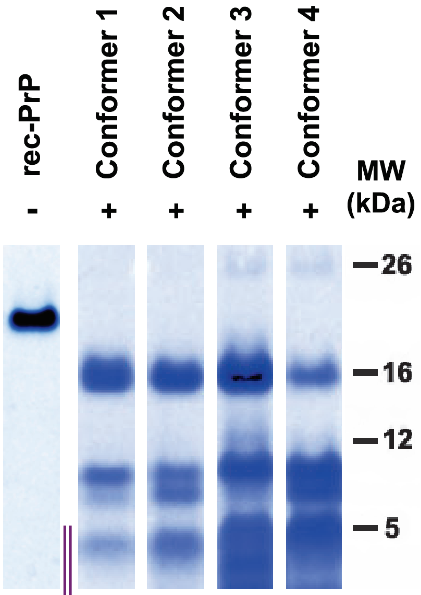

203 AA

4 Conformers

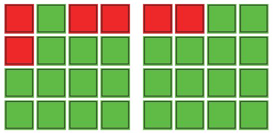

Tm^{Exp}: ND

ΔΔG: -3.33

***In vitro* studies**

TgVole (1x) — Not tested

Chinese zokor — Not tested

***In vivo* studies**

TgVole (1x) — Not tested

Chinese zokor — Not tested

pLDDT [85.59]

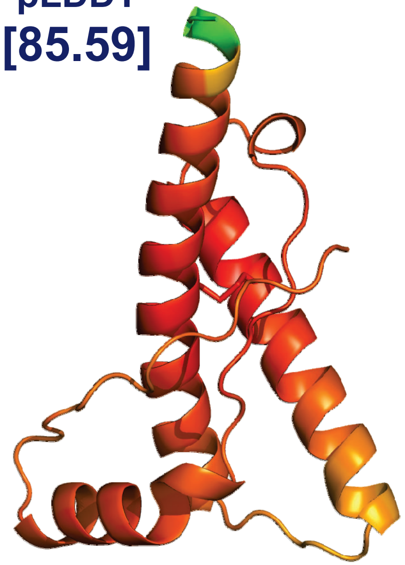

Protein code: 4002

North American porcupine

GenBank: BK063957

P29%

0

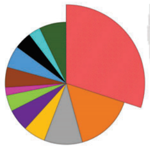

0

Erethizon dorsata

Rodentia 81/101

Erethizontidae 0/2

β2-α2 LOOP
SGQKSF
2

100 β1 α1 3_{10} β2 3_{10} α2 α3 231

```
WNKPSKPKTNLKHMAGAAAAGAVVGGLGGYMLGSAMSRPLIHFGSDYEDRYYRENMYRYPDQVYYRPVGQYSGQKSFVQDCVNITIKQHTVTATTKGENFTETDVKIMERVLEQMCTTQYQKESQAYYYRGRAG-
           I  V                                 M    N W         N   N        D  NN NN  H            T                M   V   V         EGRSS
```

PrP sequence differs by 17 amino acids from the bank vole PrP (see in red)

No deletions

No insertions

No polymorphic variants found

Distinct primary sequence across species

Tm^{Exp}: ND

ΔΔG: ND

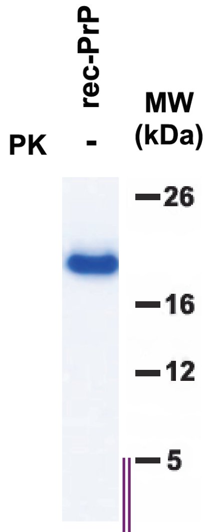

202 AA

NO MISFOLDING

0
Conformers

In vitro studies

NOT APPLICABLE

In vivo studies

NOT APPLICABLE

pLDDT
[85.98]

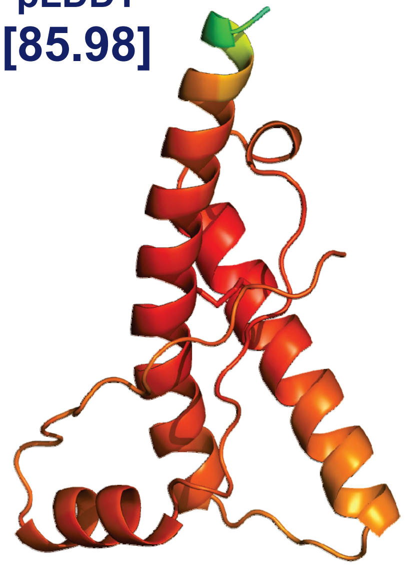

Protein code: 3085

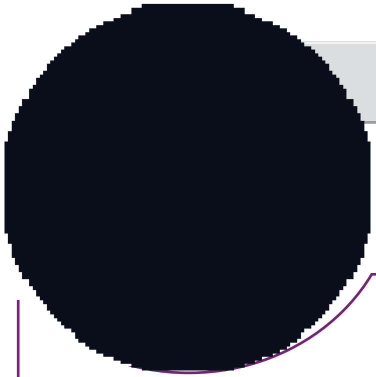

Southern flying squirrel

GenBank: BK064183

P85%

78.9

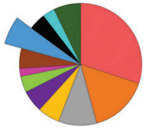

70-80

Glaucomys volans

Rodentia 81/101

Sciuridae 7/13

β2-α2 LOOP
NNQNSF
24

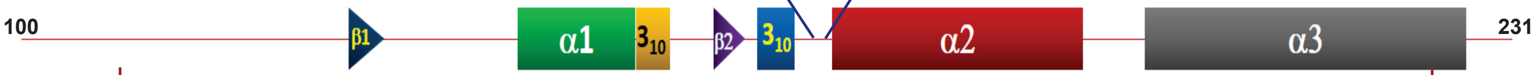

WGKPSKPKTNMKHVAGAAAAGAVVGGLGGYMLGSAMSRPLIHFGNDYEDRYYRENMNRYPNQVYYKPVDQYNNQNSFVHDCVNITVKQHTVTTTTKGENFTETDIKIMERVVEQMCITQYQQEAQAYYQRGAS-

N I M W R N I V M V K S EGRS

PrP sequence differs by 11 amino acids from the bank vole PrP (see in red)

No deletions

No insertions

No polymorphic variants found

Distinct primary sequence across species

33

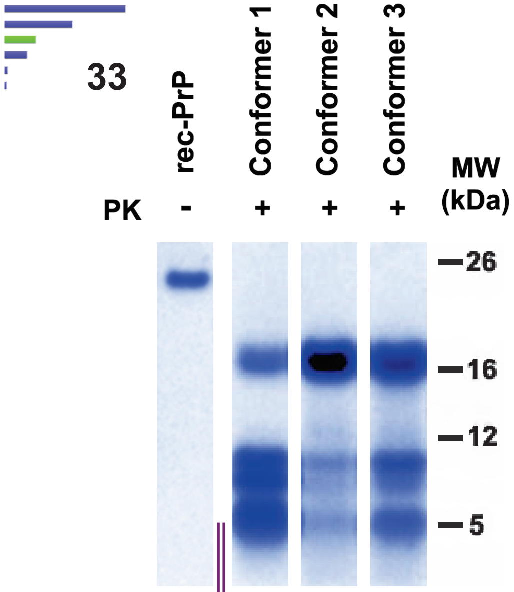

221 AA

3

Conformers

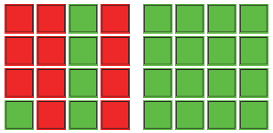

Tm^{Exp}: ND

ΔΔG: -8.59

In vitro studies

Not tested

TgVole (1x)

Not tested

Southern flying squirrel

In vivo studies

Not tested

TgVole (1x)

Not tested

Southern flying squirrel

pLDDT

[84.98]

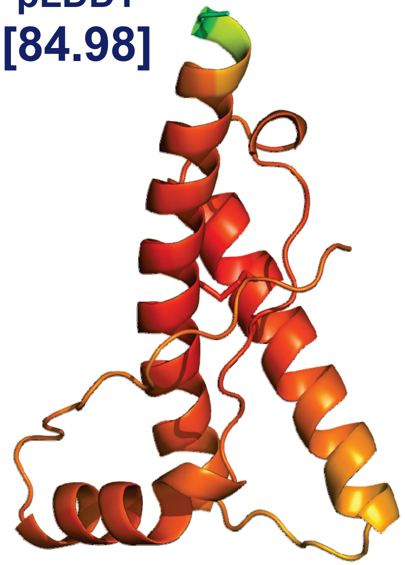

Protein code: 4022

Edible dormouse

GenBank: BK064048

P76%

64.3

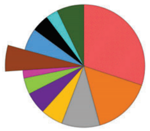

60-70

Glis glis

Rodentia 81/101
Gliridae 3/3

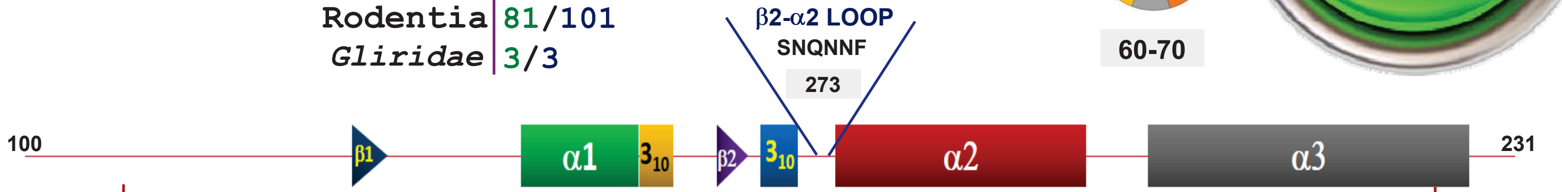

WNKPNKPKTNMKHMAGAAAAGAVVGGLGGYMVGSAMSRPAMHFGNDYEDRYYRENMNRYPNQVYYKPVDQYSNQNNFVHDCVNITVKQHTVTTTTKGENFTETDVKIMERVVEQMCITQYQKEAQAYYHGGSS-

S I V L MI W R N I M V S E R

PrP sequence differs by 12 amino acids from the bank vole PrP (see in red)

No deletions
No insertions
No polymorphic variants found

Distinct primary sequence across species

25

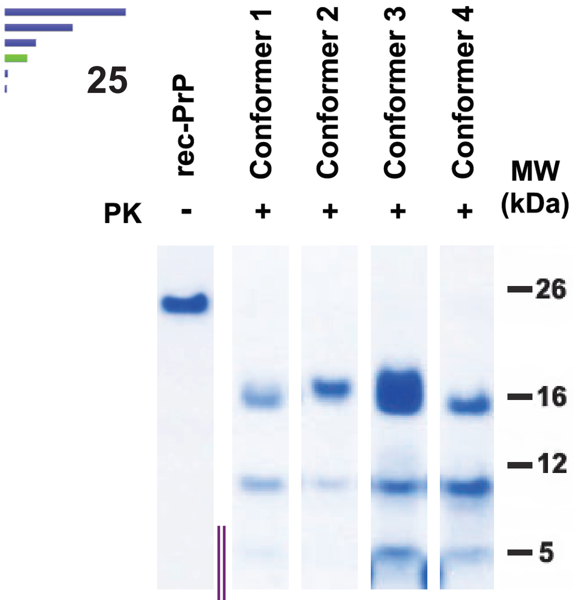

218 AA

4 Conformers

Tm^{Exp}: ND

ΔΔG: -2.93

In vitro studies

TgVole (1x) Not tested

Edible dormouse Not tested

In vivo studies

TgVole (1x) Not tested

Edible dormouse Not tested

pLDDT [84.73]

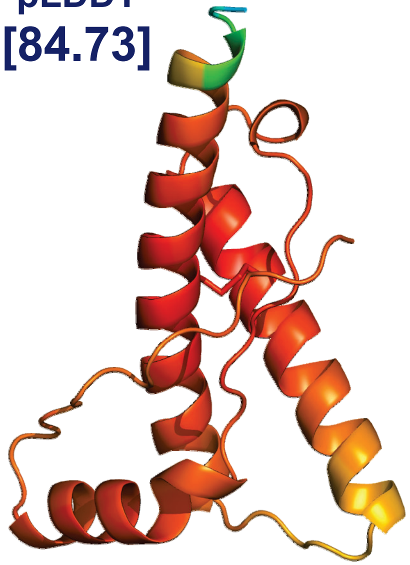

Protein code: 3217

African woodland thicket rat

GenBank: XM_028757107

Grammomys surdaster

Rodentia	81/101
Muridae	16/18

P86%

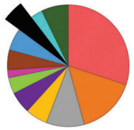

80-90

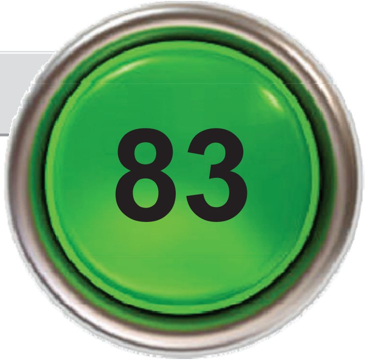

Distinct primary sequence across species

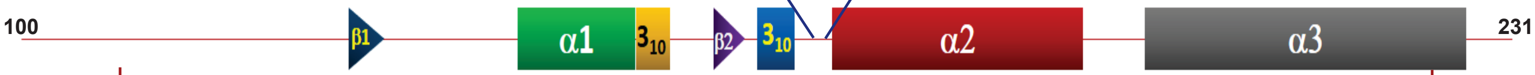

WNKPSKPKTNLKHVAGAAAAGAVVGGLGGYMLGSAMSRPMIHFGNDWEDRYYRENMYRYPNQVYYYRPVDQYNNQNSFVHDCVNITIKQHTVTTTTKGENFTETDVKMMERVVEQMCTTQYQKESQAYYDGRRS-

I N N V E S

PrP sequence differs by 4 amino acids from the bank vole PrP (see in red)

No deletions

No insertions

No polymorphic variants found

76

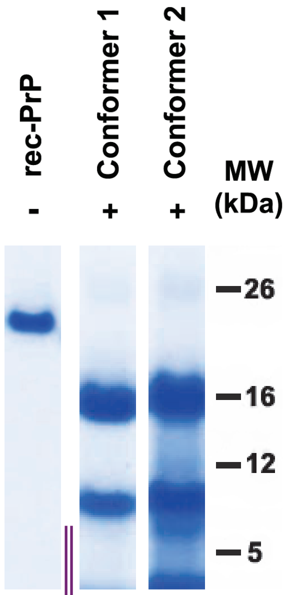

211 AA

2 Conformers

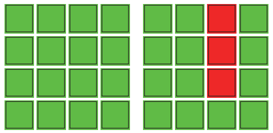

Tm[Exp]: ND

ΔΔG: -3.65

In vitro studies

TgVole (1x) Not tested

African woodland thicket rat Not tested

In vivo studies

TgVole (1x) Not tested

African woodland thicket rat Not tested

pLDDT [86.17]

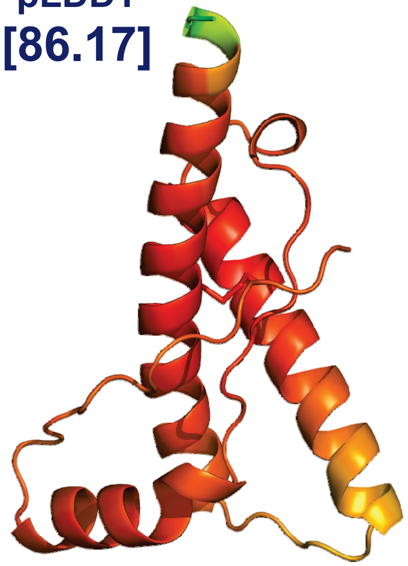

Protein code: 1001

Woodland dormouse

GenBank: BK064049

Graphiurus murinus

Rodentia 81/101
Gliridae 3/3

P47%

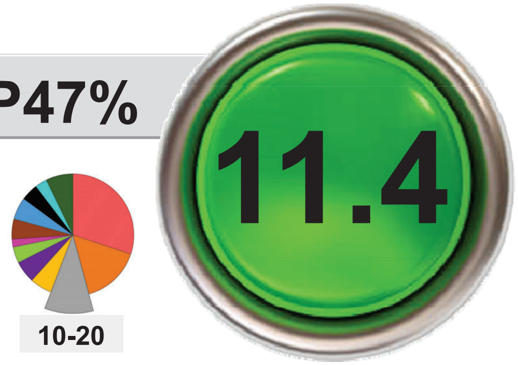

β2-α2 LOOP
NNQNNF
164

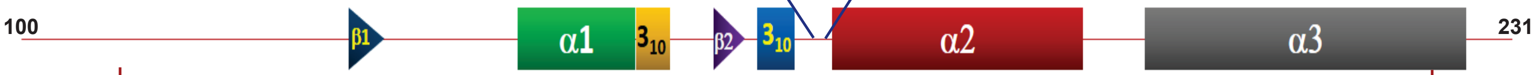

WNKPNKPKTNMKQMAGAAAAGAVVGGVGGFLVGSAMSRPPMHFGNEYEDRYYRENMNRYPNQVYYKPVDQYNNQNNFVRDCVNITVKQHTVTTTTKGENFTETDVKIMERVVEQMCITQYEKEAQAYYQRGAS-
S I HV L YML MI DW R H I M V Q S EGRS

PrP sequence differs by 18 amino acids from the bank vole PrP (see in red)

No deletions
No insertions
No polymorphic variants found

Distinct primary sequence across species

135

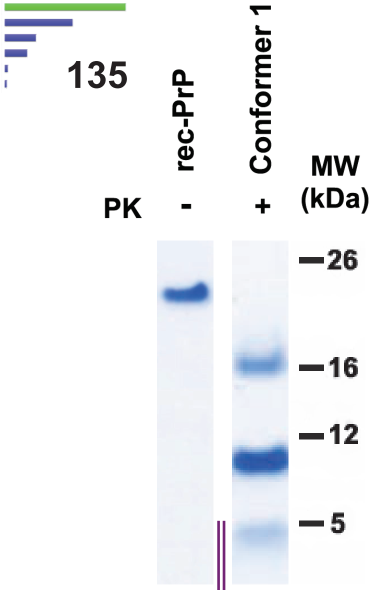

223 AA

1
Conformers

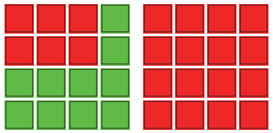

Tm^{Exp}: ND

ΔΔG: -7.47

In vitro studies

Not tested
TgVole (1x)

Not tested
Woodland dormouse

In vivo studies

Not tested
TgVole (1x)

Not tested
Woodland dormouse

pLDDT
[84.68]

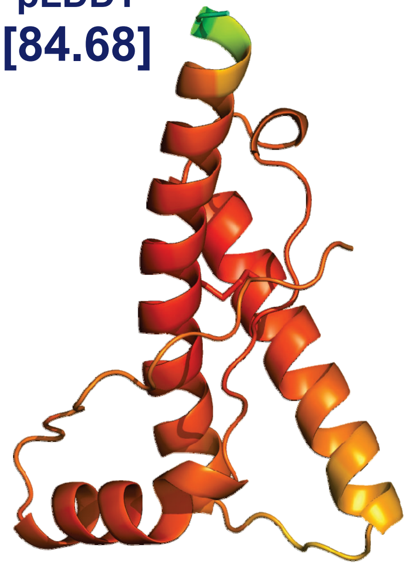

Protein code: 3218

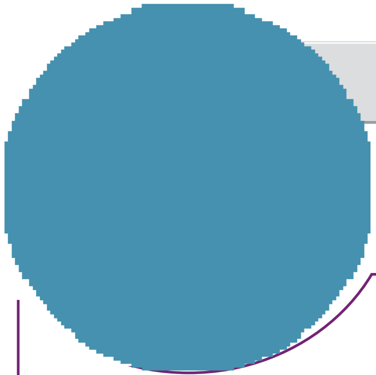

Naked mole-rat

GenBank: XM_004840771

Heterocephalus glaber

Rodentia 81/101

Bathyergidae 2/3

P29%

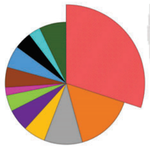

0

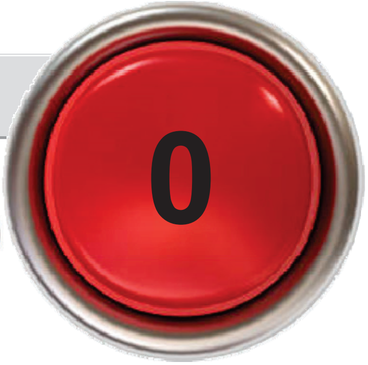

β2-α2 LOOP
NNQNSF
24

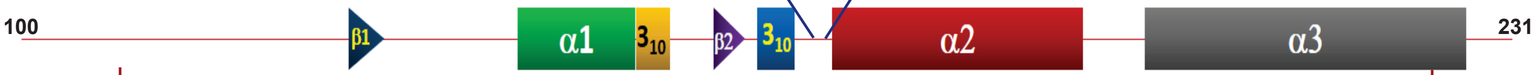

WNKPSKPKTNMKHMAGAAAAGAVVGGLTGYMLGSAMSRPLIHFGSDYEDRYYRENMYRYPNQVYYRPVDQYNNQNSFVQDCVNITIKQHTVTTTTKGENFTETDVKIMERVLEQMCTTQYQKESQAYYYRGRAG-

I V G M N W N N H M V V EGRSS

PrP sequence differs by 12 amino acids from the bank vole PrP (see in red)

No deletions

No insertions

No polymorphic variants found

Distinct primary sequence across species

rec-PrP

PK -

MW (kDa)

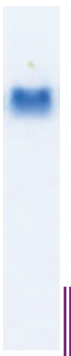

—26

—16

—12

—5

202 AA

NO MISFOLDING

0 Conformers

In vitro studies

NOT APPLICABLE

In vivo studies

NOT APPLICABLE

Tm^{Exp}: ND

ΔΔG: ND

pLDDT [85.44]

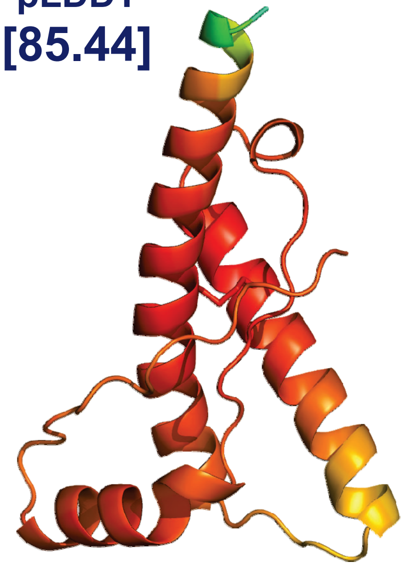

Protein code: 0978

Capybara

GenBank: KM357831

Hydrochoerus hydrochaeris

Rodentia 81/101
Caviidae 1/5

P29%

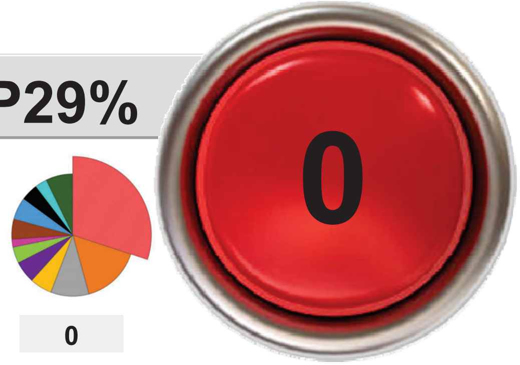

β2-α2 LOOP
SSQNSF
6

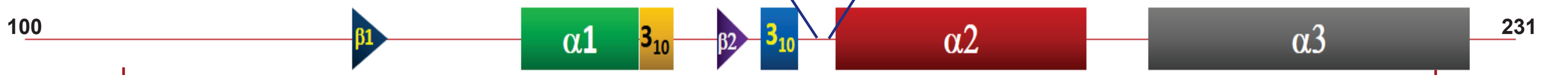

WNKPSKPKTNMKHMAGAAAAGAVVGGLGGYMLGSAMSRPLIHFGSDYEDRYYRENMYRYPNQVYYKPVDQYSSQNSFVQDCVNITIKQHTVTTTTKGENFTETDVKIMERVLEQMCTTQYQKESQAYYYHGRAG-

I V M N W N R NN N H M V V EGRSS

PrP sequence differs by 14 amino acids from the bank vole PrP (see in red)

No deletions
No insertions
No polymorphic variants found

1 species sharing the same primary sequence

Rock cavy
Kerodon rupestris

rec-PrP
PK -
MW (kDa)

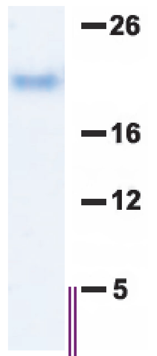

202 AA

NO MISFOLDING

0
Conformers

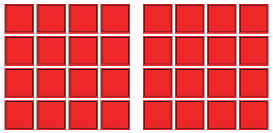

In vitro studies

NOT APPLICABLE

In vivo studies

NOT APPLICABLE

Tm^{Exp}: ND

ΔΔG: ND

pLDDT
[84.32]

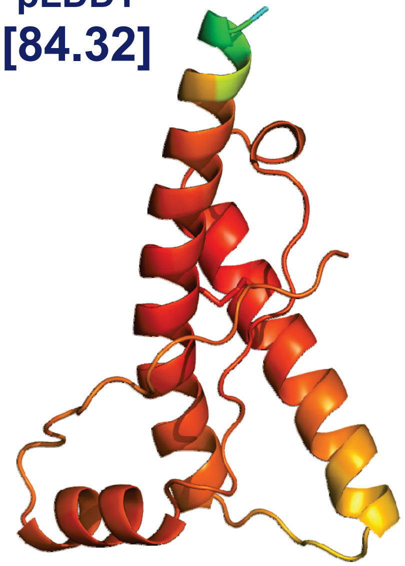

Protein code: 1435

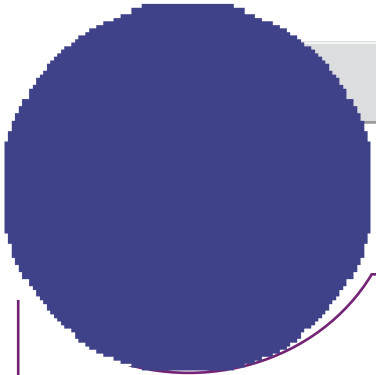

Allen's wood mouse

GenBank: BK064167

P67%

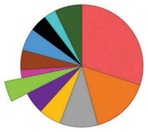

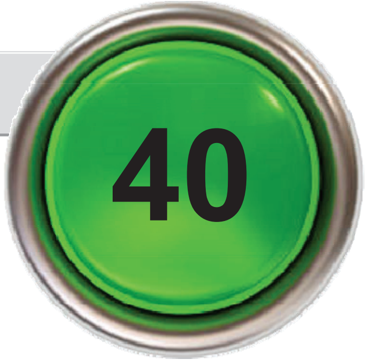

40-50

Hylomyscus alleni

Rodentia	81/101
Muridae	16/18

β2-α2 LOOP
SNQNNF
273

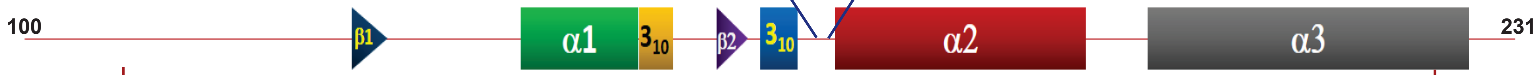

WNKPSKPKTNLKHVAGAAAAGAVVGGLGGYMLGSAMSRPMLHFGNDWEDRYYRENMYRYPNQVYYRPVDQYSNQNNFVHDCVNITIKQHTVTTTTKGDNFTETDVKMMERVVEQMCVTQYQKESQAYYEGRRS-

I I N N E S

PrP sequence differs by 5 amino acids from the bank vole PrP (see in red)

No deletions
No insertions
No polymorphic variants found

Distinct primary sequence across species

135

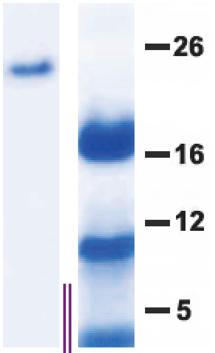

211 AA

1 Conformers

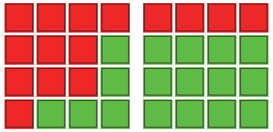

Tm^{Exp}: ND

ΔΔG: -10.63

In vitro studies

TgVole (1x) — Not tested

Allen's wood mouse — Not tested

In vivo studies

TgVole (1x) — Not tested

Allen's wood mouse — Not tested

pLDDT [85.87]

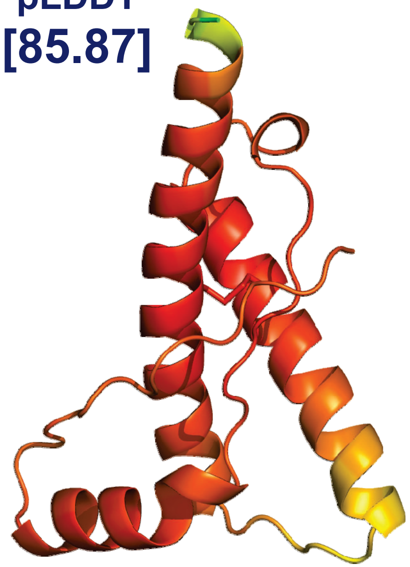

Protein code: 3803

Malayan porcupine

GenBank: BK063933

Hystrix brachyura

Rodentia	81/101
Hystricidae	0/1

P29%

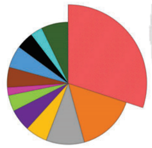

0

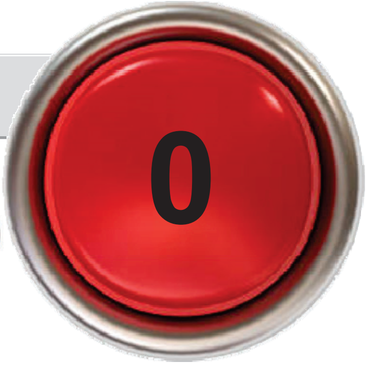

β2-α2 LOOP
SNQNSF
36

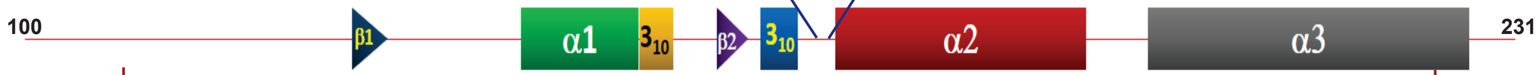


```
WNKPSKPKTNMKHMAGAAAAGAVVGGLGGYMLGSAMSRPLIHFGSDYEDRYYRENMYRYPNQVYYRPVDQYSNQNSFVQDCVNITIKQHTVTTTTKGENFTETDVKIMERVLEQMCTTQYQKESEAYYYRGRAG-
           I  V                      M   N W      N           N  N H                     M   V   V     Q  EGRSS
```

PrP sequence differs by 13 amino acids from the bank vole PrP (see in red)

No deletions
No insertions
No polymorphic variants found

1 species sharing the same primary sequence

Crested porcupine
Hystrix cristata

Tm^{Exp}: ND

ΔΔG: ND

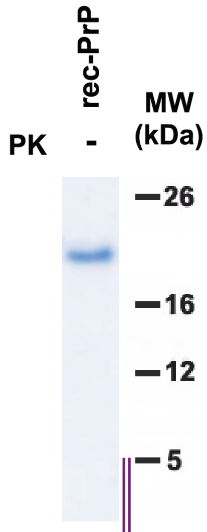

202 AA

NO MISFOLDING

0
Conformers

In vitro studies

NOT APPLICABLE

In vivo studies

NOT APPLICABLE

pLDDT
[87.14]

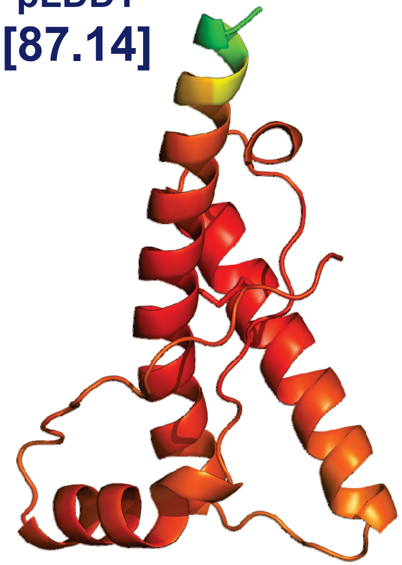

Protein code: 3056

Lesser Egyptian jerboa

GenBank: BK064939

P59%

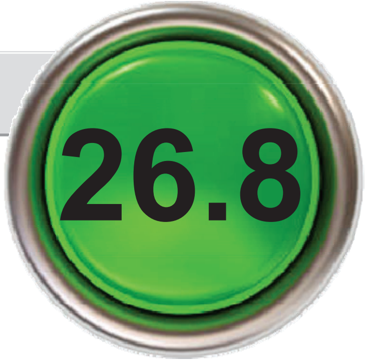

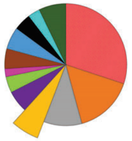

20-30

Jaculus jaculus

Rodentia 81/101

Dipodidae 3/3

β2-α2 LOOP
NNQNNF
164

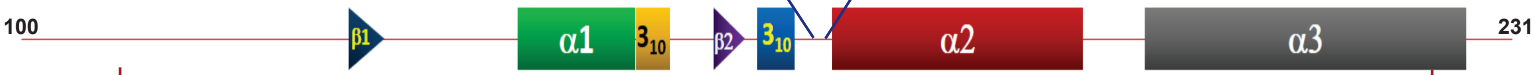

WNKPNKPKTSMKHMAGAAAAGAVAGGLGGYMLGSVMSRPSISFGNDYEDRYYRENMNRYPSQVYYKPVDQYNNQNNFVHDCVNITVKQHTVTTTTKGENFTETDVKIMERVVEQMCITQYQKESQAYLEGRRS-

S NI V V A M H W N R I M V Y S

PrP sequence differs by 13 amino acids from the bank vole PrP (see in red)

No deletions

No insertions

No polymorphic variants found

Distinct primary sequence across species

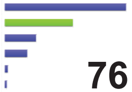

76

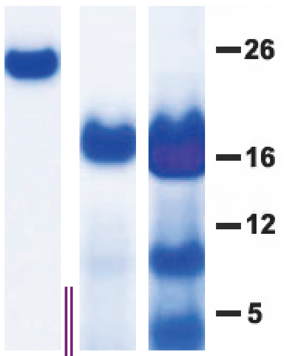

217 AA

2

Conformers

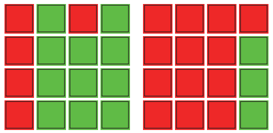

Tm^{Exp}: ND

ΔΔG: -14.88

In vitro studies

Not tested
TgVole (1x)

Not tested
Lesser Egyptian jerboa

In vivo studies

Not tested
TgVole (1x)

Not tested
Lesser Egyptian jerboa

pLDDT
[88.29]

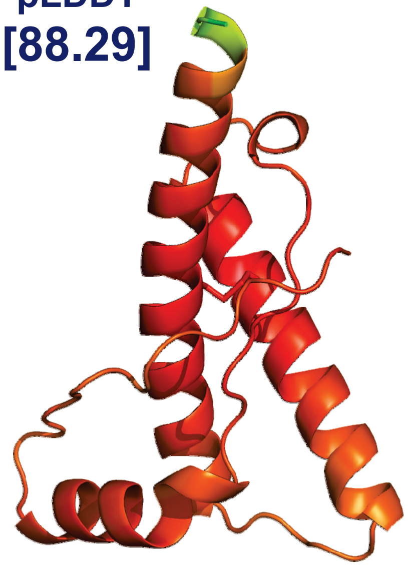

Protein code: 0936

Maned rat

GenBank: BK064153

Lophiomys imhausi

Rodentia 81/101
Cricetidae 25/27

P65%

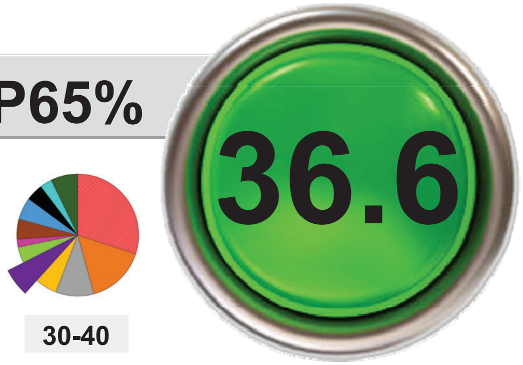

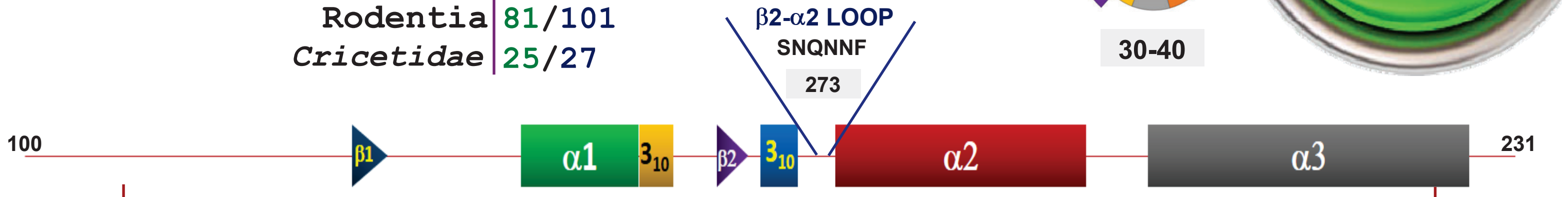

WNKPSKPKTNMKHVAGAAAAGAVVGGLGGYMLGSAMSRPMIHFGNDWEDRYYRENMYRYPNQVYYRPVDQYSNQNNFVHDCVNITIKQHTVTTTTKGENFTETDVKMMERVVEQMCVTQYQRESQAYYDGRRS-

I N N K E S

PrP sequence differs by 4 amino acids from the bank vole PrP (see in red)

No deletions
No insertions
No polymorphic variants found

Distinct primary sequence across species

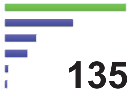

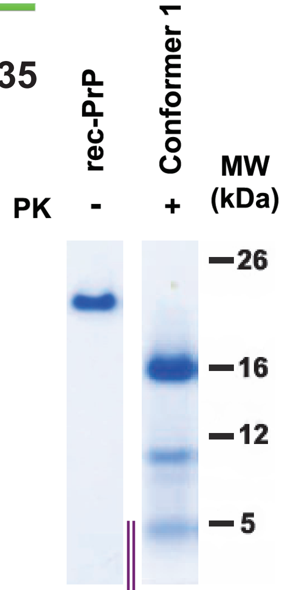

217 AA

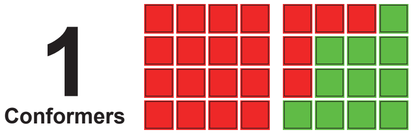

Tm^{Exp}: ND

ΔΔG: -2.47

In vitro studies

TgVole (1x) Not tested

Maned rat Not tested

In vivo studies

TgVole (1x) Not tested

Maned rat Not tested

pLDDT [86.7]

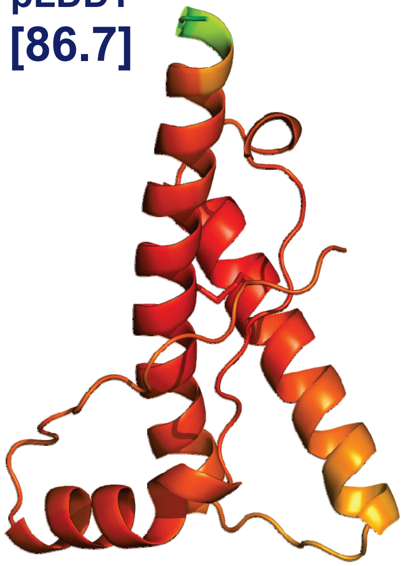

Protein code: 3608

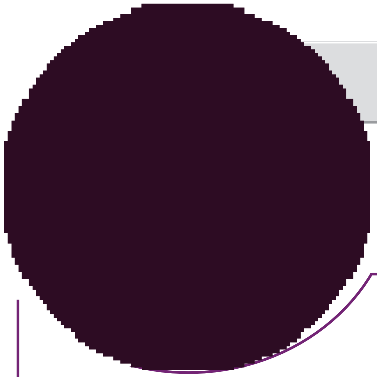

Yellow-bellied marmot

GenBank: XM_027955665

Marmota flaviventris

Rodentia	81/101
Sciuridae	7/13

P29%

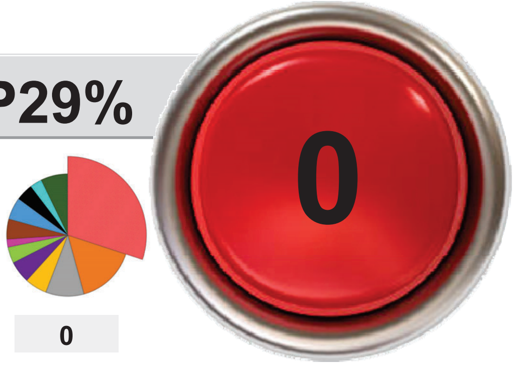

β2-α2 LOOP
PNQNSF
6

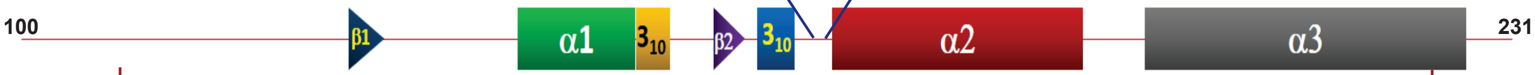

WGKPVKPKTNMKHMAGAAAAGAVVGGLGGYMLGSAMSRPMMHFGSDYEDRYYRENMNRYPSQVYYKPVDQYPNQNSFVHDCVNITIKQHTVTTTTKGDNFTETDIKIMERVVEQMCTTQYKQEAQAYYQRGSS-
N S I V I N W N R N N E V M V QK S EGR

PrP sequence differs by 16 amino acids from the bank vole PrP (see in red)

No deletions
No insertions
No polymorphic variants found

2 species sharing the same primary sequence

Groundhog
Marmota monax

Vancouver Island marmot
Marmota vancouverensis

Tm^{Exp}: ND

ΔΔG: -10.36

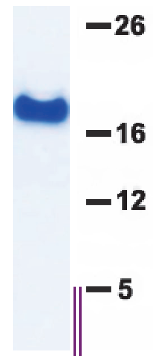

195 AA

NO MISFOLDING

0
Conformers

In vitro studies

NOT APPLICABLE

In vivo studies

NOT APPLICABLE

pLDDT
[86.34]

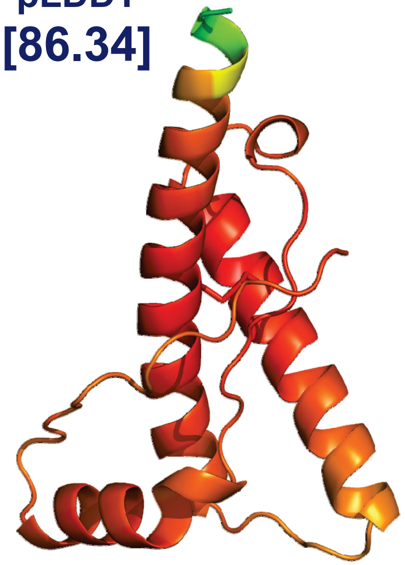

Protein code: 1099

Alpine marmot

GenBank: XM_015482632

Marmota marmota

Rodentia 81/101
Sciuridae 7/13

P29%

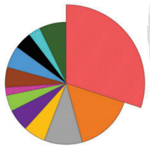

0

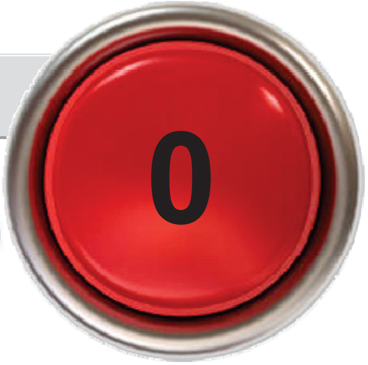

1 species sharing the same primary sequence

Himalayan marmot
Marmota himalayana

β2-α2 LOOP
PNQNSF
6

100 — β1 — α1 — 3_{10} — β2 — 3_{10} — α2 — α3 — 231

```
WGKPVKPKTNMKHMAGAAAAGAVVGGLGGYMLGSAMSRPMMHFGSDYEDRYYRENMNRYPSQVYYKPMDQYPNQNSFVHDCVNITIKQHTVTTTTKGDNFTETDIKIMERVVEQMCTTQYKQEAQAYYQRGSS-
 N  S    I  V                              I   N W             N    R V   N   N                    E      V M           V   QK S    EGR
```

PrP sequence differs by 17 amino acids from the bank vole PrP (see in red)

No deletions
No insertions
No polymorphic variants found

Tm^{Exp}: ND
ΔΔG: -8.03

PK
rec-PrP
-
MW (kDa)

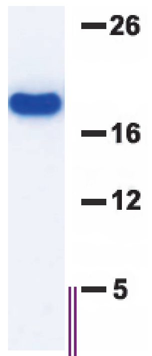

195 AA

NO MISFOLDING

0
Conformers

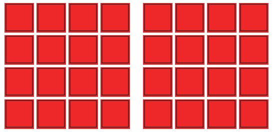

In vitro studies

NOT APPLICABLE

In vivo studies

NOT APPLICABLE

pLDDT
[86.42]

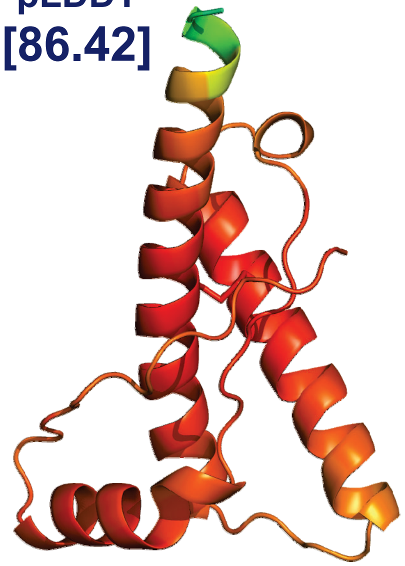

Protein code: 2667

Southern multimammate mouse

GenBank: XM_031371966

Mastomys coucha

Rodentia 81/101
Muridae 16/18

P82%

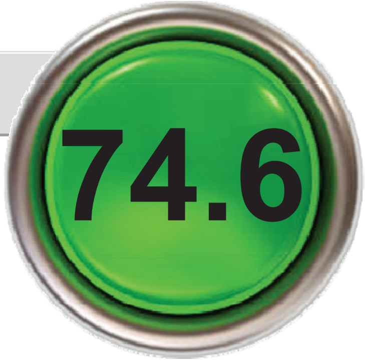

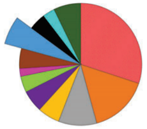

70-80

4 species sharing the same primary sequence

Natal multimammate mouse *Mastomys natalensis*

Muennink's spiny rat *Tokudaia muenninki*

Ryukyu spiny rat *Tokudaia osimensis*

Tokunoshima spiny rat *Tokudaia tokunoshimensis*

β2-α2 LOOP
SNQNNF
273

100 — β1 — α1 — 3_{10} — β2 — 3_{10} — α2 — α3 — 231

WNKPSKPKTNLKHVAGAAAAGAVVGGLGGYMLGSAMSRPMIHFGNDWEDRYYRENMYRYPNQVYYYRPVDQYSNQNNFVHDCVNITIKQHTVTTTTKGENFTETDVKMMERVVEQMCVTQYQKESQAYYEGRRS-

I N N S

PrP sequence differs by 3 amino acids from the bank vole PrP (see in red)

No deletions
No insertions
No polymorphic variants found

135

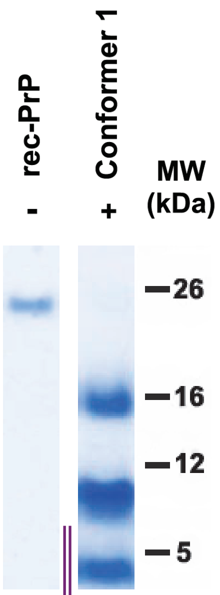

211 AA

1 Conformers

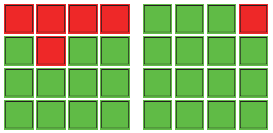

Tm^{Exp}: ND

ΔΔG: -6.17

In vitro studies

TgVole (1x) Not tested

Southern multimammate mouse Not tested

In vivo studies

TgVole (1x) Not tested

Southern multimammate mouse Not tested

pLDDT [86.01]

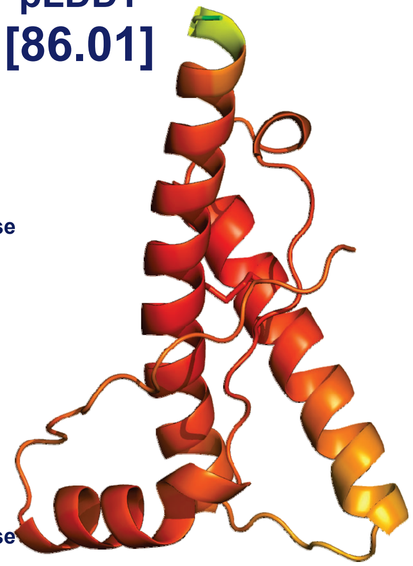

Protein code: 1098

Red spiny rat

GenBank: KF466947

Maxomys surifer

Rodentia 81/101
Muridae 16/18

P100%

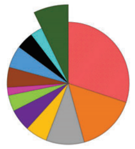

100

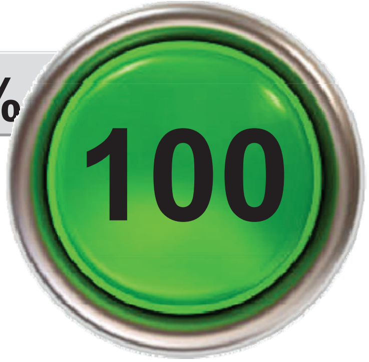

1 species sharing the same primary sequence

Gairdner's shrewmouse
Mus pahari

β2-α2 LOOP
SNQNNF
273

100 β1 α1 3_{10} β2 3_{10} α2 α3 231

WNKPSKPKTNLKHVAGAAAAGAVVGGLGGYMLGSAMSRPMIHFGNDWEDRYYRENMYRYPNQVYYRPVDQYSNQNNFVHDCVNITIKQHTVTTTTKGENFTETDVKMMERVVEQMCVTQYQKESQAYYDGRRS-
I N N E S

PrP sequence differs by 3 amino acids from the bank vole PrP (see in red)

No deletions
No insertions
No polymorphic variants found

135

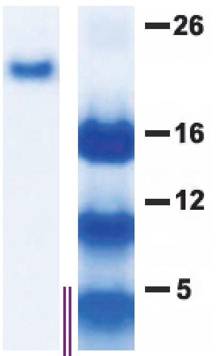

211 AA

1 Conformers

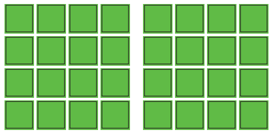

Tm^{Exp}: 67.6 ºC

ΔΔG: -0.97

In vitro studies

TgVole (1x) Not tested

Red spiny rat Not tested

In vivo studies

TgVole (1x) Not tested

Red spiny rat Not tested

pLDDT
[86.22]

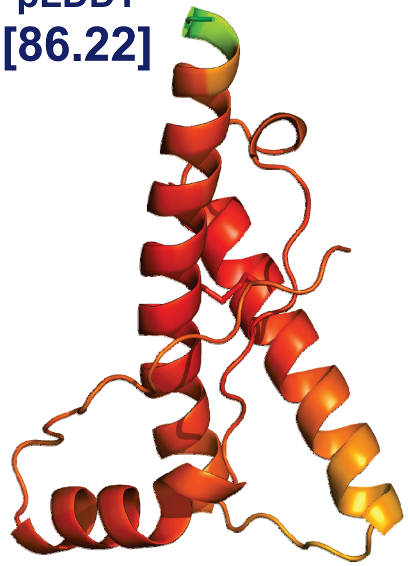

Protein code: 1440

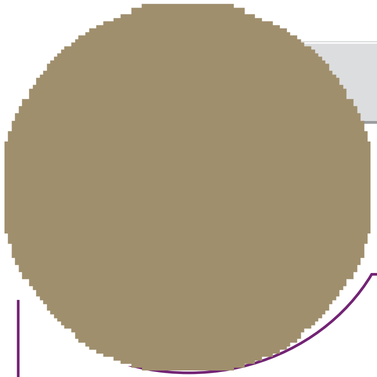

Libyan jird

GenBank: BK064826

Meriones libycus

Rodentia	81/101
Muridae	16/18

P74%

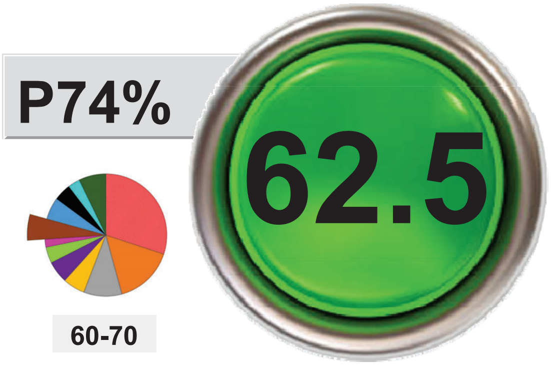

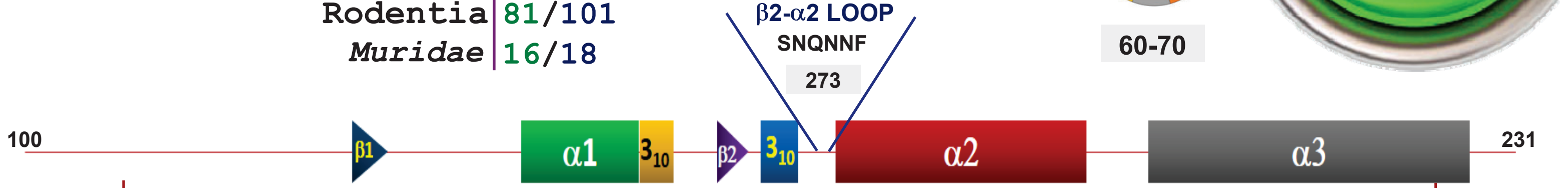

WNKPSKPKTNMKHVAGAAAAGAVVGGLGGYMLGSAMSRPMIHFGNDWEDRYYRENMYRYPNQVYYYRPVDQYSNQNNFVHDCVNITIKQHTVTTTTKGENFTETDVKMMERVVEQMCTTQYQKESQAYYDGRRS-

I N N V E S

PrP sequence differs by 4 amino acids from the bank vole PrP (see in red)

No deletions

No insertions

No polymorphic variants found

Distinct primary sequence across species

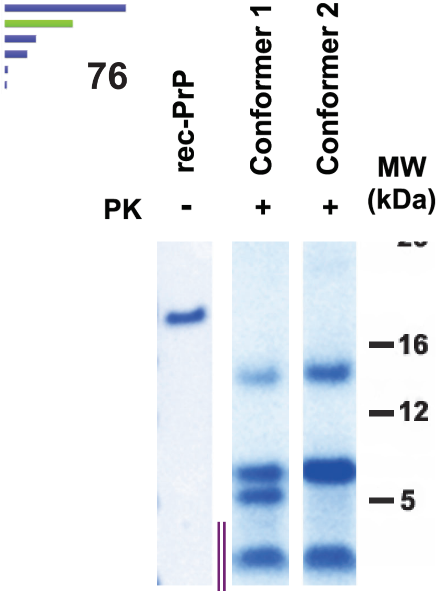

210 AA

2

Conformers

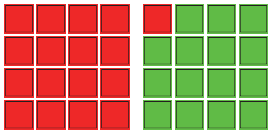

Tm^{Exp}: ND

ΔΔG: -15.45

In vitro studies

TgVole (1x)	Not tested
Libyan jird	Not tested

In vivo studies

TgVole (1x)	Not tested
Libyan jird	Not tested

pLDDT

[93.88]

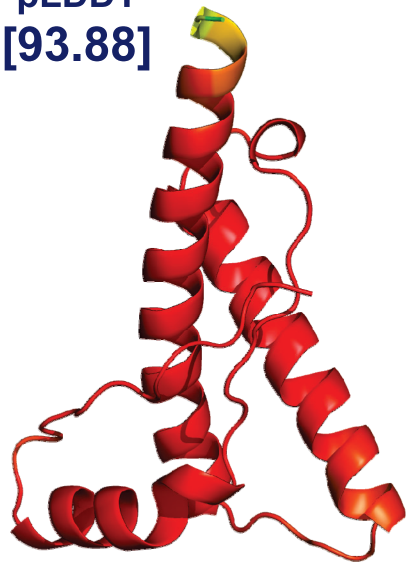

Protein code: 4640

Mongolian gerbil

GenBank: XM_021651076

Meriones unguiculatus

Rodentia	81/101
Muridae	16/18

1 species sharing the same primary sequence

Great gerbil
Rhombomys opimus

P100%

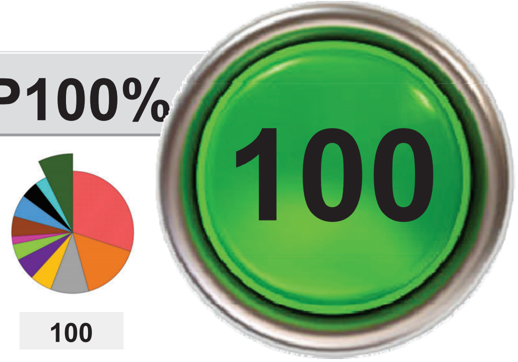

β2-α2 LOOP
SNQNNF
273

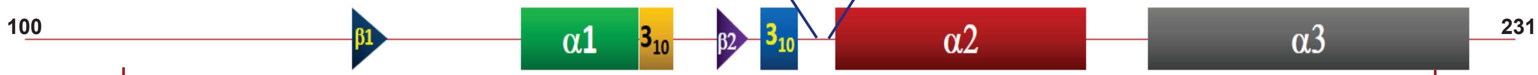

WNKPSKPKTNMKHVAGAAAAGAVVGGLGGYMLGSAMSRPMIHFGNDWEDRYYRENMYRYPNQVYYYRPVDQYSNQNNFVHDCVNITIKQHTVTTTTKGENFTETDVKMMERVVEQMCVTQYQKESQAYYDGRRS-

I N N E S

PrP sequence differs by 3 amino acids from the bank vole PrP (see in red)

No deletions
No insertions
No polymorphic variants found

135

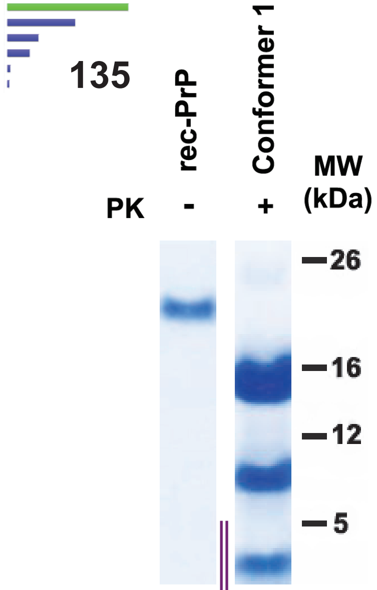

210 AA

1
Conformers

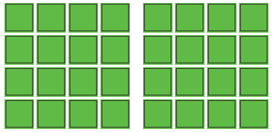

Tm[Exp]: ND

ΔΔG: -3.71

In vitro studies

TgVole (1x)	Propagate
Mongolian gerbil	Propagate

In vivo studies

TgVole (1x)	Infectious
Mongolian gerbil	Ongoing

pLDDT
[86.89]

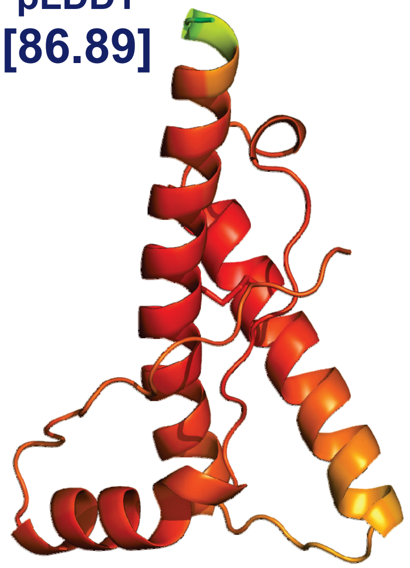

Protein code: 1111

Syrian hamster

GenBank: AH001830

Mesocricetus auratus

Rodentia 81/101
Cricetidae 25/27

P29%

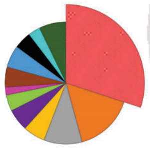

0

0

1 species sharing the same primary sequence

Turkish hamster
Mesocricetus brandti

β2-α2 LOOP
NNQNNF
164

100 β1 α1 3_{10} β2 3_{10} α2 α3 231

WNKPSKPKTNMKHMAGAAAAGAVVGGLGGYMLGSAMSRPMMHFGNDWEDRYYRENMNRYPNQVYYRPVDQYNNQNNFVHDCVNITIKQHTVTTTTKGENFTETDIKIMERVVEQMCTTQYQKESQAYYDGRRS-
I V I V M V E S

PrP sequence differs by 6 amino acids from the bank vole PrP (see in red)

No deletions
No insertions
No polymorphic variants found

Tm^{Exp}: ND

ΔΔG: -0.58

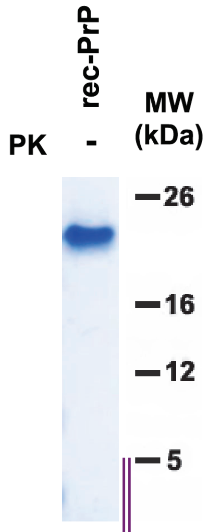

211 AA

NO MISFOLDING

0
Conformers

In vitro studies

NOT APPLICABLE

In vivo studies

NOT APPLICABLE

pLDDT
[86.06]

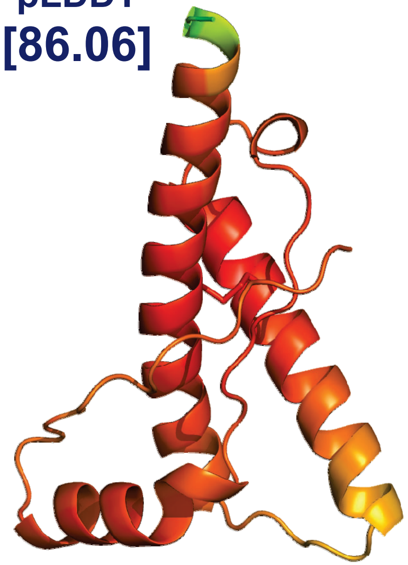

Protein code: 0133

Field vole

GenBank: AF367625

Microtus agrestis

Rodentia 81/101

Cricetidae 25/27

P100%

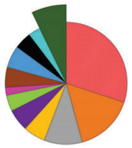

100

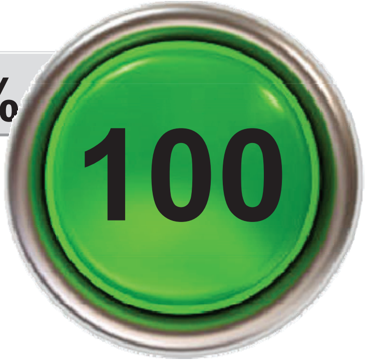

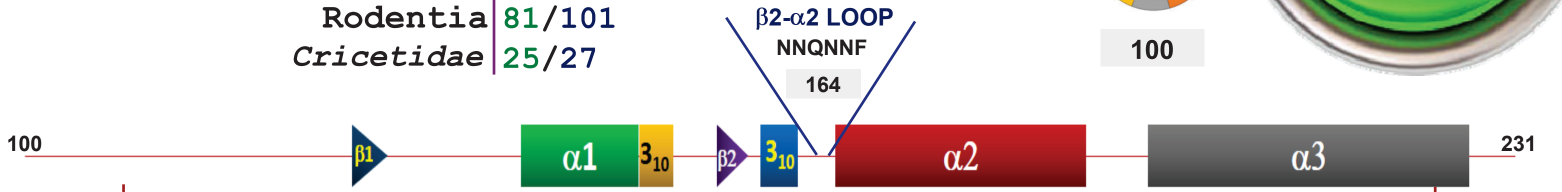

WNKPSKPKTNMKHVAGAAAAGAVVGGLGGYMLGSAMSRPMIHFGNDWEDRYYRENMNRYPNQVYYRPVDQYNNQNNFVHDCVNITIKQHTVTTTTKGENFTETDVKMMERVVEQMCVTQYQKESQAYYEGRHS-

I S

PrP sequence differs by 1 amino acids from the bank vole PrP (see in red)

No deletions

No insertions

No polymorphic variants found

Distinct primary sequence across species

135

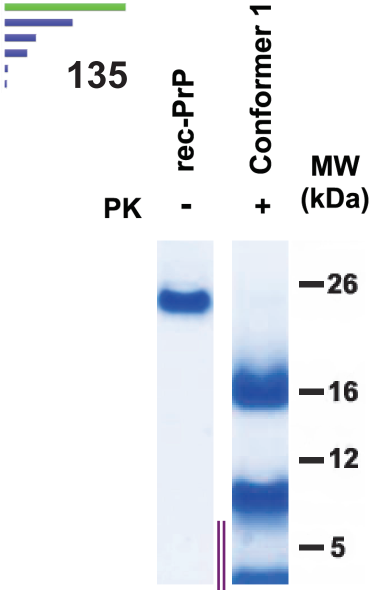

210 AA

1

Conformers

Tm^{Exp}: ND

ΔΔG: -1.99

In vitro studies

TgVole (1x) Not tested

Field vole Not tested

In vivo studies

TgVole (1x) Not tested

Field vole Not tested

pLDDT

[87.91]

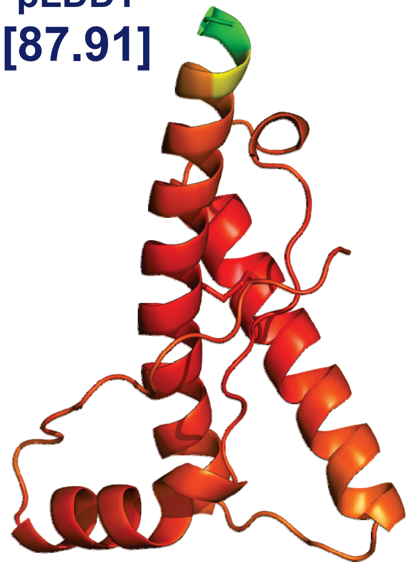

Protein code: 1423

Common vole

GenBank: BK064089

Microtus arvalis

Rodentia 81/101
Cricetidae 25/27

P100%

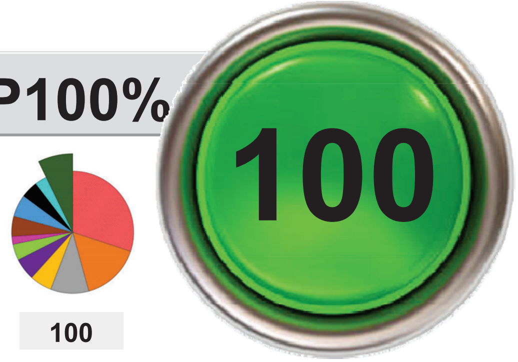

β2-α2 LOOP
NNQNNF
164

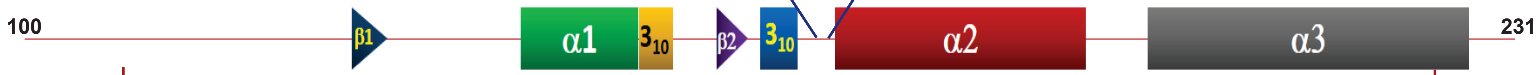

WNKPSKPKTNMKHVAGAAAAGAVVGGLGGYMLGSAMSRPMIHFGNDWEDRYYRENMNRYPNQVYYRPVDQYNNQNNFVHDCVNITIKQHTVTTTTKGENFTETDVKMMERVVEQMCVTQYQKESQAYYEGRRS-

I S

PrP sequence differs by 1 amino acids from the bank vole PrP (see in red)

No deletions
No insertions
No polymorphic variants found

Distinct primary sequence across species

135

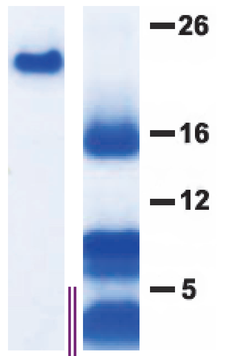

210 AA

1
Conformers

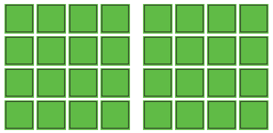

Tm^{Exp}: ND

ΔΔG: 2.34

pLDDT
[87]

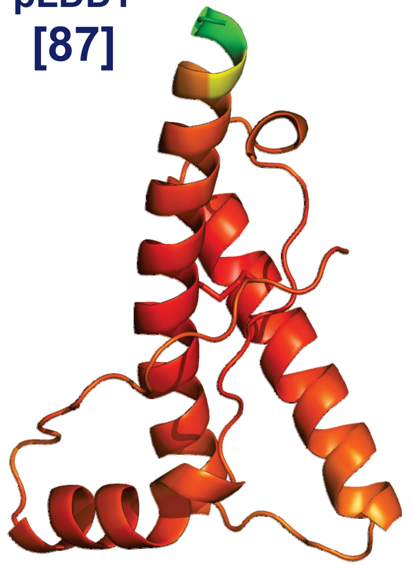

In vitro studies

TgVole (1x)	Not tested
Common vole	Not tested

In vivo studies

TgVole (1x)	Not tested
Common vole	Not tested

Protein code: 3268

Reed vole

GenBank: BK064090

Microtus fortis

Rodentia 81/101
Cricetidae 25/27

Distinct primary sequence across species

P92%

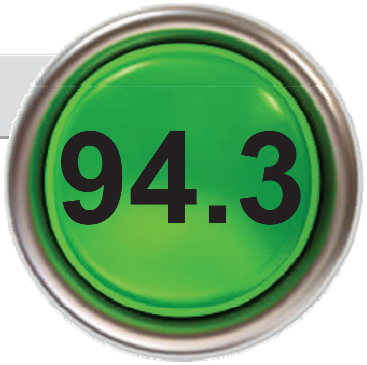

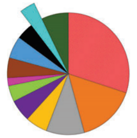

90-100

β2-α2 LOOP
SNQNNF
273

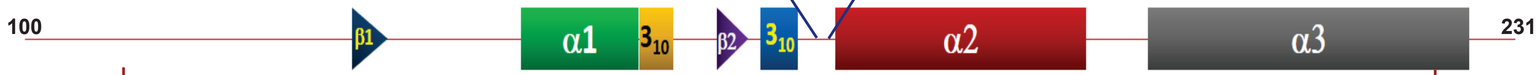


```
WNKPSKPKTNMKHVAGAAAAGAVVGGLGGYMLGSAMSRPMIHFGNDWEDRYYRENMNRYPNQVYYRPVDQYSNQNNFVHDCVNITIKQHTVTTTTKGENFTETDVKMMERVVEQMCVTQYQKESQAYYEGRHS-
           I                                                                     N                                                             S
```

PrP sequence differs by 2 amino acids from the bank vole PrP (see in red)

No deletions
No insertions
No polymorphic variants found

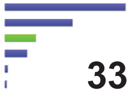

33

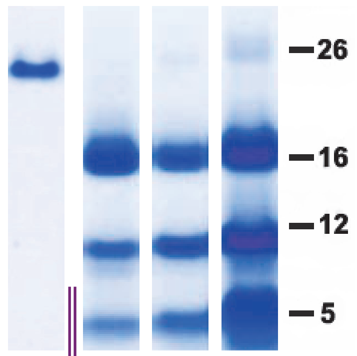

210 AA

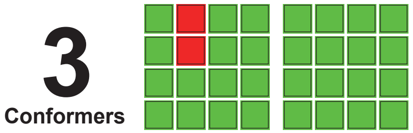

Tm[Exp]: ND

ΔΔG: -3.21

In vitro studies

TgVole (1x) Not tested

Reed vole Not tested

In vivo studies

TgVole (1x) Not tested

Reed vole Not tested

pLDDT
[88.37]

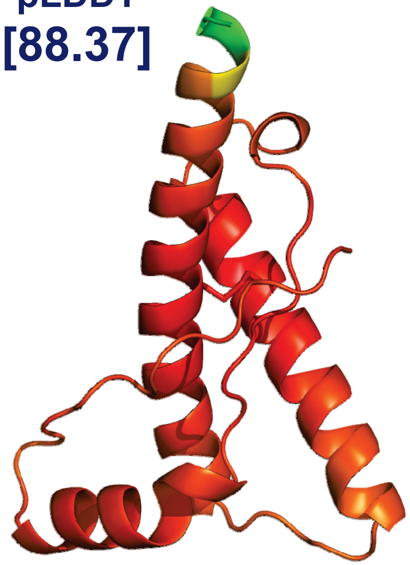

Protein code: 3269

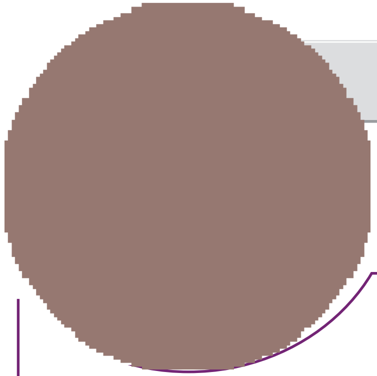

Prairie vole

GenBank: XM_005365568

Microtus ochrogaster

Rodentia 81/101
Cricetidae 25/27

P100%

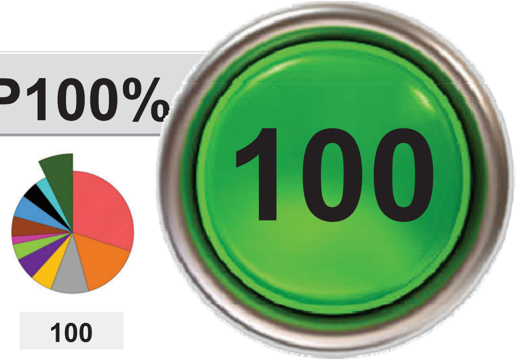

100

β2-α2 LOOP
NNQNNF
164

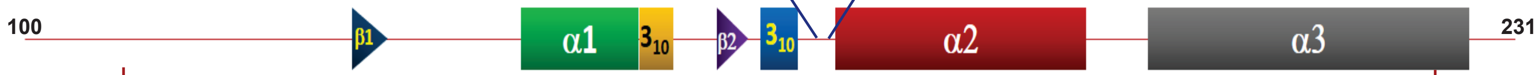

WNKPSKPKTNMKHVAGAAAAGAVVGGLGGYMLGSAMSRPMLHFGNDWEDRYYRENMNRYPNQVYYRPVDQYNNQNNFVHDCVNITIKQHTVTTTTKGENFTETDVKMMERVVEQMCVTQYQKESQAYYEGRYS-
I I S

PrP sequence differs by 2 amino acids from the bank vole PrP (see in red)

No deletions
No insertions
No polymorphic variants found

Distinct primary sequence across species

135

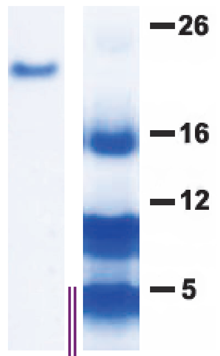

210 AA

1
Conformers

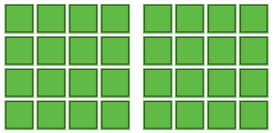

Tm^{Exp}: ND

ΔΔG: -13.33

In vitro studies

TgVole (1x) Not tested

Prairie vole Not tested

In vivo studies

TgVole (1x) Not tested

Prairie vole Not tested

pLDDT
[87.02]

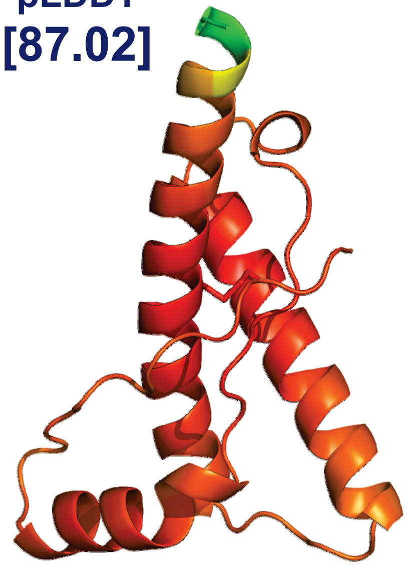

Protein code: 1094

Tundra vole

GenBank: BK064091

Microtus oeconomus

Rodentia 81/101
Cricetidae 25/27

P100%

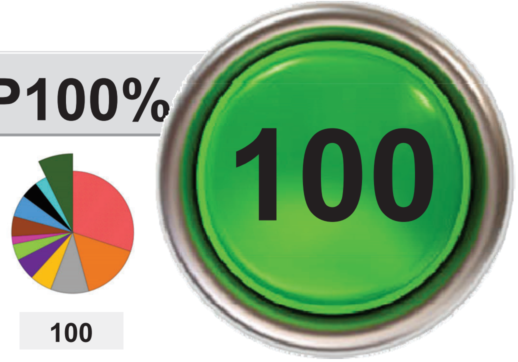

β2-α2 LOOP
NNQNNF
164

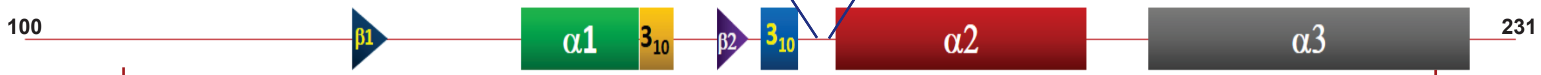

WNKPSKPKTNMKHVAGAAAAGAVVGGLGGYMLGSAMSRPMIHFGNDWEDRYYRENMNRYPNQVYYRPVDQYNNQNNFVHDCVNITIKQHTVTTTTKGENFTETDVKMMERVVEQMCVTQYQKESQAYYEGRQS-
I S

PrP sequence differs by 1 amino acids from the bank vole PrP (see in red)

No deletions
No insertions
No polymorphic variants found

Distinct primary sequence across species

135

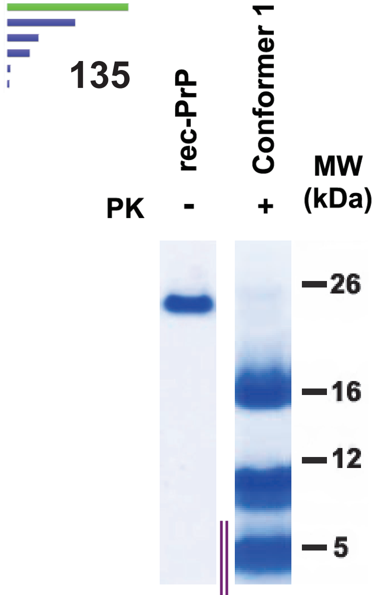

210 AA

1
Conformers

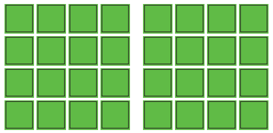

Tm^{Exp}: ND

ΔΔG: -3.36

pLDDT
[86.03]

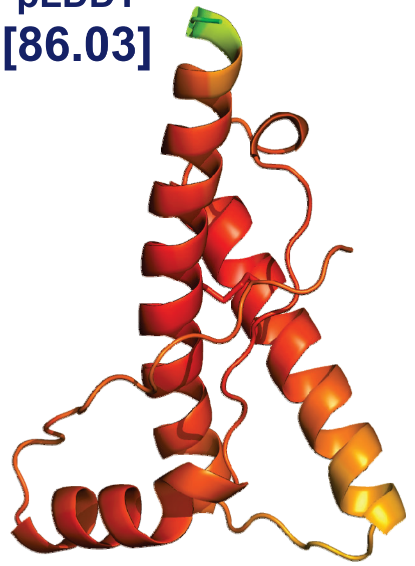

In vitro studies

TgVole (1x)	Not tested
Tundra vole	Not tested

In vivo studies

TgVole (1x)	Not tested
Tundra vole	Not tested

Protein code: 3270

Creeping vole

GenBank: BK064150

P68% **42.9**

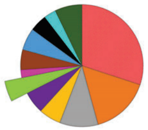

40-50

Microtus oregoni

Rodentia 81/101
Cricetidae 25/27

β2-α2 LOOP
NNQNNF
164

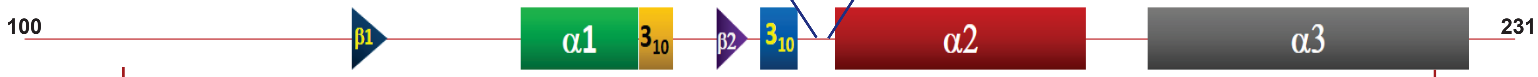

WNKPSKPKTNMKHVAGAAAAGAVVGGLGGYMLGSAMSRPMIHFGNDWEDRYYRENMYRYPNQVYYRPVDQYNNQNNFVHDCVNITIKQHTVTTTTKGENFTETDVKMMERVVEQMCVTQYQKESQAYYQGRYS-
I N E S

PrP sequence differs by 2 amino acids from the bank vole PrP (see in red)

No deletions
No insertions
No polymorphic variants found

Distinct primary sequence across species

135

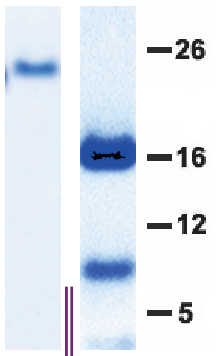

210 AA

1
Conformers

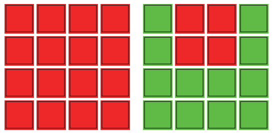

Tm^{Exp}: ND

ΔΔG: -4.39

In vitro studies

TgVole (1x) Not tested

Creeping vole Not tested

In vivo studies

TgVole (1x) Not tested

Creeping vole Not tested

pLDDT
[88.42]

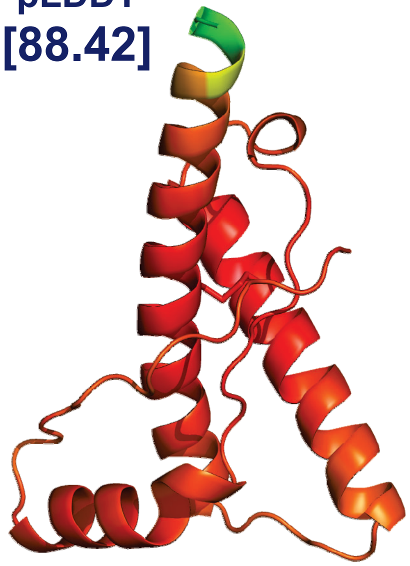

Protein code: 3388

Meadow vole

GenBank: GQ850541

Microtus pennsylvanicus

Rodentia	81/101
Cricetidae	25/27

3 species sharing the same primary sequence

- **California vole** *Microtus californicus*
- **Montane vole** *Microtus montanus*
- **Water vole** *Microtus richardsoni*

P92%

96.4

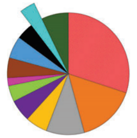

90-100

β2-α2 LOOP
NNQNNF
164

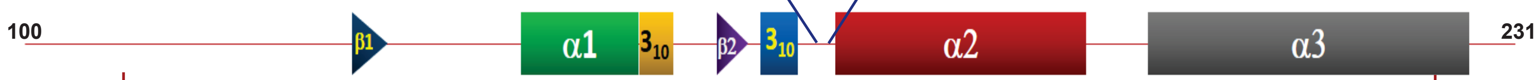

WNKPSKPKTNMKHVAGAAAAGAVVGGLGGYMLGSAMSRPMIHFGNDWEDRYYRENMNRYPNQVYYRPVDQYNNQNNFVHDCVNITIKQHTVTTTTKGENFTETDVKMMERVVEQMCVTQYQKESQAYYEGRYS-

I S

PrP sequence differs by 1 amino acids from the bank vole PrP (see in red)

No deletions

No insertions

No polymorphic variants found

135

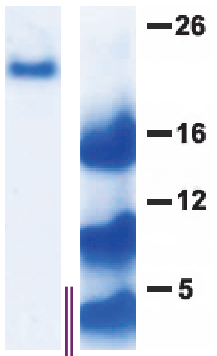

210 AA

1 Conformers

Tm[Exp]: ND

ΔΔG: -3.72

In vitro studies

TgVole (1x)	Not tested
Meadow vole	Not tested

In vivo studies

TgVole (1x)	Not tested
Meadow vole	Not tested

pLDDT [88.74]

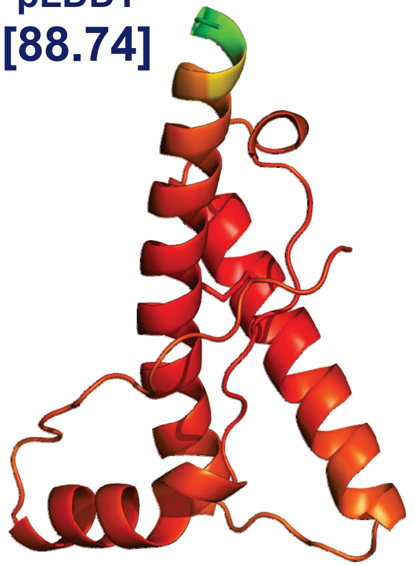

Protein code: 1422

Mouse

GenBank: NM_011170

Mus musculus

Rodentia 81/101
Muridae 16/18

β2-α2 LOOP
SNQNNF
273

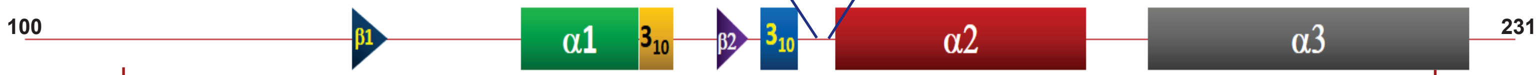

WNKPSKPKTNLKHVAGAAAAGAVVGGLGGYMLGSAMSRPMIHFGNDWEDRYYRENMYRYPNQVYYRPVDQYSNQNNFVHDCVNITIKQHTVTTTTKGENFTETDVKMMERVVEQMCVTQYQKESQAYYDGRRS-
I N N E S

PrP sequence differs by 3 amino acids from the bank vole PrP (see in red)

No deletions
No insertions
It shows polymorphic variants

P85%

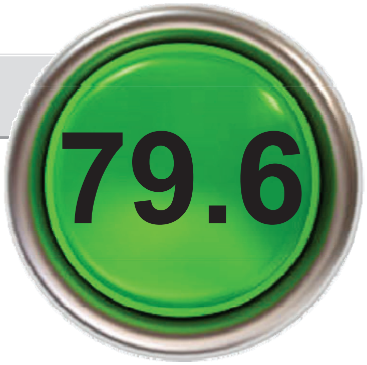

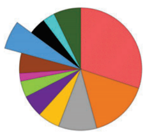

70-80

Tm^{Exp}: 68.3 ºC
ΔΔG: -3.3

16 species sharing the same primary sequence

- Ryukyu mouse *Mus caroli*
- Cook's mouse *Mus cookii*
- Fawn-colored mouse *Mus cervicolor*
- Sheath-tailed mouse *Mus fragilicauda*
- Wood mouse *Apodemus sylvaticus*
- Indo pencil-tailed tree mouse *Chiropodomys gliroides*
- Steppe mouse *Mus spicilegus*
- E broad-toothed field mouse *Apodemus mystacinus*
- African pygmy mouse *Mus minutoides*
- Algerian mouse *Mus spretus*
- Large Japanese field mouse *Apodemus speciosus*
- Delectable soft-furred mouse *Praomys delectorum*
- Desert mouse *Pseudomys desertor*
- Broad-toothed mouse *Mastacomys fuscus*
- Smoky mouse *Pseudomys fumeus*
- Rakali *Hydromys chrysogaster*

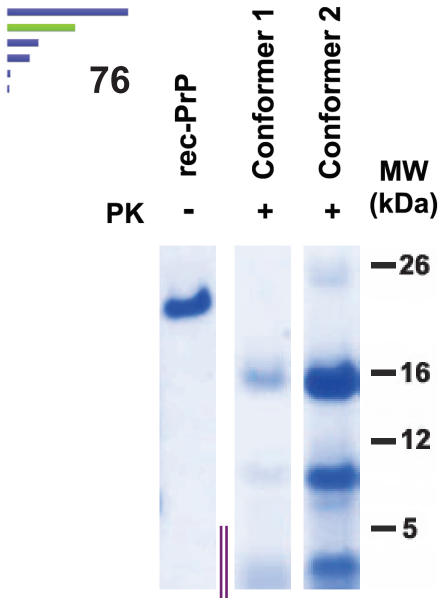

210 AA

2
Conformers

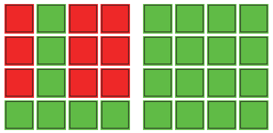

In vitro studies

TgVole (1x)	Propagate
Mouse	Propagate

In vivo studies

TgVole (1x)	Infectious
Mouse	Infectious

pLDDT
[86.15]

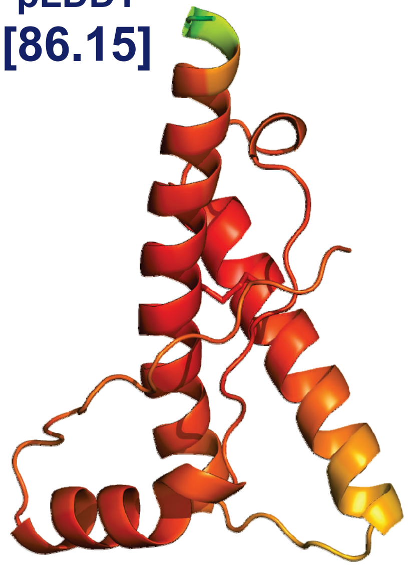

Protein code: 0113

Hazel dormouse

GenBank: BK064106

P52%

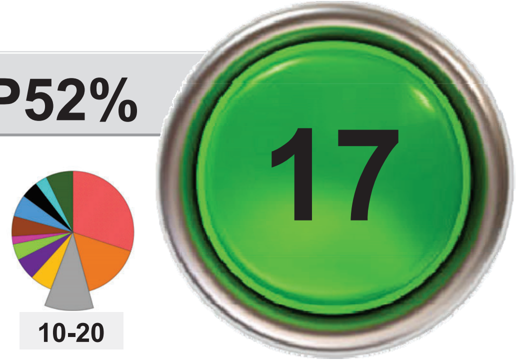

Muscardinus avellanarius

Rodentia	81/101
Gliridae	3/3

Distinct primary sequence across species

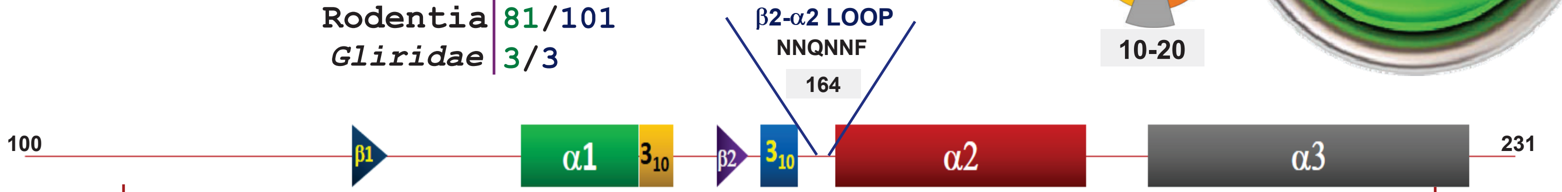

WNKPNKPKTNMKHMAGAAAAGAVVGGLGGYMVGSAMSRPPMHFGNDYEDRYYRENMNRYPNQVYYKPVDQYNNQNNFVHDCVNITVKQHTVTTTTKGENFTETDVKIMERVVEQMCITQYQKEAQAYYQRGAS-

S I V L MI W R I M V S EGRS

PrP sequence differs by 11 amino acids from the bank vole PrP (see in red)

No deletions

No insertions

No polymorphic variants found

76

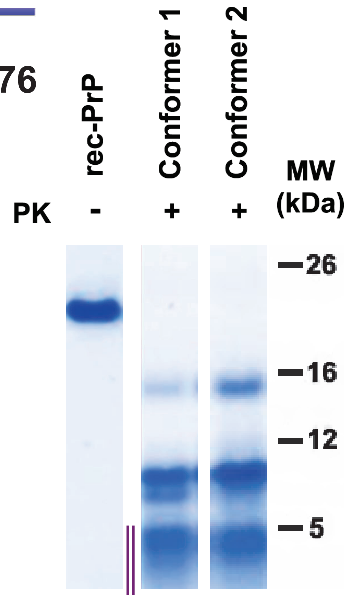

214 AA

2

Conformers

Tm^Exp: ND

ΔΔG: -5.66

In vitro studies

TgVole (1x) Not tested

Hazel dormouse Not tested

In vivo studies

TgVole (1x) Not tested

Hazel dormouse Not tested

pLDDT [86.22]

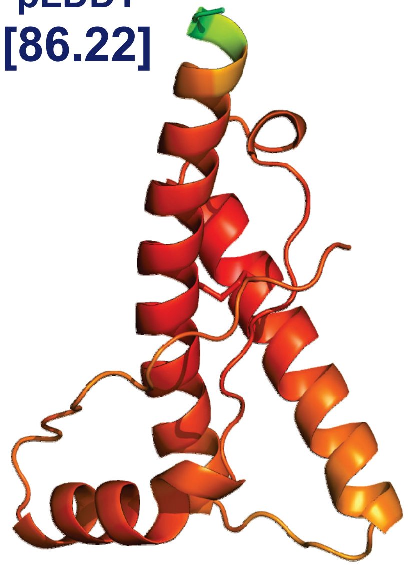

Protein code: 3285

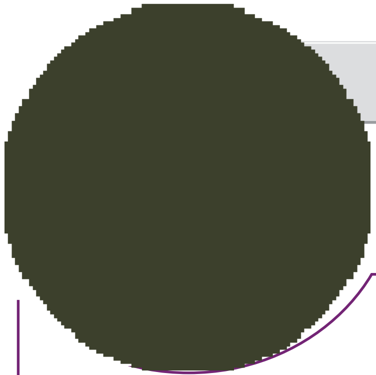

Coypu

GenBank: BK064010

Myocastor coypus

Rodentia	81/101
Echimyidae	0/1

P29%

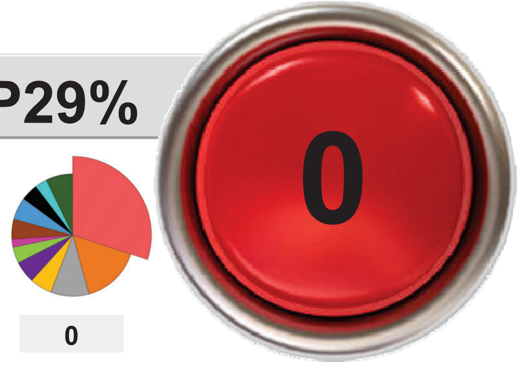

β2-α2 LOOP
SNQNNF
273

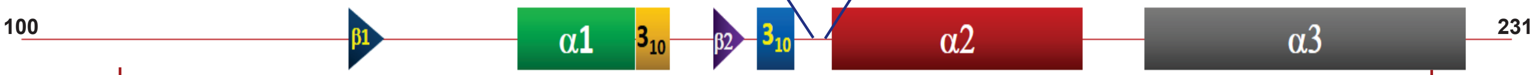

WNKPSKPKTNMKHVAGAAAAGAVVGGLGGYMLGSAMSRPLIHFGNDYEDRYYRENMYRYPNQVYYRPVDQYSNQNNFVQDCVNITIKQHTVTTTTKGENFTETDVKIMERVLQQMCTTQYQKESQAYYYHGRAG-

I M W N N H M VE V EGRSS

PrP sequence differs by 10 amino acids from the bank vole PrP (see in red)

No deletions

No insertions

No polymorphic variants found

Distinct primary sequence across species

Tm^{Exp}: ND

ΔΔG: ND

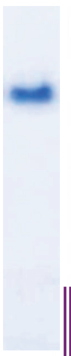

202 AA

NO MISFOLDING

0
Conformers

In vitro studies

NOT APPLICABLE

In vivo studies

NOT APPLICABLE

pLDDT
[84.21]

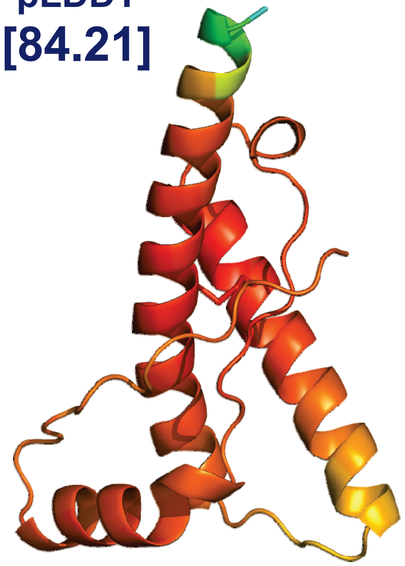

Protein code: 3171

Southern red-backed vole

GenBank: GQ850538

P100%

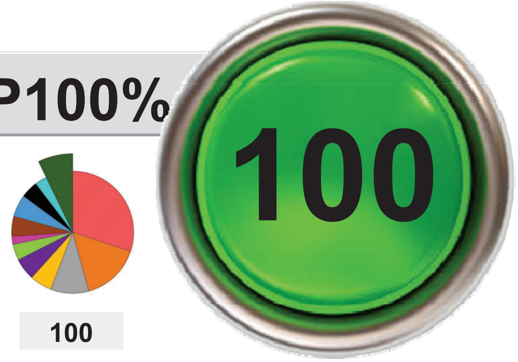

Myodes gapperi

Rodentia 81/101

Cricetidae 25/27

β2-α2 LOOP
SNQNNF
273

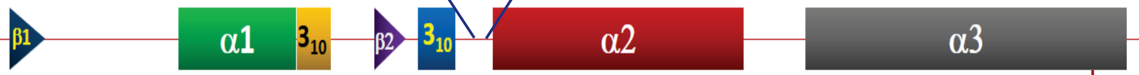

WNKPSKPKTNMKHVAGAAAAGAVVGGLGGYMLGSAMSRPMIHFGNDWEDRYYRENMNRYPNQVYYRPVDQYSNQNNFVHDCVNITIKQHTVTTTTKGENFTETDVKMMERVVEQMCVTQYQKESQAYYEGRSS-

I N

PrP sequence differs by 2 amino acids from the bank vole PrP (see in red)

No deletions

No insertions

No polymorphic variants found

Distinct primary sequence across species

76

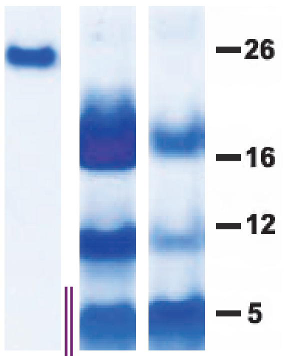

211 AA

2

Conformers

Tm^{Exp}: ND

ΔΔG: 4.84

In vitro studies

Not tested

TgVole (1x)

Not tested

Southern red-backed vole

In vivo studies

Not tested

TgVole (1x)

Not tested

Southern red-backed vole

pLDDT [85.29]

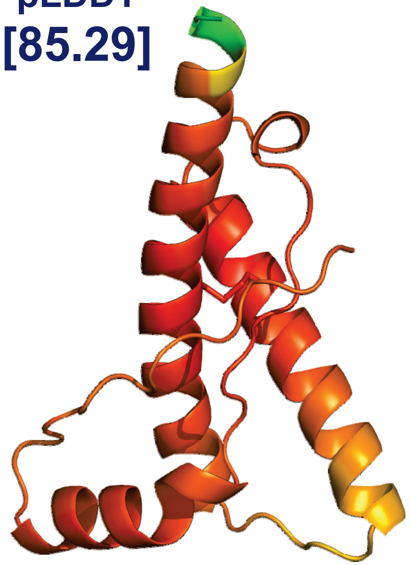

Protein code: 1390

Bank vole

GenBank: AF367624

P100%

100

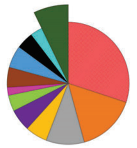

100

Myodes glareolus

Rodentia 81/101

Cricetidae 25/27

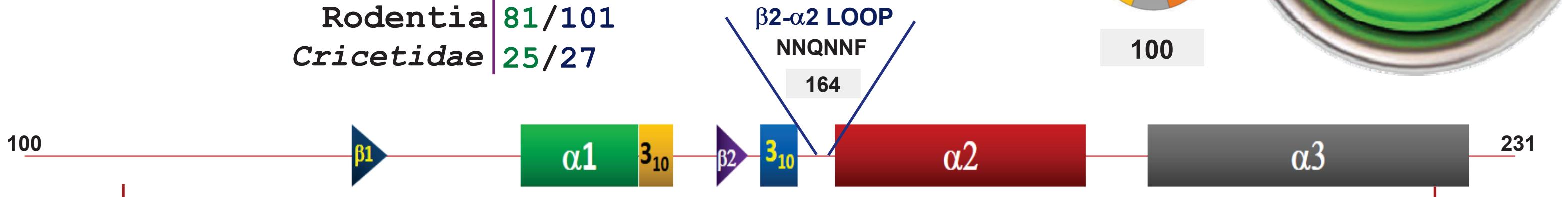

WNKPSKPKTNIKHVAGAAAAGAVVGGLGGYMLGSAMSRPMIHFGNDWEDRYYRENMNRYPNQVYYRPVDQYNNQNNFVHDCVNITIKQHTVTTTTKGENFTETDVKMMERVVEQMCVTQYQKESQAYYEGRSS-

PrP sequence differs by 0 amino acids from the bank vole PrP (see in red)

No deletions

No insertions

It shows polymorphic variants

Distinct primary sequence across species

25

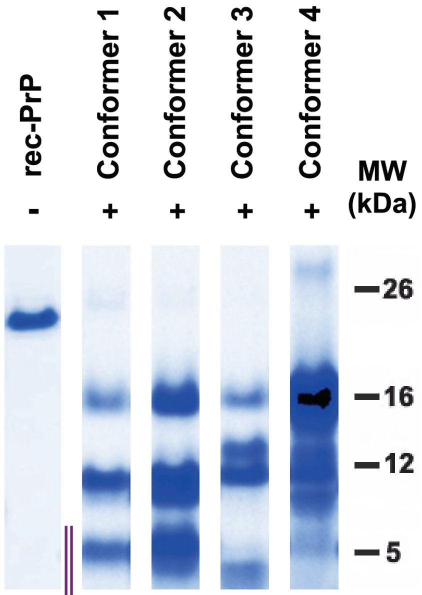

211 AA

4

Conformers

Tm[Exp]: 66.5 ºC

ΔΔG: 0

In vitro studies

TgVole (1x) Propagate

Mouse Propagate

In vivo studies

TgVole (1x) Infectious

Mouse Infectious

pLDDT [87.54]

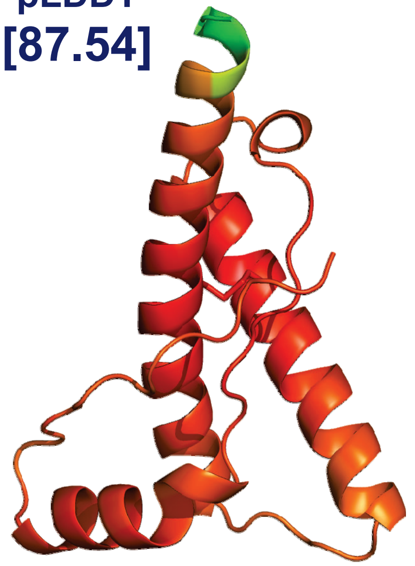

Protein code: 0092

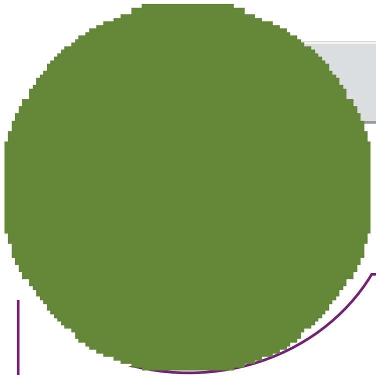

Shergylaensis vole

GenBank: BK064207

Neodon shergylaensis

Rodentia 81/101
Cricetidae 25/27

P74%

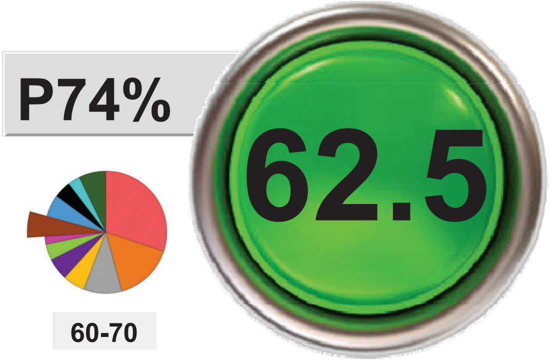

β2-α2 LOOP
NNQNNF
164

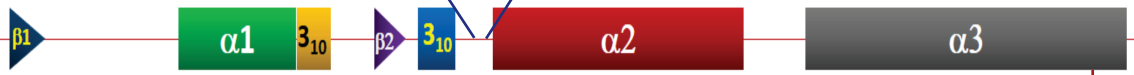

WNKPSKPKTNMKHVAGAAAAGAVVGGLGGYMLGSAMSRPMIHFGNDWEDRYYRENMNRYPNQVYYRPVDQYNNQNNFVHDCVNITIKQHTVTTTTKGENFTETDVKMMERVVEQMCVTQYEKESQAYYEGRRS-
I Q S

PrP sequence differs by 2 amino acids from the bank vole PrP (see in red)

No deletions
No insertions
No polymorphic variants found

Distinct primary sequence across species

135

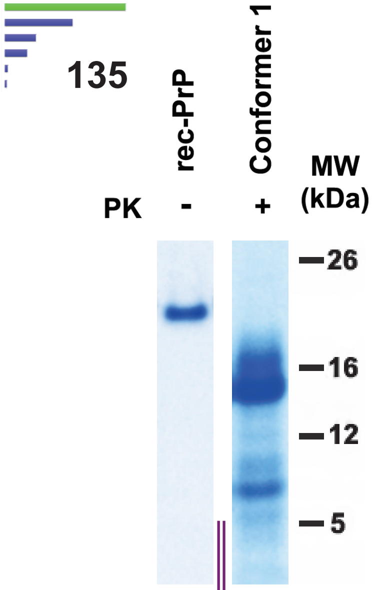

209 AA

1
Conformers

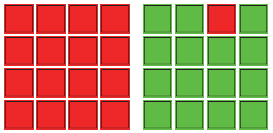

Tm^{Exp}: ND

ΔΔG: -15.47

In vitro studies

TgVole (1x) Not tested

Shergylaensis vole Not tested

In vivo studies

TgVole (1x) Not tested

Shergylaensis vole Not tested

pLDDT
[92.02]

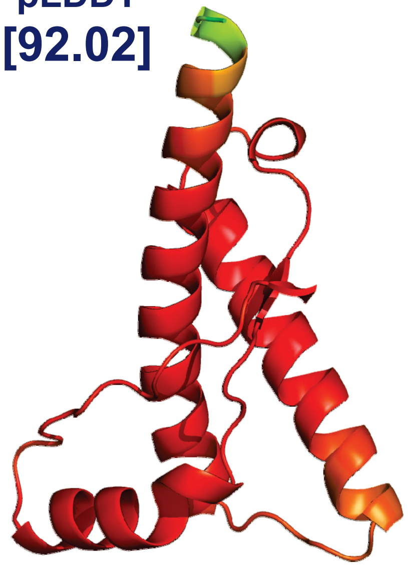

Protein code: 4584

Desert woodrat

GenBank: BK064110

P91%

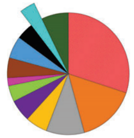

90-100

92.9

Neotoma lepida

Rodentia 81/101
Cricetidae 25/27

β2-α2 LOOP
SNQNNF
273

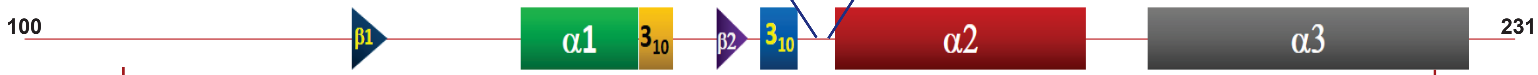

WNKPSKPKTNMKHVAGAAAAGAVVGGLGGYMLGSAMSRPMIHFGNDWEDRYYRENMYRYPNQVYYRPVDQYSNQNNFVHDCVNITIKQHTVTTTTKGENFTETDVKMMERVVEQMCVTQYQKESQAYYEGRRS-
I N N S

PrP sequence differs by 3 amino acids from the bank vole PrP (see in red)

No deletions
No insertions
No polymorphic variants found

Distinct primary sequence across species

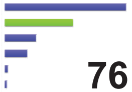

76

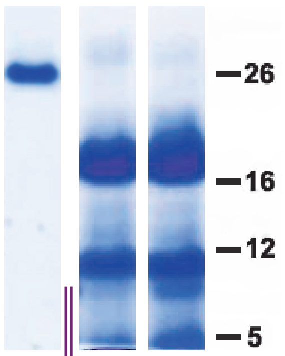

210 AA

2
Conformers

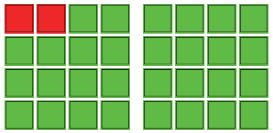

Tm^{Exp}: ND

ΔΔG: -3.66

In vitro studies

TgVole (1x) Not tested

Desert woodrat Not tested

In vivo studies

TgVole (1x) Not tested

Desert woodrat Not tested

pLDDT
[86.56]

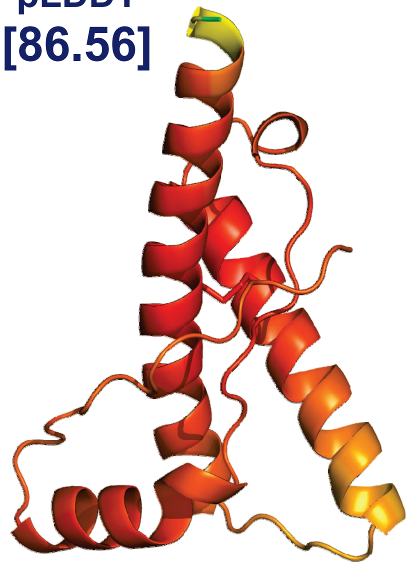

Protein code: 3290

Common degu

GenBank: XM_023718589

Octodon degus

Rodentia 81/101

Octodontidae 3/3

P81%

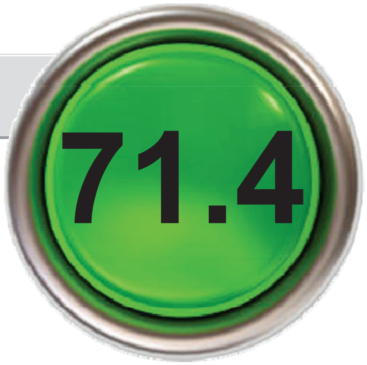

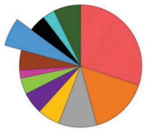

70-80

β2-α2 LOOP
SNQNNF
273

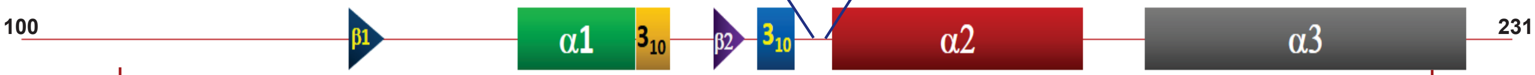

WNKPSKPKTNMKHVAGAAAAGAVVGGLGGYMLGSAMSRPLIHFGNDYEDRYYRENMYRYPNQVYYKPVDQYSNQNNFVQDCVNITIKQHTVTTTTKGENFTETDVKIMERVLQQMCTTQYQKESQAYYYHGRAG-

I M W N R N H M VE V EGRSS

PrP sequence differs by 11 amino acids from the bank vole PrP (see in red)

No deletions

No insertions

No polymorphic variants found

Distinct primary sequence across species

76

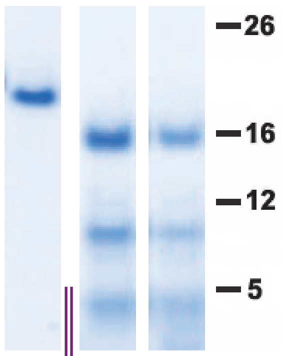

202 AA

2

Conformers

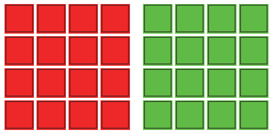

Tm^{Exp}: ND

ΔΔG: ND

In vitro studies

TgVole (1x) Not tested

Common degu Not tested

In vivo studies

TgVole (1x) Not tested

Common degu Not tested

pLDDT [86.1]

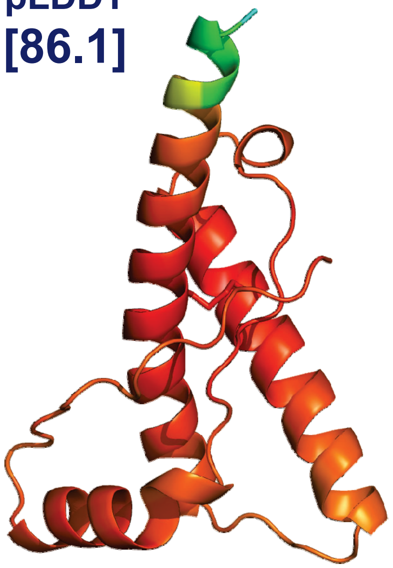

Protein code: 1003

Mountain viscacha rat

GenBank: BK064011

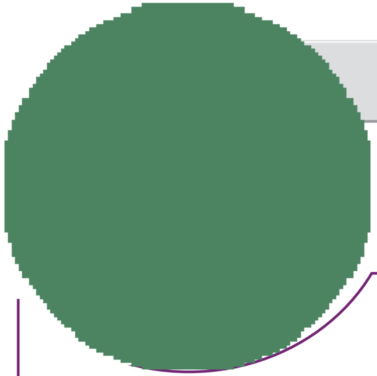

P64%

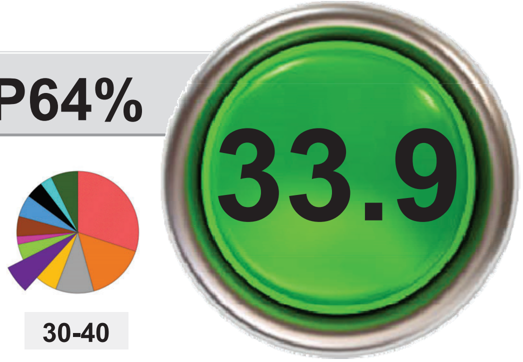

Octomys mimax

Rodentia	81/101
Octodontidae	3/3

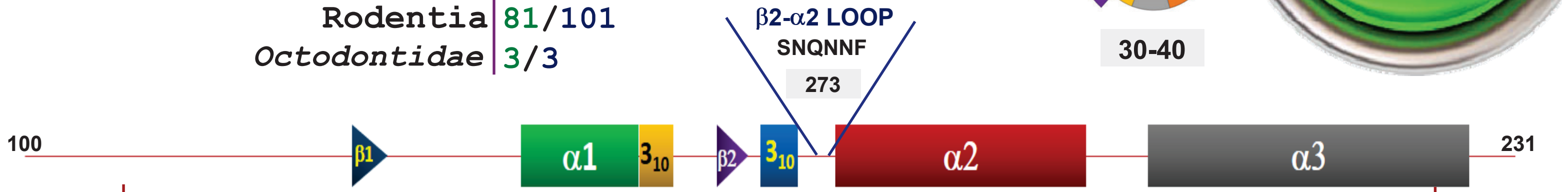

WNKPSKPKTNMKHVAGAAAAGAVVGGLGGYMLGSAMSRPLIHFGNDYEDRYYRENMYRYPNQVYYKPVDQYSNQNNFVQDCVNITIKQHTVTTTTKGENFTETDVKIMERVLQQMCTTQYQKESQAYYYHGMAG-

I M W N R N H M VE V EGRSS

PrP sequence differs by 11 amino acids from the bank vole PrP (see in red)

No deletions

No insertions

No polymorphic variants found

Distinct primary sequence across species

135

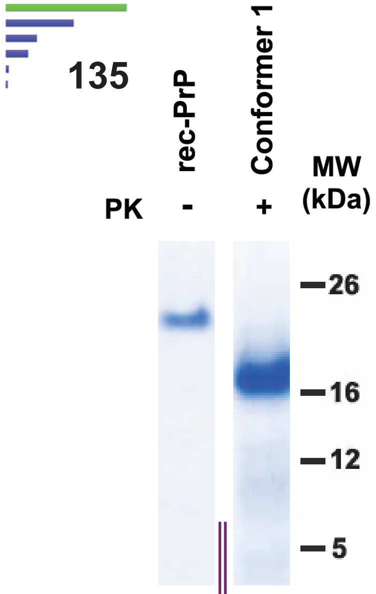

202 AA

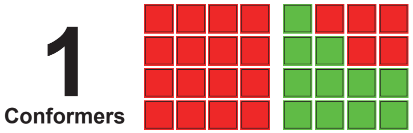

Tm^{Exp}: ND

ΔΔG: ND

In vitro studies

TgVole (1x) Not tested

Mountain viscacha rat Not tested

In vivo studies

TgVole (1x) Not tested

Mountain viscacha rat Not tested

pLDDT [84.52]

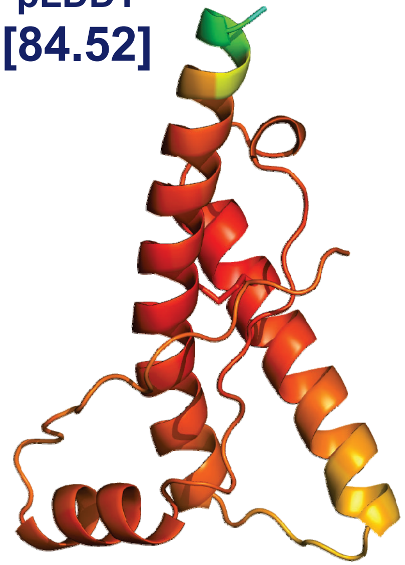

Protein code: 3172

Muskrat

GenBank: BK064026

Ondatra zibethicus

Rodentia 81/101

Cricetidae 25/27

Arctic lemming
Dicrostonyx torquatus

1 species sharing the same primary sequence

P51% **16.1**

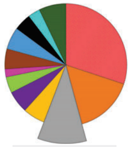

10-20

β2-α2 LOOP
NNQNNF
164

100 β1 α1 3_{10} β2 3_{10} α2 α3 231

WNKPSKPKTNMKHVAGAAAAGAVVGGLGGYMLGSAMSRPMIHFGNDWEDRYYRENMNRYPNQVYYRPVDQYNNQNNFVHDCVNITIKQHTVTTTTKGENFTETDVKMMERVVEQMCVTQYQKESQAYYEGRSS-

I

PrP sequence differs by 1 amino acids from the bank vole PrP (see in red)

No deletions
No insertions
No polymorphic variants found

135

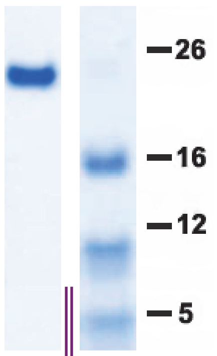

211 AA

1 Conformers

Tm^{Exp}: ND

ΔΔG: 0.61

In vitro studies

TgVole (1x) Not tested

Muskrat Not tested

In vivo studies

TgVole (1x) Not tested

Muskrat Not tested

pLDDT [85.74]

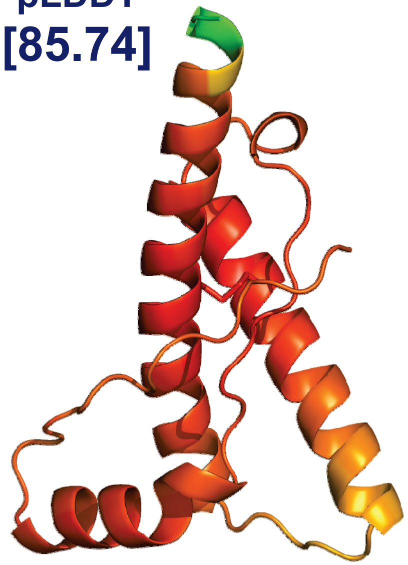

South African springhare

GenBank: BK064021

Pedetes capensis

Rodentia 81/101
Pedetidae 1/1

P100%

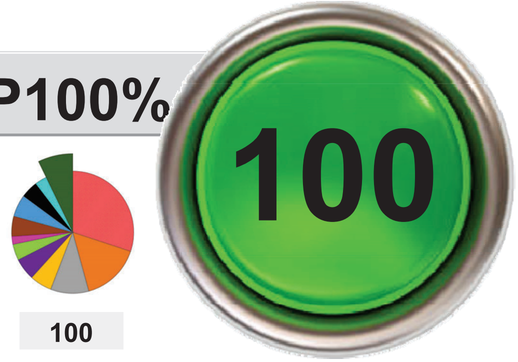

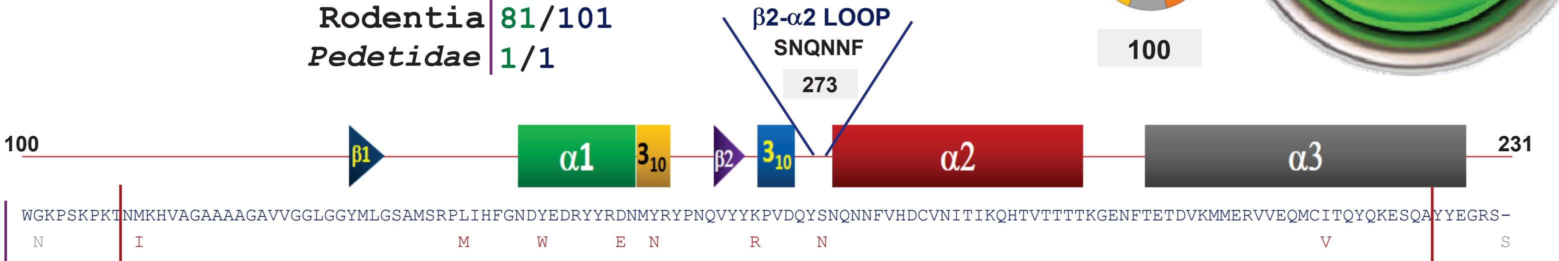

PrP sequence differs by 8 amino acids from the bank vole PrP (see in red)

No deletions
No insertions
No polymorphic variants found

Distinct primary sequence across species

135

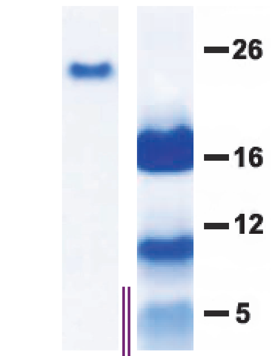

212 AA

1 Conformers

Tm^{Exp}: ND

ΔΔG: ND

In vitro studies

TgVole (1x) Not tested

South African springhare Not tested

In vivo studies

TgVole (1x) Not tested

South African springhare Not tested

pLDDT [87.98]

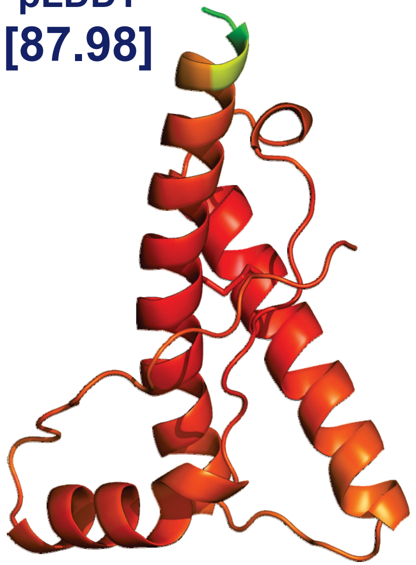

Protein code: 3186

Little pocket mouse

GenBank: BK064022

P34%

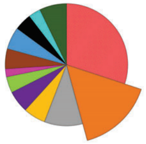

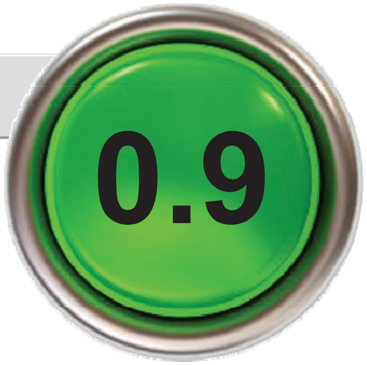

0-10

Perognathus longimembris

Rodentia 81/101

Heteromyidae 5/5

β2-α2 LOOP
NNQNNF
164

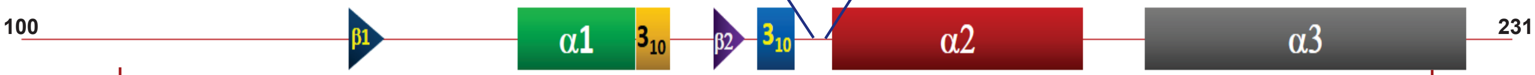

WNKPNKPKTSMKHMAGAAAAGAVVGGLGGYMLGSAMSRPLMHFGNDYEDRYYRENMNRYPSQVYYYKPVDQYNNQNNFVHDCVNITVKQHTVTTTTKGDNFTETDVKIMERMVEQMCITQYQKESRAYYEPRS-

S NI V MI W N R I E M V V Q G S

PrP sequence differs by 14 amino acids from the bank vole PrP (see in red)

No deletions

No insertions

No polymorphic variants found

135

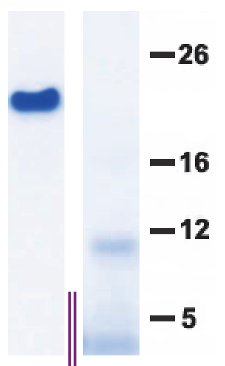

203 AA

1
Conformers

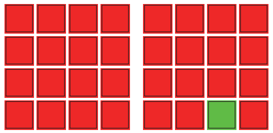

Tm^{Exp}: ND

ΔΔG: ND

In vitro studies

TgVole (1x) Not tested

Little pocket mouse Not tested

In vivo studies

TgVole (1x) Not tested

Little pocket mouse Not tested

pLDDT
[84.21]

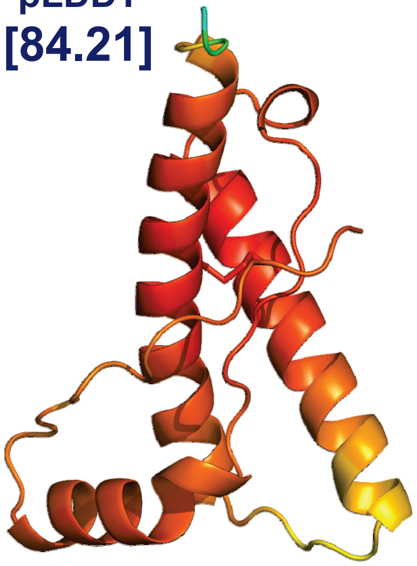

Distinct primary sequence across species

Protein code: 3187

Texas mouse

GenBank: BK064023

P59%

26.8

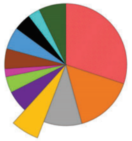

20-30

Peromyscus attwateri

Rodentia	81/101
Cricetidae	25/27

Northern rock mouse
Peromyscus nasutus

1 species sharing the same primary sequence

β2-α2 LOOP
NNQNNF
164

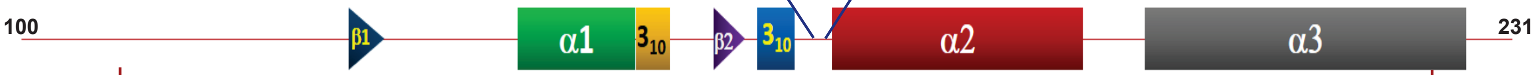

WNKPSKPKTNMKHVAGAAAAGAVVGGLGGYMLGSAMSRPMIHFGNDWEDRYYRENMYRYPNQVYYRPVDQYNNQNNFVHDCVNITIKQHTVTTTTKGENFTETDVKMMERVVEQMCVTQYQKESQAYYDGRRS-
I N E S

PrP sequence differs by 2 amino acids from the bank vole PrP (see in red)

No deletions
No insertions
No polymorphic variants found

135

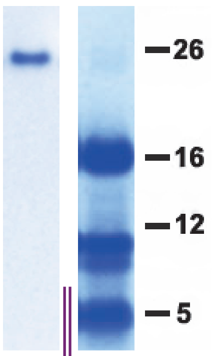

211 AA

1
Conformers

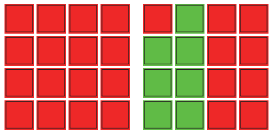

Tm^{Exp}: ND

ΔΔG: 1.56

In vitro studies

TgVole (1x) Not tested

Texas mouse Not tested

In vivo studies

TgVole (1x) Not tested

Texas mouse Not tested

pLDDT
[86.14]

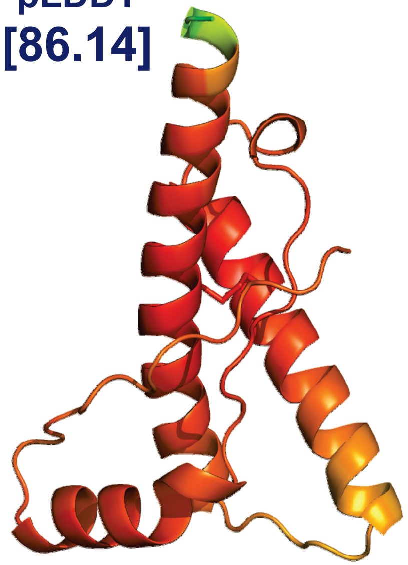

Protein code: 3188

California mouse

GenBank: BK064122

Peromyscus californicus insignis

Rodentia 81/101
Cricetidae 25/27

Cactus mouse
Peromyscus eremicus

1 species sharing the same primary sequence

P90%

91.1

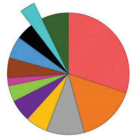

90-100

β2-α2 LOOP
NNQNNF
164

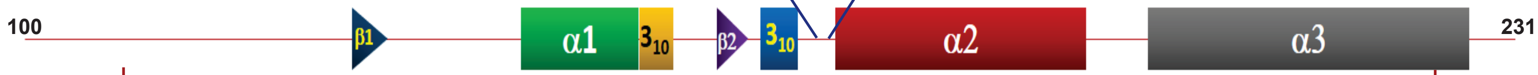

WNKPSKPKTNMKHVAGAAAAGAVVGGLGGYMLGSAMSRPMLHFGNDWEDRYYRENMYRYPNQVYYRPVDQYNNQNNFVHDCVNITIKQHTVTTTTKGENFTETDVKMMERVVEQMCVTQYQKESQAYYDGRRS-

I I N E S

PrP sequence differs by 3 amino acids from the bank vole PrP (see in red)

No deletions
No insertions
No polymorphic variants found

135

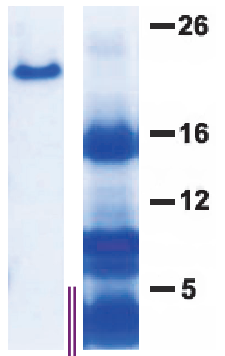

211 AA

1
Conformers

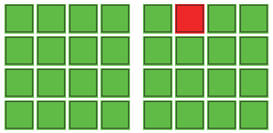

Tm^{Exp}: ND
ΔΔG: -8.01

In vitro studies

TgVole (1x) Not tested
California mouse Not tested

In vivo studies

TgVole (1x) Not tested
California mouse Not tested

pLDDT
[85.67]

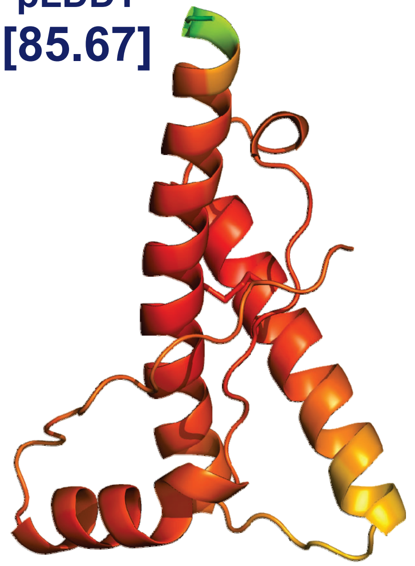

Protein code: 3303

Canyon mouse

GenBank: BK064807

Peromyscus crinitus

Rodentia 81/101
Cricetidae 25/27

P34%

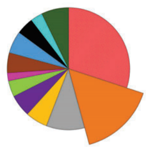

0.9

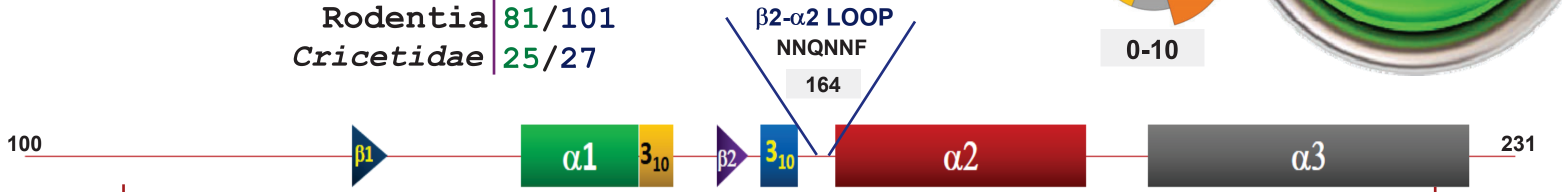

WNKPSKPKTNMKHMAGAAAAGAVVGGLGGYMLGSAMSRPMIHFGNDWEDRYYRENMYRYPNQVYYRPVDQYNNQNNFVHDCVNITIKQHTVTTTTKGENFTETDVKMMERVVEQMCVTQYQKESQAYYDGRRS-
I V N E S

PrP sequence differs by 3 amino acids from the bank vole PrP (see in red)

No deletions
No insertions
No polymorphic variants found

Distinct primary sequence across species

135

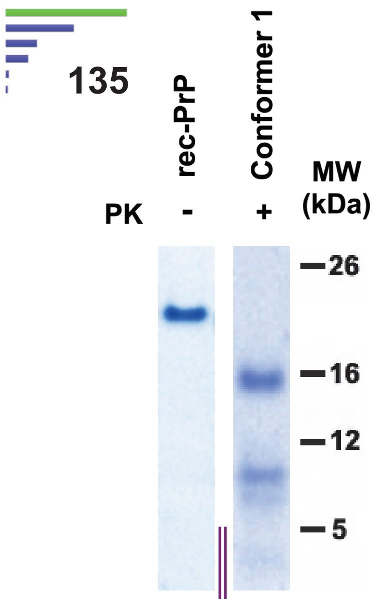

211 AA

1
Conformers

Tm^{Exp}: ND

ΔΔG: -1.39

In vitro studies

TgVole (1x) Not tested

Canyon mouse Not tested

In vivo studies

TgVole (1x) Not tested

Canyon mouse Not tested

pLDDT [86.18]

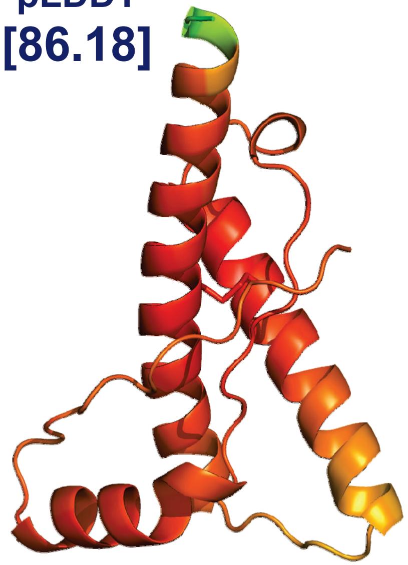

Protein code: 3975

White-footed mouse

GenBank: XM_028878611

Peromyscus leucopus

Rodentia 81/101
Cricetidae 25/27

P86%

82.1

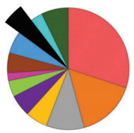

80-90

β2-α2 LOOP
NNQNNF
164

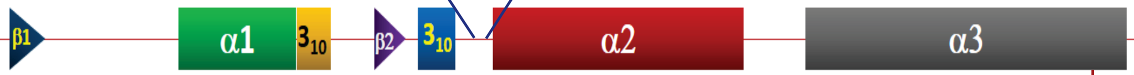

WNKPSKPKTNMKHVAGAAAAGAVVGGLGGYMLGSAMSRPMIHFGNDWEDRYYRENMYRYPNQVYYRPVDQYNNQNNFVHDCVNITIKQHTVTTTTKGENFTETDVKMMERVVEQMCVTQYQKESQAYYDGRRS-

I N E S

PrP sequence differs by 2 amino acids from the bank vole PrP (see in red)

No deletions

No insertions

No polymorphic variants found

6 species sharing the same primary sequence

Long-haired grass mouse
Abrothrix hirta

Long-haired akodont
Abrothrix longipilis hirtus

Southern grasshopper mouse
Onychomys torridus

Aztec mouse
Peromyscus aztecus

Plateau mouse
Peromyscus melanophrys

Mexican deer mouse
Peromyscus mexicanus

76

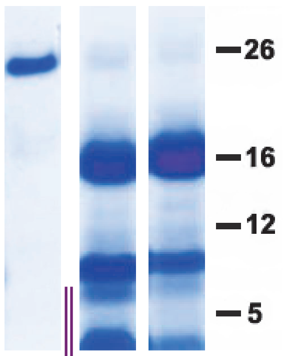

211 AA

2
Conformers

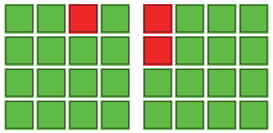

Tm[Exp]: ND

ΔΔG: -1.47

In vitro studies

TgVole (1x) — Not tested

White-footed mouse — Not tested

In vivo studies

TgVole (1x) — Not tested

White-footed mouse — Not tested

pLDDT
[86.22]

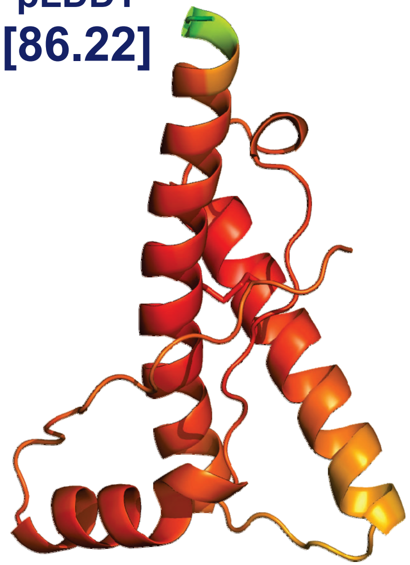

Protein code: 1011

Oldfield mouse

GenBank: EF467170

Peromyscus polionotus subgriseus

Rodentia 81/101
Cricetidae 25/27

P62%

32.5

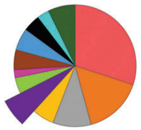

30-40

β2-α2 LOOP
NNQNNF
164

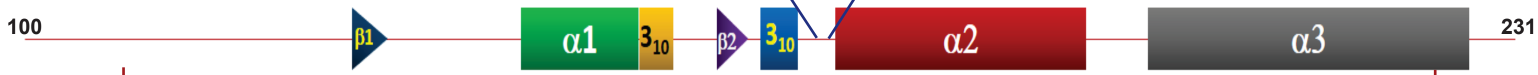

WNKPSKPKTNMKHVAGAAAAGAVVGGLGGYMLGSAMSRPMIHFGNDWEDRYYRENMNRYPNQVYYRPVDQYNNQNNFVHDCVNITIKQHTVTTTTKGENFTETDVKMMERVVEQMCVTQYQKESQAYYDGRRS-
I
E S

PrP sequence differs by 1 amino acids from the bank vole PrP (see in red)

No deletions
No insertions
No polymorphic variants found

2 species sharing the same primary sequence

Deer mouse
Peromyscus maniculatus bairdii

Roborovski hamster
Phodopus roborovskii

76

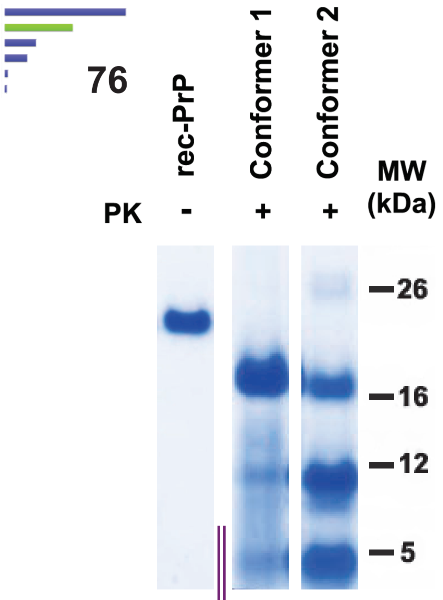

211 AA

2
Conformers

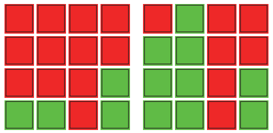

Tm^{Exp}: ND

ΔΔG: 1.23

In vitro studies

TgVole (1x) Not tested

Oldfield mouse Not tested

In vivo studies

TgVole (1x) Not tested

Oldfield mouse Not tested

pLDDT
[86.01]

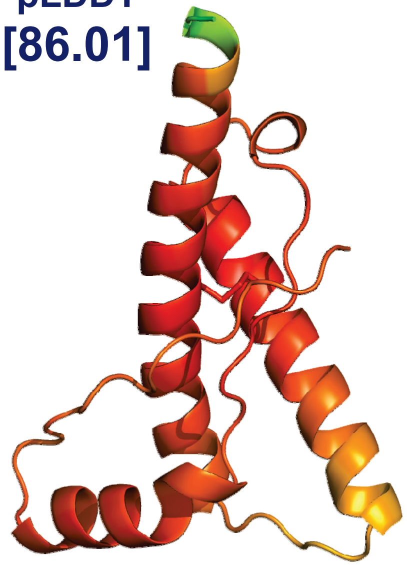

Protein code: 0941

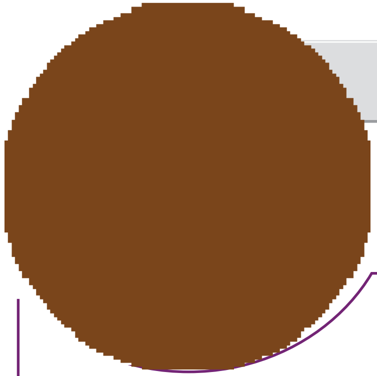

Red and white giant flying squirrel

GenBank: KM357835

Petaurista alborufus

Rodentia 81/101
Sciuridae 7/13

P100%

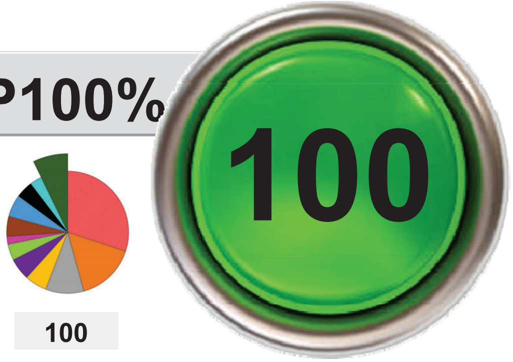

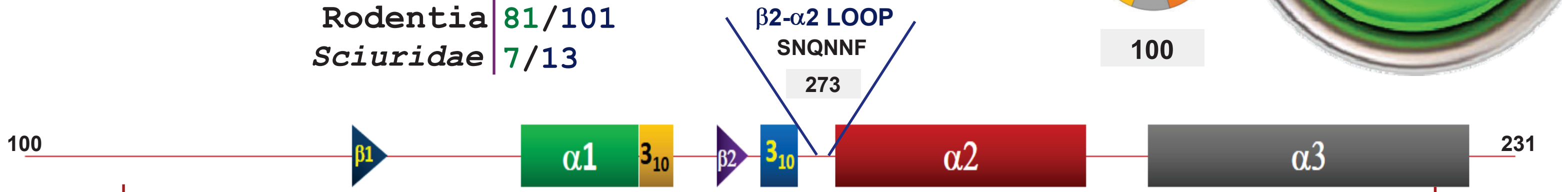

WGKPNKPKTNMKHVAGAAAAGAVVGGLGGYMLGSAMSRPLIHFGNDYEDRYYRENMYRYPNQVYYKPVDQYSNQNNFVHDCVNITVKQHTVTTTTKGENFTETDIKIMERVVEQMCITQYQQESQAYYQRGSS-

N S I M W N R N I V M V K EGR

PrP sequence differs by 11 amino acids from the bank vole PrP (see in red)

No deletions
No insertions
No polymorphic variants found

Distinct primary sequence across species

135

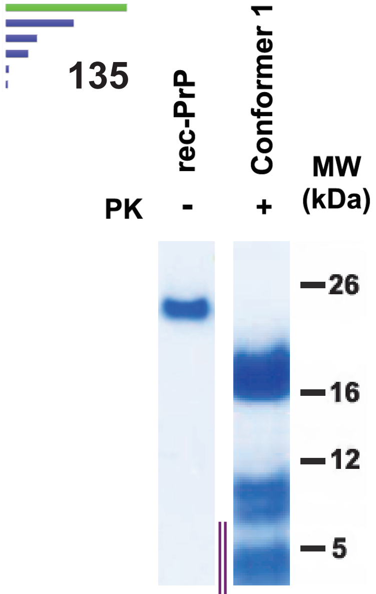

213 AA

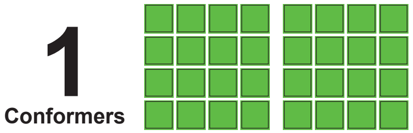

Tm^{Exp}: ND

ΔΔG: -12.03

In vitro studies

TgVole (1x) Not tested

Red and white giant flying Not tested

In vivo studies

TgVole (1x) Not tested

Red and white giant flying Not tested

pLDDT
[87.85]

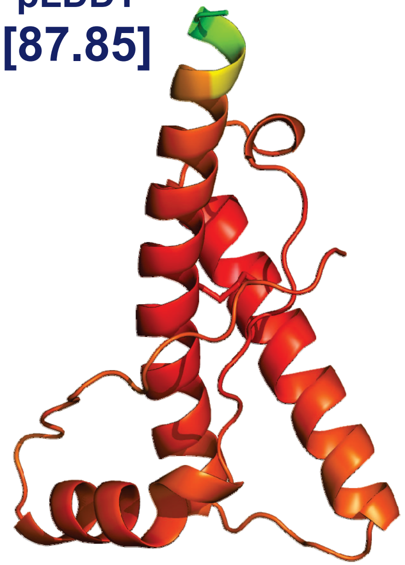

Protein code: 1454

Dassie rat

GenBank: BK063990

P71% **48.2**

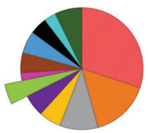

40-50

Petromus typicus

Rodentia 81/101
Petromuridae 1/1

β2-α2 LOOP
SNQNNF
273

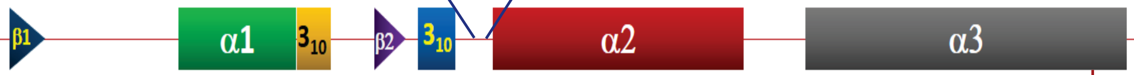

WNKPSKPKTNMKHVAGAAAAGAVVGGLGGYMLGSAMSRPLIHFGSDYEDRYYRENMYRYPNQVYYKPVDQYSNQNNFVQDCVNITIKQHTVTTTTKGENFTETDVKIMERVLEQMCTTQYQKESQAYYYRGRAG-
I M N W N R N H M V V EGRSS

PrP sequence differs by 11 amino acids from the bank vole PrP (see in red)

No deletions
No insertions
No polymorphic variants found

Distinct primary sequence across species

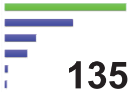

135

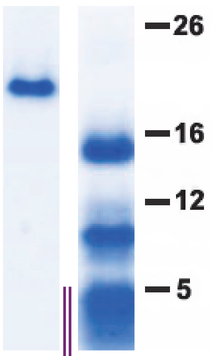

202 AA

1
Conformers

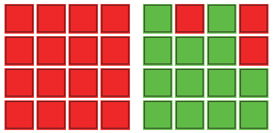

Tm^{Exp}: ND

ΔΔG: ND

In vitro studies

TgVole (1x) Not tested

Dassie rat Not tested

In vivo studies

TgVole (1x) Not tested

Dassie rat Not tested

pLDDT
[84.95]

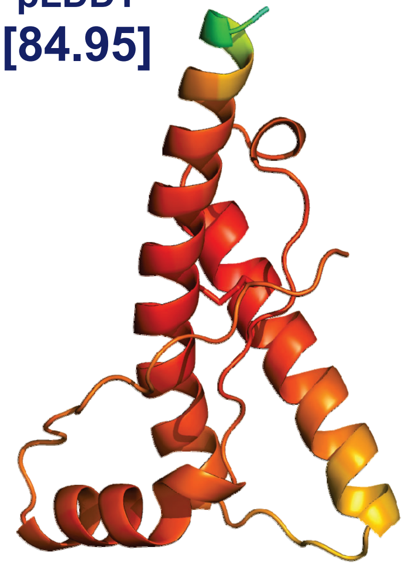

Protein code: 3148

Campbell's dwarf hamster

GenBank: EU886367

Phodopus campbelli

Rodentia 81/101
Cricetidae 25/27

Distinct primary sequence across species

P90%

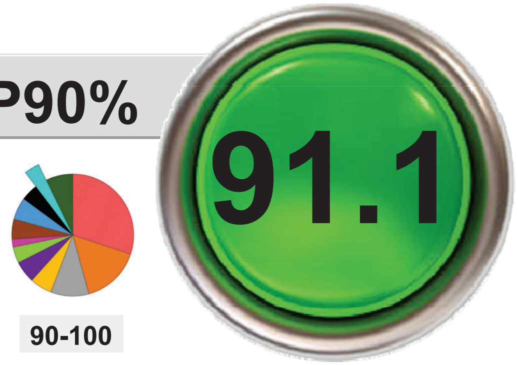

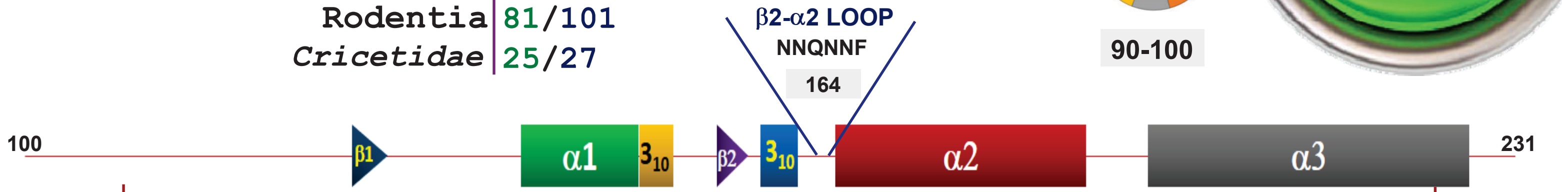

WNKPSKPKTNMKHVAGAAAAGAVVGGLGGYMLGSAMSRPMIHFGNDWEDRYYRENMNRYPNQVYYRPVDQYNNQNNFVHDCVNITIKQHTVTTTTKGENFTETDVKMMERVVEQMCVTQYQRESQAYYDGRRS-

I K E S

PrP sequence differs by 2 amino acids from the bank vole PrP (see in red)

No deletions
No insertions
No polymorphic variants found

135

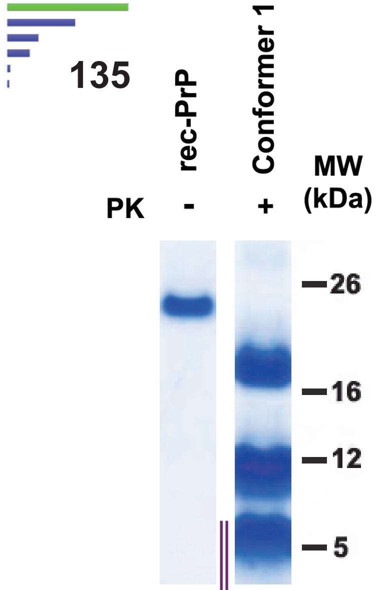

211 AA

1
Conformers

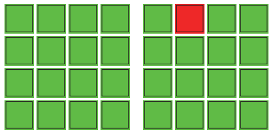

Tm^{Exp}: ND

ΔΔG: -0.53

pLDDT
[86.06]

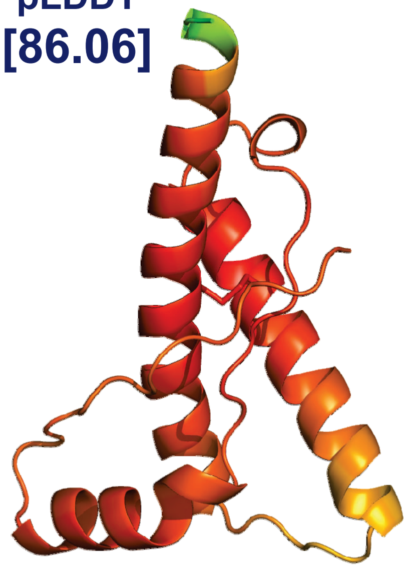

In vitro studies

TgVole (1x) Not tested

Campbell's dwarf hamster Not tested

In vivo studies

TgVole (1x) Not tested

Campbell's dwarf hamster Not tested

Protein code: 1433

Russian hamster

GenBank: EU886368

Phodopus sungorus

Rodentia 81/101
Cricetidae 25/27

P100%

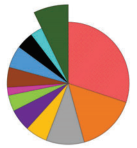

100

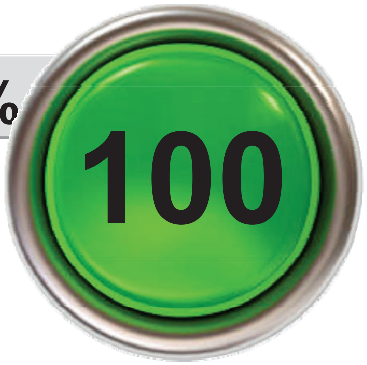

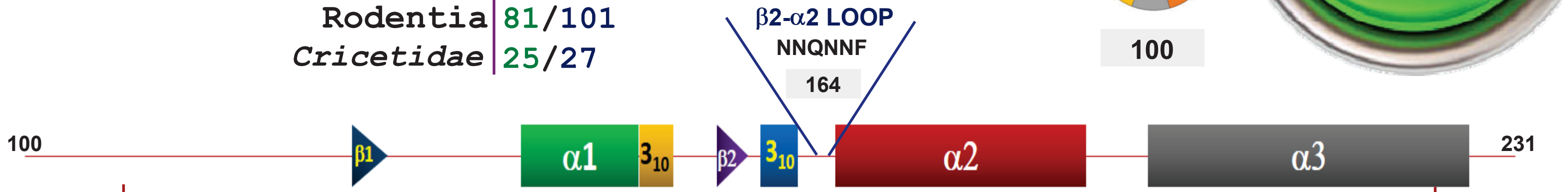

WNKPSKPKTNMKHVAGAAAAGAVVGGLGGYMLGSAMSRPMIHFGNDWEDRYYRENMNRYPNQVYYRPVDQYNNQNNFVHDCVNITIKQHTVTTTTKGENFTETDVKMMERVVEQMCITQYQRESQAYYDGRRS-
I V K E S

PrP sequence differs by 3 amino acids from the bank vole PrP (see in red)

No deletions
No insertions
No polymorphic variants found

Distinct primary sequence across species

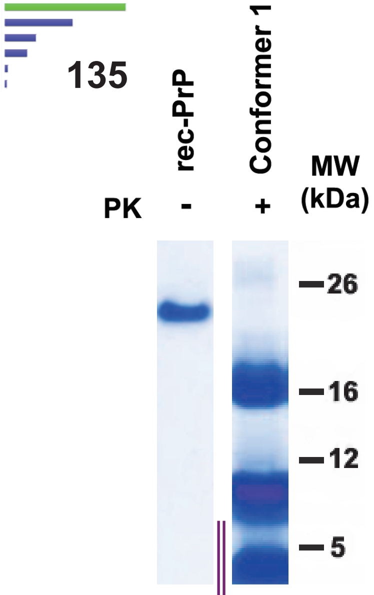

211 AA

1
Conformers

Tm^{Exp}: ND

ΔΔG: -1.18

In vitro studies

TgVole (1x) — Propagate

Russian hamster — Propagate

In vivo studies

TgVole (1x) — Infectious

Russian hamster — Not tested

pLDDT
[86.4]

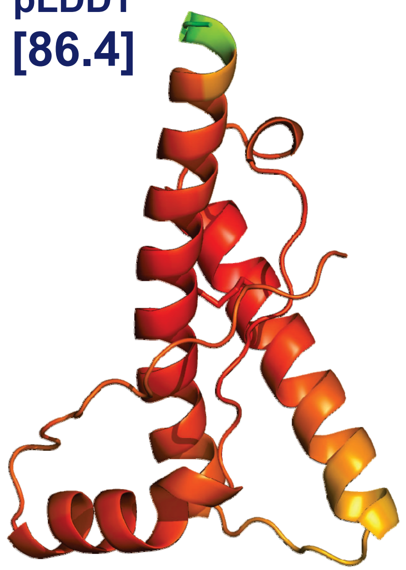

Protein code: 0822

Fat sand rat

GenBank: BK063966

Psammomys obesus

Rodentia 81/101
Muridae 16/18

P83%

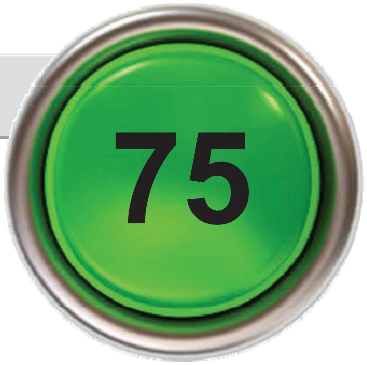

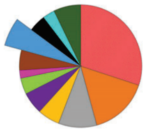

70-80

β2-α2 LOOP
SNQNNF
273

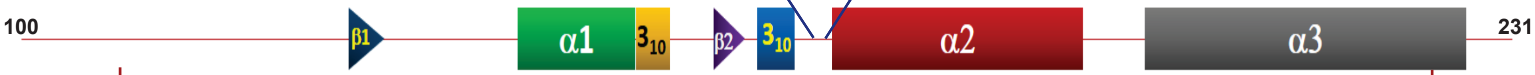

WNKPSKPKTNMKHVAGAAAAGAVVGGLGGYMLGSAMSRPMIHFGNDWEDRYYRENMYRYPNQVYYRPVDQYSNQNNFVHDCVNITIKQHTVTTTTKGENFTETDVKMMERVVEQMCVTQYQKESQAYYNGRRS-
I N N E S

PrP sequence differs by 3 amino acids from the bank vole PrP (see in red)

No deletions
No insertions
No polymorphic variants found

Distinct primary sequence across species

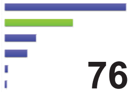

76

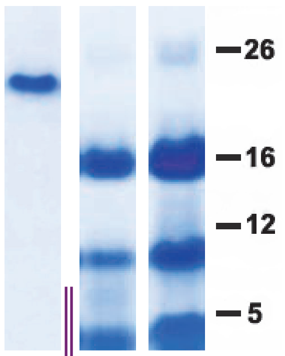

210 AA

2
Conformers

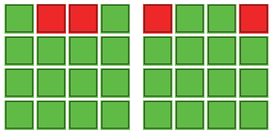

Tm^{Exp}: ND

ΔΔG: 1.23

In vitro studies

TgVole (1x) Not tested

Fat sand rat Not tested

In vivo studies

TgVole (1x) Not tested

Fat sand rat Not tested

pLDDT
[86.15]

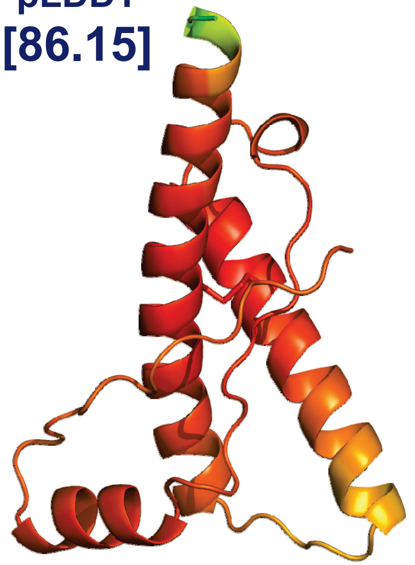

Protein code: 3096

Ricefield rat

GenBank: KF466955

Rattus argentiventer

Rodentia	81/101
Muridae	16/18

P50%

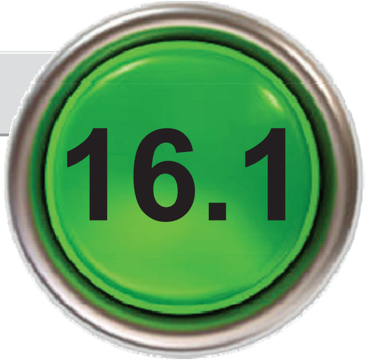

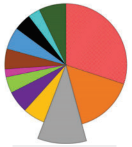

10-20

β2-α2 LOOP
SNQNNF
273

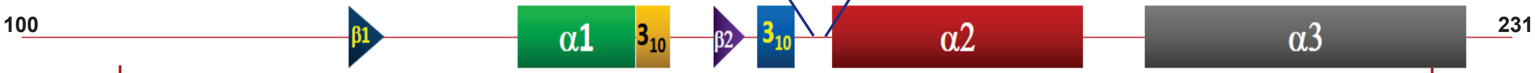

WNKPSKPKTNLKHVAGAAAAGAVVGGLGGYMLGSAMSRPMLHFGNDWEYRYYRENMYRYPNQVYYRPVDQYSNQNNFVHDCVNITIKQHTVTTTTKGENFTETDVKMMERVVEQMCVTQYQKESQAYYDGRRS-

I I D N N E S

PrP sequence differs by 5 amino acids from the bank vole PrP (see in red)

No deletions

No insertions

No polymorphic variants found

Distinct primary sequence across species

135

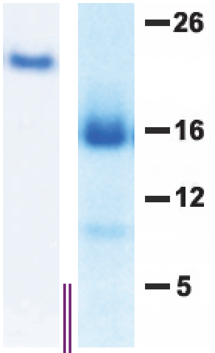

211 AA

1
Conformers

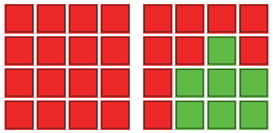

Tm[Exp]: ND

ΔΔG: -12.39

In vitro studies

TgVole (1x) — Not tested

Ricefield rat — Not tested

In vivo studies

TgVole (1x) — Not tested

Ricefield rat — Not tested

pLDDT
[87.27]

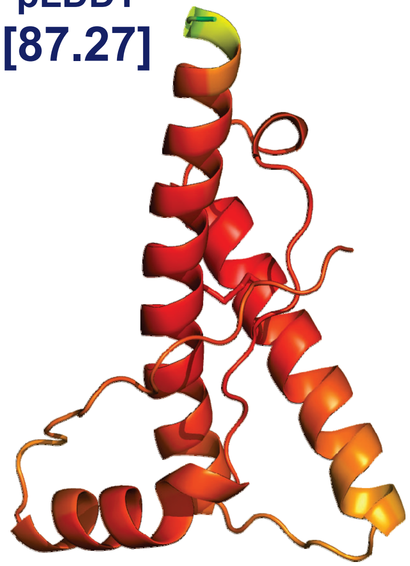

Protein code: 1341

Brown rat

GenBank: BK063913

Rattus norvegicus

Rodentia	81/101
Muridae	16/18

P100%

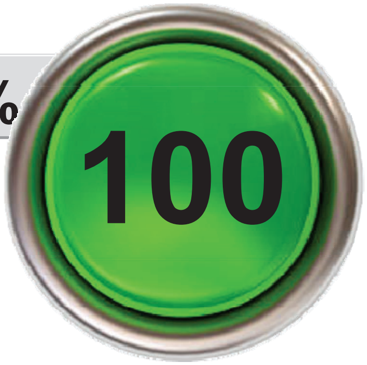

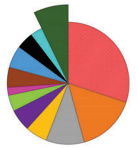

100

β2-α2 LOOP
SNQNNF
273

100 — β1 — α1 — 3_{10} — β2 — 3_{10} — α2 — α3 — 231

WNKPSKPKTNLKHVAGAAAAGAVVGGLGGYMLGSAMSRPMLHFGNDWEDRYYRENMYRYPNQVYYRPVDQYSNQNNFVHDCVNITIKQHTVTTTTKGENFTETDVKMMERVVEQMCVTQYQKESQAYYDGRRS-

I I N N E S

PrP sequence differs by 4 amino acids from the bank vole PrP (see in red)

No deletions

No insertions

No polymorphic variants found

4 species sharing the same primary sequence

Lesser ricefield rat
Rattus losea

Himalayan field rat
Rattus nitidus

Rat
Rattus rattus

Tanezumi rat
Rattus tanezumi

135

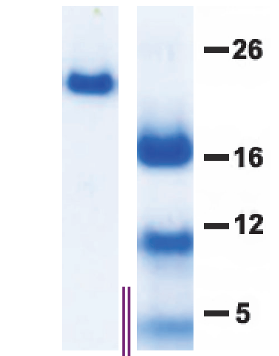

211 AA

1 Conformers

Tm^{Exp}: 68.1 ºC

ΔΔG: -6.76

***In vitro* studies**

TgVole (1x)	Propagate
Rat	Propagate

***In vivo* studies**

TgVole (1x)	Infectious
Rat	Not tested

pLDDT [86]

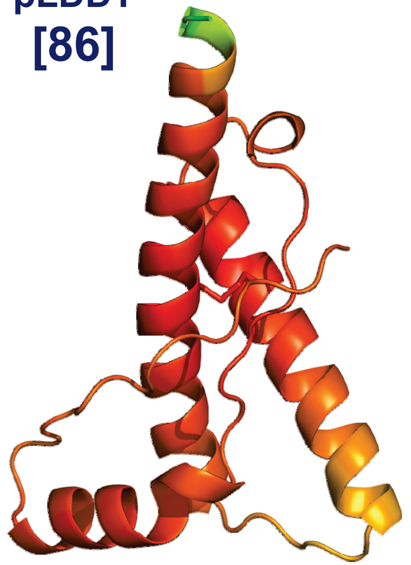

Mesic four-striped grass rat

GenBank: BK064164

Rhabdomys dilectus

Rodentia	81/101
Muridae	16/18

P67%

40.4

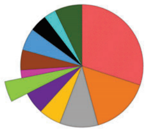

40-50

1 species sharing the same primary sequence

Four-striped grass mouse
Rhabdomys pumilio

β2-α2 LOOP
SNQNSF
36

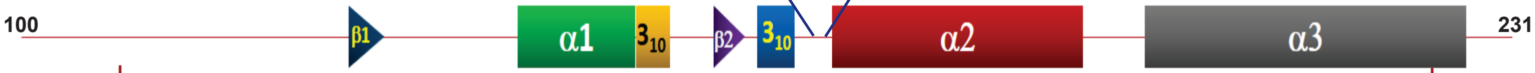

WNKPSKPKTNLKHVAGAAAAGAVVGGLGGYMLGSAMSRPMIHFGNDWEDRYYRENMYRYPNQVYYRPVDQYSNQNSFVHDCVNITIKQHTVTTTTKGENFTETDVKMMERVVEQMCTTQYQKESQAYYDGRRS-

I N N N V E S

PrP sequence differs by 5 amino acids from the bank vole PrP (see in red)

No deletions
No insertions
No polymorphic variants found

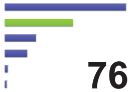

76

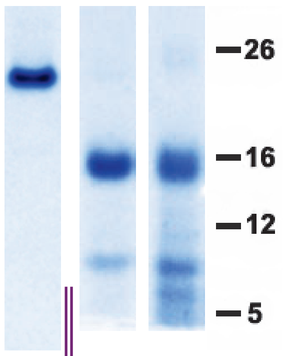

211 AA

2
Conformers

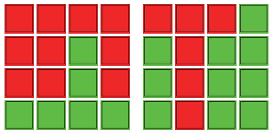

Tm^{Exp}: ND

ΔΔG: -4.89

In vitro studies

TgVole (1x) Not tested

Mesic four-striped grass rat Not tested

In vivo studies

TgVole (1x) Not tested

Mesic four-striped grass rat Not tested

pLDDT [86.05]

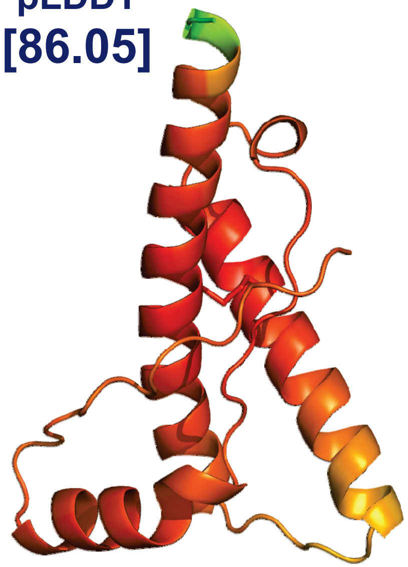

Protein code: 3800

Hoary bamboo rat

GenBank: BK063999

P89%

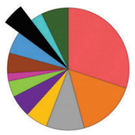

80-90

87.5

Rhizomys pruinosus

Rodentia 81/101

Spalacidae 3/3

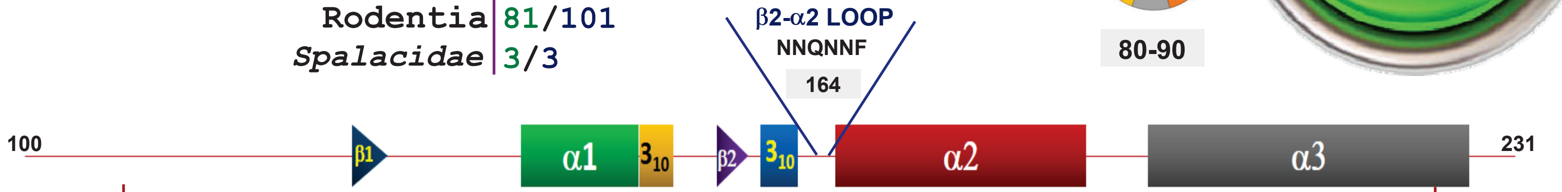

WNKPSKPKTNMKHVAGAAAAGAVVGGLGGYMLGSAMSRPLIHFPSDYENRYYQENMYRYPNQVYYRPVDQYNNQNNFVHDCVNITVKQHTITTTTKGENFTETDIKMMERVVEQMCITQYQKESQAYYDGRRS-

I M GN WD R N I V V V E S

PrP sequence differs by 12 amino acids from the bank vole PrP (see in red)

No deletions

No insertions

No polymorphic variants found

Distinct primary sequence across species

76

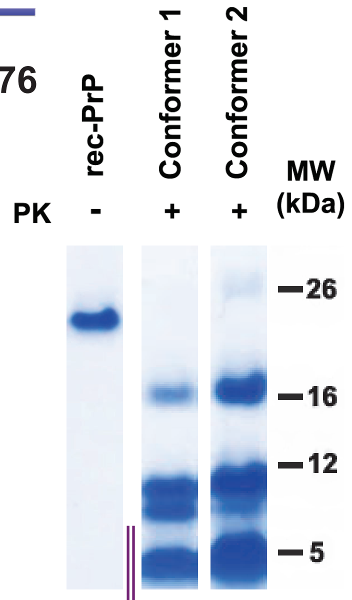

212 AA

2

Conformers

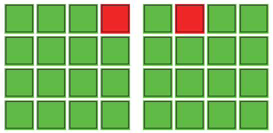

Tm^{Exp}: ND

ΔΔG: -9.79

pLDDT [86.2]

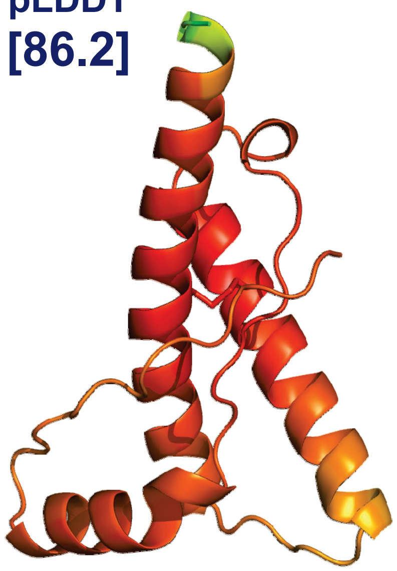

In vitro studies

TgVole (1x) — Not tested

Hoary bamboo rat — Not tested

In vivo studies

TgVole (1x) — Not tested

Hoary bamboo rat — Not tested

Protein code: 3159

Mount Data shrew-rat

GenBank: BK064163

Rhynchomys soricoides

Rodentia 81/101
Muridae 16/18

P77%

67.5

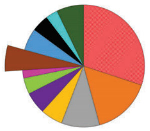

60-70

β2-α2 LOOP
SNQNNF
273

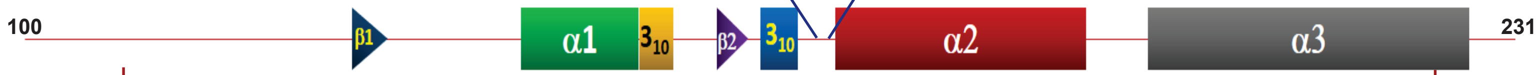

WNKPSKPKTNLKQMAGAAAAGAVVGGLGGYMLGSAMSRPMIHFGNDWEDRYYRENMYRYPNQVYYRPVDQYSNQNNFVHDCVNITIKQHTVTTTTKGENFTETDVKMMERVVEQMCVTQYQKESQAYYEGRRS-
I HV N N S

PrP sequence differs by 5 amino acids from the bank vole PrP (see in red)

No deletions
No insertions
No polymorphic variants found

Distinct primary sequence across species

135

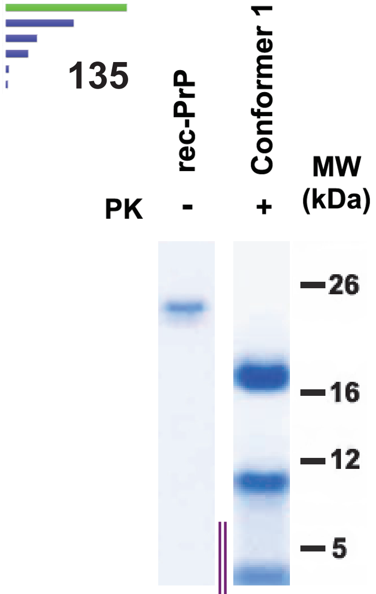

211 AA

1
Conformers

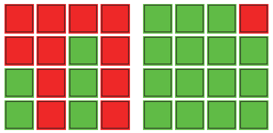

Tm^{Exp}: ND

ΔΔG: -6.81

In vitro studies

TgVole (1x) Not tested

Mount Data shrew-rat Not tested

In vivo studies

TgVole (1x) Not tested

Mount Data shrew-rat Not tested

pLDDT
[86.02]

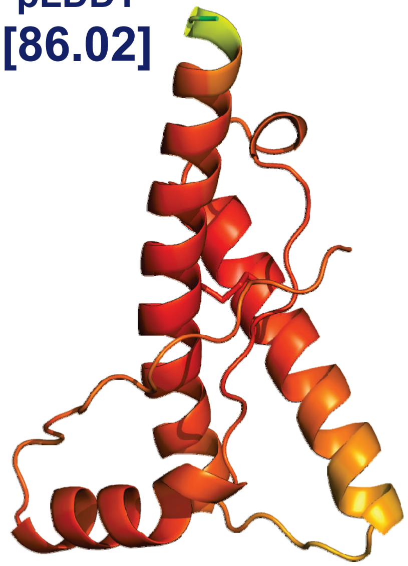

Protein code: 3799

Paulina’s limestone rat

GenBank: KF466958

P100%

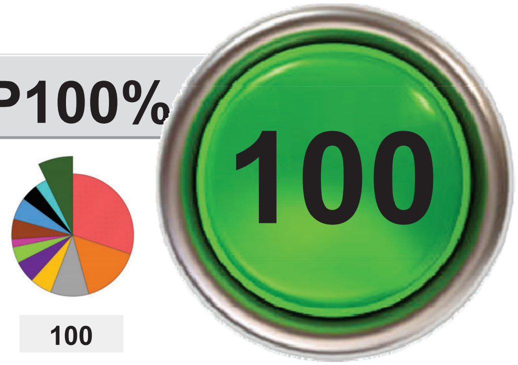

Saxatilomys paulinae

Rodentia	81/101
Muridae	16/18

β2-α2 LOOP
SNQNNF
273

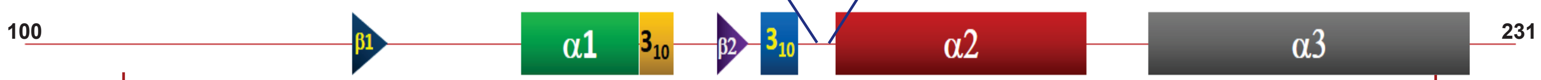

WNKPSKPKTNLKHVAGAAAAGAVVGGLGGYMLGSAMSRPMLHFGNDWEDRYYRENMYRYPNQVYYRPVDQYSNQNNFVHDCVNITIKQHTVTTTTKGENFTETDVKMMERVVEQMCVTQYQKESQAYYDGRRS-

I I N N E S

PrP sequence differs by 4 amino acids from the bank vole PrP (see in red)

No deletions

No insertions

No polymorphic variants found

Distinct primary sequence across species

135

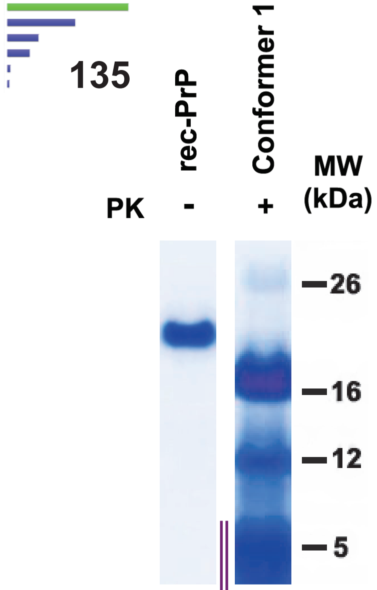

201 AA

1

Conformers

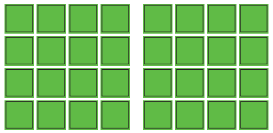

Tm^{Exp}: ND

ΔΔG: -10.57

In vitro studies

TgVole (1x) Not tested

Paulina’s limestone rat Not tested

In vivo studies

TgVole (1x) Not tested

Paulina’s limestone rat Not tested

pLDDT [86.57]

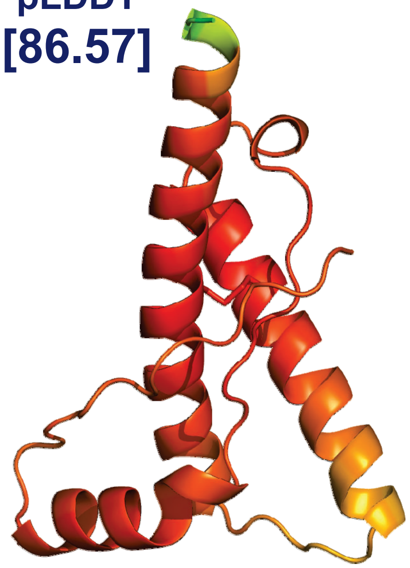

Protein code: 1449

Eastern gray squirrel

GenBank: BK064159

Sciurus carolinensis

Rodentia 81/101
Sciuridae 7/13

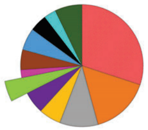

P69% **44.6**

40-50

Fox squirrel
Sciurus niger

1 species sharing the same primary sequence

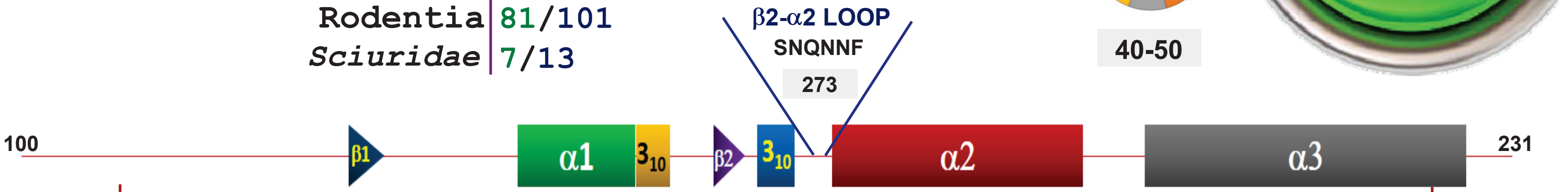


```
WGKPSKPKTNMKHVAGAAAAGAVVGGLGGYMLGSAMSRPLIHFGNDYEDRYYRENMYRYPNQVYYKPVDQYSNQNNFVHDCVNITVKQHTVTTTTKGENFTETDIKIMERVVEQMCITQYQQEAQAYYQRGSS-
 N        I                            M      W          N         R     N             I                        V M           V    K S   EGR
```

PrP sequence differs by 12 amino acids from the bank vole PrP (see in red)

No deletions
No insertions
No polymorphic variants found

Tm[Exp]: ND
ΔΔG: -14.31

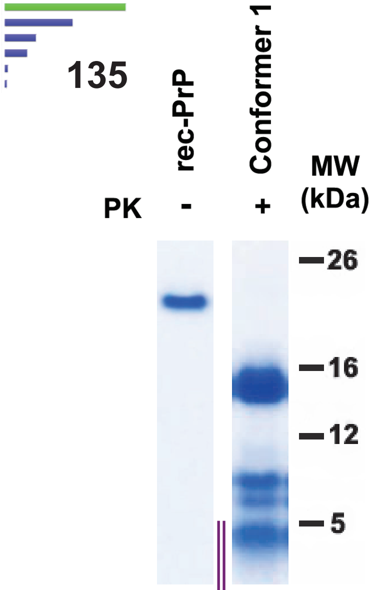

213 AA

1 Conformers

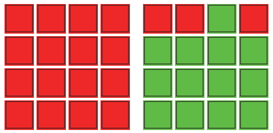

In vitro studies

TgVole (1x) — Not tested

Eastern gray squirrel — Not tested

In vivo studies

TgVole (1x) — Not tested

Eastern gray squirrel — Not tested

pLDDT
[84.98]

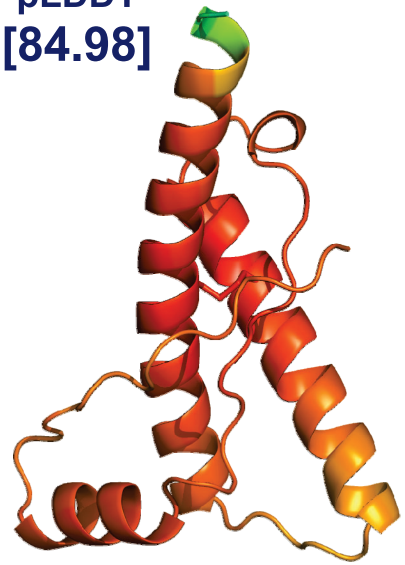

Protein code: 3708

Guayaquil squirrel

GenBank: KM357836

Sciurus stramineus

Rodentia 81/101
Sciuridae 7/13

P71%

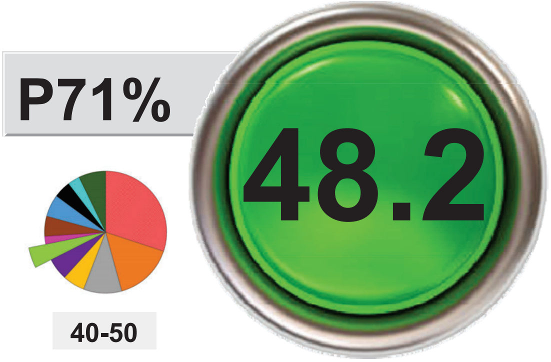

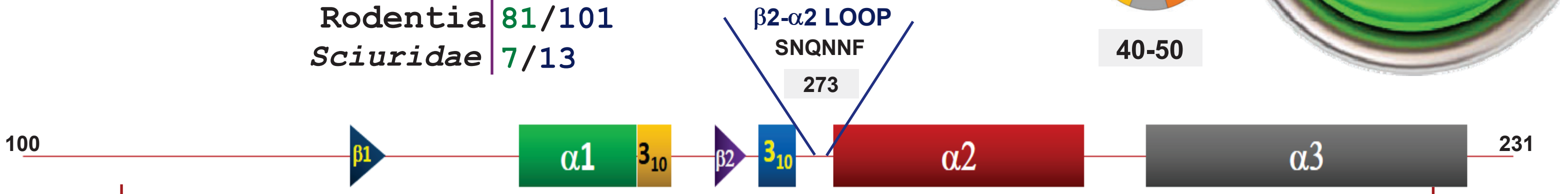

WSKPNKPKTNMKHVAGAAAAGAVVGGLGGYMLGSAMSRPLIHFGSDYEDRYYRENMNRYPNQVYYKPVDQYSNQNNFVHDCVNITIKQHTVTTTTKGENFTETDIKIMERVVEQMCITQYQQEAQAYYQRGAS-
N S I M N W R N V M V K S EGRS

PrP sequence differs by 11 amino acids from the bank vole PrP (see in red)

No deletions
No insertions
No polymorphic variants found

Distinct primary sequence across species

Tm[Exp]: ND
ΔΔG: -7.89

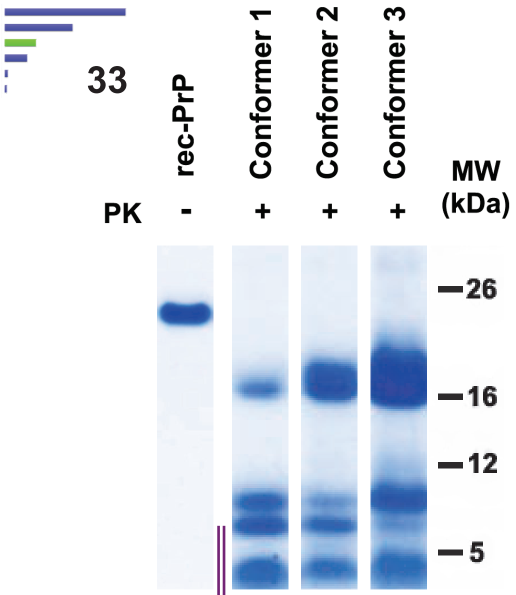

213 AA

3 Conformers

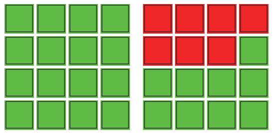

In vitro studies

TgVole (1x) Not tested

Guayaquil squirrel Not tested

In vivo studies

TgVole (1x) Not tested

Guayaquil squirrel Not tested

pLDDT [85.24]

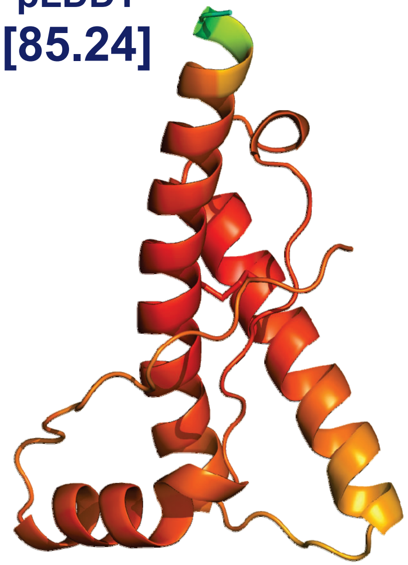

Protein code: 1091

Red squirrel

GenBank: AY133037

Sciurus vulgaris

Rodentia 81/101
Sciuridae 7/13

2 species sharing the same primary sequence

Japanese squirrel *Sciurus lis*

Cape ground squirrel *Xerus inauris*

P53%

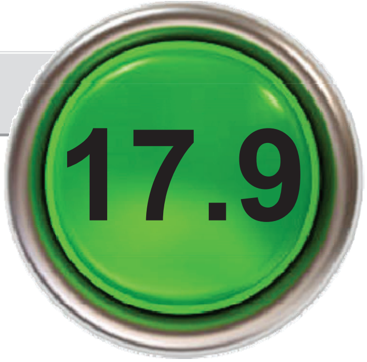

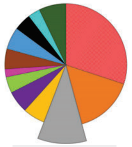

10-20

β2-α2 LOOP
SNQNNF
273

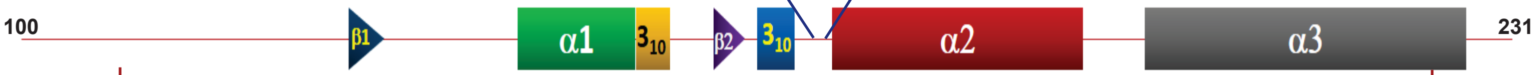

WGKPNKPKTNMKHVAGAAAAGAVVGGLGGYMLGSAMSRPLIHFGNDYEDRYYRENMYRYPNQVYYKPVDQYSNQNNFVHDCVNITVKQHTVTTTTKGENFTETDIKIMERVVEQMCITQYQQEAQAYYQRGSS-

N S I M W N R N I V M V K S EGR

PrP sequence differs by 12 amino acids from the bank vole PrP (see in red)

No deletions
No insertions
No polymorphic variants found

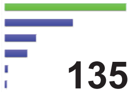

135

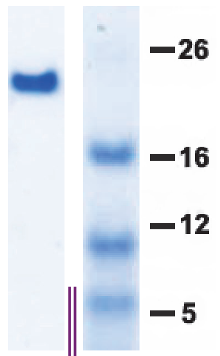

213 AA

1 Conformers

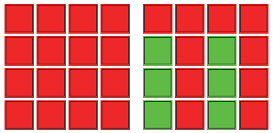

Tm^{Exp}: ND

ΔΔG: -15.13

In vitro studies

TgVole (1x) Not tested

Red squirrel Not tested

In vivo studies

TgVole (1x) Not tested

Red squirrel Not tested

pLDDT [87.73]

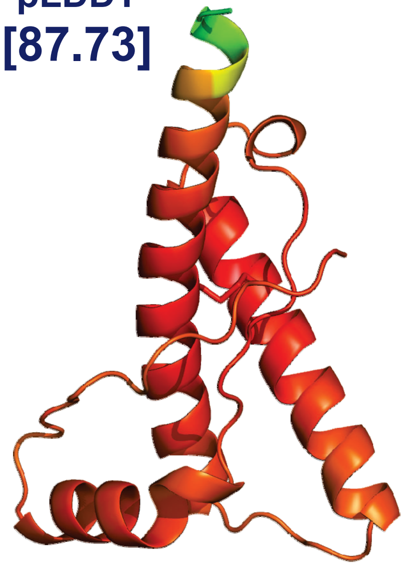

Protein code: 0949

Tawny-bellied cotton rat

GenBank: AF117324

Sigmodon fulviventer

Rodentia 81/101

Cricetidae 25/27

P83%

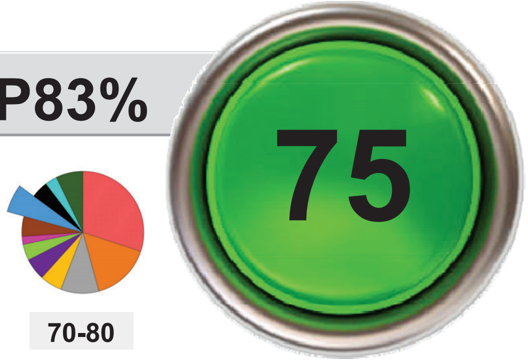

1 species sharing the same primary sequence

Hispid cotton rat *Sigmodon hispidus*

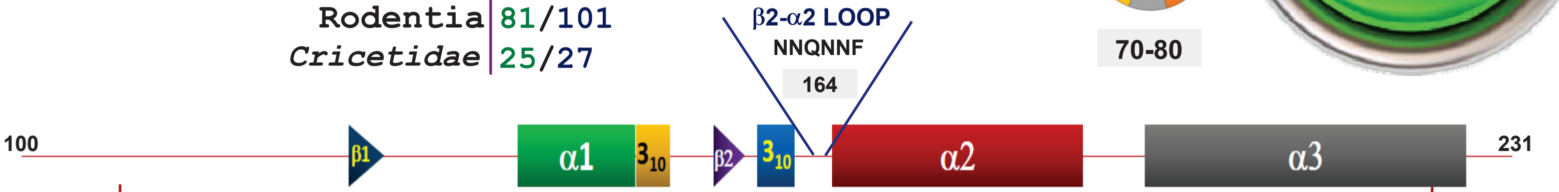

WNKPSKPKTNMKHVAGAAAAGAVVGGLGGYMLGSAMSRPMIHFGNDWEDRYYRENMYRYPNQVYYRPVDQYNNQNNFVHDCVNITIKQHTVTTTTKGENFTETDVKMMERVVEQMCVTQYQKESQAYYDGRRS-

I N E S

PrP sequence differs by 2 amino acids from the bank vole PrP (see in red)

No deletions

No insertions

No polymorphic variants found

135

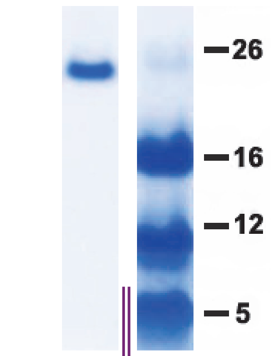

211 AA

1 Conformers

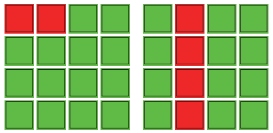

Tm^{Exp}: ND

ΔΔG: -0.82

In vitro studies

TgVole (1x) Not tested

Tawny-bellied cotton rat Not tested

In vivo studies

TgVole (1x) Not tested

Tawny-bellied cotton rat Not tested

pLDDT [85.88]

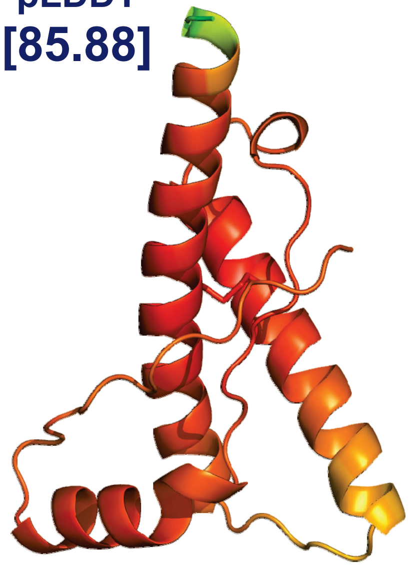

Protein code: 1349

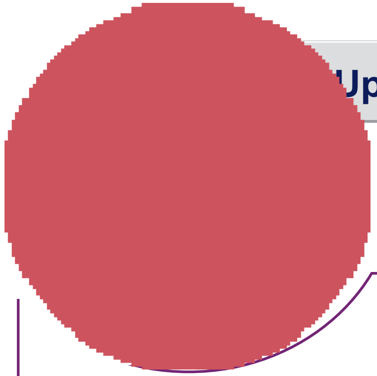

Upper Galilee Mountains blind mole-rat

GenBank: XM_008835893

Spalax galili

Rodentia	81/101
Spalacidae	3/3

P84%

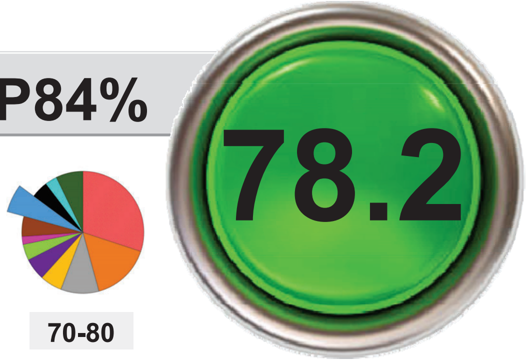

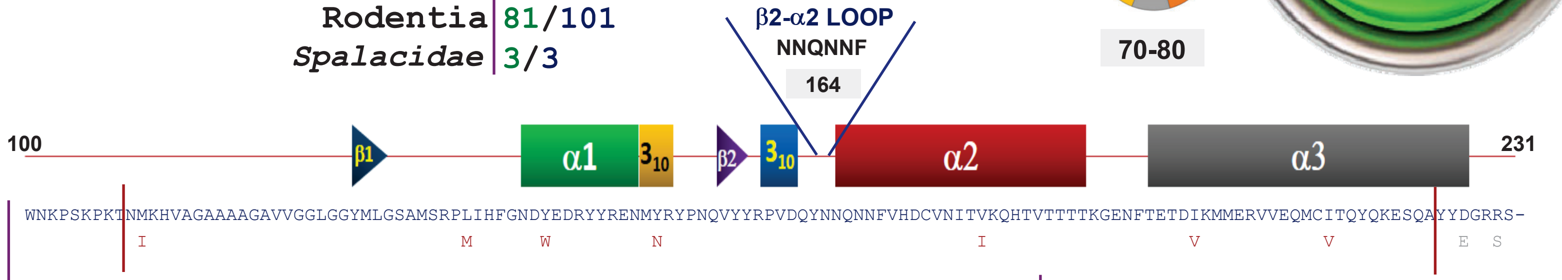

PrP sequence differs by 7 amino acids from the bank vole PrP (see in red)

No deletions

No insertions

No polymorphic variants found

Distinct primary sequence across species

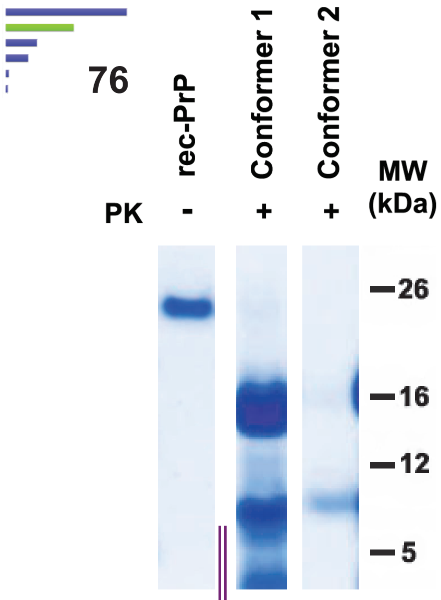

213 AA

2

Conformers

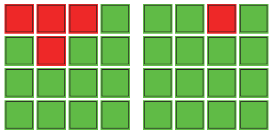

Tm[Exp]: ND

ΔΔG: -10.9

In vitro studies

Not tested

TgVole (1x)

Not tested

Upper Galilee Mountains blind

In vivo studies

Not tested

TgVole (1x)

Not tested

Upper Galilee Mountains blind

pLDDT [86.29]

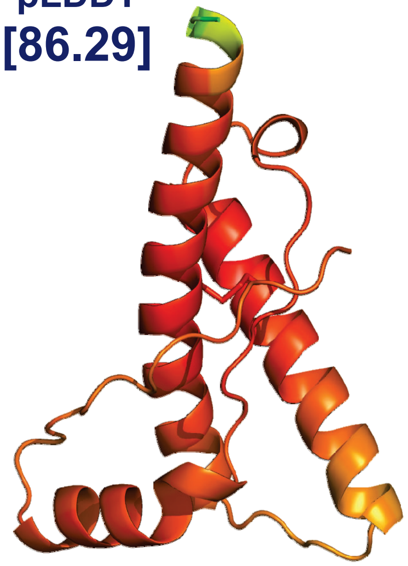

Protein code: 1088

California ground squirrel

GenBank: BK064192

Spermophilus beecheyi

Rodentia 81/101
Sciuridae 7/13

P31%

0.4

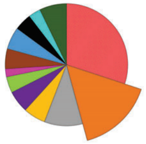

β2-α2 LOOP
PNQNSF
6

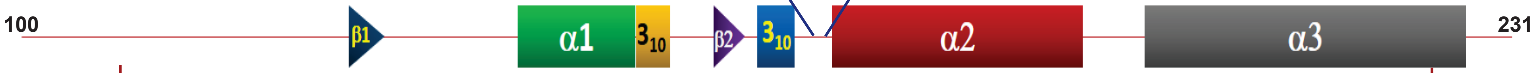

WGKPVKPKTSMKHVAGAAAAGAVVGGLGGYMLGSAMSRPMMHFGSDYEDRYYRENMNRYPNQVYYKPVDQYPNQNSFVHDCVNITIKQHTVTTTTKGDNFTETDIKIMERVVEQMCTTQYKQEAQAYYQRGSS-
N S NI I N W R N N E V M V QK S EGR

PrP sequence differs by 15 amino acids from the bank vole PrP (see in red)

No deletions
No insertions
No polymorphic variants found

Distinct primary sequence across species

135

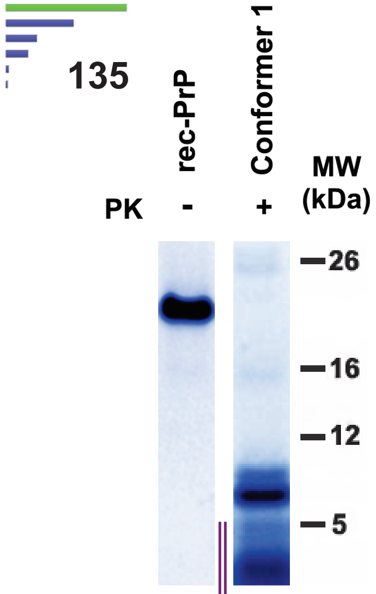

204 AA

1
Conformers

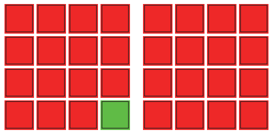

Tm^{Exp}: ND

ΔΔG: -11.31

In vitro studies

TgVole (1x) Not tested

California ground squirrel Not tested

In vivo studies

TgVole (1x) Not tested

California ground squirrel Not tested

pLDDT
[87.08]

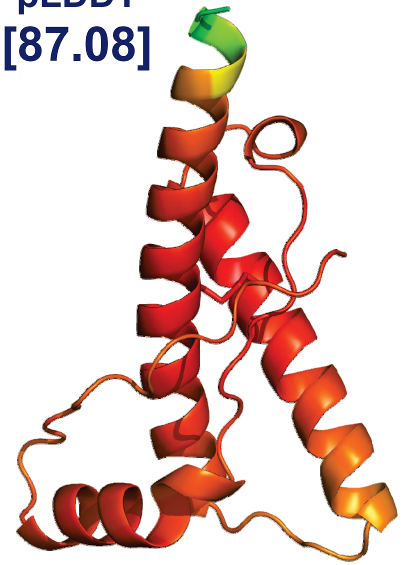

Protein code: 4486

Daurian ground squirrel

GenBank: BK063919

P29%

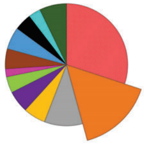

0-10

0.9

Spermophilus dauricus

Rodentia 81/101
Sciuridae 7/13

Distinct primary sequence across species

β2-α2 LOOP
PNQNSF
6

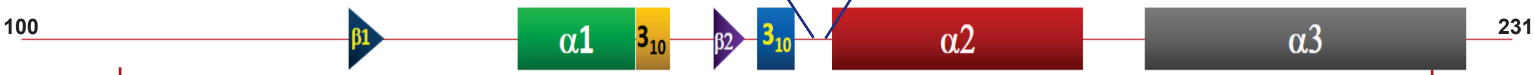

WGKPVKPKTSMKHMAGAAAAGAVVGGLGGYMLGSAMSRPMMHFGSDYEDRYYRENMNRYPSQVYYKPVDQYPNQNSFVHDCVNITIKQHTTTTTTKGDNFTETDIKIMERVVEQMCTTQYKQEAQQAYYPRGSS-
N S NI V I N W N R N N V E V M V QK S AY EG S

PrP sequence differs by 19 amino acids from the bank vole PrP (see in red)

No deletions
No insertions
No polymorphic variants found

Tm^{Exp}: ND
ΔΔG: ND

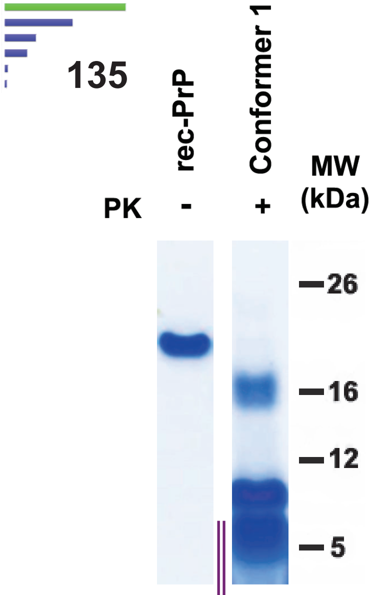

197 AA

1
Conformers

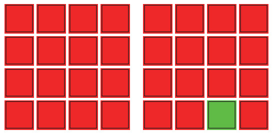

In vitro studies

Not tested
TgVole (1x)

Not tested
Daurian ground squirrel

In vivo studies

Not tested
TgVole (1x)

Not tested
Daurian ground squirrel

pLDDT
[85.23]

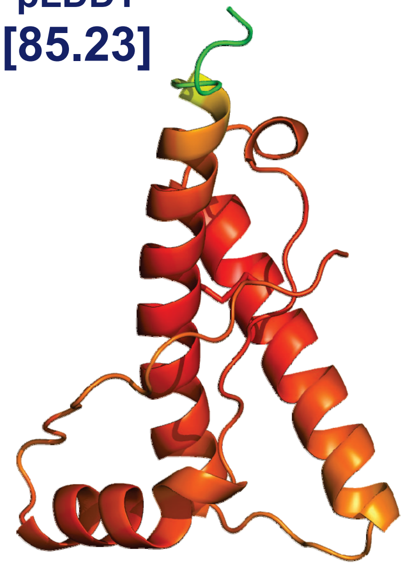

Protein code: 2025

Arctic ground squirrel

GenBank: XM_026385776

Spermophilus parryii

Rodentia	81/101
Sciuridae	7/13

P29%

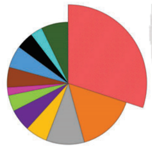

0

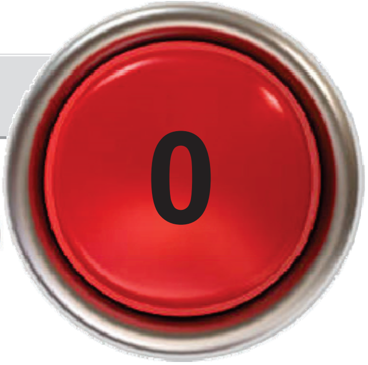

β2-α2 LOOP
PNQNSF
6

100

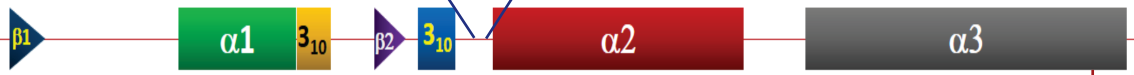

231

GGKPVKPKTSMKHMAGAAAAGAVVGGLGGYMLGSAMSRPMMHFGNDYEDRYYRENMNRYPSQVYYKPVDQYPNQNSFVHDCVNITIKQHTTTTTTKGDNFTETDIKIMERVVEQMCTTQYKQEAQQAYYPRGSS-

WN S NI V I W N R N N V E V M V QK S AY EG S

PrP sequence differs by 18 amino acids from the bank vole PrP (see in red)

No deletions

No insertions

No polymorphic variants found

Distinct primary sequence across species

Tm^{Exp}: ND

ΔΔG: ND

PK -

rec-PrP

MW (kDa)

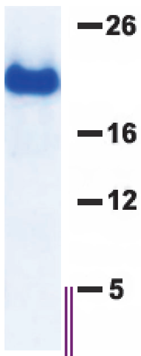

207 AA

NO MISFOLDING

0

Conformers

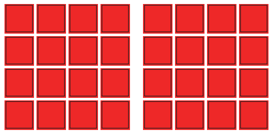

In vitro studies

NOT APPLICABLE

In vivo studies

NOT APPLICABLE

pLDDT [84.73]

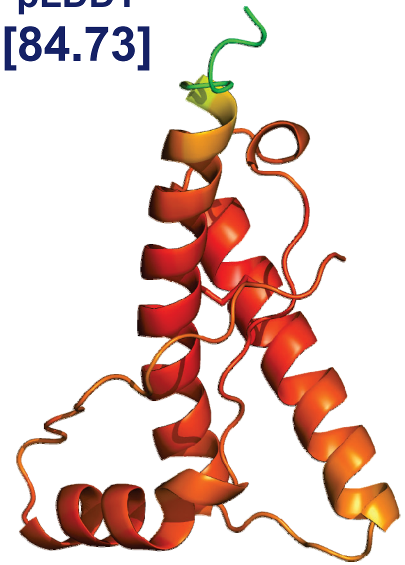

Protein code: 1119

Thirteen-lined ground squirrel

GenBank: XM_005320503

Spermophilus tridecemlineatus

Rodentia	81/101
Sciuridae	7/13

P29%

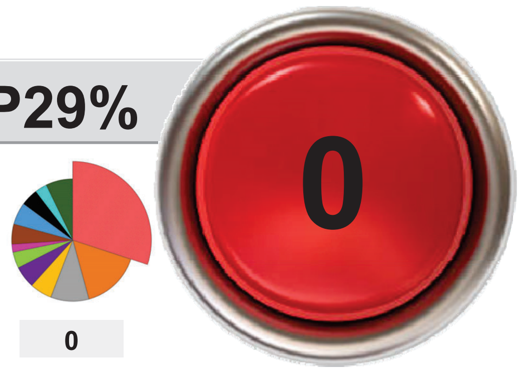

β2-α2 LOOP
PNQNSF
6

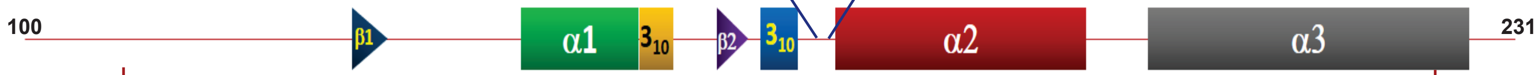


```
WGKPVKPKTSMKHVAGAAAAGAVVGGLGGYMLGSAMSRPMMHFGNDYEDRYYRENMNRYPSQVYYKPVDQYPNQNSFVHDCVNITIKQHTTTTTTKGDNFTETDIKIMERVVEQMCTTQYKQEAQQAYYPRGSS-
 N  S   NI                             I     W              N     R     N   N              V      E      V M          V   QK S AY EG S
```

PrP sequence differs by 17 amino acids from the bank vole PrP (see in red)

No deletions

No insertions

No polymorphic variants found

2 species sharing the same primary sequence

Gunnison's prairie dog
Cynomys gunnisoni

Black-tailed prairie dog
Cynomys ludovicianus

Tm^{Exp}: ND

ΔΔG: ND

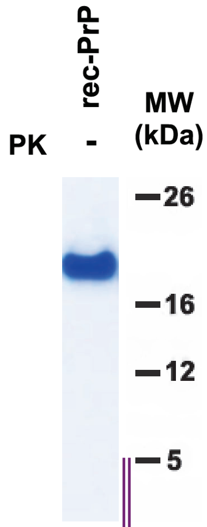

205 AA

NO MISFOLDING

0 Conformers

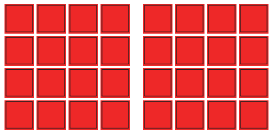

In vitro studies

NOT APPLICABLE

In vivo studies

NOT APPLICABLE

pLDDT [83.82]

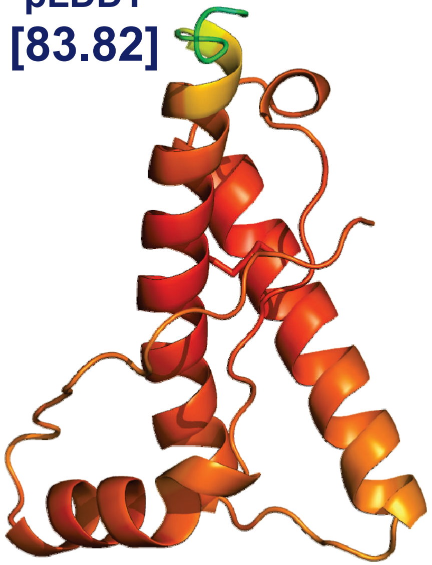

Protein code: 1106

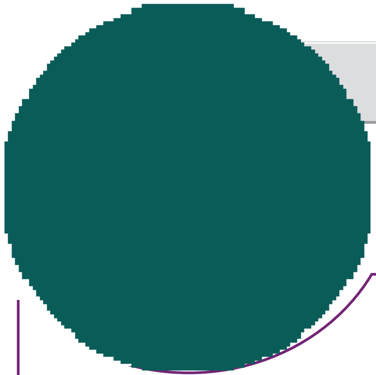

Siberian chipmunk

GenBank: BK064209

Tamias sibiricus

Rodentia	81/101
Sciuridae	7/13

P29%

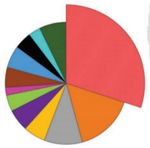

0

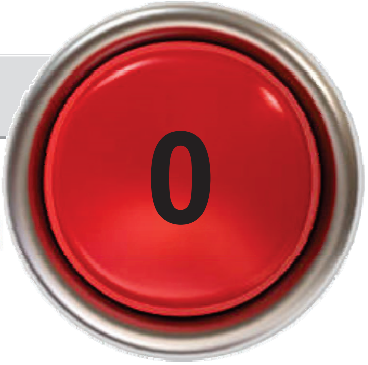

β2-α2 LOOP
ANQNSF
1

100 β1 α1 3_{10} β2 3_{10} α2 α3 231

WGKPNKPKTNMKHMAGAAAAGAVVGGLGGYMLGSAMSRPPMHFGNDYEDRYYRENMNRYPNQVYYKPVDQYANQNSFVHDCVNITVKQHTVTTTTKGENFTETDIKIMERVVEQMCTTKYQQEAQAYYQRGSS-

N S I V MI W R N N I V M V Q K S EGR

PrP sequence differs by 15 amino acids from the bank vole PrP (see in red)

No deletions

No insertions

No polymorphic variants found

Distinct primary sequence across species

Tm^{Exp}: ND

ΔΔG: -11.42

PK - rec-PrP

MW (kDa)

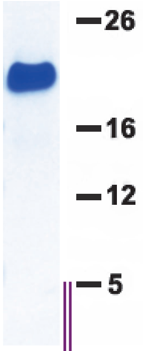

205 AA

NO MISFOLDING

0 Conformers

In vitro studies

NOT APPLICABLE

In vivo studies

NOT APPLICABLE

pLDDT [93.27]

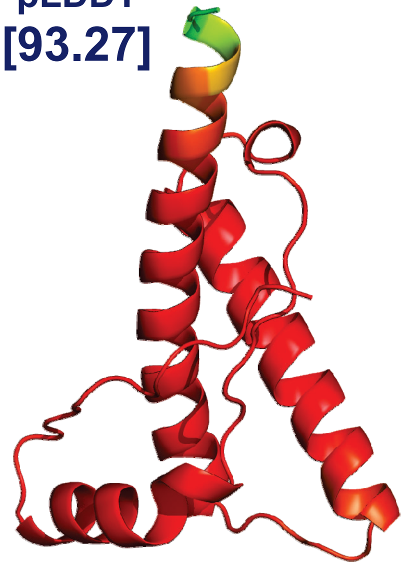

Protein code: 4618

Botta's pocket gopher

GenBank: BK064206

Thomomys bottae

Rodentia 81/101
Geomyidae 1/1

P81% **71.4**

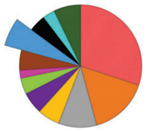

70-80

β2-α2 LOOP
NNQNNF
164

100 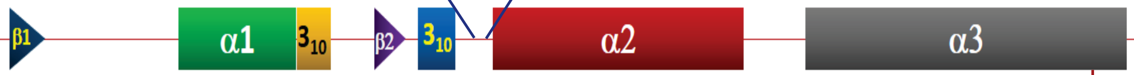 231

WNKPSKPKTSMKHVAGAAAAGAVVGGLGGYMLGSAMSRPLLHFGNDYEDRYYRENMNRYPSQVYYKPVDQYNNQNNFVHDCVNITVKQHTVTTTTKGENFTETDVKIMERMVEQMCITQYQKEAQAYYEPRS-
NI MI W N R I M V V S G S

PrP sequence differs by 12 amino acids from the bank vole PrP (see in red)

No deletions
No insertions
No polymorphic variants found

Distinct primary sequence across species

135

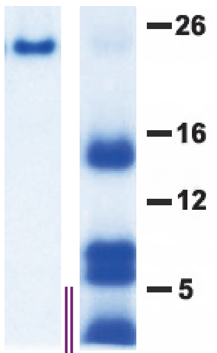

219 AA

1 Conformers

Tm^{Exp}: ND

ΔΔG: ND

In vitro studies

TgVole (1x) Not tested

Botta's pocket gopher Not tested

In vivo studies

TgVole (1x) Not tested

Botta's pocket gopher Not tested

pLDDT
[87.29]

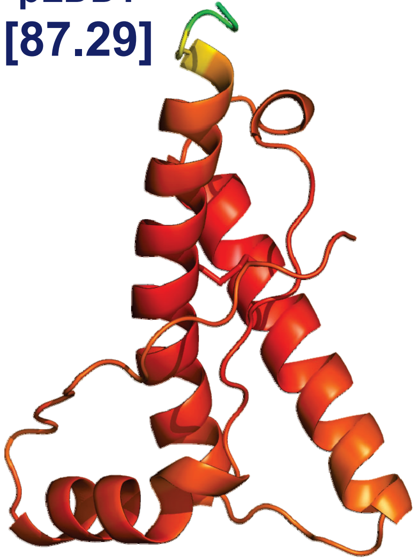

Protein code: 4580

Greater cane rat

GenBank: BK064162

Thryonomys swinderianus

Rodentia 81/101
Thryonomyidae 1/1

P79%

70.4

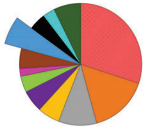

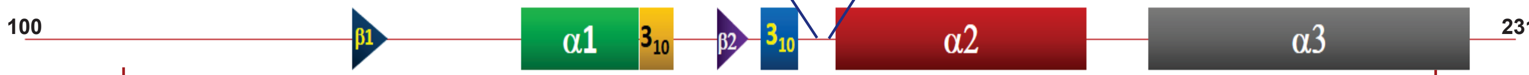

WNKPSKPKTNMKHVAGAAAAGAVVGGLGGYMLGSAMSRPLIHFGNDYEDRYYRENMNRYPNQVYYKPVDQYTNQNNFVQDCVNITIKQHTVTTTTKGENFTETDVKIMERVLEQMCTTQYQKESQAYYYQGSSG-

I M W R N H M V V EGR

PrP sequence differs by 9 amino acids from the bank vole PrP (see in red)

No deletions
No insertions
No polymorphic variants found

Distinct primary sequence across species

33

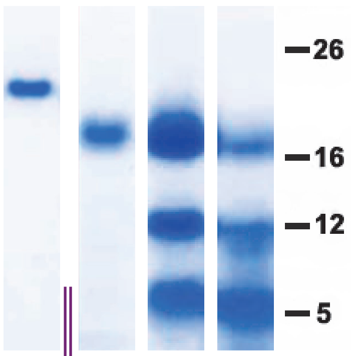

202 AA

3 Conformers

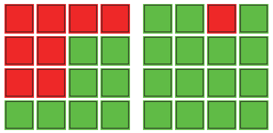

Tm^{Exp}: ND

ΔΔG: ND

In vitro studies

TgVole (1x) Not tested

Greater cane rat Not tested

In vivo studies

TgVole (1x) Not tested

Greater cane rat Not tested

pLDDT
[83.92]

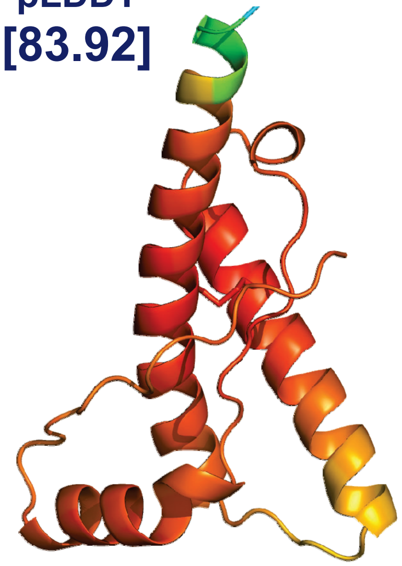

Protein code: 3776

Plains viscacha rat

GenBank: BK064138

Tympanoctomys barrerae

Rodentia 81/101
Octodontidae 3/3

P39%

3.6

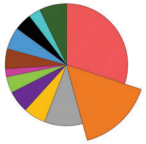

β2-α2 LOOP
SNQNNF
273

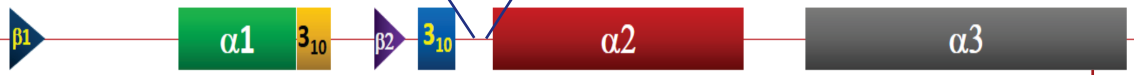

WNKPSKPKTNMKHVAGAAAAGAVVGGLGGYMLGSAMSRPLIHFGNDYEDRYYRENMYRYPNQVYYKPVDQYSNQNNFVQDCVNITIKQHTVTTTTKGENFTETDVKIMERVLQQMCTTQYQKESQAYYYHGRAG-

I M W N R N H M VE V EGRSS

PrP sequence differs by 11 amino acids from the bank vole PrP (see in red)

No deletions
No insertions
No polymorphic variants found

Distinct primary sequence across species

135

202 AA

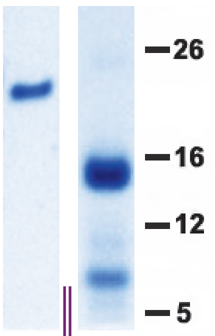

1
Conformers

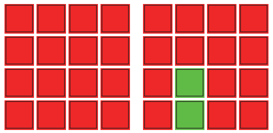

Tm^{Exp}: ND

ΔΔG: ND

In vitro studies

TgVole (1x) Not tested

Plains viscacha rat Not tested

In vivo studies

TgVole (1x) Not tested

Plains viscacha rat Not tested

pLDDT
[83.91]

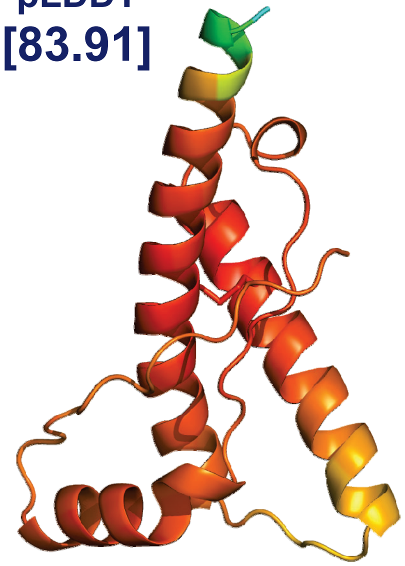

Protein code: 3322

Chinese pygmy dormouse

GenBank: BK064190

P70%

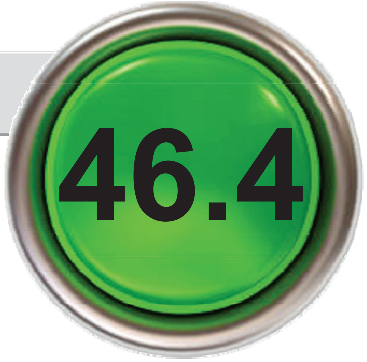

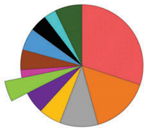

40-50

Typhlomys cinereus

Rodentia	81/101
Platacanthomyidae	1/1

β2-α2 LOOP
NNQNNF
164

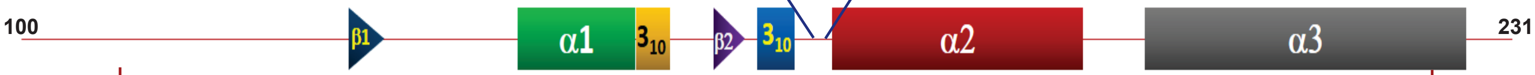

WGKPNKPKTNMKHVAGAAAAGAVVGGLGGYMLGSAMSRPLIHFGNDYEDRYYRENMYRYPNQVYYRPVDQYNNQNNFVHDCVNITVKQHTVTTTTKGENFTETDIKIMERVVEQMCITQYQKESQAYYEGRRS-

N S I M W N I V M V S

PrP sequence differs by 8 amino acids from the bank vole PrP (see in red)

No deletions

No insertions

No polymorphic variants found

Distinct primary sequence across species

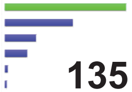

135

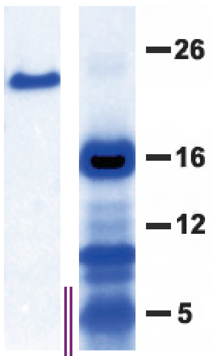

212 AA

1

Conformers

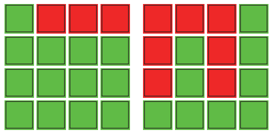

Tm[Exp]: ND

ΔΔG: -13.72

In vitro studies

Not tested
TgVole (1x)

Not tested
Chinese pygmy dormouse

In vivo studies

Not tested
TgVole (1x)

Not tested
Chinese pygmy dormouse

pLDDT
[85.95]

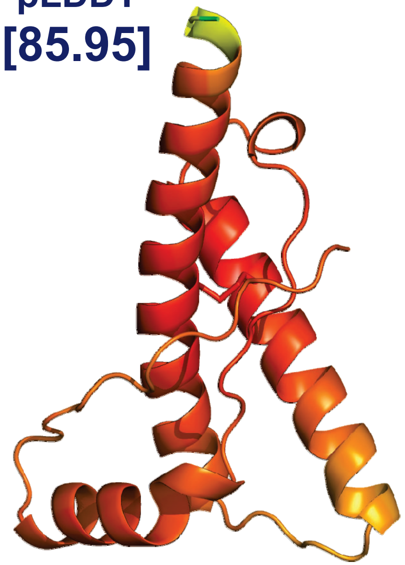

Protein code: 4478

Giant white-tailed rat

GenBank: BK064806

Uromys caudimaculatus

Rodentia	81/101
Muridae	16/18

P81%

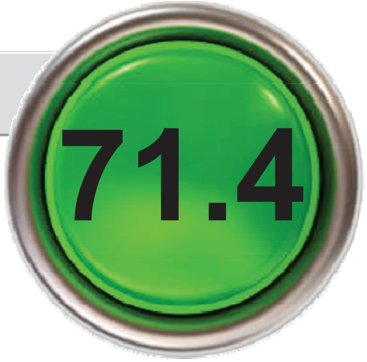

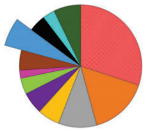

70-80

β2-α2 LOOP
SNQNNF
273

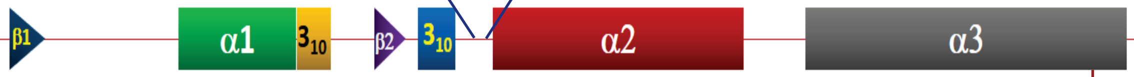

WNKPSKPKTNLKHVAGAAAAGAVVGGLGGYMLGSAMSRPMLHFGNDWEDRYYRENMYRYPNQVYYRPVDQYSNQNNFVHDCVNITIKQHTVTTTTKGENFTETDVKMMERVVEQMCVTQYQKESQAYYDGRRS-

I I N N E S

PrP sequence differs by 4 amino acids from the bank vole PrP (see in red)

No deletions

No insertions

No polymorphic variants found

Distinct primary sequence across species

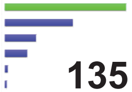

135

219 AA

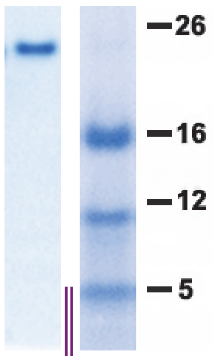

1
Conformers

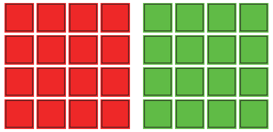

Tm[Exp]: ND

ΔΔG: -9.36

In vitro studies

TgVole (1x) — Not tested

Giant white-tailed rat — Not tested

In vivo studies

TgVole (1x) — Not tested

Giant white-tailed rat — Not tested

pLDDT
[86.38]

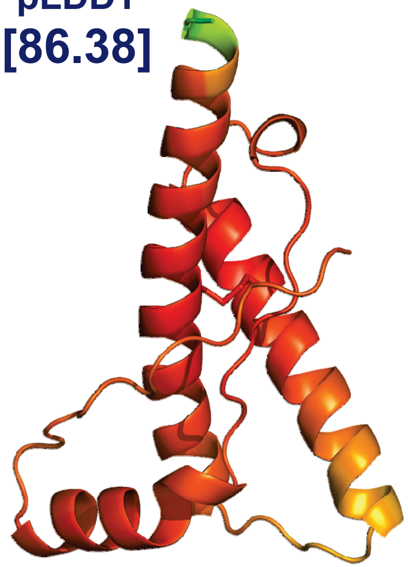

Protein code: 3974

Unstriped ground squirrel

GenBank: BK064797

Xerus rutilus

Rodentia 81/101
Sciuridae 7/13

Distinct primary sequence across species

P73%

53.6

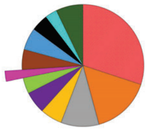

50-60

β2-α2 LOOP
SNQNNF
273

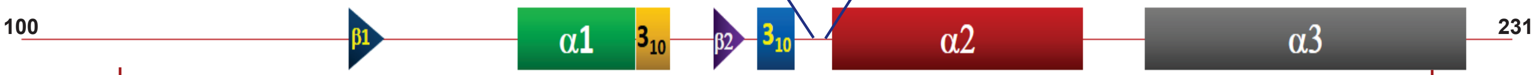

WSKPNKPKTNMKHVAGAAAAGAVVGGLGGYMLGSAMSRPLIHFGNDYEDRYYRENMYRYPNQVYYKPVDQYSNQNNFVHDCVNITVKQHTVTTTTKGENFTETDIKIMERVVEQMCITQYQQEAQAYYQRGSS-
N S I M W N R N I V M V K S EGR

PrP sequence differs by 12 amino acids from the bank vole PrP (see in red)

No deletions
No insertions
No polymorphic variants found

76

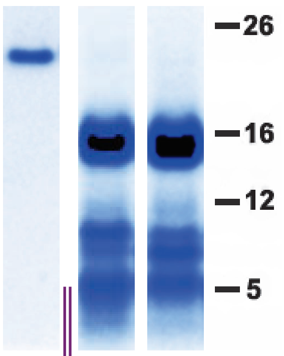

213 AA

2
Conformers

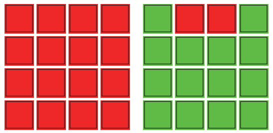

Tm[Exp]: ND
ΔΔG: -13.01

pLDDT
[85.16]

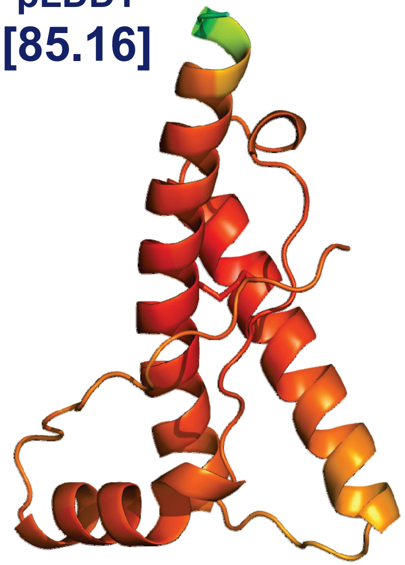

In vitro studies

TgVole (1x) Propagate
Unstriped ground squirrel Not tested

In vivo studies

TgVole (1x) Ongoing
Unstriped ground squirrel Not tested

Protein code: 3962

Meadow jumping mouse

GenBank: BK064139

P42%

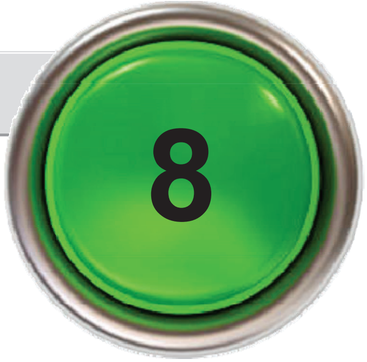

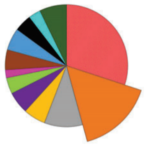

0-10

Zapus hudsonius

Rodentia	81/101
Dipodidae	3/3

β2-α2 LOOP
NNQNNF
164

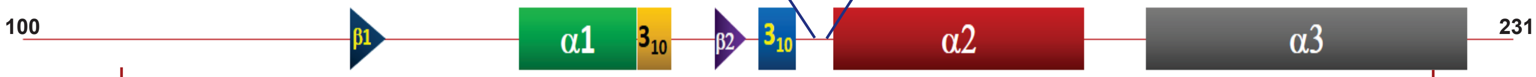

WNKPNKPKTNMKHMAGAAAAGAVVGGLGGYMLGSAMSRPSMSFGNEYEDRYYRENMNRYPSQVYYKPVDQYNNQNNFVHDCVNITVKQHTVTTTTKGENFTETDVKIMERVVEQMCITQYQKEAQAYQEGRRS-

S I V MIH DW N R I M V S Y S

PrP sequence differs by 13 amino acids from the bank vole PrP (see in red)

No deletions

No insertions

No polymorphic variants found

Distinct primary sequence across species

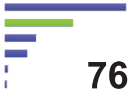

76

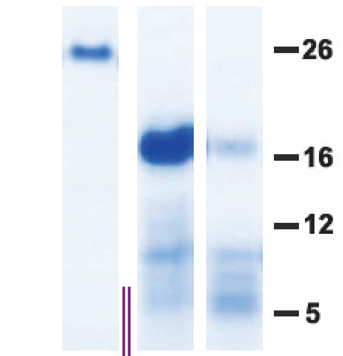

219 AA

2
Conformers

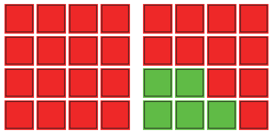

Tm[Exp]: ND

ΔΔG: -9.25

In vitro studies

TgVole (1x) — Not tested

Meadow jumping mouse — Not tested

In vivo studies

TgVole (1x) — Not tested

Meadow jumping mouse — Not tested

pLDDT
[85.07]

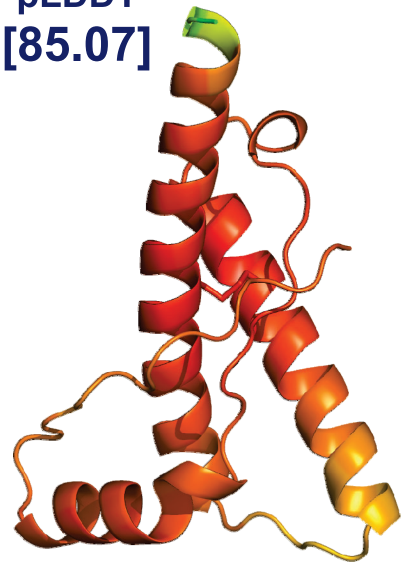

Protein code: 3323

Chinese tree shrew

GenBank: XM_006163978

Tupaia belangeri chinensis

Scandentia 2/2

Tupaiidae 2/2

Distinct primary sequence across species

P82%

71.8

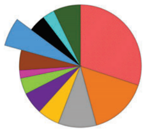

70-80

β2-α2 LOOP
SNQNNF
273

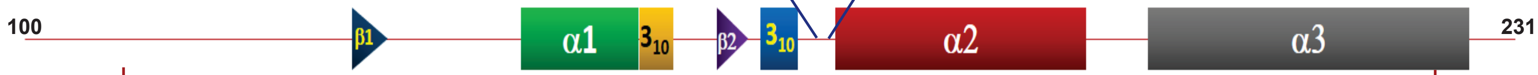

WNKPSKPKTNMKHVAGAAAAGAVVGGLGGYMLGSVMSRPMIHFGSDYEDRYYRENMNRYPNQVYYKPVDQYSNQNNFVHDCVNITIKQHTVTTTTKGENFTETDIKIMERVVEQMCITQYQQEARASYYNSS-

I A N W R N V M V K SQ Y EGR S

PrP sequence differs by 12 amino acids from the bank vole PrP (see in red)

No deletions

No insertions

No polymorphic variants found

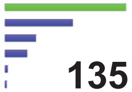

135

rec-PrP

Conformer 1

PK - +

MW (kDa)

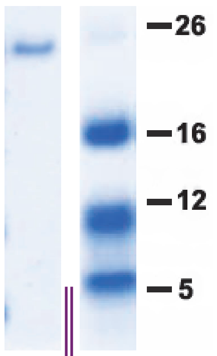

220 AA

1

Conformers

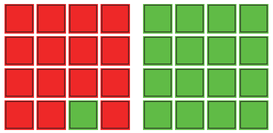

Tm^{Exp}: ND

ΔΔG: ND

In vitro studies

Not tested

TgVole (1x)

Not tested

Chinese tree shrew

In vivo studies

Not tested

TgVole (1x)

Not tested

Chinese tree shrew

pLDDT [93.79]

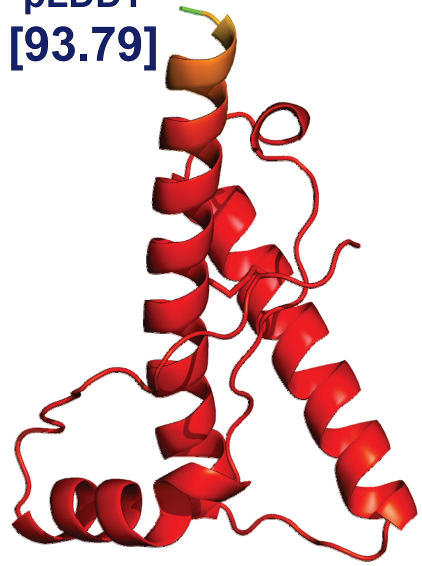

Protein code: 1093

Large treeshrew

GenBank: AY133035

Tupaia tana

Scandentia 2/2

Tupaiidae 2/2

P89%

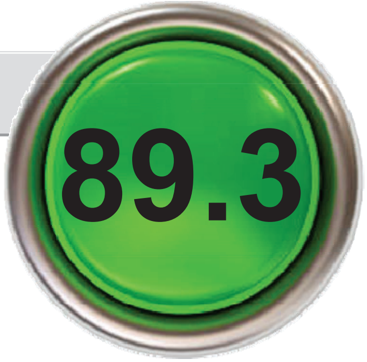

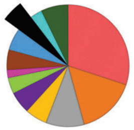

80-90

β2-α2 LOOP
SNQNNF
273

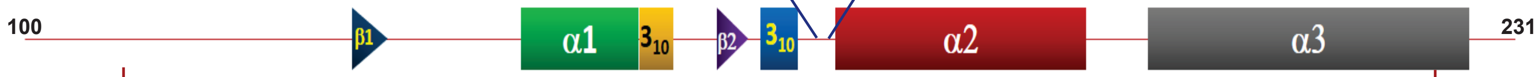

WNKPSKPKTNMKHVAGAAAAGAVVGGLGGYMLGSVMSRPMIHFGSDYEDRYYRENMNRYPNQVYYKPVDQYSNQNNFVHDCVNITIKQHTVTTTTKGENFTETDIKIMERVVEQMCITQYQQEARASYYSSS-

I A N W R N V M V K SQ Y EGR S

PrP sequence differs by 12 amino acids from the bank vole PrP (see in red)

No deletions

No insertions

No polymorphic variants found

Distinct primary sequence across species

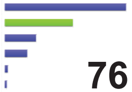

76

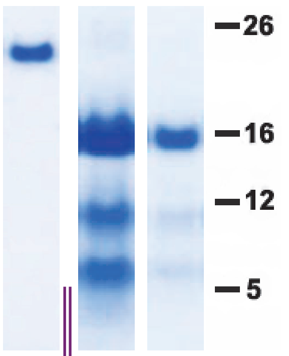

220 AA

2
Conformers

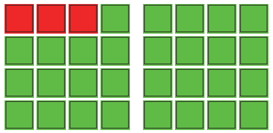

Tm^{Exp}: ND

ΔΔG: ND

In vitro studies

TgVole (1x) Not tested

Large treeshrew Not tested

In vivo studies

TgVole (1x) Not tested

Large treeshrew Not tested

pLDDT
[94.29]

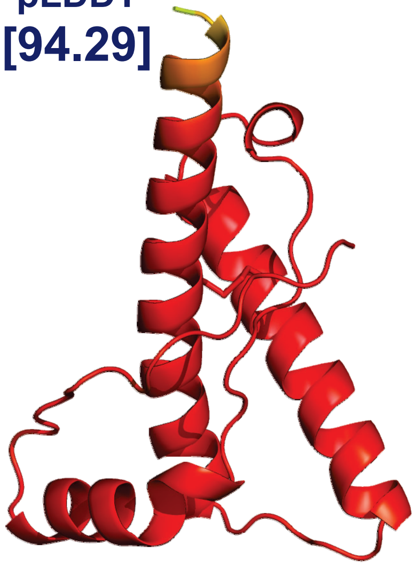

Protein code: 1371

Dugong

GenBank: BK063981

Dugong dugon

Sirenia 1/2

Dugongidae 0/1

1 species sharing the same primary sequence

Steller's sea cow
Hydrodamalis gigas

P29%

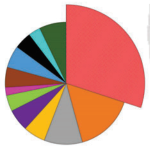

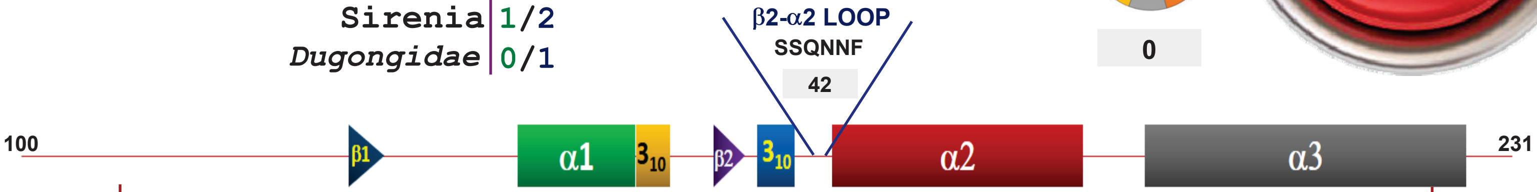

WNKPSKPKTNMKHVAGAAAAGAVVGGLGGYMLGSAMSRPLIHFGNDYEDRYYRENMNRYPNQVYYRPVNEYSSQNNFVHDCVNITVKQHTVTTTTKGENFTETDVKIMERVVEQMCITQYEKEFQAYQRGSS-
I M W DQ NN I M V Q S YE R S

PrP sequence differs by 12 amino acids from the bank vole PrP (see in red)

No deletions

No insertions

No polymorphic variants found

Tm^{Exp}: ND

ΔΔG: ND

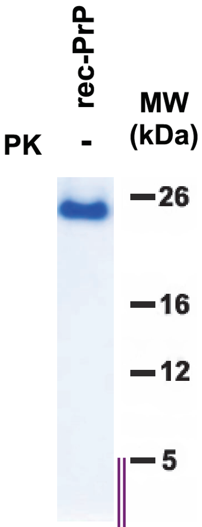

220 AA

NO MISFOLDING

0

Conformers

In vitro studies

NOT APPLICABLE

In vivo studies

NOT APPLICABLE

pLDDT

[88.27]

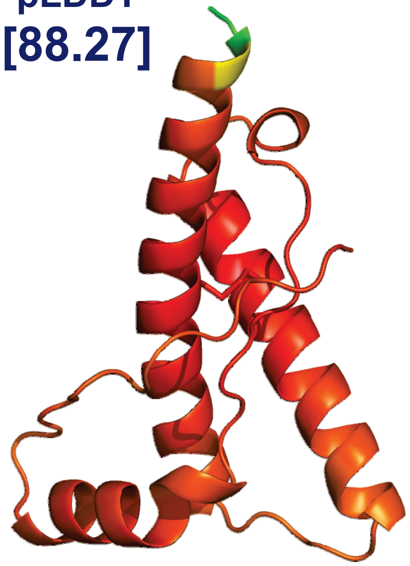

Protein code: 3138

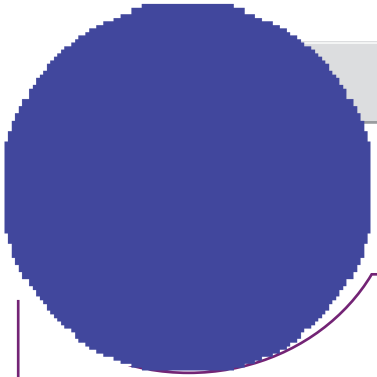

West Indian manatee

GenBank: AY133056

Trichechus manatus

Sirenia 1/2
Trichechidae 1/1

P45%

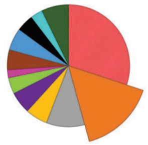

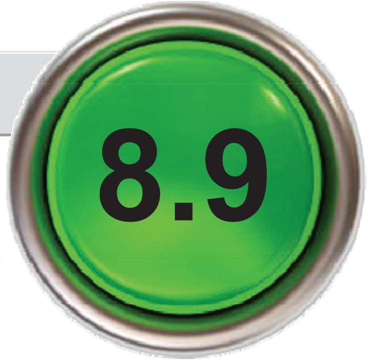

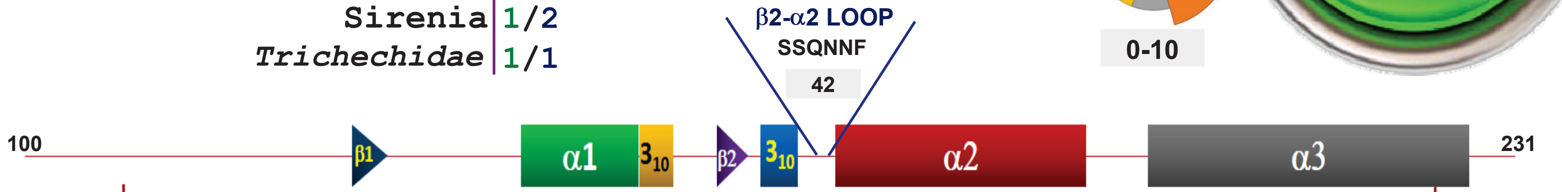

WSKPSKPKTNMKHVAGAAAAGAVVGGLGGYMLGSAMSRPLIHFGNDYEDRYYRENMNRYPNQVYYRPVNEYSSQNNFVHDCVNITVKQHTVTTTTKGENFTETDVKIMERVVEQMCITQYEKEFQAYQRGSS-

N I M W DQ NN I M V Q S YE R S

PrP sequence differs by 12 amino acids from the bank vole PrP (see in red)

No deletions
No insertions
No polymorphic variants found

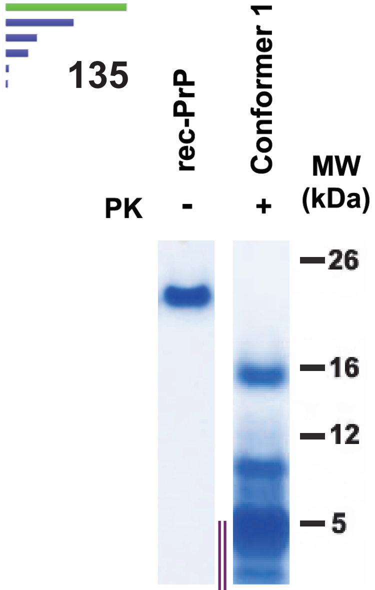

220 AA

1
Conformers

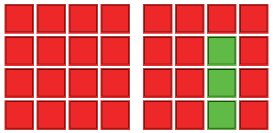

Distinct primary sequence across species

Tm^{Exp}: ND

ΔΔG: ND

In vitro studies

TgVole (1x) Not tested

West Indian manatee Not tested

In vivo studies

TgVole (1x) Not tested

West Indian manatee Not tested

pLDDT [88.15]

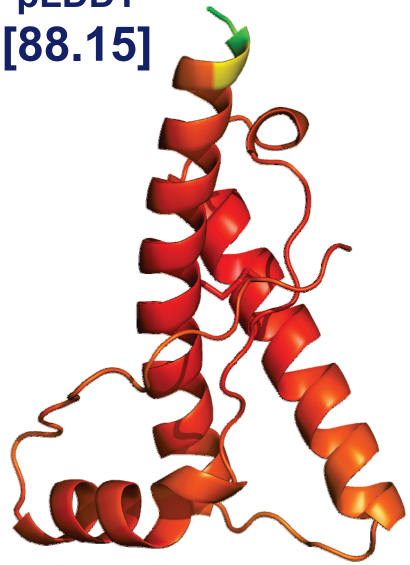

Star-nosed mole

GenBank: XM_012728373

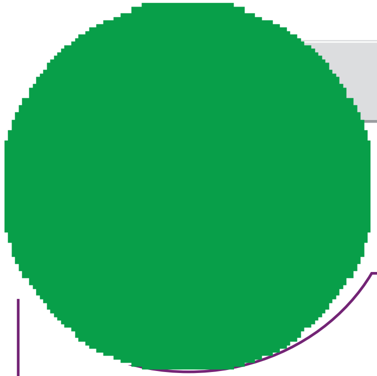

Condylura cristata

Soricomorpha 7/12
Talpidae 4/6

P36%

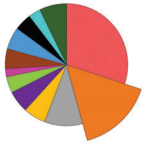

0-10

3.2

β2-α2 LOOP
NNQNNF
164

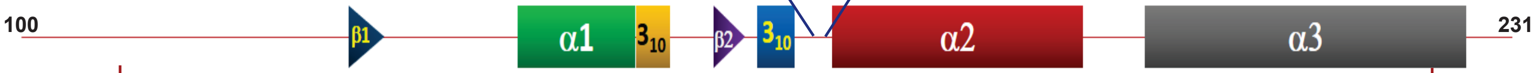

WNKPNKPKTNMKHVAGAAAAGAVVGGLGGYMLGSAMSRPLMHFGNDYEDRYYRENMNRYPNQVYYKPVDQYNNQNNFVHDCVNITVKQHTVTTTTQGENFTETDIKIMERVVEQMCINQYQKEYQSSYQRGSS-
S I MI W R I V M VT S AY EGR

PrP sequence differs by 12 amino acids from the bank vole PrP (see in red)

No deletions
No insertions
No polymorphic variants found

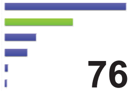

76

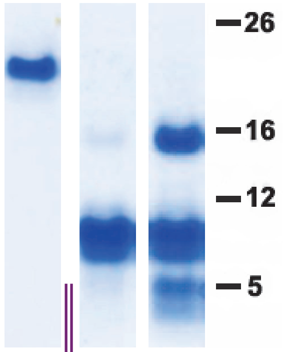

216 AA

2
Conformers

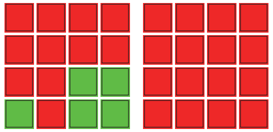

Tm^{Exp}: ND
ΔΔG: -0.07

In vitro studies

TgVole (1x) Not tested
Star-nosed mole Not tested

In vivo studies

TgVole (1x) Not tested
Star-nosed mole Not tested

pLDDT
[84.56]

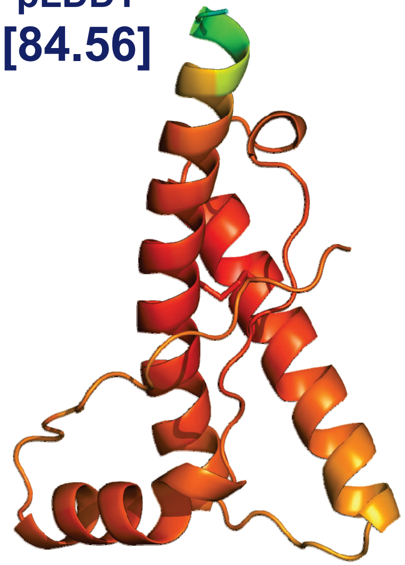

Distinct primary sequence across species

Greater white-toothed shrew

GenBank: OR47280

Crocidura russula

Soricomorpha 7/12

Soricidae 2/5

P29%

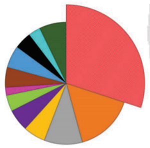

0

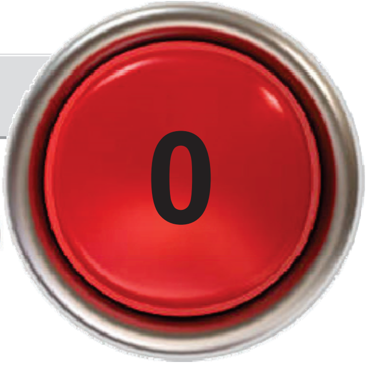

β2-α2 LOOP

NNQNNF

164

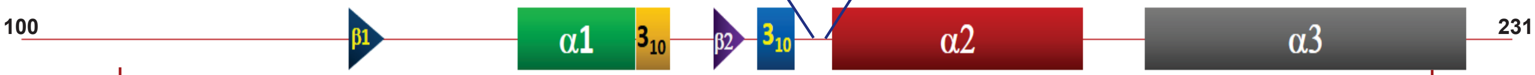

WNKPSKPKTNMKHVAGAAAAGAVVGGLGGYMLGSAMSRPLIHFDNDYENRYYRENMNRYPNQVYYKPVDQYNNQNNFVHDCVNITIKQHTVTTTSKGENFTETDIKIMERVVEQMCITQYQDASRAYYQQGTG-

I M G WD R T V M V KE Q EGRSS

PrP sequence differs by 13 amino acids from the bank vole PrP (see in red)

No deletions

No insertions

No polymorphic variants found

Distinct primary sequence across species

Tm^{Exp}: ND

ΔΔG: -18.73

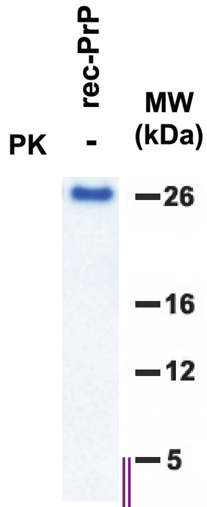

233 AA

NO MISFOLDING

0

Conformers

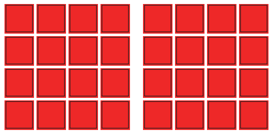

In vitro studies

NOT APPLICABLE

In vivo studies

NOT APPLICABLE

pLDDT [91.1]

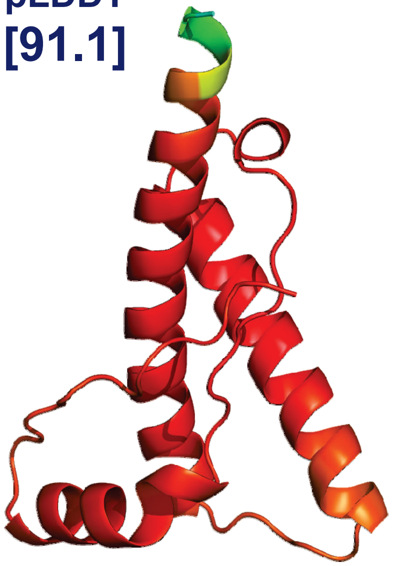

Protein code: 4606

Pyrenean desman

GenBank: BK063927

P51%

16.6

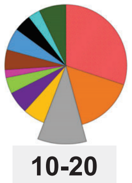

Galemys pyrenaicus

Soricomorpha 7/12
Talpidae 4/6

β2-α2 LOOP
NNQNNF
164

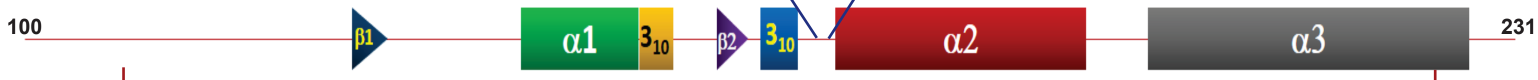

WNKPSKPKTNMKHVAGAAAAGAVVGGLGGYMLGSAMSRPLVHFGNDYEDRYYRENMNRYPNQVYYKPVDQYNNQNNFVHDCVNITVKQHTVTTTTQGENFTETDIKIMERVVEQMCITQYQKEYQASYQRGSS-

I MI W R I V M V S Y EGR

PrP sequence differs by 10 amino acids from the bank vole PrP (see in red)

No deletions
No insertions
No polymorphic variants found

Distinct primary sequence across species

33

215 AA

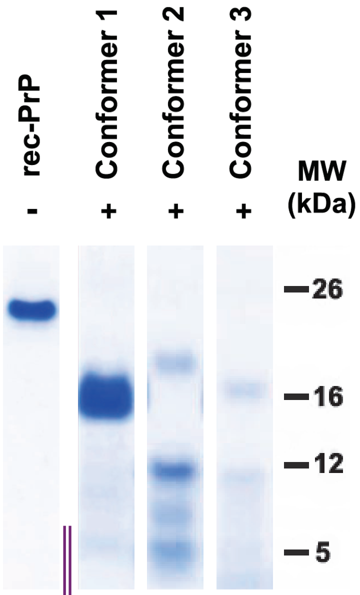

3 Conformers

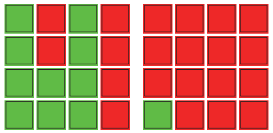

Tm[Exp]: ND

ΔΔG: -9.42

pLDDT [84.45]

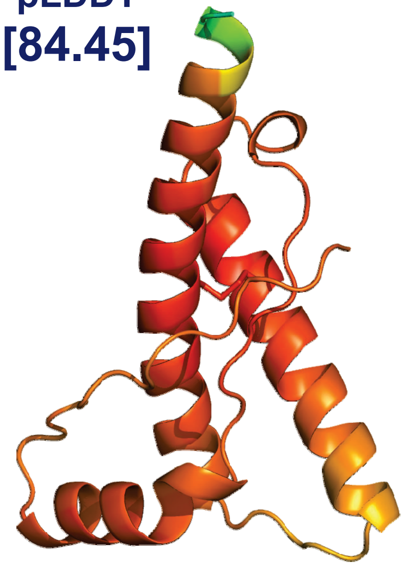

In vitro studies

Not tested
TgVole (1x)

Not tested
Pyrenean desman

In vivo studies

Not tested
TgVole (1x)

Not tested
Pyrenean desman

Protein code: 2562

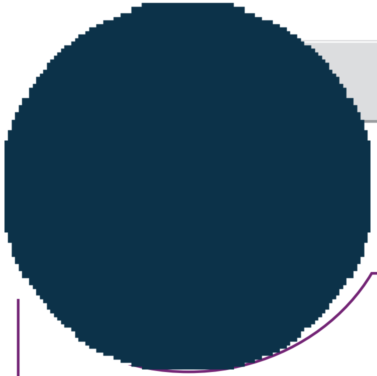

Eastern mole

GenBank: BK063985

Scalopus aquaticus

Soricomorpha 7/12
Talpidae 4/6

P29%

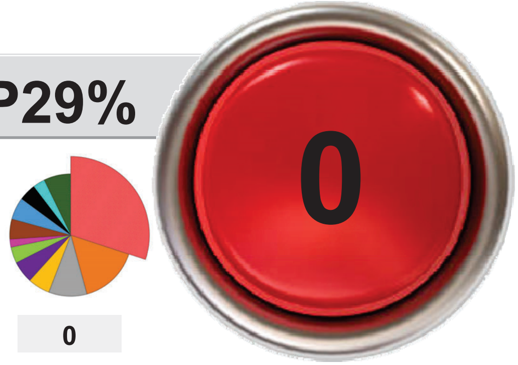

0

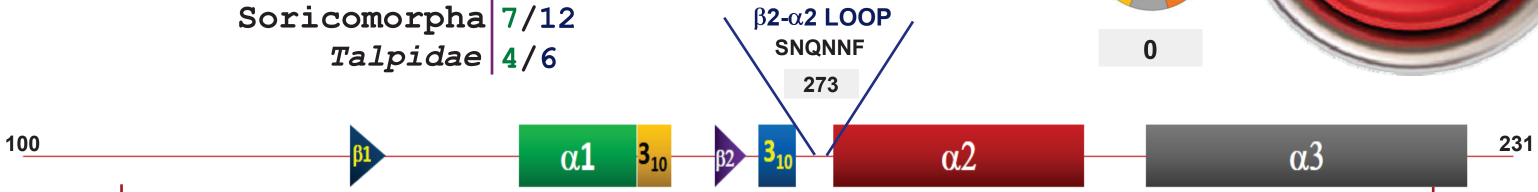

WNKPSKPKTNMKHVAGAAAAGAVVGGLGGYMLGSAMSRPLMHFDSDYENRYYRENMNRYPNQVYYKPVDQYSNQNNFVHDCVNITVKQHTVTTTTQGENFTETDIKIMERVVEQMCITQYQKEYQSAYQRGAS-

I MI GN WD R N I V M V S AY EGRS

PrP sequence differs by 15 amino acids from the bank vole PrP (see in red)

No deletions
No insertions
No polymorphic variants found

Distinct primary sequence across species

Tm^{Exp}: ND

ΔΔG: -8.06

PK -

rec-PrP

MW (kDa)

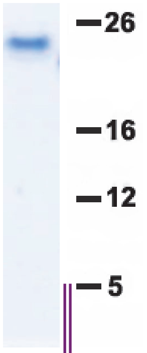

224 AA

NO MISFOLDING

0
Conformers

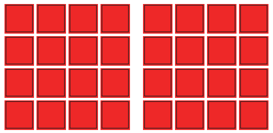

In vitro studies

NOT APPLICABLE

In vivo studies

NOT APPLICABLE

pLDDT
[86.98]

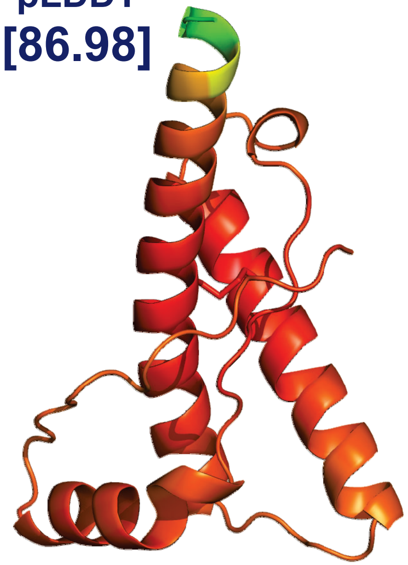

Protein code: 3142

Hispaniolan solenodon

GenBank: BK063984

Solenodon paradoxus woodi

Soricomorpha 7/12

Solenodontidae 1/1

P45%

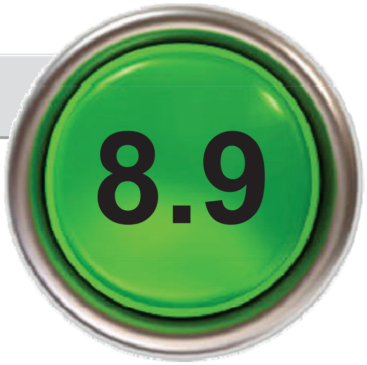

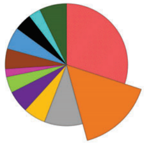

0-10

β2-α2 LOOP

SSQNNF

42

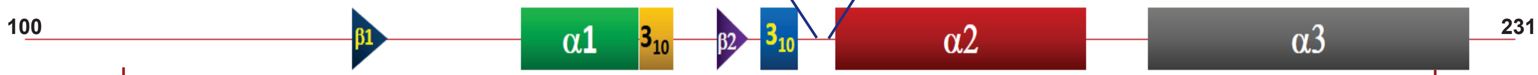

WNKPSKPKTNMKHVAGAAAAGAVVGGLGGYLVGSAMSRPMLHFGNDYEDRYYRENMYRYPSQVYYKPMDQYSSQNNFVHDCVNITIKQHTVTTTTKGENFTETDIKMMERVVEQMCTTQYQREYQSFSQRGAS-

I ML I W N N R V NN V V K S AYYEGRS

PrP sequence differs by 16 amino acids from the bank vole PrP (see in red)

No deletions

No insertions

No polymorphic variants found

Distinct primary sequence across species

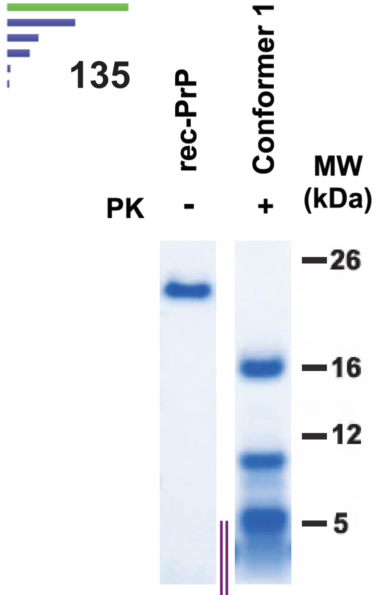

220 AA

1

Conformers

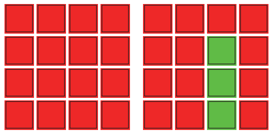

Tm^{Exp}: ND

ΔΔG: -8.12

In vitro studies

Not tested

TgVole (1x)

Not tested

Hispaniolan solenodon

In vivo studies

Not tested

TgVole (1x)

Not tested

Hispaniolan solenodon

pLDDT

[85.01]

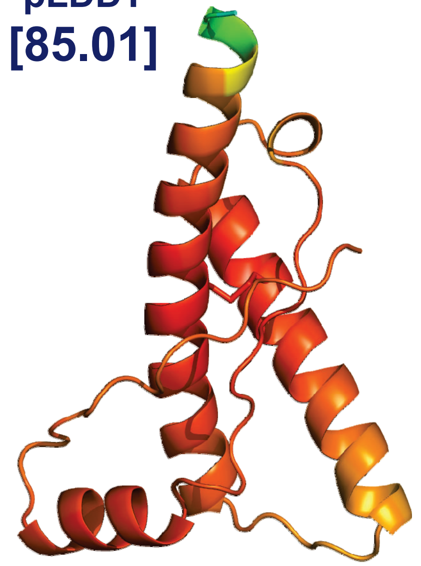

Protein code: 3141

Common shrew

GenBank: BK064940

P53%

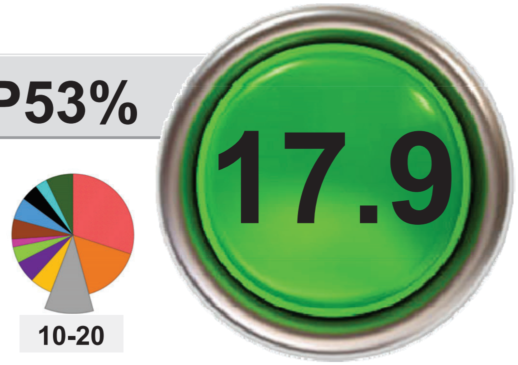

Sorex araneus

Soricomorpha 7/12
Soricidae 2/5

1 species sharing the same primary sequence

Maritime shrew
Sorex maritimensis

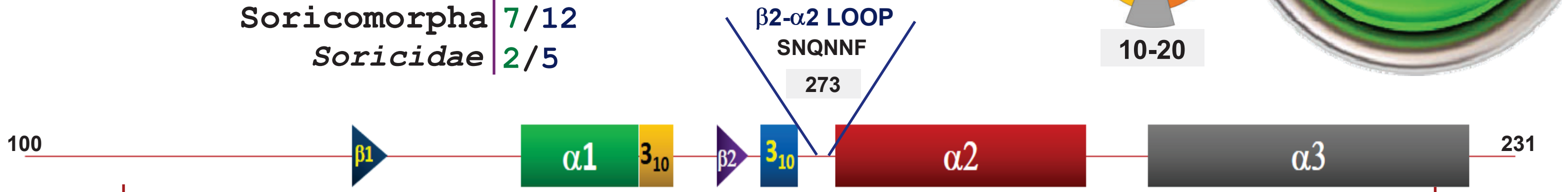

WNKPSKPKTNMKHVAGAAAAGAVVGGLGGYMLGSAMSRPLMHFGNDYEDRYYRENMYRYPNQVYYKPVDQYSNQNNFVHDCVNMTVKQHTVTTTSKGENFTETDVKIMERVVEQMCITQYREAAQASYLQGDS-

I MI W N R N I I T M V QKES Y EGRS

PrP sequence differs by 16 amino acids from the bank vole PrP (see in red)

No deletions
No insertions
No polymorphic variants found

135

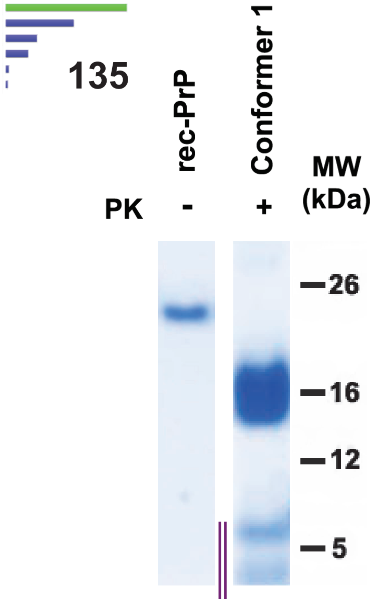

213 AA

1
Conformers

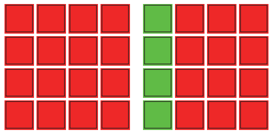

Tm^{Exp}: ND

ΔΔG: -14.08

In vitro studies

TgVole (1x) Not tested

Common shrew Not tested

In vivo studies

TgVole (1x) Not tested

Common shrew Not tested

pLDDT
[87.34]

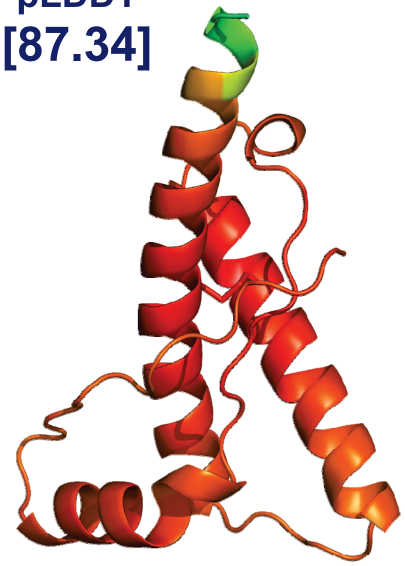

Protein code: 0976

Cinereous shrew

GenBank: BK063915

Sorex cinereus

Soricomorpha 7/12
Soricidae 2/5

1 species sharing the same primary sequence

Smoky shrew
Sorex fumeus

P29%

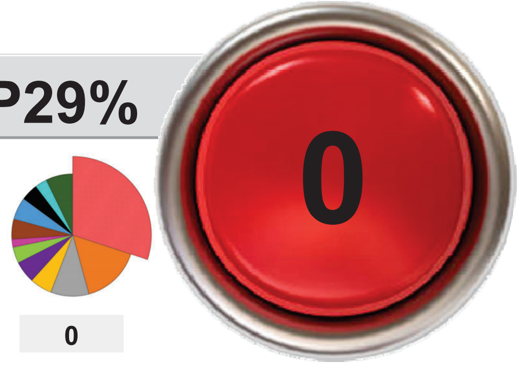

0

β2-α2 LOOP
SNQNNF
273

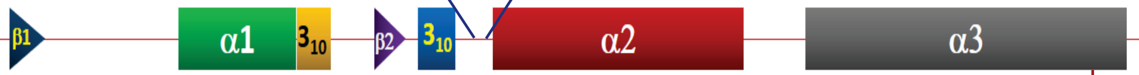

WNKPSKPKTNMKHVAGAAAAGAVVGGLGGYMLGSAMSRPLMHFGNDYEDRYYRENMYRYPNQVYYYKPVDQYSNQNNFVHDCVNMTVKQHTVTTTSKGENFTETDVKIMERVVEQMCITQYREAAQASYMQGYN-

I MI W N R N I I T M V QKES Y EGRSS

PrP sequence differs by 16 amino acids from the bank vole PrP (see in red)

No deletions
No insertions
No polymorphic variants found

Tm[Exp]: ND

ΔΔG: -4.11

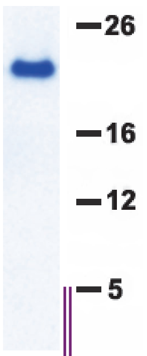

212 AA

NO MISFOLDING

0 Conformers

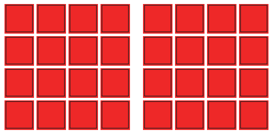

In vitro studies

NOT APPLICABLE

In vivo studies

NOT APPLICABLE

pLDDT [93.25]

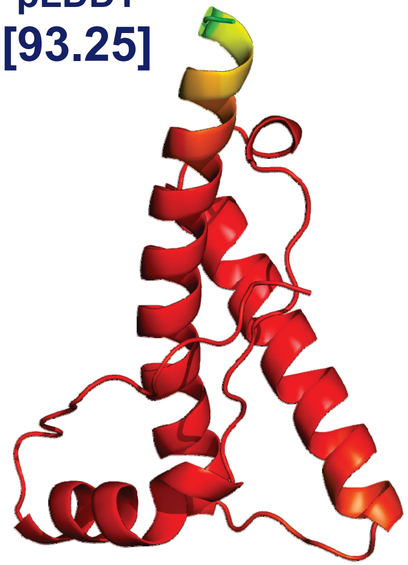

Protein code: 1352

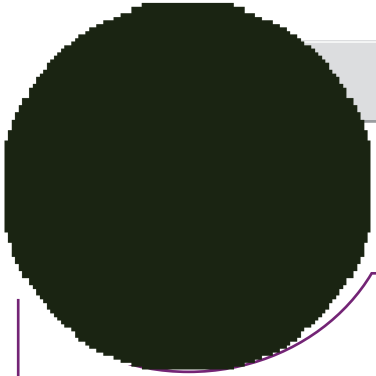

American water shrew

GenBank: BK064220

Sorex palustris

Soricomorpha 7/12

Soricidae 2/5

Distinct primary sequence across species

P29%

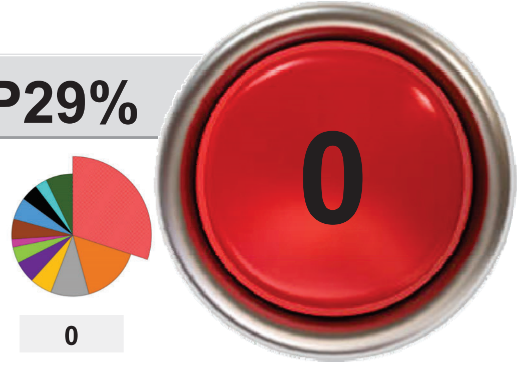

β2-α2 LOOP
SNQNNF
273

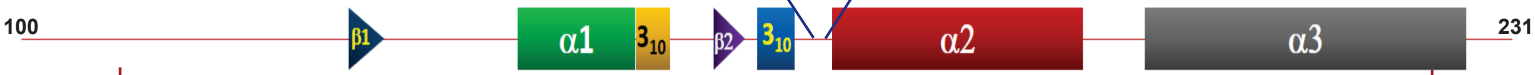


```
WNKPSKPKTNMKHVAGAAAAGAVVGGLGGYMLGSAMSRPLMHFGNDYEDRYYRENMYRYPNQVYYKPVDQYSNQNNFVHDCVNMTVKQHTVTTTSKGENFTETDVKIMERVVEQMCITQYREAAQASYMQGYS-
          I                               MI    W         N        R     N           I I       T           M         V   QKES  Y EGRS
```

PrP sequence differs by 16 amino acids from the bank vole PrP (see in red)

No deletions

No insertions

No polymorphic variants found

Tm^{Exp}: ND

ΔΔG: -9.15

rec-PrP

PK -

MW (kDa)

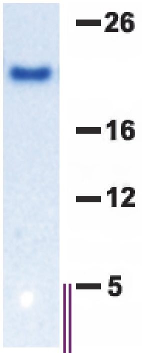

212 AA

NO MISFOLDING

0

Conformers

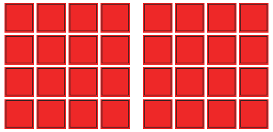

In vitro studies

NOT APPLICABLE

In vivo studies

NOT APPLICABLE

pLDDT

[92.83]

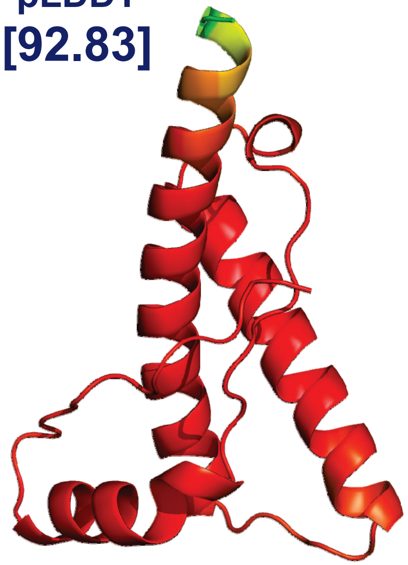

Protein code: 4817

Etruscan shrew

GenBank: BK064203

P69%

44.6

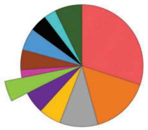

40-50

Suncus etruscus

Soricomorpha 7/12

Soricidae 2/5

β2-α2 LOOP
NNQNNF
164

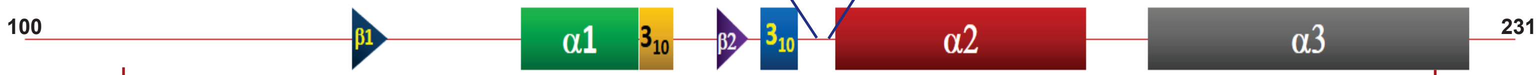

WNKPSKPKTNMKHVAGAAAAGAVVGGLGGYMLGSAMSRPLIHFDNDYENRYYRDNMNRYPNQVYYKPVDQYNNQNNFVHDCVNITIKQHTVTTTSKGENFTETDIKIMERVVEQMCITQYQDASRAYYQQAAG-

I M G WD E R T V M V KE Q EGRSS

PrP sequence differs by 14 amino acids from the bank vole PrP (see in red)

No deletions

No insertions

No polymorphic variants found

76

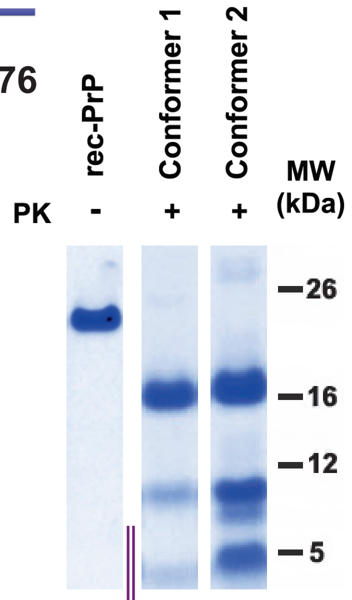

215 AA

2

Conformers

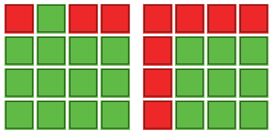

Tm[Exp]: ND

ΔΔG: -3.98

pLDDT
[87.71]

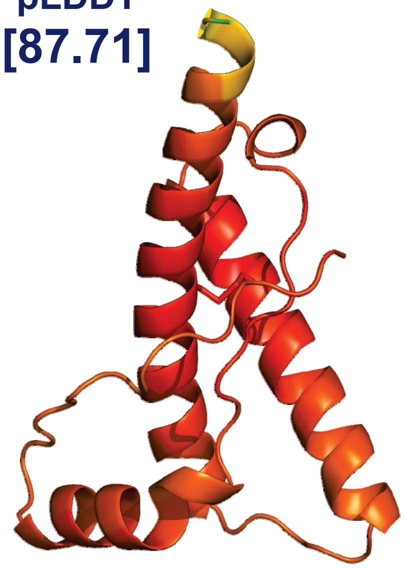

In vitro studies

TgVole (1x) Not tested

Etruscan shrew Not tested

In vivo studies

TgVole (1x) Not tested

Etruscan shrew Not tested

Distinct primary sequence across species

Protein code: 4534

European mole

GenBank: AY133042

P54%

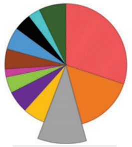

10-20

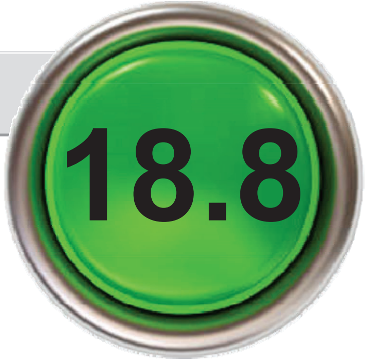

Talpa europaea

Soricomorpha 7/12

Talpidae 4/6

β2-α2 LOOP
NNQNNF
164

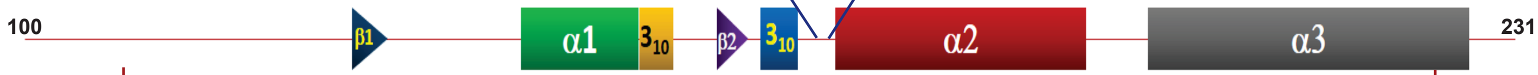

WNKPSKPKTNMKHVAGAAAAGAVVGGLGGYMLGSAMSRPLMHFGNEYEDRYYRDNMNRYPNQVYYKPVDQYNNQNNFVHDCVNITVKQHTVTTTTKGENFTETDIKIMERVVEQMCITQYQKEYQASYQRGAS-

I MI DW E R I V M V S Y EGRS

PrP sequence differs by 12 amino acids from the bank vole PrP (see in red)

No deletions

No insertions

No polymorphic variants found

135

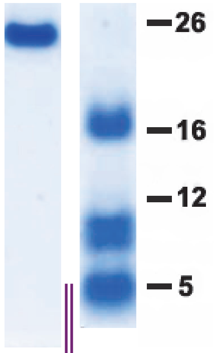

224 AA

1

Conformers

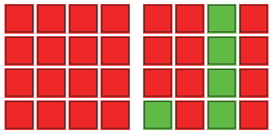

Tm^{Exp}: ND

ΔΔG: -2.87

In vitro studies

TgVole (1x) Not tested

European mole Not tested

In vivo studies

TgVole (1x) Not tested

European mole Not tested

pLDDT

[84.95]

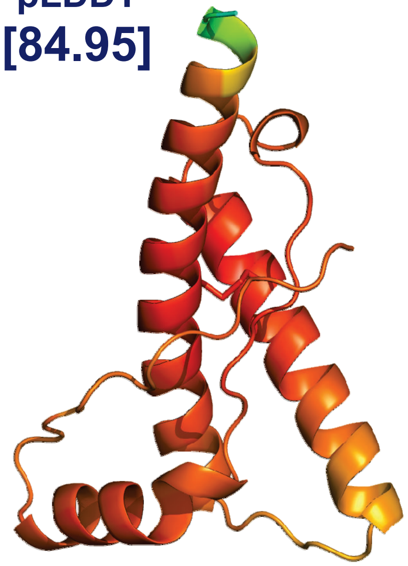

Distinct primary sequence across species

Spanish mole

GenBank: BK063986

Talpa occidentalis

Soricomorpha 7/12
Talpidae 4/6

Distinct primary sequence across species

P57% **22.9**

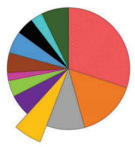

20-30

β2-α2 LOOP
NNQNNF
164

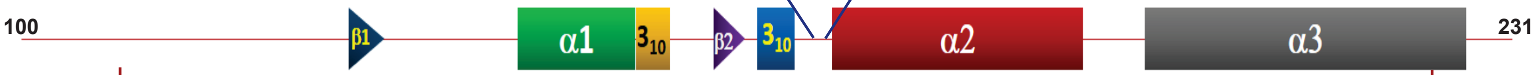

WNKPSKPKTNMKHVAGAAAAGAVVGGLGGYMLGSAMSRPLMHFGNEYEDRYYRENMNRYPNQVYYKPVDQYNNQNNFVHDCVNITVKQHTVTTTTKGENFTETDIKIMERVVEQMCITQYQKEYQASYQRGAS-

I MI DW R I V M V S Y EGRS

PrP sequence differs by 11 amino acids from the bank vole PrP (see in red)

No deletions
No insertions
No polymorphic variants found

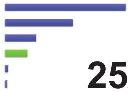

25

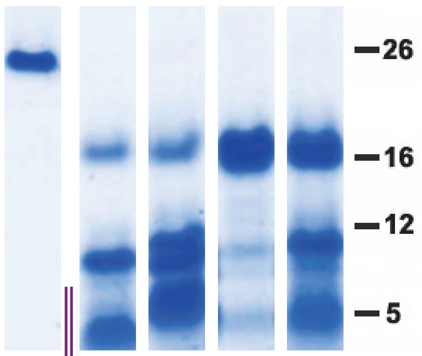

224 AA

4 Conformers

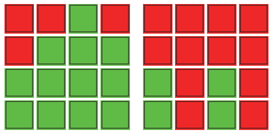

Tm^{Exp}: ND

ΔΔG: -3.35

In vitro studies

TgVole (1x) Not tested

Spanish mole Not tested

In vivo studies

TgVole (1x) Not tested

Spanish mole Not tested

pLDDT
[85.06]

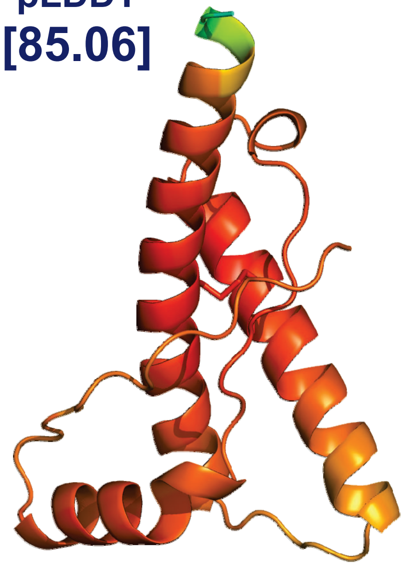

Protein code: 3143

Gracile shrew mole

GenBank: BK063987

Uropsilus gracilis

Soricomorpha 7/12
Talpidae 4/6

P29%

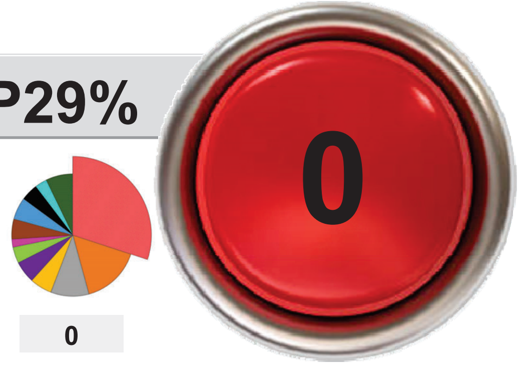

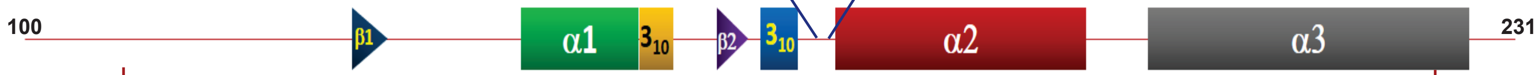

WNKPNKPKTNMKHVAGAAAAGAVVGGLGGYMLGSAMSRPLIHFGNDYEDRYYRENMNRYPNQVYYRPVDQYNNQNNFVHDCVNITVKQHTVTTTTKGENFTETDIKIMERVVEQMCINQYQKEYQASYQRDAS-

S I M W I V M VT S Y EGRS

PrP sequence differs by 9 amino acids from the bank vole PrP (see in red)

No deletions
No insertions
No polymorphic variants found

Distinct primary sequence across species

Tm^{Exp}: ND

ΔΔG: -5.6

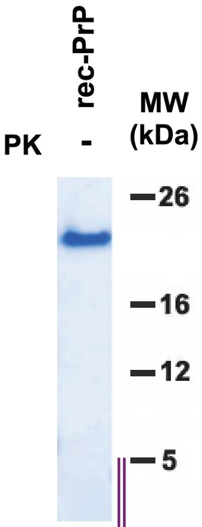

215 AA

NO MISFOLDING

0
Conformers

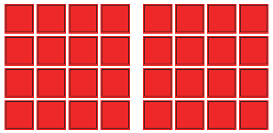

In vitro studies

NOT APPLICABLE

In vivo studies

NOT APPLICABLE

pLDDT
[87.19]

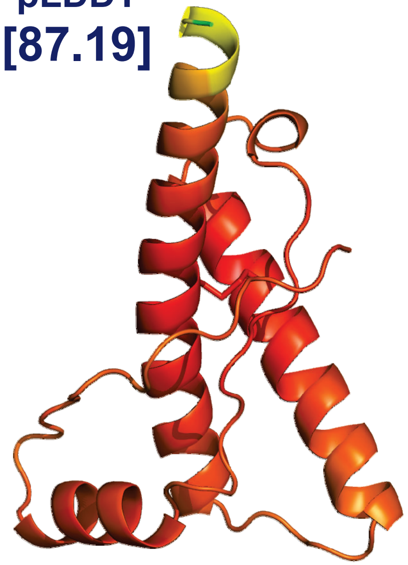

Protein code: 3144

Aardvark

GenBank: AY133058

P29%

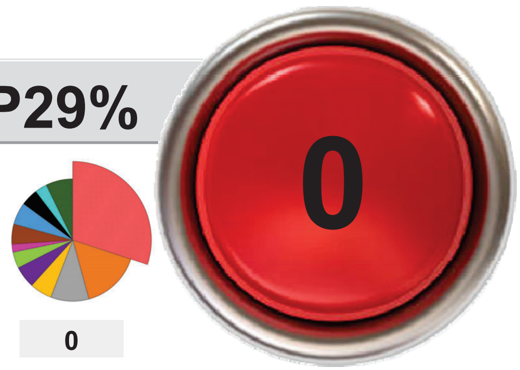

Orycteropus afer

Tubulidentata 0/1
Orycteropodidae 0/1

β2-α2 LOOP
SSENNF
8

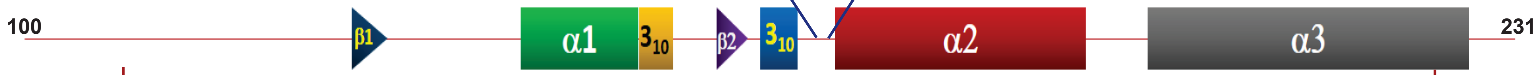


```
WNKPSKPKTNMKHVAGAAAAGAVVGGLGGYMLGSAMSRPLIHFGNDYEDRYYRENMYRYPNQVYYYRPVNEYSSENNFVHDCVNITVKQHTTTTTTKGENFTETDIKIMERVVEQMCMTQYQKEFQAYQRGSS-
           I                               M      W          N           DQ  NNQ               I    V                 V M           V      S    YE R S
```

PrP sequence differs by 15 amino acids from the bank vole PrP (see in red)

No deletions
No insertions
No polymorphic variants found

Distinct primary sequence across species

Tm^{Exp}: ND
ΔΔG: ND

PK
rec-PrP
-
MW (kDa)

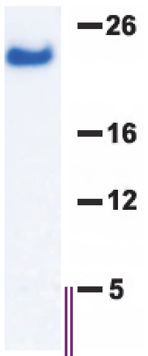

212 AA

NO MISFOLDING

0
Conformers

In vitro studies

NOT APPLICABLE

In vivo studies

NOT APPLICABLE

pLDDT
[92.56]

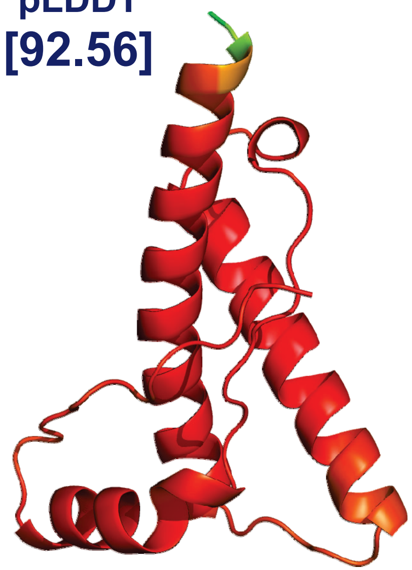

Protein code: 4930
